# Supplementary material for: Unusual KIE and dynamics effects in the Fe-catalyzed hetero-Diels-Alder reaction of unactivated aldehydes and dienes
Source: Nat Commun. 2020 Apr 15;11:1850. doi: 10.1038/s41467-020-15599-w (PMC7160212; doi:10.1038/s41467-020-15599-w)
Supplement: Supplementary file 1 — Supplementary Information [file 41467_2020_15599_MOESM1_ESM.pdf]

Supplementary Information

Unusual KIE and Dynamics Effects in the Fe-catalyzed Hetero-Diels-Alder Reaction of Unactivated Aldehydes and Dienes

Yang et al

Contents

Supplementary Figures **S1**

Supplementary Tables **S32**

Supplementary Methods **S71**

Supplementary Disscussion **S76**

Supplementary References **S81**

Supplementary Figures

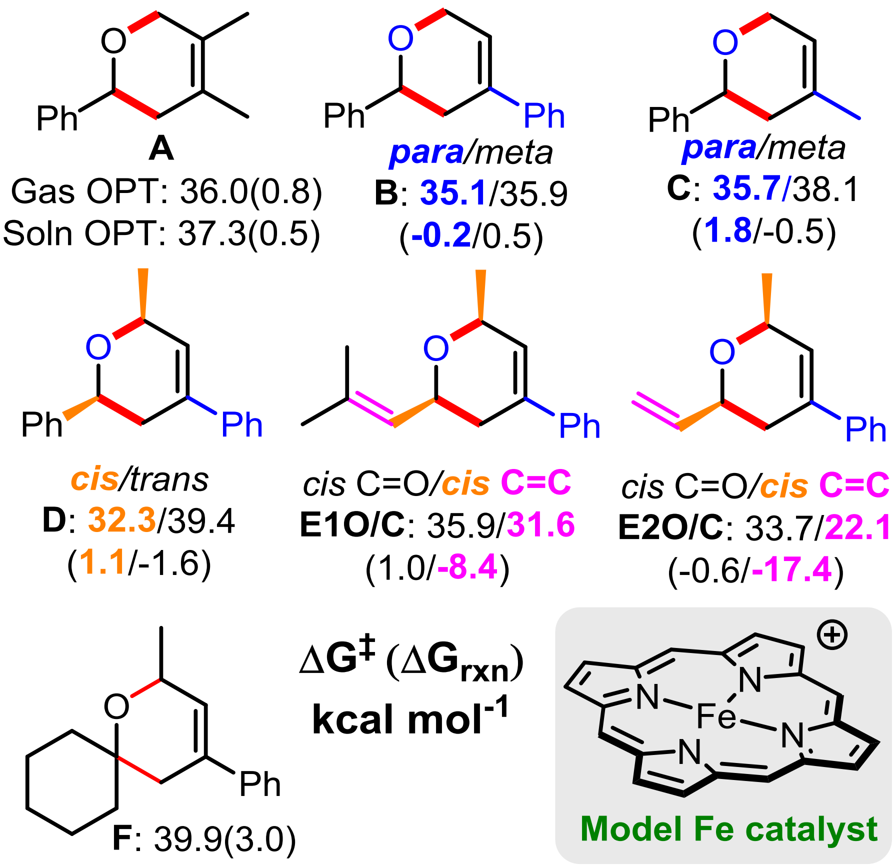

Supplementary Figure 1. **Reaction barriers and reaction energies to form A without Fe catalyst.** The computed free-energy barriers ($\Delta G^{\ddagger}$) and reaction energies (ΔG_{rxn}, in parentheses) are for the uncatalyzed *endo*-ODA reaction of several substrates in solution at 353.15 K by the SMD B3LYP-D3//B3LYP-D3 method (in kcal mol^{-1}). The computed reaction barriers and reaction energies to form **A** in solution optimized by the SMD B3LYP-D3 method (denoted Soln OPT) are also given. Notably, the standard state correction was applied here.

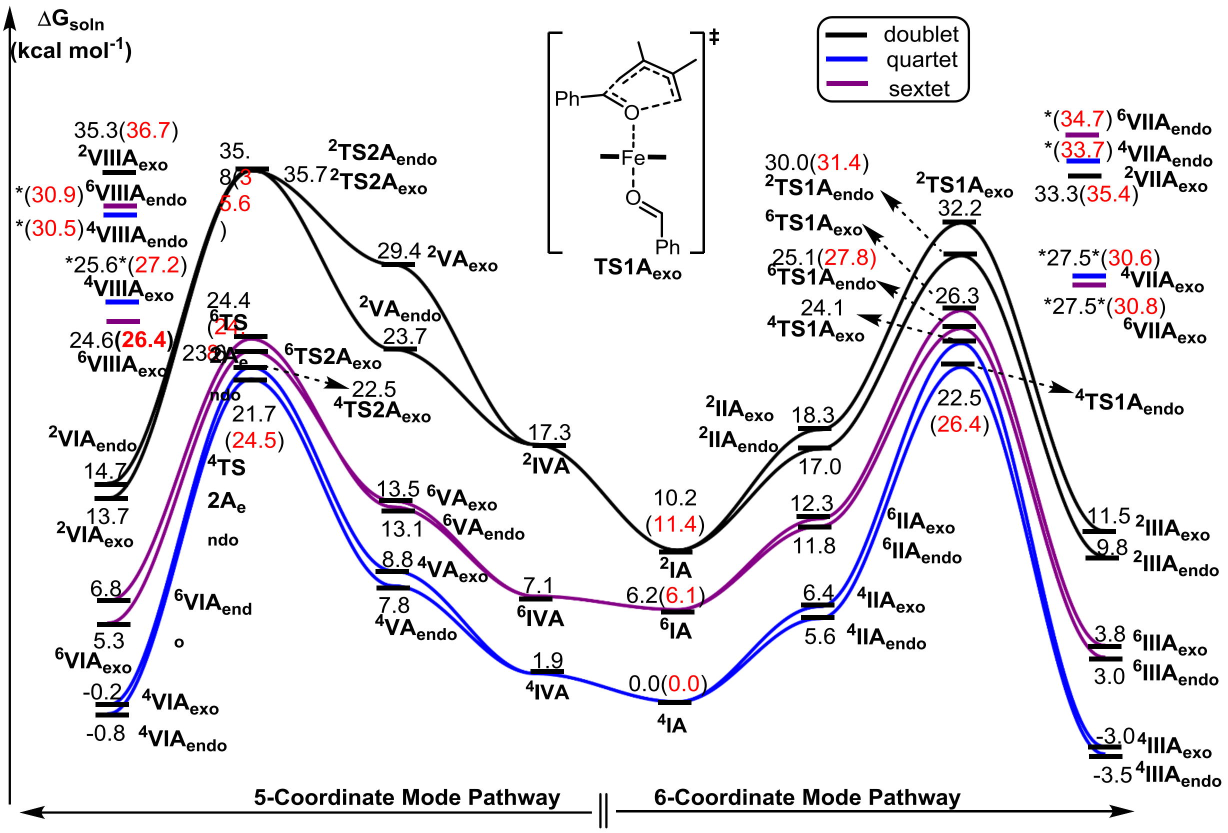

(*The carbon-carbon bond was fixed for optimization)

Supplementary Figure 2. Free energy profile of the ODA reaction to form A. Their relative free energy in three spin states in solution at 353.15 K by the SMD B3LYP-D3//B3LYP-D3 and SMD B3LYP-D3 (in parenthesis) methods are given**.** The optimized *exo*-type structures are given in the Supplementary Figure 3A. Notably, the standard state correction was not applied here.

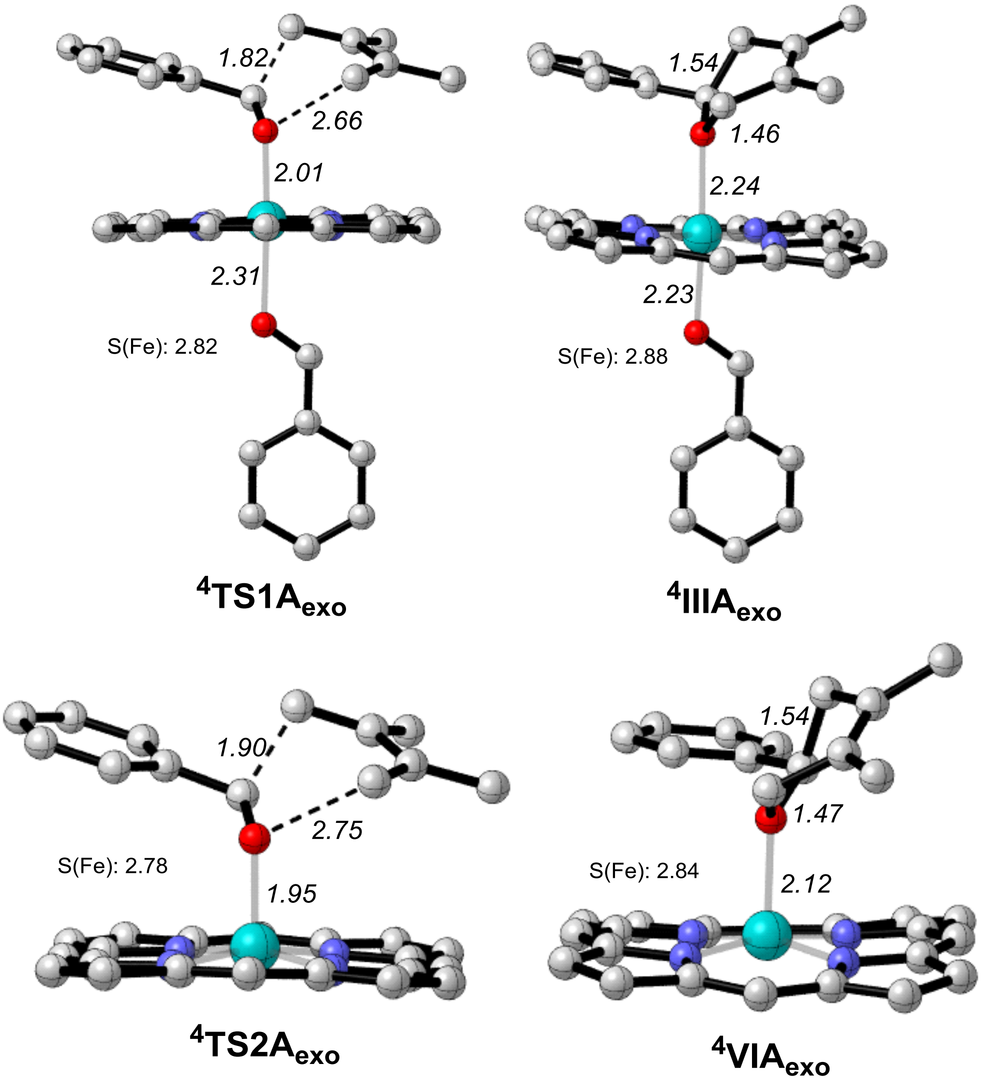

Supplementary Figure 3A. **Key *exo*-type structures optimized by the B3LYP-D3 method for the formation of A in the quartet state.** The key bond lengths (Å, in italics) and spin density on Fe are given. Unimportant hydrogen atoms are not shown for clarity.

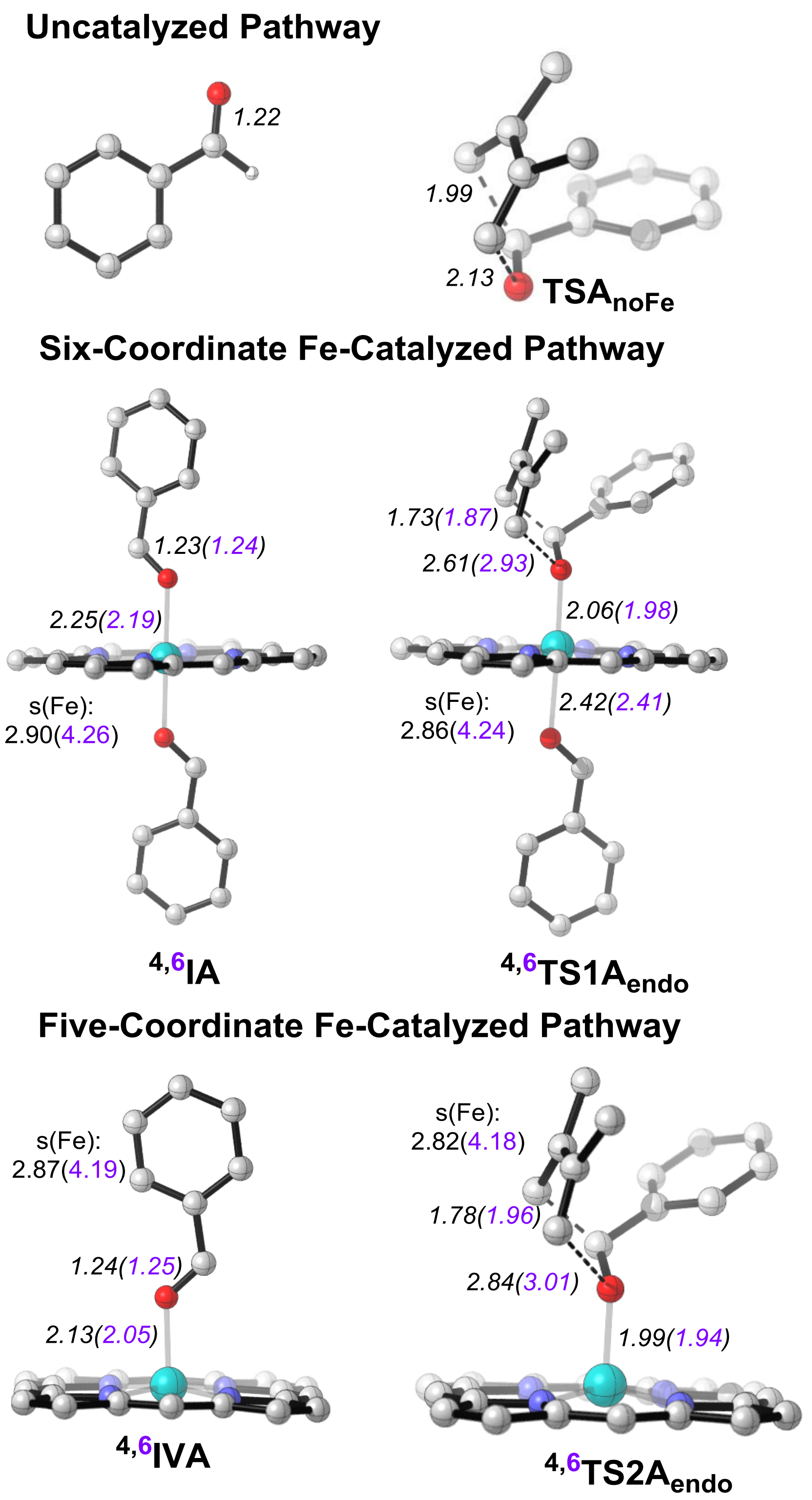

Supplementary Figure 3B. **Key optimized *endo*-type structures for the formation of A.** The formation of **A** in the singlet state for the uncatalyzed pathway (the first row) as well as in the quartet and sextet states (in purple) for the Fe-catalyzed pathways by the SMD B3LYP (no dispersion) method, key distances (Å, in italics) and spin density (s) on Fe are given. Unimportant hydrogen atoms are not shown for clarity.

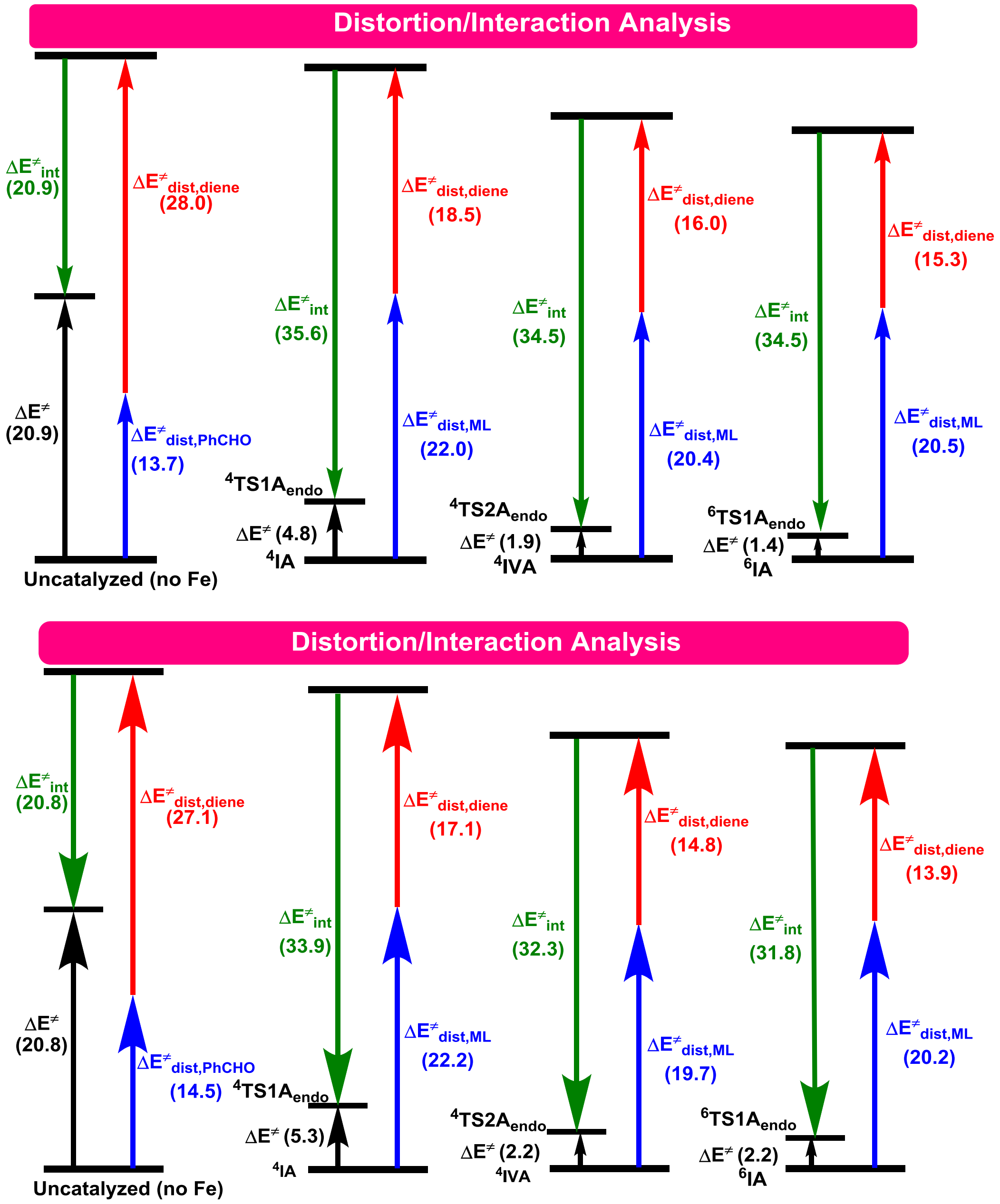

Supplementary Figure 4. Distortion/interaction analysis (in kcal mol^{-1}) results to form A. The top results were obtained by the SMD B3LYP-D3//B3LYP-D3 method and the bottom results by SMD B3LYP-D3 method. Distortion energy ($E^{\ddagger}_{dist,ML}$) includes distortion energy for the metal, ligand and PhCHO parts. $E^{\ddagger}_{dist,diene}$ represents the distortion energy for the diene part. $E^{\ddagger}_{int}$ is the interaction contribution.

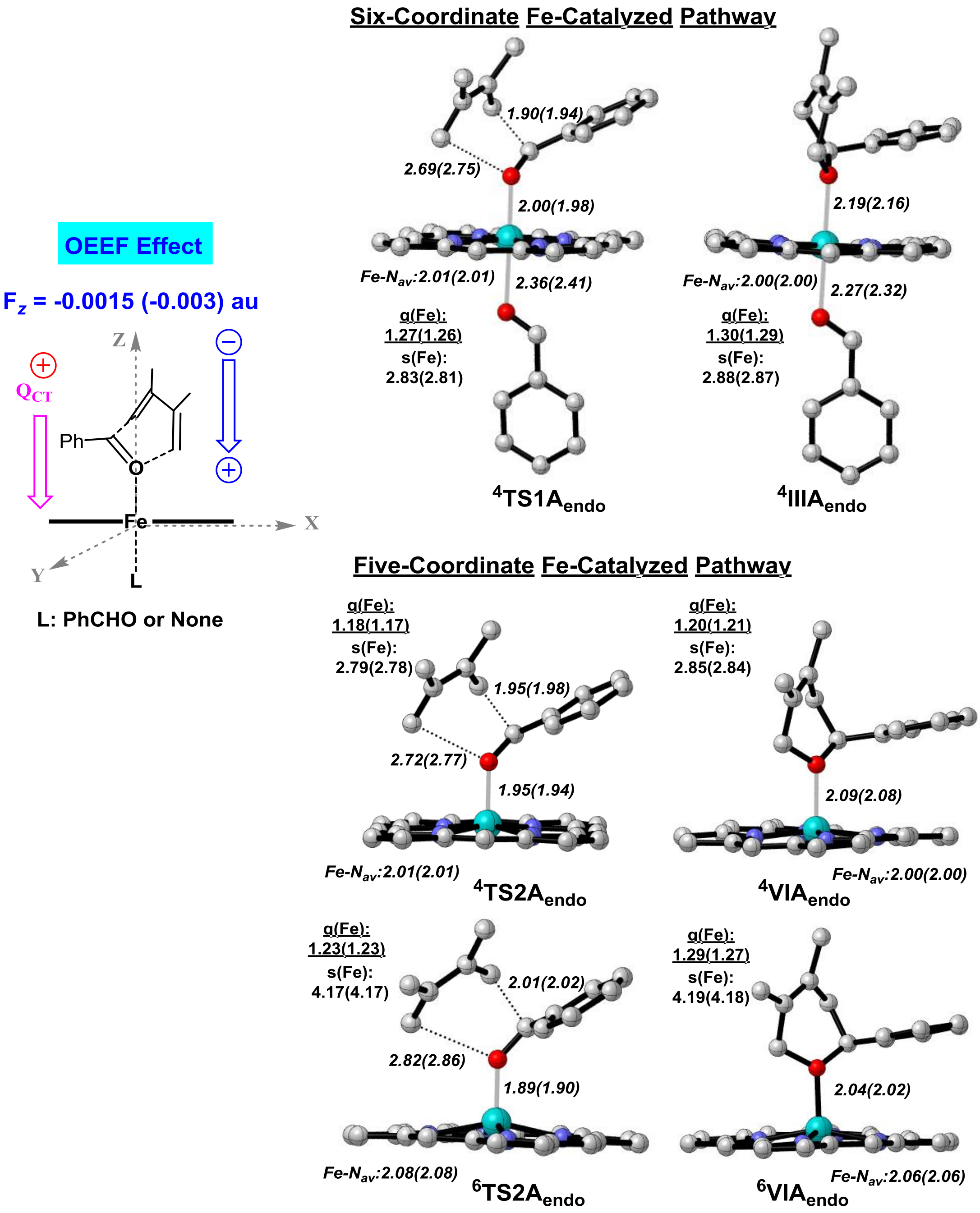

Supplementary Figure 5. Reaction profile in the presence of an OEEF to form A. The strength of the OEEF is -0.0015 au and -0.003 au (in parenthesis) were applied by the SMD B3LYP-D3 method. The key distances (in italic), Mulliken charge (in underline) and spin density (s) on Fe are given. Unimportant hydrogen atoms are not shown for clarity.

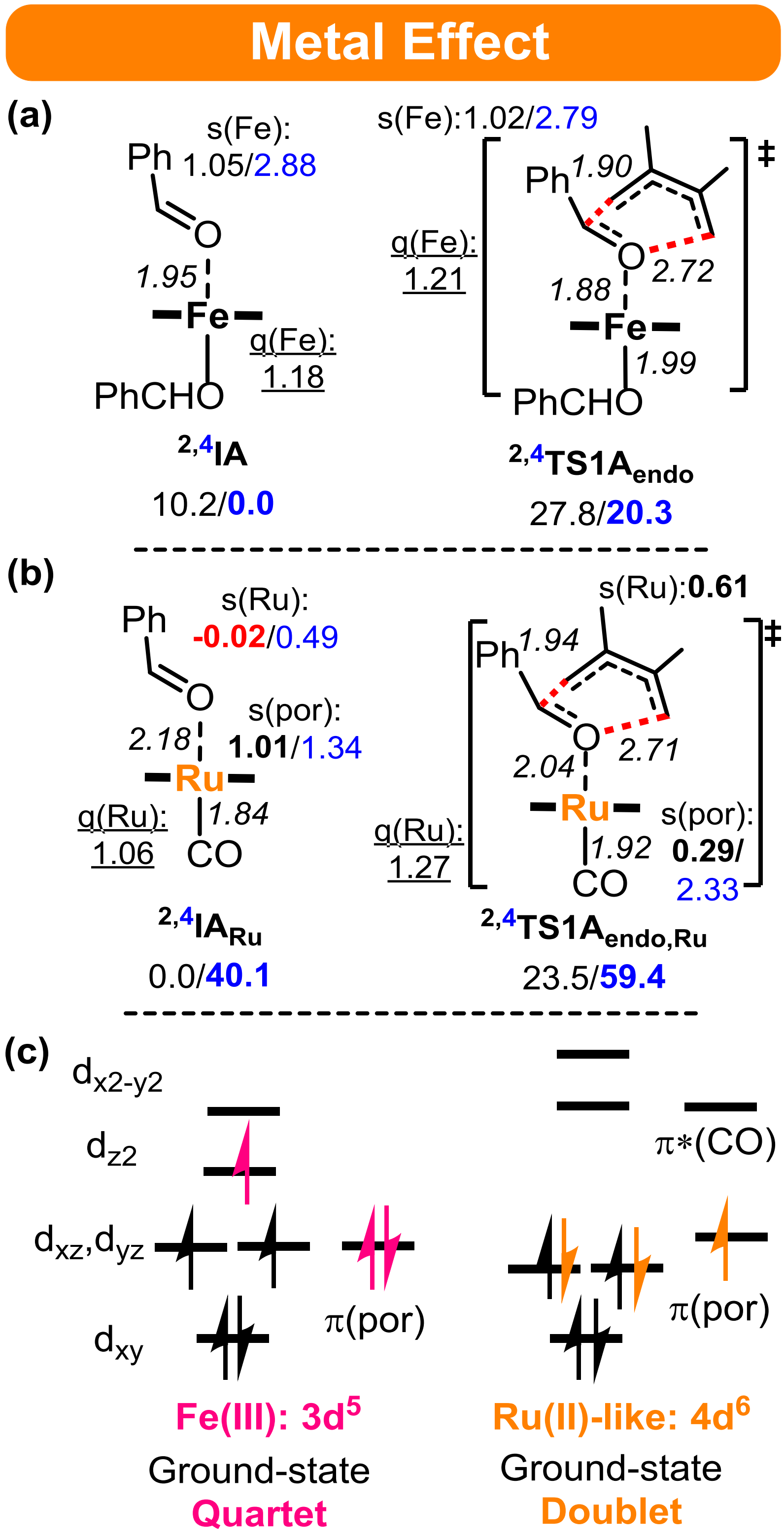

Supplementary Figure 6. Comparison between Fe(III)- and Ru(III)-catalyzed reactions to form A. Computed key energies (in kcal mol^{-1}) in solution for the (a) Fe(III)- and (b) Ru(III)-catalyzed reactions in the doublet and quartet states by the SMD B3LYP-D3//B3LYP-D3 method, the key distances (in Å), Mulliken charges and spin densities (s) on the metals are given. (c) Schematic electronic structures of the Fe(III) and Ru(III) catalysts. Notably, the standard state correction was applied here.

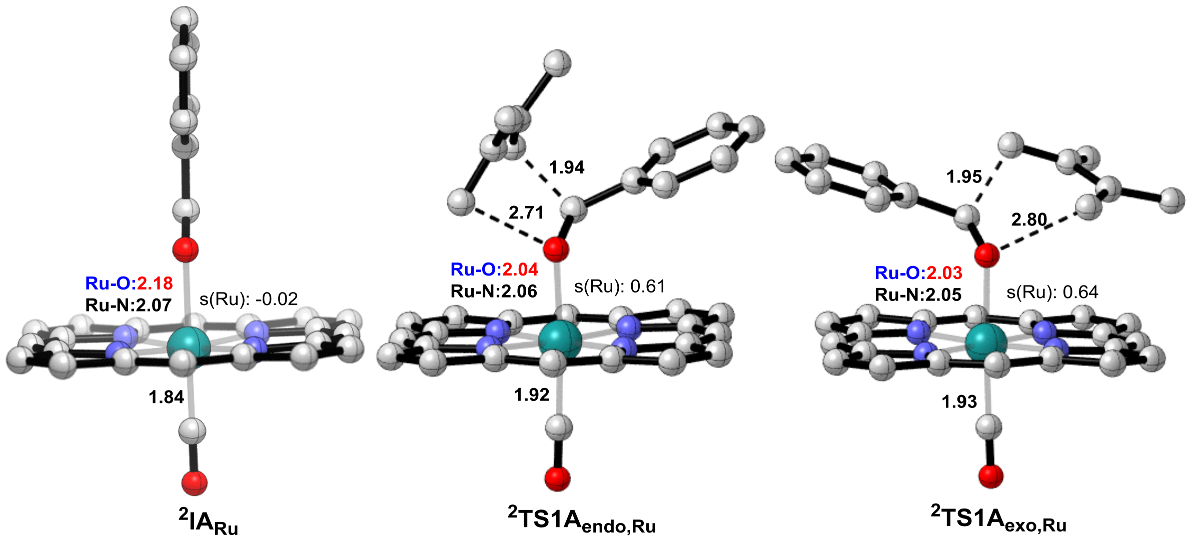

Supplementary Figure 7. Structures involved in the formation of A catalyzed by Ru(III)-Porphyrin. These structures were optimized in the doublet state by the B3LYP-D3 method. The key bond lengths (in angstrom) and the spin density (s) on the metal are given. Unimportant hydrogen atoms are not shown for clarity.

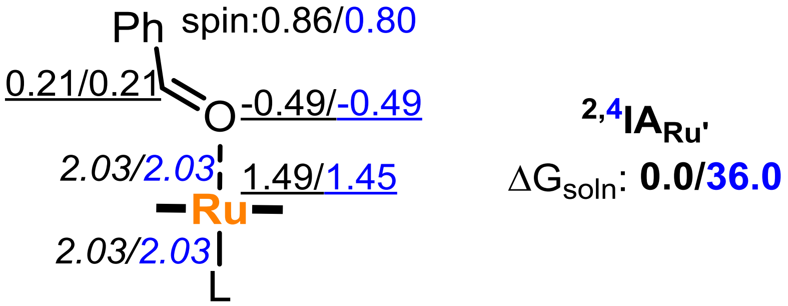

Supplementary Figure 8. The properties of the Ru catalyst. The relative free energies (in kcal mol^{-1}) for the Ru catalyst with the replacement of the CO ligand with PhCHO (L) by the SMD B3LYP-D3 method, and the key distance (in italic) and Mulliken charge (in underline) and spin density on Ru by the SMD B3LYP-D3//B3LYP-D3 are given.

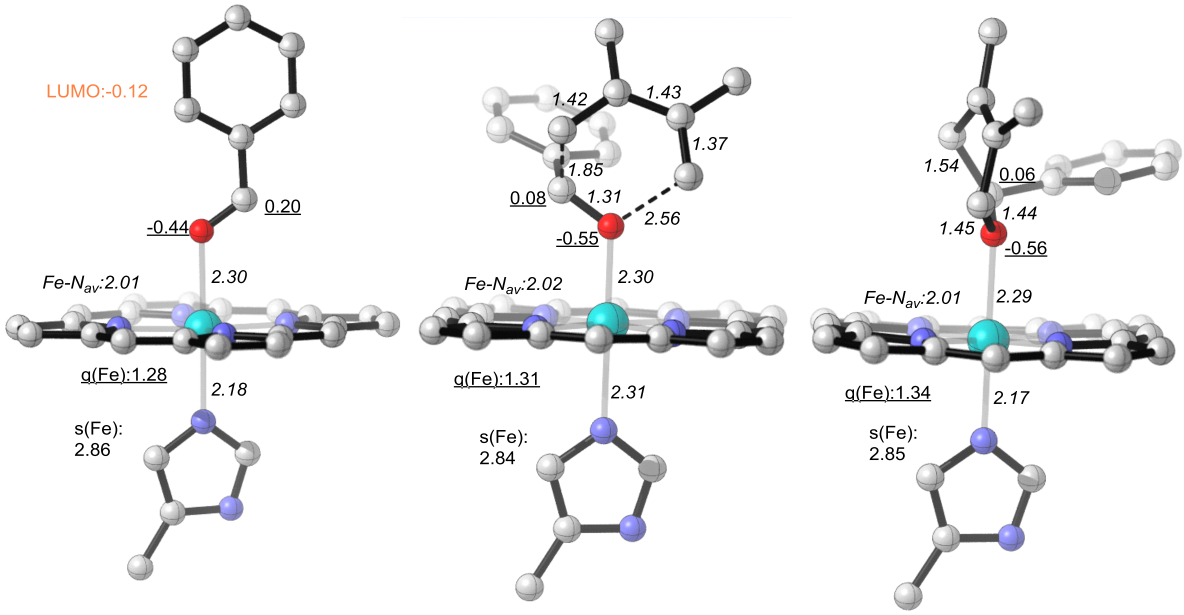

Supplementary Figure 9. Formation of A catalyzed by the imidazole-coordinated Fe complex. These structures were optimized in the quartet state in diethylether solution by the SMD B3LYP-D3 method. The key distances (in italics), Mulliken charges (underlined), LUMO energy (eV, in orange) and spin density on Fe by the SMD B3LYP-D3 method are given. Unimportant hydrogen atoms are not shown for clarity.

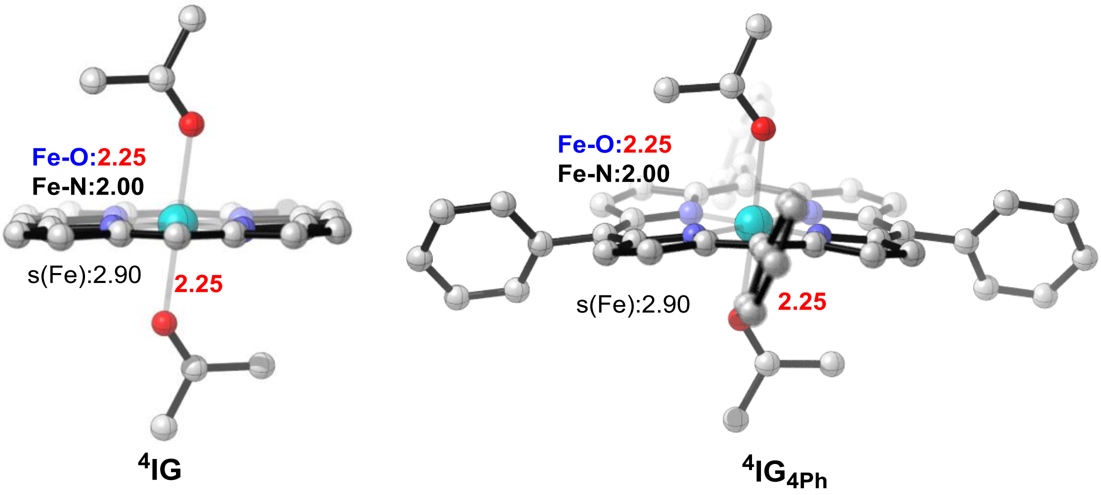

Supplementary Figure 10. Fe(III)-complexes coordinated with acetone. The key distances (in italics), spin density on Fe in the quartet state at the SMD (acetone solution) B3LYP-D3 method are given. Unimportant hydrogen atoms are not shown for clarity.

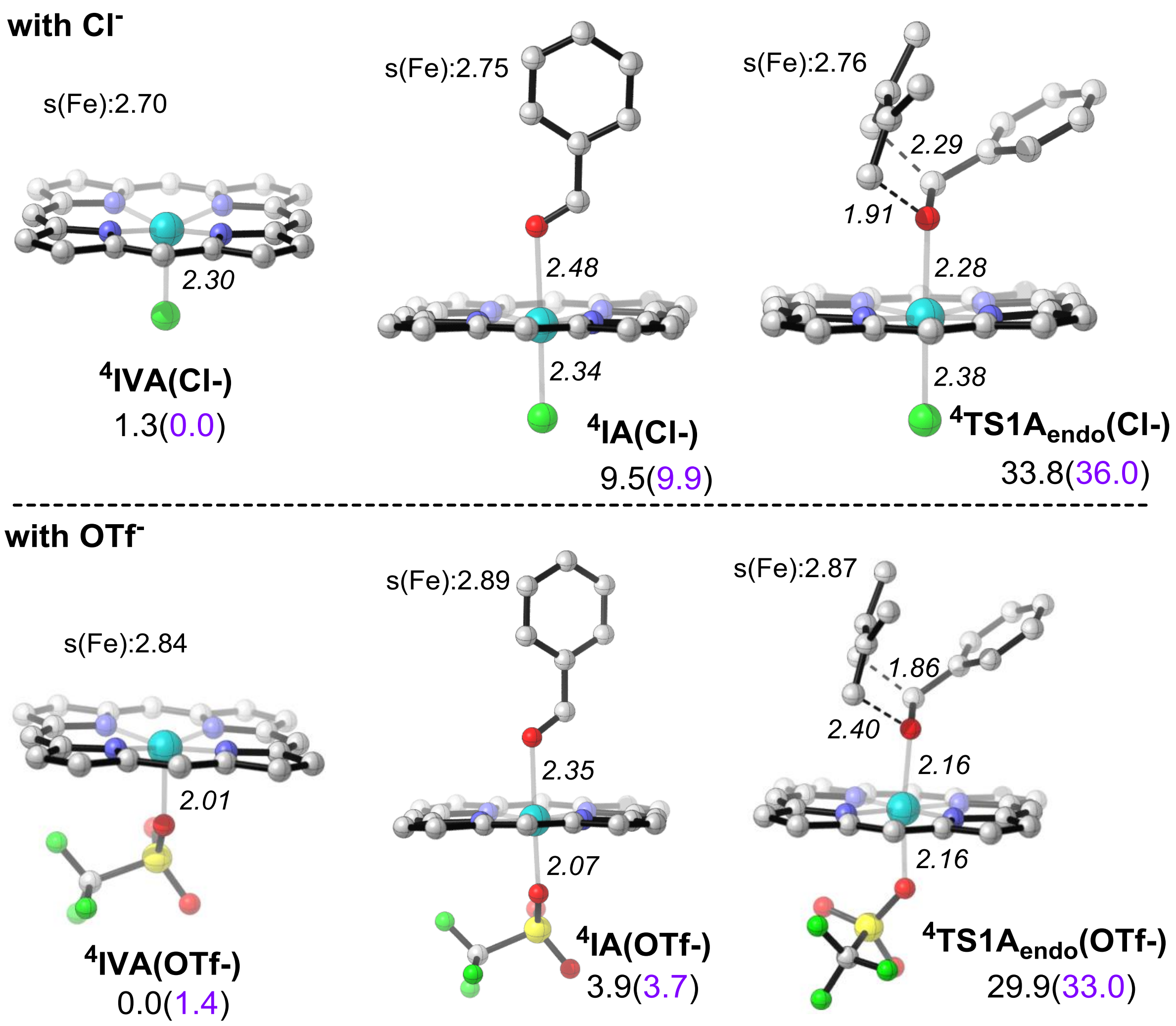

Supplementary Figure 11. The charge effect of the Fe complexes. Key optimized structures for the formation of **A** for the two neutral Fe(III) complexes in the quartet state in solution by the SMD B3LYP-D3 method. The key distances (Å, in italics), spin density on Fe and relative energies (in kcal mol^{-1}) for the quartet state and sextet state (in the parentheses in purple) at 353.15 K calculated by the SMD B3LYP-D3 method are given. Unimportant hydrogen atoms are not shown for clarity. Notably, the standard state correction was applied here. See Supplementary Table 48 for the complete energies for the three different spin states.

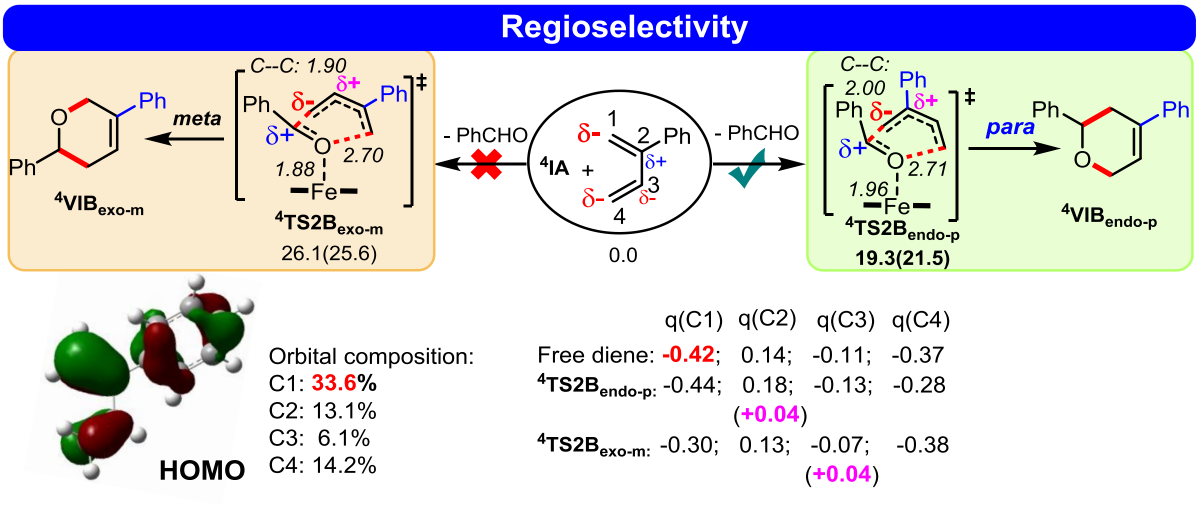

Supplementary Figure 12. Regioselectivity in the Fe-catalyzed ODA reaction. Free-energy profile (in kcal mol^{-1}) for the lowest-energy pathways to give the *para*- and *meta*-substituted products were computed by the SMD B3LYP-D3//B3LYP-D3 and SMD B3LYP-D3 (in parentheses) methods. The key distances (in Å, in italics), key Mulliken charges (q) and free diene's HOMO by the SMD B3LYP-D3//B3LYP-D3 method are given. Notably, the standard state correction was not applied here.

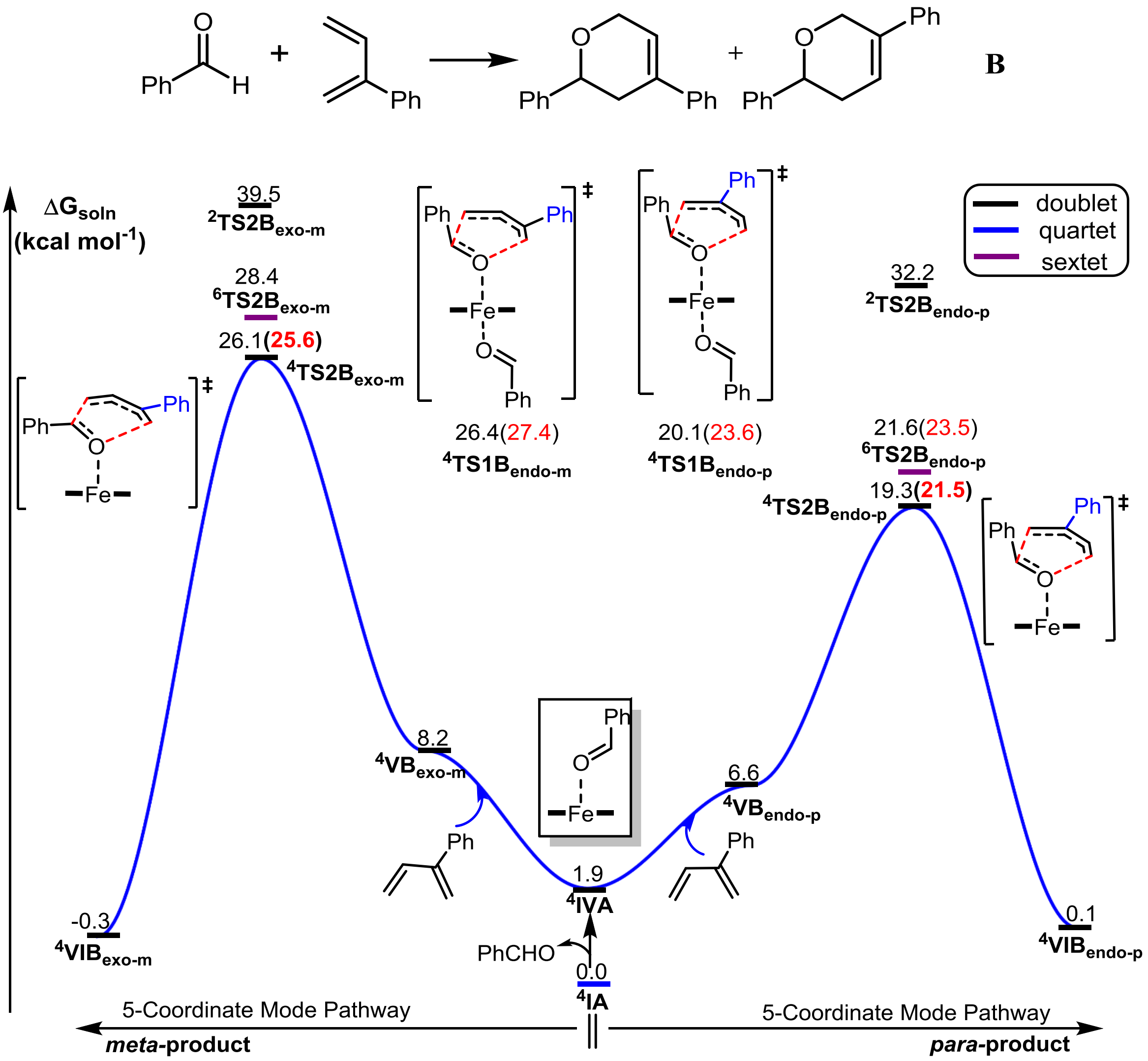

Supplementary Figure 13. Free energy profile to form product B. The most favorable pathways in three spin states in solution at 353.15 K by the SMD B3LYP-D3//B3LYP-D3 and SMD B3LYP-D3 (in parenthesis) methods are given. Notably, the standard state correction was not applied here.

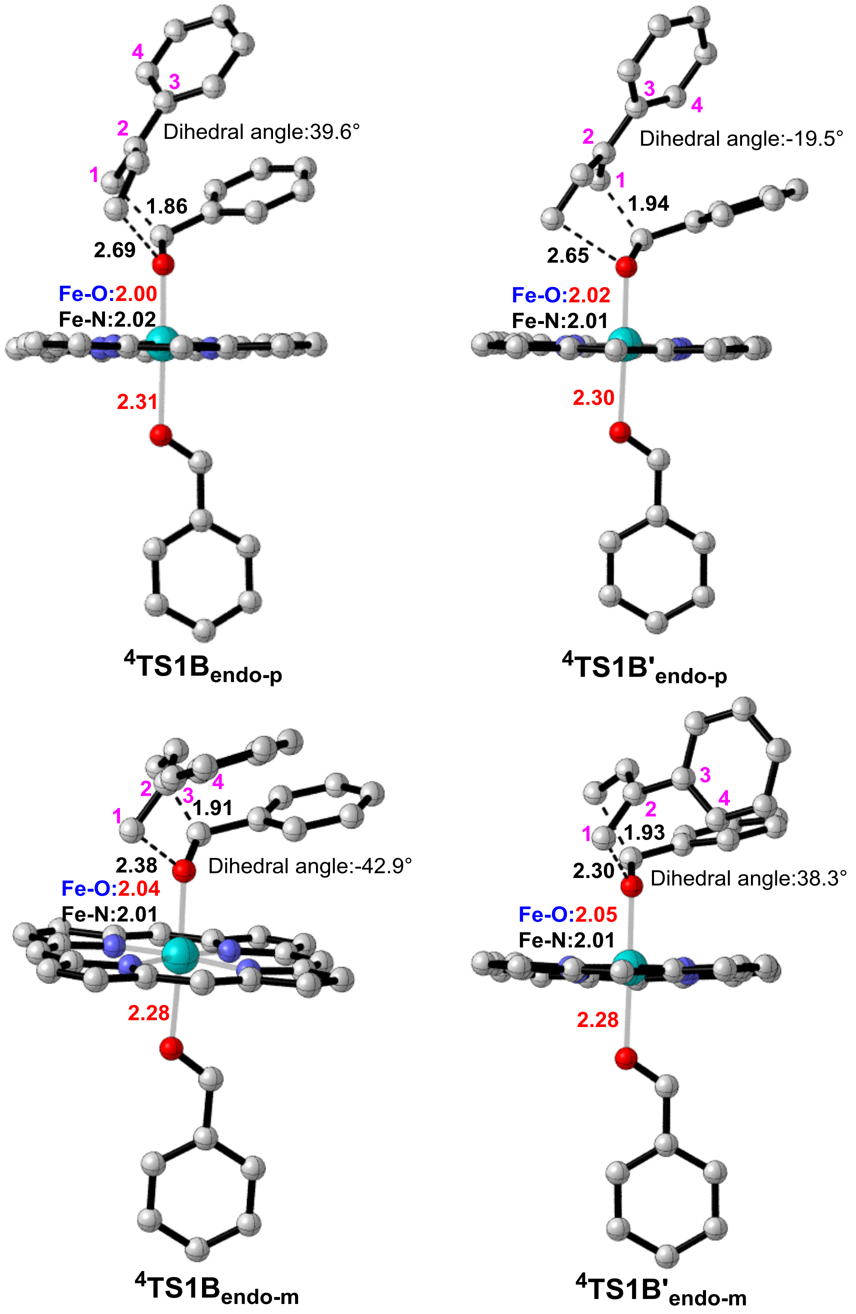

Supplementary Figure 14. Key optimized structures for the formation of B in the quartet state. The structures were optimized by the B3LYP-D3 method. The key bond lengths (in angstrom) are given. The main difference between **B** and **B'** is the dihedral angle of C1-C2-C3-C4. Unimportant hydrogen atoms are not shown for clarity.

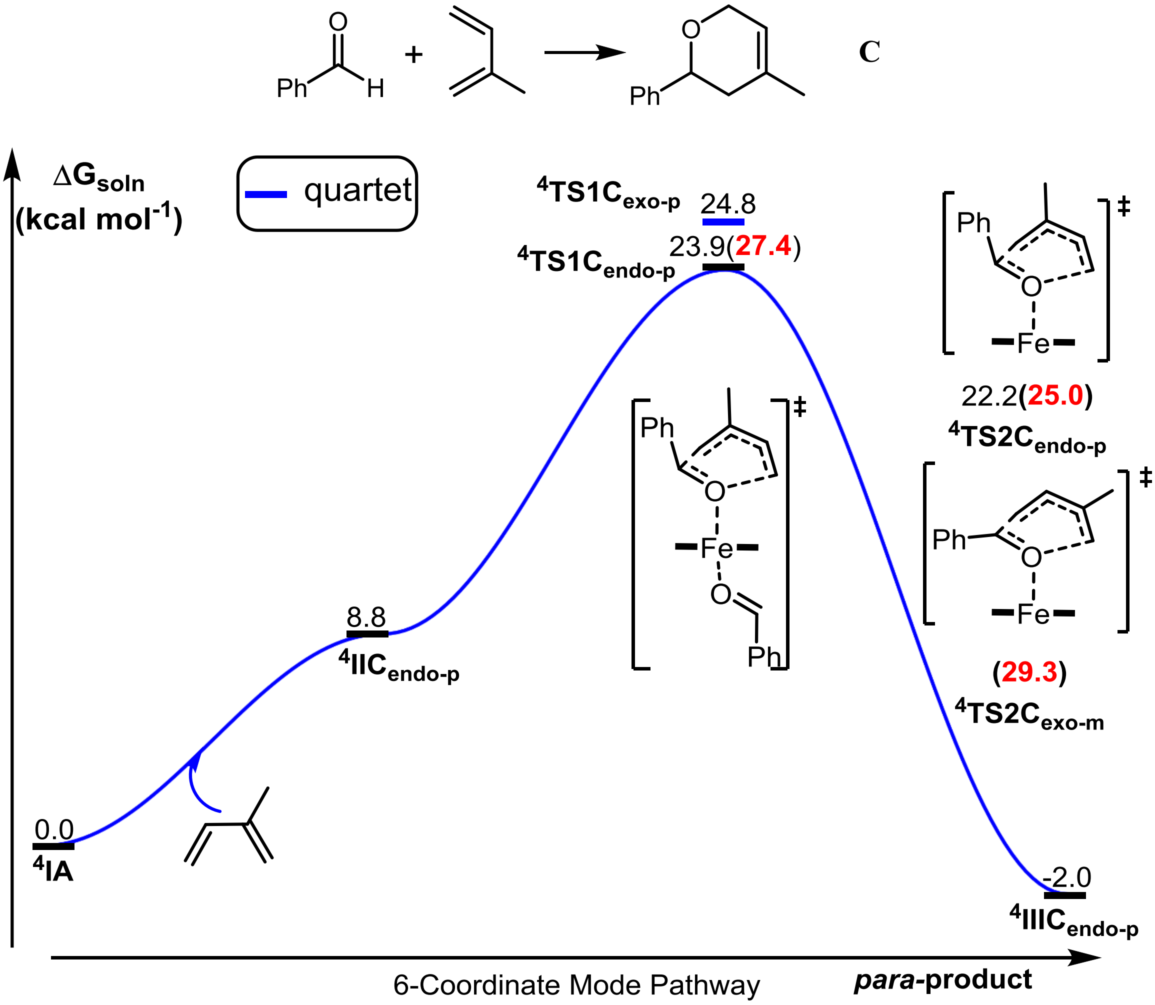

Supplementary Figure 15. **Free energy profile to form product C.** The favorable pathway in the quartet state in solution at 353.15 K by the SMD B3LYP-D3//B3LYP-D3 and SMD B3LYP-D3 (in parenthesis) methods are given. Notably, the standard state correction was not applied here.

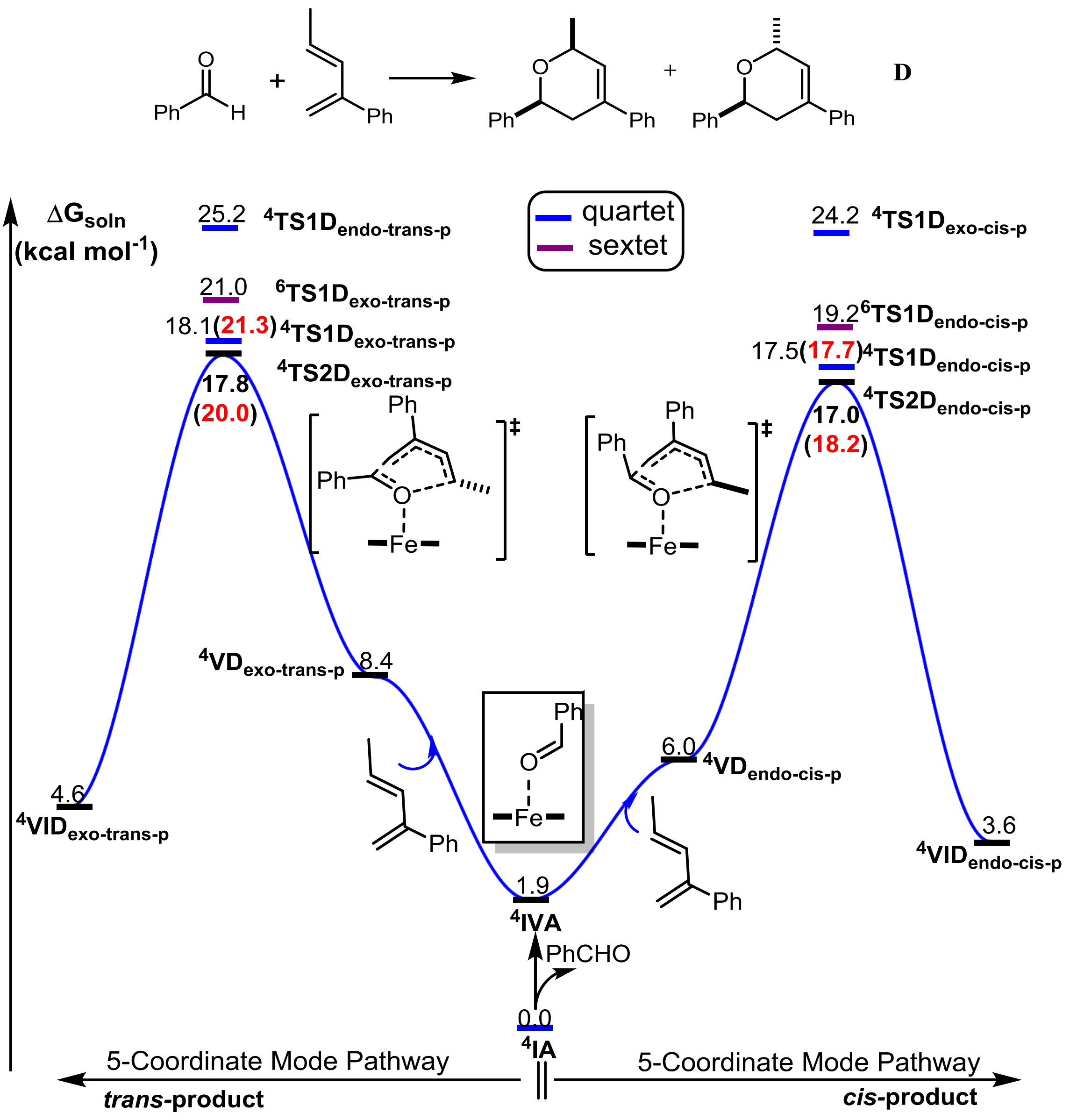

Supplementary Figure 16. **Free energy profile to form product D.** The key pathways in the quartet and sextet states in solution at 353.15 K by the SMD B3LYP-D3//B3LYP-D3 and SMD B3LYP-D3 (in parenthesis) methods are given. Notably, the standard state correction was not applied here.

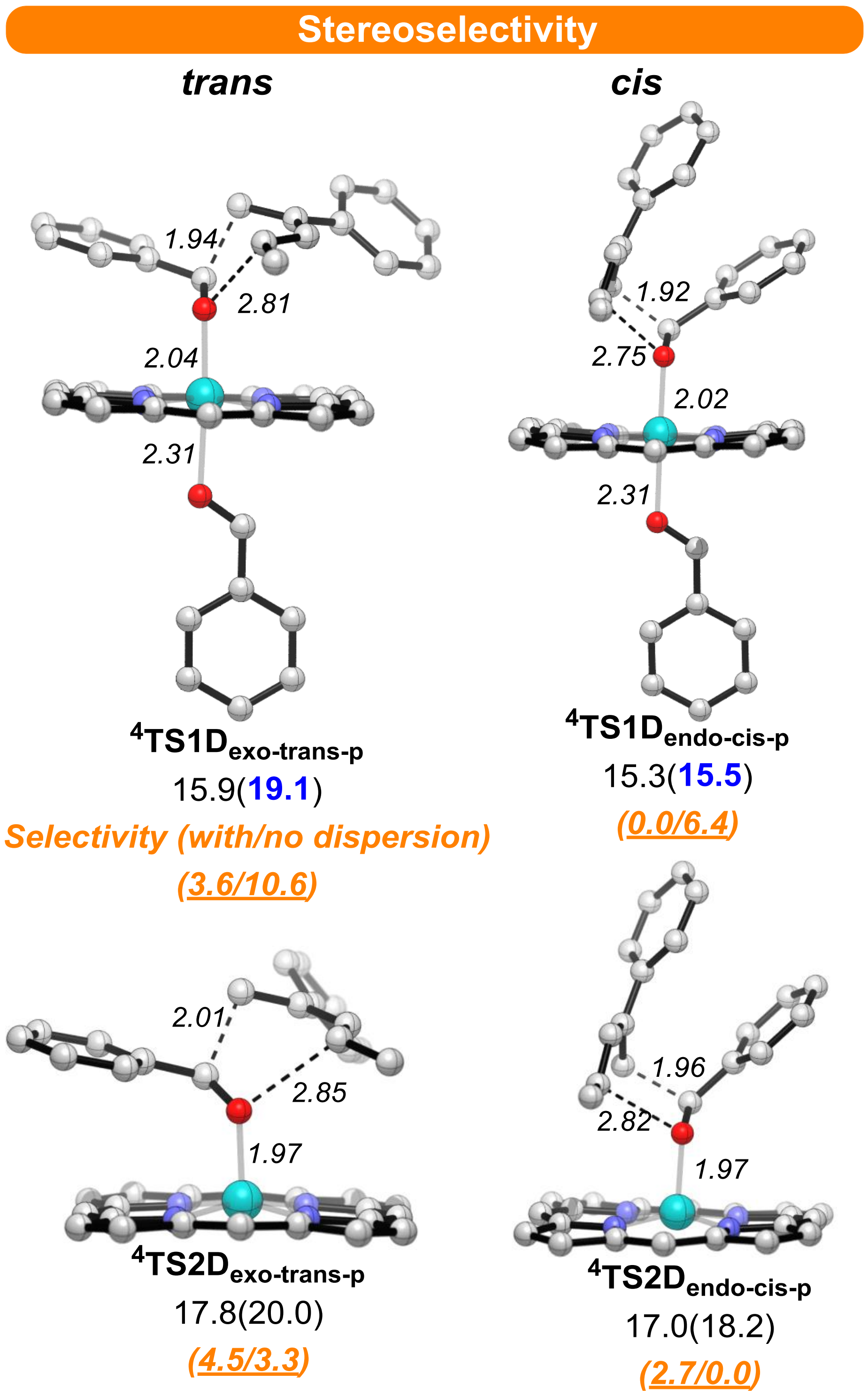

Supplementary Figure 17. Stereoselecity in the formation of D. The key transition states to form *cis*- and *trans*-**D** in the quartet state with the relative free energies (in kcal mol^{-1}) by the SMD B3LYP-D3//B3LYP-D3 and SMD B3LYP-D3 (in parentheses) methods and the key distances (in italics) by the SMD B3LYP-D3//B3LYP-D3 method are given. The dispersion contribution to the *cis*-selectivity in solution (in both parentheses, shown in orange and underlined) were estimated using the SMD B3LYP-D3-optimized structures. Unimportant hydrogen atoms are not shown for clarity. Notably, the standard state correction was applied here.

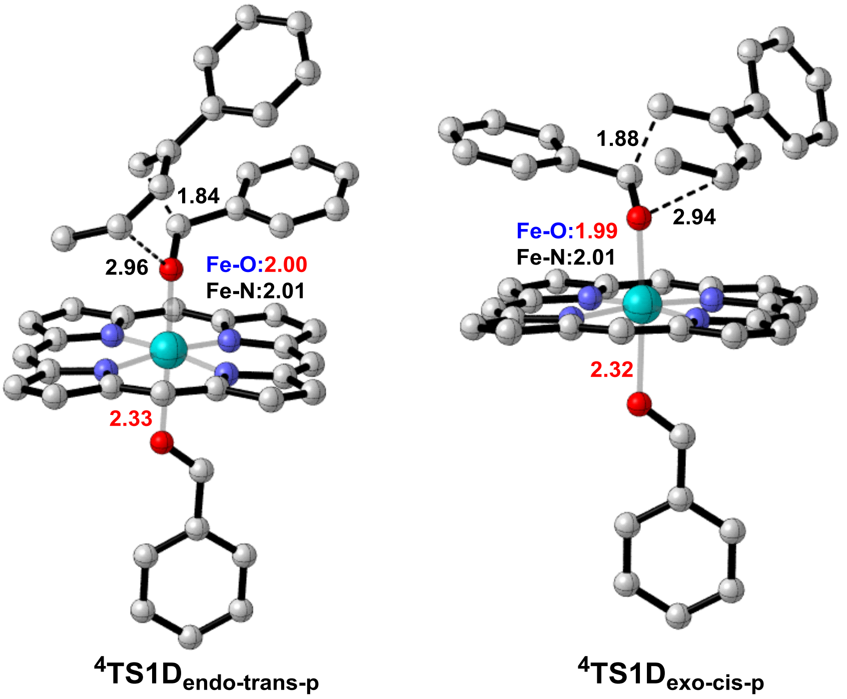

Supplementary Figure 18. **Other optimized transition state structures to form D in the quartet state by the B3LYP-D3 method.** The key bond lengths (in angstrom) are given. Unimportant hydrogen atoms are not shown for clarity.

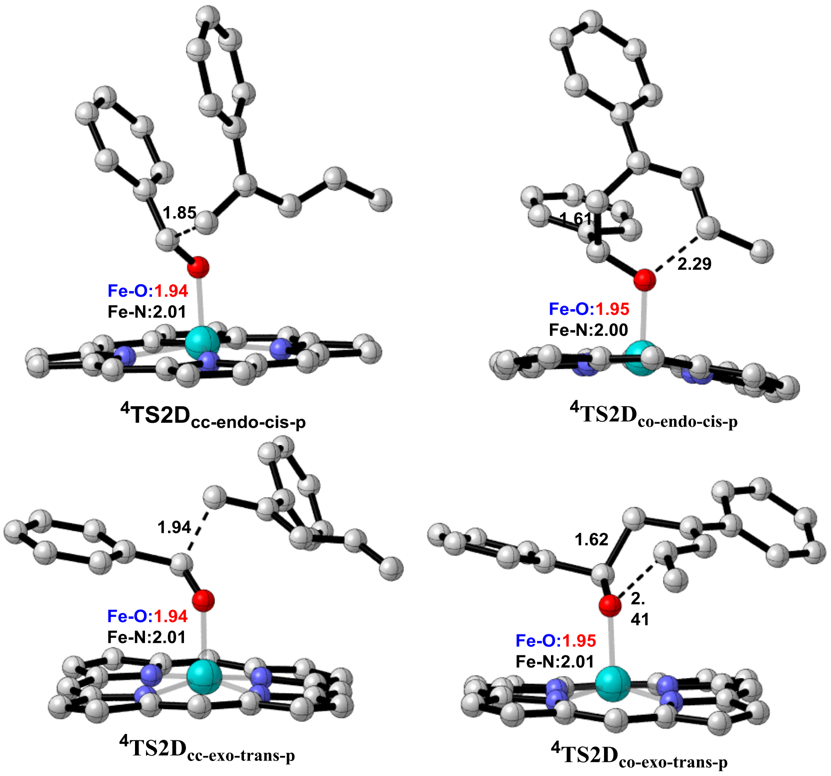

Supplementary Figure 19. **Key optimized stepwise transition state structures to form D in the quartet state.** The structures were optimized by the B3LYP-D3 method. The key bond lengths (in angstrom) are given. Unimportant hydrogen atoms are not shown for clarity.

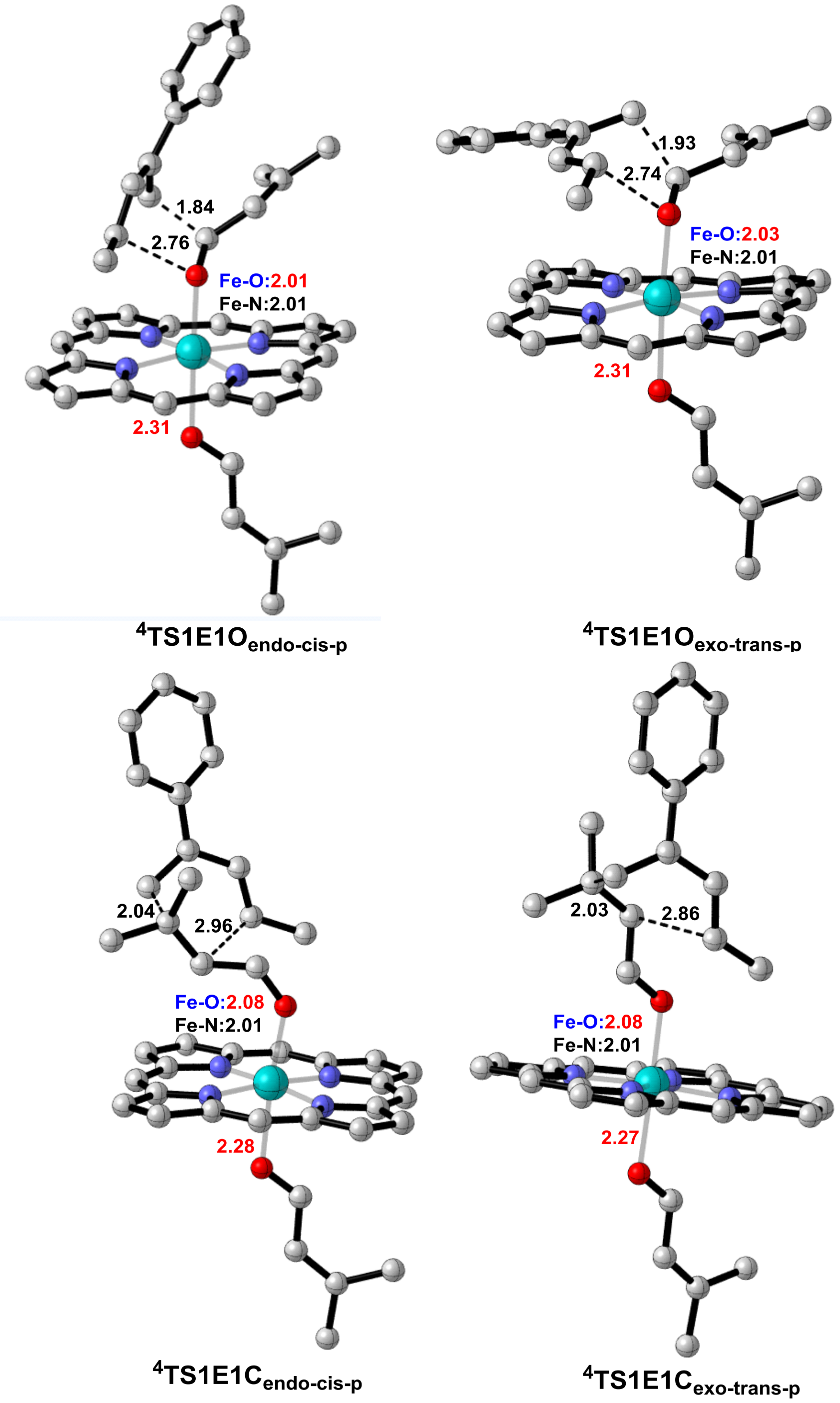

Supplementary Figure 20. Key optimized transition state structures to form E1O and E1C in the quartet state. The structures were optimized by the B3LYP-D3 method. The key bond lengths (in angstrom) are given. Unimportant hydrogen atoms are not shown for clarity.

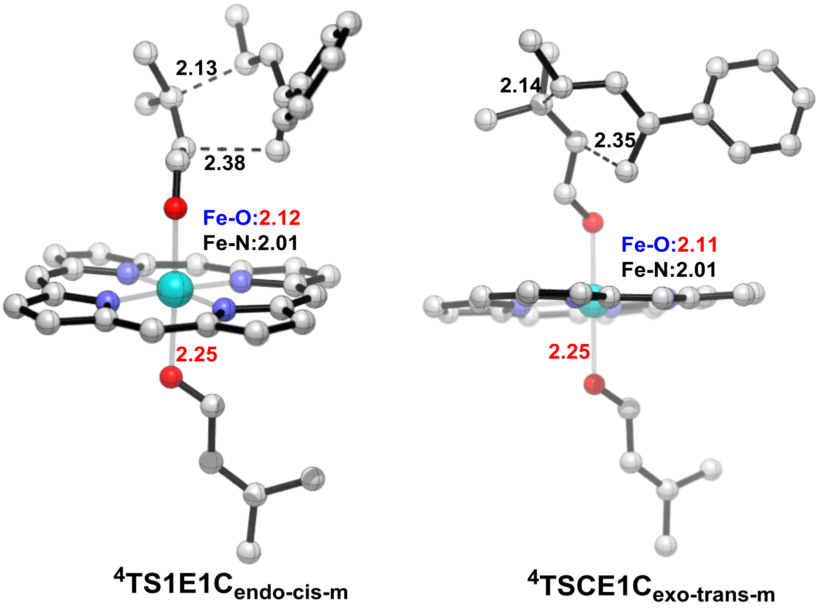

Supplementary Figure 21. Key optimized transition state structures for the less favorable *meta*-type pathway to form E1C. The reaction involves the formation of two C-C bond in the quartet state. The structures were optimized by the B3LYP-D3 method. The key bond lengths (in angstrom) are given. Unimportant hydrogen atoms are not shown for clarity.

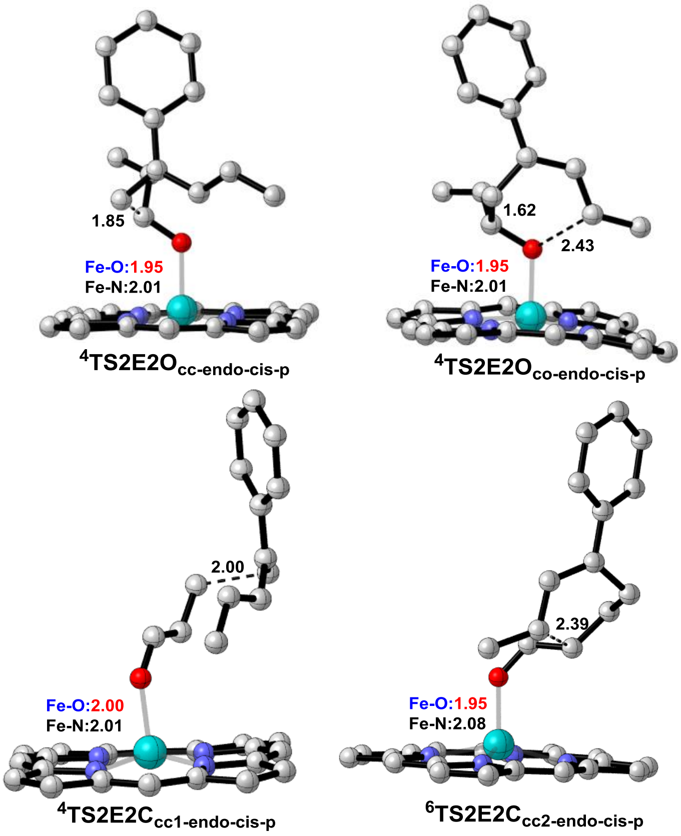

Supplementary Figure 22. Key optimized transition state structures in the stepwise mechanism to form E2O and E2C. The structures were optimized by the B3LYP-D3 method. The key bond lengths (in angstrom) are given. Unimportant hydrogen atoms are not shown for clarity.

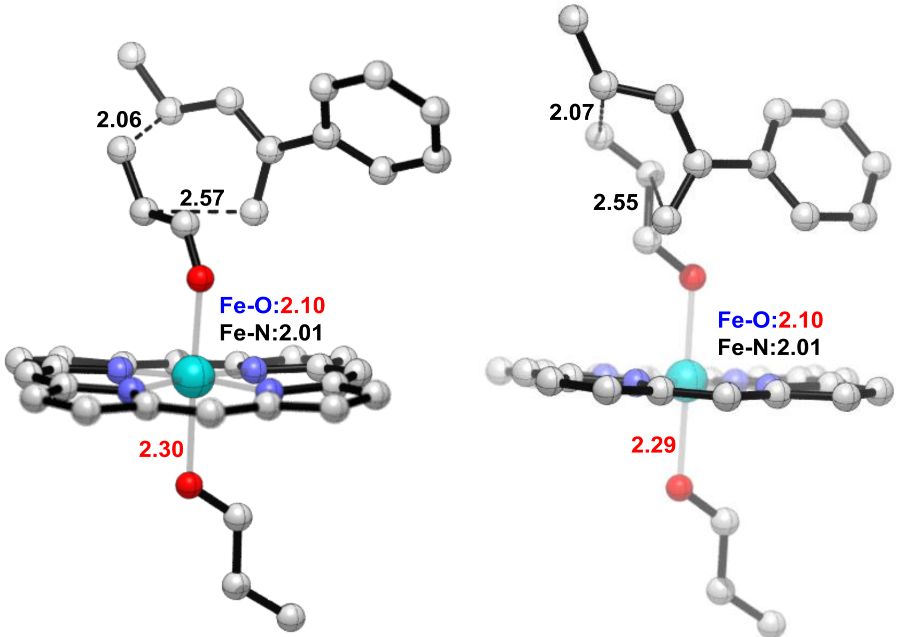

^{4}TS1E2C$_{endo\text{-}cis\text{-}m}$ ^{4}TS1E2C$_{exo\text{-}trans\text{-}m}$

Supplementary Figure 23. Key optimized *meta*-type transition states to form E2C in the quartet state. The structures were optimized by the B3LYP-D3 method. The key bond lengths (in angstrom) are given. Unimportant hydrogen atoms are not shown for clarity.

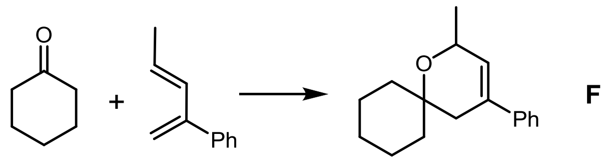

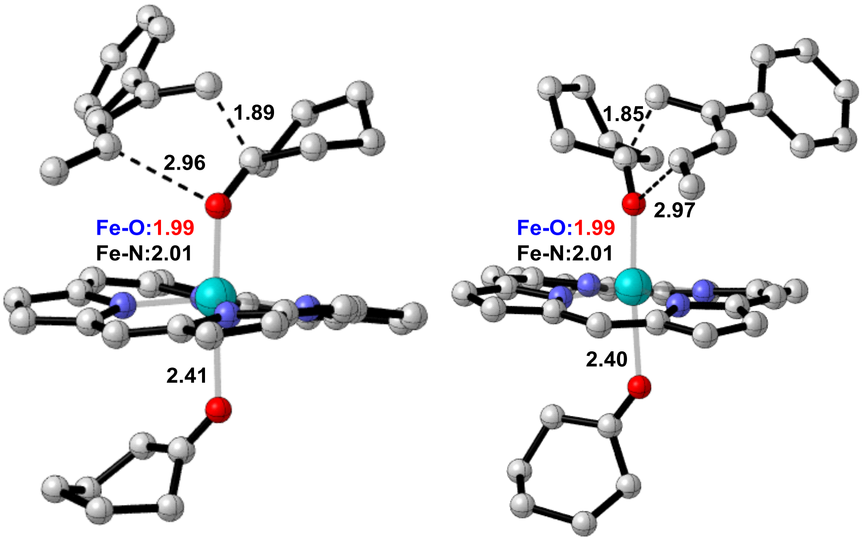

^{4}TS1F$_1$ ^{4}TS1F$_2$

Supplementary Figure 24. Key optimized transition states to form F in the quartet state. The structures were optimized by the B3LYP-D3 method. The key bond lengths (in angstrom) are given. Unimportant hydrogen atoms are not shown for clarity.

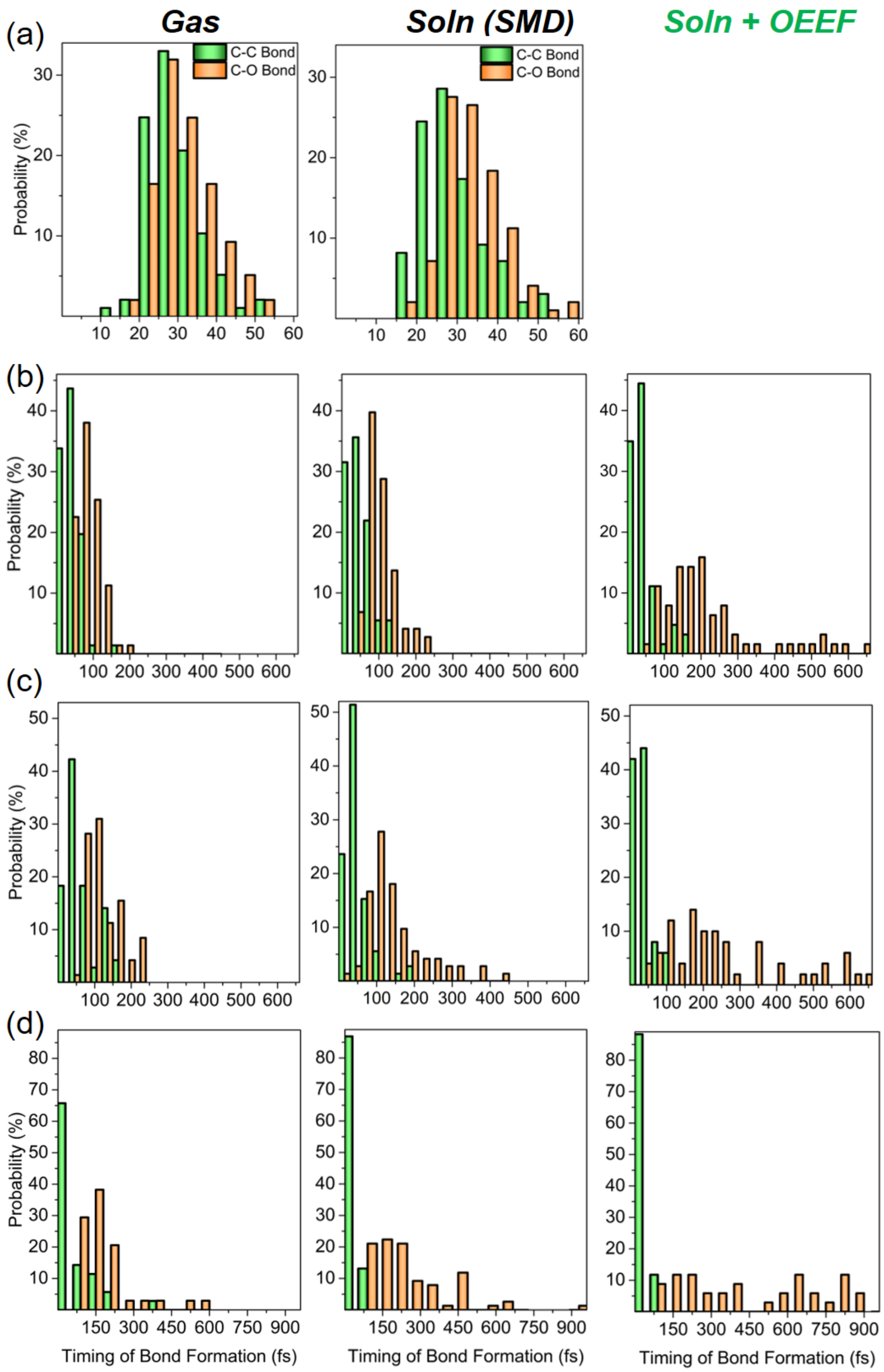

Supplementary Figure 25. Timing of the two new bond formations. Distribution of timing (in fs) of the C-C and C-O bond forms (<1.6 Å) for (a) the uncatalyzed reaction; Fe-catalyzed reaction for (b) the six-coordinate and quartet state, (c) the five-coordinate and quartet state and (d) the five-coordinate and sextet state in gas phase (left), SMD solvent (middle) and SMD solvent in the presence of an OEEF with strength of -0.003 au along the reacting Fe-O bond (right). Note that for the SMD + OEEF MD simulations, one trajectory for the five-coordinate and quartet state, and 16 trajectories of the five-coordinate sextet state do not form the C-O bond within 900 fs. Thus, these trajectories were not considered in this Figure.

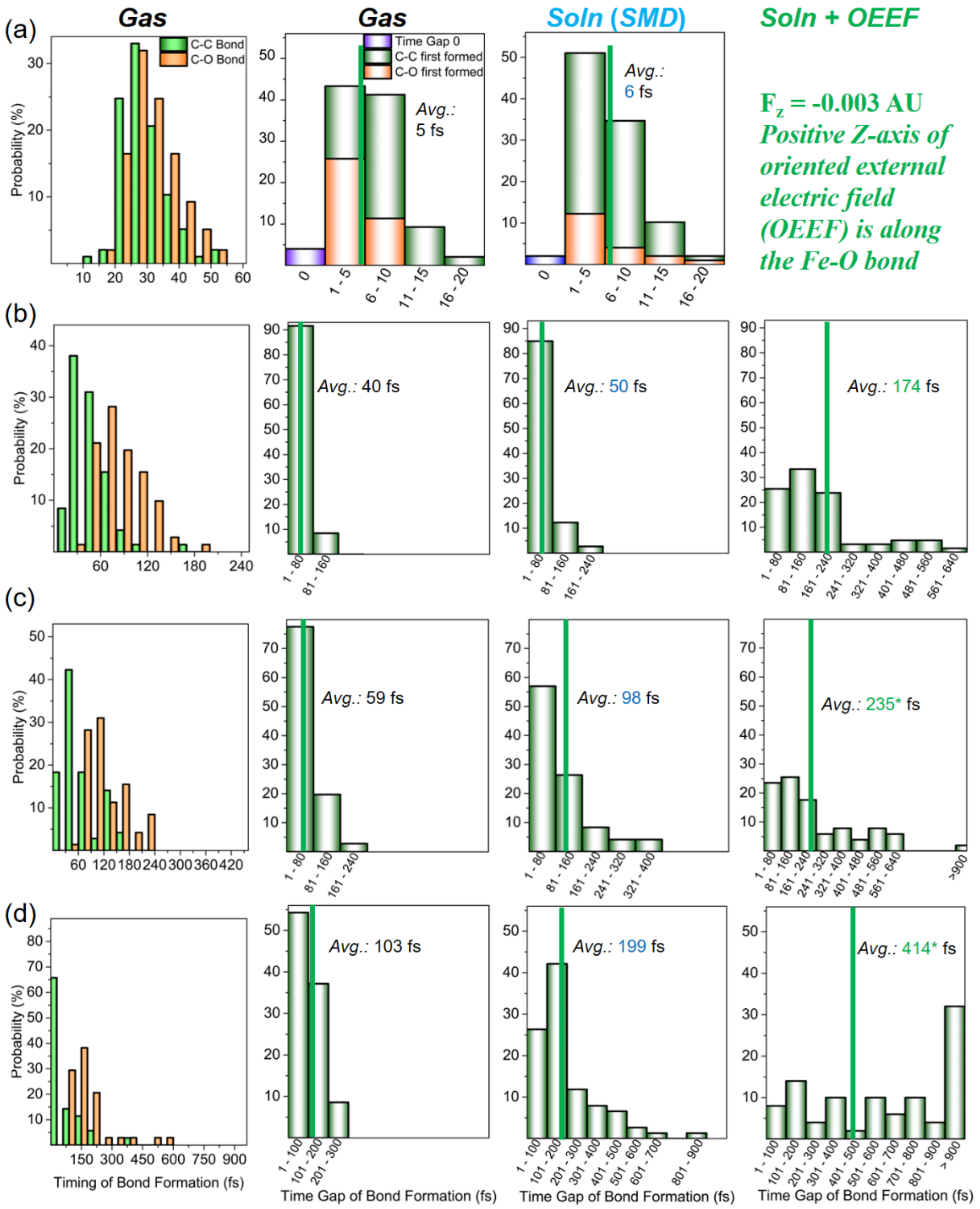

Supplementary Figure 26. Time gap between the forming C-C and C-O bonds. Distribution in the timing (in fs) of the formation of the C-C and C-O bonds (the first column) and the time gap (in fs) between C-C and C-O bond formation (the second, third and fourth columns) for (a) the uncatalyzed reaction and (b) the Fe-catalyzed reaction of the six-coordinate and quartet state, (c) the five-coordinate and quartet state and (d) the five-coordinate and sextet state in the gas phase and solution (without and with the OEEF; *some trajectories still cannot form the C-O bond (<1.6 Å) after 900 fs simulations).

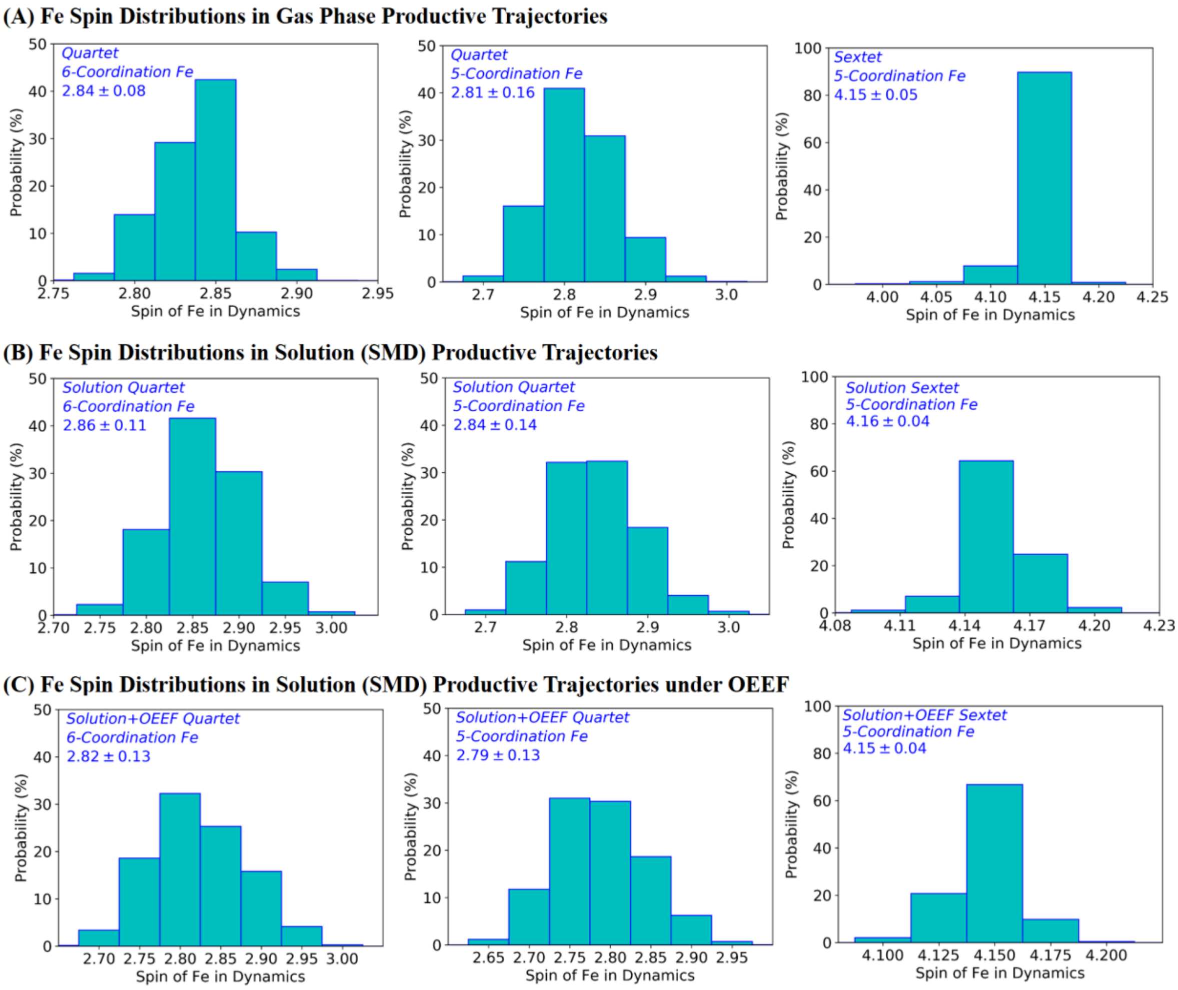

Supplementary Figure 27. Distribution of Fe spin density throughout the trajectories. Fe spin distribution in the six-coordinate quartet Fe complexes (left), five-coordinate quartet (middle) and sextet (left) Fe complexes in all the productive trajectories were ploted for the (A) gas phase simulations, solution phase (SMD) simulations (B) in absence of oriented external electric field and (C) in presence of oriented external electric field.

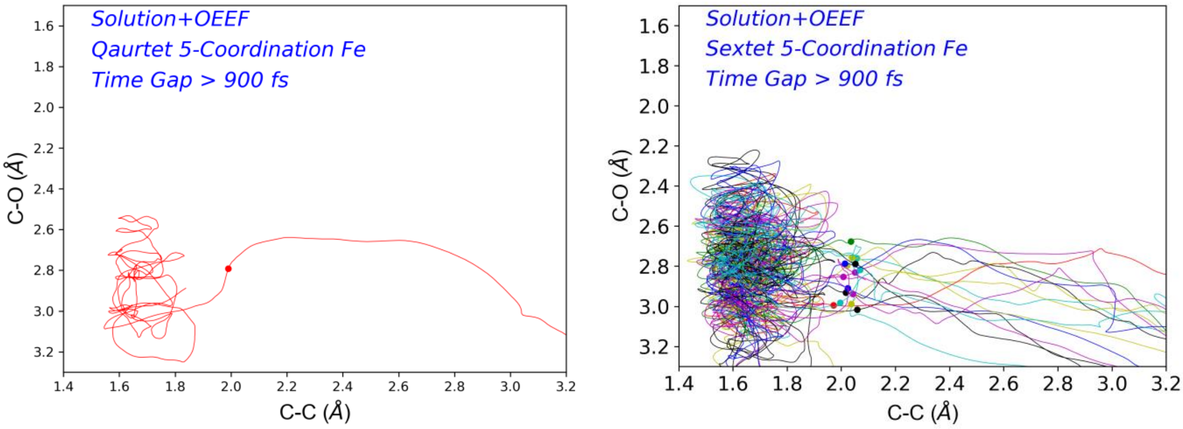

Supplementary Figure 28. Trajectories not forming the C-O bond after 900 fs. Trajectories for the quartet (left) and sextet (right) five-coordinate mode pathway in the presence of an oriented external electric field (OEEF) were ploted.

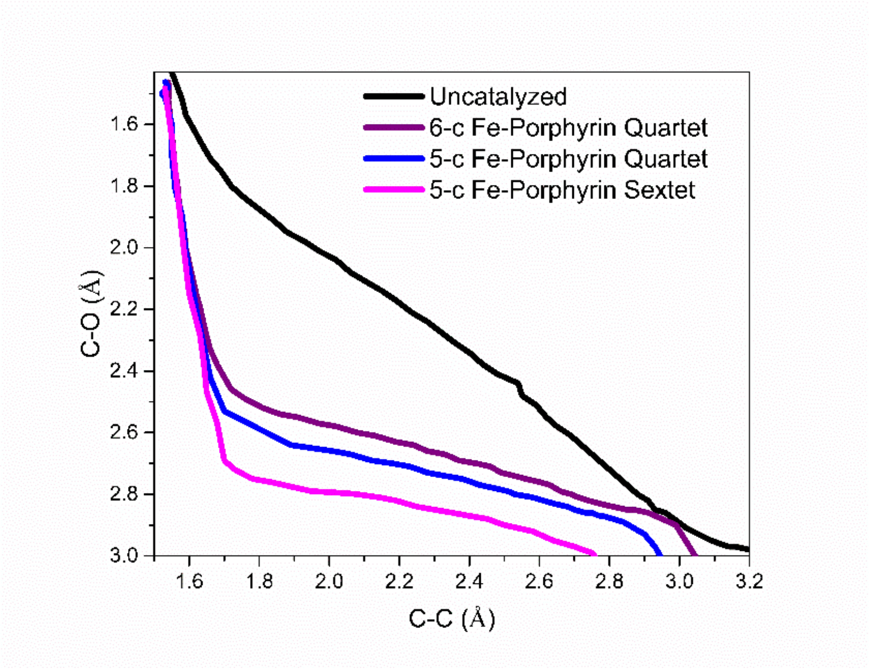

Supplementary Figure 29. Correlation of the key bond length in IRC calculations. The correlation between the forming C-O bond distance (Å) and the C-C bond distance obtained from the minimum energy path for the uncatalyzed (black line), six-coordinate (6-c) ^{4}**Fe**, five-coordinate (5-c) ^{4}**Fe** and ^{6}**Fe** catalyzed reactions in gas phase were ploted.

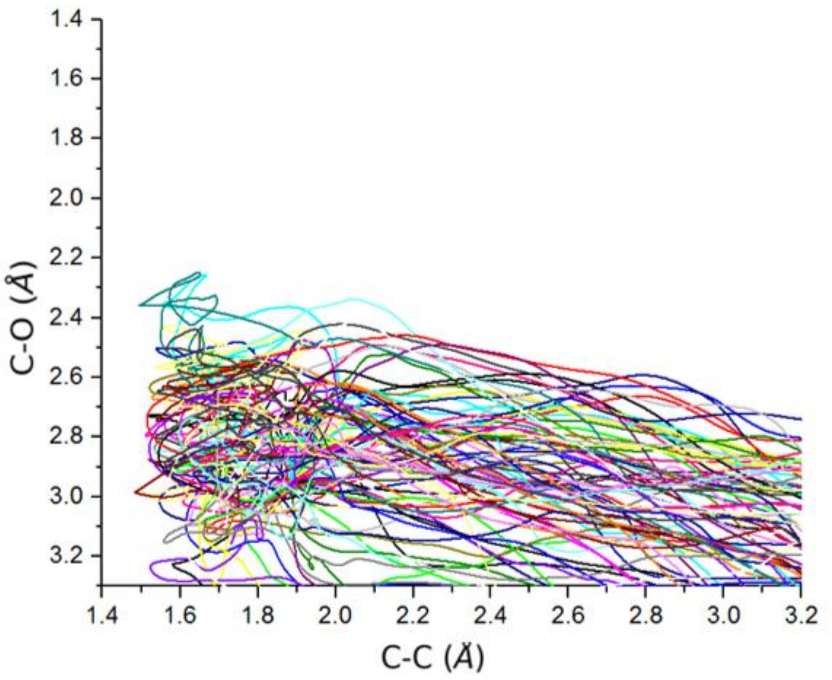

Supplementary Figure 30. Recrossing trajectories. Overlay of the re-crossing trajectories for the five-coordinate ^{6}**Fe** catalyzed reaction in gas phase.

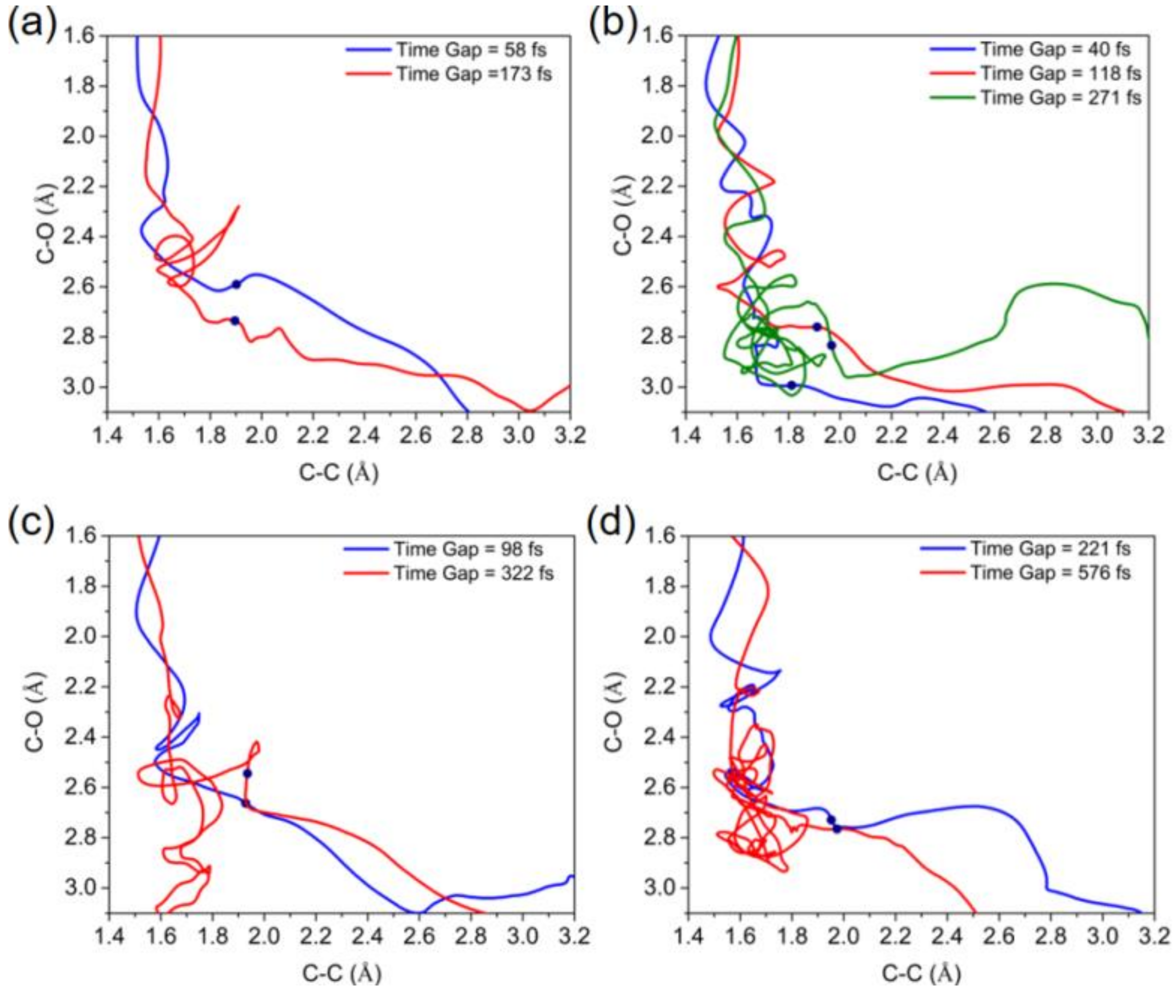

Supplementary Figure 31. Correlation between the forming C-O and C-C bond distances. The correlation between the C-O and C-C bond distances in two representative trajectories for the five-coordinate (a) quartet Fe form in the gas phase and (b) sextet Fe form in the gas phase, as well as for the five-coordinate quartet Fe form in solution in the absence (c) and presence (d) of an OEEF were ploted.

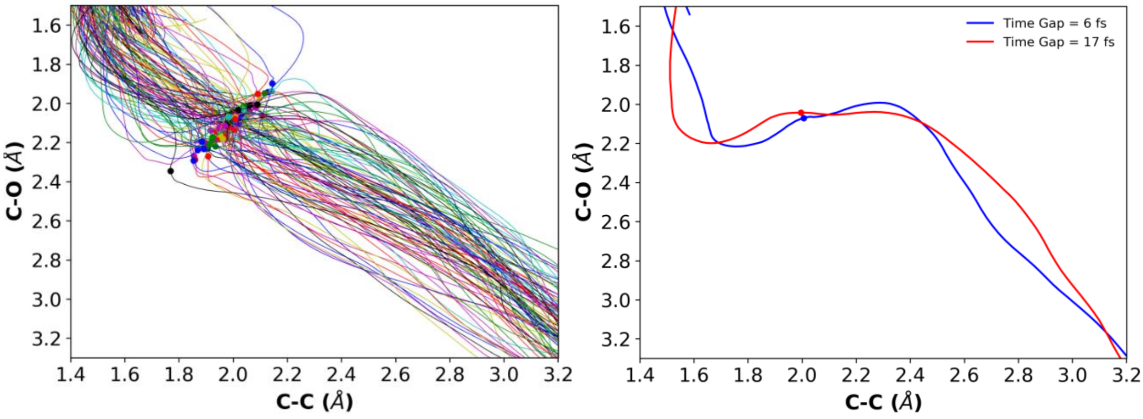

Supplementary Figure 32. Productive trajectories for the uncatalyzed ODA reaction in solution. The (left) productive and (right) two representative trajectories for the uncatalyzed ODA reaction in solution were ploted.

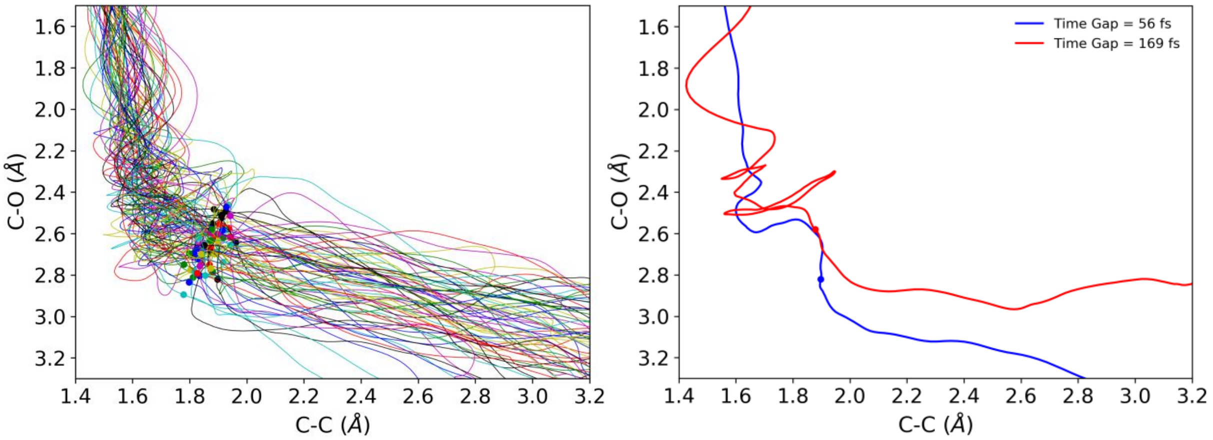

Supplementary Figure 33. Productive trajectories for the six-coordinate ^{4}Fe-catalyzed ODA reaction in solution. The (left) productive and (right) two representative trajectories for the six-coordinate and quartet state Fe-catalyzed ODA reaction in solution were ploted.

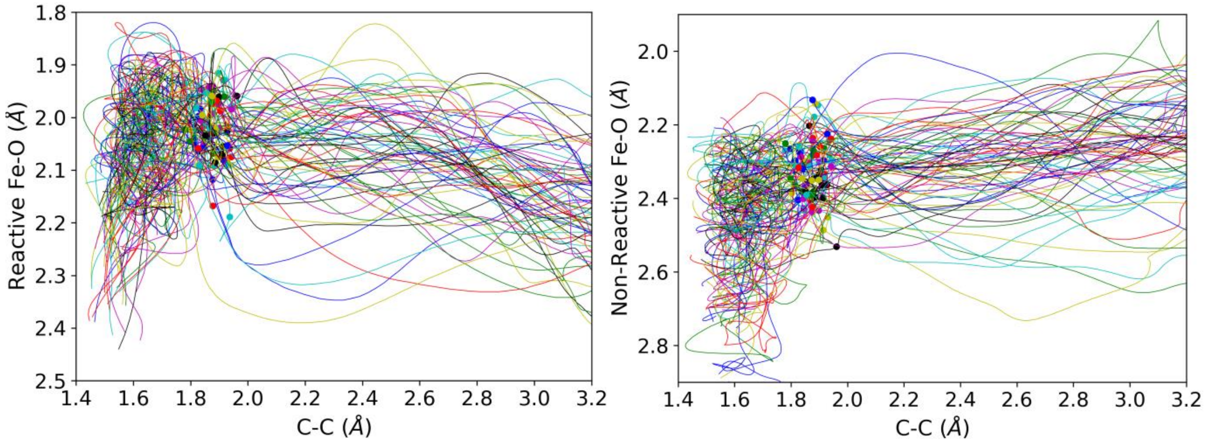

Supplementary Figure 34. Correlation between the Fe-O bond and the forming C-C bond in the six-coordinate ^{4}Fe-catalyzed ODA reaction trajectories in solution. The correlation between the C-C bond forming distance and (left) the reacting Fe-O or (right) the non-reacting Fe-O bond distance in the productive trajectories for the six-coordinate and quartet state Fe-catalyzed ODA reaction in solution were ploted.

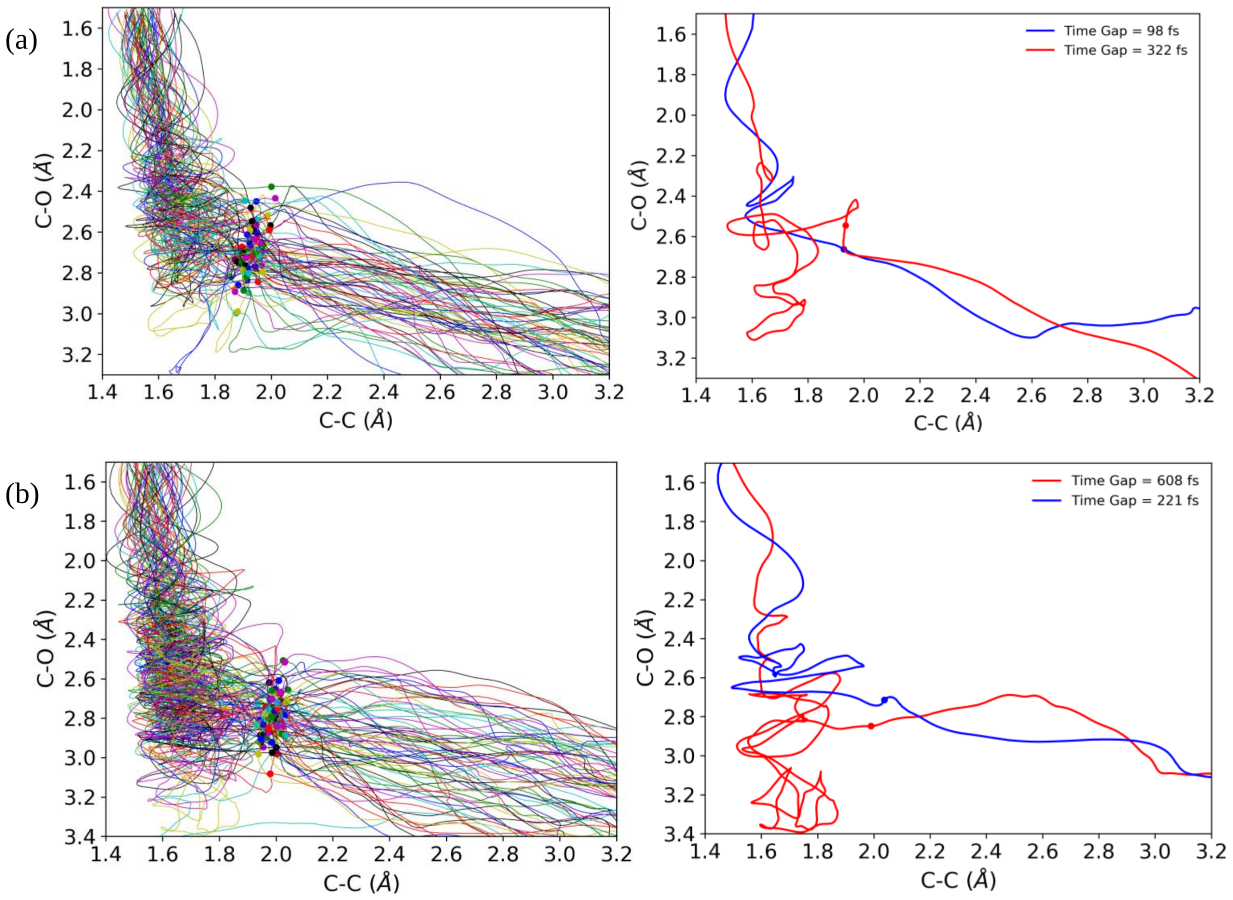

Supplementary Figure 35. Trajectories for the five-coordinate Fe-catalyzed ODA reaction in solution. The productive (left) and two representative trajectories (right) for the five-coordinate (a) quartet state and (b) sextet state Fe-catalyzed ODA reaction in solution were ploted.

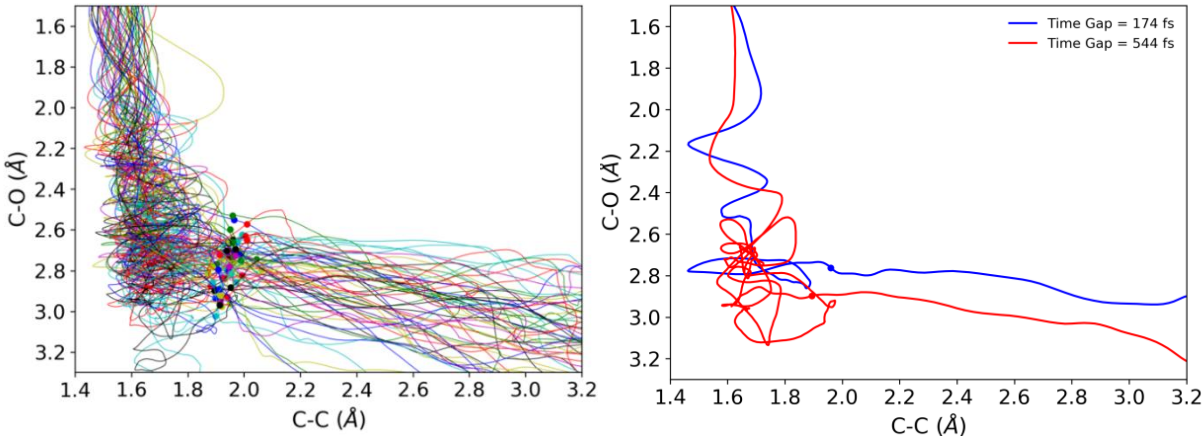

Supplementary Figure 36. Trajectories for the six-coordinate ^{4}Fe-catalyzed ODA reaction in solution with an OEEF. The productive (left) and two representative trajectories (right) for the six-coordinate and quartet state Fe-catalyzed ODA reaction in solution in the presence of an OEEF (with strength of -0.003 au) were ploted.

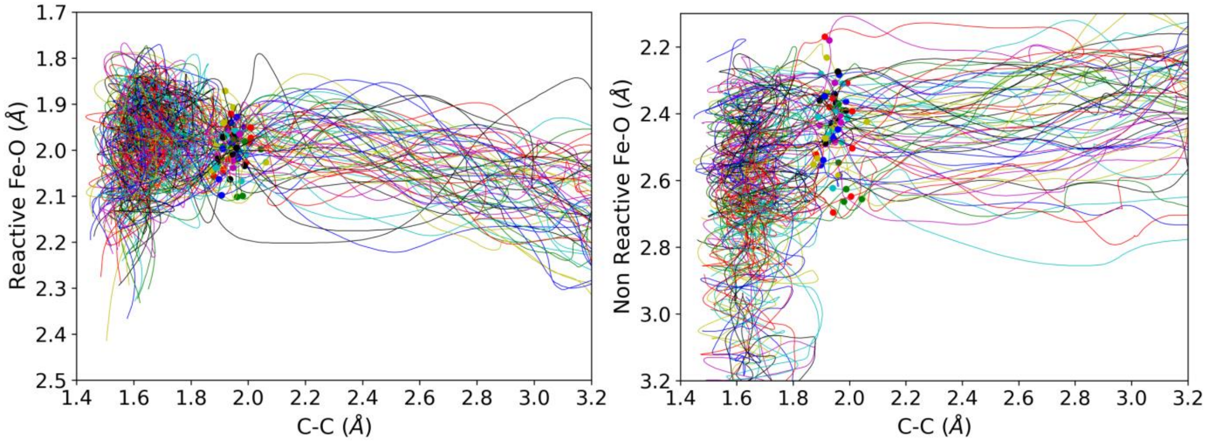

Supplementary Figure 37. Correlation between the Fe-O bond and the forming C-C bond in the six-coordinate ^{4}Fe-catalyzed ODA reaction trajectories in solution under OEEF. The correlation between the forming C-C bond distance and (left) the reacting Fe-O or (right) the non-reacting Fe-O bond distance in the productive trajectories for the six-coordinate and quartet state Fe-catalyzed ODA reaction in solution in the presence of an OEEF (with strength of -0.003 au) were ploted.

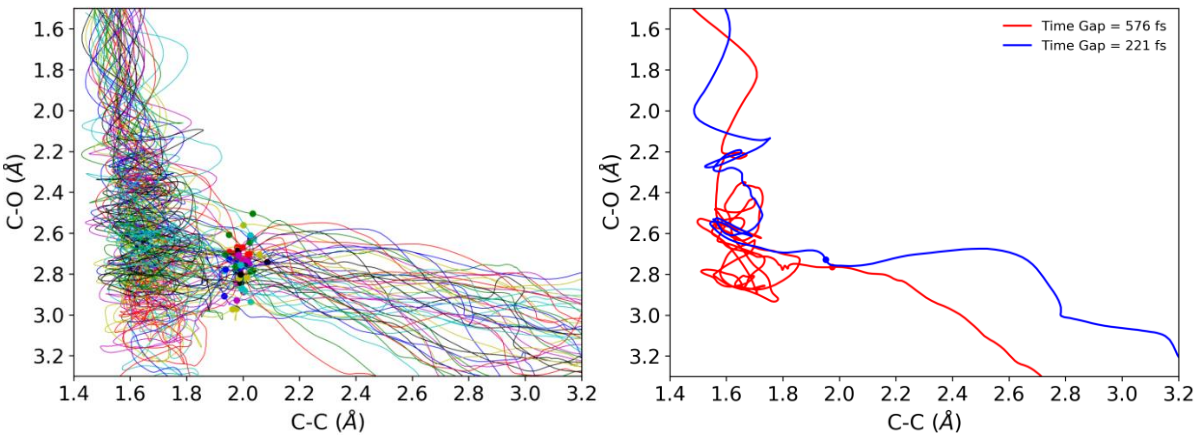

Supplementary Figure 38. Trajectories for the five-coordinate ^{4}Fe-catalyzed ODA reaction in solution with an OEEF. The (left) productive and (right) two representative trajectories for the five-coordinate and quartet state Fe-catalyzed ODA reaction in solution in the presence of an OEEF (with strength of -0.003 au) were ploted.

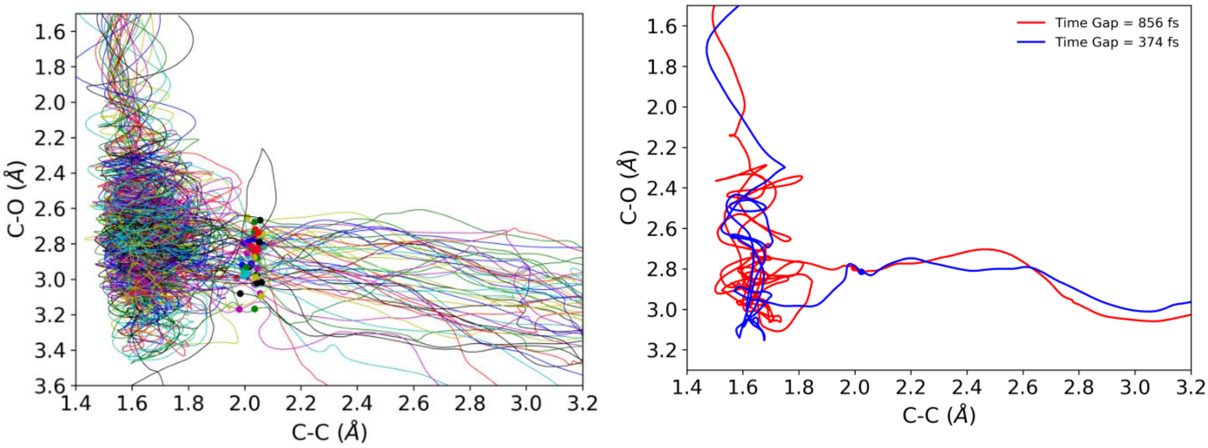

Supplementary Figure 39. Trajectories for the five-coordinate ^{6}Fe-catalyzed ODA reaction in solution with an OEEF. The (left) productive and (right) two representative trajectories for the five-coordinate and sextet state Fe-catalyzed ODA reaction in solution in the presence of an OEEF (with strength of -0.003 au) were ploted.

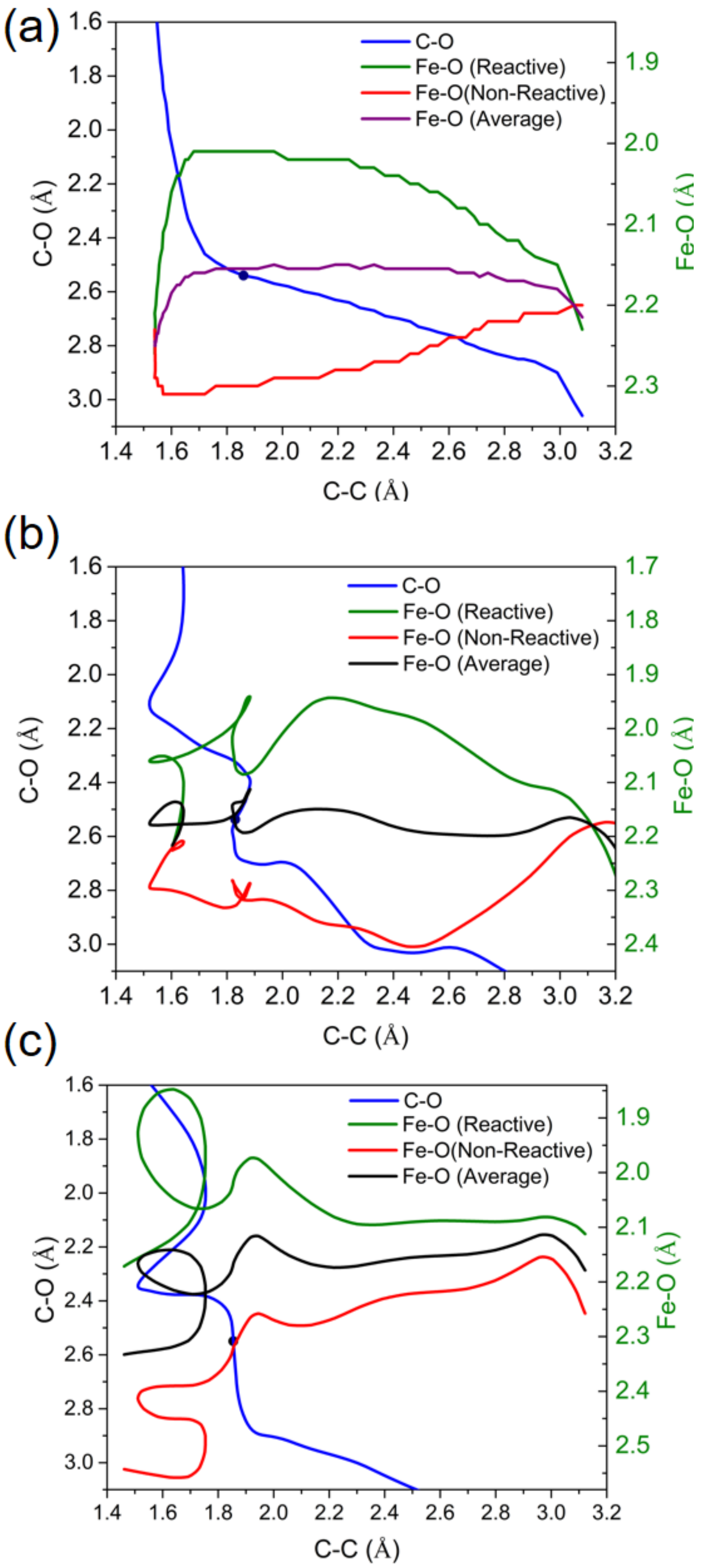

Supplementary Figure 40. Bond correlation in representative gas-phase trajectories. The correlation of the C-O, reacting Fe-O and non-reacting Fe-O bond distances versus the C-C bond forming distance for the (a) IRC and two trajectories with time gaps of (b) 26 fs and (c) 40 fs for the 6-coordinate quartet case in the gas phase were ploted.

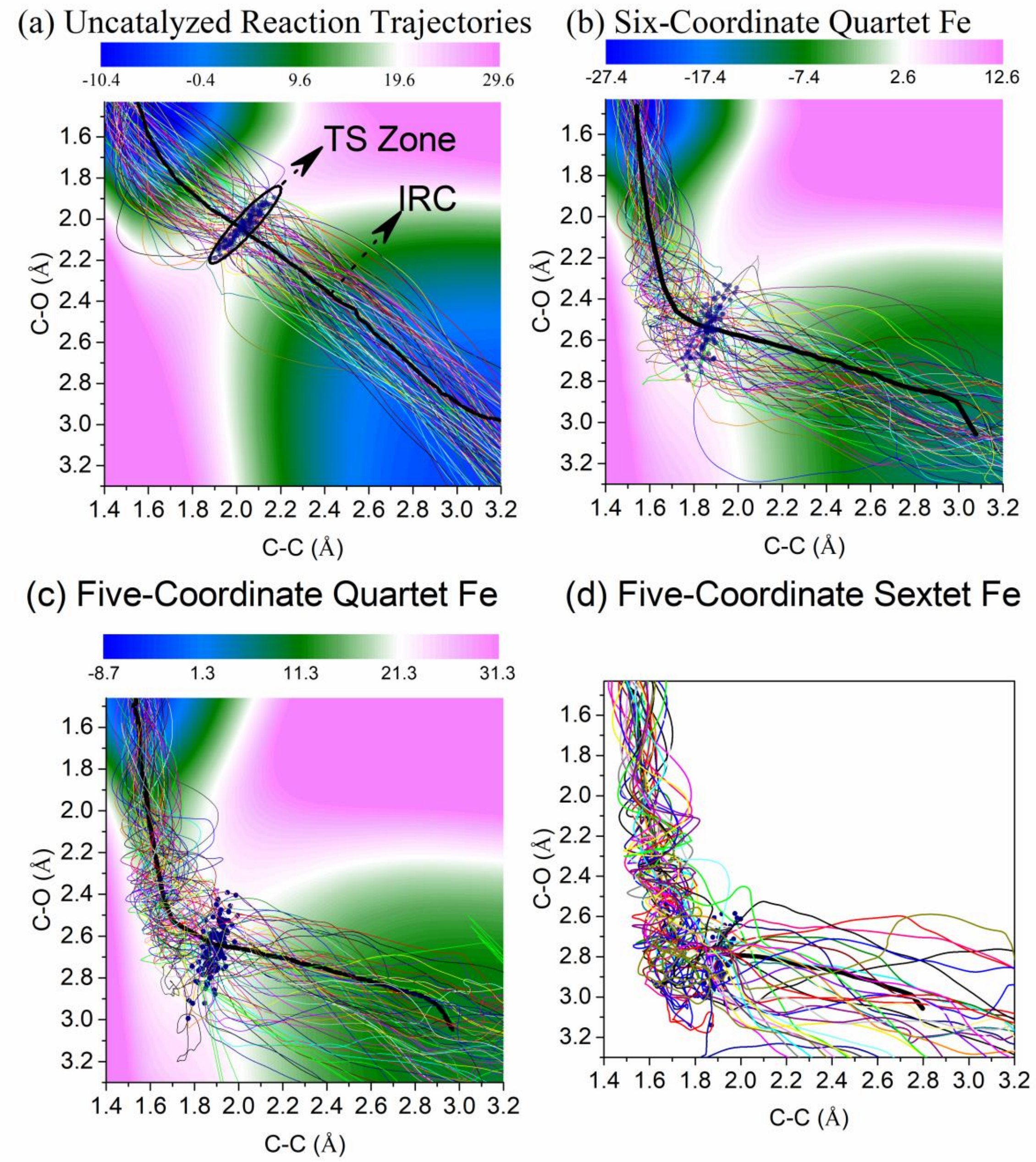

Supplementary Figure 41. Gas-phase productive trajectories for the formation of product A. Trajectories for (a) the uncatalyzed reaction and the Fe-catalyzed reaction of (b) the six-coordinate and quartet state, (c) the five-coordinate and quartet state, and (d) the five-coordinate and sextet state. The contour plots (energy in units of kcal mol^{-1}) were computed with respect to the isolated reactants (it should note that the color bars of the potential energy surfaces have different scales). The intrinsic reaction coordinate (IRC) is shown in bold, and transition state (TS) zone is defined as a zone including the sampled TS structures.

Supplementary Tables

Supplementary Table 1. Free energy profiles (in kcal mol^{-1}) of the reaction barrier and reaction energy for the *endo*-type ODA reactions without the Fe catalyst (i.e., the uncatalyzed reaction) in solution at 353.15 K by the SMD B3LYP-D3//B3LYP-D3 method**.** Notably, the standard state correction was applied here.

Type	$\triangle G^{\neq}_{soln}$	$\triangle G_{soln}$
**A**	36.0/34.7[g]/38.4[h]	0.8/-7.2[g]/-7.1[h]
**B**	35.1[a]	-0.2[a]
	35.9[b]	0.5[b]
**C**	35.7[a]	1.8[a]
	38.1[b]	-0.5[b]
**D**	32.3[c]	1.1[c]
	39.4[d]	-1.6[d]
**E1**	35.9[c,e]	1.0[c,e]
	43.5[d,e]	
	31.6[c,f]	-8.4[c,f]
	40.5[d,f]	
**E2**	33.7[c,e]	-0.6[c,e]
	41.0[d,e]	
	22.1[c,f]	-17.4[c,f]
	30.7[d,f]	
**F**	39.9	3.0

[a.] the *para*-product. [b.] the *meta*-product. [c.] the *cis*-product. [d.] the *trans*-product. [e.] Reaction with the C=O bond of the dienophile. [f.] Reaction with the C=C bond of the dienophile. [g.] The results from optimization by the M06-D3 method. [h.] The results obtained from optimization with the M06-2X-D3 method.

Supplementary Table 2. The relative free (electronic) energies (in kcal mol^{-1} at 353.15 K) for the key intermediates and transition states to form **A** in quartet (Q) and sextet (S) states optimized by other SMD DFT methods and single-point caculations by the DLPNO-CCSD(T) and B2PLYP-D3 methods in gas phase based on the SMD B3LYP-D3-optimized structures. Notably, the standard state correction was applied here.

	**LCCSD(T) (△E)[a]△G[b]**	**B2PLYP-D3 (△E)[a]△G[b]**	**PBE0-D3**	**B3PW91-D3**	**PW6B95-D3**	**BP86-D3**
**^{4}IA**	(0.0)0.0	(0.0)0.0	0.0	0.0	0.0	0.0
**^{6}IA**	(-9.8)-11.2	(4.6)3.2	2.9	7.7	5.2	1.7
**^{4}TS1A$_{endo}$**	(5.5)24.4	(6.1)24.9	20.7	22.1[c]	24.7	14.4[d]
**^{6}TS1A$_{endo}$**	(-8.2)7.7	(7.5)23.4	19.2	21.8	25.4	26.3
**^{4}TS2A$_{endo}$**	(25.2)30.4	(23.3)28.4	20.8	21.1	24.6	13.7
**^{6}TS2A$_{endo}$**	(13.3)15.1	(25.3)27.1	18.4	20.3	28.2	26.7
	**M06-L**	**B3LYP*-D3**	**OLYP-D3**	**TPSSh-D3**	**OPBE-D3**	**ωB97XD**
**^{4}IA**	0.0	0.0	0.0	0.0	0.0	0.0
**^{6}IA**	-4.5	15.4	6.0	8.9	4.4	6.4
**^{4}TS1A$_{endo}$**	26.3	18.2	18.7	20.9	14.6	21.6
**^{6}TS1A$_{endo}$**	19.4	28.7	19.8	27.2	17.1	24.3
**^{4}TS2A$_{endo}$**	25.0[e]	19.9	18.4	20.1	14.3	23.2
**^{6}TS2A$_{endo}$**	18.1	31.1	21.0	28.1	17.0	21.9

[a.]The relative electronic energies (in kcal mol^{-1}) in gas phase based on the SMD B3LYP-D3-optimized structures. [b.] The relative free energies (in kcal mol^{-1}) in solution based on the SMD B3LYP-D3 method. [c.] One additional small imaginary frequency (-6.96) exits. [d.] One additional small imaginary frequency (-9.27) exits. [e.] One additional small imaginary frequency (-10.92) exits.

Supplementary Table 3A. The relative free energies (in kcal mol^{-1}) of the key structures in solution for the formation of **A** evaluated by the other DFT methods based on the B3LYP-D3-optimized structures. Their computed reaction energies to form **A** are also given. Notably, the standard state correction was applied here.

	**B3PW91-D3**	**PBE0-D3**	**ωB97XD**
	**6-Coordinate Mode Pathway**		
^{2}**IA**	10.6	12.8	8.7
^{4}**IA**	0.0	0.0	0.0
^{6}**IA**	4.8	1.3	5.4
^{2}**TS1A**$_{endo}$	23.8	28.5	25.4
^{4}**TS1A**$_{endo}$	15.5	18.5	20.0
^{6}**TS1A**$_{endo}$	17.0	16.1	21.7
^{2}**IIIA**$_{endo}$	-0.8	1.9	-2.4
^{4}**IIIA**$_{endo}$	-14.4	-13.8	-13.6
^{6}**IIIA**$_{endo}$	-9.3	-12.1	-7.8
^{2}**VIIA**$_{exo}$	25.6	31.6	27.8
^{4}**VIIA**$_{exo}$	19.9	23.6	23.9
^{6}**VIIA**$_{exo}$	18.2	18.4	23.3
	**5-Coordinate Mode Pathway**		
^{2}**TS2A**$_{endo}$	33.0	38.0	35.5
^{4}**TS2A**$_{endo}$	17.7	19.9	20.7
^{6}**TS2A**$_{endo}$	19.3	18.1	22.1
^{2}**VIA**$_{endo}$	7.3	9.7	6.9
^{4}**VIA**$_{endo}$	-9.1	-8.8	-9.5
^{6}**VIA**$_{endo}$	-2.2	-5.3	-2.6
^{2}**VIIIA**$_{exo}$	30.7	36.7	33.7
^{4}**VIIIA**$_{exo}$	20.6	23.7	23.3
^{6}**VIIIA**$_{exo}$	18.3	17.6	21.6

Supplementary Table 3B. The reaction energy of formation of **A** by different SMD DFT methods in solution. Notably, the standard state correction was applied here.

**B3LYP-D3**	**B3PW91-D3**	**PBE0-D3**	**M06-D3**	**M06-2X-D3**
0.5	0.3	-11.5	-7.4	-6.5

Supplementary Table 4. The computed Mulliken charge of the key carbon atoms of the diene parts in the key structures for the formation of **A** in different spin states (Q: quartet; S: sextet) by the SMD B3LYP-D3//B3LYP-D3 method.

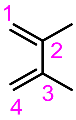

		q(C1)	q(C2)	q(C3)	q(C4)
Free diene		-0.43	0.19	0.19	-0.43
**TSA$_{noFe}$**		-0.43	0.17	0.14	-0.27
**TS1A$_{endo}$**	Q	-0.42	0.20	0.16	-0.30
	S	-0.43	0.21	0.17	-0.31
**IIIA$_{endo}$**	Q	-0.37	0.14	0.08	-0.11
	S	-0.36	0.14	0.08	-0.11
**TS2A$_{endo}$**	Q	-0.43	0.21	0.17	-0.31
	S	-0.43	0.21	0.17	-0.32
**VIA$_{endo}$**	Q	-0.37	0.14	0.08	-0.10
	S	-0.37	0.14	0.08	-0.11

Supplementary Table 5. The computed key properties (atomic charge and orbital energy) of the key structures for the formation of **A** in different spin states (D: doublet; Q: quartet; S: sextet) by the SMD B3LYP-D3//B3LYP-D3 method.

		q(Fe)/q(O)/q(C$_{C=O}$)[a]	q(C$_{diene}$)[b]	$E_{LUMO,C=O}$	$E_{HOMO,diene}$	$E_{HOMO,C=O}$	$E_{LUMO,diene}$
diene		-	-0.43/-0.43	-	-6.09 eV	-	-0.44 eV
aldehyde		-/-0.41/0.19	-	-1.62 eV	-	-6.89 eV	-
**TSA$_{noFe}$**		-/-0.49/0.10	-0.43/-0.27	-	-	-	-
**IA**	D	1.18/-0.45/0.21	-	-3.50 eV	-	-9.43 eV	-
	Q	1.27/-0.46/0.21	-	-3.16 eV	-	-8.18 eV	-
	S	1.39/-0.47/0.21	-	-3.29 eV	-	-8.62 eV	-
**TS1A$_{endo}$**	D	1.21/-0.55/0.07	-0.43/-0.31	-	-	-	-
	Q	1.28/-0.57/0.07	-0.42/-0.30	-	-	-	-
	S	1.39/-0.59/0.07	-0.43/-0.31	-	-	-	-
**IVA**	D	1.10/-0.46/0.23	-	-3.79 eV	-	-9.71 eV	-
	Q	1.16/-0.47/0.23	-	-3.60 eV	-	-9.30 eV	-
	S	1.22/-0.50/0.24	-	-3.89 eV	-	-9.72 eV	-
**TS2A$_{endo}$**	D	1.13/-0.54/0.09	-0.44/-0.32	-	-	-	-
	Q	1.18/-0.56/0.09	-0.43/-0.31	-	-	-	-
	S	1.23/-0.58/0.09	-0.43/-0.32	-	-	-	-

[a.] The computed Mulliken charge of the carbonyl carbon. [b.] The computed Mulliken charge of the two terminal diene carbon atoms.

Supplementary Table 6. The computed Mulliken charge distribution on the key groups of the key structures for the formation of **A** by the SMD B3LYP-D3//B3LYP-D3 and SMD B3LYP-D3 (in parentheses) methods.

	q(diene)	q(PhCHO)	q(Fe)	q(por)[c]
**TSA$_{noFe}$**	0.23<br>(0.23)	-0.23<br>(-0.23)	N/A	N/A
**^{4}IA**	N/A	0.15, 0.15<br>(0.15, 0.15)	1.27<br>(1.26)	-0.57<br>(-0.56)
**^{6}IA**	N/A	0.18, 0.18<br>(0.17, 0.17)	1.39<br>(1.38)	-0.75<br>(-0.72)
**^{4}TS1A$_{endo}$**	0.46<br>(0.41)	-0.23[a], 0.14[b]<br>(-0.18[a], 0.14[b])	1.28<br>(1.28)	-0.65<br>(-0.65)
**^{6}TS1A$_{endo}$**	0.47<br>(0.46)	-0.20[a], 0.16[b]<br>(-0.18[a], 0.15[b])	1.39<br>(1.38)	-0.82<br>(-0.81)
**^{4}IIIA$_{endo}$**	0.38<br>(0.38)	-0.27[a], 0.16[b]<br>(-0.27[a], 0.17[b])	1.30<br>(1.30)	-0.57<br>(-0.58)
**^{6}IIIA$_{endo}$**	0.41<br>(0.39)	-0.27[a], 0.18[b]<br>(-0.27[a], 0.17[b])	1.42<br>(1.47)	-0.74<br>(-0.76)
**^{4}IVA**	N/A	0.22<br>(0.22)	1.16<br>(1.17)	-0.38<br>(-0.39)
**^{6}IVA**	N/A	0.25<br>(0.25)	1.22<br>(1.24)	-0.47<br>(-0.49)
**^{4}TS2A$_{endo}$**	0.48<br>(0.46)	-0.16<br>(-0.15)	1.19<br>(1.19)	-0.51<br>(-0.50)
**^{6}TS2A$_{endo}$**	0.50<br>(0.47)	-0.15<br>(-0.13)	1.23<br>(1.23)	-0.58<br>(-0.57)
**^{4}VIA$_{endo}$**	0.44<br>(0.44)	-0.24<br>(-0.24)	1.22<br>(1.22)	-0.42<br>(-0.42)
**^{6}VIA$_{endo}$**	0.48<br>(0.45)	-0.24<br>(-0.23)	1.29<br>(1.39)	-0.53<br>(-0.61)

[a.] The group charge for the reacting PhCHO. [b.] The group charge for the nonreacting PhCHO. [c.] The group charge for the porphyrin ligand.

Supplementary Table 7. The NPA charge distribution of the key groups of the key structures for the formation of **A** in different spin states (Q: quartet; S: sextet) by the SMD B3LYP-D3//B3LYP-D3 method.

		q(diene)	q(PhCHO)	q(Fe)	q(por)[c]
**TSA_{noFe}**		0.26	-0.26	N/A	N/A
**IA**	Q	N/A	0.20, 0.20	0.80	-0.20
	S	N/A	0.22, 0.22	1.11	-0.55
**$TS1A_{endo}$**	Q	0.50	-0.19[a], 0.17[b]	0.77	-0.25
	S	0.52	-0.15[a], 0.18[b]	1.09	-0.64
**$IIIA_{endo}$**	Q	0.40	-0.21[a], 0.20[b]	0.80	-0.19
	S	0.42	-0.20[a], 0.22[b]	1.10	-0.54
**IVA**	Q	N/A	0.24	0.91	-0.15
	S	N/A	0.26	1.25	-0.51
**$TS2A_{endo}$**	Q	0.52	-0.16	0.88	-0.24
	S	0.54	-0.16	1.22	-0.60
**VIA_{endo}**	Q	0.43	-0.21	0.92	-0.14
	S	0.45	-0.21	1.24	-0.48

[a.] The group charge for the reacting PhCHO. [b.] The group charge for the non-reacting PhCHO. [c.] The group charge for the porphyrin ligand.

Supplementary Table 8. The computed distance of the iron atom displacement out of the porphyrin (its four N mean) plane (Δd_{4N}) of the key structures for the formation of **A** in different spin states (D: doublet; Q: quartet; S: sextet) by the SMD B3LYP-D3//B3LYP-D3 and SMD B3LYP (in parentheses) methods.

		Δd_{4N}
[a]**IA**	D	0.00(0.00)
	Q	0.00(0.01)
	S	0.00(0.02)
[a]**$TS1A_{endo}$**	D	0.04(0.05)
	Q	0.09(0.10)
	S	0.14(0.17)
[b]**$IIIA_{endo}$**	D	0.04
	Q	0.02(0.02)
	S	0.05
[b]**IVA**	D	0.21
	Q	0.19(0.16)
	S	0.35
[a]**$TS2A_{endo}$**	D	0.21(0.20)
	Q	0.22(0.22)
	S	0.41(0.42)

Supplementary Table 9. The dispersion effect on the reaction barrier and reaction energy (kcal mol^{-1}) to form **A** by the SMD B3LYP-D3 (with dispersion) and SMD B3LYP (no dispersion) methods. The net approximate dispersion effect is given in parentheses. The dispersion effects on the uncatalyzed *exo*-ODA reaction are also presented for comparison. Notably, the standard state correction was applied here.

	**Reaction Barrier**		**Reaction Energy**	
	With dispersion	No dispersion	With dispersion	No dispersion
**Uncatalyzed ODA Reaction**				
***Endo*-type**	37.3(-7.8)	45.1	0.5(-7.6)	8.1
***Exo*-type**	38.5(-6.7)	45.2	-2.9(-4.7)	1.8
**Fe-Catalyzed *endo*-type ODA Reaction**				
**6-c ^{4}Fe**[a]	24.2(-16.9)	41.1	-2.6(-15.4)	12.8
**6-c ^{6}Fe**[a]	25.6(-16.2)	41.8	-[c]	-[c]
**5-c ^{4}Fe**[b]	24.5(-7.5)	32.0	0.4(-8.8)	9.2
**5-c ^{6}Fe**[b]	24.8(-8.3)	33.1	6.1(-7.4)	13.5

[a.] 6-c stands for the 6-coordinate mode pathway. [b.] 5-c stands for the 5-coordinate mode pathway. [c.] Not considered in our calculations.

Supplementary Table 10. The relative free energies (in kcal mol^{-1}) of the key structures for the formation of **A** in solution in the absence and presence of OEEF by the SMD B3LYP method. All computed free energies are relative to the isolated **^{4}IA** and diene without the OEEF. Notably, the standard state correction was applied here.

	**No OEEF**	**OEEF (-0.0015 AU)**	**OEEF (-0.003 AU)**
**^{4}IIA$_{endo}$**	6.8	5.3	4.3
**^{4}TS1A$_{endo}$**	24.2(17.4[a])	19.2(13.9[a])	16.4(12.1[a])
**^{4}IIIA$_{endo}$**	-2.6	-3.2	-3.7
**^{4}VA$_{endo}$**	11.5	6.8	2.6
**^{4}TS2A$_{endo}$**	24.5(13.0[a])	20.4(13.6)	14.8(12.2[a])
**^{6}TS2A$_{endo}$**	24.8(13.3[a])	21.7(14.9[a])	16.6(14.0[a])
**^{4}VIA$_{endo}$**	0.4	-1.0	-3.6
**^{6}VIA$_{endo}$**	6.1	3.9	0.6

[a.] These barrier was estimated relative to their preceding intermediate (**^{4}IIA$_{endo}$** or **^{4}VA$_{endo}$**).

Supplementary Table 11. The computed Mulliken charge distribution of the key groups of the key structures for the formation of **A** in the presence of OEEF with strength of -0.0015 au and -0.003 au (in parenthesis) by the SMD B3LYP-D3 method.

	q(diene)	q(PhCHO)	q(Fe)	q(por)[c]
**^{4}IIA**	0.01	0.15[a],0.14[b]	1.27	-0.56
	(0.02)	(0.17[a],0.12[b])	(1.26)	(-0.56)
**^{4}TS1A$_{endo}$**	0.46	-0.20[a],0.12[b]	1.27	-0.65
	(0.47)	(-0.18[a],0.10[b])	(1.26)	(-0.65)
**^{4}IIIA$_{endo}$**	0.41	-0.28[a],0.14[b]	1.30	-0.57
	(0.43)	(-0.28[a],0.13[b])	(1.29)	(-0.58)
**^{4}VA**	0.02	0.21	1.18	-0.41
	(0.02)	(0.23)	(1.17)	(-0.42)
**^{4}TS2A$_{endo}$**	0.47	-0.14	1.18	-0.51
	(0.47)	(-0.12)	(1.17)	(-0.52)
**^{6}TS2A$_{endo}$**	0.47	-0.11	1.23	-0.59
	(0.48)	(-0.08)	(1.23)	(-0.63)
**^{4}VIA$_{endo}$**	0.46	-0.24	1.21	-0.42
	(0.48)	(-0.24)	(1.20)	(-0.43)
**^{6}VIA$_{endo}$**	0.49	-0.24	1.29	-0.54
	(0.52)	(-0.24)	(1.27)	(-0.55)

[a.] The group charge for the reacting PhCHO. [b.] The group charge for the non-reacting PhCHO. [c.] The group charge for the porphyrin ligand.

Supplementary Table 12. The main bond lengths and spin of iron for the structures to form **A** optimized in gas phase by the B3LYP-D3 method.

**A**		Fe-O	C-C	C-O	Fe-N_{mean}	**Spin(Fe)**
**6-Coordinate Mode**						
**IIA_{endo}**	D	1.96	3.16	3.34	2.00	1.04
	Q	2.19	3.18	3.28	2.00	2.88
	S	2.13	3.19	3.25	2.06	4.26
**$TS1A_{endo}$**	D	1.88	1.90	2.72	2.01	1.02
	Q	2.01	1.86	2.54	2.01	2.84
	S	1.96	1.90	2.66	2.07	4.24
**$IIIA_{endo}$**	D	1.99	1.54	1.48	2.01	1.03
	Q	2.21	1.54	1.46	2.00	2.87
	S	2.15	1.54	1.47	2.05	4.25
**IIA_{exo}**	D	1.96	4.19	3.62	2.01	1.05
	Q	2.22	3.44	3.25	2.01	2.88
	S	2.14	3.25	3.20	2.06	4.26
**$TS1A_{exo}$**	D	1.89	1.93	2.83	2.01	1.02
	Q	2.01	1.82	2.66	2.01	2.82
	S	1.96	1.90	2.77	2.07	4.24
**$IIIA_{exo}$**	D	2.00	1.54	1.47	2.00	1.03
	Q	2.24	1.54	1.46	2.00	2.88
	S	2.17	1.54	1.46	2.06	4.25
**$VIIA_{exo}$**	D	1.81	1.54	-	2.02	0.95
	Q	1.91	1.56	-	2.02	2.73
	S	1.85	1.57	-	2.09	4.18
**5- Coordinate Mode**						
**VA_{endo}**	D	1.95	3.09	3.25	2.00	1.14
	Q	2.07	3.14	3.24	2.00	2.84
	S	2.02	3.08	3.18	2.06	4.18
**$TS2A_{endo}$**	D	1.85	1.94	2.77	2.00	1.09
	Q	1.95	1.89	2.64	2.01	2.79
	S	1.92	1.89	2.78	2.08	4.17
**VIA_{endo}**	D	1.97	1.54	1.48	2.00	1.14
	Q	2.09	1.54	1.46	2.01	2.84
	S	2.04	1.54	1.47	2.06	4.17
**VA_{exo}**	D	1.96	4.19	3.62	2.00	1.05
	Q	2.22	3.44	3.25	2.00	2.88
	S	2.14	3.25	3.20	2.07	4.26
**$TS2A_{exo}$**	D	1.89	1.93	2.83	2.00	1.02
	Q	1.95	1.90	2.75	2.01	2.82

	S	1.96	1.90	2.77	2.08	4.24
**VIA$_{exo}$**	D	2.00	1.54	1.47	2.01	1.03
	Q	2.12	1.54	1.47	1.99	2.88
	S	2.17	1.54	1.46	2.07	4.25
**VIIIA$_{exo}$**	D	1.79	1.54	-	2.01	1.02
	Q	1.83	1.57	-	2.02	2.69
	S	1.83	1.54	-	2.09	4.14

Supplementary Table 13. Experimental results of KIE for the formation of A .

Entry	Product (mmol)	Yield (%)	Conversion (%)	KIE
1	0.281	48	60	0.918
2	0.271	45	60	0.924
3	0.271	46	59	0.936

The average value of KIE is 0.926, with a standard deviation of 0.007. (95% confidence interval: ±0.008).

Supplementary Table 14. Computed secondary deuterium (k_H/k_D) KIE and EIE results for the formation of **A** in quartet (Q) and sextet (S) states optimized by other DFT methods and SMD method in solution at 353.15 K.

	**B3LYP-D3**	**PBE0-D3**	**B3PW91-D3**
**EIE**			
**^{4}IA → ^{4}IVA**	1.069	1.082	1.080
**KIE**			
**^{4}TS1A$_{endo}$**	0.905	0.902	0.918[b]
**^{6}TS1A$_{endo}$**	0.902	0.887	0.914
**^{4}TS2A$_{endo}$**	0.985	0.975	0.992
**^{6}TS2A$_{endo}$**	N/A[a]	0.950	0.972
	**M06-L**	**B3LYP*-D3**	**OLYP-D3**
**EIE**			
**^{4}IA → ^{4}IVA**	1.103	1.068	1.079
**KIE**			
**^{4}TS1A$_{endo}$**	0.900	0.877	0.888
**^{6}TS1A$_{endo}$**	0.880	0.877	0.887
**^{4}TS2A$_{endo}$**	0.951[c]	0.975	0.951
**^{6}TS2A$_{endo}$**	0.934	0.962	N/A[a]

[a.] A significant numerical problem was found. [b.] One additional small imaginary frequency (-6.96) exits. [c.] One additional small imaginary frequency (-10.92) exits.

Supplementary Table 15. The relative free energies (in kcal mol^{-1}) for the transition states to form **A** in the doublet and quartet states catalyzed by the cationic Ru porphyrin complex in solution at 353.15 K by the SMD B3LYP-D3//B3LYP-D3 method. Notably, the standard state correction was applied here.

**IA_{Ru}**	D	0.0
	Q	40.1
**$TS1A_{endo,Ru}$**	D	23.5
	Q	59.4
**$TS1A_{exo,Ru}$**	D	24.6

Supplementary Table 16. The relative energies (in kcal mol^{-1} at 298.15 K) of the key structures to form **A** with replacing one PhCHO ligand by 5-methyl-1H-imidazole (his) ligand (see Supplementary Figure 9) in diethylether solution[a] by the SMD B3LYP-D3 method. Notably, the standard state correction was applied here.

	**$\Delta E_{ZPE}/\Delta G$**[b]	**$\Delta E_{ZPE\text{-}PBE0}$**[c]	**$\Delta E_{ZPE\text{-}\omega B97XD}$**[d]
**$^{2}IA_{his}$**	0.9/4.0	3.1	-1.0
**$^{4}IA_{his}$**	0.0/0.0	0.0	0.0
**$^{6}IA_{his}$**	4.8/4.8	-0.3	3.9
**$^{2}TS1A_{endo,his}$**	7.5/22.6	8.0	5.3
**$^{4}TS1A_{endo,his}$**	9.3/21.7	7.5	9.6
**$^{6}TS1A_{endo,his}$**	11.8/24.8	5.1	10.9
**$^{2}IIIA_{endo,his}$**	-16.3/-0.2	-22.3	-26.8
**$^{4}IIIA_{endo,his}$**	-20.6/-6.7	-28.8	-28.7
**$^{6}IIIA_{endo,his}$**	-15.3/-1.7	-28.5	-24.2

[a.] Diethylether (its dielectric constant of ~4.2) was used to mimics hydrophobic enzymatic environment. [b.] These results were obtained by the SMD B3LYP-D3 method. [c.] These results were obtained by the SMD PBE0-D3//SMD B3LYP-D3 method. [d.] These results were obtained by the SMD ωB97XD//SMD B3LYP-D3 method.

Supplementary Table 17. The relative free (electronic) energies (in kcal mol^{-1} at 298.15 K) of the cationic Fe(III) complexes with replacing PhCHO by acetone (see Supplementary Figure 10) in acetone solution by the SMD B3LYP-D3 method and other methods based on the SMD B3LYP-D3 optimized structures.

	**B3LYP-D3**	**LCCSD(T)[a] (△E)**	**LCCSD(T)[b] △G**	**PBE0-D3**	**B3PW91-D3**
**^{2}IG**	13.9	-	-	16.1	14.4
**^{4}IG**	0.0	(0.0)	0.0	**0.0**	0.0
**^{6}IG**	6.4	(-10.4)	-11.8	**1.3**	4.9
**^{2}IG$_{4Ph}$**	13.4	-	-	17.1	14.7
**^{4}IG$_{4Ph}$**	0.0	-	-	**0.0**	0.0
**^{6}IG$_{4Ph}$**	5.8	-	-	**0.8**	4.5
	**PW6B95D3**	**M06L**	**OLYP-D3**	**OPBE-D3**	**wB97XD**
**^{2}IG**	14.8	12.9	16.8	16.0	11.6
**^{4}IG**	0.0	0.0	0.0	0.0	0.0
**^{6}IG**	4.4	-3.8	6.6	5.5	5.4
**^{2}IG$_{4Ph}$**	16.3	16.1	13.9	14.9	14.8
**^{4}IG$_{4Ph}$**	0.0	0.0	0.0	0.0	0.0
**^{6}IG$_{4Ph}$**	4.1	-4.4	6.3	5.3	5.4

[a.] The relative electronic energies (in kcal mol^{-1}) in gas phase based on the SMD B3LYP-D3-optimized structures. [b.] The relative free energies (in kcal mol^{-1}) in solution based on the SMD B3LYP-D3 method.

Supplementary Table 18. The relative free energies (in kcal mol^{-1}) of the transition states (the most favorable ones highlighted in blue) to form **B** in solution by the SMD B3LYP-D3//B3LYP-D3 and SMD B3LYP-D3 (in parenthesis) methods. Notably, the standard state correction was not applied here.

**B**	coordinate	TS	Doublet	Quartet	Sextet
para	6-c	**TS1B$_{endo-p}$**	27.6	20.1(**23.6**)	22.5
		**TS1B'$_{endo-p}$**	-	21.3	-
		**TS1B$_{exo-p}$**	30.1	21.3	23.9
		**TS1B'$_{exo-p}$**	-	21.2	-
	5-c	**TS2B$_{endo-p}$**	32.2	19.3(**21.5**)	21.6(23.5)
		**TS2B'$_{endo-p}$**	33.3	21.0(24.2)	23.9
		**TS2B$_{exo-p}$**	33.0	20.3	20.8
		**TS2B'$_{exo-p}$**	-	20.7	-
meta	6-c	**TS1B$_{endo-m}$**	-	26.4(**27.4**)	-
		**TS1B'$_{endo-m}$**	34.7	27.0	30.4
		**TS1B$_{exo-m}$**	36.8	27.2	30.1
		**TS1B'$_{exo-m}$**	-	29.7	-
	5-c	**TS2B$_{endo-m}$**	40.0	28.1	29.9
		**TS2B'$_{endo-m}$**	-	26.7	-
		**TS2B$_{exo-m}$**	39.5	26.1(**25.6**)	28.4
		**TS2B'$_{exo-m}$**	-	28.5	-

Supplementary Table 19. The computed Mulliken charge and orbital analysis of the diene part in the key structures to form **B** in different spin states (Q: quartet; S: sextet) by the SMD B3LYP-D3//B3LYP-D3 method.

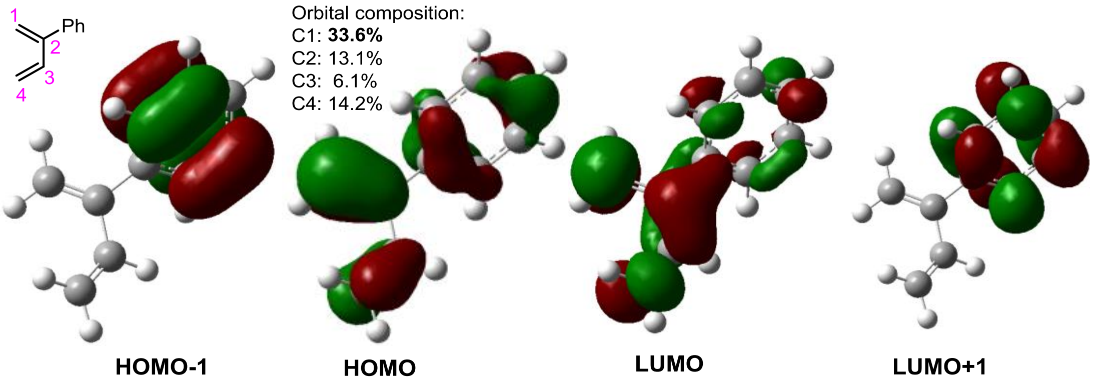

		q(C1)	q(C2)	q(C3)	q(C4)
Free diene		-0.42	0.14	-0.11	-0.37
**TS2B$_{endo-p}$**	Q	-0.44	0.18	-0.13	-0.28
	S	-0.45	0.18	-0.13	-0.29
**TS2B$_{exo-m}$**	Q	-0.30	0.13	-0.07	-0.38
	S	-0.31	0.13	-0.07	-0.38

Supplementary Table 20. The main bond lengths and spin of iron for the structures to form **B** optimized in gas phase by the B3LYP-D3 method. (D: doublet; Q: quartet; S: sextet)

B		Fe-O	C-C	C-O	Spin(Fe)
***para*-product**					
**IIB**$_{\mathbf{endo\text{-}p}}$	D	1.95	2.98	3.46	1.04
	Q	2.18	3.04	3.39	2.88
	S	2.12	3.56	4.17	4.26
**TS1B**$_{\mathbf{endo\text{-}p}}$	D	1.88	1.92	2.86	1.02
	Q	2.00	1.86	2.69	2.82
	S	1.95	1.94	2.83	4.24
**IIIB**$_{\mathbf{endo\text{-}p}}$	D	1.99	1.54	1.48	1.03
	Q	2.22	1.54	1.46	2.87
	S	2.16	1.54	1.47	4.25
**TS1B'**$_{\mathbf{endo\text{-}p}}$	Q	2.02	1.94	2.65	2.83
**IIB**$_{\mathbf{exo\text{-}p}}$	D	1.97	3.81	3.96	1.04
	Q	2.21	3.67	3.24	2.88
	S	2.15	3.37	3.12	4.26
**TS1B**$_{\mathbf{exo\text{-}p}}$	D	1.89	2.00	2.82	1.02
	Q	2.02	1.91	2.70	2.83
	S	1.97	1.97	2.77	4.24
**IIIB**$_{\mathbf{exo\text{-}p}}$	D	2.00	1.55	1.48	1.04
	Q	2.25	1.55	1.46	2.88
	S	2.19	1.55	1.47	4.25
**TS1B'**$_{\mathbf{exo\text{-}p}}$	Q	2.00	1.89	2.72	2.83
**VB'**$_{\mathbf{endo\text{-}p}}$	D	1.93	3.24	3.73	1.17
	Q	2.07	3.09	3.53	2.84
	S	2.01	3.05	3.54	4.18
**TS2B'**$_{\mathbf{endo\text{-}p}}$	D	1.87	2.07	2.74	1.10
	Q	1.96	2.00	2.71	2.79
	S	1.91	2.04	2.77	4.16
**VIB'**$_{\mathbf{endo\text{-}p}}$	D	1.97	1.54	1.49	1.14
	Q	2.10	1.54	1.48	2.84
	S	2.04	1.54	1.49	4.17
**VB**$_{\mathbf{endo\text{-}p}}$	Q	2.06	3.10	3.51	2.85
**TS2B**$_{\mathbf{endo\text{-}p}}$	D	1.88	2.00	2.76	1.10
	Q	1.95	1.93	2.77	2.79
	S	1.91	2.06	2.79	4.16

**VIB$_{endo-p}$**	Q	2.09	1.54	1.48	2.84
**VB$_{exo-p}$**	D	1.92	4.44	3.86	1.19
	Q	2.08	4.38	3.94	2.84
	S	2.02	4.31	3.85	4.18
**TS2B$_{exo-p}$**	D	1.88	2.04	2.80	1.11
	Q	1.95	1.95	2.77	2.78
	S	1.91	2.01	2.83	4.17
**VIB$_{exo-p}$**	D	1.99	1.54	1.48	1.14
	Q	2.12	1.54	1.47	2.84
	S	2.06	1.54	1.48	4.17
**TS2B'$_{exo-p}$**	Q	1.97	1.96	2.77	2.79

***meta*-product**					
**TS1B$_{endo-m}$**	Q	2.04	1.91	2.38	2.85
**IIB'$_{endo-m}$**	D	1.96	3.47	4.34	1.05
	Q	2.18	3.29	4.23	2.88
	S	2.11	3.22	4.01	4.26
**TS1B'$_{endo-m}$**	D	1.88	1.87	2.55	1.02
	Q	2.05	1.93	2.30	2.85
	S	1.97	1.89	2.40	4.24
**IIIB'$_{endo-m}$**	D	1.99	1.55	1.48	1.03
	Q	2.22	1.55	1.46	2.87
	S	2.16	1.55	1.47	4.25
**IIB$_{exo-m}$**	D	1.95	4.25	3.50	1.05
	Q	2.23	3.14	3.45	2.89
	S	2.15	3.17	3.50	4.26
**TS1B$_{exo-m}$**	D	1.90	1.85	2.67	1.02
	Q	2.06	1.86	2.42	2.85
	S	1.98	1.84	2.56	4.24
**IIIB$_{exo-m}$**	D	2.01	1.55	1.47	1.04
	Q	2.25	1.55	1.45	2.88
	S	2.23	1.55	1.46	4.25
**TS1B'$_{exo-m}$**	Q	2.04	1.81	2.49	2.84
**VB$_{endo-m}$**	D	1.95	3.24	5.33	1.25
	Q	2.10	3.28	5.42	2.84
	S	2.04	3.23	5.40	4.17
**TS2B$_{endo-m}$**	D	1.87	1.93	2.59	1.10

	Q	1.98	1.88	2.47	2.80
	S	1.92	1.86	2.63	4.16
**VIB$_{endo-m}$**	D	2.00	1.55	1.47	1.14
	Q	2.12	1.55	1.47	2.84
	S	2.05	1.55	1.47	4.17
**TS2B'$_{endo-m}$**	Q	1.96	1.87	2.49	2.80
**VB$_{exo-m}$**	D	1.95	4.18	3.45	1.21
	Q	2.07	3.09	3.43	2.84
	S	2.01	3.07	3.43	4.16
**TS2B$_{exo-m}$**	D	1.88	1.90	2.70	1.12
	Q	1.97	1.86	2.53	2.80
	S	1.92	1.86	2.67	4.17
**VIB$_{exo-m}$**	D	2.24	1.55	1.47	2.00
	Q	2.15	1.54	1.46	2.85
	S	2.07	1.54	1.47	4.17
**TS2B'$_{exo-m}$**	Q	1.96	1.82	2.60	2.78

Supplementary Table 21. The computed Mulliken charge and orbital analysis of the diene of the key structures to form **C** in the quartet state by the SMD B3LYP-D3//B3LYP-D3 method.

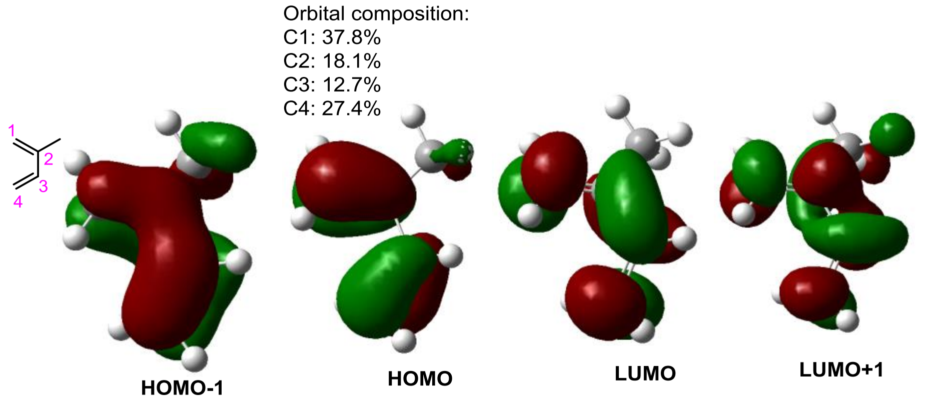

	q(C1)	q(C2)	q(C3)	q(C4)
Free diene	-0.41	0.19	-0.12	-0.35
^{4}**TS2C$_{endo-p}$**	-0.43	0.25	-0.12	-0.26
^{4}**TS2C$_{exo-m}$**	-0.30	0.19	-0.05	-0.38

Supplementary Table 22. The main bond lengths and spin of iron for the key structures in the quartet state to form **C** optimized in gas phase by the B3LYP-D3 method.

C	Fe-O	C-C	C-O	Spin(Fe)
**IIC$_{endo-p}$**	2.22	3.04	3.07	2.89
**TS1C$_{endo-p}$**	2.01	1.86	2.56	2.84
**IIIC$_{endo-p}$**	2.22	1.54	1.45	2.88
**TS2C$_{endo-p}$**	1.95	1.88	2.67	2.79
**TS1C$_{exo-p}$**	2.01	1.81	2.64	2.88

Supplementary Table 23. The relative free energies (in kcal mol^{-1}) of the key structures (the favorable ones highlighted in blue) to form **D** in solution at 353.15 K by the SMD B3LYP-D3//B3LYP-D3 and SMD B3LYP-D3 (in parenthesis) methods. Notably, the standard state correction was not applied here.

D			Quartet	Sextet
cis	6-c	**TS1D$_{endo-cis-p}$**	17.5(**17.7**)	19.2
		**TS1D$_{exo-cis-p}$**	24.2	-
	5-c	**TS2D$_{endo-cis-p}$**	17.0(18.2)	19.0(21.8)
trans	6-c	**TS1D$_{endo-trans-p}$**	25.2	-
		**TS1D$_{exo-trans-p}$**	18.1(21.3)	21.0
	5-c	**TS2D$_{exo-trans-p}$**	17.8(**20.0**)	18.9(20.5)
**Stepwise pathway**				
cis	5-c	**TS2D$_{cc-endo-cis-p}$**	16.6(19.9)	18.1(21.3)
		**TS2D$_{co-endo-cis-p}$**	17.3(19.1)	17.9
trans	5-c	**TS2D$_{cc-exo-trans-p}$**	18.5(19.8)	19.2(20.1)
		**TS2D$_{co-exo-trans-p}$**	18.2	18.5

Supplementary Table 24. The main bond lengths (in angstrom) and spin of iron of the key structures to form **D** optimized in gas phase by the B3LYP-D3 method. (Q: quartet; S: sextet)

D		Fe-O	C-C	C-O	Spin(Fe)
**IID$_{endo-cis-p}$**	Q	2.18	3.00	3.30	2.87
**TS1D$_{endo-cis-p}$**	Q	2.02	1.92	2.75	2.83
	S	1.97	1.97	2.83	4.24
**IIID$_{endo-cis-p}$**	Q	2.33	1.54	1.48	2.83
**TS2D$_{endo-cis-p}$**	Q	1.97	1.96	2.82	2.78
	S	1.93	2.02	2.87	4.17
**IID$_{exo-trans-p}$**	Q	2.18	4.17	5.10	2.88
**TS1D$_{exo-trans-p}$**	Q	2.04	1.94	2.81	2.83
	S	1.98	2.01	2.85	4.24
**IIID$_{exo-trans-p}$**	Q	2.39	1.56	1.47	2.88
**TS2D$_{exo-trans-p}$**	Q	1.97	2.02	2.88	2.80
	S	1.92	2.13	2.86	4.16

Supplementary Table 25. The relative free energies (in kcal mol^{-1}) of the key structures (reaction with the C=O or C=C bond) to form **E1O** or **E1C** in solution at 353.15 K by the SMD B3LYP-D3//B3LYP-D3 and SMD B3LYP-D3 (in parenthesis) methods. (p: The *para* position of the benzene ring relative to oxygen) The intermediates with the carbon-carbon bond forming in the stepwise process are marked in gray. The favorable structures are highlighted in blue. Notably, the standard state correction was not applied here.

**E1**			Quartet	Sextet
Reaction with the C=O bond	6-c	**TS1E1O$_{endo\text{-}cis\text{-}p}$**	23.4(**25.1**)[1.6[a]]	25.4
		**VIIE1O$_{endo\text{-}cis\text{-}p}$**	(26.7)	(26.4)
		**TS1E1O$_{endo\text{-}trans\text{-}p}$**	-	31.1
		**TS1E1O$_{exo\text{-}cis\text{-}p}$**	29.2	29.9
		**TS1E1O$_{exo\text{-}trans\text{-}p}$**	24.4	26.9
	5-c	**TS2E1O$_{endo\text{-}cis\text{-}p}$**	24.2(**24.5**)	25.4
Reaction with the C=C bond	6-c	**TS1E1C$_{endo\text{-}cis\text{-}p}$**	27.8	30.2
		**TS1E1C$_{endo\text{-}trans\text{-}p}$**	34.1	(39.0)
		**TS1E1C$_{exo\text{-}cis\text{-}p}$**	34.8	37.5
		**TS1E1C$_{exo\text{-}trans\text{-}p}$**	25.6(**28.6**)[-5.1[a]]	29.6
		**VIIE1C$_{exo\text{-}trans\text{-}p}$**	(32.4)	33.3(39.0)
	5-c	**TS2E1C$_{exo\text{-}trans\text{-}p}$**	27.8(29.3)	30.5

[a.] Reaction energies

Supplementary Table 26. The relative free energies (in kcal mol^{-1}) of the less favorable *meta*-type transition states to form **E1C** in the quartet state in solution at 353.15 K by the SMD B3LYP-D3//B3LYP-D3. (m: The *meta* position of the benzene ring relative to oxygen) Notably, the standard state correction was not applied here.

**E1**			Quartet
Reaction with C=C	6-c	**TS1E1C$_{endo\text{-}cis\text{-}m}$**	34.2
		**TS1E1C$_{endo\text{-}trans\text{-}m}$**	39.3
		**TS1E1C$_{exo\text{-}cis\text{-}m}$**	40.1
		**TS1E1C$_{exo\text{-}trans\text{-}m}$**	35.9
	5-c	**TS2E1C$_{endo\text{-}cis\text{-}m}$**	35.3
		**TS2E1C$_{endo\text{-}trans\text{-}m}$**	41.0
		**TS2E1C$_{exo\text{-}cis\text{-}m}$**	43.3
		**TS2E1C$_{exo\text{-}trans\text{-}m}$**	38.5

Supplementary Table 27. The main bond lengths (in angstrom) and spin of iron for the key structures to form **E1O** and **E1C** optimized in gas phase by the B3LYP-D3 method. (Q: quartet; S: sextet)

**E1**		**Fe-O**	**C-C**	**C-O**	**Spin(Fe)**
**Reaction with the C=O bond**					
**IIE1O$_{endo-cis-p}$**	Q	2.17	3.11	3.28	2.87
**TS1E1O$_{endo-cis-p}$**	Q	2.01	1.84	2.76	2.81
	S	1.96	1.90	2.86	4.23
**IIIE1O$_{endo-cis-p}$**	Q	2.25	1.53	1.45	2.87
**TS2E1O$_{endo-cis-p}$**	Q	1.96	1.90	2.90	2.78
	S	1.96	1.90	2.86	4.23
**TS1E1O$_{exo-trans-p}$**	Q	2.03	1.93	2.74	2.82
	S	1.97	1.99	2.85	4.23
**Reaction with the C=C bond**					
				**C-C**	
**TS1E1C$_{endo-cis-p}$**	Q	2.08	2.04	2.96	2.85
	S	1.99	2.05	3.07	4.24
**IIE1C$_{exo-trans-p}$**	Q	2.17	4.09	3.99	2.87
**TS1E1C$_{exo-trans-p}$**	Q	2.08	2.03	2.86	2.84
	S	2.04	2.05	2.92	4.25
**IIIE1C$_{exo-trans-p}$**	Q	2.23	1.55	1.56	2.87
**TS2E1C$_{exo-trans-p}$**	Q	2.00	2.06	2.95	2.81
	S	2.02	2.05	2.92	4.25

Supplementary Table 28. The relative free energies (in kcal mol^{-1}) of the key structures (via reaction with the C=O or C=C bond) to form **E2O** or **E2C** in solution at 353.15 K by the SMD B3LYP-D3//B3LYP-D3 and SMD B3LYP-D3 (in parenthesis) methods. (p: The *para* position of the benzene ring relative to oxygen). The favorable structures are highlighted in blue. Notably, the standard state correction was not applied here.

**E2**			Quartet	Sextet
Reaction with the C=O bond	6-c	**TS1E2O$_{endo-cis-p}$**	20.7(22.7)[-3.3[a]]	22.9
		**TS1E2O$_{exo-trans-p}$**	21.0[3.1[a]]	24.1
	5-c	**TS2E2O$_{endo-cis-p}$**	18.7(**18.3**)	20.8
Reaction with the C=C bond	6-c	**TS1E2C$_{endo-cis-p}$**	16.0(18.1)[-16.0[a]]	19.9
		**TS1E2C$_{exo-trans-p}$**	16.1(18.2)[-15.9[a]]	19.7
	5-c	**TS2E2C$_{endo-cis-p}$**	17.8(**16.2**)	18.0
**Stepwise-TS**				
Reaction with the C=O bond	5-c	**TS2E2O$_{cc-endo-cis-p}$**	19.7(19.7)	20.9
		**TS2E2O$_{co-endo-cis-p}$**	19.8(19.3)	19.9
Reaction with	5-c	**TS2E2C$_{cc1-endo-cis-p}$**	18.8(20.9)	20.7

the C=C bond		**TS2E2C$_{cc2\text{-}endo\text{-}cis\text{-}p}$**	-	8.5

[a.] Reaction energies

Supplementary Table 29. The relative free energies (in kcal mol^{-1}) of the less favorable *meta*-type transition states (via reaction with the C=C bond) to form **E2C** in the quartet state in solution at 353.15 K by the SMD B3LYP-D3//B3LYP-D3. (m: The *meta* position of the benzene ring relative to oxygen) Notably, the standard state correction was not applied here.

**E2**			Quartet
Reaction with the C=C bond	6-c	**TS1E2C$_{endo\text{-}cis\text{-}m}$**	24.1
		**TS1E2C$_{endo\text{-}trans\text{-}m}$**	26.9
		**TS1E2C$_{exo\text{-}cis\text{-}m}$**	27.9
		**TS1E2C$_{exo\text{-}trans\text{-}m}$**	22.8
	5-c	**TS2E2C$_{endo\text{-}cis\text{-}m}$**	21.4
		**TS2E2C$_{endo\text{-}trans\text{-}m}$**	26.3
		**TS2E2C$_{exo\text{-}cis\text{-}m}$**	28.0
		**TS2E2C$_{exo\text{-}trans\text{-}m}$**	25.5

Supplementary Table 30. The main bond lengths (in angstrom) and spin of iron for the structures to form **E2O** or **E2C** optimized in gas phase by the B3LYP-D3 method. (Q: quartet; S: sextet)

**E2**		**Fe-O**	**C-C**	**C-O**	**Spin(Fe)**
**Reaction with the C=O bond**					
**TS1E2O$_{endo\text{-}cis\text{-}p}$**	Q	2.03	1.90	2.81	2.82
	S	1.98	1.97	2.88	4.24
**IIIE2O$_{endo\text{-}cis\text{-}p}$**	Q	2.19	1.53	1.46	2.88
**TS2E2O$_{endo\text{-}cis\text{-}p}$**	Q	1.98	1.96	2.85	2.78
	S	1.94	2.03	2.90	4.17
**TS1E2O$_{exo\text{-}trans\text{-}p}$**	Q	2.04	1.96	2.77	2.83
	S	1.99	2.04	2.86	4.24
**IIIE2O$_{exo\text{-}trans\text{-}p}$**	Q	2.35	1.50	1.46	2.88
**Reaction with the C=C bond**					
				**C-C**	
**TS1E2C$_{endo\text{-}cis\text{-}p}$**	Q	2.10	2.12	3.06	2.87
	S	2.02	2.16	3.20	4.25
**IIIE2C$_{endo\text{-}cis\text{-}p}$**	Q	2.24	1.57	1.51	2.88
**TS2E2C$_{endo\text{-}cis\text{-}p}$**	Q	2.02	2.18	3.17	2.82
	S	1.97	2.27	3.27	4.17
**TS1E2C$_{exo\text{-}trans\text{-}p}$**	Q	2.10	2.12	2.91	2.86
	S	2.04	2.18	2.97	4.25
**IIIE2C$_{exo\text{-}trans\text{-}p}$**	Q	2.23	1.59	1.54	2.88

Supplementary Table 31. The relative free energies (in kcal mol^{-1}) of the key transition states (the favorable structures highlighted in blue) to form **F** in solution at 353.15 K by the SMD B3LYP-D3//B3LYP-D3 method. Notably, the standard state correction was applied here.

**F**			Quartet	Sextet
	6-c	**$TS1F_1$**	28.3	29.2
		**$TS1F_2$**	29.1	28.7
	5-c	**$TS2F_1$**	28.4	**27.1**
		**$TS2F_2$**	28.8	27.4

Supplementary Table 32. The main bond lengths (in angstrom) and spin of iron of the transition states to form **F** optimized in gas phase by the B3LYP-D3 method. (Q: quartet; S: sextet)

**F**		**Fe-O**	**C-C**	**C-O**	**Spin(Fe)**
**$TS1F_1$**	Q	1.99	1.89	2.96	2.81
	S	1.93	1.99	3.00	4.21
**$TS1F_2$**	Q	1.99	1.85	2.97	2.80
	S	1.93	1.97	3.02	4.22
**$TS2F_1$**	Q	1.95	1.95	2.98	2.76
	S	1.89	2.04	2.99	4.15
**$TS2F_2$**	Q	1.94	1.93	3.00	2.76
	S	1.88	2.02	3.02	4.15

Supplementary Table 33. Comparison of the number of the trajectories (N) and the productive trajectories (N_p), average time of the product formation (T_f, fs) and time gap of the two bond formation (T_g, fs) for the uncatalyzed and Fe-catalyzed *endo*-ODA reaction in the gas phase, solution (in parenthesis) and solution in the presence of an OEEF (with strength of -0.003 au, in square brackets) by the B3LYP-D3 and SMD B3LYP-D3 methods.

	N			N_p			T_f (fs)			T_g (fs)		
	Gas	Soln	Soln+OEEF	Gas	Soln	Soln+OEEF	Gas	Soln	Soln+OEEF	Gas	Soln	Soln+OEEF
Uncatalyzed	100	100	--	97	98	--	32	34	--	5	6	--
					**Fe-Catalyzed Reaction**							
6-c ^{4}Fe[a]	100	130	130	71	73	63	87	103	220	40	50	174
5-c ^{4}Fe[b]	130	130	130	71	72	50[c]	123	151	276[c]	59	98	235[c]
5-c ^{6}Fe[b]	100	130	130	35	76	34[d]	182	240	452[d]	103	199	414[d]

[a.] 6-c stands for the 6-coordinate mode pathway. [b.] 5-c stands for the 5-coordinate mode pathway. [c.] one trajectory does not form the C-O bond (<1.6 Å) after 900 fs, and thus the time should be longer than this value. [d.] 16 trajectories do not form the C-O bond (<1.6 Å) after 900 fs, and thus the time should be much longer than this value.

Supplementary Table 34. Percentage (%) of dynamics stepwise trajectories for each reactions in gas phase, solution (Soln) and solution phase in the presence of an OEEF.

Reaction	Gas (%)	Soln (%)	Soln+OEEF (%)
Uncatalyzed	0	0	--
6-c ^{4}Fe[a]	21	26	86
5-c ^{4}Fe[b]	39	54	88
5-c ^{6}Fe[b]	74	87	96

[a.] 6-c stands for the 6-coordinate mode pathway. [b.] 5-c stands for the 5-coordinate mode pathway.

Supplementary Table 35. The absolute and relative energies (in kcal mol^{-1}) for the distortion and interaction of the key transition states by the SMD B3LYP-D3//B3LYP-D3 method. (Q: quartet; S: sextet)

		$E^{\neq}_{dist,ML}$	$E^{\neq}_{dist,diene}$	$\triangle E^{\neq}$	$\triangle E^{\neq}_{dist,ML}$	$\triangle E^{\neq}_{dist,diene}$	$\triangle E^{\neq}_{int}$
**TS1A$_{endo}$**	Q	-2943.317981	-234.612128	4.8	22.0	18.5	35.6
	S	-2943.308346	-234.617145	1.4	20.5	15.3	34.5
**TS1A$_{exo}$**	Q	-2943.313848	-234.613599	4.4	24.6	17.6	37.8
	S	-2943.307408	-234.620220	1.1	21.1	13.4	33.4
**TS2A$_{endo}$**	Q	-2597.702489	-234.616019	1.9	20.4	16.0	34.5
	S	-2597.691312	-234.617414	-1.8	20.1	15.2	37.1
**TS2A$_{exo}$**	Q	-2597.701905	-234.619633	1.5	20.8	13.8	33.1
	S	-2597.694388	-234.623081	-2.3	18.2	11.6	32.1
**TS1B$_{endo-p}$**	Q	-2943.318995	-387.046755	2.0	21.4	14.5	33.8
	S	-2943.310688	-387.050964	-1.1	19.0	11.9	32.0
**TS2B$_{endo-p}$**	Q	-2597.707666	-387.049202	-0.8	17.2	13.0	31.0
	S	-2597.698087	-387.051239	-4.7	15.9	11.7	32.3
**TS1B$_{endo-m}$**	Q	-2943.321036	-387.039420	7.1	20.1	19.1	32.1
**TS2B$_{exo-m}$**	Q	-2597.698778	-387.046919	3.2	22.7	14.4	33.9
	S	-2597.687897	-387.045807	-0.2	22.3	15.1	37.5
**TS1C$_{endo-p}$**	Q	-2943.317600	-195.290965	5.9	22.2	19.2	35.5
**TS2C$_{endo-p}$**	Q	-2597.701114	-195.295656	2.6	21.3	16.2	34.9
**TS1D$_{endo-cis-p}$**	Q	-2943.322350	-426.372917	-2.0	19.3	12.9	34.2
	S	-2943.312654	-426.375301	-5.4	17.8	11.4	34.6
**TS2D$_{endo-cis-p}$**	Q	-2597.706671	-426.374632	-5.2	17.8	11.8	34.8
	S	-2597.697900	-426.376533	-8.8	16.0	10.6	35.4
**TS1D$_{exo-trans-p}$**	Q	-2943.323281	-426.374476	-1.2	18.7	11.9	31.8
	S	-2943.313907	-426.377585	-4.5	17.0	10.0	31.5
**TS2D$_{exo-trans-p}$**	Q	-2597.707361	-426.378237	-4.8	17.4	9.6	31.7
	S	-2597.703015	-426.382000	-9.0	12.8	7.2	28.9

**TS1E1O$_{endo\text{-}cis\text{-}p}$**	Q	-2793.273655	-426.368967	2.7	24.0	15.4	36.7
	S	-2793.266505	-426.372727	-1.1	21.6	13.0	35.7
**TS2E1O$_{endo\text{-}cis\text{-}p}$**	Q	-2522.681683	-426.371975	-1.0	21.3	13.5	35.8
	S	-2522.674688	-426.374860	-5.7	18.4	11.7	35.8
**TS1E1C$_{exo\text{-}trans\text{-}p}$**	Q	-2793.284808	-426.370217	4.4	17.0	14.6	27.2
	S	-2793.274341	-426.372309	2.3	16.7	13.3	27.7
**TS2E1C$_{exo\text{-}trans\text{-}p}$**	Q	-2522.689922	-426.372523	1.8	16.1	13.1	27.4
	S	-2522.681373	-426.375649	-1.6	14.2	11.2	27.0
**TS1E2O$_{endo\text{-}cis\text{-}p}$**	Q	-2635.972961	-426.372519	1.0	22.1	13.1	34.2
	S	-2635.964033	-426.375933	-2.8	19.9	11.0	33.7
**TS2E2O$_{endo\text{-}cis\text{-}p}$**	Q	-2444.031843	-426.375249	-2.7	19.2	11.4	33.3
	S	-2444.022872	-426.377555	-7.5	16.7	10.0	34.2
**TS1E2C$_{exo\text{-}trans\text{-}p}$**	Q	-2635.983079	-426.377111	-3.3	15.7	10.3	29.3
	S	-2635.994167	-426.379783	-5.2	1.0	8.6	14.8
**TS2E2C$_{endo\text{-}cis\text{-}p}$**	Q	-2444.047982	-426.380982	-6.4	9.1	7.8	23.3
	S	-2444.038952	-426.383612	-10.7	6.6	6.2	23.5
**TS1F$_1$**	Q	-2871.942895	-426.371463	8.6	29.5	13.8	34.7
	S	-2871.934178	-426.376776	2.7	25.9	10.5	33.7
**TS2F$_1$**	Q	-2562.011133	-426.373733	4.4	27.2	12.4	35.1
	S	-2562.004712	-426.376776	-1.8	23.9	10.5	36.3

Supplementary Table 36. The absolute energies (in Hartree) of the optimized structures to form **A** by the B3LYP-D3 method. (D: doublet; Q: quartet; S: sextet)

A		E	E+ZPE	G	E_{soln}	G(80°C)
**C_6H_{10}**		-234.636726	-234.493747	-234.523969	-234.641577	-234.531228
**PhCHO**		-345.581538	-345.471263	-345.501762	-345.591491	-345.508927
**6-Coordinate mode**						
**IA**	D	-2943.279196	-2942.776879	-2942.842978	-2943.343568	-2942.862383
	Q	-2943.289382	-2942.789111	-2942.858994	-2943.353050	-2942.879289
	S	-2943.277375	-2942.778881	-2942.849062	-2943.341033	-2942.869460
**IVA**	D	-2597.651090	-2597.262097	-2597.318421	-2597.713191	-2597.334436
	Q	-2597.673519	-2597.284741	-2597.343099	-2597.735012	-2597.359525
	S	-2597.661562	-2597.274551	-2597.334148	-2597.723399	-2597.350867
**IIA_{endo}**	D	-3177.933627	-3177.287084	-3177.363595	-3177.998743	-3177.386776
	Q	-3177.944507	-3177.300078	-3177.380724	-3178.009762	-3177.404872
	S	-3177.933311	-3177.290512	-3177.371152	-3177.998246	-3177.395333
**$TS1A_{endo}$**	D	-3177.916460	-3177.269442	-3177.343925	-3177.981292	-3177.366444
	Q	-3177.921773	-3177.276385	-3177.354635	-3177.986910	-3177.377988
	S	-3177.914715	-3177.271311	-3177.349892	-3177.980423	-3177.373393
**$IIIA_{endo}$**	D	-3177.957341	-3177.304992	-3177.376748	-3178.022187	-3177.398610
	Q	-3177.970326	-3177.320232	-3177.396524	-3178.035516	-3177.419435
	S	-3177.958308	-3177.309862	-3177.386096	-3178.023565	-3177.409036
**IIA_{exo}**	D	-3177.930531	-3177.284483	-3177.361483	-3177.995618	-3177.384805
	Q	-3177.941608	-3177.297358	-3177.379130	-3178.007010	-3177.403510
	S	-3177.929655	-3177.287241	-3177.369547	-3177.994995	-3177.394078
**$TS1A_{exo}$**	D	-3177.916672	-3177.269512	-3177.341832	-3177.980508	-3177.363910
	Q	-3177.922776	-3177.277085	-3177.352826	-3177.987664	-3177.375678
	S	-3177.916116	-3177.272339	-3177.349151	-3177.980913	-3177.372284
**$IIIA_{exo}$**	D	-3177.956226	-3177.303622	-3177.374700	-3178.020572	-3177.396413
	Q	-3177.970949	-3177.320496	-3177.396050	-3178.035957	-3177.418795
	S	-3177.958644	-3177.309913	-3177.385427	-3178.023389	-3177.408210
**$VIIA_{exo}$**	D	-3177.916651	-3177.267604	-3177.339323	-3177.981401	-3177.361271
	Q	-3177.913551	-3177.267421	-3177.344633	-3177.979230	-3177.369456
	S	-3177.914643	-3177.270724	-3177.346542	-3177.980466	-3177.369424

**5-Coordinate mode**						
**VA_{endo}**	D	-2832.307678	-2831.774322	-2831.840473	-2832.369866	-2831.860135
	Q	-2832.329140	-2831.796198	-2831.865033	-2832.391594	-2831.885251
	S	-2832.318081	-2831.786833	-2831.856636	-2832.380298	-2831.877087
**$TS2A_{endo}$**	D	-2832.292594	-2831.758672	-2831.821484	-2832.355581	-2831.840079
	Q	-2832.310540	-2831.776881	-2831.843131	-2832.373524	-2831.862563
	S	-2832.304309	-2831.772019	-2831.838251	-2832.367797	-2831.857736

**VIA$_{endo}$**	D	-2832.331752	-2831.792981	-2831.855530	-2832.394338	-2831.874128
	Q	-2832.353966	-2831.815335	-2831.879361	-2832.417076	-2831.898258
	S	-2832.341900	-2831.805333	-2831.867504	-2832.405194	-2831.886001
**VA$_{exo}$**	D	-2832.304355	-2831.771630	-2831.838918	-2832.359910	-2831.857822
	Q	-2832.326006	-2831.793546	-2831.862927	-2832.388817	-2831.883298
	S	-2832.315738	-2831.784760	-2831.855680	-2832.378023	-2831.876365
**TS2A$_{exo}$**	D	-2832.294327	-2831.760225	-2831.822837	-2832.356038	-2831.841534
	Q	-2832.312278	-2831.778600	-2831.843284	-2832.374209	-2831.862408
	S	-2832.306076	-2831.774144	-2831.840493	-2832.368645	-2831.860009
**VIA$_{exo}$**	D	-2832.329557	-2831.793727	-2831.857359	-2832.391556	-2831.876268
	Q	-2832.354906	-2831.816160	-2831.879352	-2832.417349	-2831.898075
	S	-2832.343792	-2831.806974	-2831.870546	-2832.406067	-2831.889418
**VIIIA$_{exo}$**	D	-2832.297263	-2831.760963	-2831.822158	-2832.360621	-2831.840546
	Q	-2832.306274	-2831.771235	-2831.835253	-2832.369728	-2831.855850
	S	-2832.307165	-2831.772925	-2831.837666	-2832.371297	-2831.856847

Supplementary Table 37. The absolute single-point energies (in Hartree) of the B3LYP-optimized structures to form **A** in solution by other DFT methods and SMD method. (D: doublet; Q: quartet; S: sextet)

**A**		**B3PW91-D3**	**PBE0-D3**	**ωB97XD**
**C_6H_{10}**		-234.553068	-234.338147	-234.550734
**PhCHO**		-345.457767	-345.184015	-345.462792
**IA**	D	-2942.657723	-2941.125054	-2942.676089
	Q	-2942.667932	-2941.138689	-2942.683188
	S	-2942.658120	-2941.134518	-2942.672340
**$TS1A_{endo}$**	D	-3177.213991	-3175.462347	-3177.224337
	Q	-3177.220958	-3175.472038	-3177.226674
	S	-3177.216141	-3175.473370	-3177.221529
**$IIIA_{endo}$**	D	-3177.261849	-3175.513501	-3177.277307
	Q	-3177.275799	-3175.530618	-3177.287448
	S	-3177.266027	-3175.526290	-3177.276561
**$VIIA_{exo}$**	D	-3177.216427	-3175.462807	-3177.225943
	Q	-3177.214293	-3175.464204	-3177.220803
	S	-3177.218094	-3175.473718	-3177.222972
**IVA**	D	-2597.159780	-2595.901497	-2597.172023
	Q	-2597.183278	-2595.928032	-2597.195244
	S	-2597.172896	-2595.922966	-2597.185001
**$TS2A_{endo}$**	D	-2831.720165	-2830.241838	-2831.724016
	Q	-2831.739999	-2830.266050	-2831.743066
	S	-2831.736062	-2830.267649	-2831.739442
**VIA_{endo}**	D	-2831.766255	-2830.291989	-2831.774818
	Q	-2831.790524	-2830.319621	-2831.799046
	S	-2831.779681	-2830.314262	-2831.788279
**$VIIIA_{exo}$**	D	-2831.728114	-2830.248152	-2831.731112
	Q	-2831.737827	-2830.262468	-2831.741459
	S	-2831.741391	-2830.272165	-2831.744027

Supplementary Table 38. The absolute energies (in Hartree) of the optimized structures to form **A** catalyzed by the Ru(III) complex by the B3LYP-D3 method. (D: doublet; Q: quartet)

**A**		**E**	**E+ZPE**	**G**	**E_{soln}**	**G(80°C)**
**IA_{Ru}**	D	-1542.220916	-1541.825016	-1541.883665	-1542.281989	-1541.900551
	Q	-1542.152821	-1541.760588	-1541.820632	-1542.212592	-1541.837896
**$TS1A_{endo,Ru}$**	D	-1776.851245	-1776.310023	-1776.376923	-1776.911136	-1776.396913
	Q	-1776.787976	-1776.250861	-1776.319050	-1776.848182	-1776.339389
**$TS1A_{exo,Ru}$**	D	-1776.852454	-1776.310859	-1776.375948	-1776.911823	-1776.395560

Supplementary Table 39. The absolute energies (in Hartree) of the optimized structures to form **B** by the B3LYP-D3 method. (D: doublet; Q: quartet; S: sextet)

**B**		**E**	**E+ZPE**	**G**	**E_{soln}**	**G(80°C)**
**$C_{10}H_{10}$**		-387.060399	-386.892820	-386.926515	-387.069886	-386.934794
***para*-product**						
**IIB_{endo-p}**	D	-3330.362876	-3329.691552	-3329.770329	-3330.430953	-3329.794289
	Q	-3330.373466	-3329.704200	-3329.787178	-3330.441867	-3329.812118
	S	-3330.362477	-3329.694898	-3329.779972	-3330.430280	-3329.805356
**$TS1B_{endo-p}$**	D	-3330.346527	-3329.674450	-3329.751951	-3330.414386	-3329.775404
	Q	-3330.351211	-3329.680906	-3329.762340	-3330.419687	-3329.786676
	S	-3330.344426	-3329.675907	-3329.758453	-3330.412694	-3329.783061
**$IIIB_{endo-p}$**	D	-3330.381414	-3329.703792	-3329.778343	-3330.451244	-3329.801045
	Q	-3330.394579	-3329.719004	-3329.797851	-3330.464817	-3329.821535
	S	-3330.382499	-3329.708661	-3329.787675	-3330.452694	-3329.811439
**$TS1B'_{endo-p}$**	Q	-3330.350332	-3329.680029	-3329.760946	-3330.418448	-3329.785182
**IIB_{exo-p}**	D	-3330.362995	-3329.691642	-3329.770357	-3330.430838	-3329.794342
	Q	-3330.372604	-3329.703679	-3329.788494	-3330.440309	-3329.813838
	S	-3330.357463	-3329.690307	-3329.773769	-3330.425467	-3329.798877
**$TS1B_{exo-p}$**	D	-3330.346616	-3329.674215	-3329.749531	-3330.413342	-3329.772536
	Q	-3330.353344	-3329.682684	-3329.761941	-3330.420872	-3329.785843
	S	-3330.346186	-3329.677384	-3329.757531	-3330.413666	-3329.781671
**$IIIB_{exo-p}$**	D	-3330.384058	-3329.705989	-3329.780004	-3330.452360	-3329.802559
	Q	-3330.398191	-3329.722170	-3329.800014	-3330.466879	-3329.823471
	S	-3330.385803	-3329.711608	-3329.790455	-3330.454339	-3329.814155
**$TS1B'_{exo-p}$**	Q	-3330.352889	-3329.682338	-3329.761447	-3330.420955	-3329.785323
**VB'_{endo-p}**	D	-2984.733655	-2984.175793	-2984.242948	-2984.799216	-2984.263014
	Q	-2984.755217	-2984.197486	-2984.268915	-2984.821529	-2984.289986
	S	-2984.744821	-2984.188729	-2984.261068	-2984.810440	-2984.282367
**$TS2B'_{endo-p}$**	D	-2984.719674	-2984.161027	-2984.228796	-2984.784471	-2984.250447
	Q	-2984.739318	-2984.180734	-2984.249592	-2984.804397	-2984.269891
	S	-2984.733391	-2984.176263	-2984.244627	-2984.798830	-2984.264877
**VIB'_{endo-p}**	D	-2984.756797	-2984.192562	-2984.258006	-2984.823499	-2984.277430
	Q	-2984.779056	-2984.215107	-2984.278612	-2984.846237	-2984.297531
	S	-2984.767037	-2984.205079	-2984.269559	-2984.834487	-2984.288738
**VB_{endo-p}**	Q	-2984.758297	-2984.200506	-2984.271310	-2984.823925	-2984.292255
**$TS2B_{endo-p}$**	D	-2984.724188	-2984.164975	-2984.231922	-2984.789399	-2984.251816
	Q	-2984.740654	-2984.182018	-2984.251585	-2984.806220	-2984.272015
	S	-2984.735082	-2984.177895	-2984.247619	-2984.800845	-2984.268131

**VIB$_{endo-p}$**	Q	-2984.778135	-2984.214183	-2984.280901	-2984.845924	-2984.300482
**VB$_{exo-p}$**	D	-2984.736301	-2984.177917	-2984.246504	-2984.801223	-2984.266979
	Q	-2984.759170	-2984.201096	-2984.271067	-2984.824490	-2984.291846
	S	-2984.749054	-2984.192804	-2984.264093	-2984.813794	-2984.285186
**TS2B$_{exo-p}$**	D	-2984.724202	-2984.165159	-2984.231227	-2984.789073	-2984.250954
	Q	-2984.743007	-2984.184205	-2984.251068	-2984.808039	-2984.270964
	S	-2984.737015	-2984.180014	-2984.249282	-2984.802442	-2984.269719
**VIB$_{exo-p}$**	D	-2984.758204	-2984.193712	-2984.258149	-2984.824734	-2984.277366
	Q	-2984.780929	-2984.216630	-2984.282690	-2984.847996	-2984.302246
	S	-2984.769096	-2984.206757	-2984.273480	-2984.836142	-2984.293236
**TS2B'$_{exo-p}$**	Q	-2984.742921	-2984.183886	-2984.250643	-2984.807838	-2984.270512

***meta*-product**						
**TS1B$_{endo-m}$**	Q	-3330.342265	-3329.671669	-3329.751714	-3330.411634	-3329.775738
**IIB'$_{endo-m}$**	D	-3330.363103	-3329.692007	-3329.771653	-3330.430782	-3329.795814
	Q	-3330.374144	-3329.704962	-3329.787203	-3330.442063	-3329.812019
	S	-3330.362787	-3329.695322	-3329.778212	-3330.430464	-3329.803193
**TS1B'$_{endo-m}$**	D	-3330.335881	-3329.663586	-3329.740486	-3330.404037	-3329.763769
	Q	-3330.343878	-3329.673301	-3329.751941	-3330.412452	-3329.775695
	S	-3330.335591	-3329.666763	-3329.746030	-3330.404424	-3329.769962
**IIIB'$_{endo-m}$**	D	-3330.383040	-3329.704968	-3329.778604	-3330.452159	-3329.801089
	Q	-3330.396422	-3329.720691	-3329.799108	-3330.465860	-3329.822686
	S	-3330.384180	-3329.710208	-3329.788713	-3330.453524	-3329.812356
**IIB$_{exo-m}$**	D	-3330.360278	-3329.689646	-3329.769888	-3330.428364	-3329.794205
	Q	-3330.370660	-3329.701570	-3329.785532	-3330.438490	-3329.810685
	S	-3330.358290	-3329.690865	-3329.774981	-3330.426036	-3329.800202
**TS1B$_{exo-m}$**	D	-3330.336488	-3329.663774	-3329.738036	-3330.404238	-3329.760783
	Q	-3330.345505	-3329.674721	-3329.752677	-3330.413116	-3329.776283
	S	-3330.337274	-3329.668168	-3329.746989	-3330.405677	-3329.770813
**IIIB$_{exo-m}$**	D	-3330.385248	-3329.707312	-3329.780123	-3330.453269	-3329.802450
	Q	-3330.400432	-3329.724699	-3329.801631	-3330.468587	-3329.824919
	S	-3330.387081	-3329.712952	-3329.790192	-3330.454726	-3329.813585
**TS1B'$_{exo-m}$**	Q	-3330.339242	-3329.668686	-3329.747293	-3330.408151	-3329.771031
**VB$_{endo-m}$**	D	-2984.737250	-2984.179130	-2984.247028	-2984.802201	-2984.267387
	Q	-2984.759636	-2984.201830	-2984.272102	-2984.824828	-2984.292956
	S	-2984.747928	-2984.191926	-2984.262740	-2984.813515	-2984.283757
**TS2B$_{endo-m}$**	D	-2984.710438	-2984.151408	-2984.218267	-2984.776952	-2984.238113

	Q	-2984.731256	-2984.172161	-2984.238552	-2984.796555	-2984.258291
	S	-2984.725213	-2984.167588	-2984.234354	-2984.791637	-2984.254234
**VIB$_{endo\text{-}m}$**	D	-2984.755834	-2984.191372	-2984.256274	-2984.822130	-2984.275582
	Q	-2984.778579	-2984.214324	-2984.281070	-2984.845733	-2984.300755
	S	-2984.766957	-2984.204665	-2984.272449	-2984.834199	-2984.292407
**TS2B'$_{endo\text{-}m}$**	Q	-2984.730622	-2984.171881	-2984.239503	-2984.796922	-2984.259521
**VB$_{exo\text{-}m}$**	D	-2984.734007	-2984.176486	-2984.246275	-2984.799194	-2984.267053
	Q	-2984.754295	-2984.196694	-2984.268833	-2984.819586	-2984.290052
	S	-2984.743512	-2984.187350	-2984.258306	-2984.808584	-2984.279334
**TS2B$_{exo\text{-}m}$**	D	-2984.715074	-2984.155720	-2984.220475	-2984.780642	-2984.239897
	Q	-2984.734436	-2984.175444	-2984.241703	-2984.799782	-2984.261437
	S	-2984.727878	-2984.170399	-2984.237509	-2984.793541	-2984.257467
**VIB$_{exo\text{-}m}$**	D	-2984.760702	-2984.198939	-2984.263107	-2984.825115	-2984.282363
	Q	-2984.783981	-2984.219597	-2984.284197	-2984.849402	-2984.303462
	S	-2984.772345	-2984.209733	-2984.275067	-2984.837896	-2984.294537
**TS2B'$_{exo\text{-}m}$**	Q	-2984.728324	-2984.169559	-2984.236762	-2984.794502	-2984.256691

Supplementary Table 40. The absolute energies (in Hartree) of the optimized structures to form **C** by the B3LYP-D3 method. (Q: quartet)

**C**		**E**	**E+ZPE**	**G**	**E$_{soln}$**	**G(80°C)**
**C_5H_8**		-195.317180	-195.202860	-195.231240	-195.321515	-195.237894
**IIC$_{endo\text{-}p}$**	Q	-3138.618484	-3138.002964	-3138.082967	-3138.682868	-3138.106749
**TS1C$_{endo\text{-}p}$**	Q	-3138.600405	-3137.983629	-3138.059745	-3138.665126	-3138.082427
**IIIC$_{endo\text{-}p}$**	Q	-3138.648082	-3138.025883	-3138.101000	-3138.713118	-3138.123355
**TS2C$_{endo\text{-}p}$**	Q	-2792.989269	-2792.484085	-2792.549040	-2793.052320	-2792.567950
**TS1C$_{exo\text{-}p}$**	Q	-3138.601373	-3137.984227	-3138.058696	-3138.665927	-3138.081038

Supplementary Table 41. The absolute energies (in Hartree) of the optimized structures to form **D** by the B3LYP-D3 method. (Q: quartet; S: sextet)

**D**		**E**	**E+ZPE**	**G**	**E_{soln}**	**G(80°C)**
**$C_{11}H_{12}$**		-426.382528	-426.186714	-426.222603	-426.393467	-426.231620
**$IID_{endo\text{-}cis\text{-}p}$**	Q	-3369.697684	-3369.000059	-3369.085223	-3369.767322	-3369.110891
**$TS1D_{endo\text{-}cis\text{-}p}$**	Q	-3369.681242	-3368.982586	-3369.064438	-3369.749698	-3369.089163
	S	-3369.674415	-3368.977484	-3369.061055	-3369.743152	-3369.086161
**$IIID_{endo\text{-}cis\text{-}p}$**	Q	-3369.713139	-3369.009612	-3369.090427	-3369.783444	-3369.114800
**$VD_{endo\text{-}cis\text{-}p}$**	Q	-3024.082761	-3023.496454	-3023.569151	-3024.149082	-3023.590751
**$TS2D_{endo\text{-}cis\text{-}p}$**	Q	-3024.070786	-3023.483601	-3023.552922	-3024.136721	-3023.573595
	S	-3024.065287	-3023.479648	-3023.549826	-3024.130868	-3023.570722
**$VID_{endo\text{-}cis\text{-}p}$**	Q	-3024.097548	-3023.505538	-3023.573205	-3024.165047	-3023.593397
**$TS1D_{endo\text{-}trans\text{-}p}$**	Q	-3369.669302	-3368.970416	-3369.052177	-3369.737775	-3369.076856
**$TS1D_{exo\text{-}cis\text{-}p}$**	Q	-3369.673291	-3368.974296	-3369.054768	-3369.741055	-3369.079216
**$IID_{exo\text{-}trans\text{-}p}$**	Q	-3369.697060	-3368.999666	-3369.084783	-3369.766508	-3369.110475
**$TS1D_{exo\text{-}trans\text{-}p}$**	Q	-3369.679722	-3368.981189	-3369.063207	-3369.748399	-3369.087982
	S	-3369.672967	-3368.976168	-3369.058430	-3369.741732	-3369.083307
**$IIID_{exo\text{-}trans\text{-}p}$**	Q	-3369.715557	-3369.011265	-3369.089942	-3369.784595	-3369.113862
**$VD_{exo\text{-}trans\text{-}p}$**	Q	-3024.077332	-3023.491296	-3023.564442	-3024.144370	-3023.586154
**$TS2D_{exo\text{-}trans\text{-}p}$**	Q	-3024.070726	-3023.483669	-3023.552321	-3024.136065	-3023.572880
	S	-3024.065627	-3023.480431	-3023.550137	-3024.131145	-3023.570975
**$VID_{exo\text{-}trans\text{-}p}$**	Q	-3024.099782	-3023.507052	-3023.573077	-3024.166195	-3023.592908
**Stepwise-TS**						
**$TS2D_{cc\text{-}endo\text{-}cis\text{-}p}$**	Q	-3024.067634	-3023.480549	-3023.550376	-3024.134157	-3023.573651
	S	-3024.063326	-3023.477877	-3023.547966	-3024.129754	-3023.571291
**$TS2D_{co\text{-}endo\text{-}cis\text{-}p}$**	Q	-3024.075258	-3023.486106	-3023.553340	-3024.140881	-3023.573457
	S	-3024.070059	-3023.482497	-3023.551009	-3024.136677	-3023.571467
**$TS2D_{cc\text{-}exo\text{-}trans\text{-}p}$**	Q	-3024.069073	-3023.482197	-3023.551233	-3024.134376	-3023.571874
	S	-3024.064401	-3023.479250	-3023.549600	-3024.129924	-3023.570570
**$TS2D_{co\text{-}exo\text{-}trans\text{-}p}$**	Q	-3024.075613	-3023.486033	-3023.551885	-3024.141462	-3023.571731
	S	-3024.072590	-3023.484129	-3023.550702	-3024.138998	-3023.570741

Supplementary Table 42. The absolute energies (in Hartree) of the optimized structures to form **E1O** and **E1C** by the B3LYP-D3 method. (Q: quartet; S: sextet)

E1		E	E+ZPE	G	E_{soln}	G(80°C)
**C_5H_8O**		-270.558638	-270.440208	-270.471117	-270.566830	-270.478529
**IE1**	Q	-2793.251495	-2792.734786	-2792.806612	-2793.311976	-2792.827593
	S	-2793.240494	-2792.725401	-2792.796688	-2793.300971	-2792.817598
***para*-product**						
**IIE1O$_{endo-cis-p}$**	Q	-3219.660426	-3218.945902	-3219.028688	-3219.725710	-3219.054013
**TS1E1O$_{endo-cis-p}$**	Q	-3219.636129	-3218.920465	-3219.003192	-3219.701137	-3219.028347
	S	-3219.631151	-3218.917090	-3218.999897	-3219.696113	-3219.025134
**IIIE1O$_{endo-cis-p}$**	Q	-3219.678290	-3218.957125	-3219.037454	-3219.744393	-3219.061981
**TS2E1O$_{endo-cis-p}$**	Q	-2949.046893	-2948.451076	-2948.520712	-2949.110708	-2948.541573
	S	-2949.042874	-2948.448669	-2948.518674	-2949.106648	-2948.539662
**TS1E1O$_{endo-trans-p}$**	S	-3219.624073	-3218.909992	-3218.991885	-3219.688213	-3219.016925
**TS1E1O$_{exo-cis-p}$**	Q	-3219.629952	-3218.913887	-3218.995160	-3219.693973	-3219.020026
	S	-3219.624958	-3218.910795	-3218.993385	-3219.689338	-3219.018579
**TS1E1O$_{exo-trans-p}$**	Q	-3219.638341	-3218.922821	-3219.003576	-3219.701794	-3219.028360
	S	-3219.632685	-3218.918721	-3218.999431	-3219.696201	-3219.024256
**TS1E1C$_{endo-cis-p}$**	Q	-3219.633395	-3218.916739	-3218.996835	-3219.698359	-3219.021386
	S	-3219.625829	-3218.910898	-3218.992741	-3219.690667	-3219.017672
**TS1E1C$_{endo-trans-p}$**	Q	-3219.619122	-3218.902900	-3218.985224	-3219.685094	-3219.010241
**TS1E1C$_{exo-cis-p}$**	Q	-3219.618288	-3218.901929	-3218.983951	-3219.684608	-3219.008903
	S	-3219.611285	-3218.896648	-3218.979284	-3219.677700	-3219.004407
**IIE1C$_{exo-trans-p}$**	Q	-3219.660514	-3218.945854	-3219.032113	-3219.725971	-3219.058255
**TS1E1C$_{exo-trans-p}$**	Q	-3219.632653	-3218.916457	-3218.999024	-3219.698409	-3219.024073
	S	-3219.625052	-3218.910500	-3218.992725	-3219.690827	-3219.017765
**IIIE1C$_{exo-trans-p}$**	Q	-3219.685181	-3218.964251	-3219.045433	-3219.753822	-3219.070069
**VIIE1C$_{exo-trans-p}$**	S	-3219.618341	-3218.902680	-3218.984571	-3219.686248	-3219.009609
**TS2E1C$_{exo-trans-p}$**	Q	-2949.041713	-2948.445380	-2948.514520	-2949.106174	-2948.535210
	S	-2949.035734	-2948.441052	-2948.510232	-2949.099972	-2948.530998
***meta*-product(reaction with C=C)**						
**TS1E1C$_{endo-cis-m}$**	Q	-3219.620383	-3218.903863	-3218.985024	-3219.686701	-3219.009794
**TS1E1C$_{endo-trans-m}$**	Q	-3219.614251	-3218.897430	-3218.977579	-3219.680126	-3219.002128
**TS1E1C$_{exo-cis-m}$**	Q	-3219.609922	-3218.893153	-3218.975182	-3219.676609	-3219.000084
**TS1E1C$_{exo-trans-m}$**	Q	-3219.616411	-3218.900244	-3218.981787	-3219.683252	-3219.006633
**TS2E1C$_{endo-cis-m}$**	Q	-2949.027676	-2948.431114	-2948.501278	-2949.093183	-2948.522120

**TS2E1C$_{endo-trans-m}$**	Q	-2949.022658	-2948.425514	-2948.493101	-2949.087862	-2948.513429
**TS2E1C$_{exo-cis-m}$**	Q	-2949.017130	-2948.420294	-2948.488679	-2949.082939	-2948.509169
**TS2E1C$_{exo-trans-m}$**	Q	-2949.023404	-2948.427060	-2948.496052	-2949.089287	-2948.516685

Supplementary Table 43. The absolute energies (in Hartree) of the optimized structures to form **E2O** and **E2C** by the B3LYP-D3 method. (Q: quartet; S: sextet)

E2		E	E+ZPE	G	E$_{soln}$	G(80°C)
**C_3H_4O**		-191.914757	-191.853097	-191.879369	-191.920068	-191.885316
**IE2**	Q	-2635.950647	-2635.547086	-2635.609997	-2636.008104	-2635.628052
	S	-2635.937886	-2635.536143	-2635.599677	-2635.995721	-2635.617896
***para*-product**						
**TS1E2O$_{endo-cis-p}$**	Q	-3062.337975	-3061.735751	-3061.810548	-3062.400055	-3061.833003
	S	-3062.331515	-3061.730974	-3061.806653	-3062.393686	-3061.829330
**IIIE2O$_{endo-cis-p}$**	Q	-3062.382594	-3061.774893	-3061.848195	-3062.445763	-3061.870169
**TS2E2O$_{endo-cis-p}$**	Q	-2870.397239	-2869.858163	-2869.924907	-2870.460224	-2869.944616
	S	-2870.392046	-2869.854665	-2869.921628	-2870.454926	-2869.941443
**TS1E2O$_{exo-trans-p}$**	Q	-3062.340960	-3061.738626	-3061.811431	-3062.402024	-3061.833465
	S	-3062.334371	-3061.733692	-3061.806700	-3062.395271	-3061.828833
**IIIE2O$_{exo-trans-p}$**	Q	-3062.377105	-3061.769328	-3061.840359	-3062.438426	-3061.861856
**TS1E2C$_{endo-cis-p}$**	Q	-3062.344191	-3061.741462	-3061.815582	-3062.407727	-3061.838978
	S	-3062.336189	-3061.734930	-3061.809307	-3062.399522	-3061.832999
**IIIE2C$_{endo-cis-p}$**	Q	-3062.397922	-3061.790320	-3061.865115	-3062.464146	-3061.887370
**TS2E2C$_{endo-cis-p}$**	Q	-2870.402324	-2869.862925	-2869.926390	-2870.466062	-2869.945263
	S	-2870.395985	-2869.858263	-2869.924960	-2870.459997	-2869.944683
**TS1E2C$_{exo-trans-p}$**	Q	-3062.343313	-3061.740808	-3061.8162631	-3062.406903	-3061.838803
	S	-3062.334005	-3061.733351	-3061.810208	-3062.397548	-3061.833080
**IIIE2C$_{exo-trans-p}$**	Q	-3062.397002	-3061.789324	-3061.864201	-3062.463876	-3061.886467
**Stepwise-TS**						
**TS2E2O$_{cc-endo-cis-p}$**	Q	-2870.395404	-2869.856531	-2869.923153	-2870.458543	-2869.942852
	S	-2870.391299	-2869.854094	-2869.921171	-2870.454368	-2869.941025
**TS2E2O$_{co-endo-cis-p}$**	Q	-2870.401986	-2869.860806	-2869.924484	-2870.464471	-2869.943404
	S	-2870.398280	-2869.858350	-2869.923569	-2870.461180	-2869.942846
**TS2E2C$_{cc1-endo-cis-p}$**	Q	-2870.398167	-2869.858466	-2869.924362	-2870.461829	-2869.943846
	S	-2870.393288	-2869.855098	-2869.921231	-2870.456856	-2869.940817
**TS2E2C$_{cc2-endo-cis-p}$**	S	-2870.413481	-2869.873062	-2869.939174	-2870.478728	-2869.958588
***meta*-product(reaction with C=C)**						
**TS1E2C$_{endo-cis-m}$**	Q	-3062.333688	-3061.731456	-3061.803680	-3062.397935	-3061.825433
**TS1E2C$_{endo-trans-m}$**	Q	-3062.326584	-3061.723812	-3061.798722	-3062.390641	-3061.821133
**TS1E2C$_{exo-cis-m}$**	Q	-3062.323898	-3061.721146	-3061.796364	-3062.388701	-3061.818834
**TS1E2C$_{exo-trans-m}$**	Q	-3062.331141	-3061.728829	-3061.804283	-3062.396021	-3061.826817

**TS2E2C$_{endo\text{-}cis\text{-}m}$**	Q	-2870.391025	-2869.851637	-2869.918335	-2870.456344	-2869.937971
**TS2E2C$_{endo\text{-}trans\text{-}m}$**	Q	-2870.384326	-2869.844656	-2869.911039	-2870.449298	-2869.930616
**TS2E2C$_{exo\text{-}cis\text{-}m}$**	Q	-2870.380587	-2869.840913	-2869.907631	-2870.446183	-2869.927275
**TS2E2C$_{exo\text{-}trans\text{-}m}$**	Q	-2870.386981	-2869.847887	-2869.911997	-2870.452833	-2869.930993

Supplementary Table 44. The absolute energies (in Hartree) of the optimized structures to form **F** by the B3LYP-D3 method. (Q: quartet; S: sextet)

**F**		**E**	**E+ZPE**	**G**	**E$_{soln}$**	**G(80°C)**
**$C_6H_{10}O$**		-309.897194	-309.745481	-309.776418	-309.907390	-309.783738
**IF**	Q	-2871.926857	-2871.343712	-2871.412294	-2871.989947	-2871.432448
	S	-2871.912448	-2871.331978	-2871.403021	-2871.975441	-2871.423742
**TS1F$_1$**	Q	-3298.303946	-3297.521400	-3297.599721	-3298.369656	-3297.623816
	S	-3298.298921	-3297.518328	-3297.597861	-3298.364644	-3297.622273
**TS1F$_2$**	Q	-3298.302137	-3297.519508	-3297.597685	-3298.368613	-3297.621750
	S	-3298.298694	-3297.518269	-3297.598336	-3298.364705	-3297.622859
**TS2F$_1$**	Q	-2988.377016	-2987.747783	-2987.814806	-2988.440829	-2987.835053
	S	-2988.375539	-2987.748065	-2987.816592	-2988.439261	-2987.837201
**TS2F$_2$**	Q	-2988.376170	-2987.746839	-2987.813975	-2988.440232	-2987.834246
	S	-2988.375214	-2987.747559	-2987.815653	-2988.439468	-2987.836171

Supplementary Table 45. The absolute energies (in Hartree) for the key intermediates and transition states optimized in solution by the SMD B3LYP-D3 method. (D: doublet; Q: quartet; S: sextet)

		E_{soln}	$(E+ZPE)_{soln}$	G_{soln}	$G_{soln\text{-}80\,^{o}C}$
**A**					
C_6H_{10}		-234.641583	-234.498997	-234.529433	-234.537124
**PhCHO**		-345.591507	-345.481207	-345.511842	-345.520033
**IA**	D	-2943.343757	-2942.841073	-2942.905545	-2942.926854
	Q	-2943.354579	-2942.853856	-2942.922593	-2942.945025
	S	-2943.342673	-2942.844051	-2942.913251	-2942.935364
$TS1A_{endo}$	D	-3177.981439	-3177.334162	-3177.40749	-3177.432150
	Q	-3177.987685	-3177.341614	-3177.417297	-3177.440125
	S	-3177.980679	-3177.336486	-3177.412675	-3177.437872
$TS2A_{endo}$	D	-2832.356082	-2831.821285	-2831.883539	-2831.905361
	Q	-2832.37385	-2831.840099	-2831.904616	-2831.923099
	S	-2832.368099	-2831.836329	-2831.903023	-2831.922615
$IIIA_{endo}$	Q	-3178.035812	-3177.385359	-3177.460233	-3177.48285
VIA_{endo}	Q	-2832.417261	-2831.87856	-2831.942547	-2831.961427
$VIIA_{exo}$	D	-3177.981824	-3177.332506	-3177.40389	-3177.425746
	Q	-3177.97984	-3177.3352	-3177.4106	-3177.4334
	S	-3177.98109	-3177.336682	-3177.410598	-3177.433086
$VIIIA_{exo}$	D	-2832.360896	-2831.824194	-2831.885251	-2831.903599
	Q	-2832.370235	-2831.836226	-2831.899878	-2831.918768
	S	-2832.371636	-2831.837098	-2831.901055	-2831.920046
$VIIA_{endo}$	Q	-3177.97427	-3177.329985	-3177.405669	-3177.428414
	S	-3177.971868	-3177.328994	-3177.404187	-3177.426869
$VIIIA_{endo}$	Q	-2832.364763	-2831.829512	-2831.895017	-2831.91346
	S	-2832.363698	-2831.831546	-2831.894263	-2831.912868
**Catalyzed by Ru(III)**					
IA_{Ru}	D	-1542.282148	-1541.885797	-1541.943717	-1541.960428
	Q	-1542.212659	-1541.819918	-1541.878786	-1541.895791
$TS1A_{endo,Ru}$	D	-1776.91134	-1776.369964	-1776.436917	-1776.456888
	Q	-1776.848571	-1776.311584	-1776.379005	-1776.399187
$TS1A_{exo,Ru}$	D	-1776.912069	-1776.369973	-1776.433894	-1776.453236
B					
$C_{10}H_{10}$		-387.069894	-386.902256	-386.935891	-386.944156
$TS1B_{endo\text{-}p}$	Q	-3330.42002	-3329.749098	-3329.827755	-3329.851541
$TS2B_{endo\text{-}p}$	Q	-2984.806732	-2984.247448	-2984.314834	-2984.334816
	S	-2984.801378	-2984.243385	-2984.311559	-2984.331727
$TS2B'_{endo\text{-}p}$	Q	-2984.80443	-2984.245872	-2984.311087	-2984.330516
$TS1B_{endo\text{-}m}$	Q	-3330.4119	-3329.741284	-3329.821411	-3329.845454
$TS2B_{exo\text{-}m}$	Q	-2984.799971	-2984.241073	-2984.308427	-2984.328385
C					

**C_5H_8**		-195.321521	-195.20731	-195.235678	-195.242331
**TS1C$_{endo\text{-}p}$**	Q	-3138.665653	-3138.048317	-3138.121527	-3138.143613
**TS2C$_{endo\text{-}p}$**	Q	-2793.052114	-2792.546763	-2792.609151	-2792.627543
**TS2C$_{exo\text{-}m}$**	Q	-2793.046155	-2792.540414	-2792.602316	-2792.620605
D					
**$C_{11}H_{12}$**		-426.393482	-426.19772	-426.23368	-426.242715
**TS1D$_{endo\text{-}cis\text{-}p}$**	Q	-3369.750144	-3369.051853	-3369.134595	-3369.1595
**TS2D$_{endo\text{-}cis\text{-}p}$**	Q	-3024.137077	-3023.549681	-3023.618217	-3023.638725
	S	-3024.131156	-3023.545961	-3023.612900	-3023.633025
**TS1D$_{exo\text{-}trans\text{-}p}$**	Q	-3369.748794	-3369.049689	-3369.129535	-3369.153864
**TS2D$_{exo\text{-}trans\text{-}p}$**	Q	-3024.136361	-3023.548612	-3023.615659	-3023.635868
	S	-3024.13147	-3023.545794	-3023.614455	-3023.635060
Stepwise					
**TS2D$_{cc\text{-}endo\text{-}cis\text{-}p}$**	Q	-3024.134222	-3023.546945	-3023.615518	-3023.636050
	S	-3024.129895	-3023.544295	-3023.614326	-3023.633735
**TS2D$_{co\text{-}endo\text{-}cis\text{-}p}$**	Q	-3024.141221	-3023.551641	-3023.617456	-3023.637243
**TS2D$_{cc\text{-}exo\text{-}trans\text{-}p}$**	Q	-3024.13501	-3023.547785	-3023.615684	-3023.636080
	S	-3024.130448	-3023.545314	-3023.614871	-3023.635688
**E1**					
**C_5H_8O**		-270.566864	-270.448398	-270.479434	-270.48687
**IE1**	Q	-2793.31214	-2792.795459	-2792.866102	-2792.886853
**TS1E1O$_{endo\text{-}cis\text{-}p}$**	Q	-3219.701736	-3218.985578	-3219.065135	-3219.089645
**TS2E1O$_{endo\text{-}cis\text{-}p}$**	Q	-2949.110925	-2948.514811	-2948.583109	-2948.603689
**TS1E1C$_{exo\text{-}trans\text{-}p}$**	Q	-3219.69882	-3218.981776	-3219.059848	-3219.08397
**TS1E1C$_{endo\text{-}trans\text{-}p}$**	S	-3219.679273	-3218.963976	-3219.043043	-3219.067411
**TS2E1C$_{exo\text{-}trans\text{-}p}$**	Q	-2949.106822	-2948.509356	-2948.575863	-2948.595986
**VIIE1O$_{endo\text{-}cis\text{-}p}$**	Q	-3219.696323	-3218.980152	-3219.061968	-3219.087029
	S	-3219.694552	-3218.979699	-3219.062194	-3219.087427
**VIIE1C$_{exo\text{-}trans\text{-}p}$**	Q	-3219.689712	-3218.97251	-3219.053222	-3219.077986
	S	-3219.676291	-3218.960584	-3219.042407	-3219.067442
**E2**					
**C_3H_4O**		-191.920089	-191.85833	-191.884567	-191.890502
**IE2**	Q	-2636.008174	-2635.604665	-2635.666552	-2635.684401
**TS1E2O$_{endo\text{-}cis\text{-}p}$**	Q	-3062.400404	-3061.798164	-3061.869295	-3061.890866
**TS2E2O$_{endo\text{-}cis\text{-}p}$**	Q	-2870.460382	-2869.921456	-2869.987816	-2870.007452
**TS1E2C$_{endo\text{-}cis\text{-}p}$**	Q	-3062.40793	-3061.804264	-3061.876379	-3061.898211
**TS1E2C$_{exo\text{-}trans\text{-}p}$**	Q	-3062.407483	-3061.804148	-3061.87627	-3061.898108
**TS2E2C$_{endo\text{-}cis\text{-}p}$**	Q	-2870.466414	-2869.926676	-2869.991546	-2870.010831
Stepwise					
**TS2E2O$_{cc\text{-}endo\text{-}cis\text{-}p}$**	Q	-2870.45885	-2869.919876	-2869.985649	-2870.00517
**TS2E2O$_{co\text{-}endo\text{-}cis\text{-}p}$**	Q	-2870.464772	-2869.92348	-2869.986983	-2870.00587
**TS2E2C$_{cc1\text{-}endo\text{-}cis\text{-}p}$**	Q	-2870.462188	-2869.92239	-2869.984686	-2870.003307

Supplementary Table 46. The absolute gibbs free energies (in Hartree) for the key intermediates and transition states for **A** optimized in solution by other methods. (Q: quartet; S: sextet)

		PBE0-D3 $G_{soln\text{-}80^{o}C}$	B3PW91-D3 $G_{soln\text{-}80^{o}C}$	PW6B95-D3 $G_{soln\text{-}80^{o}C}$	BP86-D3 $G_{soln\text{-}80^{o}C}$	M06-L $G_{soln\text{-}80^{o}C}$
C_6H_{10}		-234.232792	-234.448219	-234.828150	-234.527064	-234.482404
PhCHO		-345.111890	-345.386156	-345.954621	-345.518676	-345.465606
IA	Q	-2940.726386	-2942.259719	-2945.779668	-2943.150245	-2942.616059
	S	-2940.721805	-2942.247387	-2945.771324	-2943.147610	-2942.623161
$TS1A_{endo}$	Q	-3174.922689	-3176.669262	-3180.564935	-3177.650904	-3177.053013
	S	-3174.925042	-3176.669658	-3180.563759	-3177.631901	-3177.064074
$TS2A_{endo}$	Q	-2829.814075	-2831.288090	-2834.613923	-2832.136865	-2831.593000
	S	-2829.817948	-2831.289425	-2834.608298	-2832.116028	-2831.603963

		B3LYP*-D3 $G_{soln\text{-}80^{o}C}$	OLYP-D3 $G_{soln\text{-}80^{o}C}$	TPSSh-D3 $G_{soln\text{-}80^{o}C}$	OPBE-D3 $G_{soln\text{-}80^{o}C}$	ωB97XD $G_{soln\text{-}80^{o}C}$
C_6H_{10}		-234.398689	-234.448725	-234.551132	-234.451620	-234.445809
PhCHO		-345.391724	-345.425174	-345.538846	-345.389235	-345.390480
IA	Q	-2942.403293	-2942.781961	-2943.064564	-2942.589705	-2942.268036
	S	-2942.378807	-2942.772401	-2943.050358	-2942.582675	-2942.257806
$TS1A_{endo}$	Q	-3176.769394	-3177.197311	-3177.578862	-3177.014521	-3176.675910
	S	-3176.752751	-3177.195690	-3177.568863	-3177.010569	-3176.671638
$TS2A_{endo}$	Q	-2831.378476	-2831.776206	-2832.044873	-2831.629305	-2831.286426
	S	-2831.360742	-2831.772080	-2832.031996	-2831.624925	-2831.288469

Supplementary Table 47. The absolute single-point energies (in Hartree) in gas phase by DLPNO-CCSD(T) and B2PLYP methods based on the SMD B3LYP-D3-optimized structures to form **A** in solution. (Q: quartet; S: sextet)

		DLPNO-CCSD(T)	B2PLYP-D3
C_6H_{10}		-234.083718	-234.516789
PhCHO		-344.837364	-345.464362
IA	Q	-2938.723110	-2942.472010
	S	-2938.738756	-2942.464730
$TS1A_{endo}$	Q	-3172.797990	-3176.979100
	S	-3172.819840	-3176.976830
$TS2A_{endo}$	Q	-2827.929240	-2831.487350
	S	-2827.948330	-2831.484150

Supplementary Table 48. The absolute and relative Gibbs free energies (in Hartree) for the key intermediates and transition states with the neutral Fe(III) complexes (see **Supplementary Figure 11**) optimized in solution by the SMD B3LYP-D3 method. (D: doublet; Q: quartet; S: sextet).

		E_{soln}	$(E+ZPE)_{soln}$	G_{soln}	$G_{soln\text{-}80\,^{o}C}$	$\triangle G_{soln\text{-}80\,^{o}C}$
**Fe(III)Cl complex**						
**IVA(Cl$^-$)**	D	-2712.507668	-2712.22979	-2712.276031	-2712.291746	15.4
	Q	-2712.527632	-2712.250088	-2712.297404	-2712.314112	1.3
	S	-2712.527784	-2712.251840	-2712.300162	-2712.316221	0.0
**IA(Cl$^-$)**	D	-3058.122934	-3057.731720	-3057.789253	-3057.805813	19.1
	Q	-3058.133045	-3057.744082	-3057.803900	-3057.821150	9.5
	S	-3058.130714	-3057.743154	-3057.803329	-3057.820553	9.9
**TS1A$_{endo}$ (Cl$^-$)**	Q	-3292.756029	-3292.222505	-3292.290300	-3292.315960	33.8
	S	-3292.753262	-3292.221261	-3292.288500	-3292.312460	36.0
**Fe(III)OTf complex**						
**IVA (OTf$^-$)**	D	-3213.725261	-3213.420210	-3213.476301	-3213.493332	15.4
	Q	-3213.747663	-3213.442821	-3213.500565	-3213.517873	0.0
	S	-3213.741920	-3213.438970	-3213.497970	-3213.515619	1.4
**IA(OTf$^-$)**	D	-3559.343687	-3558.924665	-3558.991300	-3559.014920	14.4
	Q	-3559.357785	-3558.941575	-3559.011100	-3559.031730	3.9
	S	-3559.34854	-3558.934337	-3559.003000	-3559.031940	3.7
**TS1A$_{endo}$ (OTf$^-$)**	Q	-3793.984849	-3793.422845	-3793.500200	-3793.523840	29.9
	S	-3793.977665	-3793.417160	-3793.495200	-3793.519010	33.0

Supplementary Table 49. The absolute Gibbs free energies (in Hartree) for the key intermediates and transition states used acetone instead of PhCHO with diene to form **G** optimized in solution by the SMD B3LYP-D3 method. (D: doublet; Q: quartet; S: sextet)

		E_{soln}	$(E+ZPE)_{soln}$	G_{soln}
**IG**	D	-2638.506766	-2638.057053	-2638.115820
	Q	-2638.522448	-2638.074656	-2638.138018
	S	-2638.510061	-2638.064240	-2638.127856
**IG$_{4Ph}$**	D	-3562.775876	-3562.002013	-3562.090231
	Q	-3562.793890	-3562.022279	-3562.111553
	S	-3562.781641	-3562.012533	-3562.102293

Supplementary Table 50. The absolute single-point energies (in Hartree) for the key intermediates and transition states used acetone instead of PhCHO with diene to form **G** in solution by other DFT methods and SMD method. (D: doublet; Q: quartet; S: sextet)

		**LCCSD(T)**	**PBE0-D3**	**B3PW91-D3**	**OLYP-D3**
**IG**	D	-	-2636.642659	-2637.944737	-2638.393915
	Q	-2634.572862	-2636.661752	-2637.961101	-2638.414207
	S	-2634.589484	-2636.657388	-2637.951114	-2638.401401
**IG_{4Ph}**	D	-	-3559.811441	-3561.870091	-3562.403091
	Q	-	-3559.835342	-3561.890259	-3562.421925
	S	-	-3559.831031	-3561.880170	-3562.408855
		**PW6B95-D3**	**M06-L**	**OPBE-D3**	**ωB97XD**
**IG**	D	-2640.925028	-2638.224145	-2638.247592	-2637.966376
	Q	-2640.942059	-2638.238216	-2638.266541	-2637.978406
	S	-2640.932797	-2638.242084	-2638.255535	-2637.967608
**IG_{4Ph}**	D	-3566.425894	-3562.320507	-3562.204901	-3561.885443
	Q	-3566.448534	-3562.342804	-3562.225392	-3561.905691
	S	-3566.439073	-3562.346798	-3562.213921	-3561.894157

Supplementary Table 51. The absolute energies (in Hartree) of the reaction Energy of ODA by some methods and SMD method in solution.

	**E_{soln}**	**$(E+ZPE)_{soln}$**	**G_{soln}**	**$G_{soln\text{-}80^{o}C}$**
		**B3LYP-D3**		
**C_6H_{10}**	-234.641583	-234.498997	-234.529433	-234.537124
**PhCHO**	-345.591507	-345.481207	-345.511842	-345.520033
**Product-A**	-580.259732	-580.000336	-580.040399	-580.052801
		**B3PW91-D3**		
**C_6H_{10}**	-234.553113	-234.410445	-234.440891	-234.448219
**PhCHO**	-345.457803	-345.347281	-345.377947	-345.386156
**Product-A**	-580.039551	-579.779773	-579.819947	-579.830373
		**PBE0-D3**		
**C_6H_{10}**	-234.338278	-234.195031	-234.225466	-234.232792
**PhCHO**	-345.184132	-345.073031	-345.103682	-345.111890
**Product-A**	-579.570408	-579.309263	-579.349171	-579.359526
		**M06-D3**		
**C_6H_{10}**	-234.431510	-234.290092	-234.320617	-234.327979
**PhCHO**	-345.337550	-345.227680	-345.258262	-345.265455
**Product-A**	-579.808987	-579.550843	-579.591220	-579.601688
		**M06-2X-D3**		
**C_6H_{10}**	-234.504378	-234.361221	-234.392133	-234.399584
**PhCHO**	-345.430369	-345.319061	-345.349729	-345.356932
**Product-A**	-579.973947	-579.712580	-579.752866	-579.763312

Supplementary Table 52. The absolute energies (in Hartree) for the key complexes and transition states optimized in solution and in the presence of an OEEF field by the SMD B3LYP-D3 method. (D: doublet; Q: quartet; S: sextet)

		E_{soln}	$(E+ZPE)_{soln}$	G_{soln}	$G_{soln\text{-}80\,^{o}C}$
**A**					
**OEEF (*Fz* = -0.0015 AU) along the reacting Fe-O bond**					
**IIA**	Q	-3178.010108	-3177.365194	-3177.443087	-3177.466663
**TS1A$_{endo}$**	Q	-3177.990484	-3177.345315	-3177.421533	-3177.444518
**IIIA$_{endo}$**	Q	-3178.03525	-3177.384414	-3177.457928	-3177.480257
**VA**	Q	-2832.39766	-2831.86469	-2831.93145	-2831.951263
**TS2A$_{endo}$**	Q	-2832.37974	-2831.84609	-2831.9105	-2831.929576
	S	-2832.37585	-2831.84348	-2831.90831	-2831.927506
**VIA$_{endo}$**	Q	-2832.42072	-2831.88195	-2831.94501	-2831.963702
	S	-2832.40969	-2831.87277	-2831.93688	-2831.955859
**OEEF (*Fz* = -0.003 AU) along the reacting Fe-O bond**					
**IIA**	Q	-3178.01190	-3177.36703	-3177.44469	-3177.46822
**TS1A$_{endo}$**	Q	-3177.99473	-3177.34961	-3177.42600	-3177.44902
**IIIA**	Q	-3178.03605	-3177.38532	-3177.45873	-3177.48104
**VA**	Q	-2832.40433	-2831.87143	-2831.93811	-2831.95790
**TS2A$_{endo}$**	Q	-2832.38715	-2831.85394	-2831.91923	-2831.93849
	S	-2832.38363	-2831.85136	-2831.91641	-2831.93565
**VIA$_{endo}$**	Q	-2832.42516	-2831.88640	-2831.94928	-2831.96793
	S	-2832.41500	-2831.87818	-2831.94217	-2831.96116

Supplementary Table 53. The absolute energies (in Hartree) for the key intermediates and transition states for the formation of **A** with the replacement of the 5-methyl-1H-imidazole(his) ligand with PhCHO optimized in diethylether solution by the SMD B3LYP-D3 method. (D: doublet; Q: quartet; S: sextet)

		E_{soln}	$(E+ZPE)_{soln}$	G_{soln}
**C_6H_{10}**		-234.641635	-234.499248	-234.529757
**IA$_{his}$**	D	-2863.339981	-2862.84821	-2862.91051
	Q	-2863.339258	-2862.84969	-2862.91683
	S	-2863.329882	-2862.84198	-2862.90912
**TS1A$_{endo,his}$**	D	-3097.973359	-3097.33692	-3097.40712
	Q	-3097.968834	-3097.33406	-3097.40847
	S	-3097.963921	-3097.33016	-3097.4035
**IIIA$_{endo,his}$**	D	-3098.016912	-3097.37489	-3097.44345
	Q	-3098.021127	-3097.38175	-3097.45382
	S	-3098.01124	-3097.37331	-3097.44586

Supplementary Methods

A cationic Fe(III)-porphine was used as our model catalyst. All calculations were carried out with Gaussian 09 (except DLPNO-CCSD(T)/def2-TZVP(-f) with RIJCOSX and RI-B2PLYP-D3/def2-TZVP methods using ORCA 4.01).[1-3] The B3LYP-D3[4-6] method combined with the 6-31G* basis set was used to optimize all the structures in the gas phase for all atoms, except def2-TZVP[7-8] (augmented with the corresponding effective core potential for the Ru atom) basis set applied for Fe and Ru atoms. Then, vibration frequency calculations were carried out at the same level of theory. Intrinsic reaction coordinate (IRC) calculations[9] were also carried out for the key reaction step to connect the reactant and product adducts, as well as obtain the minimum energy path (MEP). The effect of the solvent (benzene) was then included by single-point calculations with an implicit solvent model (SMD method, denoted as the SMD B3LYP-D3//B3LYP-D3 method).[10] The geometries of the key structures were also further optimized in solution and verified by vibration frequency calculations with the SMD B3LYP-D3 method and the same basis sets. All mechanistic conclusions can be supported by these two computational approaches. The effect of the OEEF on the reactivity in solution was also considered by optimizing structures with the SMD B3LYP-D3 method and adding an OEEF along the reacting Fe-O bond as the OEEF positive z-axis (***Fz***, with a strength of -0.0015 or -0.0030 au).[11-16] Furthermore, the effect of a biological histidine ligand as the axial ligand on the reaction was aslo examined by using a 5-methyl-1H-imidazole ligand to replace PhCHO for the 6-coordinate pathway optimized by SMD B3LYP-D3 method in diethylether solution. Diethylether (its dielectric constant of ~4.2) was used to mimics hydrophobic enzymatic environment. Moreover, to examine the effect of the DFT functional, M06-D3, M06-2X-D3, B3PW91-D3, PBE0-D3 and ωB97XD methods[17-19] were used for the single-point energy or geometry optimization calculations in the key reaction steps. We have also performed additional energy calculations of the key intermediates and transition states to form **A** by the DLPNO-CCSD(T), B2PLYP-D3 PW6B95-D3, OLYP-D3, TPSSh-D3 and OPBE-D3 methods.[20-25] In addition, a Fe(III) porphyrin complex coordinating with two acetone as axial ligands which was recently experimentally found to have admixed spin states was used to test different DFT and

DLPNO-CCSD(T) methods in our additional calculations.

A standard-state concentration (RTln(24.5), or 2.2 kcal mol^{-1}) was applied to correct the relative free energy in solution in the text, when the number of molecules is changed.[26] All energies presented are relative Gibbs free energies in solution (at 353.15 K in kcal mol^{-1}) above the most stable Fe(III)-porphine complex coordinated to two carbonyl molecules[27] by the SMD B3LYP-D3//B3LYP-D3 method, unless otherwise stated. Furthermore, the secondary deuterium kinetic isotope effect (KIE) and equilibrium isotope effect (EIE) for the formation of **A** were evaluated using the DFT-computed harmonic frequencies and the Bigeleisen-Mayer equation via PyQuiver code.[28] The 3D geometries were illustrated by CYLview.[29]

On-the-fly quasi-classical DFT MD simulations were first initiated by normal mode sampling at 353.15 K from the optimized transition states for the reaction of benzaldehyde with 2,3-dimethyl-1,3-butadiene by using Progdyn code developed by Singleton.[30] When the total energy of the initial geometry did not agree (within 1.0 kcal mol^{-1}) with the desired energy, the normal mode sampling was rejected and resampled again. Each trajectory was then propagated in both the forward and backward directions with a time step of 1 fs until either the cycloaddition product formed (the formed C-C and C-O bonds < 1.6 Å) or the two reactants separated from each other by more than ~3.3 Å. If the trajectory did not meet either of these two stopping criteria, it could be propagated in one direction only up to 900 fs. The motions of the atomic nucleus were integrated using the Velocity-Velert algorithm. The energy and force of all structures at each step were calculated on-the-fly by the abovementioned B3LYP-D3 (gas phase) and SMD B3LYP-D3 (benzene solution) methods. A total of 100-130 trajectories were propagated in the four key cases in the gas and solution phases. Furthermore, 130 trajectories were propagated to investigate the effect of the OEEF (-0.003 au) on the reaction dynamics in solution in the three key Fe-catalyzed cases. In total, 1310 quasi-classical DFT trajectories were performed. Only productive trajectories without recrossing events were used for our analysis and discussion.

Preparation of [Fe(TPP)]BF$_4$. Fe(TPP)Cl (0.5 mmol, 352 mg) and AgBF$_4$ (0.5 mmol, 97 mg) was dissolved in dry CH_2Cl_2 (10 mL) and stirred for 6 h in dry flask. The reaction mixture was filtered and concentrated to dryness. The complex was used without further purification.

Preparation of deuterated benzaldehyde.

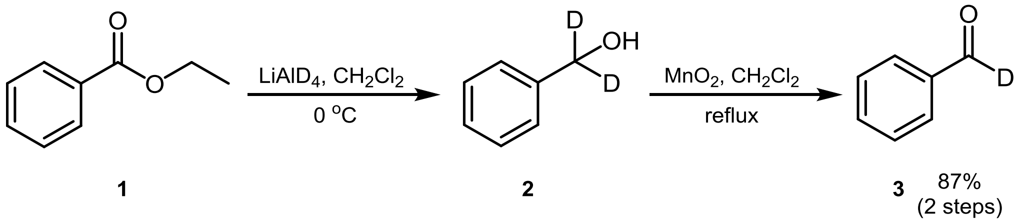

To a solution of the above ester **1** (1.00 g) in CH_2Cl_2 (20 ml) were added LiAlD$_4$ (0.336 g, 8 mmol, 1.2 eq.) at 0 ℃. After stirring for 30 min at same temperature, the reaction was quenched with saturated aqueous ammonium chloride. The aqueous phase was extracted twice with CH_2Cl_2 (2×20 mL). The combined organic phase was washed with brine (30 mL), dried over sodium sulfate, filtered and concentrated in vacuo, which was used for the next step without further purification. To a solution of the above mixture including **2** in CH_2Cl_2 (25 ml), MnO_2 (13 g 25 eq.) was added, heat up to reflux temperature. After stirring for 2.5 h at the same temperature, the mixture was filtered and concentrated in vacuo. The residue was purified by flash column chromatography (2% EtOAc in hexane) to afford **3** (0.620 g, 87% for 2 steps) as a colorless oil.

Procedure for the cycloaddition of aldehydes with dienes.

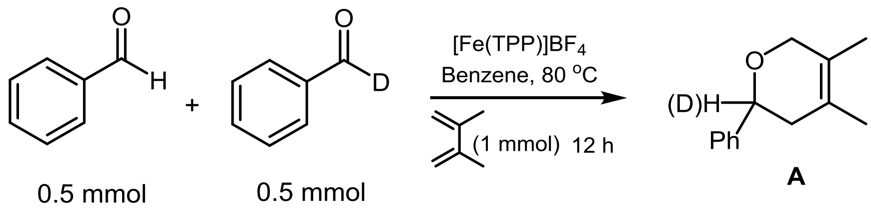

To a flask [Fe(TPP)]BF$_4$ (0.050 mmol, 45 mg) was added, followed by the aldehyde (1 mmol, 1:1), the diene (82 mg, 1.0 mmol) and dry benzene (2.0 mL) under the protection of argon. The flask was sealed and stirred under the indicated conditions. The reaction mixture was diluted with hexane (10 mL), passed through a short silica gel pad and washed with

hexane/ethyl acetate = 10/1, concentrated in vacuo. The crude H-1 NMR was determined with $CDCl_3$. The crude product was purified by flash column chromatography using basic Al_2O_3 (hexane/ethyl acetate, 100:1).

Procedure for the cycloaddition of acrolein with dienes.

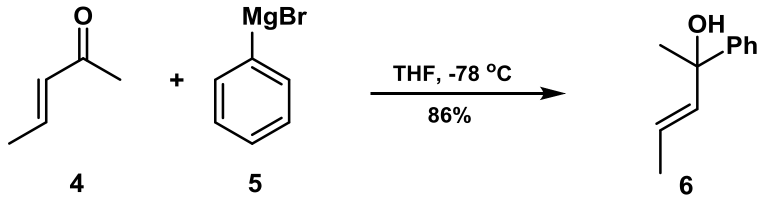

4 (12.0 mmol, 1 g) and THF 50 mL were added to a flask, cooled down to −78 °C with the use of dry ice in acetone bath. Followed by addition of **5** (24 mmol, 0.5 M in toluene) under the protection of argon. The reaction mixture was stirred under the indicated condition for 2.5 h. The reaction mixture was quenched with water (1 mL) and filtered, extracted with diethyl ether and dried over sodium sulfate. concentrated and purified by column chromatography to give **6**.

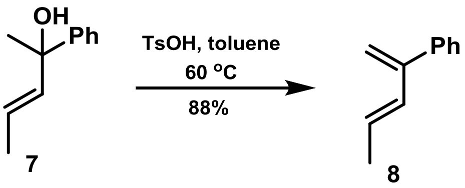

To a flask, **7** (10.3 mmol, 1.7 g, 1 eq), toluene 50 mL, and TsOH (0.51 mmol, 0.05 eq. 88 mg) was added. The reaction mixture was stirred under the indicated conditions monitored by TLC. The reaction mixture was purified by column chromatography.

Procedure for the cycloaddition of acrolein with dienes.

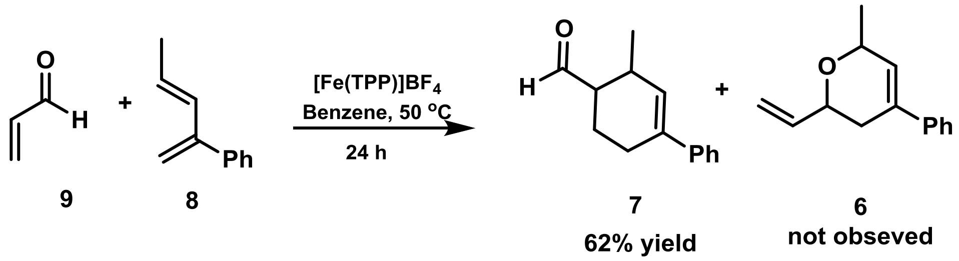

To a flask was added $[Fe(TPP)]BF_4$ (0.050 mmol, 45 mg), followed by the aldehyde **9**

(56 mg, 1.0 mmol), the diene **8** (144 mg, 1.0 mmol) and dry benzene (4.0 mL) under the protection of argon. The flask was sealed and stirred under the indicated condition. The reaction mixture was diluted with hexane (10 mL), purified by flash column chromatography using basic Al_2O_3 (hexane/ethyl acetate) to afford 7 in 62% yield.

Supplementary Discussion

For the formation of A product, considerable charge transfer from the electron-rich diene substrate to the carbonyl substrate was also found to occur in the uncatalyzed transition state ($\Delta q_{(diene)}$: 0.23) and, to a greater extent, in the Fe-catalyzed transition states ($\Delta q_{(diene)}$: 0.46-0.50), based on Mulliken charge analysis ($\Delta q_{(PhCHO)}$: -0.38~-0.40 in ^{4,6}**TS1A$_{endo}$** and ^{4,6}**TS2A$_{endo}$**; see Supplementary Table 7). Therefore, the reacting carbonyl oxygen becomes more negatively charged and can interact with the cationic Fe(III) metal more strongly in these transition states with some zwitterionic character. Such bond strengthening is analogous to the enhanced hydrogen bonds in DA or ODA transition states observed in previous works.[31-33] Moreover, our Mulliken charge analysis further showed ~15-27 % charge transfer from the diene to the porphyrin ligand in ^{4,6}**TS1A$_{endo}$** and ^{4,6}**TS2A$_{endo}$** ($\Delta q_{(por)}$: -0.07~-0.13; see Supplementary Table 6), but almost no transfer to the metal was observed ($\Delta q_{(Fe)}$: 0.00~0.03). These charge transfer features were also supported by the NPA charge analysis (see Supplementary Table 7). Hence, the porphyrin ligand acts as an auxiliary electron reservoir during the ODA reaction.

The enhanced Fe-O axial interaction could partly render the iron atom displacement out of the porphyrin (its four N mean) plane in these transition states relative to their catalyst complexes (Δd_{4N}: 0.09-0.14 Å in ^{4,6}**TS1A$_{endo}$** and 0.03-0.06 Å in ^{4,6}**TS2A$_{endo}$**). Also, the Fe(III) atom was essentially positioned in the porphyrin plane in the six-coordinate ^{4,6}**IA** ((Δd_{4N}: 0.00-0.02 Å), while the Fe(III) atom was significantly displaced 0.16-0.35 from the four N mean plane in the five-coordinate ^{4,6}**IVA**. Such structural features are analogous to the Fe(II) six-coordinate oxy form and five-coordinate deoxy form in heme/porphyrin systems.[34-35]

In contrast to the structures optimized in solution by the SMD B3LYP-D3 (with dispersion) method, the bond between the Fe-O elongated in ^{4,6}**IA** (by 0.03 Å) and in ^{4,6}**IVA** (by 0.01-0.03 Å) by the SMD B3LYP (no dispersion) method; the bond between the Fe and the reacting carbonyl O was also elongated in ^{4,6}**TS1A$_{endo}$** (by 0.02-0.05 Å) and ^{4,6}**TS2A$_{endo}$** (by 0.03-0.04 Å) as well as the bond between the Fe and nonreacting carbonyl O elongated in ^{4,6}**TS1A$_{endo}$** (by 0.10-0.12 Å) (see Supplementary Figure 3B). In addition, the new C-C bond

distance in ^{4,6}**TS1A$_{endo}$** and ^{4,6}**TS2A$_{endo}$** was found to be shorter by 0.02-0.14 Å by the SMD B3LYP (no dispersion) method than that by the SMD B3LYP-D3 (with dispersion) method, whereas the new C-O bond distances were longer by 0.14-0.24 Å. In the uncatalyzed reaction, the new C-C bond distance was not changed, and the new C-O bond distance was elongated by 0.03 Å (see Supplementary Figure 3B).

Our distortion/interaction analysis[36-37] further indicated that a smaller total distortion energy (~35.8-40.5 kcal mol^{-1}) and, especially, a larger interaction energy were vital in lowering the reaction barrier for the Fe-catalyzed reaction compared to the uncatalyzed reaction (see Supplementary Figure 4).

Moreover, the inclusion of an oriented external electric field (OEEF)[38-41] along the reacting Fe-O bond axis (as the OEEF positive z-axis (***Fz***) with a strength of -0.0015 or -0.0030 au) could further reduce the reaction barrier to roughly 14.8-21.7 kcal mol^{-1} by the SMD B3LYP-D3 method (Supplementary Table 10 and Supplementary Figure 5), primarily due to favorable electrostatic interactions with the OEEF.

Additiontly, a [Ru(TBPP)(CO)]SbF_6 complex was also reported to catalyze the cycloaddition of an aldehyde with a diene, but in a lower yield than the [Fe(TPP)]SbF_6 complex.[42-43] Our calculations suggest that the ground-state Ru(III) complex (^{2}**IA$_{Ru}$**) is doublet state, but its ODA barrier to form **A** is much higher (23.5 kcal mol^{-1}, Supplementary Figure 6b). Extraordinarily, in contrast to the related ^{2}**IA** Fe(III) complex, a very small spin density on the metal center (-0.02) and substantial spin density on the porphyrin ligand (1.01) in ^{2}**IA$_{Ru}$**, as well as a considerable increase in spin density on the Ru metal (0.61) in ^{2}**TS1A$_{endo,Ru}$** were found in our study. These results indicate significant Ru(II) character in this formal Ru(III) complex with a strong π-accepting CO ligand, which could qualitatively account for the higher barrier. Such an electronic feature of the Ru(II) metal with a porphyrin radical cation, which is analogous to Compound I in the P450 enzyme can maximize back-donation from the two filled d_{xz} and d_{yz} orbitals from the d6 Ru(II) center to the two vacant π* orbitals of the CO ligand (see Supplementary Figure 6c).

No natural or artificial biocatalyst has been reported to catalyze this challenging ODA

reaction with unactivated substrates so far, even though some natural or artificial enzymes (Diels-Alderases) were found or developed to catalyze DA reactions.[44-47] Three examples (one antibody and two natural enzymes) were reported to catalyze ODA reaction only with an activated/unstable carbonyl substrate.[48-50] In this connection, effects of using one axial 5-methyl-1H-imidazole (histidine) ligand for the six-coordinate mode pathway were also examined by the SMD B3LYP-D3 method. Our predicted free-energy barrier for the ODA reaction using this biological ligand in the quartet and doublet states is roughly 21.7-22.6 kcal mol^{-1} (see Supplementary Table 16). As to the abovementioned OEEF-enhanced reactivity, the reaction barrier should be further reduced by including negative electrostatic potential around the diene substrate. Also, if the precursor (**IIA**-like) complex can be stabilized by non-covalent interactions in an enzymatic pocket, the unfavorable entropy cost and, thus, the overall barrier should be significantly diminished. Therefore, we believe and propose that combination of the cationic iron(III) porphyrin catalyst with rational design or directed revolution of artificial metalloenzymes[48-50] as well as the inclusion of favorable electrostatic interactions in the active site should be the key elements to realize the first biocatalyst for this challenging ODA reaction with unactivated substrates.

For the admixture spin states, additional calculations have been done by other methods. However, compared to the experimental observation (admixture quartet and sextet states), the DLPNO-CCSD(T) method was found to significantly over-stabilize the high spin state ($\Delta G_{S\text{-}Q}$ = ~-11.8 kcal mol^{-1}, see Supplementary Table 17). Recently, Harvey and coworkers performed their multireference approach CASPT2/CC to calculate the quintet-triplet gaps of a series of non-heme Fe(IV)=O species.[51-52] Their works also concluded that "current implementations of the local coupled-cluster method are not sufficiently accurate. DLPNO-CCSD(T) systematically overstabilizes the quintet state". Unfortunately, the practical, advanced and state-of-the-art DLPNO-CCSD(T) method predicted the wrong ground state. Furthermore, Radoń performed CCSD(T) calculations on simplified heme-type Fe(III) complexes and compared with different DFT methods.[53] This study summarized that "Although the DFT results are highly functional-dependent, it is shown that the spin-state energetics of a full heme model and its simplified mimic remain in a good linear correlation."

PBE0 method was shown to be the best method for energy gap between the quartet and sextet states for the heme-like Fe(III) cases in this study. It is also in agreement with our new calculations on the Fe(III) porphyrin complex coordinating with two acetone that the PBE0-D3 method qualitatively reproduce the observed admixture states (see Supplementary Table 17). Our PBE0-D3 results did support our B3LYP-D3 results that the key transition states have the similar energy for the quartet and sextet states (see Supplementary Table 2).

2-Phenyl-1,3-butadiene was then employed to examine the mechanism of the regioselective formation of product **B**. Our SMD B3LYP-D3 results indicated that the lowest-energy pathway via $^{4}\mathbf{TS2B}_{\mathbf{endo\text{-}p}}$, to form desired *para*-product $^{4}\mathbf{VIB}_{\mathbf{endo\text{-}p}}$ has a lower barrier (21.5 kcal mol^{-1}) than that via $^{4}\mathbf{TS2B}_{\mathbf{exo\text{-}m}}$ to form *meta*-product $^{4}\mathbf{VIB}_{\mathbf{exo\text{-}m}}$ by about 4.1 kcal mol^{-1}. The regioselectivity in the formation of **B** in the Fe(III)-catalyzed reaction is much higher than that in the uncatalyzed reaction (0.8 kcal mol^{-1}). Similarly, when 2-methyl-1,3-butadiene was used, our DFT study supported a high kinetic preference for the formation of desired *para*-product **C** ($^{4}\mathbf{VIC}_{\mathbf{endo\text{-}p}}$) over *meta*-product $^{4}\mathbf{VIC}_{\mathbf{exo\text{-}m}}$ by approximately 4.3 kcal mol^{-1}. These DFT results are in agreement with the experimental observations. The presence of a Ph or Me group on C2 of the diene was found to increase the HOMO coefficient and the negative charge on C1 of the diene, which enhance the regioselective addition of the electrophilic carbonyl carbon to this electron-rich C1 atom of the diene. Moreover, in the Fe-catalyzed asynchronous transition states, the significant C-C bond formation that leads to the pronounced charge transfer from the diene substrate to the carbonyl group makes the *para*-carbon more electron-deficient by ~0.04-0.07. Therefore, an electron-donating methyl or conjugated phenyl group can stabilize the relatively electron-deficient *para*-carbon, favoring the formation of *para*-form product **B** or **C** due to favorable electrostatic matching and larger orbital overlap of the diene (HOMO-type) with the carbonyl (LUMO-type).

Additional calculations were also carried out to understand the *cis*-stereoselective formation of product **D** from the reaction with 2-phenyl-1,3-pentadiene. As summarized in Figure S12, the lowest barrier to form the *cis*-product via $^{4}\mathbf{TS1D}_{\mathbf{endo\text{-}cis\text{-}p}}$ is approximately 15.5 kcal mol^{-1}, which is smaller than that to form the *trans*-product via $^{4}\mathbf{TS1D}_{\mathbf{exo\text{-}trans\text{-}p}}$ by 3.6 kcal

mol^{-1} by the SMD B3LYP-D3 method. In fact, the *cis*-product was derived from the commonly favorable *endo*-cycloaddition, while the *exo*-cycloaddition, leading to the *trans*-product, requires a higher barrier due to the absence of secondary orbital interactions.[54-56] Dispersion interactions not only reduce the reaction barriers for the formation of the *cis*- and *trans*-products, they can also slightly increase the kinetic preference for the *cis*-stereoselectivity.

In short, our systematic DFT static simulations revealed that iron has pronounced effects on the reactivity, mechanism and secondary deuterium KIE of the ODA reaction. The Lewis-acid Fe(III) metal activates the carbonyl substrate by lowering its LUMO energy, facilitating it to react with the electron-rich diene. During the reaction, along with substantial charge transfer from the diene substrate to the metal-ligated carbonyl, the porphyrin ligand also functions as an auxiliary electron reservoir: a redox-inactive noninnocent ligand. The Fe(III)-catalyzed ODA reaction was suggested to follow a concerted asynchronous pathway in the quartet and sextet states with five- or six-coordination mode (two-mode reactivity with admixed spin states) based on the computed energies and computed/measured secondary KIE values. Surprisingly, the secondary KIE values was computed to be abnormally large, even though substantial C-C bond formation occurs in the five-coordinate transition states, due to a noticeable and possibly unrecognized EIE resulted from the change in the coordination of the metal. Moreover, the iron can adapt and change its coordinate mode or other Fe-ligand bonding features to activate the carbonyl substrate. Also, dispersion interactions and an OEEF can reduce the ODA reaction barriers. Interestingly, compared to the ground-state quartet Fe(III) catalyst, the ground-state electronic structure of the related formal Ru(III) catalyst with one CO ligand was shown to become a closed-shell Ru(II) center with doublet porphyrin radical cation character, which increases the reaction barrier. Our combined computational and experimental studies further indicated that steric and electronic effects are the key elements in the unique chemoselectivity for the C=O bond of α,β-unsaturated aldehydes.

Compared to the static geometry optimization methods (usually at 0 K), MD simulations with sufficient trajectories include thermal and entropy effects and, thus, should give more realistic ensemble structure. Moreover, MD simulations can give us branching ratio of

different products in bifurcate pathways/reactions, while the static geometry optimization cannot. In addition, driven by the thermal effect (and possibly non-equilibrium effect), reaction trajectories do not have to follow minimum energy path (derived from the static geometry optimization) and have shown unusual mechanistic details (e.g. roaming) in some post-transition state dynamics.[57-58] Broader entrance channel was observed in our Fe-catalyzed ODA trajectories which should be related to derivate from the minimum energy path.

Supplementary References

1. Frisch, M. J. et al. Gaussian 09, Revision D.01, Gaussian, Inc., Wallingford CT, 2009.
2. Neese, F. The ORCA program system. *Wiley Inter discip. Rev.: Comput. Mol. Sci.* **2**, 73-78 (2012).
3. Riplinger, C., Sandhoefer, B., Hansen, A. & Neese, F. Natural triple excitations in local coupled cluster calculations with pair natural orbitals. *J. Chem. Phys.* **139**, 134101 (2013).
4. Lee, C., Yang, W. & Parr, R. G. Development of the Colle-Salvetti correlation-energy formula into a functional of the electron density. *Phys. Rev. B* **37**, 785-789 (1988).
5. Becke. A. D. Density - functional thermochemistry. III. The role of exact exchange. *J. Chem. Phys.* **98**, 5648-5652 (1993).
6. Grimme, S., Antony, J., Ehrlich, S. & Krieg, H. A consistent and accurate ab initio parametrization of density functional dispersion correction (DFT-D) for the 94 elements H-Pu. *J. Chem. Phys.* **132**, 154104 (2010).
7. Weigenda, F. & Ahlrichs, R. Balanced basis sets of split valence, triple zeta valence and quadruple zeta valence quality for H to Rn: Design and assessment of accuracy. *Phys. Chem. Chem. Phys*. **7,** 3297-3305 (2005).
8. Andrae, D., Häußermann, U., Dolg, M., Stoll, H. & Preuß, H. Energy-adjustedab initio pseudopotentials for the second and third row transition elements. *Theor. Chim. Acta.* **77,** 123-141 (1990).
9. Fukui, K. The path of chemical reactions-the IRC approach. *Acc. Chem. Res.***14**, 363-368 (1981).
10. Marenich, A. V., Cramer, C. J. & Truhlar, D. G. Universal Solvation Model Based on Solute ElectronDensity and on a Continuum Model of the Solvent Defined by the Bulk Dielectric Constant and Atomic Surface Tensions. *J. Phys. Chem. B* **113**, 6378-6396 (2009).
11. Wang, Z., Danovich, D., Ramanan, R. & Shaik, S. Oriented-external electric fields create absolute enantioselectivity in Diels-Alder reactions: Importance of the molecular dipole moment. *J. Am. Chem. Soc.* **140**, 13350-13359 (2018).
12. Shaik, S., Mandal, D.& Ramanan, R. Oriented electric fields as future smart reagents in chemistry. *Nat. Chem.* **8**, 1091-1098 (2016).
13. Shaik, S., Ramanan, R., Danovich, D. & Mandal, D. Structure and reactivity/selectivity control by oriented-external electric fields. *Chem. Soc. Rev.* **47**, 5125-5145 (2018).
14. Ciampi, S., Darwish, N., Aitken, H. M., Díez-Pérez, I. & Coote, M. L. Harnessing electrostatic catalysis in single molecule, electrochemical and chemical systems: a rapidly growing experimental tool box. *Chem. Soc. Rev.* **47**, 5146-5164 (2018).
15. Warshel, A., Sharma, P. K., Kato, M., Xiang, Y., Liu, H. & Olsson, M. H. M. Electrostatic Basis for

Enzyme Catalysis. *Chem. Rev.* **106**, 3210-3235 (2006).
16. Fried, S. D., Bagchi, S. & Boxer, S. G. Extreme electric fields power catalysis in the active site of ketosteroid isomerase. *Science* **346**, 1510-1514 (2014).
17. Zhao, Y. & Truhlar, D. G. Density Functionals with Broad Applicability in Chemistry. *Acc. Chem. Res.* **41**, 157- 167 (2008).
18. Adamo, C. & Barone, V. Toward reliable density functional methods without adjustable parameters: The PBE0 model. *J. Chem. Phys.* **110**, 6158-6169 (1999).
19. Chai, J.-D. & Head-Gordon, M. Systematic optimization of long-range corrected hybrid density functionals. *J. Chem. Phys.* **128**, 084106 (2008).
20. Radoń, M. Spin-State Energetics of Heme-Related Models from DFT and Coupled Cluster Calculations. *J. Chem. Theory Comput.* **10**, 2306-2321 (2014).
21. Phung, Q. M., Feldt, M., Harvey, J. N. & Pierloot, K. Toward Highly Accurate Spin State Energetics in First-Row Transition Metal Complexes: A Combined CASPT2/CC Approach. *J. Chem. Theory Comput.* **14**, 2446-2455 (2018).
22. Phung, Q. M., Martín-Fernández, C., Harvey, J. N. & Feldt, M. Ab Initio Calculations for Spin-Gaps of Non-Heme Iron Complexes. *J. Chem. Theory Comput.* **15**, 4297-4304 (2019).
23. Feldt, M., Phung, Q. M., Pierloot, K., Mata, R. A. & Harvey, J. N. Limits of Coupled-Cluster Calculations for Non-Heme Iron Complexes. *J. Chem. Theory Comput.* **15**, 922-937 (2019).
24. Kepp, K. P. Theoretical study of spin crossover in 30 iron complexes. *Inorg. Chem.* **55**, 2717-2727 (2016).
25. Cirera, J. Via-Nadal, M. & Ruiz, E. Benchmarking Density Functional Methods for Calculation of State Energies of First Row Spin-Crossover Molecules. *Inorg. Chem.* **57**, 14097-14105 (2018).
26. Kelly, C. P., Cramer, C. J. & Truhlar. D. G. Aqueous solvation free energies of ions and ion− water clusters based on an accurate value for the absolute aqueous solvation free energy of the proton. *J. Phys. Chem. B* **110**, 16066-16081 (2006).
27. Fujiwara, K., Kurahashi, T. & Matsubara. S. Cationic iron (III) porphyrin-catalyzed [4+ 2] cycloaddition of unactivated aldehydes with simple dienes. *J. Am. Chem. Soc.* **134**, 5512-5515 (2012).
28. Anderson, T. L. & Kwan, E. E. PyQuiver 2016, www.github.com/ekwan/PyQuiver.
29. Legault, C. Y. CYL View, version 1.0 b; Universite de Sherbrooke, Sherbrooke, Quebec, Canada, **2009**; http://www.cylview.org.
30. Kelly, K. K., Hirschi, J. S. & Singleton. D. A. Newtonian kinetic isotope effects. Observation, prediction, and origin of heavy-atom dynamic isotope effects. *J. Am. Chem. Soc.* **131**, 8382-8383 (2009).
31. Wang, Z., Danovich, D., Ramanan, R. & Shaik, S. Oriented-external electric fields create absolute enantioselectivity in Diels–Alder reactions: Importance of the molecular dipole moment. *J. Am. Chem. Soc.* **140**, 13350-13359 (2018).
32. Shaik, S., Mandal, D. & Ramanan. R. Oriented electric fields as future smart reagents in chemistry. *Nat. Chem.* **8,** 1091-1098 (2016).
33. Ciampi, S., Darwish, N., Aitken, H. M., Díez-Pérez, I. & Coote, M. L. Harnessing electrostatic catalysis in single molecule, electrochemical and chemical systems: a rapidly growing experimental tool box. *Chem. Soc. Rev.* **47**, 5146-5164 (2018).
34. Momenteau, M., Scheidt, W. R., Eigenbrot, C. W. & Reed. C. A. A deoxymyoglobin model with a sterically unhindered axial imidazole. *J. Am. Chem. Soc.* **110**, 1207-1215 (1988).
35. Zhao, J., Peng, Q., Wang, Z., Xu, W., Xiao, H., Wu, Q., Sun, H.-L., Ma, F., Zhao, J., Sun, C.-J., Zhao, J. & Li, J. Proton mediated spin state transition of cobalt heme analogs. *Nat. Commun.* **10**, 2303 (2019).
36. Morokuma, K. Why do molecules interact? The origin of electron donor-acceptor complexes, hydrogen bonding and proton affinity. *Acc. Chem. Res.* **10**, 294-300 (1977)
37. Ess, D. H. & Houk. K. N. Distortion/interaction energy control of 1, 3-dipolar cycloaddition reactivity. *J. Am. Chem. Soc.* **129**, 10646-10647 (2007).
38. Fried, S. D., Bagchi, S. & Boxer. S. G. Extreme electric fields power catalysis in the active site of

ketosteroid isomerase. *Science* **346**, 1510-1514 (2014).
39. Zhang, X., Du, H., Wang, Z., Wu, Y.-D. & Ding. K. Experimental and Theoretical Studies on the Hydrogen-Bond-Promoted Enantioselective Hetero-Diels−Alder Reaction of Danishefsky's Diene with Benzaldehyde. *J. Org. Chem*. **71**, 2862-2869 (2006).
40. Anderson, C. D., Dudding, T., Gordillo, R. & Houk. K. N. Origin of Enantioselection in Hetero-Diels-Alder Reactions Catalyzed by Naphthyl-TADDOL. *Org. Lett.* **10**, 2749-2752 (2008).
41. Thomas, L. L., Tirado-Rives, J. & Jorgensen, W. L. Quantum Mechanical/Molecular Mechanical Modeling Finds Diels-Alder Reactions Are Accelerated Less on the Surface of Water Than in Water. *J. Am. Chem. Soc.* **132**, 3097-3104 (2010).
42. Terada, T., Kurahashi, T. & Matsubara, S. Diastereoselective Construction of *Trans*-Fused Octalone Framework via Ruthenium-Porphyrin-Catalyzed Cycloaddition. *Org. Lett.* **16**, 2594-2597 (2014).
43. Shaik, S., Kumar, D., de Visser, S. P., Altun, A. & Thiel, W. Theoretical Perspective on the Structure and Mechanism of Cytochrome P450 Enzymes. *Chem. Rev*. **105**, 2279-2328 (2005).
44. Eschenbrenner-Lux, V., Kumar, K. & Waldmann. H. The Asymmetric Hetero-Diels-Alder Reaction in the Syntheses of Biologically Relevant Compounds. *Angew. Chem. Int. Ed.* **53**, 11146-11157 (2014).
45. Jeon, B. S., Wang, S. A., Ruszczycky, M. W. & Liu. H.-W. Natural [4+2]-Cyclases. *Chem. Rev.* **117**, 5367-5388 (2017).
46. Walsh, C. T. & Tang, Y. Recent advances in enzymatic complexity generation: cyclization reactions. *Biochemistry* **57**, 3087-3104 (2018).
47. Chen, Q., Gao, J., Jamieson, C., Liu, J., Ohashi, M., Bai, J., Yan, D., Liu, B., Che, Y., Wang, Y., Houk, K. N. & Hu, Y. Enzymatic Intermolecular Hetero-Diels-Alder Reaction in the Biosynthesis of Tropolonic Sesquiterpenes. *J. Am. Chem. Soc.* **141**, 14052-14056 (2019).
48. Arnold, F. H. Innovation by Evolution: Bringing New Chemistry to Life. *Angew. Chem. Int. Ed.* **58**, 14420-14426 (2019).
49. Schwizer, F., Okamoto, Y., Heinisch, T., Gu, Y., Pellizzoni, M. M., Lebrun, V., Reuter, R., Köhler, V., Lewis, J. C. & Ward, T. R. Artificial metalloenzymes: reaction scope and optimization strategies. *Chem. Rev.* **118**, 142-231 (2018).
50. Reetz, M. T. Directed Evolution of Artificial Metalloenzymes: A Universal Means to Tune the Selectivity of Transition Metal Catalysts? *Acc. Chem. Res.* **52,** 336-344 (2019).
51. Phung, Q. M., Martín-Fernández, C., Harvey, J. N. & Feldt, M. Ab Initio Calculations for Spin-Gaps of Non-Heme Iron Complexes. *J. Chem. Theory Comput.* **15,** 4297-4304 (2019).
52. Feldt, M., Phung, Q. M., Pierloot, K., Mata, R. A. & Harvey. J. N. Limits of Coupled-Cluster Calculations for Non-Heme Iron Complexes. *J. Chem. Theory Comput.* **15**, 922-937 (2019).
53. Radoń, M. Spin-state energetics of heme-related models from DFT and coupled cluster calculations. *J. Chem. Theory Comput*. **10**, 2306-2321 (2014).
54. Wannere, C. S., Paul, A., Herges, R., Houk, K. N., Schaefer, H. F., III. & von Ragué Schleyer, P. The existence of secondary orbital interactions. *J. Comput. Chem.* **28**, 344-361 (2007).
55. Houk, K. N. & Strozier, R. W. Lewis acid catalysis of Diels-Alder reactions. *J. Am. Chem. Soc*. **95**, 4094-4096 (1973).
56. Hoffmann, R. & Woodward, R. B. Orbital Symmetries and endo-exo Relationships in Concerted Cycloaddition Reactions. *J. Am. Chem. Soc.* **87**, 4388-4389 (1965).
57. Sun, L., Song, K. & Hase, W. L. A SN2 Reaction That Avoids Its Deep Potential Energy Minimum. *Science* **296**, 875-878 (2002).
58. Ammal, S. C., Yamataka, H., Aida, M. & Dupuis, M. Dynamics-Driven Reaction Pathway in an

Intramolecular Rearrangement. *Science* **299**, 1555-1557 (2003).
59. Townsend, D., Lahankar, S. A., Lee, S. K., Chambreau, S. D., Suits, A. G., Zhang, X., Rheinecker, J.; Harding, L. B. & Bowman, J. M. The Roaming Atom: Straying from the Reaction Path in Formaldehyde Decomposition. *Science* **306**, 1158-1161 (2004).
