# Supplementary material for: Unusual KIE and dynamics effects in the Fe-catalyzed hetero-Diels-Alder reaction of unactivated aldehydes and dienes
Source: Nat Commun. 2020 Apr 15;11:1850. doi: 10.1038/s41467-020-15599-w (PMC7160212; doi:10.1038/s41467-020-15599-w)
Supplement: Supplementary file 3 — Description of Additional Supplementary File [file 41467_2020_15599_MOESM3_ESM.pdf]

Description of Additional Supplementary Files

File Name: Supplementary Data 1

Description: Cartesian coordinates of all optimized structures.

File Name: Supplementary Movie 1

Description: One representative MD trajectory for the uncatalyzed reaction in solution.

File Name: Supplementary Movie 2

Description: One representative MD trajectory for the Fe-catalyzed reaction in the quartet state and six-coordinate form in solution.

File Name: Supplementary Movie 3

Description: One representative MD trajectory for the Fe-catalyzed reaction in the sextet state and five-coordinate form in solution.

File Name: Supplementary Movie 4

Description: One representative MD trajectory for the Fe-catalyzed reaction in the sextet state and five-coordinate form in solution in the presence of the oriented external electric field (OEEF).
