# Supplementary material for: Unusual KIE and dynamics effects in the Fe-catalyzed hetero-Diels-Alder reaction of unactivated aldehydes and dienes
Source: Nat Commun. 2020 Apr 15;11:1850. doi: 10.1038/s41467-020-15599-w (PMC7160212; doi:10.1038/s41467-020-15599-w)
Supplement: Supplementary file 4 — Supplementary Data 1 [file 41467_2020_15599_MOESM4_ESM.pdf]

Supplementary Data

Unusual KIE and Dynamics Effects in the Fe-catalyzed Hetero-Diels-Alder Reaction of Unactivated Aldehydes and Dienes

Yang et al

Cartesian coordinates of all optimized structures

Reaction A

^{2}IA

Fe	0.000065	0.000318	0.000727
N	0.258823	1.389317	-1.426056
N	0.257680	1.393350	1.423871
O	-1.900419	0.418567	-0.000401
C	0.571134	3.371515	-0.003789
C	0.467819	2.737464	-1.234078
C	0.414284	2.459586	-3.468486
H	0.426474	2.581590	-4.543801
C	-4.225894	0.007062	-0.002504
C	0.000322	-0.004542	-3.433406
C	0.467217	2.740745	1.228123
C	0.214014	1.205602	-2.792117
C	0.570890	3.408756	-2.504230
H	0.737492	4.471493	-2.624491
C	-2.838437	-0.398386	-0.001894
H	-2.614040	-1.471670	-0.002801
C	0.211709	1.213554	2.790203
C	0.569610	3.415645	2.496421
H	0.736503	4.478654	2.613731
C	0.411923	2.469298	3.463159
H	0.423349	2.594399	4.538067
C	-4.584629	1.370529	-0.001971
C	-5.926378	1.723948	-0.002614
C	-5.220096	-0.989482	-0.003679
H	-4.933179	-2.038188	-0.004110
C	-6.562979	-0.626967	-0.004262
H	-7.334754	-1.390072	-0.005134
C	-6.912718	0.726992	-0.003744
H	-7.961403	1.010296	-0.004240
N	-0.258825	-1.388684	1.427639
N	-0.257464	-1.392951	-1.422237
C	-0.568417	-3.371306	0.005483
C	-0.466765	-2.736836	1.235628
C	-0.416820	-2.458374	3.469915
H	-0.430565	-2.580109	4.545179
C	-0.003217	0.005553	3.434766
C	-0.464989	-2.740682	-1.226483
C	-0.216200	-1.204553	2.793491
C	-0.571243	-3.407886	2.505773
H	-0.737311	-4.470669	2.626046
C	-0.212850	-1.212966	-2.788721
C	-0.567298	-3.415625	-2.494704
H	-0.732766	-4.478841	-2.611996
C	-0.411907	-2.469002	-3.461598
H	-0.424061	-2.594000	-4.536559
O	1.900425	-0.418370	0.002180
C	4.226053	-0.007813	-0.000152
C	2.838737	0.398196	-0.000406
H	2.614828	1.471626	-0.003078
C	4.584206	-1.371423	0.002428
C	5.925801	-1.725421	0.002369
C	5.220696	0.988312	-0.002773
H	4.934227	2.037162	-0.004810
C	6.563431	0.625204	-0.002767
H	7.335531	1.387988	-0.004770
C	6.912576	-0.728909	-0.000214
H	7.961137	-1.012667	-0.000265
H	0.000248	-0.006020	-4.518408
H	-0.734185	-4.443648	0.007047
H	-0.004541	0.007261	4.519680
H	0.738034	4.443719	-0.005218
H	-6.214196	2.770665	-0.002263
H	-3.801985	2.122422	-0.001113
H	3.801246	-2.122959	0.004354
H	6.213162	-2.772256	0.004306

^{4}IA

Fe	-0.000052	0.000057	-0.000187
N	-0.002298	-0.002989	-2.002457
N	0.379370	1.969695	-0.003618
O	-2.164576	0.460693	0.001963
C	0.449007	2.378093	-2.428694
C	0.200993	1.077381	-2.837495
C	-0.131373	-0.676031	-4.208900
H	-0.257224	-1.339648	-5.054502
C	-4.500948	0.035501	0.001562
C	-0.454154	-2.385446	-2.420432
C	0.526403	2.788013	-1.106985
C	-0.207201	-1.085944	-2.833761
C	0.122354	0.663258	-4.211201
H	0.246512	1.324265	-5.059087
C	-3.098816	-0.348177	0.001244
H	-2.873545	-1.427397	0.000283
C	0.528661	2.791391	1.096945

C	0.780888	4.138640	-0.689459
H	0.932956	4.970985	-1.364331
C	0.782275	4.140741	0.674748
H	0.935718	4.975153	1.346756
C	-4.874986	1.393102	0.002641
C	-6.220858	1.733742	0.002853
C	-5.483895	-0.969794	0.000695
H	-5.185652	-2.015682	-0.000147
C	-6.831565	-0.622336	0.000909
H	-7.594979	-1.394034	0.000237
C	-7.196784	0.727309	0.001989
H	-8.248537	0.999248	0.002155
N	0.002290	0.003112	2.002119
N	-0.379377	-1.969602	0.003271
C	-0.448959	-2.377978	2.428352
C	-0.200969	-1.077254	2.837124
C	0.131374	0.676138	4.208537
H	0.257222	1.339747	5.054134
C	0.454137	2.385528	2.420080
C	-0.526359	-2.787926	1.106646
C	0.207182	1.086036	2.833398
C	-0.122316	-0.663150	4.210843
H	-0.246442	-1.324174	5.058721
C	-0.528656	-2.791318	-1.097286
C	-0.780802	-4.138569	0.689130
H	-0.932827	-4.970921	1.364016
C	-0.782212	-4.140675	-0.675084
H	-0.935634	-4.975101	-1.347087
O	2.164557	-0.460696	-0.002015
C	4.500952	-0.035629	-0.001195
C	3.098834	0.348126	-0.001144
H	2.873627	1.427356	-0.000204
C	4.874916	-1.393252	-0.002313
C	6.220768	-1.733968	-0.002118
C	5.483962	0.969607	0.000098
H	5.185785	2.015512	0.000954
C	6.831611	0.622061	0.000311
H	7.595062	1.393720	0.001339
C	7.196749	-0.727598	-0.000793
H	8.248482	-0.999596	-0.000620
H	-0.595310	-3.139539	-3.187421
H	-0.588610	-3.129661	3.197966
H	0.595297	3.139625	3.187054
H	0.588692	3.129777	-3.198297
H	-6.519193	2.777679	0.003684
H	-4.098707	2.151708	0.003305
H	4.098587	-2.151812	-0.003317
H	6.519030	-2.777925	-0.002977

^{6}IA

Fe	-0.000006	0.000019	-0.000147
N	-0.000172	0.000870	-2.056123
N	0.379229	2.022316	0.000668
O	-2.114794	0.431526	0.000170
C	0.439490	2.394917	-2.434043
C	0.199512	1.091399	-2.872999
C	-0.124029	-0.670327	-4.251010
H	-0.244303	-1.325566	-5.104153
C	-4.450945	0.024894	0.000442
C	-0.439957	-2.392848	-2.435938
C	0.516752	2.828774	-1.108919
C	-0.200067	-1.088971	-2.873866
C	0.123145	0.673899	-4.250478
H	0.243222	1.329857	-5.103098
C	-3.056577	-0.373302	0.000162
H	-2.835734	-1.451943	-0.000082
C	0.516912	2.827859	1.110893
C	0.758364	4.183526	-0.682406
H	0.903588	5.024788	-1.347981
C	0.758446	4.182962	0.685473
H	0.903762	5.023672	1.351722
C	-4.811778	1.386908	0.000686
C	-6.154088	1.739755	0.000923
C	-5.443673	-0.971914	0.000421
H	-5.155306	-2.020444	0.000229
C	-6.787482	-0.611412	0.000662
H	-7.558442	-1.375466	0.000654
C	-7.139273	0.742025	0.000910
H	-8.188368	1.023996	0.001097
N	0.000185	-0.000840	2.055835
N	-0.379236	-2.022280	-0.000933
C	-0.439404	-2.394902	2.433762
C	-0.199442	-1.091379	2.872708
C	0.124007	0.670359	4.250722
H	0.244249	1.325604	5.103865
C	0.439881	2.392899	2.435665
C	-0.516721	-2.828754	1.108642
C	0.200030	1.089009	2.873579

C	-0.123073	-0.673883	4.250187
H	-0.243103	-1.329853	5.102805
C	-0.516962	-2.827806	-1.111167
C	-0.758347	-4.183497	0.682122
H	-0.903544	-5.024769	1.347690
C	-0.758502	-4.182909	-0.685754
H	-0.903860	-5.023608	-1.352007
O	2.114794	-0.431558	-0.000456
C	4.450945	-0.024944	-0.000065
C	3.056583	0.373263	-0.000158
H	2.835742	1.451905	-0.000006
C	4.811764	-1.386962	-0.000318
C	6.154072	-1.739820	-0.000245
C	5.443681	0.971857	0.000273
H	5.155326	2.020390	0.000470
C	6.787488	0.611341	0.000350
H	7.558455	1.375389	0.000608
C	7.139266	-0.742099	0.000085
H	8.188360	-1.024077	0.000133
H	-0.575380	-3.147388	-3.204645
H	-0.574617	-3.150073	3.201885
H	0.575263	3.147442	3.204377
H	0.574750	3.150088	-3.202160
H	-6.442924	2.786288	0.001120
H	-4.028644	2.138382	0.000695
H	4.028622	-2.138427	-0.000579
H	6.442898	-2.786356	-0.000449

^{2}VIA

Fe	-0.869919	0.050002	0.411305
N	-1.906922	1.255620	-0.787840
N	-1.828173	-1.522450	-0.355538
O	0.702847	-0.141161	-0.731715
C	-2.983941	-0.420622	-2.223995
C	-2.671515	0.882979	-1.877030
C	-2.662110	3.130701	-1.924273
H	-2.810385	4.178139	-2.151884
C	2.998378	-0.130884	-1.295716
C	-1.213067	3.448858	0.077219
C	-2.603442	-1.537448	-1.501517
C	-1.890082	2.640238	-0.817885
C	-3.141319	2.040851	-2.585488
H	-3.768060	2.007639	-3.467028
C	1.893027	-0.013974	-0.378670
H	2.115730	0.194660	0.675934
C	-1.715670	-2.851487	0.017031
C	-2.989872	-2.881185	-1.833257
H	-3.608330	-3.149345	-2.679803
C	-2.445531	-3.694810	-0.887490
H	-2.521740	-4.770345	-0.796173
C	2.783819	-0.390853	-2.666211
C	3.871956	-0.497545	-3.519295
C	4.307607	0.019012	-0.795956
H	4.463191	0.218802	0.261330
C	5.392525	-0.090203	-1.658276
H	6.403796	0.023740	-1.281366
C	5.172806	-0.347785	-3.015673
H	6.020788	-0.432866	-3.689125
N	-0.154221	-1.135871	1.860612
N	-0.242872	1.645472	1.439334
C	1.072052	0.528585	3.191417
C	0.694381	-0.769907	2.891423
C	0.520498	-3.005480	3.056404
H	0.588351	-4.047042	3.341872
C	-0.988196	-3.317204	1.097249
C	0.614921	1.655331	2.526079
C	-0.255597	-2.513561	1.953155
C	1.115627	-1.925573	3.634152
H	1.770814	-1.895759	4.495006
C	-0.440776	2.980963	1.126675
C	0.947374	3.001953	2.894754
H	1.592816	3.270387	3.720943
C	0.286985	3.823602	2.030766
H	0.282800	4.905219	1.998436
H	-1.283815	4.522992	-0.057931
H	1.742275	0.677075	4.031808
H	-0.983014	-4.386626	1.280077
H	-3.597037	-0.574800	-3.105760
H	3.719402	-0.696689	-4.575327
H	1.767821	-0.502664	-3.030748

^{4}VIA

Fe	-0.915399	-0.001816	0.401853
N	-1.886964	1.409939	-0.615548
N	-1.890735	-1.398173	-0.633144
O	0.779726	0.003970	-0.832071
C	-3.102104	0.017572	-2.240440
C	-2.713221	1.235866	-1.713923

C	-2.533643	3.463568	-1.467874
H	-2.607916	4.539629	-1.554858
C	3.112553	0.004698	-1.261768
C	-1.042549	3.422055	0.523337
C	-2.716513	-1.208264	-1.729239
C	-1.772186	2.781873	-0.461957
C	-3.118441	2.505898	-2.241791
H	-3.771201	2.633794	-3.095319
C	1.948031	-0.000703	-0.405971
H	2.117658	-0.009453	0.681626
C	-1.779478	-2.772186	-0.496697
C	-3.125017	-2.470547	-2.272919
H	-3.778071	-2.586079	-3.127983
C	-2.542687	-3.439310	-1.511034
H	-2.619687	-4.514010	-1.611483
C	2.978874	0.016263	-2.665697
C	4.115640	0.021309	-3.460905
C	4.390359	-0.001766	-0.669641
H	4.483614	-0.010525	0.413665
C	5.525132	0.003234	-1.473333
H	6.513238	-0.001698	-1.024442
C	5.385434	0.014775	-2.865133
H	6.271879	0.018722	-3.492952
N	-0.237882	-1.415317	1.643721
N	-0.234432	1.394276	1.661482
C	0.912739	-0.022420	3.316425
C	0.538770	-1.242053	2.779395
C	0.368888	-3.469952	2.523200
H	0.429433	-4.546051	2.620065
C	-1.051471	-3.426565	0.480413
C	0.541953	1.204793	2.794738
C	-0.345778	-2.788508	1.484368
C	0.915033	-2.512638	3.326283
H	1.515582	-2.642004	4.217235
C	-0.338655	2.769613	1.519266
C	0.921572	2.467456	3.357469
H	1.522569	2.584085	4.249872
C	0.377871	3.436160	2.566481
H	0.441222	4.510807	2.676776
H	-1.039683	4.506629	0.528141
H	1.524847	-0.028869	4.211890
H	-1.051569	-4.511117	0.471658
H	-3.755199	0.023919	-3.106298
H	4.024721	0.030238	-4.542552
H	1.984597	0.021050	-3.100558

^{6}**VIA**

Fe	-0.927502	-0.006519	0.409229
N	-1.751379	1.601448	-0.447440
N	-1.981504	-1.180885	-0.811905
O	0.780947	0.005571	-0.810781
C	-3.060059	0.530415	-2.231579
C	-2.567414	1.637982	-1.551449
C	-2.196705	3.806959	-1.066847
H	-2.172242	4.888454	-1.033855
C	3.111864	0.041290	-1.248257
C	-0.759502	3.393639	0.912549
C	-2.785511	-0.784975	-1.881857
C	-1.507229	2.949925	-0.126420
C	-2.852410	3.001735	-1.936684
H	-3.479600	3.279051	-2.773742
C	1.949425	0.059296	-0.389350
H	2.120502	0.124472	0.696047
C	-1.991439	-2.559843	-0.856322
C	-3.284622	-1.922842	-2.570783
H	-3.936038	-1.884206	-3.433743
C	-2.780693	-3.036232	-1.922974
H	-2.943554	-4.079768	-2.156342
C	2.975093	-0.048956	-2.649008
C	4.109492	-0.064421	-3.447517
C	4.390479	0.115465	-0.662658
H	4.486333	0.185031	0.418240
C	5.522880	0.100273	-1.469671
H	6.511546	0.157744	-1.025654
C	5.380130	0.010232	-2.858292
H	6.264718	-0.002029	-3.488617
N	-0.372577	-1.619906	1.468328
N	-0.136899	1.166103	1.828668
C	0.847312	-0.549061	3.315002
C	0.384798	-1.657714	2.612842
C	0.077676	-3.825948	2.081482
H	0.065067	-4.907385	2.040123
C	-1.302524	-3.408509	0.062049
C	0.596247	0.768350	2.950907
C	-0.579396	-2.968893	1.120647
C	0.664506	-3.021476	3.001126
H	1.238903	-3.300313	3.874808
C	-0.119341	2.545809	1.868100

C	1.058027	1.906515	3.664584
H	1.643381	1.868058	4.573775
C	0.605954	3.020659	2.979665
H	0.753864	4.063956	3.223744
H	-0.652514	4.463711	1.053258
H	1.433976	-0.724953	4.209908
H	-1.388563	-4.478164	-0.096092
H	-3.700227	0.705179	-3.089117
H	4.015980	-0.133915	-4.526763
H	1.980240	-0.105371	-3.078960

^{2}IIA$_{endo}$

Fe	0.626425	-0.009074	-0.038039
N	0.361054	0.683722	1.824636
N	0.365000	1.831218	-0.785447
O	2.525403	0.398977	0.154435
C	0.102929	3.076143	1.315645
C	0.187059	2.000475	2.187042
C	0.193158	0.851030	4.124526
H	0.165661	0.533692	5.158901
C	4.856854	0.039724	0.032840
C	0.596407	-1.398910	3.110480
C	0.167199	2.986267	-0.066458
C	0.387778	-0.031473	3.004652
C	0.076482	2.110422	3.618651
H	-0.074300	3.040175	4.151758
C	3.474674	-0.343306	-0.151935
H	3.266379	-1.331272	-0.580173
C	0.357297	2.205503	-2.111148
C	0.017331	4.107864	-0.959550
H	-0.156169	5.127027	-0.639612
C	0.129208	3.622754	-2.226363
H	0.072104	4.161223	-3.163334
C	5.196706	1.293967	0.579378
C	6.533290	1.628465	0.743977
C	5.864771	-0.867624	-0.343860
H	5.592595	-1.832627	-0.764383
C	7.202387	-0.524871	-0.175813
H	7.984613	-1.219825	-0.464235
C	7.533438	0.720652	0.366925
H	8.577992	0.988413	0.498068
N	0.912261	-0.708391	-1.899107
N	0.894873	-1.858282	0.714632
C	1.263234	-3.088412	-1.384821
C	1.147712	-2.016665	-2.259461
C	1.040428	-0.878443	-4.199828
H	1.030771	-0.564431	-5.235499
C	0.566296	1.356076	-3.185973
C	1.137027	-3.010165	-0.004662
C	0.828430	-0.001183	-3.079200
C	1.241670	-2.126508	-3.692741
H	1.428623	-3.049500	-4.226350
C	0.839751	-2.245364	2.038365
C	1.243472	-4.136090	0.885858
H	1.433475	-5.153311	0.568471
C	1.056680	-3.662754	2.150093
H	1.064774	-4.211013	3.083267
O	-1.269534	-0.395574	-0.333677
C	-3.304568	-1.500241	0.119929
C	-2.028530	-0.935195	0.496118
H	-1.703452	-1.035107	1.537606
C	-3.799296	-1.358681	-1.191719
C	-5.012450	-1.944086	-1.530662
C	-4.031421	-2.229924	1.078685
H	-3.642905	-2.327347	2.089329
C	-5.237600	-2.825984	0.727164
H	-5.799248	-3.396005	1.460465
C	-5.726124	-2.681392	-0.575958
H	-6.670281	-3.143589	-0.849880
H	0.585005	-1.834902	4.104040
H	1.451392	-4.066785	-1.814856
H	0.530075	1.785107	-4.181912
H	-0.048578	4.059760	1.747904
H	6.806151	2.591657	1.163816
H	4.403749	1.978549	0.862921
H	-3.227310	-0.784003	-1.910733
H	-5.405679	-1.835513	-2.536740
C	-4.364072	2.075860	-0.547824
C	-4.345985	1.556660	0.847867
C	-3.283240	1.726818	1.653574
H	-2.416839	2.295475	1.338762
H	-3.270464	1.338210	2.669041
C	-5.585779	0.843866	1.334714
H	-6.440064	1.529931	1.388007
H	-5.870610	0.032413	0.655327
H	-5.435415	0.419569	2.331429
C	-5.697119	2.534283	-1.096362
H	-6.409093	1.701711	-1.161896

H	-6.156090	3.293329	-0.450484
H	-5.586916	2.960728	-2.097350
C	-3.254026	2.113766	-1.303355
H	-2.297833	1.746200	-0.950507
H	-3.277202	2.512930	-2.313966

$^{4}\mathbf{IIA}_{endo}$

Fe	-0.630385	-0.034418	-0.049337
N	-0.320618	-0.657094	1.827522
N	-0.354367	-1.895486	-0.717619
O	-2.785453	-0.490786	0.223240
C	-0.006947	-3.062746	1.418473
C	-0.100526	-1.954272	2.245269
C	-0.084735	-0.716112	4.125141
H	-0.036119	-0.352731	5.143457
C	-5.109424	-0.010154	0.101824
C	-0.555782	1.477875	3.030987
C	-0.107838	-3.024102	0.037376
C	-0.327893	0.111358	2.975817
C	0.049186	-1.995768	3.673171
H	0.235982	-2.896021	4.243931
C	-3.697517	0.287388	-0.076736
H	-3.444641	1.271096	-0.505018
C	-0.369298	-2.317828	-2.031722
C	0.046123	-4.167942	-0.818545
H	0.252588	-5.171188	-0.469601
C	-0.110733	-3.729951	-2.099739
H	-0.063693	-4.301208	-3.017646
C	-5.522132	-1.243167	0.642224
C	-6.876415	-1.502442	0.804347
C	-6.062572	0.953459	-0.271830
H	-5.734601	1.903029	-0.688380
C	-7.418791	0.688199	-0.106824
H	-8.159274	1.428443	-0.393333
C	-7.822491	-0.537986	0.430284
H	-8.880971	-0.745662	0.559150
N	-0.969699	0.596395	-1.923704
N	-0.922232	1.840715	0.625177
C	-1.372040	2.987872	-1.505460
C	-1.251298	1.883516	-2.335417
C	-1.167874	0.661358	-4.223922
H	-1.178904	0.303211	-5.245116
C	-0.619684	-1.516191	-3.133646
C	-1.208245	2.963201	-0.128256
C	-0.904312	-0.160856	-3.075194
C	-1.386233	1.926678	-3.765804
H	-1.610529	2.819929	-4.334286
C	-0.838270	2.277005	1.933060
C	-1.312141	4.115588	0.722839
H	-1.531195	5.117085	0.375823
C	-1.080253	3.691325	1.998495
H	-1.072909	4.273666	2.910771
O	1.476747	0.467869	-0.392182
C	3.516707	1.590613	0.056217
C	2.225470	1.027363	0.418323
H	1.897132	1.146166	1.462616
C	4.040711	1.401014	-1.235719
C	5.271010	1.957283	-1.564754
C	4.231523	2.336663	1.007822
H	3.821838	2.469730	2.006297
C	5.457391	2.901912	0.668997
H	6.011305	3.483450	1.399381
C	5.975032	2.711212	-0.616176
H	6.933903	3.148316	-0.880131
H	-0.526429	1.953955	4.005439
H	-1.594412	3.943463	-1.968487
H	-0.599221	-1.984636	-4.111881
H	0.181445	-4.024189	1.884044
H	-7.204174	-2.450346	1.220087
H	-4.768531	-1.971869	0.923478
H	3.478249	0.806415	-1.946709
H	5.686341	1.810279	-2.557191
C	4.411525	-2.085618	-0.502053
C	4.444932	-1.509662	0.871823
C	3.405653	-1.627770	1.715717
H	2.517727	-2.189097	1.450480
H	3.430570	-1.194621	2.712662
C	5.710905	-0.799769	1.292584
H	6.556290	-1.496899	1.344006
H	5.984907	-0.017293	0.575961
H	5.598294	-0.337285	2.277126
C	5.722483	-2.576491	-1.075563
H	6.437926	-1.752998	-1.194715
H	6.196769	-3.315003	-0.416969
H	5.576773	-3.038664	-2.056018
C	3.277083	-2.147186	-1.218428
H	2.336487	-1.754009	-0.852367
H	3.265414	-2.587254	-2.212206

^{6}IIA$_{endo}$

Fe	0.604361	0.072809	-0.126263
N	0.278957	1.630044	1.162475
N	0.492772	1.347795	-1.718513
O	2.731558	0.485450	0.015738
C	0.079434	3.442732	-0.495286
C	0.098230	2.950658	0.810915
C	0.019795	2.886828	3.069195
H	-0.058361	3.125299	4.122053
C	5.026889	-0.073756	0.238536
C	0.393163	0.404014	3.297248
C	0.260498	2.703649	-1.665310
C	0.242827	1.565854	2.538737
C	-0.070473	3.739928	2.004465
H	-0.236986	4.809230	2.019607
C	3.609216	-0.374484	0.170958
H	3.312876	-1.430667	0.263313
C	0.625239	1.007192	-3.046304
C	0.233359	3.233342	-3.005427
H	0.065591	4.272931	-3.255720
C	0.457616	2.187684	-3.856706
H	0.509867	2.207857	-4.937675
C	5.488439	1.252835	0.130665
C	6.850498	1.512127	0.196000
C	5.939590	-1.130045	0.411478
H	5.573702	-2.150835	0.493299
C	7.303733	-0.863512	0.475763
H	8.013334	-1.674133	0.608464
C	7.755738	0.455394	0.367973
H	8.820731	0.663702	0.417947
N	1.012107	-1.506877	-1.398707
N	0.764297	-1.223323	1.484609
C	1.165367	-3.322961	0.256221
C	1.179058	-2.827758	-1.050416
C	1.288747	-2.757272	-3.306579
H	1.374069	-2.991791	-4.359858
C	0.888156	-0.275903	-3.528886
C	0.973482	-2.585132	1.428175
C	1.060309	-1.437661	-2.772130
C	1.360892	-3.615158	-2.245012
H	1.517006	-4.686197	-2.262845
C	0.629524	-0.885688	2.814277
C	0.969411	-3.120624	2.765743
H	1.113016	-4.164283	3.014922
C	0.758814	-2.072931	3.620036
H	0.696515	-2.097013	4.700492
O	-1.444203	-0.458529	-0.355748
C	-3.477626	-1.611810	0.021424
C	-2.179347	-1.097645	0.412855
H	-1.824611	-1.324076	1.428505
C	-4.026415	-1.290238	-1.234494
C	-5.268456	-1.801639	-1.589869
C	-4.179402	-2.445064	0.910041
H	-3.750406	-2.679780	1.881280
C	-5.416674	-2.964630	0.542561
H	-5.960782	-3.612637	1.222531
C	-5.959150	-2.641479	-0.705683
H	-6.927265	-3.043286	-0.991043
H	0.323062	0.514610	4.374962
H	1.314410	-4.392005	0.373613
H	0.958571	-0.384086	-4.606846
H	-0.092723	4.507944	-0.612117
H	7.216153	2.531044	0.113853
H	4.765929	2.051682	-0.002398
H	-3.473451	-0.632977	-1.895640
H	-5.703017	-1.553769	-2.553466
C	-4.356709	2.068324	-0.188917
C	-4.331121	1.408062	1.146032
C	-3.259401	1.481202	1.954481
H	-2.386884	2.068288	1.695474
H	-3.246866	0.987586	2.923047
C	-5.577785	0.667959	1.572404
H	-6.421173	1.357620	1.700049
H	-5.879892	-0.069746	0.820413
H	-5.426560	0.146966	2.522125
C	-5.692018	2.580301	-0.681610
H	-6.403808	1.757992	-0.828345
H	-6.148603	3.269207	0.040126
H	-5.586131	3.106192	-1.634622
C	-3.250877	2.182326	-0.943025
H	-2.294942	1.778047	-0.635186
H	-3.276962	2.679089	-1.909611

^{2}TS1A$_{endo}$

Fe	-0.445600	-0.270938	0.088549
N	-0.468340	-0.731331	2.041944
N	-0.648662	-2.211147	-0.396203

O	-2.431136	-0.179822	0.157833
C	-0.546622	-3.182078	1.858303
C	-0.484269	-1.997298	2.580240
C	-0.478351	-0.600525	4.348800
H	-0.483734	-0.150490	5.333126
C	-4.579376	0.728442	-0.236343
C	-0.423924	1.522665	3.020829
C	-0.653741	-3.272318	0.476316
C	-0.470421	0.137327	3.111718
C	-0.477004	-1.922405	4.020134
H	-0.486011	-2.780627	4.679769
C	-3.132902	0.721328	-0.326411
H	-2.648474	1.556379	-0.848948
C	-0.782539	-2.750499	-1.653796
C	-0.786384	-4.511840	-0.250748
H	-0.816983	-5.492660	0.206124
C	-0.875086	-4.187476	-1.570427
H	-0.992401	-4.846786	-2.420859
C	-5.270289	-0.311062	0.417111
C	-6.656116	-0.274600	0.486095
C	-5.288117	1.796853	-0.815117
H	-4.746028	2.594608	-1.317173
C	-6.677273	1.827215	-0.741796
H	-7.229791	2.649013	-1.186322
C	-7.357596	0.792663	-0.092362
H	-8.442167	0.815975	-0.035325
N	-0.489516	0.202218	-1.867874
N	-0.338298	1.682521	0.572206
C	-0.122313	2.627029	-1.686675
C	-0.284207	1.450717	-2.407505
C	-0.506084	0.069722	-4.173875
H	-0.579221	-0.376638	-5.157237
C	-0.779808	-2.032209	-2.841313
C	-0.161067	2.730556	-0.302823
C	-0.614498	-0.657583	-2.934175
C	-0.292224	1.374679	-3.847785
H	-0.157065	2.221493	-4.508420
C	-0.338013	2.236429	1.833383
C	-0.054207	3.969892	0.425236
H	0.091880	4.939835	-0.032277
C	-0.172862	3.664980	1.747367
H	-0.143564	4.332750	2.598651
O	1.427331	-0.381236	-0.043026
C	3.097405	1.283065	0.341555
C	2.223276	0.179976	0.840102
H	1.790685	0.379036	1.825419
C	3.252609	1.486432	-1.035864
C	4.063613	2.521822	-1.499987
C	3.749996	2.128571	1.246452
H	3.617634	1.978956	2.316053
C	4.558763	3.164503	0.780465
H	5.059077	3.820809	1.486691
C	4.719980	3.360252	-0.593974
H	5.349464	4.167672	-0.956875
H	-0.419271	2.086745	3.947890
H	0.030688	3.541322	-2.250722
H	-0.881524	-2.589854	-3.766829
H	-0.557021	-4.109664	2.421752
H	-7.197832	-1.070949	0.987107
H	-4.703426	-1.125510	0.856703
H	2.713305	0.842891	-1.723000
H	4.177093	2.681452	-2.568740
C	3.762423	-2.197813	-0.720391
C	4.191586	-1.499284	0.468499
C	3.310926	-1.214505	1.531794
H	2.487362	-1.888625	1.747169
H	3.759882	-0.783649	2.423652
C	5.536429	-0.841008	0.463375
H	6.318366	-1.542828	0.152104
H	5.538401	-0.018383	-0.266922
H	5.797227	-0.425563	1.437727
C	4.652378	-2.195077	-1.945089
H	4.899401	-1.174462	-2.258608
H	5.598928	-2.715495	-1.757572
H	4.160702	-2.695545	-2.782910
C	2.529046	-2.766572	-0.765019
H	1.885472	-2.862270	0.096407
H	2.139873	-3.187680	-1.686635

^{4}TS1A$_{endo}$

Fe	0.361228	-0.181604	-0.341746
N	0.095033	1.424407	-1.515179
N	0.689471	-1.316889	-1.969958
O	2.610003	0.293143	-0.407355
C	0.238868	0.346293	-3.721243
C	0.038365	1.440732	-2.892249
C	-0.348591	3.565534	-2.255078
H	-0.549467	4.627759	-2.205094

C	4.739757	0.582874	0.614230
C	-0.172799	3.163209	0.203638
C	0.558333	-0.931799	-3.284395
C	-0.135560	2.721538	-1.109713
C	-0.246465	2.772042	-3.358437
H	-0.342251	3.050349	-4.399997
C	3.310449	0.295322	0.606706
H	2.847241	0.067429	1.581913
C	1.018647	-2.653347	-1.995052
C	0.811886	-2.050542	-4.155016
H	0.775978	-2.008493	-5.235926
C	1.103082	-3.115423	-3.356644
H	1.352965	-4.127598	-3.647444
C	5.419489	0.884329	-0.580368
C	6.781218	1.155752	-0.546419
C	5.434550	0.556546	1.835060
H	4.900805	0.322971	2.753489
C	6.799424	0.829237	1.864050
H	7.340734	0.810324	2.804931
C	7.469541	1.128159	0.674140
H	8.534591	1.341298	0.695681
N	0.779511	-1.755203	0.842457
N	0.231701	0.996665	1.292175
C	0.446007	-0.713282	3.046699
C	0.711361	-1.796589	2.219250
C	1.227845	-3.894919	1.580114
H	1.477582	-4.947078	1.532680
C	1.216351	-3.456609	-0.881318
C	0.235392	0.586405	2.608569
C	1.092143	-3.035253	0.435566
C	0.983976	-3.129694	2.684495
H	0.998339	-3.427023	3.725285
C	-0.013768	2.351802	1.316745
C	0.002499	1.711735	3.475936
H	-0.036078	1.656986	4.556311
C	-0.143786	2.805580	2.675871
H	-0.334825	3.831083	2.963919
O	-1.598994	-0.628240	-0.291738
C	-3.277286	1.082890	-0.220814
C	-2.589228	-0.050087	-0.924380
H	-2.409865	0.156598	-1.990764
C	-3.134924	1.228366	1.164267
C	-3.752815	2.290377	1.823556
C	-4.042739	2.007979	-0.938395
H	-4.143390	1.904139	-2.017061
C	-4.662310	3.069775	-0.278636
H	-5.251128	3.787979	-0.842190
C	-4.519249	3.212058	1.104300
H	-4.998803	4.040706	1.617578
H	-0.374071	4.215312	0.373981
H	0.422587	-0.892820	4.116608
H	1.469528	-4.497797	-1.052238
H	0.165106	0.507389	-4.791698
H	7.313494	1.389277	-1.463566
H	4.861798	0.898699	-1.511470
H	-2.514867	0.518468	1.701610
H	-3.624774	2.406093	2.896246
C	-3.537469	-2.466083	0.926752
C	-4.350218	-1.749728	-0.014004
C	-3.847645	-1.372751	-1.282803
H	-3.168892	-2.050292	-1.793858
H	-4.561877	-0.904421	-1.956737
C	-5.639473	-1.147274	0.454871
H	-6.264356	-1.891320	0.961632
H	-5.429374	-0.354093	1.187343
H	-6.208302	-0.703438	-0.363814
C	-3.989802	-2.585817	2.366837
H	-4.182572	-1.602165	2.810322
H	-4.916864	-3.165231	2.449647
H	-3.230906	-3.086844	2.973117
C	-2.301379	-2.913347	0.557783
H	-1.956420	-2.961409	-0.464550
H	-1.613761	-3.309165	1.298970

^{6}TS1A$_{endo}$

Fe	0.334725	0.345880	0.084083
N	0.540060	1.082821	2.007836
N	0.561610	2.241336	-0.677810
O	2.591011	0.286287	0.103988
C	0.660434	3.477726	1.450578
C	0.620338	2.409922	2.352767
C	0.614293	1.253926	4.296588
H	0.632526	0.947753	5.334783
C	4.711411	-0.725121	-0.251537
C	0.465549	-1.042661	3.243926
C	0.644157	3.401998	0.054245
C	0.538778	0.352630	3.169900
C	0.663811	2.522615	3.792498

H	0.731520	3.454576	4.339152
C	3.255862	-0.685455	-0.265852
H	2.736361	-1.586566	-0.630233
C	0.572492	2.586753	-2.007315
C	0.696247	4.526257	-0.849280
H	0.765937	5.562869	-0.544999
C	0.652968	4.023734	-2.120684
H	0.683373	4.571152	-3.054228
C	5.456346	0.379756	0.201621
C	6.843545	0.316579	0.208262
C	5.367230	-1.886247	-0.694856
H	4.783257	-2.735190	-1.042808
C	6.758018	-1.944442	-0.686121
H	7.269677	-2.839032	-1.027370
C	7.492732	-0.843910	-0.235008
H	8.578165	-0.888632	-0.227713
N	0.407170	-0.436826	-1.830912
N	0.387008	-1.591861	0.842848
C	0.212826	-2.828542	-1.278421
C	0.279266	-1.761178	-2.178387
C	0.308395	-0.604104	-4.120497
H	0.298582	-0.297194	-5.158615
C	0.504564	1.686844	-3.074994
C	0.259550	-2.752754	0.116299
C	0.419501	0.294503	-2.995327
C	0.219970	-1.870845	-3.616848
H	0.123951	-2.800531	-4.163139
C	0.390257	-1.939430	2.173837
C	0.186807	-3.874316	1.021258
H	0.082794	-4.908162	0.717952
C	0.269423	-3.373501	2.289663
H	0.247507	-3.919154	3.224383
O	-1.618189	0.428081	0.138044
C	-3.121869	-1.411817	0.418443
C	-2.405492	-0.218039	0.960617
H	-2.010514	-0.367122	1.975503
C	-3.146492	-1.639954	-0.963695
C	-3.793554	-2.765324	-1.473017
C	-3.742866	-2.320504	1.283753
H	-3.710555	-2.151590	2.358209
C	-4.390437	-3.444277	0.772680
H	-4.867444	-4.148739	1.447933
C	-4.419053	-3.666819	-0.607210
H	-4.922281	-4.543550	-1.004722
H	0.463472	-1.475757	4.239671
H	0.104915	-3.819924	-1.707457
H	0.513049	2.115835	-4.072475
H	0.727512	4.472663	1.880477
H	7.426222	1.164103	0.556340
H	4.927672	1.265305	0.539834
H	-2.631870	-0.943195	-1.617450
H	-3.802845	-2.943841	-2.544710
C	-3.863545	2.218605	-0.619418
C	-4.418162	1.372229	0.409762
C	-3.694973	1.038792	1.571963
H	-2.977205	1.748612	1.972040
H	-4.237256	0.493383	2.340487
C	-5.681588	0.623112	0.119134
H	-6.466860	1.292083	-0.249970
H	-5.491147	-0.114377	-0.675056
H	-6.052256	0.084136	0.992190
C	-4.580633	2.340363	-1.947038
H	-4.731855	1.360642	-2.414668
H	-5.569780	2.798451	-1.829261
H	-4.009452	2.960654	-2.642067
C	-2.653373	2.813694	-0.436731
H	-2.124739	2.824336	0.505506
H	-2.163173	3.345084	-1.247005

^{2}IIIA$_{endo}$

Fe	0.396236	0.371494	0.128280
N	0.397672	0.478375	2.128985
N	0.776649	2.336917	0.004702
O	2.330257	0.090897	0.213832
C	0.615797	2.912678	2.388512
C	0.464502	1.625998	2.885176
C	0.358495	-0.061271	4.375216
H	0.321440	-0.678736	5.263279
C	4.409236	-0.904317	-0.301129
C	0.202297	-1.905442	2.688501
C	0.799304	3.230794	1.050524
C	0.336333	-0.566038	3.027537
C	0.422533	1.296732	4.287339
H	0.456887	2.022865	5.089244
C	2.976767	-0.756046	-0.427649
H	2.447531	-1.422863	-1.118960
C	1.057734	3.069904	-1.127817

C	1.087879	4.555778	0.562325
H	1.158249	5.437694	1.185789
C	1.262650	4.452928	-0.784567
H	1.499077	5.233852	-1.495566
C	5.150310	-0.085061	0.574828
C	6.523794	-0.255755	0.673030
C	5.056577	-1.888877	-1.071264
H	4.476534	-2.516382	-1.743504
C	6.433908	-2.053405	-0.967557
H	6.939444	-2.810450	-1.558586
C	7.163596	-1.237858	-0.096845
H	8.239134	-1.366710	-0.015646
N	0.450654	0.258205	-1.874060
N	0.112379	-1.605904	0.251378
C	-0.111952	-2.120452	-2.142025
C	0.168874	-0.854410	-2.637197
C	0.658038	0.774532	-4.116275
H	0.848564	1.367903	-5.001123
C	1.069865	2.572521	-2.422844
C	-0.113192	-2.467656	-0.798993
C	0.757171	1.265148	-2.767585
C	0.281850	-0.533217	-4.036340
H	0.106511	-1.233808	-4.842601
C	0.071924	-2.380403	1.392626
C	-0.309357	-3.806215	-0.306142
H	-0.521799	-4.663624	-0.930849
C	-0.181416	-3.754210	1.048364
H	-0.267098	-4.560398	1.765013
O	-1.568096	0.688157	0.054184
C	-3.032565	-1.257276	0.402985
C	-2.489408	0.028921	0.986809
H	-1.888946	-0.192487	1.867059
C	-3.219441	-1.444320	-0.969951
C	-3.793086	-2.622880	-1.448951
C	-3.419528	-2.272044	1.286961
H	-3.263276	-2.141042	2.355757
C	-3.998784	-3.447503	0.810418
H	-4.295081	-4.225423	1.508413
C	-4.188926	-3.625098	-0.561704
H	-4.639161	-4.540034	-0.936178
H	0.154135	-2.627501	3.496916
H	-0.314275	-2.906854	-2.861462
H	1.296628	3.264774	-3.226901
H	0.652134	3.725673	3.106237
H	7.103838	0.369457	1.344660
H	4.631459	0.667397	1.159961
H	-2.893905	-0.678521	-1.664130
H	-3.930225	-2.756815	-2.518582
C	-3.549763	1.883002	-0.884182
C	-4.332452	1.484076	0.130836
C	-3.601679	1.018057	1.370032
H	-3.149305	1.862913	1.910139
H	-4.273801	0.512849	2.070258
C	-5.827419	1.356195	0.123135
H	-6.291087	1.747888	-0.784490
H	-6.111619	0.299580	0.221156
H	-6.263920	1.882766	0.981836
C	-3.978510	2.335283	-2.253733
H	-3.603259	1.645034	-3.022403
H	-5.063450	2.395149	-2.357129
H	-3.566488	3.325322	-2.490842
C	-2.063952	1.892264	-0.643072
H	-1.757266	2.747678	-0.032487
H	-1.506514	1.918067	-1.576725

^{4}IIIA$_{endo}$

Fe	0.398600	0.432496	0.161516
N	0.329584	0.510379	2.159267
N	0.642435	2.416102	0.086542
O	2.610168	0.223143	0.285688
C	0.443967	2.946335	2.480831
C	0.341811	1.644822	2.945983
C	0.223176	-0.078499	4.390088
H	0.168248	-0.715508	5.263270
C	4.677369	-0.808833	-0.273023
C	0.168396	-1.890040	2.669764
C	0.606964	3.296524	1.149539
C	0.256173	-0.554767	3.034876
C	0.264986	1.283570	4.334382
H	0.256752	1.991495	5.152954
C	3.236254	-0.633859	-0.347999
H	2.683976	-1.316842	-1.014112
C	0.869677	3.188784	-1.034733
C	0.802029	4.641558	0.683815
H	0.813913	5.516171	1.321094
C	0.976341	4.573524	-0.667068
H	1.155537	5.381403	-1.364564
C	5.461553	0.018770	0.553396

C	6.835662	-0.171726	0.609410
C	5.281539	-1.822712	-1.037194
H	4.667813	-2.457605	-1.672008
C	6.659320	-2.009336	-0.976756
H	7.131353	-2.790678	-1.564163
C	7.432728	-1.184253	-0.154456
H	8.508246	-1.329529	-0.106847
N	0.503275	0.348897	-1.840742
N	0.200966	-1.556024	0.232421
C	0.100638	-2.054556	-2.175035
C	0.326426	-0.766508	-2.636158
C	0.722253	0.927579	-4.066301
H	0.885203	1.552882	-4.934499
C	0.937441	2.718971	-2.337482
C	0.052620	-2.416208	-0.838338
C	0.745328	1.396859	-2.708597
C	0.452853	-0.408996	-4.021869
H	0.354597	-1.103478	-4.846168
C	0.116304	-2.345832	1.362894
C	-0.130928	-3.761325	-0.369618
H	-0.286704	-4.617838	-1.011712
C	-0.081797	-3.719339	0.991409
H	-0.188167	-4.534869	1.694411
O	-1.797634	0.660554	0.021702
C	-3.073566	-1.384441	0.404025
C	-2.684521	-0.023588	0.945468
H	-2.106511	-0.152225	1.864055
C	-3.149288	-1.643197	-0.967546
C	-3.577165	-2.889878	-1.427463
C	-3.417801	-2.397055	1.307894
H	-3.346302	-2.208128	2.377303
C	-3.849415	-3.641690	0.850527
H	-4.116532	-4.417057	1.563206
C	-3.933039	-3.890389	-0.521525
H	-4.270065	-4.858706	-0.880689
H	0.098367	-2.626303	3.463286
H	-0.031082	-2.838700	-2.912836
H	1.118929	3.442159	-3.125419
H	0.434840	3.745749	3.214112
H	7.448510	0.461433	1.243674
H	4.973392	0.794191	1.135134
H	-2.852661	-0.872518	-1.669664
H	-3.632708	-3.078284	-2.496368
C	-3.780471	1.740836	-1.005110
C	-4.591313	1.262248	-0.047287
C	-3.910943	0.859045	1.242845
H	-3.575481	1.744169	1.803390
H	-4.585167	0.300988	1.899523
C	-6.068212	1.015567	-0.151675
H	-6.499283	1.357742	-1.094903
H	-6.275747	-0.058718	-0.053584
H	-6.602361	1.516666	0.666309
C	-4.158071	2.183253	-2.392429
H	-3.678687	1.542975	-3.146389
H	-5.235478	2.157633	-2.566437
H	-3.814856	3.208580	-2.586302
C	-2.317071	1.843390	-0.660514
H	-2.109452	2.707954	-0.015620
H	-1.699373	1.937212	-1.554201

^{6}IIIA$_{endo}$

Fe	0.385395	0.410604	0.158180
N	0.363442	0.539495	2.205555
N	0.718312	2.433029	0.018935
O	2.546698	0.161350	0.265889
C	0.623861	2.979387	2.420501
C	0.458970	1.696713	2.946792
C	0.265010	0.009819	4.439376
H	0.190604	-0.593623	5.334896
C	4.614124	-0.867656	-0.280920
C	0.099616	-1.849624	2.737109
C	0.760854	3.319666	1.072926
C	0.248387	-0.506683	3.094061
C	0.389445	1.368492	4.348211
H	0.438314	2.088259	5.155185
C	3.174963	-0.702642	-0.360995
H	2.623595	-1.391944	-1.019105
C	0.932051	3.157572	-1.132246
C	0.996329	4.647859	0.565998
H	1.073700	5.538364	1.176432
C	1.108015	4.547092	-0.793042
H	1.292006	5.339746	-1.506863
C	5.391862	-0.027357	0.539918
C	6.766707	-0.208769	0.599340
C	5.225852	-1.884665	-1.036010
H	4.617153	-2.528703	-1.666263
C	6.604479	-2.061405	-0.971986
H	7.082693	-2.844382	-1.552054

C	7.371062	-1.224077	-0.155421
H	8.447434	-1.361875	-0.105150
N	0.486672	0.265788	-1.898957
N	0.155047	-1.626688	0.283714
C	0.063752	-2.150351	-2.121866
C	0.294642	-0.876173	-2.645952
C	0.672703	0.786753	-4.130264
H	0.821641	1.381071	-5.022645
C	0.938906	2.629972	-2.425483
C	-0.000889	-2.498923	-0.771728
C	0.721295	1.297203	-2.783247
C	0.404689	-0.551464	-4.045836
H	0.295448	-1.261145	-4.855928
C	0.039143	-2.365488	1.441997
C	-0.222240	-3.827584	-0.261368
H	-0.390643	-4.704052	-0.873014
C	-0.192740	-3.746357	1.102023
H	-0.330551	-4.544330	1.819890
O	-1.747276	0.695387	0.042332
C	-3.091275	-1.314740	0.413303
C	-2.654640	0.026440	0.965425
H	-2.073790	-0.125806	1.877644
C	-3.160929	-1.569180	-0.959242
C	-3.633453	-2.797307	-1.425119
C	-3.488711	-2.312064	1.312487
H	-3.422355	-2.127023	2.382814
C	-3.965931	-3.537272	0.848713
H	-4.273463	-4.301099	1.557444
C	-4.042063	-3.782092	-0.524495
H	-4.413625	-4.735786	-0.888574
H	-0.001134	-2.561851	3.550135
H	-0.074946	-2.954244	-2.838011
H	1.111406	3.330216	-3.236919
H	0.681324	3.794451	3.135348
H	7.374768	0.433502	1.228948
H	4.898274	0.750083	1.114246
H	-2.824377	-0.812775	-1.658936
H	-3.681847	-2.983249	-2.494713
C	-3.720070	1.803324	-0.987578
C	-4.530647	1.362680	-0.011643
C	-3.844477	0.952265	1.272844
H	-3.470944	1.830112	1.820349
H	-4.526154	0.421806	1.944471
C	-6.014774	1.155153	-0.093857
H	-6.450183	1.504005	-1.032535
H	-6.249040	0.087200	0.013109
H	-6.523063	1.674483	0.729119
C	-4.103720	2.232896	-2.377458
H	-3.653847	1.565342	-3.125838
H	-5.183725	2.235297	-2.535922
H	-3.733809	3.244113	-2.594355
C	-2.250076	1.876449	-0.668310
H	-2.006871	2.746425	-0.045367
H	-1.644429	1.929275	-1.572781

$^2IIA_{exo}$

Fe	0.587766	-0.408170	0.121485
N	0.593659	-1.771855	-1.360858
N	0.889092	-1.851165	1.481586
O	2.518958	-0.261795	-0.092042
C	0.984114	-3.795045	-0.019124
C	0.787267	-3.127396	-1.218758
C	0.538148	-2.794271	-3.433056
H	0.453014	-2.889878	-4.507796
C	4.630790	0.770385	-0.314553
C	0.222396	-0.314802	-3.303013
C	1.024361	-3.195479	1.231049
C	0.445619	-1.551063	-2.713562
C	0.751904	-3.770346	-2.507540
H	0.876997	-4.833728	-2.665882
C	3.194095	0.780514	-0.150515
H	2.689455	1.751299	-0.075629
C	0.974585	-1.709999	2.847578
C	1.206007	-3.914754	2.467637
H	1.338196	-4.986578	2.539819
C	1.171986	-2.993928	3.469489
H	1.271643	-3.151188	4.535592
C	5.340815	-0.443056	-0.418611
C	6.719370	-0.417405	-0.574573
C	5.313621	1.999969	-0.368296
H	4.757222	2.930498	-0.286647
C	6.695718	2.017327	-0.524876
H	7.228758	2.961846	-0.566970
C	7.394713	0.810478	-0.627338
H	8.474038	0.824182	-0.749240
N	0.594847	0.956830	1.587262
N	0.277747	1.032977	-1.244148
C	0.275614	2.991346	0.245363

C	0.453268	2.318742	1.444888
C	0.653335	1.974366	3.661591
H	0.725135	2.065676	4.737587
C	0.888736	-0.510274	3.537164
C	0.176931	2.387106	-0.999320
C	0.715136	0.729345	2.942469
C	0.499421	2.959897	2.733867
H	0.413920	4.027449	2.891062
C	0.147612	0.886609	-2.612774
C	-0.032208	3.098287	-2.232586
H	-0.145024	4.172081	-2.309255
C	-0.057231	2.169136	-3.229949
H	-0.191722	2.324604	-4.292526
O	-1.328704	-0.695895	0.433668
C	-3.523853	-1.250907	-0.241743
C	-2.157817	-0.855758	-0.482429
H	-1.845751	-0.704501	-1.521691
C	-3.999405	-1.466815	1.066992
C	-5.321115	-1.840209	1.260659
C	-4.376746	-1.426741	-1.346821
H	-3.997932	-1.256772	-2.351292
C	-5.698455	-1.808394	-1.145919
H	-6.363038	-1.946009	-1.992998
C	-6.168444	-2.008762	0.155976
H	-7.202702	-2.301125	0.313731
H	0.110765	-0.282956	-4.381826
H	0.178031	4.070370	0.286337
H	0.970370	-0.543096	4.618580
H	1.112546	-4.871692	-0.061834
H	7.275925	-1.345928	-0.655461
H	4.794107	-1.379548	-0.374429
H	-3.319693	-1.334027	1.902269
H	-5.700629	-2.003196	2.264505
C	-3.394258	2.823767	0.633246
C	-4.285972	2.181052	-0.372626
C	-5.471705	1.669495	-0.018318
H	-5.831602	1.732791	1.003951
H	-6.120312	1.181615	-0.740426
C	-3.792242	2.144691	-1.802045
H	-3.703384	3.153508	-2.223845
H	-2.790591	1.697476	-1.864940
H	-4.470885	1.573747	-2.443259
C	-2.888372	4.210505	0.308551
H	-2.271568	4.207395	-0.598226
H	-3.725291	4.895784	0.123912
H	-2.286764	4.616343	1.127268
C	-3.063540	2.191148	1.767114
H	-3.419727	1.187454	1.976488
H	-2.420075	2.649974	2.512801

^{4}IIA$_{exo}$

Fe	0.451018	-0.342364	0.193758
N	0.186887	-1.928274	-1.013002
N	0.508089	-1.554022	1.782404
O	2.637328	-0.629450	-0.056265
C	0.184625	-3.723017	0.667992
C	0.104236	-3.251831	-0.633004
C	-0.141047	-3.260738	-2.870663
H	-0.278741	-3.522850	-3.911684
C	4.859728	0.075722	-0.512778
C	0.033570	-0.788879	-3.187098
C	0.366572	-2.925587	1.786586
C	0.035318	-1.919447	-2.384216
C	-0.097051	-4.085691	-1.786173
H	-0.192554	-5.163231	-1.755026
C	3.425267	0.267792	-0.374801
H	3.035723	1.280500	-0.568517
C	0.660820	-1.186913	3.102122
C	0.432259	-3.425475	3.133263
H	0.349658	-4.469927	3.404414
C	0.612657	-2.348386	3.948332
H	0.709310	-2.326700	5.026006
C	5.442324	-1.185073	-0.280906
C	6.815044	-1.342156	-0.417019
C	5.662587	1.169713	-0.880493
H	5.204050	2.139890	-1.057231
C	7.038040	1.006343	-1.015719
H	7.663124	1.847303	-1.299304
C	7.610856	-0.247882	-0.783643
H	8.684501	-0.375864	-0.888617
N	0.772547	1.236595	1.376575
N	0.391635	0.874257	-1.404884
C	0.813119	3.033944	-0.303631
C	0.905072	2.556698	0.993642
C	1.141596	2.562464	3.232147
H	1.283419	2.822693	4.273012
C	0.848097	0.106860	3.561633
C	0.557652	2.246965	-1.415610

C	0.910382	1.228715	2.750831
C	1.144983	3.383615	2.143296
H	1.285452	4.456126	2.108173
C	0.202966	0.508324	-2.726148
C	0.454796	2.746220	-2.757813
H	0.543464	3.789295	-3.032712
C	0.229732	1.671170	-3.568007
H	0.100310	1.654005	-4.642374
O	-1.752960	-0.336569	0.455546
C	-3.875853	-1.020359	-0.366533
C	-2.540623	-0.457092	-0.489131
H	-2.219937	-0.152478	-1.497966
C	-4.365594	-1.443692	0.881875
C	-5.649077	-1.965579	0.974317
C	-4.673323	-1.136483	-1.516970
H	-4.283883	-0.805770	-2.476998
C	-5.958429	-1.661552	-1.419951
H	-6.581276	-1.750581	-2.304670
C	-6.444961	-2.071532	-0.174604
H	-7.449563	-2.477547	-0.097135
H	-0.100167	-0.929414	-4.254513
H	0.934823	4.100394	-0.457473
H	0.962951	0.251015	4.630614
H	0.094357	-4.793032	0.821932
H	7.273291	-2.310364	-0.239929
H	4.802553	-2.014908	0.002056
H	-3.729002	-1.345617	1.755385
H	-6.038407	-2.289448	1.934755
C	-2.716077	3.241312	0.753050
C	-3.644918	2.685566	-0.269608
C	-4.787236	2.082649	0.089870
H	-5.083885	2.005177	1.131285
H	-5.463729	1.655168	-0.645187
C	-3.260349	2.845737	-1.724148
H	-3.246669	3.901614	-2.022365
H	-2.253661	2.453650	-1.916328
H	-3.966661	2.327050	-2.380184
C	-2.175231	4.631500	0.506768
H	-1.579805	4.670545	-0.413969
H	-2.990608	5.356367	0.388206
H	-1.539691	4.961826	1.333753
C	-2.378587	2.533618	1.840162
H	-2.730693	1.518509	1.990125
H	-1.717219	2.940951	2.599532

^{6}IIA$_{exo}$

Fe	0.404469	-0.297381	0.221704
N	0.087648	-1.914599	-1.032276
N	0.446875	-1.546830	1.840529
O	2.528487	-0.665987	-0.055676
C	0.017045	-3.679072	0.684849
C	-0.055015	-3.223623	-0.634081
C	-0.307421	-3.221312	-2.880260
H	-0.460961	-3.487001	-3.918356
C	4.783338	-0.071930	-0.494967
C	-0.002682	-0.731784	-3.186656
C	0.245915	-2.908261	1.826045
C	-0.063285	-1.883624	-2.398119
C	-0.301913	-4.047670	-1.791703
H	-0.450849	-5.119526	-1.767846
C	3.373190	0.206791	-0.297859
H	3.052199	1.257195	-0.371742
C	0.624367	-1.172302	3.151405
C	0.301841	-3.409051	3.177368
H	0.178153	-4.447218	3.457680
C	0.534483	-2.338739	3.994718
H	0.638085	-2.332669	5.072143
C	5.274289	-1.390442	-0.424406
C	6.627611	-1.629336	-0.619821
C	5.657964	0.996898	-0.761725
H	5.269615	2.011255	-0.814696
C	7.013575	0.750870	-0.956115
H	7.693932	1.571222	-1.161963
C	7.494910	-0.560153	-0.885115
H	8.553175	-0.752490	-1.037299
N	0.816298	1.309065	1.422825
N	0.413052	0.950098	-1.428931
C	0.918316	3.075784	-0.288715
C	0.984452	2.618268	1.027801
C	1.214622	2.608792	3.276232
H	1.362844	2.872285	4.315595
C	0.857172	0.132960	3.590149
C	0.647879	2.308615	-1.424314
C	0.951004	1.276487	2.795141
C	1.240814	3.434649	2.186540
H	1.410674	4.503444	2.164306
C	0.227195	0.573499	-2.743991
C	0.592357	2.804739	-2.775557

H	0.736708	3.838888	-3.061156
C	0.327474	1.736454	-3.588101
H	0.217451	1.730448	-4.664936
O	-1.725877	-0.231439	0.453032
C	-3.821168	-0.994180	-0.357081
C	-2.531121	-0.342063	-0.483892
H	-2.245932	0.026502	-1.480340
C	-4.242662	-1.518639	0.878722
C	-5.479782	-2.142487	0.972282
C	-4.638578	-1.113344	-1.494394
H	-4.300828	-0.705503	-2.444117
C	-5.875342	-1.742031	-1.395763
H	-6.512801	-1.835165	-2.269485
C	-6.295262	-2.251511	-0.162402
H	-7.262952	-2.738803	-0.083892
H	-0.135453	-0.865498	-4.255897
H	1.082515	4.137097	-0.444619
H	0.981612	0.272813	4.659468
H	-0.122150	-4.744760	0.838487
H	7.015687	-2.641912	-0.567562
H	4.580578	-2.199049	-0.217458
H	-3.590369	-1.420908	1.740657
H	-5.816843	-2.545304	1.922507
C	-2.613217	3.321532	0.712323
C	-3.514087	2.775354	-0.339670
C	-4.647204	2.132081	-0.018558
H	-4.959944	2.011864	1.014101
H	-5.304771	1.720293	-0.779142
C	-3.119637	2.996681	-1.783282
H	-3.119073	4.063476	-2.038936
H	-2.106357	2.626651	-1.981603
H	-3.813212	2.495091	-2.465675
C	-2.054096	4.707850	0.484676
H	-1.421028	4.740350	-0.410566
H	-2.858762	5.438417	0.332768
H	-1.449965	5.034679	1.335994
C	-2.311309	2.612334	1.809330
H	-2.673970	1.600297	1.953882
H	-1.667380	3.016184	2.585314

^{2}TS1A$_{exo}$

Fe	0.230803	-0.075832	0.185083
N	-0.344064	-1.542558	-1.077076
N	0.129467	-1.343250	1.742508
O	2.085237	-0.726385	-0.090378
C	-0.614649	-3.359634	0.551994
C	-0.651001	-2.835703	-0.731528
C	-0.976234	-2.727375	-2.958105
H	-1.196438	-2.921509	-3.999869
C	4.404415	-0.642321	-0.536989
C	-0.348494	-0.313686	-3.199903
C	-0.265469	-2.656576	1.696454
C	-0.534692	-1.457268	-2.436566
C	-1.045848	-3.582020	-1.901657
H	-1.340864	-4.623204	-1.894267
C	3.092391	-0.053511	-0.355559
H	2.999600	1.035128	-0.460218
C	0.346765	-1.055729	3.067913
C	-0.293041	-3.213134	3.028569
H	-0.568131	-4.233702	3.261814
C	0.081392	-2.219344	3.879320
H	0.181590	-2.253794	4.956461
C	4.595390	-2.033432	-0.420775
C	5.863506	-2.568071	-0.601233
C	5.491086	0.200185	-0.833475
H	5.334175	1.272604	-0.920961
C	6.759691	-0.342611	-1.012738
H	7.602306	0.302316	-1.241424
C	6.943189	-1.723866	-0.896557
H	7.933550	-2.147729	-1.036520
N	0.911537	1.359327	1.423144
N	0.399795	1.169845	-1.384573
C	1.275523	3.153094	-0.216177
C	1.296384	2.632349	1.070527
C	1.541139	2.536746	3.308385
H	1.742330	2.733149	4.353679
C	0.776955	0.165766	3.564914
C	0.834755	2.477932	-1.347601
C	1.053898	1.283101	2.789459
C	1.701014	3.370375	2.241718
H	2.057124	4.392794	2.232365
C	0.090455	0.907813	-2.703773
C	0.783044	3.051596	-2.667510
H	1.080074	4.064136	-2.910324
C	0.313858	2.081231	-3.505556
H	0.149391	2.136546	-4.574030
O	-1.555816	0.404363	0.553751
C	-3.415523	-0.890018	-0.216404

C	-2.498019	0.281342	-0.350137
H	-2.226037	0.514401	-1.384439
C	-3.532143	-1.557365	1.009717
C	-4.344312	-2.683959	1.111613
C	-4.118459	-1.355783	-1.335089
H	-4.012468	-0.844605	-2.289508
C	-4.925741	-2.486678	-1.231743
H	-5.459040	-2.853566	-2.103851
C	-5.042921	-3.149611	-0.006687
H	-5.674652	-4.029513	0.074937
H	-0.545930	-0.380855	-4.264893
H	1.610173	4.177248	-0.346053
H	0.921037	0.250059	4.637222
H	-0.904221	-4.398147	0.674265
H	6.020755	-3.638711	-0.513829
H	3.743040	-2.664867	-0.191232
H	-2.955689	-1.204223	1.858403
H	-4.427414	-3.205397	2.060958
C	-2.182716	3.526477	0.657236
C	-3.028218	2.879773	-0.325518
C	-3.760933	1.715061	-0.051264
H	-4.028975	1.485487	0.974675
H	-4.526518	1.443210	-0.771812
C	-3.000524	3.390062	-1.734970
H	-3.277839	4.450678	-1.770930
H	-1.982718	3.322009	-2.145685
H	-3.677392	2.832514	-2.385794
C	-1.561325	4.865071	0.322548
H	-0.960203	4.814209	-0.590254
H	-2.331835	5.630289	0.168105
H	-0.910915	5.201724	1.133067
C	-1.900534	2.905749	1.830981
H	-2.324910	1.954259	2.118689
H	-1.180668	3.335154	2.521081

^{4}TS1A$_{exo}$

Fe	0.167609	-0.095881	0.219750
N	-0.404931	-1.554574	-1.044752
N	0.102187	-1.392649	1.749992
O	2.310134	-0.874572	-0.146184
C	-0.698398	-3.389638	0.562408
C	-0.739546	-2.847914	-0.712912
C	-1.086810	-2.697173	-2.931872
H	-1.325757	-2.872393	-3.972809
C	4.632313	-0.631287	-0.603483
C	-0.413108	-0.299857	-3.158564
C	-0.314408	-2.703152	1.704132
C	-0.612225	-1.445101	-2.402129
C	-1.164003	-3.565907	-1.885788
H	-1.483770	-4.599591	-1.889844
C	3.268315	-0.149335	-0.416653
H	3.109124	0.937153	-0.527340
C	0.363202	-1.121288	3.073536
C	-0.313370	-3.268707	3.029051
H	-0.596641	-4.287017	3.261789
C	0.102444	-2.288278	3.877883
H	0.232758	-2.335855	4.951276
C	4.934199	-1.999629	-0.475802
C	6.240947	-2.434774	-0.655080
C	5.647123	0.290148	-0.911092
H	5.404737	1.346069	-1.007438
C	6.955447	-0.150707	-1.090314
H	7.743221	0.557477	-1.328074
C	7.249605	-1.511313	-0.961805
H	8.270297	-1.856391	-1.100837
N	0.946022	1.304691	1.440640
N	0.405891	1.155540	-1.351082
C	1.319958	3.116697	-0.181863
C	1.345841	2.579277	1.097178
C	1.612106	2.448382	3.330802
H	1.828503	2.631006	4.375523
C	0.824065	0.086808	3.573920
C	0.857453	2.457911	-1.312933
C	1.104411	1.208929	2.805910
C	1.769251	3.294655	2.271118
H	2.138924	4.312218	2.271712
C	0.061581	0.908370	-2.663209
C	0.783896	3.042792	-2.625370
H	1.089135	4.053002	-2.867255
C	0.284508	2.084481	-3.460519
H	0.097084	2.150832	-4.524557
O	-1.716268	0.503196	0.558943
C	-3.535322	-0.829048	-0.260802
C	-2.676101	0.408250	-0.337373
H	-2.380381	0.636886	-1.371824
C	-3.630634	-1.541412	0.939511
C	-4.389025	-2.708916	0.998211
C	-4.205131	-1.291192	-1.398746

H	-4.118140	-0.743626	-2.335073
C	-4.959634	-2.462655	-1.340788
H	-5.468618	-2.823931	-2.229859
C	-5.055342	-3.171523	-0.140543
H	-5.644980	-4.082707	-0.093693
H	-0.631730	-0.355023	-4.219949
H	1.664333	4.138520	-0.302766
H	0.991909	0.156450	4.643610
H	-1.006805	-4.423110	0.678031
H	6.482697	-3.488888	-0.557911
H	4.133588	-2.692688	-0.237328
H	-3.078205	-1.188207	1.804435
H	-4.455077	-3.263715	1.930100
C	-2.271639	3.502465	0.704791
C	-3.184975	2.944581	-0.252986
C	-3.880137	1.729413	-0.021102
H	-4.158174	1.489420	1.001609
H	-4.680971	1.512710	-0.724199
C	-3.225078	3.534402	-1.630540
H	-3.424278	4.611879	-1.594289
H	-2.243652	3.418444	-2.116289
H	-3.980975	3.060278	-2.259746
C	-1.545994	4.790715	0.381949
H	-0.987110	4.717039	-0.556031
H	-2.251837	5.624115	0.281744
H	-0.838032	5.045708	1.173807
C	-1.986120	2.815277	1.847965
H	-2.523690	1.936360	2.171026
H	-1.178435	3.141957	2.496718

^{6}TS1A$_{exo}$

Fe	0.142297	-0.085147	0.256792
N	-0.393525	-1.600584	-1.049326
N	0.147892	-1.420480	1.820864
O	2.246016	-0.851599	-0.127681
C	-0.672139	-3.398820	0.608431
C	-0.730877	-2.881547	-0.688279
C	-1.086476	-2.745138	-2.916498
H	-1.331573	-2.937329	-3.953216
C	4.560335	-0.649609	-0.627418
C	-0.381464	-0.328030	-3.152882
C	-0.268880	-2.727635	1.765034
C	-0.597698	-1.486426	-2.402178
C	-1.168268	-3.604998	-1.859187
H	-1.496647	-4.636245	-1.861895
C	3.215004	-0.138911	-0.399970
H	3.076936	0.951739	-0.480473
C	0.444837	-1.136355	3.129967
C	-0.235100	-3.292350	3.095062
H	-0.510592	-4.309601	3.342245
C	0.203756	-2.311460	3.936952
H	0.357505	-2.369900	5.006867
C	4.829053	-2.028594	-0.544643
C	6.119726	-2.491303	-0.765277
C	5.591910	0.254633	-0.931739
H	5.374868	1.318570	-0.993384
C	6.883979	-0.214234	-1.152113
H	7.684681	0.480093	-1.387410
C	7.145117	-1.585086	-1.068337
H	8.153251	-1.951804	-1.239800
N	0.989524	1.349955	1.480793
N	0.429774	1.178385	-1.377719
C	1.316441	3.138771	-0.180679
C	1.360349	2.624373	1.117690
C	1.658731	2.494580	3.355999
H	1.888905	2.688962	4.395834
C	0.909670	0.094069	3.600093
C	0.870074	2.480041	-1.331096
C	1.164895	1.240264	2.840465
C	1.784597	3.346339	2.292962
H	2.137238	4.370118	2.298166
C	0.093985	0.901864	-2.682755
C	0.800686	3.051222	-2.654989
H	1.096296	4.060833	-2.911782
C	0.316952	2.078560	-3.487887
H	0.140225	2.142745	-4.554103
O	-1.712214	0.467397	0.546288
C	-3.557802	-0.824003	-0.265317
C	-2.658527	0.368528	-0.354983
H	-2.370553	0.616896	-1.384535
C	-3.660423	-1.537755	0.935265
C	-4.450962	-2.682796	0.996333
C	-4.252779	-1.261475	-1.399735
H	-4.158565	-0.713399	-2.334847
C	-5.040174	-2.409518	-1.337268
H	-5.568789	-2.753269	-2.221573
C	-5.142688	-3.119576	-0.137685
H	-5.757713	-4.013586	-0.088089

H	-0.595803	-0.390294	-4.215576
H	1.648245	4.164888	-0.307097
H	1.101106	0.166698	4.666395
H	-0.982052	-4.431978	0.731100
H	6.336082	-3.553529	-0.703345
H	4.016071	-2.707782	-0.308238
H	-3.091418	-1.203973	1.796996
H	-4.523335	-3.240193	1.925914
C	-2.237713	3.540648	0.680162
C	-3.131084	2.945765	-0.290225
C	-3.884736	1.787256	-0.028382
H	-4.148190	1.547792	0.996939
H	-4.671314	1.553528	-0.740368
C	-3.105526	3.473470	-1.693613
H	-3.316876	4.549245	-1.713557
H	-2.100980	3.347682	-2.125501
H	-3.827197	2.965490	-2.336694
C	-1.539582	4.839171	0.338374
H	-0.959318	4.755985	-0.585417
H	-2.263817	5.651575	0.202498
H	-0.854267	5.130403	1.137560
C	-1.968308	2.899561	1.847776
H	-2.460784	1.988461	2.156634
H	-1.200756	3.276731	2.517455

^{2}IIIA$_{exo}$

Fe	0.242337	0.030101	0.202029
N	-0.414242	-1.817842	-0.164934
N	0.298676	-0.346873	2.167178
O	2.055733	-0.696430	0.090657
C	-0.716719	-2.582177	2.149925
C	-0.778122	-2.751108	0.775316
C	-1.078902	-3.764780	-1.213686
H	-1.307806	-4.445461	-2.022921
C	4.315707	-0.823731	-0.582228
C	-0.404693	-1.809061	-2.619429
C	-0.190002	-1.469791	2.790895
C	-0.590909	-2.421022	-1.389939
C	-1.209886	-3.963714	0.125495
H	-1.564597	-4.843893	0.645068
C	3.002066	-0.220647	-0.561091
H	2.844187	0.693990	-1.145500
C	0.843053	0.436476	3.161322
C	0.014216	-1.371428	4.214948
H	-0.288831	-2.123862	4.931536
C	0.667698	-0.198375	4.442228
H	1.004734	0.214730	5.384045
C	4.586747	-2.001740	0.143576
C	5.859541	-2.552640	0.101853
C	5.327858	-0.209596	-1.343775
H	5.109148	0.698560	-1.900104
C	6.601398	-0.767737	-1.379917
H	7.386639	-0.299360	-1.964730
C	6.864151	-1.936438	-0.658237
H	7.858700	-2.372384	-0.686523
N	1.022489	1.848521	0.551457
N	0.245538	0.403594	-1.768399
C	0.940899	2.762640	-1.730183
C	1.246698	2.841280	-0.378072
C	2.105634	3.627089	1.552321
H	2.574050	4.213564	2.332042
C	1.455258	1.666143	2.965281
C	0.492901	1.618225	-2.374763
C	1.536876	2.320228	1.743781
C	1.911829	3.956751	0.242790
H	2.194183	4.866507	-0.271161
C	-0.047189	-0.478867	-2.790756
C	0.326973	1.499305	-3.799513
H	0.461808	2.311193	-4.502628
C	0.008983	0.198491	-4.057600
H	-0.178946	-0.271697	-5.014144
O	-1.648497	0.661098	0.407414
C	-3.403124	-0.884691	-0.382185
C	-2.654924	0.404140	-0.633330
H	-2.080103	0.332473	-1.553247
C	-3.703984	-1.331802	0.908606
C	-4.487838	-2.470989	1.094751
C	-3.895612	-1.596534	-1.482289
H	-3.654053	-1.262066	-2.488775
C	-4.686264	-2.730624	-1.297185
H	-5.062692	-3.273070	-2.159889
C	-4.986346	-3.169737	-0.006431
H	-5.600871	-4.053314	0.140130
H	-0.599958	-2.397074	-3.510048
H	1.133816	3.639094	-2.340052
H	1.870917	2.166952	3.833350
H	-1.057110	-3.404579	2.770353
H	6.079121	-3.459486	0.656687

H	3.792130	-2.458815	0.724468
H	-3.298369	-0.810033	1.769222
H	-4.709336	-2.812335	2.102252
C	-2.219953	3.042122	0.583189
C	-2.999945	2.947397	-0.506266
C	-3.631202	1.595169	-0.755888
H	-4.454246	1.446328	-0.042584
H	-4.082989	1.533383	-1.751363
C	-3.356678	4.050731	-1.461188
H	-2.900704	5.008764	-1.202705
H	-3.039337	3.788482	-2.479721
H	-4.444694	4.194968	-1.499845
C	-1.615670	4.285312	1.172537
H	-1.787253	5.168723	0.554082
H	-2.052680	4.489446	2.160256
H	-0.537390	4.177874	1.319887
C	-1.985451	1.754234	1.329832
H	-2.871445	1.423510	1.886672
H	-1.152321	1.816065	2.023274

^{4}IIIA$_{exo}$

Fe	0.251700	-0.001648	0.267157
N	-0.380291	-1.874433	0.041436
N	0.270632	-0.231512	2.248946
O	2.340756	-0.766297	0.200002
C	-0.695730	-2.486435	2.401640
C	-0.747439	-2.749093	1.043130
C	-1.053748	-3.883361	-0.876168
H	-1.288611	-4.614158	-1.638449
C	4.592744	-0.734691	-0.561381
C	-0.385866	-2.034247	-2.411750
C	-0.198902	-1.317649	2.957976
C	-0.567072	-2.557035	-1.142289
C	-1.177991	-3.997305	0.475075
H	-1.530746	-4.842959	1.050142
C	3.222998	-0.252498	-0.496670
H	2.974274	0.626574	-1.113724
C	0.757438	0.653319	3.190580
C	-0.031689	-1.096278	4.367997
H	-0.325774	-1.797122	5.138400
C	0.573270	0.117378	4.511473
H	0.870902	0.617933	5.423693
C	4.999066	-1.855383	0.188138
C	6.313602	-2.294856	0.107184
C	5.512357	-0.063794	-1.386715
H	5.189526	0.801038	-1.961725
C	6.828828	-0.508409	-1.463456
H	7.543249	0.005677	-2.098813
C	7.226279	-1.622044	-0.717187
H	8.253700	-1.969814	-0.776841
N	0.979924	1.857852	0.480277
N	0.264030	0.236480	-1.723066
C	0.934674	2.600091	-1.865624
C	1.198723	2.788949	-0.516945
C	1.941072	3.757126	1.376707
H	2.351351	4.423567	2.124258
C	1.324331	1.888551	2.916698
C	0.512407	1.403154	-2.423023
C	1.422246	2.446562	1.650494
C	1.789847	3.974562	0.037237
H	2.055389	4.853562	-0.535678
C	-0.030865	-0.720310	-2.676751
C	0.351245	1.175087	-3.831662
H	0.486732	1.930388	-4.594971
C	0.029486	-0.141864	-3.989432
H	-0.158546	-0.683036	-4.907630
O	-1.876075	0.691692	0.405725
C	-3.467293	-0.935868	-0.484364
C	-2.803910	0.409705	-0.679181
H	-2.189892	0.395062	-1.582008
C	-3.769534	-1.422992	0.791381
C	-4.469248	-2.620956	0.940992
C	-3.869398	-1.668628	-1.606799
H	-3.626079	-1.300978	-2.601460
C	-4.574408	-2.863470	-1.459173
H	-4.883662	-3.421099	-2.338916
C	-4.878616	-3.341532	-0.182852
H	-5.427680	-4.271421	-0.065162
H	-0.585527	-2.683431	-3.257419
H	1.121821	3.431196	-2.537253
H	1.688462	2.473395	3.754637
H	-1.027343	-3.266390	3.078590
H	6.636116	-3.158317	0.680927
H	4.272133	-2.357632	0.818488
H	-3.435374	-0.873581	1.665834
H	-4.696252	-2.991083	1.937206
C	-2.462973	3.078056	0.424359
C	-3.262277	2.915612	-0.642935

C	-3.851099	1.533539	-0.824225
H	-4.645508	1.368197	-0.082065
H	-4.324492	1.415922	-1.804549
C	-3.653655	3.962224	-1.647422
H	-3.219330	4.942864	-1.441070
H	-3.340098	3.659630	-2.656075
H	-4.745224	4.077734	-1.681937
C	-1.838065	4.353015	0.916880
H	-2.102388	5.216787	0.303256
H	-2.164656	4.568988	1.943703
H	-0.745719	4.276658	0.943354
C	-2.202155	1.837143	1.242307
H	-3.069533	1.560825	1.858756
H	-1.345022	1.953774	1.903970

^{6}IIIA$_{exo}$

Fe	0.224423	0.000704	0.239713
N	-0.430694	-1.891086	-0.139234
N	0.282149	-0.379137	2.249496
O	2.259679	-0.782913	0.128119
C	-0.658869	-2.654805	2.191750
C	-0.770295	-2.827536	0.811979
C	-1.177059	-3.791874	-1.190714
H	-1.458693	-4.459465	-1.994302
C	4.523547	-0.791609	-0.586254
C	-0.494264	-1.810658	-2.596059
C	-0.158235	-1.533423	2.857671
C	-0.669142	-2.454737	-1.371213
C	-1.245495	-4.018670	0.154204
H	-1.592416	-4.908407	0.662807
C	3.173703	-0.262668	-0.525547
H	2.966671	0.654285	-1.098680
C	0.772825	0.443459	3.238043
C	0.034257	-1.420811	4.281703
H	-0.228575	-2.183351	5.003562
C	0.615339	-0.205583	4.515799
H	0.915013	0.217451	5.466009
C	4.872165	-1.963955	0.112746
C	6.171403	-2.446647	0.036149
C	5.485118	-0.113434	-1.356989
H	5.206334	0.790770	-1.892958
C	6.785874	-0.602051	-1.429257
H	7.532606	-0.083251	-2.022109
C	7.125789	-1.766400	-0.733404
H	8.141152	-2.148451	-0.789679
N	1.033521	1.877617	0.583482
N	0.273761	0.389456	-1.789068
C	1.072557	2.718771	-1.731861
C	1.312468	2.841539	-0.360956
C	2.030551	3.675424	1.614013
H	2.444184	4.288865	2.404149
C	1.330857	1.705849	3.026110
C	0.597924	1.585308	-2.394523
C	1.462309	2.364873	1.800837
C	1.931588	3.972587	0.281969
H	2.250951	4.874315	-0.224660
C	-0.073571	-0.492876	-2.792060
C	0.435336	1.455365	-3.820018
H	0.620384	2.244028	-4.538115
C	0.027750	0.173065	-4.065271
H	-0.186530	-0.285991	-5.021852
O	-1.825421	0.692807	0.432142
C	-3.495756	-0.877457	-0.432005
C	-2.788919	0.443715	-0.636415
H	-2.190415	0.411269	-1.547974
C	-3.745646	-1.388634	0.845322
C	-4.482026	-2.563861	1.001644
C	-3.987587	-1.561690	-1.549897
H	-3.783757	-1.176561	-2.546761
C	-4.730198	-2.731931	-1.394374
H	-5.108719	-3.252232	-2.269798
C	-4.980795	-3.235383	-0.116020
H	-5.557769	-4.147410	0.007353
H	-0.737206	-2.382059	-3.486421
H	1.318272	3.577649	-2.348775
H	1.692430	2.233040	3.903499
H	-0.967856	-3.490678	2.811635
H	6.450042	-3.349591	0.570670
H	4.113828	-2.470860	0.700873
H	-3.339858	-0.881309	1.714721
H	-4.666262	-2.954500	1.998751
C	-2.370343	3.082373	0.541861
C	-3.169083	2.968120	-0.532322
C	-3.795543	1.608092	-0.752510
H	-4.597429	1.449078	-0.017377
H	-4.269330	1.531036	-1.736541
C	-3.531725	4.051597	-1.507960
H	-3.078401	5.016221	-1.269943

H	-3.216597	3.771637	-2.522561
H	-4.620344	4.190899	-1.546152
C	-1.721514	4.328509	1.075137
H	-1.959155	5.213264	0.480985
H	-2.056288	4.525203	2.103170
H	-0.632058	4.226679	1.112250
C	-2.136826	1.811460	1.319226
H	-3.012710	1.517795	1.913664
H	-1.282546	1.884741	1.989982

^{2}VIIA$_{exo}$

Fe	0.134622	-0.058696	0.145159
N	-0.492836	-1.405399	-1.218325
N	-0.105242	-1.408032	1.620257
O	1.990018	-0.863726	-0.109998
C	-1.055097	-3.245882	0.301973
C	-0.967008	-2.665510	-0.957527
C	-1.100289	-2.440899	-3.193201
H	-1.255022	-2.571006	-4.256442
C	4.341335	-0.931824	-0.390756
C	-0.179788	-0.107446	-3.275615
C	-0.654657	-2.658837	1.493665
C	-0.561951	-1.251734	-2.579993
C	-1.356028	-3.317980	-2.183511
H	-1.769473	-4.316039	-2.244503
C	3.061880	-0.260883	-0.242563
H	3.063803	0.838404	-0.250060
C	0.108192	-1.221583	2.956838
C	-0.776713	-3.283001	2.789498
H	-1.177669	-4.273611	2.960391
C	-0.302550	-2.389012	3.700266
H	-0.229745	-2.493282	4.775039
C	4.424555	-2.337582	-0.386880
C	5.661955	-2.951467	-0.526760
C	5.503836	-0.154308	-0.535464
H	5.430228	0.930684	-0.536772
C	6.741290	-0.775494	-0.675273
H	7.642034	-0.180043	-0.786889
C	6.817834	-2.171475	-0.670664
H	7.783485	-2.657032	-0.779467
N	0.908766	1.254097	1.487198
N	0.518177	1.253197	-1.349179
C	1.424113	3.119574	-0.024753
C	1.370978	2.512222	1.239130
C	1.560288	2.258327	3.468597
H	1.748092	2.371625	4.528501
C	0.638681	-0.068276	3.536358
C	1.069826	2.501715	-1.229145
C	1.010078	1.073883	2.852809
C	1.789361	3.151029	2.466534
H	2.205117	4.148543	2.534876
C	0.329018	1.049138	-2.704502
C	1.224560	3.107661	-2.528150
H	1.644171	4.090194	-2.705444
C	0.760779	2.207753	-3.442225
H	0.723063	2.301873	-4.519923
O	-1.520677	0.571164	0.504147
C	-3.485007	-0.632508	-0.230075
C	-2.557176	0.550741	-0.420152
H	-2.190467	0.555710	-1.453663
C	-3.684052	-1.169370	1.046632
C	-4.524049	-2.268752	1.222507
C	-4.142035	-1.201537	-1.325632
H	-3.974147	-0.799389	-2.322563
C	-4.982126	-2.301613	-1.151324
H	-5.479434	-2.744429	-2.009864
C	-5.175052	-2.837310	0.124647
H	-5.828492	-3.694413	0.261663
H	-0.278158	-0.126492	-4.356666
H	1.925110	4.080256	-0.090941
H	0.762659	-0.067468	4.614851
H	-1.478087	-4.243618	0.357990
H	5.736099	-4.034629	-0.524412
H	3.514533	-2.917939	-0.272909
H	-3.150611	-0.737301	1.887416
H	-4.666034	-2.685593	2.215968
C	-1.728903	3.619789	0.550836
C	-2.466907	3.030221	-0.497965
C	-3.388457	1.903305	-0.236170
H	-3.793842	1.896025	0.776231
H	-4.211632	1.879979	-0.956837
C	-2.355267	3.412745	-1.938676
H	-1.525848	4.070359	-2.189529
H	-2.261945	2.508020	-2.552699
H	-3.293412	3.896446	-2.247084
C	-1.962287	3.215513	1.993813
H	-2.981588	3.461813	2.313884
H	-1.809634	2.141209	2.109698

H	-1.266782	3.735149	2.657361
C	-0.741710	4.544512	0.270330
H	-0.542114	4.915331	-0.725638
H	-0.219897	5.047145	1.076713

$^{2}VA_{endo}$

Fe	1.475134	0.282184	0.192460
N	0.961588	1.055183	1.949636
N	1.304864	2.083331	-0.628382
C	0.581757	3.401092	1.313572
C	0.605478	2.365339	2.231522
C	0.239539	1.240750	4.144327
H	-0.004177	0.936683	5.153913
C	1.022626	-0.981363	3.326537
C	0.879461	3.257530	-0.029866
C	0.737797	0.359840	3.126551
C	0.176883	2.486709	3.595115
H	-0.141418	3.411977	4.057058
C	1.428784	2.386921	-1.972136
C	0.743246	4.299688	-1.007224
H	0.433438	5.312159	-0.784368
C	1.067038	3.755913	-2.213916
H	1.087534	4.233346	-3.184859
N	2.417885	-0.359639	-1.446985
N	2.018195	-1.407291	1.122260
C	3.153487	-2.584892	-0.710658
C	3.016281	-1.588381	-1.661163
C	3.029611	-0.582948	-3.671037
H	3.154968	-0.315552	-4.712134
C	1.913683	1.524649	-2.940675
C	2.664235	-2.505646	0.581616
C	2.408885	0.257228	-2.683524
C	3.421321	-1.720225	-3.033107
H	3.929950	-2.583273	-3.442236
C	1.658910	-1.793227	2.400577
C	2.722893	-3.576283	1.537193
H	3.193401	-4.533921	1.356816
C	2.085558	-3.141000	2.659551
H	1.930849	-3.664867	3.593835
O	-0.218440	-0.454137	-0.418355
C	-2.101859	-1.875836	-0.444715
C	-0.992972	-1.214627	0.200610
H	-0.799400	-1.431445	1.257580
C	-2.456727	-1.562248	-1.772142
C	-3.518472	-2.228429	-2.370149
C	-2.815454	-2.856246	0.270190
H	-2.539396	-3.084208	1.296673
C	-3.866112	-3.530501	-0.342139
H	-4.415518	-4.293074	0.200605
C	-4.216504	-3.214728	-1.659441
H	-5.040842	-3.737874	-2.135572
H	0.797037	-1.405079	4.299633
H	3.642351	-3.505867	-1.010540
H	1.965033	1.889113	-3.961320
H	0.266614	4.378998	1.661530
H	-1.903960	-0.792618	-2.298313
H	-3.805089	-1.989479	-3.389552
C	-3.459688	1.617168	-0.611569
C	-3.540272	0.860154	0.668819
C	-2.614910	0.993173	1.636638
H	-1.800561	1.702688	1.554989
H	-2.681284	0.433191	2.565760
C	-4.724190	-0.059773	0.852475
H	-5.661967	0.507755	0.887960
H	-4.811213	-0.767016	0.019747
H	-4.644619	-0.631176	1.781442
C	-4.764643	1.962938	-1.293297
H	-5.305958	1.058754	-1.598983
H	-5.431366	2.520017	-0.623431
H	-4.595057	2.570541	-2.186429
C	-2.281533	1.958289	-1.160316
H	-1.332829	1.683475	-0.716235
H	-2.235033	2.525803	-2.085726

$^{4}VA_{endo}$

Fe	1.531143	-0.243744	-0.180151
N	0.978790	-1.593350	-1.544788
N	1.626823	-1.694577	1.185720
C	0.771289	-3.593391	-0.123078
C	0.676198	-2.930166	-1.332932
C	0.235859	-2.570401	-3.508947
H	-0.041381	-2.651217	-4.551788
C	0.855945	-0.161646	-3.545401
C	1.212021	-3.009199	1.050055
C	0.705185	-1.368133	-2.885588
C	0.220880	-3.538361	-2.548552
H	-0.072706	-4.575702	-2.640925
C	1.986595	-1.562698	2.516715

C	1.304729	-3.695339	2.305750
H	1.041944	-4.734246	2.456055
C	1.780825	-2.798245	3.215179
H	1.991283	-2.949737	4.265784
N	2.487277	0.980728	1.077062
N	1.759136	1.105033	-1.638806
C	2.738502	2.964776	-0.356978
C	2.865923	2.292117	0.844980
C	3.321344	1.929975	3.017060
H	3.624029	2.001958	4.053490
C	2.505746	-0.420375	3.098963
C	2.221625	2.406019	-1.512489
C	2.752931	0.757371	2.417137
C	3.393540	2.880028	2.042658
H	3.766754	3.893257	2.114172
C	1.358842	0.987437	-2.961194
C	2.098704	3.102177	-2.759439
H	2.398704	4.129643	-2.919049
C	1.562140	2.224918	-3.655803
H	1.333314	2.384902	-4.701400
O	-0.318040	0.473797	0.436893
C	-2.239672	1.856946	0.430952
C	-1.092611	1.224587	-0.184009
H	-0.881274	1.456651	-1.237191
C	-2.620970	1.527297	1.746414
C	-3.722502	2.153154	2.316011
C	-2.966991	2.813092	-0.301021
H	-2.670349	3.053265	-1.319146
C	-4.059741	3.446629	0.281322
H	-4.621296	4.189750	-0.275912
C	-4.435946	3.114943	1.587425
H	-5.292494	3.605939	2.040480
H	0.590074	-0.122207	-4.596230
H	3.069958	3.996725	-0.397800
H	2.754033	-0.457076	4.154132
H	0.482163	-4.638103	-0.093366
H	-2.055822	0.775127	2.284790
H	-4.029186	1.900996	3.326452
C	-3.459329	-1.713299	0.586917
C	-3.555064	-0.968677	-0.699728
C	-2.621237	-1.084067	-1.660985
H	-1.786849	-1.768497	-1.568624
H	-2.698978	-0.531860	-2.594156
C	-4.764975	-0.085679	-0.897642
H	-5.685118	-0.681281	-0.936323
H	-4.879337	0.624482	-0.070773
H	-4.695329	0.481833	-1.829829
C	-4.757574	-2.104156	1.256910
H	-5.336704	-1.218915	1.548492
H	-5.394586	-2.691743	0.584110
H	-4.575259	-2.697227	2.157360
C	-2.274768	-2.004608	1.150094
H	-1.333783	-1.691744	0.715203
H	-2.216038	-2.561023	2.081931

^{6}VA$_{endo}$

Fe	-1.395536	-0.123547	0.169771
N	-0.986121	-1.474176	1.678027
N	-1.785124	-1.724241	-1.066735
C	-0.989562	-3.559777	0.365101
C	-0.782824	-2.835717	1.535213
C	-0.243447	-2.363166	3.674507
H	0.061776	-2.402681	4.712075
C	-0.752025	0.092164	3.565885
C	-1.458655	-3.048052	-0.840292
C	-0.665914	-1.171971	2.989767
C	-0.317491	-3.390635	2.777215
H	-0.083394	-4.435242	2.935744
C	-2.229949	-1.653796	-2.373503
C	-1.683799	-3.816207	-2.034893
H	-1.508716	-4.880365	-2.125348
C	-2.157472	-2.954530	-2.982847
H	-2.448216	-3.174980	-4.001770
N	-2.507910	1.056208	-1.102428
N	-1.652054	1.316148	1.630035
C	-2.609324	3.113835	0.244879
C	-2.817035	2.391052	-0.926084
C	-3.401811	1.909563	-3.050932
H	-3.759134	1.935642	-4.072180
C	-2.713030	-0.505237	-2.994076
C	-2.076971	2.617599	1.431476
C	-2.857620	0.746027	-2.402715
C	-3.378750	2.924772	-2.138605
H	-3.713171	3.946223	-2.265572
C	-1.224214	1.244248	2.943393
C	-1.896507	3.376470	2.639451
H	-2.155897	4.420822	2.755706
C	-1.367870	2.529679	3.572187

H	-1.110566	2.744944	4.601277
O	0.415837	0.496016	-0.474865
C	2.420974	1.748601	-0.525159
C	1.262787	1.192123	0.129045
H	1.094278	1.432201	1.186692
C	2.734704	1.394036	-1.853276
C	3.850762	1.951790	-2.462912
C	3.232665	2.657889	0.179674
H	2.987724	2.915860	1.207004
C	4.339842	3.222689	-0.443220
H	4.965778	3.929975	0.091410
C	4.646726	2.868869	-1.761994
H	5.514494	3.306825	-2.246836
H	-0.458750	0.179389	4.607337
H	-2.901719	4.159117	0.236241
H	-3.032192	-0.601415	-4.027032
H	-0.774596	-4.622877	0.399647
H	2.106448	0.676711	-2.369129
H	4.105529	1.681692	-3.483003
C	3.257970	-1.933914	-0.636138
C	3.504985	-1.148847	0.605141
C	2.630249	-1.130270	1.628792
H	1.734645	-1.739197	1.631564
H	2.825406	-0.548802	2.526185
C	4.800604	-0.376267	0.686055
H	5.662850	-1.053861	0.684367
H	4.920252	0.292822	-0.173828
H	4.850372	0.224986	1.597906
C	4.467606	-2.431914	-1.395903
H	5.076792	-1.600156	-1.771022
H	5.119825	-3.038880	-0.755780
H	4.170390	-3.042645	-2.252710
C	2.015607	-2.178911	-1.086350
H	1.131957	-1.809175	-0.583256
H	1.846259	-2.763587	-1.986425

$^{2}\mathbf{TS2A}_{endo}$

Fe	-0.968518	0.873522	-0.357433
N	-1.412549	1.501500	1.491898
N	-2.670885	-0.179366	-0.392654
C	-3.335918	0.044521	1.963138
C	-2.378281	0.976106	2.331402
C	-1.277748	2.484523	3.587450
H	-0.925023	3.143808	4.369835
C	0.351472	3.180785	1.827544
C	-3.489104	-0.466332	0.684576
C	-0.730446	2.429332	2.260061
C	-2.293337	1.577901	3.634612
H	-2.948916	1.342327	4.462924
C	-3.190080	-0.909362	-1.446218
C	-4.533293	-1.376309	0.296519
H	-5.307470	-1.740718	0.959275
C	-4.351594	-1.645519	-1.026216
H	-4.945967	-2.276325	-1.674405
N	-0.757934	0.542615	-2.316673
N	0.477036	2.258067	-0.444834
C	1.321887	1.791346	-2.702685
C	0.311500	0.930798	-3.098008
C	-0.920462	-0.401857	-4.427446
H	-1.335422	-0.982335	-5.241077
C	-2.655779	-0.959413	-2.722835
C	1.382218	2.426345	-1.473591
C	-1.510962	-0.290730	-3.120354
C	0.215137	0.347726	-4.410691
H	0.925950	0.514563	-5.209549
C	0.918667	3.090963	0.567232
C	2.387630	3.388367	-1.107985
H	3.200367	3.699727	-1.751085
C	2.094695	3.807669	0.153319
H	2.617385	4.533165	0.762883
O	0.178150	-0.512676	0.087380
C	2.522885	-0.609877	0.524424
C	1.132861	-0.337893	0.977749
H	1.041916	0.535507	1.629444
C	2.751593	-1.273208	-0.689099
C	4.057816	-1.517572	-1.111705
C	3.607677	-0.187288	1.303279
H	3.429160	0.338309	2.239053
C	4.912243	-0.432085	0.877575
H	5.750825	-0.098213	1.481478
C	5.138920	-1.100864	-0.328832
H	6.155476	-1.290302	-0.660927
H	0.798339	3.873101	2.533652
H	2.103874	2.013001	-3.421431
H	-3.159300	-1.580232	-3.456726
H	-4.045862	-0.272432	2.720219
H	1.900575	-1.568617	-1.293934
H	4.234304	-2.025606	-2.055554

C	0.126101	-3.440558	0.949949
C	0.978138	-2.784087	1.921704
C	0.685562	-1.514882	2.449200
H	-0.342661	-1.175328	2.525047
H	1.322498	-1.165454	3.257952
C	2.327361	-3.372003	2.194315
H	2.249608	-4.434069	2.453213
H	2.943029	-3.312182	1.284789
H	2.854694	-2.847886	2.992369
C	0.645751	-4.657342	0.214678
H	1.586594	-4.440750	-0.303930
H	0.837160	-5.491877	0.899272
H	-0.077926	-4.999488	-0.529023
C	-1.090858	-2.920774	0.649874
H	-1.530344	-2.091319	1.183568
H	-1.699784	-3.340796	-0.144882

^{4}TS2A$_{endo}$

Fe	0.927135	0.733465	0.609684
N	1.466518	1.960555	-0.885369
N	2.684972	-0.217647	0.435052
C	3.518156	0.826264	-1.632306
C	2.535415	1.795102	-1.748255
C	1.405142	3.568325	-2.548903
H	1.053988	4.424775	-3.109656
C	-0.404625	3.557412	-0.831650
C	3.592793	-0.096569	-0.601530
C	0.764337	3.047555	-1.373998
C	2.499025	2.789606	-2.783593
H	3.231857	2.877523	-3.575159
C	3.158410	-1.252161	1.220951
C	4.641188	-1.067586	-0.462086
H	5.476343	-1.170890	-1.142617
C	4.375070	-1.780024	0.670032
H	4.947301	-2.587243	1.108607
N	0.619475	-0.210183	2.352322
N	-0.597032	1.969394	1.036459
C	-1.507254	0.832437	3.017198
C	-0.485115	-0.088235	3.173799
C	0.722114	-1.765093	4.063152
H	1.114660	-2.571694	4.668571
C	2.548749	-1.734205	2.367064
C	-1.547283	1.796990	2.025903
C	1.363257	-1.244183	2.887395
C	-0.425992	-1.052756	4.237059
H	-1.170170	-1.151465	5.016593
C	-1.036151	3.047123	0.289816
C	-2.587163	2.778672	1.896750
H	-3.442829	2.854760	2.554666
C	-2.268234	3.556532	0.824827
H	-2.807690	4.403429	0.421278
O	-0.127675	-0.495934	-0.484165
C	-2.490981	-0.411844	-0.866734
C	-1.088097	-0.123897	-1.296014
H	-0.971305	0.873714	-1.743147
C	-2.731206	-1.285839	0.201585
C	-4.038788	-1.534111	0.618922
C	-3.567768	0.213500	-1.506910
H	-3.380898	0.903195	-2.327733
C	-4.874157	-0.036278	-1.089131
H	-5.705543	0.454768	-1.586499
C	-5.111660	-0.912849	-0.026492
H	-6.129302	-1.105899	0.300440
H	-0.856776	4.413602	-1.320984
H	-2.321544	0.810151	3.733280
H	3.031211	-2.552658	2.890843
H	4.301852	0.809862	-2.382276
H	-1.885175	-1.741304	0.706477
H	-4.221604	-2.207043	1.452128
C	0.058875	-3.087692	-1.905667
C	-0.924950	-2.352675	-2.662055
C	-0.768600	-0.980536	-2.949242
H	0.231531	-0.582332	-3.093955
H	-1.506083	-0.540161	-3.615920
C	-2.256149	-2.987247	-2.919213
H	-2.144566	-3.989208	-3.347973
H	-2.792424	-3.107358	-1.965595
H	-2.880835	-2.384050	-3.579636
C	-0.274448	-4.477058	-1.406008
H	-1.185261	-4.477019	-0.796311
H	-0.441105	-5.173121	-2.236449
H	0.539216	-4.878169	-0.796857
C	1.228854	-2.490244	-1.548281
H	1.558832	-1.536098	-1.932620
H	1.918109	-2.978135	-0.865546

^{6}TS2A$_{endo}$

Fe	1.080330	0.588337	-0.203050

N	1.400045	-0.231713	-2.090246
N	2.646187	-0.593271	0.479928
C	2.950289	-2.046629	-1.484020
C	2.115392	-1.378905	-2.377078
C	1.038525	-0.839818	-4.286081
H	0.657584	-0.793497	-5.298197
C	-0.050068	1.253926	-3.414077
C	3.211947	-1.673511	-0.167291
C	0.747201	0.121571	-3.254543
C	1.884907	-1.765006	-3.745060
H	2.331725	-2.624891	-4.227308
C	3.161455	-0.592797	1.761161
C	4.090350	-2.374402	0.732492
H	4.664840	-3.254508	0.473607
C	4.056581	-1.708848	1.924798
H	4.601396	-1.934752	2.832417
N	1.394101	1.814907	1.432888
N	0.188835	2.202752	-1.157168
C	-0.038920	3.704634	0.780457
C	0.747715	3.004515	1.691758
C	1.840785	2.482439	3.595059
H	2.263882	2.464214	4.591097
C	2.879444	0.356901	2.740689
C	-0.290396	3.340179	-0.539243
C	2.072982	1.480840	2.585323
C	1.022747	3.424153	3.042646
H	0.642776	4.330252	3.496468
C	-0.293598	2.229088	-2.449251
C	-1.089035	4.098248	-1.468185
H	-1.575455	5.037517	-1.238966
C	-1.092397	3.411940	-2.647262
H	-1.580891	3.678367	-3.575661
O	-0.515668	-0.350120	0.311478
C	-2.900437	-0.200599	0.169671
C	-1.578540	-0.490197	-0.457505
H	-1.480853	-0.092215	-1.475406
C	-3.016016	-0.109160	1.562993
C	-4.258541	0.146161	2.143141
C	-4.033778	-0.029051	-0.634918
H	-3.942870	-0.091342	-1.717501
C	-5.274826	0.225841	-0.052854
H	-6.150010	0.362625	-0.681146
C	-5.389368	0.310494	1.337585
H	-6.355560	0.510932	1.791222
H	-0.497233	1.406388	-4.391499
H	-0.485906	4.631042	1.127107
H	3.359045	0.224712	3.705546
H	3.461335	-2.929453	-1.855435
H	-2.125201	-0.221774	2.172626
H	-4.344724	0.224895	3.223163
C	-1.010068	-3.243407	1.146292
C	-1.919617	-3.031809	0.041924
C	-1.558962	-2.274533	-1.092155
H	-0.524791	-2.255154	-1.423331
H	-2.272233	-2.272258	-1.912847
C	-3.356144	-3.410006	0.216586
H	-3.453828	-4.430743	0.602198
H	-3.818456	-2.744091	0.960847
H	-3.923068	-3.321317	-0.711069
C	-1.548974	-3.759795	2.462906
H	-2.362522	-3.128787	2.837755
H	-1.945638	-4.776928	2.363410
H	-0.762695	-3.784779	3.221208
C	0.300086	-2.908792	1.017537
H	0.745271	-2.618327	0.077661
H	0.982333	-2.962642	1.860734

^{2}VIA$_{endo}$

Fe	0.864690	0.481874	0.755348
N	1.128335	2.288711	-0.048972
N	2.761460	0.029279	0.293838
C	3.259444	1.908199	-1.211394
C	2.134152	2.662024	-0.923412
C	0.736200	4.422742	-0.863855
H	0.240118	5.375517	-0.994923
C	-0.925472	3.412369	0.699258
C	3.567193	0.699582	-0.610607
C	0.261435	3.368538	-0.014382
C	1.890209	3.979406	-1.438380
H	2.539315	4.496487	-2.132916
C	3.479155	-1.102268	0.643947
C	4.798534	-0.012448	-0.813489
H	5.596354	0.316465	-1.466475
C	4.750068	-1.121193	-0.023964
H	5.497492	-1.893611	0.101419
N	0.795943	-1.154855	1.896382
N	-0.811958	1.129014	1.603054
C	-1.484825	-0.919775	2.778657

C	-0.309082	-1.622953	2.585427
C	1.237108	-3.242385	2.805236
H	1.798421	-4.132397	3.057922
C	3.014889	-2.117041	1.463330
C	-1.701383	0.377418	2.348265
C	1.750306	-2.150179	2.026309
C	-0.043377	-2.921578	3.140407
H	-0.749653	-3.492159	3.729280
C	-1.428032	2.360830	1.443370
C	-2.873903	1.144607	2.659966
H	-3.713735	0.773698	3.232030
C	-2.698009	2.378276	2.111277
H	-3.364648	3.230071	2.137824
O	0.077619	-0.391062	-0.825873
C	-2.341374	-0.054653	-1.005409
C	-0.986298	0.287221	-1.582578
H	-0.789477	1.352773	-1.454060
C	-2.623634	-1.306742	-0.448640
C	-3.908282	-1.598250	0.011060
C	-3.359841	0.902257	-1.086526
H	-3.144015	1.883794	-1.503206
C	-4.646029	0.608806	-0.634527
H	-5.427620	1.359814	-0.704414
C	-4.923496	-0.644475	-0.084540
H	-5.924011	-0.874540	0.270210
H	-1.513452	4.322637	0.647299
H	-2.274658	-1.401178	3.345224
H	3.673924	-2.958504	1.649599
H	3.978304	2.321497	-1.911156
H	-1.834709	-2.044334	-0.358721
H	-4.115484	-2.573079	0.443814
C	-0.042465	-2.276217	-2.437225
C	-0.862422	-1.584028	-3.245998
C	-0.847086	-0.080907	-3.067694
H	0.088011	0.352611	-3.451704
H	-1.667291	0.399295	-3.609454
C	-1.839207	-2.147196	-4.235710
H	-1.768537	-3.231037	-4.344932
H	-2.864950	-1.901680	-3.929108
H	-1.687647	-1.695500	-5.224650
C	0.118654	-3.769826	-2.349967
H	-0.184487	-4.134000	-1.358310
H	-0.472480	-4.301213	-3.097762
H	1.168044	-4.063207	-2.486840
C	0.818626	-1.479633	-1.496147
H	1.667782	-1.005602	-2.001369
H	1.199192	-2.091067	-0.679177

^{4}VIA$_{endo}$

Fe	0.934754	0.490743	0.754993
N	1.175376	2.276849	-0.107577
N	2.794980	-0.000529	0.214037
C	3.315008	1.899367	-1.264103
C	2.192102	2.652526	-0.971506
C	0.773905	4.398148	-0.944368
H	0.269245	5.343872	-1.092717
C	-0.886769	3.401903	0.623676
C	3.604349	0.676081	-0.686361
C	0.298716	3.350286	-0.089249
C	1.941567	3.962037	-1.498085
H	2.593119	4.478320	-2.190891
C	3.495209	-1.152985	0.536158
C	4.811851	-0.059210	-0.922929
H	5.605845	0.263696	-1.583486
C	4.748673	-1.186756	-0.158382
H	5.478736	-1.980195	-0.066016
N	0.865088	-1.142270	1.900491
N	-0.733332	1.152466	1.610766
C	-1.364097	-0.848691	2.897893
C	-0.206562	-1.569176	2.668011
C	1.315469	-3.218185	2.821781
H	1.868781	-4.118734	3.054046
C	3.041642	-2.160126	1.369714
C	-1.593699	0.427823	2.419915
C	1.806957	-2.154442	1.994548
C	0.065643	-2.859306	3.231778
H	-0.615924	-3.403272	3.872554
C	-1.365236	2.370016	1.409097
C	-2.764840	1.196900	2.720426
H	-3.587369	0.845266	3.328479
C	-2.618916	2.402939	2.103530
H	-3.297445	3.245652	2.100956
O	0.004235	-0.419320	-0.888296
C	-2.404809	-0.024535	-0.960571
C	-1.067776	0.260512	-1.611079
H	-0.840210	1.326534	-1.526836
C	-2.674457	-1.234071	-0.312303
C	-3.941959	-1.481344	0.217232

C	-3.418518	0.935810	-1.062806
H	-3.212793	1.884431	-1.554538
C	-4.686804	0.687539	-0.538966
H	-5.464879	1.440483	-0.626781
C	-4.951977	-0.524555	0.102473
H	-5.938920	-0.720194	0.511857
H	-1.484676	4.303776	0.550030
H	-2.130381	-1.302414	3.516588
H	3.688969	-3.015107	1.531882
H	4.036632	2.314478	-1.959415
H	-1.887296	-1.972148	-0.209589
H	-4.139774	-2.424546	0.719190
C	-0.200364	-2.336231	-2.444941
C	-1.064608	-1.661330	-3.221380
C	-1.025589	-0.153460	-3.092394
H	-0.110688	0.255683	-3.545559
H	-1.871819	0.318507	-3.600317
C	-2.105409	-2.244916	-4.131461
H	-2.050763	-3.332433	-4.210402
H	-3.107923	-1.980226	-3.768865
H	-2.013303	-1.826160	-5.141989
C	-0.036735	-3.827535	-2.331414
H	-0.282975	-4.168308	-1.315846
H	-0.669191	-4.375905	-3.031781
H	1.003174	-4.126026	-2.520715
C	0.708013	-1.512418	-1.571667
H	1.530846	-1.055773	-2.136746
H	1.138812	-2.105306	-0.764187

^{6}VIA$_{endo}$

Fe	0.887330	0.444424	0.635498
N	1.289318	2.288046	-0.203304
N	2.837213	-0.097634	0.232366
C	3.477426	1.791156	-1.216090
C	2.367623	2.604893	-1.008681
C	0.987601	4.386725	-1.113417
H	0.521495	5.341194	-1.320954
C	-0.773688	3.471272	0.430313
C	3.704965	0.548823	-0.631005
C	0.436561	3.375235	-0.252447
C	2.179378	3.910390	-1.581298
H	2.879505	4.398735	-2.246650
C	3.458594	-1.280833	0.590106
C	4.885213	-0.249579	-0.821259
H	5.723466	0.028695	-1.446768
C	4.734186	-1.377740	-0.065868
H	5.424168	-2.203927	0.047207
N	0.803129	-1.111842	1.980545
N	-0.728645	1.289997	1.571450
C	-1.382213	-0.610593	2.996249
C	-0.271090	-1.422925	2.793845
C	1.158435	-3.158676	2.983937
H	1.657767	-4.081650	3.248798
C	2.921920	-2.241540	1.443184
C	-1.591140	0.647748	2.441214
C	1.692942	-2.163827	2.092765
C	-0.054361	-2.701823	3.415160
H	-0.741475	-3.177341	4.103000
C	-1.310909	2.510762	1.280658
C	-2.735576	1.479727	2.692703
H	-3.564060	1.209091	3.333649
C	-2.561481	2.630444	1.979038
H	-3.218700	3.488230	1.922524
O	-0.010188	-0.454616	-0.953567
C	-2.414231	-0.019085	-0.975182
C	-1.095794	0.217112	-1.678251
H	-0.841575	1.280736	-1.644063
C	-2.675650	-1.189271	-0.255317
C	-3.929155	-1.392100	0.323700
C	-3.418458	0.948811	-1.097416
H	-3.216570	1.868937	-1.641804
C	-4.673044	0.743067	-0.524987
H	-5.444784	1.500364	-0.628493
C	-4.931740	-0.430614	0.186590
H	-5.907947	-0.592081	0.634619
H	-1.343909	4.385076	0.296453
H	-2.152572	-0.986651	3.661683
H	3.522353	-3.126698	1.627899
H	4.251487	2.173144	-1.874205
H	-1.894553	-1.931540	-0.136081
H	-4.121393	-2.304051	0.882063
C	-0.287325	-2.438419	-2.411978
C	-1.159317	-1.779519	-3.194079
C	-1.090285	-0.268022	-3.137501
H	-0.179059	0.103139	-3.628679
H	-1.939497	0.195031	-3.648446
C	-2.236870	-2.381609	-4.047502
H	-2.207470	-3.472400	-4.077884

H	-3.222710	-2.077814	-3.670300
H	-2.163934	-2.011122	-5.078183
C	-0.152247	-3.926554	-2.234618
H	-0.378976	-4.215674	-1.198828
H	-0.815005	-4.490436	-2.893113
H	0.875326	-4.256664	-2.437375
C	0.661144	-1.600168	-1.599423
H	1.479886	-1.179927	-2.196249
H	1.094076	-2.162062	-0.770515

^{2}VA$_{exo}$

Fe	-1.310305	-0.429974	-0.360385
N	-1.932303	1.109375	-1.486023
N	-0.240627	-1.088950	-1.905666
O	0.047314	0.636734	0.551520
C	-0.383106	0.873424	-3.379650
C	-1.372672	1.541708	-2.675370
C	-2.928643	3.086268	-2.175277
H	-3.601899	3.933279	-2.159137
C	1.670744	2.340906	0.771273
C	-3.642904	2.076492	-0.010311
C	0.119219	-0.371590	-3.034857
C	-2.880144	2.066340	-1.165507
C	-1.983904	2.769561	-3.103900
H	-1.725521	3.299097	-4.011710
C	0.800679	1.475979	0.018070
H	0.804628	1.570685	-1.074619
C	0.412914	-2.303968	-2.025946
C	1.018544	-1.137176	-3.851093
H	1.438981	-0.796206	-4.788121
C	1.185110	-2.341758	-3.234176
H	1.775596	-3.188862	-3.557890
C	1.707114	2.291639	2.179546
C	2.578596	3.120551	2.869387
C	2.502502	3.232139	0.067273
H	2.466211	3.260822	-1.018731
C	3.373858	4.060027	0.765649
H	4.021591	4.747738	0.231426
C	3.412389	3.999929	2.162842
H	4.095634	4.644661	2.708173
N	-1.039552	-2.171683	0.550527
N	-2.699482	0.042423	0.992333
C	-2.254662	-1.725854	2.637256
C	-1.434383	-2.500222	1.834045
C	-0.259952	-4.279226	1.117448
H	0.241039	-5.231678	1.003938
C	0.357248	-3.333207	-1.102470
C	-2.868800	-0.554693	2.227112
C	-0.301619	-3.257235	0.110193
C	-0.943689	-3.801500	2.194823
H	-1.127685	-4.284355	3.145648
C	-3.536877	1.143493	1.006082
C	-3.828704	0.172915	3.012126
H	-4.139884	-0.103782	4.010964
C	-4.255086	1.216066	2.248309
H	-4.984800	1.977247	2.491312
H	-4.345959	2.892012	0.123155
H	-2.482821	-2.094808	3.631702
H	0.905703	-4.241777	-1.324621
H	-0.025935	1.324634	-4.299577
H	2.619285	3.088686	3.953537
H	1.054298	1.599088	2.700580
C	3.792935	-0.426682	0.285622
C	3.153914	-1.682231	0.770674
C	3.697916	-0.132518	-1.194834
H	4.248214	-0.872385	-1.789003
H	2.656943	-0.174352	-1.543422
H	4.107588	0.854524	-1.431068
C	3.518182	-2.959616	0.049816
H	3.229968	-2.918453	-1.007553
H	4.602496	-3.125196	0.078476
H	3.026307	-3.826033	0.501445
C	2.301167	-1.664272	1.804280
H	2.036317	-0.735259	2.299695
H	1.840180	-2.572308	2.182504
C	4.428043	0.394424	1.131774
H	4.524220	0.154413	2.186066
H	4.876887	1.325180	0.795221

^{4}VA$_{exo}$

Fe	-1.357602	-0.179274	-0.376375
N	-1.680791	1.528830	-1.374106
N	-0.462793	-0.923462	-1.999148
O	0.335231	0.593597	0.582709
C	-0.350889	1.099236	-3.399912
C	-1.135717	1.904029	-2.591775
C	-2.272318	3.709551	-1.879933
H	-2.737119	4.679935	-1.764288

C	2.192188	2.065245	0.701673
C	-3.045405	2.739199	0.278507
C	-0.057745	-0.226544	-3.128248
C	-2.373811	2.642711	-0.927123
C	-1.499200	3.255046	-2.907011
H	-1.202593	3.774544	-3.808879
C	1.199255	1.278376	0.008132
H	1.216669	1.301868	-1.091073
C	-0.025330	-2.223901	-2.198331
C	0.662914	-1.091164	-4.016181
H	1.083182	-0.782597	-4.964453
C	0.671927	-2.330337	-3.445911
H	1.105486	-3.244996	-3.828636
C	2.222439	2.115031	2.109177
C	3.209809	2.851782	2.746268
C	3.148268	2.765742	-0.055660
H	3.114867	2.720513	-1.141485
C	4.138350	3.499975	0.588977
H	4.883479	4.038624	0.012213
C	4.168389	3.538927	1.986947
H	4.942624	4.110181	2.491102
N	-1.407082	-2.005469	0.415285
N	-2.558413	0.464166	1.080333
C	-2.547160	-1.514408	2.540406
C	-1.892820	-2.360232	1.663029
C	-1.035005	-4.255858	0.810842
H	-0.705972	-5.269786	0.624826
C	-0.229004	-3.279341	-1.329064
C	-2.871125	-0.200561	2.253924
C	-0.867653	-3.168137	-0.108298
C	-1.658615	-3.752817	1.913849
H	-1.951935	-4.269770	2.818134
C	-3.118401	1.722982	1.214375
C	-3.626909	0.651729	3.125502
H	-3.994701	0.359359	4.100289
C	-3.787312	1.840445	2.478095
H	-4.310993	2.726040	2.813524
H	-3.539777	3.676688	0.509024
H	-2.863206	-1.919265	3.495664
H	0.151928	-4.253129	-1.615288
H	0.020465	1.520461	-4.328003
H	3.245789	2.894018	3.830363
H	1.474671	1.565591	2.671515
C	3.465479	-1.285103	0.414665
C	2.604695	-2.373666	0.957311
C	3.480644	-1.095684	-1.085926
H	3.910224	-1.965667	-1.598033
H	2.463255	-0.976053	-1.481178
H	4.072631	-0.220027	-1.370400
C	2.762668	-3.745839	0.343587
H	2.538136	-3.732561	-0.729939
H	3.795427	-4.102046	0.447079
H	2.098402	-4.473593	0.818799
C	1.738093	-2.133982	1.950873
H	1.613428	-1.137971	2.363829
H	1.119164	-2.922421	2.369584
C	4.198253	-0.516895	1.231397
H	4.210334	-0.684742	2.303896
H	4.811789	0.297441	0.854384

^{6}VA$_{exo}$

Fe	0.550960	0.887732	0.489861
N	-0.989501	1.274428	1.834497
N	1.234113	-0.529637	1.828206
O	-0.707580	-0.134242	-0.717896
C	-0.737766	-0.688231	3.295313
C	-1.424434	0.433690	2.840540
C	-3.015189	2.021533	2.651348
H	-3.889828	2.649077	2.763337
C	-2.798773	-1.217763	-0.982360
C	-1.930551	3.286651	0.772645
C	0.511022	-1.117063	2.851040
C	-1.958792	2.249734	1.700199
C	-2.688099	0.897242	3.352172
H	-3.240773	0.423140	4.152981
C	-1.503746	-1.034977	-0.369845
H	-1.244838	-1.668882	0.488340
C	2.446109	-1.194921	1.780379
C	1.269900	-2.193663	3.427409
H	0.932846	-2.817499	4.245098
C	2.469044	-2.234597	2.772909
H	3.303576	-2.901229	2.947917
C	-3.215269	-0.393256	-2.046395
C	-4.476297	-0.572658	-2.598004
C	-3.658407	-2.210624	-0.474160
H	-3.327088	-2.840025	0.348195
C	-4.920621	-2.382558	-1.029420
H	-5.588381	-3.146093	-0.643248

C	-5.326111	-1.565920	-2.091605
H	-6.311717	-1.702393	-2.527223
N	2.456548	0.989923	-0.284279
N	0.196983	2.744864	-0.339216
C	2.144012	2.876011	-1.835170
C	2.870702	1.806769	-1.319089
C	4.573239	0.371537	-0.962216
H	5.507683	-0.174070	-0.985040
C	3.489626	-0.896570	0.911156
C	0.913390	3.325940	-1.367146
C	3.492442	0.106800	-0.051139
C	4.185119	1.415281	-1.753019
H	4.740569	1.895219	-2.548261
C	-0.932998	3.519417	-0.169651
C	0.212236	4.483047	-1.857521
H	0.560682	5.121463	-2.658978
C	-0.926372	4.605150	-1.114686
H	-1.695199	5.363451	-1.187556
H	-2.764414	3.981488	0.787127
H	2.595899	3.432103	-2.650373
H	4.384291	-1.505571	0.989354
H	-1.192720	-1.251315	4.104218
H	-4.805865	0.054865	-3.420185
H	-2.540030	0.371125	-2.417252
C	0.432635	-3.344823	-1.334901
C	1.658843	-2.627530	-1.776942
C	0.482353	-4.059668	-0.003358
H	0.790248	-3.384043	0.804013
H	-0.490253	-4.491079	0.253266
H	1.211798	-4.878305	-0.020085
C	2.985104	-3.288629	-1.476687
H	3.139541	-3.406768	-0.397880
H	3.032073	-4.292087	-1.918261
H	3.816869	-2.700272	-1.873584
C	1.592350	-1.450411	-2.418975
H	0.647682	-0.955729	-2.617614
H	2.490720	-0.939462	-2.753185
C	-0.672300	-3.384963	-2.100635
H	-0.702736	-2.928792	-3.085265
H	-1.566351	-3.912343	-1.780635

^{2}TS2A$_{exo}$

Fe	-0.276646	0.696326	-0.809368
N	1.356325	-0.094899	-1.687732
N	-1.358470	-0.805481	-1.578555
O	0.063758	-0.041454	0.873339
C	0.431311	-2.218095	-2.501917
C	1.476558	-1.337980	-2.283000
C	3.569921	-0.522310	-2.225575
H	4.634534	-0.350333	-2.314135
C	2.103845	-1.178302	1.388671
C	3.000663	1.636533	-1.114742
C	-0.892977	-1.962525	-2.178880
C	2.640611	0.408354	-1.642450
C	2.848058	-1.604872	-2.623934
H	3.195155	-2.506484	-3.111535
C	0.673324	-1.197911	0.984656
H	0.421522	-1.964166	0.245248
C	-2.726707	-0.976861	-1.470133
C	-1.978727	-2.865451	-2.441362
H	-1.873221	-3.830549	-2.919816
C	-3.117034	-2.251608	-2.006852
H	-4.137125	-2.610554	-2.056938
C	2.659209	-0.023236	1.956675
C	4.014635	0.009336	2.273373
C	2.912767	-2.296215	1.141593
H	2.480933	-3.185295	0.687204
C	4.269050	-2.258445	1.455472
H	4.896819	-3.121218	1.253636
C	4.820353	-1.106662	2.025411
H	5.877500	-1.078465	2.273151
N	-1.962593	1.659974	-0.332661
N	0.752700	2.372213	-0.446140
C	-1.023295	3.755090	0.538877
C	-2.075734	2.892654	0.281141
C	-4.179477	2.104375	0.175158
H	-5.250599	1.956051	0.224445
C	-3.611553	-0.057544	-0.932005
C	0.293733	3.509898	0.190421
C	-3.250949	1.167343	-0.399008
C	-3.449109	3.171540	0.604038
H	-3.795957	4.080300	1.078485
C	2.117889	2.547758	-0.561762
C	1.380399	4.414301	0.454176
H	1.272939	5.379873	0.930927
C	2.511113	3.818677	-0.014270
H	3.526426	4.193184	-0.001966
H	4.051763	1.903970	-1.136563

H	-1.248347	4.693442	1.035002
H	-4.664811	-0.317940	-0.927199
H	0.660439	-3.167565	-2.974794
H	4.446641	0.906063	2.708087
H	2.025936	0.843661	2.113558
C	-1.445614	-2.311226	2.164449
C	-2.504104	-1.321008	2.267378
C	-1.750549	-3.601537	1.462777
H	-2.079897	-3.408295	0.431873
H	-0.886188	-4.268528	1.435852
H	-2.577356	-4.126401	1.956703
C	-3.926674	-1.742749	1.970156
H	-4.023101	-2.166283	0.966020
H	-4.268034	-2.503900	2.682168
H	-4.605580	-0.889901	2.041569
C	-2.214944	-0.030711	2.568940
H	-1.218706	0.317466	2.803424
H	-2.991307	0.728240	2.543506
C	-0.128218	-2.068769	2.569980
H	0.080544	-1.284137	3.289390
H	0.529431	-2.928787	2.650456

^{4}TS2A$_{exo}$

Fe	0.286383	0.718990	0.796120
N	-1.374593	0.017497	1.684525
N	1.326574	-0.782860	1.633986
O	-0.035587	-0.134578	-0.932530
C	-0.528581	-2.135336	2.519991
C	-1.538597	-1.219097	2.282219
C	-3.598212	-0.316009	2.233082
H	-4.655240	-0.103830	2.324334
C	-2.110081	-1.240388	-1.388631
C	-2.956019	1.808311	1.101711
C	0.809979	-1.918969	2.229813
C	-2.638536	0.575955	1.645345
C	-2.918134	-1.429799	2.623472
H	-3.300957	-2.319383	3.106399
C	-0.652425	-1.285435	-1.048283
H	-0.414440	-2.040442	-0.287472
C	2.694331	-0.978904	1.586494
C	1.865025	-2.842223	2.540294
H	1.719813	-3.801611	3.019857
C	3.034105	-2.255195	2.150331
H	4.043076	-2.635845	2.244501
C	-2.670186	-0.068958	-1.912756
C	-4.038359	-0.007308	-2.167799
C	-2.927058	-2.346386	-1.122459
H	-2.491468	-3.250980	-0.702773
C	-4.295828	-2.281766	-1.375471
H	-4.928309	-3.137996	-1.160048
C	-4.852616	-1.112431	-1.901752
H	-5.919163	-1.062351	-2.101301
N	2.011544	1.624260	0.324912
N	-0.691607	2.414244	0.359352
C	1.132323	3.684682	-0.690174
C	2.159003	2.814209	-0.361794
C	4.245155	1.997516	-0.147713
H	5.314289	1.829044	-0.162170
C	3.615553	-0.090091	1.059105
C	-0.191903	3.500333	-0.333042
C	3.289625	1.114324	0.460094
C	3.543325	3.046049	-0.666528
H	3.918634	3.914639	-1.191918
C	-2.044014	2.657673	0.499864
C	-1.244287	4.436359	-0.621710
H	-1.105117	5.371785	-1.147821
C	-2.390146	3.917350	-0.099756
H	-3.387499	4.337256	-0.110323
H	-3.993734	2.121817	1.134215
H	1.388840	4.587022	-1.234945
H	4.663745	-0.366624	1.098945
H	-0.797669	-3.073162	2.994355
H	-4.472738	0.904153	-2.568661
H	-2.028698	0.789543	-2.083038
C	1.410767	-2.405840	-2.202561
C	2.453916	-1.406085	-2.267573
C	1.717850	-3.705394	-1.519607
H	1.995178	-3.524508	-0.470049
H	0.869931	-4.393026	-1.542217
H	2.579786	-4.199463	-1.983583
C	3.866567	-1.787153	-1.880483
H	3.913723	-2.198541	-0.867554
H	4.271101	-2.544326	-2.562926
H	4.525264	-0.916615	-1.917954
C	2.153316	-0.121100	-2.594150
H	1.175028	0.199855	-2.921935
H	2.907613	0.656654	-2.515119
C	0.078076	-2.146634	-2.574529

H	-0.127675	-1.373641	-3.308287
H	-0.571587	-3.012098	-2.671084

^{6}TS2A$_{exo}$

Fe	-0.321673	0.727984	-0.622873
N	1.403430	0.276347	-1.690000
N	-1.267543	-0.783587	-1.693256
O	0.129986	-0.214975	0.980008
C	0.715007	-1.875076	-2.662957
C	1.663538	-0.902521	-2.358257
C	3.647699	0.161282	-2.210685
H	4.686634	0.457631	-2.272664
C	2.238271	-1.292451	1.330270
C	2.794733	2.176690	-0.974820
C	-0.647542	-1.814303	-2.370061
C	2.608120	0.940922	-1.588534
C	3.065315	-0.977581	-2.684553
H	3.532277	-1.797651	-3.214562
C	0.793790	-1.346018	0.979533
H	0.558501	-2.046927	0.170165
C	-2.611157	-1.095441	-1.646581
C	-1.623101	-2.806336	-2.741148
H	-1.404308	-3.713308	-3.290116
C	-2.837497	-2.359154	-2.299438
H	-3.805730	-2.828883	-2.418064
C	2.772866	-0.152180	1.945479
C	4.139386	-0.081499	2.204146
C	3.079087	-2.357887	0.978367
H	2.662849	-3.235242	0.487749
C	4.445080	-2.283482	1.237784
H	5.096411	-3.105812	0.957213
C	4.975938	-1.145918	1.853965
H	6.041296	-1.088287	2.057387
N	-2.186827	1.535460	-0.242018
N	0.485620	2.592902	-0.234434
C	-1.483512	3.662764	0.777340
C	-2.438623	2.702285	0.449672
C	-4.439711	1.682218	0.225728
H	-5.489131	1.416755	0.239061
C	-3.597036	-0.311143	-1.053026
C	-0.132128	3.621218	0.445427
C	-3.402363	0.901290	-0.397251
C	-3.843544	2.792443	0.753545
H	-4.308973	3.613168	1.284003
C	1.812506	2.946789	-0.357341
C	0.831445	4.647811	0.754648
H	0.605926	5.568311	1.277380
C	2.031897	4.231662	0.258004
H	2.984525	4.744281	0.292477
H	3.802389	2.580084	-0.981766
H	-1.830998	4.536687	1.319389
H	-4.615946	-0.681963	-1.104781
H	1.063701	-2.751424	-3.200560
H	4.555219	0.804728	2.674576
H	2.114232	0.675869	2.186061
C	-1.244754	-2.653851	2.042791
C	-2.345305	-1.718620	2.200845
C	-1.483076	-3.890539	1.227178
H	-1.789113	-3.621799	0.205211
H	-0.594646	-4.523114	1.171009
H	-2.304283	-4.483520	1.647503
C	-3.736956	-2.154613	1.796174
H	-3.773480	-2.476131	0.751122
H	-4.080886	-2.994051	2.412456
H	-4.449156	-1.335556	1.918722
C	-2.122648	-0.455573	2.644711
H	-1.156253	-0.100309	2.974924
H	-2.928162	0.273233	2.656113
C	0.060696	-2.387078	2.476071
H	0.234319	-1.657330	3.259994
H	0.755122	-3.221725	2.490439

^{2}VIA$_{exo}$

Fe	0.472874	0.542827	0.856189
N	-1.439121	0.561680	1.487020
N	0.727410	-1.238432	1.767118
O	-0.019311	-0.167658	-1.049309
C	-1.566939	-1.652809	2.529817
C	-2.127339	-0.451947	2.128478
C	-3.679124	1.112666	1.694543
H	-4.584134	1.696187	1.592113
C	-2.324880	-0.936433	-1.278024
C	-2.127808	2.721194	0.558020
C	-0.234604	-2.030005	2.346734
C	-2.382598	1.515877	1.211862
C	-3.518441	-0.108997	2.271498
H	-4.263606	-0.736938	2.741214
C	-0.871844	-1.344269	-1.174651

H	-0.708257	-1.909671	-0.255439
C	1.880035	-1.997451	1.789107
C	0.311676	-3.304141	2.732399
H	-0.245735	-4.094706	3.217432
C	1.627929	-3.286118	2.379333
H	2.372947	-4.058927	2.516579
C	-2.708213	0.273918	-1.864887
C	-4.059627	0.587584	-2.015014
C	-3.313721	-1.825181	-0.840904
H	-3.023115	-2.763905	-0.373642
C	-4.665254	-1.516829	-0.998075
H	-5.422492	-2.216706	-0.656257
C	-5.041790	-0.308075	-1.586616
H	-6.093335	-0.064996	-1.708664
N	2.475746	0.699453	0.652464
N	0.293399	2.464616	0.282976
C	2.630899	2.979383	-0.242151
C	3.193616	1.779615	0.173163
C	4.734217	0.191344	0.562494
H	5.639465	-0.392078	0.668316
C	3.125451	-1.553621	1.370668
C	1.272847	3.293439	-0.210460
C	3.411210	-0.278914	0.881939
C	4.598622	1.475212	0.124525
H	5.368747	2.160599	-0.204077
C	-0.883553	3.166647	0.133673
C	0.708195	4.532331	-0.679294
H	1.276772	5.350088	-1.102258
C	-0.635346	4.451115	-0.469943
H	-1.396345	5.189925	-0.684711
H	-2.975238	3.377972	0.387562
H	3.305957	3.737016	-0.628639
H	3.958193	-2.243293	1.469691
H	-2.222548	-2.371074	3.012755
H	-4.345653	1.531705	-2.470746
H	-1.948704	0.981840	-2.180625
C	1.072376	-2.287320	-2.532954
C	1.724453	-1.112920	-2.484301
C	1.672403	-3.643946	-2.770528
H	1.454655	-4.311463	-1.925614
H	1.230288	-4.112203	-3.660007
H	2.755296	-3.618444	-2.909166
C	3.185547	-0.860442	-2.730191
H	3.659089	-0.383544	-1.864915
H	3.738589	-1.773826	-2.958472
H	3.320116	-0.176992	-3.580033
C	0.868873	0.090073	-2.178296
H	0.237022	0.380865	-3.028834
H	1.456978	0.957759	-1.881276
C	-0.432088	-2.219511	-2.367970
H	-0.886455	-1.815493	-3.283974
H	-0.871850	-3.211971	-2.225776

^{4}VIA$_{exo}$

Fe	0.344202	0.688039	0.779956
N	-0.202813	-0.837741	1.933776
N	2.259921	0.212466	1.094737
O	0.190786	-0.370412	-1.056052
C	1.957160	-2.003872	2.119814
C	0.588402	-1.915642	2.302956
C	-1.474874	-2.415561	3.048759
H	-2.345649	-2.869635	3.503320
C	-2.089530	-1.219808	-1.236711
C	-2.595765	-0.349287	2.225331
C	2.737980	-0.982885	1.606448
C	-1.473715	-1.139731	2.396412
C	-0.202476	-2.904126	2.975362
H	0.183598	-3.837821	3.363054
C	-0.627166	-1.584289	-1.161641
H	-0.426951	-2.152911	-0.250686
C	3.387673	0.943447	0.757150
C	4.171720	-0.994703	1.588815
H	4.782172	-1.816121	1.940419
C	4.574547	0.206491	1.082201
H	5.582342	0.568385	0.925682
C	-2.522365	-0.094834	-1.946524
C	-3.884758	0.174500	-2.071134
C	-3.034932	-2.070552	-0.654699
H	-2.701278	-2.938769	-0.090642
C	-4.398665	-1.806941	-0.787061
H	-5.125367	-2.475965	-0.334772
C	-4.826086	-0.682972	-1.496465
H	-5.887105	-0.474355	-1.599207
N	0.938279	2.409093	-0.033154
N	-1.511584	1.400670	0.880793
C	-1.256379	3.406162	-0.522236
C	0.124334	3.371829	-0.608987
C	2.225352	4.073909	-0.998821

H	3.113116	4.616896	-1.295700
C	3.383222	2.176578	0.128124
C	-2.007553	2.512125	0.219109
C	2.235378	2.843003	-0.263569
C	0.918976	4.391730	-1.228211
H	0.516120	5.252603	-1.745534
C	-2.608421	0.829463	1.504062
C	-3.418864	2.636178	0.439138
H	-4.035095	3.424960	0.028172
C	-3.788291	1.602518	1.246278
H	-4.771660	1.361585	1.627143
H	-3.533150	-0.699338	2.642604
H	-1.775130	4.226956	-1.005523
H	4.339619	2.634850	-0.099145
H	2.458958	-2.899885	2.468696
H	-4.212492	1.055254	-2.615973
H	-1.788998	0.579903	-2.377794
C	1.320707	-2.618072	-2.392947
C	2.050612	-1.501666	-2.230767
C	1.834612	-4.016671	-2.580989
H	1.476372	-4.669409	-1.773207
H	1.453342	-4.443377	-3.518150
H	2.924642	-4.077052	-2.603157
C	3.548685	-1.373311	-2.244342
H	3.922996	-1.041712	-1.268417
H	4.047556	-2.311282	-2.495903
H	3.868845	-0.623135	-2.979941
C	1.268092	-0.227671	-2.042352
H	0.794569	0.109205	-2.974026
H	1.889964	0.583010	-1.668094
C	-0.179823	-2.428328	-2.367292
H	-0.527860	-1.939022	-3.288703
H	-0.712364	-3.383036	-2.309301

^{6}VIA$_{exo}$

Fe	0.277834	0.667850	0.628086
N	-0.470427	-0.706550	1.962292
N	2.178006	0.138987	1.252270
O	0.268435	-0.458702	-1.096914
C	1.606142	-1.942628	2.436398
C	0.225062	-1.777809	2.496233
C	-1.933699	-2.107874	3.062784
H	-2.867402	-2.482835	3.461489
C	-1.992334	-1.357532	-1.246129
C	-2.839706	-0.038912	1.966793
C	2.514912	-1.039096	1.893154
C	-1.795368	-0.890931	2.312212
C	-0.688016	-2.661595	3.167704
H	-0.403129	-3.575315	3.673082
C	-0.525438	-1.700282	-1.174404
H	-0.306046	-2.243277	-0.251328
C	3.369945	0.758701	0.929367
C	3.946170	-1.163071	1.962394
H	4.472275	-1.991314	2.419073
C	4.474655	-0.046790	1.378551
H	5.517874	0.215960	1.259649
C	-2.446744	-0.245827	-1.963803
C	-3.813635	0.009368	-2.068992
C	-2.919151	-2.211001	-0.638505
H	-2.568947	-3.065436	-0.063862
C	-4.286995	-1.962838	-0.754218
H	-4.999615	-2.633268	-0.282283
C	-4.736437	-0.850754	-1.468908
H	-5.800951	-0.653444	-1.556475
N	1.088101	2.432166	-0.076490
N	-1.553678	1.614248	0.676546
C	-0.998264	3.595547	-0.673048
C	0.386924	3.463822	-0.675349
C	2.566456	3.972997	-0.954766
H	3.510754	4.439034	-1.204733
C	3.484494	1.962898	0.240075
C	-1.896627	2.757867	-0.018697
C	2.429010	2.739110	-0.228923
C	1.305992	4.415421	-1.238381
H	1.015941	5.316315	-1.763330
C	-2.729827	1.118337	1.206014
C	-3.315338	2.977894	0.071756
H	-3.840785	3.807426	-0.383282
C	-3.828358	1.969272	0.834418
H	-4.858131	1.804448	1.122659
H	-3.833119	-0.314576	2.304329
H	-1.411216	4.462712	-1.178649
H	4.488533	2.328040	0.048704
H	2.016564	-2.830145	2.907643
H	-4.158117	0.881591	-2.616434
H	-1.728888	0.430639	-2.417652
C	1.451221	-2.738006	-2.340220
C	2.181295	-1.624185	-2.159066

C	1.965371	-4.141360	-2.486168
H	1.611841	-4.582295	-3.427478
H	3.055134	-4.205581	-2.472597
H	1.579060	-4.778275	-1.678811
C	3.679175	-1.506050	-2.089255
H	3.997746	-1.177336	-1.092073
H	4.183923	-2.447983	-2.311746
H	4.046577	-0.758260	-2.804631
C	1.405565	-0.340465	-2.031339
H	0.975529	-0.013098	-2.986038
H	2.014855	0.470583	-1.635678
C	-0.049495	-2.544029	-2.365981
H	-0.372065	-2.054350	-3.296417
H	-0.585109	-3.497604	-2.320574

^{2}VIIIA$_{exo}$

Fe	-0.283475	0.470406	-0.857740
N	1.320941	-0.469462	-1.615467
N	0.759556	2.183432	-0.794458
C	2.989125	1.307335	-1.334823
C	2.609862	0.013914	-1.662801
C	2.770680	-2.118608	-2.357035
H	3.096022	-3.085075	-2.719241
C	0.341353	-2.662557	-2.124708
C	2.125436	2.314064	-0.939167
C	1.410261	-1.780740	-2.036137
C	3.516772	-1.003775	-2.121351
H	4.582668	-0.865688	-2.245071
C	0.322855	3.410074	-0.353304
C	2.541880	3.652487	-0.610772
H	3.562726	4.008207	-0.660774
C	1.420853	4.334510	-0.247305
H	1.328449	5.367194	0.062580
N	-1.968986	1.521774	-0.493294
N	-1.412159	-1.127423	-1.338602
C	-3.635468	-0.263826	-0.742715
C	-3.251666	1.044043	-0.437860
C	-3.423551	3.196857	0.190598
H	-3.754823	4.179687	0.499657
C	-0.993961	3.725991	-0.033275
C	-2.775716	-1.257219	-1.204613
C	-2.056899	2.849297	-0.108434
C	-4.167323	2.076958	-0.018424
H	-5.237985	1.949861	0.080102
C	-0.973322	-2.352689	-1.817275
C	-3.200587	-2.574399	-1.597258
H	-4.228309	-2.915282	-1.599794
C	-2.081036	-3.255609	-1.972957
H	-2.001450	-4.269016	-2.344637
O	0.036751	0.118652	0.870676
C	2.123784	-0.883223	1.555649
C	0.648825	-1.060672	1.271984
H	0.517303	-1.865477	0.538673
C	2.618835	0.366323	1.945141
C	3.982604	0.535634	2.182964
C	3.003689	-1.961665	1.415119
H	2.624260	-2.930407	1.096073
C	4.367495	-1.792841	1.652593
H	5.046518	-2.631983	1.530994
C	4.859155	-0.542609	2.037916
H	5.921283	-0.410391	2.223582
H	0.548836	-3.666101	-2.482674
H	-4.698693	-0.482315	-0.748523
H	-1.199301	4.739790	0.295220
H	4.044663	1.549533	-1.399835
H	1.932128	1.202509	2.026708
H	4.361859	1.510268	2.477553
C	-2.503730	-0.823243	2.322001
C	-1.513351	-1.832072	2.236452
C	-0.111393	-1.549133	2.601147
H	0.002046	-0.755036	3.339436
H	0.405866	-2.448819	2.946046
C	-1.748535	-3.209935	1.708573
H	-2.726681	-3.387076	1.267323
H	-0.991424	-3.442731	0.947791
H	-1.594410	-3.931381	2.523345
C	-2.187240	0.532582	2.921364
H	-1.919193	0.444499	3.980677
H	-1.353360	0.992743	2.388317
H	-3.050915	1.197662	2.846668
C	-3.765897	-1.050974	1.817540
H	-4.078591	-1.997832	1.399239
H	-4.541333	-0.301065	1.926210

^{6}VIIIA$_{exo}$

Fe	-0.339502	0.543831	-0.641295
N	1.196506	-0.177149	-1.855275
N	0.585614	2.408058	-0.691361

C	2.778279	1.678411	-1.529623
C	2.448065	0.389225	-1.949794
C	2.636843	-1.662618	-2.867150
H	2.979056	-2.568361	-3.350757
C	0.228747	-2.335286	-2.519978
C	1.917896	2.619240	-0.966035
C	1.290813	-1.428849	-2.409774
C	3.354959	-0.537159	-2.577495
H	4.400422	-0.341072	-2.776395
C	0.112285	3.565203	-0.132673
C	2.286532	3.959305	-0.581361
H	3.273302	4.387895	-0.699307
C	1.166134	4.547492	-0.068522
H	1.054623	5.551878	0.318840
N	-2.126180	1.533503	-0.188746
N	-1.527908	-1.030572	-1.387043
C	-3.711348	-0.321085	-0.505834
C	-3.370731	0.981020	-0.089399
C	-3.583245	3.071075	0.730981
H	-3.940872	4.005618	1.143574
C	-1.198157	3.754992	0.327766
C	-2.868337	-1.214635	-1.189024
C	-2.228712	2.826829	0.296194
C	-4.291251	1.929671	0.490945
H	-5.343863	1.746589	0.666022
C	-1.075848	-2.140471	-2.083558
C	-3.284509	-2.474196	-1.757388
H	-4.299206	-2.852206	-1.752866
C	-2.173929	-3.047726	-2.307183
H	-2.100658	-3.987904	-2.838615
O	0.276652	-0.054587	0.973114
C	2.385594	-1.070939	1.541825
C	0.916638	-1.262472	1.228264
H	0.832190	-1.939595	0.364650
C	2.859866	0.166081	1.990829
C	4.218541	0.338619	2.258494
C	3.282046	-2.130828	1.362625
H	2.919308	-3.090105	0.997455
C	4.638860	-1.959223	1.633406
H	5.329081	-2.785315	1.487680
C	5.109562	-0.722343	2.083252
H	6.166720	-0.587107	2.293219
H	0.440076	-3.272810	-3.025681
H	-4.766753	-0.574791	-0.480007
H	-1.431738	4.731576	0.740928
H	3.809594	1.986059	-1.672773
H	2.161747	0.988607	2.106440
H	4.581749	1.302825	2.603435
C	-2.263658	-1.271822	2.262222
C	-1.245999	-2.213021	2.009712
C	0.156020	-1.966522	2.434632
H	0.241092	-1.305325	3.298211
H	0.674426	-2.906746	2.647712
C	-1.443644	-3.493921	1.263580
H	-2.374616	-3.572755	0.706110
H	-0.618921	-3.638103	0.554894
H	-1.384651	-4.330530	1.974986
C	-1.988882	-0.008966	3.057005
H	-1.756327	-0.245231	4.102269
H	-1.145901	0.535230	2.630192
H	-2.861456	0.649309	3.048590
C	-3.546847	-1.484553	1.781632
H	-3.844357	-2.390087	1.270949
H	-4.351951	-0.820490	2.074549

Optimized by other DFT methods in solution

PBE0-D3

^{4}IA

Fe	0.001055	-0.000984	-0.054008
N	-0.313025	1.647816	1.024860
N	-0.222935	1.056244	-1.726443
O	2.143530	0.502540	-0.069700
C	-0.634226	3.278939	-0.776944
C	-0.541573	2.918816	0.555226
C	-0.535404	3.111729	2.789384
H	-0.574215	3.453089	3.816156
C	4.459693	0.046751	0.084016
C	-0.095204	0.715734	3.282236
C	-0.478649	2.401225	-1.833855
C	-0.300787	1.755345	2.394391
C	-0.685970	3.834474	1.646842
H	-0.874992	4.894991	1.537069
C	3.053795	-0.314349	0.070825
H	2.815227	-1.383459	0.194926
C	-0.126760	0.593458	-3.015249
C	-0.556739	2.785381	-3.211875
H	-0.750288	3.793248	-3.557102
C	-0.335211	1.662292	-3.945651

H	-0.310933	1.550277	-5.022156
C	4.857196	1.384664	-0.061467
C	6.205273	1.702410	-0.043437
C	5.416809	-0.963574	0.246433
H	5.095333	-1.996778	0.358418
C	6.766885	-0.639173	0.263367
H	7.514742	-1.416626	0.387623
C	7.157143	0.691567	0.118724
H	8.213568	0.945970	0.131948
N	0.322437	-1.648800	-1.129289
N	0.222036	-1.060661	1.620487
C	0.596179	-3.290324	0.671391
C	0.533421	-2.923081	-0.660450
C	0.575195	-3.107075	-2.894752
H	0.634292	-3.444159	-3.921972
C	0.136789	-0.711513	-3.387449
C	0.446984	-2.411551	1.728918
C	0.337548	-1.751298	-2.499081
C	0.694146	-3.835544	-1.752509
H	0.873576	-4.897715	-1.642785
C	0.141877	-0.594238	2.909875
C	0.519545	-2.795962	3.106842
H	0.690090	-3.807712	3.452880
C	0.330942	-1.666343	3.840212
H	0.315257	-1.552548	4.916712
O	-2.145603	-0.484611	-0.028458
C	-4.465235	-0.039054	0.101350
C	-3.061409	0.329824	0.087857
H	-2.828726	1.402555	0.191721
C	-4.855143	-1.381109	-0.025193
C	-6.201462	-1.706081	-0.003394
C	-5.428006	0.968120	0.249362
H	-5.112330	2.004555	0.347110
C	-6.776221	0.636377	0.271077
H	-7.528460	1.411230	0.384783
C	-7.158950	-0.698421	0.144976
H	-8.213934	-0.958511	0.161961
H	-0.123004	0.942000	4.342949
H	0.770247	-4.336239	0.901350
H	0.180863	-0.935349	-4.448109
H	-0.827615	4.321342	-1.007537
H	6.523775	2.734751	-0.154632
H	4.097494	2.150805	-0.184495
H	-4.091000	-2.144663	-0.136506
H	-6.514251	-2.741617	-0.100154

^{6}IA

Fe	0.001135	-0.007263	0.119249
N	0.151766	-0.890802	1.953405
N	-0.317787	1.810986	1.005783
O	2.102095	0.395084	0.102829
C	-0.196131	1.094418	3.352193
C	0.038117	-0.265930	3.166256
C	0.402733	-2.440641	3.621311
H	0.560132	-3.401690	4.094957
C	4.408385	0.021593	-0.254258
C	0.523364	-3.206616	1.230324
C	-0.353503	2.053301	2.354163
C	0.369779	-2.220045	2.201552
C	0.196001	-1.232734	4.217846
H	0.151634	-1.008572	5.276368
C	3.008589	-0.341911	-0.296973
H	2.756982	-1.330449	-0.710606
C	-0.476914	3.017094	0.374777
C	-0.556730	3.457580	2.581251
H	-0.626070	3.922318	3.556848
C	-0.631203	4.053896	1.357829
H	-0.774004	5.103570	1.132742
C	4.818067	1.264195	0.255721
C	6.166419	1.578250	0.282519
C	5.353841	-0.896995	-0.732613
H	5.021522	-1.856047	-1.123817
C	6.703783	-0.575831	-0.702362
H	7.442185	-1.281348	-1.071075
C	7.105847	0.659500	-0.195454
H	8.162601	0.911667	-0.171543
N	-0.162390	0.883015	-1.726846
N	0.299105	-1.822223	-0.785529
C	0.142012	-1.110182	-3.129714
C	-0.063227	0.254651	-2.941375
C	-0.365306	2.439328	-3.396909
H	-0.494327	3.404320	-3.871195
C	-0.492224	3.206381	-1.005504
C	0.300493	-2.071610	-2.133631
C	-0.341157	2.218661	-1.977418
C	-0.194865	1.225022	-3.992783
H	-0.156708	0.999539	-5.051321
C	0.476769	-3.024704	-0.150500
C	0.501666	-3.476188	-2.357274

H	0.546219	-3.947491	-3.331209
C	0.611495	-4.065093	-1.131738
H	0.763722	-5.113117	-0.905075
O	-2.093952	-0.449019	0.135955
C	-4.387371	-0.013019	-0.231252
C	-2.978912	0.318574	-0.254612
H	-2.704070	1.309859	-0.645941
C	-4.828369	-1.256807	0.248929
C	-6.183131	-1.543144	0.254918
C	-5.308172	0.934146	-0.701320
H	-4.952320	1.893649	-1.070033
C	-6.664713	0.640624	-0.692354
H	-7.384135	1.368492	-1.055028
C	-7.097975	-0.595824	-0.214820
H	-8.159865	-0.826539	-0.207919
H	0.680337	-4.221185	1.584144
H	0.182889	-1.460468	-4.156678
H	-0.627050	4.224315	-1.358787
H	-0.254392	1.444419	4.378358
H	6.495786	2.535984	0.674343
H	4.069177	1.960174	0.622117
H	-4.098147	-1.975977	0.607913
H	-6.536337	-2.501566	0.623640

^{4}TS1A$_{endo}$

Fe	0.413566	0.283177	0.051410
N	0.429977	-1.571976	0.813101
N	0.408827	1.038450	1.904830
O	2.703506	0.279381	0.205596
C	0.363310	-1.039297	3.205176
C	0.391545	-1.921428	2.139094
C	0.339828	-3.864069	1.018660
H	0.303604	-4.902001	0.712725
C	4.863006	-0.639351	-0.127927
C	0.424275	-2.874527	-1.263339
C	0.369686	0.340207	3.084338
C	0.396409	-2.752849	0.114852
C	0.340803	-3.348057	2.276580
H	0.306341	-3.872703	3.223073
C	3.410715	-0.621294	-0.235805
H	2.947849	-1.480754	-0.750435
C	0.416441	2.364833	2.254842
C	0.331189	1.244358	4.196635
H	0.297145	0.938893	5.234878
C	0.356210	2.502652	3.681262
H	0.349102	3.449089	4.207132
C	5.549549	0.407690	0.503809
C	6.931643	0.363201	0.594455
C	5.567084	-1.724087	-0.665166
H	5.023040	-2.530610	-1.152129
C	6.952495	-1.764009	-0.572076
H	7.504333	-2.602361	-0.986776
C	7.630710	-0.721117	0.057021
H	8.714472	-0.751570	0.130806
N	0.601976	2.123714	-0.698169
N	0.580889	-0.485059	-1.789514
C	0.748916	1.587413	-3.085648
C	0.718683	2.468807	-2.019608
C	0.720945	4.413863	-0.901268
H	0.747535	5.452273	-0.595596
C	0.499896	3.429488	1.374191
C	0.672404	0.211356	-2.966769
C	0.607315	3.304110	-0.000244
C	0.792113	3.894779	-2.156401
H	0.892286	4.416787	-3.099786
C	0.513197	-1.809457	-2.142826
C	0.671453	-0.691415	-4.080982
H	0.739792	-0.386472	-5.117695
C	0.569892	-1.947031	-3.569391
H	0.536580	-2.892175	-4.096570
O	-1.622842	0.286856	-0.083563
C	-3.317174	-1.346166	0.307726
C	-2.407503	-0.261763	0.785464
H	-1.980693	-0.462324	1.780097
C	-3.527161	-1.524650	-1.061439
C	-4.372538	-2.534423	-1.511580
C	-3.951914	-2.191422	1.219922
H	-3.778040	-2.061057	2.286688
C	-4.796574	-3.200930	0.769078
H	-5.285001	-3.858385	1.483352
C	-5.010867	-3.371954	-0.597816
H	-5.669357	-4.161419	-0.949929
H	7.472186	1.169045	1.082378
H	4.983024	1.239020	0.913270
H	-3.007854	-0.872552	-1.757810
H	-4.529880	-2.673180	-2.578158
C	-3.935199	2.110026	-0.698033
C	-4.381311	1.418554	0.484921
C	-3.503259	1.112916	1.531495

H	-2.669543	1.774904	1.748468
H	-3.940810	0.657000	2.417386
C	-5.738147	0.803370	0.477936
H	-6.499724	1.544126	0.205888
H	-5.788808	0.009622	-0.281359
H	-5.998537	0.367080	1.443699
C	-4.827116	2.153465	-1.909233
H	-5.119722	1.147870	-2.232659
H	-5.751385	2.709206	-1.710234
H	-4.320735	2.643340	-2.745328
C	-2.675629	2.610308	-0.748003
H	-2.033157	2.686149	0.117989
H	-2.275766	3.018570	-1.672126
H	0.380808	-3.874514	-1.682202
H	0.505896	4.431359	1.790971
H	0.330920	-1.457375	4.206024
H	0.832471	2.004092	-4.084113

^{6}TS1A$_{endo}$

Fe	0.367612	0.261804	0.011278
N	0.442807	-1.638664	0.829488
N	0.453663	1.058581	1.909438
O	2.608696	0.260938	0.157238
C	0.389959	-1.023201	3.205579
C	0.398835	-1.947933	2.161791
C	0.332167	-3.915436	1.067142
H	0.288716	-4.960909	0.788191
C	4.780931	-0.638733	-0.114259
C	0.428675	-2.940744	-1.250578
C	0.428349	0.366585	3.088444
C	0.401775	-2.819358	0.138183
C	0.332208	-3.377024	2.318840
H	0.289342	-3.893791	3.269567
C	3.332877	-0.647623	-0.244008
H	2.887187	-1.529273	-0.732786
C	0.522895	2.389654	2.219385
C	0.456817	1.294845	4.187453
H	0.445747	1.016293	5.233936
C	0.513915	2.546282	3.650024
H	0.559751	3.495592	4.169169
C	5.441665	0.438653	0.494402
C	6.822191	0.416873	0.609147
C	5.508620	-1.730563	-0.604568
H	4.983237	-2.559707	-1.073790
C	6.892506	-1.747172	-0.487041
H	7.463427	-2.590471	-0.864144
C	7.544971	-0.674566	0.119255
H	8.627565	-0.687180	0.212667
N	0.624103	2.130332	-0.787268
N	0.577461	-0.569043	-1.861180
C	0.718712	1.508947	-3.158976
C	0.720028	2.434540	-2.116770
C	0.803285	4.401152	-1.020155
H	0.869047	5.445403	-0.740626
C	0.617803	3.429374	1.294384
C	0.644882	0.122425	-3.039550
C	0.682166	3.307763	-0.093183
C	0.826087	3.861338	-2.272000
H	0.918379	4.375737	-3.220565
C	0.508440	-1.899562	-2.174561
C	0.625339	-0.805214	-4.139437
H	0.671614	-0.526234	-5.184849
C	0.540895	-2.055707	-3.604779
H	0.503008	-3.004969	-4.124602
O	-1.609958	0.255798	-0.073812
C	-3.319970	-1.361268	0.303997
C	-2.383810	-0.315246	0.787763
H	-1.963524	-0.515181	1.783199
C	-3.538200	-1.528104	-1.066283
C	-4.396485	-2.526380	-1.515936
C	-3.959714	-2.203834	1.216919
H	-3.776868	-2.081600	2.282895
C	-4.817744	-3.200069	0.764948
H	-5.309988	-3.856198	1.477517
C	-5.039257	-3.360681	-0.602368
H	-5.707873	-4.141009	-0.955505
H	7.343213	1.245621	1.079513
H	4.856692	1.274378	0.867280
H	-3.014894	-0.881182	-1.764461
H	-4.560219	-2.658997	-2.582079
C	-3.901109	2.269730	-0.550218
C	-4.332357	1.465609	0.576934
C	-3.475321	1.116734	1.615955
H	-2.615855	1.736504	1.854016
H	-3.910056	0.603016	2.470205
C	-5.678852	0.831201	0.512839
H	-6.455869	1.582973	0.329322
H	-5.722536	0.126055	-0.329572
H	-5.924344	0.287395	1.426405

C	-4.845663	2.482858	-1.701552
H	-5.181861	1.534590	-2.136610
H	-5.743516	3.028364	-1.386546
H	-4.365758	3.063768	-2.493624
C	-2.639506	2.755820	-0.593713
H	-1.946418	2.681433	0.232960
H	-2.273914	3.278279	-1.473602
H	0.382481	-3.948503	-1.652419
H	0.669768	4.436589	1.697228
H	0.361578	-1.426121	4.213693
H	0.784665	1.908296	-4.166696

^{4}TS2A$_{endo}$

Fe	0.986723	0.770043	0.523447
N	1.729916	1.641333	-1.114054
N	2.594316	-0.414635	0.603202
C	3.680605	0.206539	-1.505833
C	2.823346	1.235679	-1.844398
C	1.954141	2.946738	-3.002964
H	1.734265	3.730377	-3.716991
C	0.078787	3.423480	-1.444361
C	3.573072	-0.548121	-0.353209
C	1.193364	2.695339	-1.816671
C	2.967076	2.039922	-3.019913
H	3.756431	1.919908	-3.751405
C	2.884552	-1.359716	1.558278
C	4.481814	-1.595614	0.002402
H	5.341056	-1.888443	-0.587726
C	4.054799	-2.099375	1.191672
H	4.489490	-2.893662	1.785260
N	0.482176	0.168035	2.356614
N	-0.376274	2.225139	0.645164
C	-1.484689	1.582077	2.734813
C	-0.611480	0.571067	3.084910
C	0.295562	-1.095121	4.277633
H	0.544195	-1.847663	5.015199
C	2.161904	-1.580869	2.714586
C	-1.359613	2.355244	1.598005
C	1.042174	-0.858685	3.078222
C	-0.734370	-0.208571	4.279889
H	-1.513415	-0.075437	5.019968
C	-0.643988	3.198967	-0.288635
C	-2.250609	3.422496	1.258065
H	-3.104203	3.720390	1.853494
C	-1.804238	3.949350	0.087304
H	-2.212140	4.774083	-0.483178
O	-0.194414	-0.513499	-0.396739
C	-2.542173	-0.402000	-0.799587
C	-1.132904	-0.201111	-1.223353
H	-0.977621	0.690840	-1.848259
C	-2.825206	-1.135796	0.355483
C	-4.142452	-1.291508	0.774578
C	-3.584450	0.179851	-1.525359
H	-3.360322	0.764084	-2.416020
C	-4.900545	0.020871	-1.106255
H	-5.708599	0.477364	-1.671178
C	-5.181237	-0.716634	0.043633
H	-6.209835	-0.837596	0.372325
H	-0.241941	4.230422	-2.094833
H	-2.314782	1.790093	3.401402
H	2.500772	-2.365003	3.383387
H	4.506593	-0.006768	-2.176316
H	-2.000731	-1.558446	0.922425
H	-4.359807	-1.856915	1.676932
C	-0.171032	-3.314868	-1.529668
C	-1.110474	-2.592801	-2.364648
C	-0.850006	-1.312197	-2.840949
H	0.172386	-0.973049	-2.978246
H	-1.564543	-0.886062	-3.541149
C	-2.478014	-3.156741	-2.540208
H	-2.430977	-4.185486	-2.917339
H	-2.995117	-3.201796	-1.570651
H	-3.084905	-2.558975	-3.222433
C	-0.605259	-4.604992	-0.888766
H	-1.495532	-4.467334	-0.264249
H	-0.855237	-5.364189	-1.639317
H	0.189802	-5.010178	-0.257581
C	1.044898	-2.787071	-1.257405
H	1.428651	-1.896127	-1.736675
H	1.715517	-3.271641	-0.552776

^{6}TS2A$_{endo}$

Fe	1.027570	0.753903	0.000282
N	1.451228	0.702534	-2.017510
N	2.702709	-0.404997	0.322550
C	3.207536	-1.013441	-1.999693
C	2.317846	-0.160949	-2.643493
C	1.239069	0.918044	-4.296019
H	0.880512	1.292195	-5.246849
C	-0.166674	2.369520	-2.812111

C	3.398284	-1.111933	-0.625981
C	0.790166	1.380154	-3.013324
C	2.183596	-0.036563	-4.067217
H	2.755142	-0.601518	-4.793124
C	3.200717	-0.791781	1.541533
C	4.351291	-1.975234	0.012903
H	5.031831	-2.637541	-0.507611
C	4.229364	-1.776294	1.355215
H	4.790920	-2.241767	2.155670
N	1.092785	1.291188	1.981724
N	-0.137471	2.423195	-0.362049
C	-0.639343	3.029770	1.960219
C	0.249397	2.176533	2.603968
C	1.393387	1.167302	4.255885
H	1.803373	0.844441	5.204669
C	2.776012	-0.305951	2.772504
C	-0.809529	3.151785	0.586494
C	1.802445	0.664944	2.974104
C	0.431655	2.103331	4.026701
H	-0.106267	2.703848	4.749609
C	-0.589916	2.858320	-1.581508
C	-1.709757	4.069944	-0.053621
H	-2.355949	4.765558	0.466951
C	-1.572999	3.889241	-1.396059
H	-2.083622	4.407030	-2.198377
O	-0.403232	-0.533306	0.080848
C	-2.768607	-0.538854	-0.203119
C	-1.395854	-0.587264	-0.743993
H	-1.269558	-0.104426	-1.721354
C	-2.985038	-0.621973	1.176032
C	-4.279832	-0.549100	1.678519
C	-3.852517	-0.376856	-1.071623
H	-3.679292	-0.302595	-2.143354
C	-5.144687	-0.306615	-0.565685
H	-5.985374	-0.177910	-1.241417
C	-5.359586	-0.395427	0.809626
H	-6.370009	-0.337672	1.204593
H	-0.609269	2.813808	-3.698494
H	-1.244134	3.675638	2.589427
H	3.260518	-0.709842	3.656227
H	3.833219	-1.638739	-2.629481
H	-2.130734	-0.720218	1.839522
H	-4.448372	-0.604611	2.750374
C	-0.602352	-3.551661	0.378255
C	-1.507413	-3.241737	-0.719396
C	-1.169335	-2.361721	-1.734878
H	-0.132239	-2.139709	-1.968216
H	-1.859801	-2.250389	-2.567271
C	-2.909835	-3.736528	-0.633902
H	-2.932108	-4.815880	-0.443546
H	-3.424718	-3.259069	0.212788
H	-3.479259	-3.526208	-1.540988
C	-1.149005	-4.260015	1.588071
H	-2.002432	-3.725049	2.020365
H	-1.495158	-5.271156	1.342089
H	-0.382643	-4.350758	2.362151
C	0.689201	-3.158483	0.336648
H	1.143749	-2.702412	-0.534494
H	1.349834	-3.326570	1.182683

B3PW91-D3

^{4}IA

Fe	-0.000025	-0.000100	0.000253
N	0.253320	-1.473422	1.322204
N	-0.416346	1.271744	1.483695
O	2.120867	0.579157	0.159852
C	-0.199553	-0.246611	3.401803
C	0.120724	-1.389117	2.689884
C	0.618401	-3.540553	2.278789
H	0.839184	-4.599100	2.337788
C	4.445240	0.111934	0.126225
C	0.761584	-3.335018	-0.197904
C	-0.444543	0.989598	2.830669
C	0.551053	-2.791871	1.058018
C	0.352962	-2.669647	3.291515
H	0.309951	-2.864742	4.355726
C	3.037786	-0.244779	0.103461
H	2.798616	-1.317417	0.028249
C	-0.692790	2.617026	1.380048
C	-0.758240	2.171165	3.579825
H	-0.842643	2.208883	4.658714
C	-0.911820	3.181536	2.679517
H	-1.147877	4.221800	2.865641
C	4.846055	1.454454	0.236538
C	6.196971	1.767142	0.243203
C	5.404178	-0.907689	0.022340
H	5.083166	-1.943627	-0.063070
C	6.756841	-0.587656	0.026768
H	7.504473	-1.371378	-0.054111

C	7.149637	0.747405	0.136810
H	8.207279	0.997773	0.140543
N	-0.253229	1.473268	-1.321692
N	0.416312	-1.271929	-1.483178
C	0.199211	0.246356	-3.401318
C	-0.120858	1.388916	-2.689389
C	-0.618076	3.540451	-2.278255
H	-0.838669	4.599038	-2.337243
C	-0.761158	3.334930	0.198448
C	0.444301	-0.989825	-2.830168
C	-0.550714	2.791774	-1.057483
C	-0.352976	2.669472	-3.291009
H	-0.310094	2.864541	-4.355230
C	0.692982	-2.617166	-1.379518
C	0.758059	-2.171379	-3.579321
H	0.842323	-2.209127	-4.658221
C	0.911932	-3.181687	-2.678993
H	1.148123	-4.221923	-2.865106
O	-2.120922	-0.579229	-0.159289
C	-4.445262	-0.111722	-0.126799
C	-3.037776	0.244811	-0.103291
H	-2.798517	1.317409	-0.027836
C	-4.846205	-1.454170	-0.237570
C	-6.197158	-1.766680	-0.244923
C	-5.404120	0.908005	-0.023187
H	-5.083017	1.943887	0.062564
C	-6.756822	0.588149	-0.028310
H	-7.504388	1.371955	0.052368
C	-7.149742	-0.746842	-0.138777
H	-8.207414	-0.997070	-0.143045
H	0.990023	-4.394047	-0.260215
H	0.262945	0.325344	-4.481580
H	-0.989415	4.393998	0.260779
H	-0.263458	-0.325653	4.482051
H	6.516659	2.801774	0.328510
H	4.087619	2.227872	0.313181
H	-4.087843	-2.227679	-0.314035
H	-6.516938	-2.801254	-0.330584

[6]IA

Fe	-0.000256	0.001249	0.054189
N	0.270230	-1.117719	1.747008
N	-0.350287	1.669048	1.194403
O	2.078968	0.512915	0.011098
C	-0.102338	0.647461	3.417808
C	0.176173	-0.665218	3.039548
C	0.622774	-2.871235	3.183920
H	0.823533	-3.881790	3.517903
C	4.400121	0.068163	-0.103210
C	0.682196	-3.306213	0.705792
C	-0.337598	1.726331	2.566616
C	0.533242	-2.463972	1.807051
C	0.400705	-1.759945	3.945256
H	0.385233	-1.682013	5.025242
C	3.001789	-0.306636	-0.081724
H	2.765710	-1.378841	-0.155522
C	-0.578694	2.944541	0.738048
C	-0.582609	3.079164	2.989180
H	-0.627413	3.406169	4.020692
C	-0.729519	3.832135	1.859576
H	-0.920532	4.895705	1.786031
C	4.787334	1.416313	-0.002369
C	6.133808	1.744293	-0.031560
C	5.368039	-0.940947	-0.234055
H	5.056672	-1.980315	-0.311661
C	6.715921	-0.604754	-0.264710
H	7.470500	-1.379282	-0.365578
C	7.095009	0.735186	-0.163461
H	8.149343	0.998010	-0.186800
N	-0.268213	1.123154	-1.644556
N	0.339958	-1.666993	-1.093893
C	0.076031	-0.648079	-3.316142
C	-0.183135	0.668350	-2.937354
C	-0.590925	2.881852	-3.082708
H	-0.774406	3.895515	-3.417260
C	-0.657693	3.316157	-0.604078
C	0.310509	-1.727831	-2.465600
C	-0.508377	2.473675	-1.705896
C	-0.390630	1.765894	-3.843494
H	-0.378452	1.686855	-4.923464
C	0.579730	-2.940282	-0.636668
C	0.553263	-3.081121	-2.887438
H	0.585724	-3.411186	-3.918443
C	0.719574	-3.830249	-1.757552
H	0.916296	-4.892783	-1.683858
O	-2.078312	-0.522882	0.080715
C	-4.397447	-0.076567	-0.067222
C	-2.997912	0.294522	-0.053364
H	-2.760557	1.362728	-0.169780

C	-4.788715	-1.420584	0.067561
C	-6.136171	-1.745087	0.046911
C	-5.362434	0.931949	-0.221963
H	-5.047985	1.968124	-0.325638
C	-6.711443	0.599473	-0.241388
H	-7.463751	1.373787	-0.359251
C	-7.094536	-0.736463	-0.107414
H	-8.149698	-0.996517	-0.122908
H	0.883686	-4.352816	0.913778
H	0.098904	-0.852805	-4.382276
H	-0.846273	4.365212	-0.811722
H	-0.134099	0.851168	4.483887
H	6.444004	2.782306	0.046186
H	4.022350	2.180725	0.095619
H	-4.026075	-2.184729	0.183826
H	-6.449356	-2.780053	0.149466

^{4}TS1A$_{endo}$

Fe	0.365249	0.380210	0.087614
N	0.420688	-1.461892	0.895460
N	0.532467	1.180419	1.918312
O	2.673195	0.372189	0.044910
C	0.574602	-0.866812	3.272445
C	0.484705	-1.778171	2.232703
C	0.282832	-3.747252	1.167902
H	0.189824	-4.791062	0.895448
C	4.769528	-0.695742	-0.268816
C	0.209186	-2.814699	-1.141819
C	0.592820	0.510267	3.115575
C	0.294563	-2.660371	0.231571
C	0.405613	-3.200597	2.409407
H	0.436450	-3.702937	3.368195
C	3.313438	-0.633737	-0.256499
H	2.778309	-1.558053	-0.534076
C	0.563573	2.517287	2.232127
C	0.660639	1.444759	4.204355
H	0.719051	1.167262	5.249507
C	0.638172	2.690792	3.656138
H	0.677195	3.650124	4.156909
C	5.538787	0.431860	0.061990
C	6.923216	0.345860	0.048565
C	5.396227	-1.903164	-0.609434
H	4.791644	-2.771554	-0.862870
C	6.784407	-1.984880	-0.619419
H	7.275456	-2.917883	-0.880684
C	7.544344	-0.861103	-0.290905
H	8.629407	-0.923940	-0.299059
N	0.474656	2.202205	-0.724164
N	0.366815	-0.438901	-1.743972
C	0.418287	1.603515	-3.103922
C	0.470691	2.514275	-2.061767
C	0.523533	4.490233	-0.991623
H	0.553242	5.537165	-0.716393
C	0.540925	3.560100	1.320428
C	0.358245	0.229183	-2.944706
C	0.515339	3.401697	-0.055785
C	0.501206	3.939397	-2.237510
H	0.508213	4.439908	-3.197765
C	0.252178	-1.773493	-2.054239
C	0.246968	-0.704251	-4.030634
H	0.224545	-0.428583	-5.077646
C	0.177186	-1.946704	-3.477659
H	0.083800	-2.903979	-3.975190
O	-1.642498	0.425032	0.140447
C	-3.069745	-1.463498	0.426492
C	-2.382752	-0.250458	0.957215
H	-2.002586	-0.369735	1.983527
C	-3.076253	-1.700358	-0.951348
C	-3.683950	-2.845150	-1.460626
C	-3.671460	-2.383448	1.290342
H	-3.655254	-2.206510	2.364130
C	-4.280583	-3.526812	0.780167
H	-4.744097	-4.241084	1.455646
C	-4.289969	-3.758473	-0.596674
H	-4.763887	-4.652497	-0.993680
H	7.525574	1.213766	0.301894
H	5.032938	1.356643	0.323655
H	-2.577930	-0.988820	-1.602485
H	-3.677912	-3.029392	-2.532014
C	-3.971261	2.090341	-0.598247
C	-4.491486	1.233181	0.440500
C	-3.762062	0.954087	1.601183
H	-3.064369	1.689004	1.992928
H	-4.261199	0.368728	2.370168
C	-5.718164	0.431608	0.155252
H	-6.542152	1.074619	-0.177013
H	-5.519476	-0.274991	-0.663941
H	-6.047806	-0.142792	1.022974
C	-4.700148	2.179201	-1.914796

H	-4.810807	1.194526	-2.384418
H	-5.708936	2.591122	-1.788784
H	-4.163969	2.826232	-2.614541
C	-2.774040	2.710674	-0.432479
H	-2.242992	2.749584	0.508754
H	-2.306483	3.247753	-1.253287
H	0.103491	-3.822146	-1.530380
H	0.568935	4.572500	1.710215
H	0.621480	-1.258363	4.283526
H	0.409854	1.994492	-4.116193

^{6}TS1A$_{endo}$

Fe	0.337752	0.364649	0.074160
N	0.424966	-1.540975	0.896417
N	0.609704	1.164193	1.956063
O	2.583273	0.322331	0.012838
C	0.606495	-0.912247	3.266826
C	0.486029	-1.842998	2.233077
C	0.233186	-3.811907	1.159678
H	0.115262	-4.855381	0.894772
C	4.683075	-0.732529	-0.299268
C	0.165793	-2.849370	-1.167234
C	0.668883	0.476931	3.138535
C	0.267409	-2.722991	0.218603
C	0.371020	-3.268943	2.404377
H	0.389770	-3.781641	3.358266
C	3.229283	-0.681158	-0.293338
H	2.698437	-1.604169	-0.577155
C	0.701081	2.498408	2.251355
C	0.795350	1.413163	4.227444
H	0.865337	1.141216	5.273571
C	0.813712	2.662551	3.679212
H	0.903752	3.614422	4.188146
C	5.444567	0.398124	0.040864
C	6.829184	0.318759	0.034430
C	5.317557	-1.935836	-0.642074
H	4.718495	-2.806006	-0.902039
C	6.705885	-2.010345	-0.644513
H	7.203226	-2.939659	-0.906824
C	7.457975	-0.883868	-0.306948
H	8.543368	-0.941195	-0.309237
N	0.516696	2.226273	-0.756260
N	0.342253	-0.483309	-1.807393
C	0.376685	1.592195	-3.125040
C	0.480596	2.525414	-2.092661
C	0.632010	4.500674	-1.010609
H	0.704901	5.547666	-0.742862
C	0.693974	3.535331	1.316563
C	0.303133	0.204995	-2.992168
C	0.620515	3.409028	-0.071528
C	0.547816	3.954912	-2.259920
H	0.539694	4.468335	-3.213587
C	0.201681	-1.814740	-2.102705
C	0.145303	-0.728571	-4.079072
H	0.089750	-0.456034	-5.125931
C	0.080816	-1.975966	-3.529408
H	-0.039545	-2.925154	-4.037102
O	-1.607246	0.412040	0.183221
C	-3.084258	-1.436133	0.449418
C	-2.356930	-0.262765	0.992629
H	-1.990858	-0.383366	2.021830
C	-3.105960	-1.652062	-0.932938
C	-3.747658	-2.772642	-1.452522
C	-3.706660	-2.349495	1.306686
H	-3.678790	-2.187189	2.382235
C	-4.350431	-3.467344	0.784444
H	-4.829883	-4.177843	1.452459
C	-4.373304	-3.679491	-0.595616
H	-4.873683	-4.554965	-1.001207
H	7.426055	1.188331	0.294709
H	4.932916	1.319192	0.304143
H	-2.592899	-0.946437	-1.579022
H	-3.752411	-2.943428	-2.525885
C	-3.882754	2.194860	-0.527318
C	-4.420211	1.323899	0.499601
C	-3.722418	1.030743	1.669685
H	-2.983616	1.723687	2.062058
H	-4.235126	0.443968	2.428217
C	-5.654663	0.543587	0.191872
H	-6.469377	1.203088	-0.130400
H	-5.459392	-0.147234	-0.641454
H	-5.994742	-0.045030	1.045780
C	-4.614003	2.317726	-1.839788
H	-4.739311	1.343688	-2.327338
H	-5.616550	2.741820	-1.704380
H	-4.069228	2.969066	-2.528616
C	-2.687752	2.810714	-0.349653
H	-2.142947	2.805776	0.585156
H	-2.225272	3.374760	-1.155000

H	0.038473	-3.854890	-1.556824
H	0.771964	4.545078	1.709065
H	0.651989	-1.309359	4.276783
H	0.343078	1.986081	-4.136632

^{4}TS2A$_{endo}$

Fe	0.930260	0.717206	0.666917
N	1.664579	1.868290	-0.794919
N	2.599784	-0.379532	0.624064
C	3.699525	0.605812	-1.338740
C	2.795414	1.631565	-1.547215
C	1.861481	3.460226	-2.456361
H	1.617961	4.332596	-3.049844
C	-0.079200	3.594151	-0.905486
C	3.607724	-0.318628	-0.313599
C	1.086615	2.992292	-1.345092
C	2.921852	2.615399	-2.582386
H	3.730356	2.648418	-3.302061
C	2.911601	-1.452962	1.430067
C	4.559986	-1.366738	-0.092513
H	5.446358	-1.527157	-0.693538
C	4.128872	-2.069846	0.991647
H	4.587962	-2.927390	1.467571
N	0.381364	-0.216256	2.344554
N	-0.531636	2.054856	0.952995
C	-1.689285	1.004200	2.845855
C	-0.771352	-0.005489	3.068098
C	0.188231	-1.802104	4.011968
H	0.449094	-2.655286	4.625575
C	2.158253	-1.902365	2.499000
C	-1.561871	1.969555	1.865081
C	0.976006	-1.319205	2.915220
C	-0.899500	-0.989189	4.103806
H	-1.718434	-1.031702	4.810933
C	-0.826299	3.150589	0.170413
C	-2.507244	3.025506	1.652841
H	-3.403846	3.172601	2.241598
C	-2.048772	3.761194	0.603501
H	-2.488900	4.640473	0.149965
O	-0.123021	-0.453512	-0.489899
C	-2.463632	-0.347486	-0.925806
C	-1.055541	-0.041567	-1.287405
H	-0.912716	0.945399	-1.750501
C	-2.733304	-1.233718	0.122913
C	-4.050223	-1.490792	0.494620
C	-3.519061	0.282370	-1.593259
H	-3.306560	0.982266	-2.399320
C	-4.834699	0.022523	-1.220942
H	-5.652681	0.516079	-1.738838
C	-5.101989	-0.866619	-0.178032
H	-6.129638	-1.066892	0.113620
H	-0.426681	4.474177	-1.436548
H	-2.560398	1.048567	3.491217
H	2.516974	-2.768577	3.045086
H	4.549327	0.535644	-2.010210
H	-1.900587	-1.690362	0.649524
H	-4.257369	-2.173537	1.314704
C	0.088140	-2.997751	-1.990405
C	-0.879418	-2.233016	-2.749332
C	-0.691829	-0.881823	-3.041852
H	0.310128	-0.469764	-3.119211
H	-1.423253	-0.403634	-3.689016
C	-2.213636	-2.843164	-3.021196
H	-2.108657	-3.814347	-3.519520
H	-2.739979	-3.030452	-2.073811
H	-2.844479	-2.198257	-3.635706
C	-0.275691	-4.384898	-1.526318
H	-1.184056	-4.379725	-0.912242
H	-0.464138	-5.056929	-2.372516
H	0.530713	-4.820802	-0.930346
C	1.259235	-2.430486	-1.607255
H	1.601709	-1.465388	-1.956813
H	1.938256	-2.951994	-0.938282

^{6}TS2A$_{endo}$

Fe	-1.177084	-0.451906	-0.234358
N	-1.338970	0.619823	-2.000971
N	-2.536968	0.866267	0.599228
C	-2.632835	2.546802	-1.190303
C	-1.890151	1.872422	-2.154389
C	-0.870674	1.411995	-4.110366
H	-0.484673	1.427998	-5.122079
C	-0.096742	-0.893349	-3.488769
C	-2.946444	2.072727	0.079578
C	-0.728044	0.312770	-3.196105
C	-1.592648	2.374966	-3.467539
H	-1.913454	3.337257	-3.847046
C	-3.043567	0.800219	1.876741
C	-3.715518	2.789125	1.059766
H	-4.160326	3.764223	0.904436

C	-3.772084	2.003382	2.173488
H	-4.276173	2.204614	3.110745
N	-1.661770	-1.789161	1.255432
N	-0.497251	-2.051815	-1.362039
C	-0.524042	-3.782872	0.381198
C	-1.200891	-3.078712	1.371495
C	-2.192297	-2.611469	3.338625
H	-2.601541	-2.636779	4.340887
C	-2.901259	-0.279429	2.742369
C	-0.207034	-3.309782	-0.887356
C	-2.272805	-1.484879	2.448633
C	-1.530818	-3.598679	2.671037
H	-1.288239	-4.596283	3.015537
C	-0.014311	-1.999624	-2.648173
C	0.476732	-4.066033	-1.901294
H	0.811990	-5.089344	-1.786997
C	0.598362	-3.254857	-2.990091
H	1.052823	-3.480086	-3.946985
O	0.539438	0.176452	0.368921
C	2.887028	-0.204849	0.287626
C	1.614409	0.176763	-0.368163
H	1.506244	-0.173469	-1.401734
C	2.970906	-0.249396	1.683757
C	4.174534	-0.588044	2.296216
C	4.011632	-0.505306	-0.488055
H	3.944901	-0.471363	-1.573529
C	5.213172	-0.844851	0.127404
H	6.084088	-1.081643	-0.477713
C	5.296981	-0.883975	1.520158
H	6.234934	-1.149498	2.000370
H	0.337214	-0.993778	-4.479066
H	-0.224683	-4.799431	0.617275
H	-3.351246	-0.184915	3.726063
H	-3.013415	3.527479	-1.458987
H	2.086417	-0.025435	2.272184
H	4.236943	-0.628015	3.380466
C	1.516580	2.984440	1.204558
C	2.316231	2.673617	0.033727
C	1.787180	2.020759	-1.080141
H	0.732206	2.091643	-1.327186
H	2.437886	1.900983	-1.942984
C	3.793008	2.863065	0.114834
H	4.036363	3.890292	0.413183
H	4.215968	2.203743	0.886147
H	4.290805	2.644434	-0.831526
C	2.210616	3.422289	2.468597
H	2.953784	2.687020	2.798017
H	2.737988	4.373577	2.327567
H	1.491136	3.559772	3.280151
C	0.173906	2.810396	1.173005
H	-0.366665	2.571585	0.267239
H	-0.432569	2.949750	2.063555

BP86-D3

^{4}IA

Fe	0.000685	-0.007655	-0.031268
N	0.330121	-1.035376	1.655179
N	-0.358338	1.649614	1.040460
O	2.142185	0.619984	-0.126604
C	-0.029499	0.757136	3.324314
C	0.262821	-0.554198	2.958950
C	0.760800	-2.750904	3.169414
H	0.994001	-3.755326	3.526932
C	4.481171	0.148586	-0.004380
C	0.795734	-3.279875	0.722104
C	-0.312062	1.781912	2.425029
C	0.630732	-2.388977	1.780259
C	0.531526	-1.610930	3.901330
H	0.539173	-1.483697	4.985012
C	3.066900	-0.207132	0.012644
H	2.824420	-1.282579	0.160601
C	-0.661417	2.921495	0.561483
C	-0.597026	3.140652	2.811803
H	-0.622242	3.496920	3.842960
C	-0.814591	3.848550	1.654123
H	-1.055417	4.906673	1.538231
C	4.892636	1.493382	-0.174017
C	6.253940	1.802337	-0.180103
C	5.443524	-0.876136	0.156759
H	5.113022	-1.913600	0.287579
C	6.806448	-0.559288	0.148489
H	7.556072	-1.347668	0.271218
C	7.209003	0.777835	-0.018949
H	8.275988	1.026077	-0.024492
N	-0.337532	1.017805	-1.714724
N	0.369322	-1.661918	-1.100562
C	0.043844	-0.769097	-3.384628
C	-0.269451	0.537229	-3.018437
C	-0.801371	2.725961	-3.227120

H	-1.050614	3.726809	-3.583829
C	-0.810329	3.261144	-0.781485
C	0.336272	-1.791308	-2.485784
C	-0.656498	2.367129	-1.838439
C	-0.559170	1.589362	-3.959888
H	-0.570316	1.461658	-5.043478
C	0.670489	-2.933970	-0.621610
C	0.631703	-3.147769	-2.873075
H	0.668302	-3.501429	-3.904791
C	0.838653	-3.857808	-1.714930
H	1.080906	-4.915576	-1.598843
O	-2.143354	-0.624910	0.063776
C	-4.479410	-0.125951	0.105121
C	-3.062945	0.219460	0.076877
H	-2.810530	1.302437	0.069648
C	-4.899562	-1.478499	0.097522
C	-6.262848	-1.777243	0.131516
C	-5.434488	0.917254	0.148259
H	-5.096776	1.960562	0.154076
C	-6.799285	0.610479	0.184766
H	-7.543931	1.412371	0.218795
C	-7.210487	-0.734311	0.176262
H	-8.279021	-0.974451	0.203922
H	1.032722	-4.320563	0.961480
H	0.057480	-1.008304	-4.451893
H	-1.055154	4.299885	-1.021440
H	-0.038425	0.997428	4.391370
H	6.580979	2.839293	-0.309335
H	4.130957	2.270039	-0.295560
H	-4.142573	-2.268575	0.065152
H	-6.597027	-2.819855	0.124734

^{6}IA

Fe	0.000662	0.007553	0.031130
N	0.330303	1.035978	-1.654863
N	-0.358088	-1.649311	-1.041296
O	2.142232	-0.619716	0.126705
C	-0.028460	-0.756019	-3.324739
C	0.263538	0.555241	-2.958826
C	0.760755	2.752194	-3.168368
H	0.993656	3.756836	-3.525460
C	4.481211	-0.148296	0.004699
C	0.795039	3.280251	-0.720851
C	-0.311413	-1.781104	-2.425916
C	0.630472	2.389718	-1.779379
C	0.532193	1.612386	-3.900755
H	0.540205	1.485545	-4.984481
C	3.066940	0.207411	-0.012420
H	2.824446	1.282852	-0.160363
C	-0.661438	-2.921363	-0.562893
C	-0.596404	-3.139673	-2.813286
H	-0.621373	-3.495527	-3.844593
C	-0.814425	-3.847980	-1.655942
H	-1.055412	-4.906117	-1.540510
C	4.892674	-1.493095	0.174309
C	6.253983	-1.802030	0.180531
C	5.443565	0.876443	-0.156308
H	5.113061	1.913903	-0.287148
C	6.806492	0.559617	-0.147910
H	7.556119	1.348009	-0.270550
C	7.209046	-0.777506	0.019521
H	8.276034	-1.025731	0.025168
N	-0.337753	-1.018587	1.714133
N	0.369008	1.661413	1.101109
C	0.043597	0.767598	3.384795
C	-0.269649	-0.538581	3.018066
C	-0.801473	-2.727424	3.225815
H	-1.050650	-3.728447	3.582088
C	-0.810515	-3.261560	0.779931
C	0.335949	1.790201	2.486371
C	-0.656667	-2.367988	1.837279
C	-0.559307	-1.591132	3.959064
H	-0.570414	-1.463915	5.042709
C	0.669846	2.933737	0.622703
C	0.631113	3.146545	2.874252
H	0.667711	3.499742	3.906124
C	0.837825	3.857140	1.716412
H	1.079816	4.915013	1.600775
O	-2.143274	0.624823	-0.064293
C	-4.479363	0.125979	-0.105336
C	-3.062907	-0.219492	-0.077475
H	-2.810543	-1.302490	-0.070559
C	-4.899458	1.478541	-0.097558
C	-6.262744	1.777340	-0.131037
C	-5.434494	-0.917195	-0.148106
H	-5.096819	-1.960516	-0.154058
C	-6.799296	-0.610369	-0.184051
H	-7.543991	-1.412230	-0.217781
C	-7.210441	0.734441	-0.175389

H	-8.278976	0.974619	-0.202636
H	1.031683	4.321110	-0.959804
H	0.057221	1.006395	4.452146
H	-1.055365	-4.300394	1.019465
H	-0.036970	-0.995992	-4.391870
H	6.581024	-2.838986	0.309765
H	4.130990	-2.269770	0.295727
H	-4.142424	2.268581	-0.065471
H	-6.596889	2.819962	-0.124151

^{4}TS1A$_{endo}$

Fe	0.349389	0.180976	0.377224
N	0.052418	-1.394662	1.576872
N	0.759005	1.327581	1.984690
O	2.594417	-0.340574	0.432791
C	0.227533	-0.280870	3.780672
C	-0.015618	-1.385602	2.965512
C	-0.446872	-3.524228	2.364002
H	-0.672874	-4.591363	2.332927
C	4.698527	-0.613383	-0.670412
C	-0.242561	-3.180289	-0.109524
C	0.617033	0.975429	3.317088
C	-0.201881	-2.707634	1.200241
C	-0.341162	-2.701538	3.459339
H	-0.455291	-2.955203	4.514772
C	3.266730	-0.321782	-0.614945
H	2.772048	-0.068668	-1.579941
C	1.157750	2.654339	1.988781
C	0.938880	2.096543	4.171127
H	0.906710	2.073514	5.261803
C	1.285436	3.136869	3.345857
H	1.591955	4.148819	3.616304
C	5.422319	-0.934805	0.503297
C	6.789083	-1.208494	0.418637
C	5.355967	-0.570049	-1.921489
H	4.784703	-0.320663	-2.823821
C	6.725778	-0.847201	-2.000137
H	7.239393	-0.816460	-2.966544
C	7.439656	-1.165116	-0.831117
H	8.511899	-1.381819	-0.891982
N	0.777863	1.724346	-0.833558
N	0.162619	-1.016506	-1.232381
C	0.343627	0.671830	-3.030381
C	0.653933	1.762240	-2.217233
C	1.264945	3.857913	-1.616365

H	1.551103	4.910728	-1.587233
C	1.356702	3.440403	0.854539
C	0.142340	-0.627434	-2.566901
C	1.153759	3.007980	-0.455703
C	0.943610	3.087108	-2.709354
H	0.918487	3.376253	-3.761555
C	-0.083205	-2.382571	-1.239878
C	-0.108871	-1.767175	-3.417976
H	-0.170549	-1.724333	-4.506820
C	-0.237764	-2.857982	-2.594165
H	-0.437838	-3.896325	-2.863470
O	-1.573761	0.711984	0.408404
C	-3.216698	-1.037175	0.253821
C	-2.570738	0.092969	0.986560
H	-2.486655	-0.057527	2.082944
C	-3.074224	-1.126950	-1.145079
C	-3.641044	-2.202298	-1.841089
C	-3.935895	-2.026815	0.949647
H	-4.034925	-1.960667	2.040222
C	-4.501214	-3.104392	0.251742
H	-5.052225	-3.878884	0.796613
C	-4.354700	-3.193842	-1.143658
H	-4.794484	-4.037933	-1.686245
H	-0.455575	-4.242221	-0.260761
H	0.288436	0.842295	-4.109730
H	1.662156	4.480392	1.003386
H	0.143966	-0.421672	4.862483
H	7.356464	-1.456950	1.321705
H	4.893402	-0.962895	1.461403
H	-2.494570	-0.354263	-1.659603
H	-3.511802	-2.276000	-2.926173
C	-3.540855	2.447630	-1.016900
C	-4.388880	1.768438	-0.054072
C	-3.945407	1.499499	1.257142
H	-3.243056	2.180560	1.750149
H	-4.660154	1.026084	1.941100
C	-5.646223	1.107940	-0.541333
H	-6.291177	1.824412	-1.083475
H	-5.399644	0.300028	-1.259945
H	-6.226247	0.663138	0.281700
C	-3.945954	2.455497	-2.477900
H	-4.090295	1.429898	-2.865139
H	-4.899219	2.994070	-2.632907
H	-3.178076	2.947660	-3.096874

C	-2.331465	2.971376	-0.639177
H	-2.015091	3.080791	0.398638
H	-1.637946	3.364983	-1.389959

^{6}TS1A$_{endo}$

Fe	0.318405	0.210773	0.375050
N	0.190032	-1.269867	1.808526
N	0.847868	1.582941	1.838174
O	2.546826	-0.301135	0.397271
C	0.496697	0.143543	3.818588
C	0.231795	-1.080323	3.179698
C	-0.162733	-3.296134	2.862305
H	-0.347478	-4.365107	2.984190
C	4.632714	-0.649287	-0.714931
C	-0.155262	-3.220893	0.326535
C	0.799109	1.370181	3.202786
C	-0.038941	-2.616780	1.589137
C	-0.001642	-2.345256	3.845813
H	-0.024748	-2.487477	4.927887
C	3.203003	-0.351641	-0.660623
H	2.694719	-0.160758	-1.632063
C	1.207403	2.902362	1.643473
C	1.135666	2.600091	3.894909
H	1.174190	2.716720	4.979591
C	1.395526	3.546117	2.930389
H	1.685282	4.589205	3.070926
C	5.363510	-0.934465	0.463944
C	6.729327	-1.212487	0.379037
C	5.282129	-0.645257	-1.970977
H	4.705428	-0.423134	-2.876885
C	6.651941	-0.922364	-2.049059
H	7.160095	-0.919268	-3.018809
C	7.372509	-1.205877	-0.875293
H	8.444402	-1.424358	-0.936028
N	0.732917	1.624683	-1.082604
N	0.153537	-1.234747	-1.115646
C	0.238638	0.256508	-3.087151
C	0.545146	1.470751	-2.447079
C	1.128324	3.645767	-2.133834
H	1.383623	4.699977	-2.257949
C	1.326329	3.527429	0.390823
C	0.075404	-0.999443	-2.477185
C	1.093539	2.945309	-0.866737
C	0.778260	2.736949	-3.109747
H	0.700347	2.904219	-4.185841
C	-0.075328	-2.585136	-0.922579
C	-0.200767	-2.244903	-3.166786
H	-0.311111	-2.349944	-4.247877
C	-0.288480	-3.225888	-2.205598
H	-0.492437	-4.289029	-2.345017
O	-1.587748	0.636323	0.430952
C	-3.287538	-1.061781	0.298706
C	-2.596703	0.044318	1.018707
H	-2.510862	-0.093884	2.115275
C	-3.107942	-1.212773	-1.091073
C	-3.737010	-2.266219	-1.767370
C	-4.111189	-1.961662	1.002442
H	-4.245900	-1.841937	2.084520
C	-4.743322	-3.012056	0.322234
H	-5.378778	-3.714564	0.872406
C	-4.556011	-3.166751	-1.062781
H	-5.046530	-3.991157	-1.591736
H	-0.356151	-4.297022	0.315949
H	0.141805	0.288679	-4.177484
H	1.605695	4.586165	0.395272
H	0.503169	0.131594	4.913666
H	7.301693	-1.435381	1.285605
H	4.840660	-0.932636	1.425747
H	-2.455345	-0.508760	-1.615875
H	-3.577963	-2.392235	-2.843554
C	-3.495632	2.424038	-0.993393
C	-4.359283	1.770670	-0.023245
C	-3.931904	1.513247	1.293554
H	-3.201625	2.170144	1.777998
H	-4.657442	1.063535	1.981706
C	-5.623048	1.123594	-0.507897
H	-6.240125	1.831599	-1.091235
H	-5.378141	0.284170	-1.191047
H	-6.228197	0.721536	0.318742
C	-3.887315	2.405662	-2.457766
H	-4.050641	1.374214	-2.820475
H	-4.825818	2.962365	-2.636954
H	-3.100629	2.863632	-3.079195
C	-2.288479	2.950379	-0.616788
H	-1.970419	3.063377	0.420879
H	-1.593016	3.335606	-1.369708

^{4}TS2A$_{endo}$

Fe	0.865364	0.646006	0.776102
N	1.323477	2.212319	-0.393017

N	2.692486	-0.110309	0.457723
C	3.514479	1.465888	-1.264745
C	2.442364	2.351827	-1.210599
C	1.159358	4.157523	-1.665227
H	0.736691	5.089316	-2.044794
C	-0.714716	3.585081	-0.105937
C	3.630523	0.324329	-0.476210
C	0.529197	3.321477	-0.674318
C	2.346107	3.553733	-2.000503
H	3.103652	3.887749	-2.711418
C	3.232719	-1.274201	0.998704
C	4.756358	-0.574296	-0.519187
H	5.623909	-0.446137	-1.168501
C	4.511135	-1.565341	0.399899
H	5.135319	-2.421262	0.661981
N	0.569797	-0.722399	2.215985
N	-0.794952	1.602785	1.372954
C	-1.662213	-0.024914	3.021219
C	-0.564588	-0.879866	3.007524
C	0.752054	-2.644535	3.519392
H	1.195659	-3.552808	3.930762
C	2.626179	-2.071855	1.964214
C	-1.761795	1.134533	2.259961
C	1.381352	-1.808021	2.527330
C	-0.458352	-2.069404	3.815707
H	-1.219169	-2.405601	4.521985
C	-1.328460	2.776986	0.846204
C	-2.900637	2.016468	2.282063
H	-3.789705	1.859401	2.894436
C	-2.630533	3.038484	1.406519
H	-3.249460	3.900414	1.152383
O	-0.014947	-0.366117	-0.639267
C	-2.383095	-0.248986	-1.029676
C	-0.981788	0.117935	-1.379148
H	-0.853310	1.159295	-1.739606
C	-2.626279	-1.256430	-0.073333
C	-3.941795	-1.570366	0.294246
C	-3.466050	0.439788	-1.612865
H	-3.273082	1.231040	-2.348030
C	-4.780102	0.121889	-1.245042
H	-5.619660	0.661703	-1.696168
C	-5.019843	-0.885086	-0.292576
H	-6.047837	-1.130664	-0.004572
H	-1.243624	4.487070	-0.427049
H	-2.499620	-0.277550	3.677764
H	3.162568	-2.963517	2.301335
H	4.330194	1.692678	-1.957592
H	-1.770506	-1.755877	0.391054
H	-4.126812	-2.346421	1.045200
C	0.349973	-2.709007	-2.418565
C	-0.708200	-1.953865	-3.074039
C	-0.630608	-0.559592	-3.243206
H	0.343718	-0.066224	-3.326803
H	-1.430538	-0.071181	-3.812353
C	-2.021231	-2.631748	-3.333650
H	-1.890678	-3.590656	-3.867077
H	-2.512219	-2.871651	-2.366902
H	-2.710339	-1.994805	-3.908351
C	0.117660	-4.171617	-2.099149
H	-0.782214	-4.313057	-1.472663
H	-0.033485	-4.768423	-3.017376
H	0.977798	-4.600695	-1.559954
C	1.489685	-2.083935	-1.987689
H	1.746721	-1.051501	-2.228505
H	2.227320	-2.621881	-1.383118

^{6}TS2A$_{endo}$

Fe	0.839108	0.589219	0.659114
N	1.388599	2.246508	-0.467274
N	2.759404	-0.147807	0.418292
C	3.558389	1.419460	-1.323730
C	2.504936	2.342320	-1.288045
C	1.234531	4.160256	-1.769688
H	0.826756	5.090244	-2.169961
C	-0.643178	3.625095	-0.160791
C	3.682627	0.269380	-0.531812
C	0.597999	3.351984	-0.752595
C	2.414026	3.535351	-2.101290
H	3.165340	3.852941	-2.826507
C	3.275971	-1.306652	0.983200
C	4.793902	-0.654454	-0.573492
H	5.658216	-0.559132	-1.233029
C	4.542812	-1.629492	0.365227
H	5.161782	-2.489672	0.626663
N	0.616009	-0.685963	2.283124
N	-0.764227	1.693766	1.381661
C	-1.579327	0.110637	3.100143
C	-0.501699	-0.784817	3.100253
C	0.802610	-2.562634	3.633793

H	1.233611	-3.467149	4.066795
C	2.654553	-2.057044	1.990002
C	-1.702717	1.259531	2.309649
C	1.424877	-1.768709	2.596377
C	-0.391557	-1.955191	3.943681
H	-1.134313	-2.262464	4.681962
C	-1.275905	2.859506	0.826930
C	-2.829609	2.164768	2.327930
H	-3.708451	2.050092	2.964403
C	-2.565329	3.155598	1.411581
H	-3.183049	4.016930	1.151508
O	-0.086380	-0.355612	-0.715238
C	-2.463082	-0.248201	-1.047734
C	-1.076419	0.096097	-1.445089
H	-0.941958	1.101679	-1.890242
C	-2.686126	-1.181249	-0.012219
C	-3.994483	-1.466970	0.399648
C	-3.558041	0.392315	-1.665849
H	-3.379719	1.124741	-2.462878
C	-4.863715	0.099624	-1.253614
H	-5.712566	0.600482	-1.731040
C	-5.083584	-0.831694	-0.222120
H	-6.105968	-1.056422	0.100447
H	-1.163798	4.527895	-0.495662
H	-2.400771	-0.103507	3.791201
H	3.182181	-2.950050	2.339691
H	4.372748	1.625431	-2.025985
H	-1.823606	-1.644217	0.477714
H	-4.165411	-2.182160	1.211575
C	0.269597	-2.799977	-2.336082
C	-0.814814	-2.092873	-3.011256
C	-0.743623	-0.724702	-3.309861
H	0.224005	-0.227419	-3.435068
H	-1.564076	-0.277264	-3.882234
C	-2.134123	-2.788433	-3.169793
H	-2.018730	-3.782486	-3.638380
H	-2.586877	-2.962155	-2.170803
H	-2.846902	-2.196320	-3.762909
C	0.044488	-4.231983	-1.894827
H	-0.823701	-4.316420	-1.215339
H	-0.154997	-4.896202	-2.755776
H	0.927645	-4.625159	-1.365909
C	1.432843	-2.158289	-2.008792
H	1.680805	-1.145208	-2.330315
H	2.196567	-2.658713	-1.404017

M06L

^{4}IA

Fe	-0.002547	-0.004072	0.085380
N	-0.202143	-1.108287	-1.575576
N	-0.323037	-1.638365	1.198439
O	2.176714	-0.472334	0.061253
C	-0.579013	-3.317036	-0.573811
C	-0.424454	-2.465820	-1.655813
C	-0.281461	-1.762903	-3.782597
H	-0.251595	-1.676220	-4.863147
C	4.496248	-0.024479	-0.125052
C	0.143064	0.636193	-3.268678
C	-0.528411	-2.927563	0.754745
C	-0.103567	-0.669527	-2.877968
C	-0.479766	-2.877615	-3.024737
H	-0.646261	-3.898287	-3.352214
C	3.094405	0.338912	-0.094366
H	2.862478	1.417385	-0.226199
C	-0.349193	-1.710289	2.574238
C	-0.692606	-3.812596	1.866009
H	-0.867150	-4.879723	1.780547
C	-0.583933	-3.057324	2.994675
H	-0.648420	-3.374380	4.029923
C	4.894290	-1.365692	0.002439
C	6.240193	-1.688112	-0.037490
C	5.458417	0.983110	-0.290652
H	5.135877	2.019772	-0.388694
C	6.806571	0.654408	-0.327279
H	7.556845	1.431392	-0.453689
C	7.194114	-0.679212	-0.201719
H	8.250866	-0.937722	-0.231782
N	0.195542	1.097594	1.743295
N	0.316763	1.630800	-1.031320
C	0.593303	3.303476	0.742200
C	0.433147	2.452647	1.822928
C	0.278885	1.752839	3.949972
H	0.246371	1.666869	5.030470
C	-0.161953	-0.643264	3.436489
C	0.531400	2.917829	-0.587163
C	0.092257	0.660670	3.045559
C	0.491128	2.864542	3.191852
H	0.668547	3.883574	3.518608
C	0.333931	1.703696	-2.407262

C	0.695487	3.803064	-1.698339
H	0.877439	4.868993	-1.612988
C	0.574091	3.049904	-2.827432
H	0.633862	3.368225	-3.862593
O	-2.175596	0.493233	0.061186
C	-4.491929	0.039272	-0.142552
C	-3.090376	-0.322585	-0.086669
H	-2.857184	-1.403967	-0.187084
C	-4.890255	1.382736	-0.042484
C	-6.235411	1.705285	-0.101472
C	-5.453024	-0.970464	-0.300945
H	-5.130512	-2.008917	-0.376813
C	-6.800435	-0.641442	-0.357845
H	-7.549981	-1.419962	-0.479093
C	-7.188312	0.694326	-0.258682
H	-8.244583	0.952824	-0.303545
H	0.189129	0.838993	-4.336078
H	0.773669	4.354957	0.953043
H	-0.212585	-0.845708	4.503692
H	-0.748336	-4.370693	-0.783159
H	6.556949	-2.724402	0.057461
H	4.132222	-2.131776	0.127538
H	-4.128946	2.150217	0.078705
H	-6.552284	2.743131	-0.026740

^{6}IA

Fe	-0.003612	-0.005343	0.084155
N	-0.232283	-1.223801	-1.556299
N	-0.320048	-1.620431	1.307452
O	2.131730	-0.469301	0.052083
C	-0.580911	-3.364457	-0.404147
C	-0.443836	-2.582608	-1.552380
C	-0.323689	-1.980174	-3.719801
H	-0.304149	-1.958543	-4.804255
C	4.449540	-0.022303	-0.124978
C	0.090531	0.467003	-3.312351
C	-0.521400	-2.925597	0.918943
C	-0.145961	-0.835903	-2.873071
C	-0.505609	-3.059812	-2.903150
H	-0.665915	-4.094281	-3.188450
C	3.052229	0.345479	-0.092681
H	2.819656	1.423746	-0.210805
C	-0.317573	-1.608666	2.682868
C	-0.660458	-3.753170	2.082055
H	-0.829294	-4.824729	2.064801
C	-0.537145	-2.938538	3.171622
H	-0.581476	-3.213940	4.219905
C	4.844604	-1.365908	-0.005829
C	6.189668	-1.690075	-0.047497
C	5.413826	0.985256	-0.284764
H	5.093261	2.022959	-0.376246
C	6.760814	0.653856	-0.324103
H	7.512713	1.429786	-0.446528
C	7.145256	-0.681555	-0.206283
H	8.201466	-0.941957	-0.238043
N	0.228643	1.208315	1.717794
N	0.312902	1.608786	-1.148228
C	0.606040	3.344648	0.565663
C	0.464185	2.563813	1.712798
C	0.333835	1.963983	3.880631
H	0.314199	1.943061	4.965065
C	-0.111915	-0.477997	3.473348
C	0.526640	2.910900	-0.758976
C	0.137801	0.822268	3.034665
C	0.535160	3.039695	3.063639
H	0.713889	4.071189	3.348525
C	0.297856	1.598801	-2.523295
C	0.662116	3.739770	-1.921675
H	0.839635	4.809948	-1.904204
C	0.521503	2.928189	-3.011799
H	0.558426	3.205544	-4.059857
O	-2.133521	0.486174	0.062777
C	-4.451215	0.044397	-0.130839
C	-3.055303	-0.326953	-0.082953
H	-2.827550	-1.407531	-0.187350
C	-4.843129	1.390279	-0.027875
C	-6.186966	1.717759	-0.081862
C	-5.417274	-0.962029	-0.287015
H	-5.099209	-2.001547	-0.365211
C	-6.762986	-0.627299	-0.338593
H	-7.516270	-1.402357	-0.457969
C	-7.144368	0.710329	-0.236791
H	-8.199634	0.973309	-0.277911
H	0.118473	0.616963	-4.389948
H	0.784300	4.407373	0.718814
H	-0.144416	-0.628154	4.550714
H	-0.743572	-4.429853	-0.555948
H	6.504804	-2.727272	0.041537
H	4.081552	-2.131626	0.114756

H	-4.078616	2.154850	0.090872
H	-6.499725	2.756630	-0.005354

^{4}TS1A$_{endo}$

Fe	0.390911	0.166384	0.363143
N	0.148949	-1.458785	1.514318
N	0.673835	1.289690	2.006202
O	2.662475	-0.255341	0.448894
C	0.261196	-0.405535	3.728391
C	0.089688	-1.495334	2.888500
C	-0.244933	-3.613573	2.222707
H	-0.421481	-4.681836	2.158204
C	4.770691	-0.530916	-0.618733
C	-0.074838	-3.175357	-0.226151
C	0.548909	0.886052	3.313950
C	-0.047375	-2.754837	1.094202
C	-0.165430	-2.831554	3.335321
H	-0.257920	-3.124013	4.375951
C	3.346495	-0.251047	-0.575092
H	2.872768	-0.023166	-1.555024
C	0.974330	2.630133	2.051325
C	0.771958	1.993862	4.195555
H	0.732253	1.936280	5.277877
C	1.042045	3.074422	3.412282
H	1.268505	4.091138	3.715204
C	5.491590	-0.843717	0.545190
C	6.848338	-1.110243	0.459825
C	5.422745	-0.489315	-1.859265
H	4.849366	-0.245795	-2.753866
C	6.782239	-0.757379	-1.939883
H	7.291555	-0.726696	-2.900504
C	7.491930	-1.067177	-0.780387
H	8.557997	-1.278737	-0.840907
N	0.770623	1.764767	-0.795273
N	0.291423	-0.993231	-1.285140
C	0.463816	0.744580	-3.007291
C	0.708145	1.822867	-2.168827
C	1.190340	3.914002	-1.504029
H	1.425929	4.971164	-1.441766
C	1.164988	3.447002	0.947560
C	0.284666	-0.566902	-2.593236
C	1.059980	3.042970	-0.375008
C	0.962317	3.159227	-2.616484
H	0.976001	3.467377	-3.656520
C	0.069633	-2.349397	-1.329795
C	0.073922	-1.678619	-3.473227
H	0.031947	-1.607608	-4.554799
C	-0.051054	-2.785107	-2.689366
H	-0.226456	-3.812697	-2.989938
O	-1.608394	0.544681	0.268351
C	-3.330138	-1.109272	0.183128
C	-2.593398	-0.019847	0.881367
H	-2.470283	-0.204597	1.966710
C	-3.195099	-1.272923	-1.198895
C	-3.856412	-2.311914	-1.846156
C	-4.134939	-1.992546	0.907167
H	-4.228516	-1.870413	1.987808
C	-4.801570	-3.026853	0.258340
H	-5.425206	-3.713120	0.828410
C	-4.663170	-3.187640	-1.119931
H	-5.181232	-3.999301	-1.627270
H	-0.251524	-4.232534	-0.411199
H	0.436863	0.939694	-4.077198
H	1.399588	4.492630	1.134016
H	0.189492	-0.583360	4.799081
H	7.414443	-1.354280	1.356133
H	4.967985	-0.872946	1.498709
H	-2.553239	-0.582884	-1.747293
H	-3.737617	-2.443361	-2.920888
C	-3.581172	2.478808	-0.952428
C	-4.371159	1.738689	0.003078
C	-3.861671	1.382690	1.256161
H	-3.124909	2.022513	1.738813
H	-4.558301	0.916271	1.953172
C	-5.654253	1.139308	-0.448502
H	-6.317863	1.900307	-0.880490
H	-5.482181	0.403518	-1.249894
H	-6.185743	0.634842	0.362726
C	-4.063518	2.589080	-2.369163
H	-4.231007	1.602416	-2.821286
H	-5.017111	3.128504	-2.437495
H	-3.336944	3.121938	-2.990433
C	-2.373585	2.986590	-0.598620
H	-2.004931	2.991742	0.421985
H	-1.729861	3.459546	-1.339924

^{6}TS1A$_{endo}$

Fe	0.355638	0.159272	0.354429
N	0.160513	-1.488481	1.572648
N	0.729230	1.335160	2.002736

O	2.592571	-0.279778	0.440973
C	0.354060	-0.353876	3.743859
C	0.140632	-1.480505	2.945874
C	-0.247448	-3.613383	2.330036
H	-0.443605	-4.680119	2.303839
C	4.701053	-0.551149	-0.623131
C	-0.116005	-3.212825	-0.154269
C	0.639035	0.943990	3.314600
C	-0.063012	-2.783235	1.172850
C	-0.123187	-2.807666	3.425489
H	-0.194254	-3.086503	4.471680
C	3.279074	-0.267792	-0.583395
H	2.805932	-0.027219	-1.558745
C	1.045805	2.669791	1.998504
C	0.896836	2.072699	4.166587
H	0.890153	2.046418	5.251215
C	1.151568	3.138214	3.353144
H	1.394069	4.156067	3.640028
C	5.418635	-0.869834	0.541593
C	6.775232	-1.137033	0.458282
C	5.356220	-0.504675	-1.862220
H	4.785208	-0.256482	-2.756958
C	6.715404	-0.774254	-1.940455
H	7.227182	-0.740148	-2.899608
C	7.421785	-1.089381	-0.780318
H	8.487840	-1.301489	-0.839014
N	0.819015	1.744941	-0.879376
N	0.293463	-1.081884	-1.306019
C	0.499442	0.635869	-3.048369
C	0.753756	1.755652	-2.251938
C	1.242013	3.867978	-1.633417
H	1.477872	4.926806	-1.607387
C	1.224549	3.444737	0.850520
C	0.293387	-0.677100	-2.617877
C	1.113627	3.025589	-0.477420
C	1.011603	3.084823	-2.729518
H	1.026008	3.377793	-3.774115
C	0.037871	-2.430352	-1.300316
C	0.047443	-1.810762	-3.467502
H	-0.000773	-1.776288	-4.550931
C	-0.105096	-2.894466	-2.652735
H	-0.311459	-3.921489	-2.935606
O	-1.602501	0.514823	0.274326
C	-3.366680	-1.088110	0.181954
C	-2.588453	-0.045152	0.888274
H	-2.473093	-0.224835	1.972801
C	-3.237294	-1.244419	-1.202431
C	-3.933837	-2.254590	-1.856837
C	-4.202699	-1.947756	0.901129
H	-4.290780	-1.831573	1.982502
C	-4.905034	-2.951415	0.243547
H	-5.552195	-3.620352	0.807606
C	-4.771333	-3.105915	-1.136207
H	-5.316358	-3.896039	-1.649135
H	-0.317841	-4.270720	-0.312656
H	0.475948	0.802798	-4.124003
H	1.468467	4.494111	1.007002
H	0.315477	-0.510222	4.820480
H	7.339140	-1.384835	1.354906
H	4.892982	-0.902311	1.493833
H	-2.570734	-0.574578	-1.745648
H	-3.818080	-2.383246	-2.931920
C	-3.523349	2.551939	-0.890889
C	-4.325098	1.806848	0.058732
C	-3.837597	1.441290	1.310623
H	-3.059567	2.030591	1.792155
H	-4.537378	0.975420	2.003961
C	-5.612567	1.227115	-0.405963
H	-6.257161	1.994113	-0.854791
H	-5.436401	0.481463	-1.197886
H	-6.163791	0.736363	0.400463
C	-4.006761	2.681530	-2.305479
H	-4.189072	1.701613	-2.766085
H	-4.952855	3.234737	-2.367676
H	-3.273327	3.209133	-2.922972
C	-2.318369	3.056180	-0.531260
H	-1.942836	3.032661	0.487108
H	-1.678694	3.547200	-1.263793

^{4}TS2A$_{endo}$

Fe	1.111959	0.807792	0.304523
N	1.154559	1.742090	-1.473906
N	2.752780	-0.216617	-0.219873
C	2.958584	0.486403	-2.564549
C	1.952727	1.436841	-2.558129
C	0.573331	3.051749	-3.282367
H	0.043577	3.806646	-3.853311
C	-0.690895	3.330417	-1.156384
C	3.335963	-0.272592	-1.470416

C	0.303724	2.736895	-1.914540
C	1.594252	2.243179	-3.682437
H	2.079227	2.193811	-4.651380
C	3.459992	-1.113938	0.554120
C	4.406003	-1.220860	-1.477915
H	5.020720	-1.445810	-2.342594
C	4.483572	-1.741362	-0.221696
H	5.177414	-2.480893	0.162792
N	1.339315	0.164232	2.189620
N	-0.265042	2.124204	0.937065
C	-0.495752	1.387731	3.265863
C	0.545369	0.476576	3.273221
C	1.993981	-1.059216	4.031642
H	2.570389	-1.762107	4.623340
C	3.217935	-1.382899	1.889342
C	-0.869952	2.153987	2.176067
C	2.230877	-0.780786	2.648833
C	0.945870	-0.280909	4.418258
H	0.479908	-0.207694	5.394810
C	-0.954109	3.040075	0.170689
C	-1.947719	3.093913	2.181155
H	-2.574655	3.304269	3.040585
C	-1.997415	3.646634	0.938329
H	-2.677392	4.401645	0.559410
O	-0.274593	-0.563705	-0.167941
C	-2.639770	-0.397624	-0.493662
C	-1.243814	-0.361387	-1.001919
H	-1.083666	0.414264	-1.774044
C	-2.924592	-0.929871	0.767630
C	-4.231431	-0.931600	1.243881
C	-3.675719	0.132984	-1.268780
H	-3.450180	0.560294	-2.247839
C	-4.982150	0.126892	-0.792730
H	-5.783673	0.545212	-1.398657
C	-5.261924	-0.408339	0.463974
H	-6.283913	-0.411152	0.838047
H	-1.303902	4.089261	-1.636905
H	-1.051220	1.523930	4.190891
H	3.856562	-2.114141	2.379366
H	3.507846	0.340733	-3.491881
H	-2.107291	-1.320907	1.372700
H	-4.448294	-1.339066	2.229791
C	-0.650951	-3.622719	-0.821027
C	-1.497224	-2.905489	-1.751309
C	-1.086981	-1.727356	-2.378338
H	-0.028713	-1.551384	-2.562544
H	-1.739368	-1.338130	-3.159772
C	-2.916255	-3.325098	-1.879835
H	-2.987203	-4.372475	-2.204318
H	-3.430841	-3.280047	-0.908038
H	-3.470243	-2.704789	-2.588999
C	-1.228416	-4.783625	-0.066781
H	-2.092865	-4.488714	0.542518
H	-1.580781	-5.573850	-0.742058
H	-0.486489	-5.227509	0.602992
C	0.616378	-3.207147	-0.579321
H	1.097727	-2.403207	-1.127055
H	1.220911	-3.697273	0.182514

^{6}TS2A$_{endo}$

Fe	1.020745	0.757174	-0.042302
N	1.617824	0.510005	-2.005206
N	2.765557	-0.164778	0.548772
C	3.522510	-0.995955	-1.634794
C	2.612657	-0.333013	-2.453133
C	1.557826	0.397288	-4.302000
H	1.237954	0.599640	-5.318681
C	-0.089910	1.878471	-3.118933
C	3.609326	-0.907535	-0.248073
C	0.970923	0.976032	-3.129391
C	2.574156	-0.411047	-3.883693
H	3.250938	-1.004215	-4.489339
C	3.203892	-0.337157	1.843695
C	4.585577	-1.570476	0.564909
H	5.369707	-2.217212	0.185837
C	4.335461	-1.216795	1.858782
H	4.876511	-1.513597	2.750938
N	0.879770	1.569235	1.856830
N	-0.247255	2.263293	-0.699622
C	-0.988724	3.114674	1.481757
C	-0.085987	2.443246	2.300460
C	1.007636	1.763711	4.144784
H	1.367192	1.606034	5.156025
C	2.633968	0.259977	2.963895
C	-1.063770	3.038874	0.094568
C	1.561773	1.146870	2.974351
C	-0.013017	2.565466	3.727514
H	-0.659197	3.196311	4.328249
C	-0.651438	2.484018	-1.997775

C	-2.000772	3.752934	-0.721900
H	-2.753052	4.436606	-0.343824
C	-1.746094	3.409741	-2.016812
H	-2.248047	3.756115	-2.914074
O	-0.327349	-0.658371	0.099552
C	-2.696993	-0.562072	-0.167976
C	-1.335787	-0.725029	-0.710526
H	-1.197294	-0.321199	-1.728969
C	-2.919372	-0.598461	1.214080
C	-4.201117	-0.402427	1.714699
C	-3.767009	-0.327627	-1.038805
H	-3.584768	-0.285545	-2.113975
C	-5.047680	-0.136941	-0.534826
H	-5.877064	0.050181	-1.213736
C	-5.265846	-0.177603	0.842220
H	-6.268457	-0.026585	1.237420
H	-0.507209	2.154864	-4.085420
H	-1.699417	3.775487	1.974003
H	3.085372	0.025524	3.926011
H	4.259386	-1.624473	-2.131355
H	-2.073050	-0.758689	1.881533
H	-4.373098	-0.421018	2.789173
C	-0.571962	-3.768980	0.520298
C	-1.546252	-3.408340	-0.495121
C	-1.246211	-2.564929	-1.556375
H	-0.219546	-2.442203	-1.896481
H	-1.995293	-2.452641	-2.339094
C	-2.960036	-3.814664	-0.285567
H	-3.045054	-4.903821	-0.175086
H	-3.355267	-3.390159	0.650080
H	-3.609933	-3.498717	-1.105564
C	-1.019714	-4.611451	1.677933
H	-1.823817	-4.128280	2.248325
H	-1.412802	-5.581465	1.347358
H	-0.193488	-4.808022	2.367088
C	0.697403	-3.302907	0.459337
H	1.078912	-2.717954	-0.372510
H	1.405534	-3.516183	1.258575

OLYP-D3

^{4}IA

Fe	-0.002742	-0.002693	0.104423
N	0.356795	-0.709246	1.940622
N	-0.412705	1.802434	0.869395
O	2.190856	0.625745	-0.092100
C	-0.112302	1.316812	3.271044
C	0.260049	-0.015841	3.136689
C	0.915531	-2.098318	3.713828
H	1.220024	-3.004375	4.229050
C	4.518580	0.153209	-0.309822
C	0.938040	-3.046444	1.400365
C	-0.417182	2.159651	2.208898
C	0.749765	-1.992666	2.287208
C	0.607774	-0.870997	4.242178
H	0.608684	-0.557398	5.281839
C	3.103801	-0.200079	-0.251688
H	2.856268	-1.271615	-0.357251
C	-0.719810	2.963156	0.175636
C	-0.749181	3.553106	2.355824
H	-0.819626	4.068660	3.308870
C	-0.931547	4.053626	1.092179
H	-1.182995	5.066293	0.790789
C	4.939183	1.496604	-0.176309
C	6.298072	1.803907	-0.236010
C	5.470349	-0.873004	-0.502149
H	5.133407	-1.906215	-0.603026
C	6.831079	-0.558724	-0.561659
H	7.570095	-1.346538	-0.709723
C	7.242483	0.777599	-0.428404
H	8.304484	1.023087	-0.473854
N	-0.350084	0.710197	-1.739085
N	0.386687	-1.811759	-0.665154
C	-0.007092	-1.348954	-3.058292
C	-0.300303	0.004297	-2.931357
C	-0.796277	2.127352	-3.522144
H	-1.031323	3.050275	-4.043911
C	-0.824506	3.073386	-1.206559
C	0.314324	-2.188236	-1.997820
C	-0.651034	2.016570	-2.093980
C	-0.583421	0.876106	-4.042203
H	-0.606044	0.557491	-5.080176
C	0.747017	-2.959705	0.025853
C	0.641790	-3.582672	-2.142789
H	0.656362	-4.112884	-3.090268
C	0.914380	-4.060856	-0.886127
H	1.197061	-5.065924	-0.587045
O	-2.194897	-0.631471	0.303024
C	-4.508009	-0.172158	-0.065306
C	-3.083425	0.142925	-0.087928

H	-2.804460	1.135684	-0.482471
C	-4.974091	-1.417014	0.417762
C	-6.342096	-1.687683	0.419861
C	-5.423588	0.791525	-0.543409
H	-5.051555	1.748094	-0.914779
C	-6.793284	0.514224	-0.539280
H	-7.503951	1.254203	-0.907935
C	-7.250181	-0.723658	-0.058035
H	-8.319234	-0.941000	-0.054997
H	1.237979	-4.008632	1.811797
H	-0.015276	-1.777648	-4.058784
H	-1.066706	4.050106	-1.621882
H	-0.150667	1.733724	4.275813
H	6.630575	2.837360	-0.133648
H	4.187402	2.270918	-0.027969
H	-4.250811	-2.146672	0.780053
H	-6.710258	-2.644830	0.790331

^{6}IA

Fe	-0.000423	0.000975	0.001274
N	0.478916	-1.341836	1.490952
N	-0.278098	1.479094	1.409773
O	2.134832	0.640799	-0.165819
C	0.239784	0.163020	3.437694
C	0.507450	-1.079901	2.848273
C	1.007557	-3.281639	2.625367
H	1.259228	-4.325644	2.788647
C	4.467901	0.156259	-0.131257
C	0.836273	-3.352692	0.101621
C	-0.114874	1.346487	2.776284
C	0.772354	-2.683910	1.331742
C	0.843696	-2.288746	3.563870
H	0.935220	-2.363119	4.643707
C	3.055082	-0.194175	-0.092829
H	2.809643	-1.264173	0.012571
C	-0.600408	2.801501	1.166962
C	-0.356096	2.619810	3.414499
H	-0.296369	2.796912	4.484476
C	-0.655265	3.520545	2.418437
H	-0.888825	4.577011	2.515098
C	4.882631	1.502100	-0.267928
C	6.243123	1.805615	-0.286784
C	5.427357	-0.875127	-0.014456
H	5.094852	-1.909094	0.091174
C	6.789405	-0.563574	-0.032431
H	7.534622	-1.354165	0.057819
C	7.194732	0.774581	-0.167727
H	8.258102	1.017832	-0.181141
N	-0.479287	1.344211	-1.488251
N	0.278009	-1.476420	-1.407380
C	-0.240647	-0.160510	-3.435157
C	-0.508137	1.082283	-2.845557
C	-1.007639	3.284032	-2.622672
H	-1.258800	4.328108	-2.786058
C	-0.834769	3.355566	-0.099292
C	0.114549	-1.343894	-2.773917
C	-0.772228	2.686343	-1.329135
C	-0.844325	2.291087	-3.561131
H	-0.936201	2.365398	-4.640935
C	0.601815	-2.798624	-1.164689
C	0.356885	-2.617014	-3.412145
H	0.297055	-2.794264	-4.482137
C	0.657513	-3.517387	-2.416293
H	0.892338	-4.573609	-2.513059
O	-2.135098	-0.640341	0.170057
C	-4.468477	-0.159377	0.129650
C	-3.056228	0.193300	0.095679
H	-2.812174	1.263786	-0.007780
C	-4.881329	-1.505634	0.267987
C	-6.241314	-1.811593	0.281780
C	-5.429242	0.869960	0.006019
H	-5.098123	1.904256	-0.100738
C	-6.790776	0.555900	0.018576
H	-7.537059	1.344859	-0.077155
C	-7.194213	-0.782618	0.155743
H	-8.257181	-1.027754	0.165178
H	1.082519	-4.413671	0.133072
H	-0.317598	-0.212075	-4.520727
H	-1.079585	4.416823	-0.130922
H	0.316342	0.214591	4.523279
H	6.571727	2.839997	-0.391229
H	4.126006	2.281014	-0.353754
H	-4.123694	-2.282941	0.359417
H	-6.568544	-2.846270	0.387815

^{4}TS1A$_{endo}$

Fe	0.356526	0.106827	0.294415
N	0.005703	-1.600415	1.296015
N	0.465559	1.104248	2.039883
O	2.671107	-0.373311	0.600861

C	-0.119343	-0.716958	3.595993
C	-0.184412	-1.743759	2.658874
C	-0.450233	-3.816873	1.801564
H	-0.619187	-4.876227	1.632821
C	4.847666	-0.536466	-0.376171
C	-0.069710	-3.189204	-0.590719
C	0.193166	0.607076	3.300644
C	-0.154177	-2.865739	0.759230
C	-0.474168	-3.119181	2.981506
H	-0.662278	-3.486273	3.986213
C	3.402873	-0.301381	-0.394959
H	2.964160	-0.042831	-1.375811
C	0.769502	2.441018	2.219095
C	0.311667	1.653505	4.287426
H	0.146839	1.513599	5.351808
C	0.673573	2.792368	3.615788
H	0.866735	3.784700	4.012761
C	5.522757	-0.844220	0.826679
C	6.901182	-1.058854	0.810501
C	5.565448	-0.450763	-1.588927
H	5.031497	-0.215083	-2.511361
C	6.946240	-0.669513	-1.600473
H	7.504268	-0.605228	-2.535001
C	7.611923	-0.972024	-0.401473
H	8.689555	-1.141896	-0.409082
N	0.910187	1.769197	-0.692488
N	0.411261	-0.924313	-1.439641
C	0.837740	0.926671	-3.012062
C	1.016973	1.929656	-2.063538
C	1.476979	3.961801	-1.187101
H	1.727198	5.004270	-1.012921
C	1.122807	3.330425	1.208650
C	0.560266	-0.403820	-2.712996
C	1.184252	3.010878	-0.144362
C	1.364817	3.292507	-2.379783
H	1.508981	3.670201	-3.387927
C	0.176344	-2.276400	-1.610721
C	0.418743	-1.448010	-3.699274
H	0.496615	-1.292181	-4.771355
C	0.187145	-2.612388	-3.013826
H	0.031582	-3.613618	-3.404669
O	-1.604930	0.502895	0.051560
C	-3.423044	-1.056271	0.082504
C	-2.573850	-0.018242	0.746153
H	-2.354906	-0.231137	1.804203
C	-3.298764	-1.273953	-1.301242
C	-4.054714	-2.273084	-1.925855
C	-4.310448	-1.845960	0.835163
H	-4.397613	-1.682469	1.910935
C	-5.068577	-2.843153	0.209364
H	-5.752108	-3.457353	0.797435
C	-4.943501	-3.057384	-1.173212
H	-5.531290	-3.837400	-1.659294
H	-0.234467	-4.228180	-0.870910
H	0.942567	1.198653	-4.061103
H	1.351398	4.355887	1.494456
H	-0.301722	-0.972113	4.638671
H	7.429789	-1.294103	1.734943
H	4.948585	-0.907492	1.750562
H	-2.585236	-0.671911	-1.859464
H	-3.944768	-2.446965	-2.997605
C	-3.431191	2.654331	-0.890321
C	-4.256360	1.908816	0.034792
C	-3.749663	1.437159	1.262248
H	-2.973883	2.003102	1.776475
H	-4.453005	0.936975	1.928840
C	-5.580322	1.390405	-0.441802
H	-6.172763	2.186411	-0.916474
H	-5.412222	0.617200	-1.211463
H	-6.162187	0.940081	0.370254
C	-3.960985	2.944701	-2.278795
H	-4.262053	2.018916	-2.792090
H	-4.849633	3.593982	-2.237091
H	-3.199776	3.447434	-2.889678
C	-2.142128	2.979597	-0.565658
H	-1.721783	2.858913	0.425401
H	-1.475821	3.413051	-1.310438

^{6}TS1A$_{endo}$

Fe	0.331782	0.030884	0.136045
N	0.131060	-1.844390	1.003333
N	0.379801	0.874848	2.026482
O	2.634976	-0.365929	0.562197
C	-0.140239	-1.143316	3.353326
C	-0.095616	-2.109617	2.336597
C	-0.150540	-4.116947	1.282058
H	-0.221046	-5.169645	1.023792
C	4.895823	-0.362556	-0.204763
C	0.269083	-3.214266	-1.046453

C	0.100787	0.232284	3.215844
C	0.104432	-3.051157	0.336419
C	-0.279596	-3.533765	2.519716
H	-0.474834	-4.015906	3.473393
C	3.456419	-0.132201	-0.334362
H	3.109398	0.277659	-1.299574
C	0.671042	2.189213	2.331117
C	0.170248	1.184555	4.303117
H	-0.010257	0.946265	5.347493
C	0.530188	2.393652	3.756705
H	0.698694	3.337977	4.266521
C	5.443576	-0.899678	0.982210
C	6.820261	-1.104259	1.077539
C	5.738289	-0.036250	-1.289478
H	5.303040	0.376617	-2.201264
C	7.117670	-0.241667	-1.188413
H	7.772495	0.010044	-2.023099
C	7.656206	-0.774923	-0.006187
H	8.732255	-0.936488	0.073093
N	0.953040	1.833224	-0.675518
N	0.640310	-0.863558	-1.711273
C	1.007785	1.171170	-3.054778
C	1.099679	2.113789	-2.020133
C	1.459101	4.069888	-0.927138
H	1.666757	5.100675	-0.654063
C	1.044022	3.170212	1.400879
C	0.800248	-0.209074	-2.913669
C	1.170685	3.010544	0.013357
C	1.405682	3.517650	-2.185845
H	1.569230	4.007463	-3.141691
C	0.492216	-2.205466	-1.994363
C	0.758683	-1.170239	-3.995768
H	0.861232	-0.922662	-5.048499
C	0.575425	-2.406673	-3.425729
H	0.494424	-3.370482	-3.919969
O	-1.624502	0.224047	-0.185887
C	-3.644783	-1.058622	-0.046538
C	-2.546824	-0.274933	0.591810
H	-2.202976	-0.687278	1.550858
C	-3.802061	-1.056316	-1.444435
C	-4.834604	-1.796785	-2.033781
C	-4.526655	-1.807411	0.753738
H	-4.398396	-1.809649	1.837613
C	-5.558869	-2.545723	0.163327
H	-6.238156	-3.127756	0.787661
C	-5.716302	-2.539404	-1.232221
H	-6.519588	-3.115937	-1.692924
H	0.191208	-4.231675	-1.428582
H	1.139054	1.545132	-4.069938
H	1.236560	4.167402	1.795791
H	-0.333047	-1.505516	4.362913
H	7.250575	-1.518987	1.989696
H	4.773975	-1.145032	1.805943
H	-3.101050	-0.485075	-2.051165
H	-4.949897	-1.799530	-3.118712
C	-3.425047	2.673996	-0.497155
C	-4.084936	1.881238	0.522049
C	-3.366769	1.218695	1.536216
H	-2.419601	1.619401	1.891299
H	-3.952765	0.706637	2.299651
C	-5.534261	1.549122	0.338875
H	-6.126123	2.455995	0.143456
H	-5.646862	0.900741	-0.546650
H	-5.949142	1.021983	1.204674
C	-4.229055	3.170485	-1.679706
H	-4.717924	2.338903	-2.209128
H	-5.025041	3.859511	-1.357444
H	-3.584992	3.702675	-2.391810
C	-2.076218	2.881680	-0.442897
H	-1.467063	2.603912	0.406731
H	-1.550758	3.372272	-1.261392

^{4}TS2A$_{endo}$

Fe	1.081671	0.864069	-0.226951
N	1.553915	0.017726	-1.983317
N	2.648084	-0.069204	0.624841
C	3.427558	-1.430179	-1.279406
C	2.512540	-0.960638	-2.214347
C	1.441866	-0.705979	-4.187292
H	1.099644	-0.757827	-5.216589
C	-0.094142	1.125798	-3.450862
C	3.493234	-1.001513	0.040995
C	0.906625	0.193588	-3.198460
C	2.442331	-1.418992	-3.577409
H	3.091935	-2.179150	-4.000952
C	3.031383	-0.007737	1.956617
C	4.412114	-1.527883	1.016158
H	5.174359	-2.270568	0.800185
C	4.120740	-0.915569	2.208305

H	4.598248	-1.045540	3.175030
N	0.847597	1.952304	1.444585
N	-0.183639	2.103638	-1.187470
C	-0.927022	3.500530	0.705672
C	-0.081197	2.956957	1.664894
C	0.893413	2.610231	3.671575
H	1.208175	2.631483	4.710607
C	2.461616	0.813559	2.922223
C	-0.963731	3.103662	-0.624966
C	1.448989	1.731907	2.672879
C	-0.055697	3.373450	3.044218
H	-0.687645	4.153052	3.458672
C	-0.580552	2.031376	-2.514358
C	-1.847516	3.667996	-1.612030
H	-2.553098	4.469216	-1.413968
C	-1.610443	3.001290	-2.785927
H	-2.079947	3.140318	-3.755283
O	-0.350428	-0.438437	0.281996
C	-2.733733	-0.607791	0.083975
C	-1.362094	-0.695083	-0.502764
H	-1.292295	-0.302299	-1.526383
C	-2.904196	-0.513937	1.476753
C	-4.191575	-0.419511	2.020207
C	-3.860299	-0.606078	-0.758423
H	-3.723951	-0.675267	-1.839170
C	-5.146676	-0.513016	-0.213888
H	-6.017054	-0.507818	-0.871506
C	-5.314581	-0.421481	1.177302
H	-6.316579	-0.346673	1.601683
H	-0.508326	1.163945	-4.456822
H	-1.606699	4.291165	1.017735
H	2.850983	0.750882	3.936746
H	4.137761	-2.188994	-1.602693
H	-2.020305	-0.501797	2.112734
H	-4.320697	-0.339088	3.100618
C	-0.369167	-3.409023	1.010747
C	-1.347795	-3.299355	-0.052144
C	-1.132731	-2.490627	-1.185531
H	-0.122304	-2.320910	-1.552681
H	-1.892918	-2.532651	-1.966191
C	-2.718000	-3.859364	0.178572
H	-2.663932	-4.924024	0.454637
H	-3.192506	-3.337930	1.026250
H	-3.360963	-3.744203	-0.700333
C	-0.745527	-4.130565	2.286115
H	-1.624674	-3.668705	2.760796
H	-1.003977	-5.181651	2.085754
H	0.084778	-4.112593	3.003936
C	0.836579	-2.776530	0.900923
H	1.174381	-2.300748	-0.009797
H	1.532009	-2.748140	1.739333

^{6}TS2A$_{endo}$

Fe	0.928037	0.497313	0.516084
N	0.181307	2.436175	0.545057
N	2.575282	1.230277	-0.534912
C	1.669768	3.440192	-1.151673
C	0.550501	3.458859	-0.312694
C	-1.295964	4.203744	0.766794
H	-2.139247	4.777894	1.139478
C	-1.631847	2.154786	2.197441
C	2.619893	2.417083	-1.240995
C	-0.941339	2.880290	1.223152
C	-0.375387	4.560420	-0.187747
H	-0.311305	5.487605	-0.749781
C	3.709080	0.521560	-0.885708
C	3.809721	2.454775	-2.062101
H	4.086851	3.282186	-2.708871
C	4.487140	1.282778	-1.838073
H	5.429723	0.954649	-2.266613
N	2.108424	-1.124554	1.082578
N	-0.263120	0.102830	2.188381
C	0.562941	-2.165981	2.701773
C	1.708169	-2.167977	1.897520
C	3.661220	-2.834644	0.962685
H	4.559464	-3.368880	0.666489
C	4.044869	-0.747315	-0.403358
C	-0.338321	-1.107631	2.853555
C	3.305274	-1.510686	0.506784
C	2.667011	-3.245638	1.817948
H	2.591961	-4.181394	2.364422
C	-1.317595	0.869084	2.647133
C	-1.475055	-1.105223	3.746833
H	-1.756484	-1.934321	4.389720
C	-2.076711	0.122648	3.624708
H	-2.953059	0.500237	4.143417
O	-0.083553	-0.275903	-0.914444
C	-2.436167	0.160789	-1.219923
C	-1.035760	0.121113	-1.714780

H	-0.771509	0.935876	-2.407811
C	-2.785236	-0.486393	-0.019859
C	-4.102880	-0.428891	0.447650
C	-3.416905	0.859685	-1.950196
H	-3.141115	1.366785	-2.876459
C	-4.734303	0.911937	-1.482073
H	-5.491217	1.458358	-2.046003
C	-5.079545	0.265689	-0.283103
H	-6.106694	0.308437	0.081321
H	-2.013302	-1.000198	0.545987
H	-4.363885	-0.919260	1.385821
C	-0.454719	-3.147197	-1.779891
C	-1.367154	-2.383590	-2.614134
C	-0.996938	-1.174459	-3.228867
H	0.046330	-0.982033	-3.475245
H	-1.718140	-0.718784	-3.907547
C	-2.812275	-2.773898	-2.630356
H	-2.930297	-3.844483	-2.855109
H	-3.243334	-2.612405	-1.627091
H	-3.389267	-2.182711	-3.349577
C	-0.989525	-4.332385	-1.003992
H	-1.833313	-4.041648	-0.360761
H	-1.359405	-5.116946	-1.682341
H	-0.205412	-4.766654	-0.370788
C	0.845240	-2.760503	-1.619339
H	1.300592	-1.949122	-2.177821
H	1.492554	-3.278341	-0.913336
H	4.972338	-1.184652	-0.771924
H	0.374201	-3.061532	3.292846
H	1.839249	4.323695	-1.766033
H	-2.503274	2.633527	2.642549

TPSSh-D3

^{4}IA

Fe	0.000892	-0.000087	0.053793
N	0.333176	-1.626499	1.166953
N	-0.242473	1.092738	1.706554
O	2.132911	0.528941	0.073293
C	0.127759	-0.642070	3.414590
C	0.344426	-1.702751	2.547713
C	0.719327	-3.806991	1.841636
H	0.906383	-4.869693	1.756057
C	4.454740	0.059656	-0.079041
C	0.622536	-3.313449	-0.603018
C	-0.148023	0.657104	3.014820
C	0.555204	-2.918706	0.725631
C	0.591851	-3.051930	2.972090
H	0.650434	-3.367066	4.005948
C	3.046395	-0.300477	-0.061744
H	2.799746	-1.367242	-0.175442
C	-0.510884	2.446620	1.787963
C	-0.371053	1.747327	3.922472
H	-0.351014	1.659139	5.000921
C	-0.599129	2.857468	3.161385
H	-0.802723	3.870152	3.484991
C	4.855834	1.404139	0.054881
C	6.208393	1.721711	0.032338
C	5.416109	-0.956634	-0.234596
H	5.094253	-1.990302	-0.336954
C	6.770539	-0.631427	-0.256154
H	7.517901	-1.409881	-0.374617
C	7.163238	0.705328	-0.123073
H	8.219404	0.959027	-0.139909
N	-0.322446	1.627921	-1.063495
N	0.238880	-1.095306	-1.601293
C	-0.081040	0.649844	-3.310194
C	-0.302698	1.710240	-2.443972
C	-0.710163	3.808400	-1.736834
H	-0.908327	4.869094	-1.651187
C	-0.667766	3.302717	0.707662
C	0.163402	-0.655501	-2.910108
C	-0.565134	2.916261	-0.621337
C	-0.546347	3.060254	-2.867555
H	-0.581878	3.380436	-3.900904
C	0.472208	-2.456104	-1.683753
C	0.363994	-1.749702	-3.817767
H	0.354105	-1.659398	-4.896200
C	0.554113	-2.867473	-3.057072
H	0.731776	-3.884823	-3.381383
O	-2.133421	-0.513445	0.027808
C	-4.459072	-0.055164	-0.096793
C	-3.053069	0.313524	-0.076314
H	-2.812701	1.384439	-0.161239
C	-4.852011	-1.404086	0.014461
C	-6.202633	-1.729423	-0.014270
C	-5.426436	0.957670	-0.236944
H	-5.110767	1.994793	-0.322220
C	-6.778766	0.624526	-0.266657
H	-7.530701	1.400135	-0.374462

C	-7.163415	-0.716539	-0.155412
H	-8.217982	-0.976365	-0.178100
H	0.803206	-4.362701	-0.810773
H	-0.103371	0.854620	-4.375160
H	-0.870405	4.347851	0.916024
H	0.168687	-0.843927	4.479539
H	6.527768	2.754446	0.134052
H	4.097150	2.171574	0.171954
H	-4.088675	-2.168677	0.119538
H	-6.515927	-2.765515	0.070853

^{6}IA

Fe	0.000173	0.001420	0.055965
N	0.256977	-1.125663	1.748481
N	-0.341695	1.670814	1.200302
O	2.085602	0.504505	0.018956
C	-0.107439	0.641098	3.429055
C	0.162967	-0.676274	3.048577
C	0.597058	-2.890410	3.186624
H	0.791161	-3.902486	3.518491
C	4.410782	0.054584	-0.100836
C	0.659958	-3.320667	0.701735
C	-0.332395	1.727295	2.578795
C	0.513147	-2.479498	1.808413
C	0.379270	-1.776794	3.952353
H	0.361792	-1.701821	5.032072
C	3.010915	-0.322059	-0.078941
H	2.772251	-1.392434	-0.156681
C	-0.558981	2.956089	0.747148
C	-0.567081	3.083324	3.003750
H	-0.611438	3.408151	4.035508
C	-0.704725	3.842528	1.872774
H	-0.886068	4.907498	1.801682
C	4.798181	1.406357	0.004808
C	6.147324	1.735926	-0.025651
C	5.382066	-0.956143	-0.237469
H	5.069937	-1.994560	-0.318320
C	6.732543	-0.617862	-0.269401
H	7.487544	-1.390722	-0.374617
C	7.111520	0.725449	-0.163464
H	8.165055	0.989150	-0.187655
N	-0.255605	1.130732	-1.643512
N	0.332569	-1.668474	-1.097967
C	0.075567	-0.642683	-3.325278
C	-0.173615	0.678609	-2.943937
C	-0.565234	2.900569	-3.082996
H	-0.740918	3.915846	-3.415398
C	-0.631821	3.331236	-0.597870
C	0.301872	-1.729409	-2.475790
C	-0.486824	2.489015	-1.704840
C	-0.372829	1.781799	-3.848186
H	-0.360753	1.705274	-4.927893
C	0.562250	-2.951238	-0.643763
C	0.532987	-3.086238	-2.899898
H	0.561744	-3.414730	-3.931052
C	0.693918	-3.840863	-1.768549
H	0.881277	-4.904779	-1.697045
O	-2.083586	-0.518215	0.076306
C	-4.406240	-0.062995	-0.072187
C	-3.004706	0.308201	-0.056182
H	-2.763271	1.374987	-0.168149
C	-4.799050	-1.410911	0.059662
C	-6.149535	-1.735473	0.036790
C	-5.373602	0.948971	-0.226159
H	-5.057357	1.984405	-0.326883
C	-6.725592	0.615920	-0.248008
H	-7.477623	1.389952	-0.365084
C	-7.109907	-0.723667	-0.116966
H	-8.164565	-0.983395	-0.134123
H	0.853830	-4.368844	0.907479
H	0.096504	-0.845470	-4.391505
H	-0.811783	4.381896	-0.803458
H	-0.140027	0.842628	4.495198
H	6.457342	2.773226	0.055373
H	4.032366	2.168757	0.107120
H	-4.036269	-2.174426	0.175153
H	-6.463571	-2.769908	0.136789

^{4}TS1A$_{endo}$

Fe	0.374624	0.160091	0.327327
N	0.109529	-1.454106	1.482469
N	0.630384	1.288554	1.965690
O	2.625422	-0.281638	0.478011
C	0.173757	-0.391690	3.702061
C	0.031882	-1.487311	2.860530
C	-0.278038	-3.617796	2.201337
H	-0.447333	-4.684915	2.137823
C	4.759227	-0.551348	-0.544852
C	-0.097667	-3.188861	-0.251400
C	0.466061	0.898317	3.277769

C	-0.076621	-2.757648	1.067355
C	-0.216957	-2.828921	3.313598
H	-0.320395	-3.115369	4.352455
C	3.328808	-0.268214	-0.539879
H	2.870267	-0.029966	-1.512849
C	0.944819	2.630446	2.010986
C	0.669977	2.016454	4.160231
H	0.598147	1.967432	5.239160
C	0.974070	3.089864	3.374384
H	1.200898	4.105193	3.673705
C	5.443752	-0.858691	0.647186
C	6.808081	-1.123204	0.606417
C	5.450260	-0.513394	-1.769498
H	4.909167	-0.276211	-2.682736
C	6.817235	-0.781019	-1.804655
H	7.355634	-0.754357	-2.747093
C	7.492896	-1.084456	-0.617399
H	8.558822	-1.292870	-0.643226
N	0.812239	1.737809	-0.833062
N	0.295761	-1.003623	-1.312542
C	0.539052	0.715161	-3.056682
C	0.787145	1.791443	-2.213429
C	1.294399	3.883432	-1.547758
H	1.545474	4.934518	-1.484011
C	1.188290	3.438399	0.908121
C	0.316615	-0.587351	-2.629100
C	1.117855	3.018214	-0.413874
C	1.079268	3.124872	-2.664242
H	1.123283	3.425339	-3.703436
C	0.058616	-2.362337	-1.355053
C	0.097900	-1.704860	-3.508838
H	0.072363	-1.638918	-4.589025
C	-0.054638	-2.806725	-2.718157
H	-0.237639	-3.831910	-3.013382
O	-1.589450	0.557531	0.200598
C	-3.339266	-1.076368	0.185411
C	-2.567203	0.007060	0.868821
H	-2.381747	-0.181233	1.935244
C	-3.202402	-1.255028	-1.198296
C	-3.895492	-2.281262	-1.842752
C	-4.173174	-1.932312	0.917747
H	-4.268871	-1.802140	1.993938
C	-4.867834	-2.956319	0.271356
H	-5.509779	-3.620370	0.843680
C	-4.731207	-3.131595	-1.110260
H	-5.268918	-3.931680	-1.611485
H	-0.277155	-4.242974	-0.433533
H	0.538565	0.902222	-4.125443
H	1.432499	4.479497	1.091788
H	0.079643	-0.561845	4.769507
H	7.345247	-1.359701	1.520078
H	4.890896	-0.883680	1.581282
H	-2.533082	-0.597586	-1.744498
H	-3.775069	-2.424240	-2.913299
C	-3.513114	2.476045	-0.933293
C	-4.317697	1.789799	0.045507
C	-3.792001	1.423811	1.298692
H	-3.037838	2.049021	1.768869
H	-4.483435	0.967969	2.004183
C	-5.637752	1.221523	-0.378996
H	-6.270025	1.997867	-0.827473
H	-5.486609	0.452108	-1.150151
H	-6.174643	0.766268	0.455408
C	-4.008600	2.584995	-2.358564
H	-4.214176	1.596465	-2.786617
H	-4.938406	3.163616	-2.417228
H	-3.264675	3.079844	-2.989101
C	-2.257496	2.898505	-0.607743
H	-1.880137	2.934260	0.405032
H	-1.591193	3.288625	-1.371567

^{6}TS1A$_{endo}$

Fe	0.336602	0.175498	0.325185
N	0.153001	-1.495374	1.516292
N	0.725659	1.318575	1.996814
O	2.542990	-0.274409	0.414985
C	0.335059	-0.399159	3.716401
C	0.135594	-1.513066	2.892825
C	-0.217439	-3.647965	2.237330
H	-0.395395	-4.714749	2.189293
C	4.667553	-0.571147	-0.612545
C	-0.112115	-3.197246	-0.244567
C	0.623573	0.906751	3.304695
C	-0.052627	-2.788220	1.091145
C	-0.105466	-2.859857	3.350273
H	-0.170119	-3.157538	4.389317
C	3.242947	-0.269073	-0.607260
H	2.783466	-0.024815	-1.576915
C	1.052884	2.653736	2.014458

C	0.884101	2.025307	4.180069
H	0.870392	1.980768	5.261741
C	1.154465	3.103274	3.383099
H	1.403379	4.112850	3.685048
C	5.349442	-0.885919	0.579455
C	6.709584	-1.170043	0.536702
C	5.357082	-0.544544	-1.838636
H	4.817880	-0.301121	-2.751246
C	6.719785	-0.832183	-1.875367
H	7.257072	-0.814925	-2.818608
C	7.392734	-1.143192	-0.688475
H	8.455501	-1.366989	-0.715628
N	0.810969	1.779722	-0.882784
N	0.268694	-1.032524	-1.359828
C	0.471993	0.716923	-3.081127
C	0.741627	1.817409	-2.258706
C	1.262558	3.918117	-1.600520
H	1.511287	4.970917	-1.553605
C	1.242803	3.450875	0.880386
C	0.255243	-0.602512	-2.667618
C	1.126405	3.051759	-0.455883
C	1.016707	3.157944	-2.713571
H	1.031429	3.469128	-3.750630
C	0.019661	-2.386015	-1.375696
C	0.000136	-1.725726	-3.538761
H	-0.059458	-1.670440	-4.618477
C	-0.139348	-2.827574	-2.740509
H	-0.341898	-3.848550	-3.038424
O	-1.581173	0.537798	0.277323
C	-3.337833	-1.087720	0.223533
C	-2.563271	-0.027897	0.925676
H	-2.398763	-0.211720	1.994915
C	-3.193162	-1.253494	-1.161912
C	-3.891947	-2.267106	-1.819074
C	-4.185917	-1.941059	0.944135
H	-4.287138	-1.819058	2.020522
C	-4.886545	-2.950830	0.283464
H	-5.539354	-3.613420	0.844731
C	-4.741454	-3.114424	-1.098945
H	-5.283885	-3.904410	-1.610787
H	-0.305209	-4.250640	-0.421946
H	0.444118	0.903737	-4.150436
H	1.496236	4.492263	1.054600
H	0.292143	-0.573490	4.787278
H	7.244994	-1.412453	1.449802
H	4.798022	-0.901172	1.514545
H	-2.513091	-0.600200	-1.699654
H	-3.765048	-2.402371	-2.889579
C	-3.485904	2.517015	-0.858622
C	-4.293164	1.801224	0.106416
C	-3.796166	1.438928	1.366473
H	-3.025168	2.037054	1.843398
H	-4.493385	0.975376	2.060470
C	-5.600217	1.224472	-0.344158
H	-6.233996	1.999557	-0.792484
H	-5.427088	0.468160	-1.123737
H	-6.144601	0.751866	0.475494
C	-3.973420	2.635805	-2.285826
H	-4.157959	1.649341	-2.727735
H	-4.912592	3.199040	-2.344826
H	-3.232503	3.150601	-2.903516
C	-2.252701	2.980374	-0.515916
H	-1.874816	2.983844	0.497901
H	-1.598345	3.417050	-1.264614

^{4}TS2A$_{endo}$

Fe	0.968723	0.759914	0.569159
N	1.681337	1.742583	-1.023160
N	2.621381	-0.369226	0.594129
C	3.701532	0.410049	-1.477882
C	2.801582	1.422248	-1.772201
C	1.846976	3.144246	-2.861855
H	1.591042	3.945376	-3.543066
C	-0.060551	3.463369	-1.287229
C	3.616480	-0.416209	-0.367391
C	1.092604	2.805128	-1.688214
C	2.907804	2.287197	-2.912905
H	3.703068	2.239121	-3.645712
C	2.940762	-1.368268	1.497292
C	4.557554	-1.457175	-0.066087
H	5.427995	-1.687940	-0.666718
C	4.138493	-2.047984	1.091475
H	4.595088	-2.863176	1.637836
N	0.474065	0.035571	2.368916
N	-0.464179	2.147357	0.753598
C	-1.545706	1.367433	2.823204
C	-0.636503	0.366192	3.124596
C	0.329271	-1.339037	4.229046
H	0.601614	-2.121572	4.925300

C	2.217593	-1.679671	2.638352
C	-1.456235	2.198686	1.718715
C	1.069917	-1.015864	3.041126
C	-0.730509	-0.481884	4.279886
H	-1.510674	-0.413271	5.027044
C	-0.777372	3.157486	-0.140396
C	-2.392622	3.244801	1.423173
H	-3.255895	3.481726	2.031351
C	-1.970707	3.841403	0.270762
H	-2.414993	4.671531	-0.262957
O	-0.133573	-0.514186	-0.434189
C	-2.491458	-0.393628	-0.821270
C	-1.086111	-0.155480	-1.254340
H	-0.931130	0.784059	-1.801234
C	-2.749992	-1.212919	0.287686
C	-4.063004	-1.406520	0.719325
C	-3.553879	0.230267	-1.490815
H	-3.349235	0.875178	-2.343061
C	-4.865734	0.033338	-1.058134
H	-5.686294	0.522052	-1.575669
C	-5.122033	-0.786384	0.046773
H	-6.143499	-0.936101	0.384951
H	-0.416521	4.281317	-1.904316
H	-2.379762	1.514619	3.500841
H	2.579623	-2.489439	3.262657
H	4.537706	0.264472	-2.153672
H	-1.913340	-1.668611	0.808574
H	-4.261273	-2.033894	1.584095
C	-0.119377	-3.241141	-1.719772
C	-1.049236	-2.479308	-2.526762
C	-0.789601	-1.155491	-2.909667
H	0.235034	-0.817469	-3.031756
H	-1.495601	-0.690799	-3.593900
C	-2.418324	-3.039822	-2.760460
H	-2.356147	-4.033194	-3.222072
H	-2.943067	-3.169235	-1.802853
H	-3.022988	-2.391431	-3.397165
C	-0.541006	-4.591410	-1.184620
H	-1.445076	-4.516253	-0.568810
H	-0.762597	-5.291220	-1.999567
H	0.252394	-5.029190	-0.573137
C	1.077498	-2.700182	-1.362338
H	1.460186	-1.775439	-1.771736
H	1.729259	-3.210275	-0.658690

^{6}TS2A$_{endo}$

Fe	0.975382	0.784682	0.023086
N	1.447316	0.811563	-1.994455
N	2.714352	-0.284239	0.351572
C	3.308835	-0.804438	-1.982300
C	2.376057	0.006447	-2.628697
C	1.245854	1.048619	-4.282364
H	0.876838	1.415466	-5.231650
C	-0.257758	2.404126	-2.786277
C	3.480406	-0.925638	-0.602934
C	0.755801	1.467822	-2.995476
C	2.247742	0.145951	-4.055817
H	2.859908	-0.371376	-4.783571
C	3.212666	-0.679083	1.578588
C	4.468481	-1.752234	0.039016
H	5.199187	-2.357664	-0.481913
C	4.302199	-1.600593	1.388264
H	4.871631	-2.055933	2.188509
N	0.986933	1.293944	2.023471
N	-0.254346	2.417769	-0.324373
C	-0.829924	2.956682	2.009131
C	0.089562	2.130929	2.655360
C	1.256681	1.135354	4.310397
H	1.663715	0.810332	5.259333
C	2.737066	-0.244395	2.815394
C	-0.984937	3.097217	0.631005
C	1.711761	0.677323	3.022759
C	0.252865	2.034240	4.083085
H	-0.324628	2.592266	4.808957
C	-0.720499	2.852131	-1.549102
C	-1.927537	3.977613	-0.009566
H	-2.618209	4.626775	0.513168
C	-1.763522	3.827046	-1.357726
H	-2.292888	4.328542	-2.157783
O	-0.390286	-0.569330	0.042762
C	-2.766098	-0.646602	-0.246500
C	-1.389882	-0.649224	-0.800693
H	-1.273294	-0.113485	-1.750164
C	-2.971315	-0.750934	1.138073
C	-4.268839	-0.729550	1.652129
C	-3.866348	-0.517976	-1.108893
H	-3.705639	-0.430017	-2.181103
C	-5.160869	-0.499781	-0.591102
H	-6.010212	-0.397665	-1.260251

C	-5.363870	-0.608221	0.789747
H	-6.372688	-0.592254	1.191781
H	-0.715753	2.838377	-3.669506
H	-1.476334	3.559513	2.639211
H	3.224643	-0.645690	3.698339
H	3.982496	-1.376444	-2.612761
H	-2.110200	-0.827770	1.794839
H	-4.426615	-0.802293	2.724385
C	-0.339295	-3.588182	0.241405
C	-1.337965	-3.316968	-0.776369
C	-1.135337	-2.367970	-1.785285
H	-0.130109	-2.117101	-2.110593
H	-1.890756	-2.294681	-2.563916
C	-2.707812	-3.894240	-0.600811
H	-2.662875	-4.967693	-0.384306
H	-3.195611	-3.417540	0.263323
H	-3.336494	-3.730319	-1.477712
C	-0.709552	-4.456020	1.422925
H	-1.606499	-4.083755	1.931306
H	-0.917530	-5.486683	1.110090
H	0.106376	-4.485131	2.150060
C	0.882693	-2.997236	0.174991
H	1.217369	-2.421634	-0.677986
H	1.602900	-3.109766	0.979936

OPBE-D3

^{4}IA

Fe	-0.000096	0.001653	0.014109
N	0.031891	0.225928	1.989385
N	-0.506102	1.918267	-0.195273
O	2.174309	0.612187	-0.086951
C	-0.565425	2.607625	2.166949
C	-0.229856	1.375385	2.707567
C	0.248923	-0.181419	4.259904
H	0.439982	-0.741791	5.171423
C	4.507826	0.149991	-0.096779
C	0.660023	-2.054159	2.675440
C	-0.685588	2.851856	0.807014
C	0.327361	-0.733434	2.936874
C	-0.101204	1.133670	4.116884
H	-0.257535	1.886073	4.885385
C	3.097195	-0.213189	-0.058800
H	2.861712	-1.291818	0.002583
C	-0.736675	2.600825	-1.373799
C	-1.039004	4.127706	0.252279
H	-1.232001	5.021202	0.840518
C	-1.073096	3.970484	-1.107651
H	-1.297651	4.707423	-1.874254
C	4.905919	1.499373	-0.198836
C	6.260969	1.817026	-0.219740
C	5.474528	-0.872490	-0.015835
H	5.153686	-1.913725	0.062502
C	6.831205	-0.547449	-0.033895
H	7.585430	-1.333233	0.029348
C	7.221318	0.795033	-0.135175
H	8.282907	1.049138	-0.149700
N	-0.032089	-0.222725	-1.961700
N	0.505511	-1.915109	0.223332
C	0.571597	-2.602688	-2.139355
C	0.233315	-1.371175	-2.679992
C	-0.251267	0.184092	-4.232073
H	-0.444580	0.743908	-5.143461
C	-0.666621	2.055665	-2.647167
C	0.688849	-2.847650	-0.779299
C	-0.330983	0.735730	-2.908923
C	0.103836	-1.129692	-4.089300
H	0.262717	-1.881424	-4.857930
C	0.732643	-2.598652	1.401929
C	1.041429	-4.123747	-0.224600
H	1.237002	-5.016548	-0.813048
C	1.070345	-3.967964	1.135613
H	1.292544	-4.705571	1.902259
O	-2.174591	-0.610376	0.103589
C	-4.508329	-0.153266	0.065924
C	-3.098255	0.213836	0.067461
H	-2.864102	1.293828	0.029613
C	-4.903746	-1.505606	0.130388
C	-6.257809	-1.827802	0.115965
C	-5.476506	0.867814	-0.014764
H	-5.157443	1.911337	-0.064581
C	-6.832144	0.538361	-0.032256
H	-7.587571	1.323020	-0.095049
C	-7.219661	-0.807083	0.033006
H	-8.280451	-1.064592	0.020224
H	0.870945	-2.704950	3.522510
H	0.750894	-3.429311	-2.825109
H	-0.880220	2.705813	-3.494069
H	-0.741631	3.434947	2.852657
H	6.578652	2.857853	-0.298919

H	4.139237	2.272448	-0.257878
H	-4.135415	-2.277073	0.188695
H	-6.573522	-2.870997	0.166368

^{6}IA

Fe	-0.003474	0.043127	-0.135357
N	0.225306	-1.090362	1.567818
N	-0.463081	1.679511	1.017204
O	2.107174	0.648725	-0.017445
C	-0.241335	0.652816	3.246058
C	0.080552	-0.653696	2.864745
C	0.545775	-2.861378	3.005142
H	0.747021	-3.877871	3.334234
C	4.397339	0.105523	0.292594
C	0.746896	-3.259799	0.524079
C	-0.491216	1.732232	2.392521
C	0.504301	-2.436141	1.629380
C	0.285537	-1.753090	3.772651
H	0.230093	-1.681774	4.855955
C	2.978964	-0.203189	0.222282
H	2.680858	-1.252768	0.392542
C	-0.772422	2.942016	0.561629
C	-0.835926	3.064009	2.818340
H	-0.926130	3.379957	3.854742
C	-1.013973	3.814047	1.681436
H	-1.278931	4.865589	1.603437
C	4.859140	1.426786	0.110257
C	6.223313	1.692555	0.175947
C	5.310318	-0.941063	0.537485
H	4.939789	-1.959100	0.675189
C	6.676747	-0.667537	0.598891
H	7.389775	-1.471548	0.785936
C	7.130004	0.646735	0.417997
H	8.199376	0.860753	0.466327
N	-0.220685	1.170379	-1.823628
N	0.496381	-1.589468	-1.267844
C	0.378010	-0.538167	-3.494245
C	-0.011213	0.750965	-3.117607
C	-0.611936	2.925356	-3.262476
H	-0.867298	3.928911	-3.593536
C	-0.844564	3.315293	-0.784695
C	0.603881	-1.621985	-2.639830
C	-0.579805	2.497888	-1.887550
C	-0.254399	1.841989	-4.025971
H	-0.160120	1.781507	-5.107240
C	0.744434	-2.867175	-0.818202
C	0.946206	-2.953899	-3.067872
H	1.092990	-3.253750	-4.102551
C	1.028300	-3.728336	-1.937425
H	1.259081	-4.788123	-1.862493
O	-2.115887	-0.561774	-0.193524
C	-4.438854	-0.228012	0.181251
C	-3.040571	0.166224	0.202621
H	-2.805729	1.169441	0.598860
C	-4.832724	-1.496114	-0.297295
C	-6.180919	-1.840667	-0.297698
C	-5.403036	0.685921	0.654919
H	-5.083688	1.663594	1.022675
C	-6.752453	0.333951	0.650666
H	-7.503937	1.035248	1.015923
C	-7.138320	-0.927169	0.175100
H	-8.194530	-1.202958	0.172334
H	0.964161	-4.307571	0.730807
H	0.500233	-0.721879	-4.561544
H	-1.107530	4.352035	-0.993815
H	-0.314950	0.847057	4.315948
H	6.590102	2.710836	0.038553
H	4.134046	2.218889	-0.078020
H	-4.070014	-2.186622	-0.658284
H	-6.496383	-2.818532	-0.664539

^{4}TS1A$_{endo}$

Fe	0.363513	0.134472	0.343424
N	0.017648	-1.435111	1.524643
N	0.505109	1.312067	1.950291
O	2.652791	-0.323129	0.638520
C	-0.138949	-0.291944	3.697973
C	-0.190577	-1.418344	2.886618
C	-0.431981	-3.573889	2.283775
H	-0.592308	-4.648011	2.240952
C	4.779989	-0.569888	-0.409671
C	-0.051564	-3.231393	-0.155501
C	0.206858	0.977566	3.251916
C	-0.131598	-2.751247	1.144028
C	-0.477274	-2.741493	3.369094
H	-0.677144	-2.986688	4.409137
C	3.343196	-0.305091	-0.385813
H	2.869907	-0.075296	-1.359298
C	0.860134	2.641709	1.981334
C	0.356549	2.119934	4.113598

H	0.177470	2.109664	5.185682
C	0.776395	3.154697	3.322887
H	1.011268	4.177803	3.605286
C	5.482641	-0.869991	0.775226
C	6.852120	-1.116272	0.720284
C	5.458029	-0.521252	-1.643280
H	4.901232	-0.289401	-2.554098
C	6.830293	-0.769947	-1.693105
H	7.361003	-0.734334	-2.645699
C	7.524403	-1.066423	-0.512117
H	8.597967	-1.261136	-0.550064
N	0.888362	1.664412	-0.826805
N	0.371823	-1.072960	-1.255479
C	0.686848	0.594513	-3.035034
C	0.919987	1.684239	-2.205517
C	1.471277	3.774972	-1.572915
H	1.753352	4.823436	-1.520361
C	1.221255	3.406608	0.879836
C	0.447167	-0.694941	-2.578777
C	1.216795	2.943526	-0.429030
C	1.270604	2.994242	-2.680735
H	1.361597	3.263957	-3.730005
C	0.161759	-2.433884	-1.270954
C	0.286636	-1.836881	-3.439017
H	0.310128	-1.796877	-4.525061
C	0.122668	-2.922630	-2.622637
H	-0.027722	-3.964508	-2.893433
O	-1.576110	0.507647	0.095841
C	-3.388148	-1.038612	0.118505
C	-2.555356	0.008055	0.767895
H	-2.405959	-0.122513	1.854032
C	-3.160588	-1.347764	-1.231337
C	-3.906594	-2.351241	-1.853994
C	-4.365523	-1.739835	0.841708
H	-4.534512	-1.501645	1.894192
C	-5.113124	-2.740721	0.216448
H	-5.871968	-3.286872	0.780104
C	-4.886435	-3.046799	-1.133051
H	-5.469017	-3.832068	-1.618604
H	-0.209578	-4.296673	-0.315541
H	0.730957	0.755711	-4.111159
H	1.488679	4.448679	1.048635
H	-0.340960	-0.421336	4.760381
H	7.404332	-1.348140	1.632513
H	4.935173	-0.903461	1.717830
H	-2.375038	-0.809950	-1.759236
H	-3.716261	-2.599201	-2.900107
C	-3.342887	2.526680	-1.084292
C	-4.213805	1.869799	-0.132702
C	-3.784093	1.546160	1.158995
H	-3.003719	2.131213	1.647314
H	-4.508035	1.089755	1.836145
C	-5.496130	1.287035	-0.628347
H	-6.086013	2.033668	-1.180874
H	-5.273149	0.469357	-1.334473
H	-6.105273	0.875325	0.183969
C	-3.796262	2.654275	-2.516014
H	-4.023291	1.668516	-2.949113
H	-4.713840	3.257247	-2.594290
H	-3.023568	3.130907	-3.133015
C	-2.079007	2.902596	-0.729860
H	-1.701922	2.878278	0.287761
H	-1.383398	3.276581	-1.480457

^{6}TS1A$_{endo}$

Fe	0.362419	0.013037	0.103668
N	0.182424	-1.891584	0.886953
N	0.255540	0.782465	2.007313
O	2.608278	-0.316979	0.637729
C	-0.257069	-1.293160	3.231487
C	-0.109670	-2.215874	2.188386
C	-0.037255	-4.175809	1.066303
H	-0.057751	-5.219948	0.764281
C	4.880010	-0.267369	-0.077443
C	0.485631	-3.177592	-1.189996
C	-0.072631	0.092410	3.150447
C	0.235184	-3.068309	0.182334
C	-0.256554	-3.646080	2.312278
H	-0.489971	-4.169553	3.236159
C	3.444334	-0.051724	-0.233538
H	3.112583	0.374271	-1.197947
C	0.456481	2.092853	2.371494
C	-0.130565	1.004788	4.265270
H	-0.372009	0.722606	5.287117
C	0.205193	2.245347	3.783425
H	0.288710	3.180511	4.331495
C	5.407873	-0.812748	1.110713
C	6.781867	-1.005546	1.228819
C	5.736070	0.078707	-1.141379

H	5.314318	0.498662	-2.057270
C	7.112309	-0.116242	-1.017506
H	7.780772	0.150514	-1.837444
C	7.632255	-0.656964	0.166332
H	8.708827	-0.809927	0.263655
N	0.952388	1.852865	-0.606108
N	0.793716	-0.796766	-1.744839
C	1.186846	1.286106	-2.991660
C	1.179282	2.187594	-1.921509
C	1.393510	4.108355	-0.747469
H	1.544294	5.135693	-0.424867
C	0.847962	3.120072	1.505179
C	1.012828	-0.100292	-2.908228
C	1.079051	3.009138	0.129568
C	1.447695	3.600538	-2.021665
H	1.659113	4.128502	-2.948455
C	0.725139	-2.127386	-2.082267
C	1.088865	-1.018018	-4.018650
H	1.257375	-0.728865	-5.053106
C	0.915674	-2.277587	-3.504745
H	0.911951	-3.226840	-4.034482
O	-1.584037	0.142080	-0.278628
C	-3.649135	-1.034235	-0.127691
C	-2.486073	-0.363171	0.502205
H	-2.138752	-0.808312	1.446981
C	-3.902616	-0.855463	-1.496603
C	-5.018908	-1.457666	-2.082923
C	-4.516374	-1.817339	0.649246
H	-4.315612	-1.948124	1.714733
C	-5.631383	-2.419497	0.060375
H	-6.303001	-3.031387	0.665323
C	-5.885552	-2.238587	-1.305951
H	-6.756519	-2.709534	-1.765411
H	0.472884	-4.183239	-1.610156
H	1.374246	1.700574	-3.982316
H	0.968770	4.110662	1.943394
H	-0.499545	-1.698291	4.214103
H	7.199602	-1.427270	2.144390
H	4.724481	-1.074822	1.919058
H	-3.215727	-0.242974	-2.079337
H	-5.213902	-1.321699	-3.148537
C	-3.471733	2.451814	-0.532737
C	-4.026015	1.759894	0.612542
C	-3.208344	1.163726	1.581204
H	-2.207041	1.537127	1.793585
H	-3.699914	0.672981	2.423060
C	-5.483487	1.441260	0.611079
H	-6.084811	2.340406	0.409789
H	-5.697742	0.727852	-0.202444
H	-5.809047	0.990314	1.554856
C	-4.377812	2.798646	-1.685116
H	-4.909787	1.909319	-2.052182
H	-5.141844	3.531732	-1.383779
H	-3.806409	3.230198	-2.517141
C	-2.128785	2.673564	-0.606478
H	-1.459582	2.506370	0.229690
H	-1.668862	3.074913	-1.509014

^{4}TS2A$_{endo}$

Fe	1.266863	0.637003	-0.258982
N	1.470713	-0.399665	-1.942796
N	2.561458	-0.586217	0.639110
C	2.967611	-2.199179	-1.174218
C	2.162725	-1.584963	-2.121875
C	1.124875	-1.214230	-4.084804
H	0.749848	-1.247886	-5.104341
C	0.116864	0.978460	-3.472277
C	3.157821	-1.716389	0.112072
C	0.854671	-0.153725	-3.156719
C	1.945142	-2.102355	-3.442050
H	2.383353	-3.022420	-3.819869
C	2.955455	-0.555353	1.963238
C	3.935612	-2.393138	1.110001
H	4.500356	-3.304791	0.933510
C	3.801422	-1.674564	2.267189
H	4.239191	-1.865038	3.243664
N	1.305628	1.824483	1.345875
N	0.326218	2.077979	-1.282078
C	-0.018952	3.724329	0.515294
C	0.662799	3.034448	1.506184
C	1.521577	2.564179	3.531577
H	1.835188	2.559150	4.572124
C	2.600605	0.414206	2.888690
C	-0.163820	3.271186	-0.786784
C	1.836614	1.530840	2.584556
C	0.793084	3.504733	2.857223
H	0.376187	4.439002	3.223924
C	-0.098418	2.032467	-2.595707
C	-0.884919	3.985319	-1.802284

H	-1.359073	4.951885	-1.653911
C	-0.847749	3.210949	-2.929664
H	-1.282137	3.405705	-3.906804
O	-0.427507	-0.228031	0.333166
C	-2.796291	-0.073405	0.170339
C	-1.462588	-0.301182	-0.441090
H	-1.347218	0.077446	-1.468053
C	-2.937332	-0.113853	1.566143
C	-4.196482	0.065419	2.144535
C	-3.918763	0.147443	-0.641486
H	-3.803146	0.170910	-1.727227
C	-5.176675	0.330752	-0.060466
H	-6.048721	0.507672	-0.692638
C	-5.317733	0.287312	1.333059
H	-6.300488	0.429320	1.786250
H	-0.296039	1.055294	-4.476919
H	-0.475145	4.677245	0.778062
H	2.970003	0.308288	3.907402
H	3.474541	-3.121262	-1.453360
H	-2.050770	-0.288800	2.174421
H	-4.306039	0.036155	3.230338
C	-1.201113	-3.054846	1.104570
C	-1.980143	-2.851502	-0.099343
C	-1.475324	-2.142182	-1.197060
H	-0.407978	-2.111469	-1.411557
H	-2.118182	-2.052607	-2.074142
C	-3.437453	-3.170395	-0.059801
H	-3.608017	-4.192324	0.310748
H	-3.939853	-2.490363	0.648482
H	-3.914371	-3.057095	-1.039772
C	-1.886547	-3.583950	2.336638
H	-2.749452	-2.960175	2.611009
H	-2.263739	-4.605624	2.174722
H	-1.194465	-3.612466	3.188173
C	0.104362	-2.663444	1.147889
H	0.657556	-2.351262	0.270201
H	0.666484	-2.688701	2.081581

^{6}TS2A$_{endo}$

Fe	1.223655	0.399533	-0.236596
N	1.352481	-0.755436	-1.952230
N	2.512265	-0.935767	0.679768
C	2.574387	-2.693668	-1.042135
C	1.857543	-2.037176	-2.043775
C	0.845612	-1.638878	-4.023803
H	0.451902	-1.690220	-5.035765
C	0.181664	0.735098	-3.525688
C	2.891338	-2.176798	0.215459
C	0.753916	-0.486793	-3.166331
C	1.535940	-2.599346	-3.329033
H	1.817519	-3.597607	-3.654604
C	2.988701	-0.842124	1.969805
C	3.609662	-2.887703	1.240521
H	4.018324	-3.888856	1.129528
C	3.666076	-2.060270	2.332593
H	4.136338	-2.243513	3.295548
N	1.721302	1.777663	1.214138
N	0.615946	1.973325	-1.444801
C	0.684430	3.780182	0.229008
C	1.310534	3.089856	1.268390
C	2.230590	2.662168	3.284936
H	2.613752	2.707211	4.301114
C	2.863873	0.277697	2.794077
C	0.373317	3.265685	-1.030170
C	2.289317	1.498816	2.436858
C	1.623691	3.652044	2.557458
H	1.407879	4.675308	2.854048
C	0.146665	1.887154	-2.736877
C	-0.264318	4.009086	-2.086153
H	-0.558220	5.053316	-2.017051
C	-0.407662	3.151795	-3.145911
H	-0.842174	3.350543	-4.122426
O	-0.551989	-0.062241	0.416328
C	-2.893648	0.349093	0.324428
C	-1.613325	0.003637	-0.331634
H	-1.479603	0.383683	-1.355646
C	-3.011401	0.262632	1.721330
C	-4.235644	0.539684	2.334133
C	-4.004303	0.714348	-0.452570
H	-3.907990	0.772505	-1.538707
C	-5.226301	0.994376	0.164338
H	-6.088238	1.281569	-0.440368
C	-5.344467	0.904542	1.557715
H	-6.299632	1.121759	2.039082
H	-0.241612	0.807875	-4.527374
H	0.418986	4.820241	0.416444
H	3.285591	0.203102	3.796254
H	2.918914	-3.703314	-1.263782
H	-2.139337	-0.028907	2.305155

H	-4.327116	0.474454	3.419924
C	-1.663132	-2.815236	1.158930
C	-2.412352	-2.495880	-0.042772
C	-1.828079	-1.838065	-1.131360
H	-0.761671	-1.911539	-1.338959
H	-2.453439	-1.657741	-2.007294
C	-3.895998	-2.641516	-0.008368
H	-4.189059	-3.632085	0.370073
H	-4.314042	-1.899276	0.693257
H	-4.351118	-2.478093	-0.991396
C	-2.413108	-3.261280	2.386240
H	-3.204193	-2.546156	2.651372
H	-2.898888	-4.235148	2.219119
H	-1.735666	-3.365110	3.243531
C	-0.314707	-2.626569	1.197107
H	0.271356	-2.385933	0.317858
H	0.245499	-2.756048	2.123338

wB97XD

^{4}IA

Fe	0.001057	0.004417	-0.009395
N	-0.321009	1.759539	0.883420
N	-0.191356	0.878789	-1.788299
O	2.150912	0.494720	-0.044985
C	-0.606616	3.190124	-1.085579
C	-0.538076	2.974209	0.277960
C	-0.565909	3.402794	2.478820
H	-0.620036	3.852825	3.461217
C	4.472845	0.039729	0.052605
C	-0.138598	1.070255	3.228685
C	-0.439700	2.205863	-2.040398
C	-0.327970	2.010663	2.234081
C	-0.697259	3.999941	1.266760
H	-0.879894	5.043267	1.045259
C	3.061973	-0.312348	0.127470
H	2.824570	-1.365782	0.347117
C	-0.080166	0.281568	-3.019370
C	-0.494355	2.441486	-3.454097
H	-0.676184	3.406025	-3.909324
C	-0.268334	1.249577	-4.060779
H	-0.227809	1.027807	-5.119053
C	4.870705	1.359690	-0.203652
C	6.220400	1.663821	-0.278347
C	5.429890	-0.965979	0.232206
H	5.111366	-1.986576	0.429461
C	6.781383	-0.655949	0.153499
H	7.528563	-1.431128	0.288194
C	7.172432	0.657249	-0.100894
H	8.228993	0.900129	-0.163172
N	0.326573	-1.750593	-0.900077
N	0.196407	-0.872214	1.771042
C	0.577400	-3.189377	1.067652
C	0.526305	-2.968522	-0.295327
C	0.590227	-3.390943	-2.496322
H	0.659935	-3.837480	-3.479316
C	0.176247	-1.056693	-3.246039
C	0.420339	-2.203467	2.024007
C	0.356134	-1.997725	-2.250806
C	0.693424	-3.993067	-1.284784
H	0.865286	-5.038219	-1.063257
C	0.100773	-0.271234	3.002649
C	0.476034	-2.438536	3.437215
H	0.641140	-3.405842	3.892970
C	0.277590	-1.241051	4.043681
H	0.247037	-1.017801	5.102006
O	-2.153166	-0.470205	0.017290
C	-4.482290	-0.044162	0.081029
C	-3.076855	0.330217	0.147352
H	-2.854587	1.394933	0.323473
C	-4.862558	-1.375333	-0.141719
C	-6.208306	-1.699624	-0.203227
C	-5.452381	0.952353	0.241019
H	-5.147266	1.981582	0.413020
C	-6.799795	0.621590	0.178007
H	-7.557009	1.389262	0.299022
C	-7.173424	-0.702519	-0.043523
H	-8.226800	-0.961436	-0.094131
H	-0.180548	1.409497	4.258071
H	0.744699	-4.204808	1.410709
H	0.232913	-1.393301	-4.275533
H	-0.790604	4.202430	-1.429295
H	6.537820	2.682808	-0.474862
H	4.115222	2.126743	-0.340069
H	-4.097111	-2.135079	-0.263094
H	-6.512486	-2.727054	-0.375210

^{6}IA

Fe	-0.000095	0.004416	0.000031
N	0.334134	1.719813	-1.066564
N	-0.216391	-1.047629	-1.740194

O	2.092950	-0.479790	-0.023571
C	0.148634	0.786626	-3.329115
C	0.344667	1.832360	-2.432328
C	0.687494	3.911462	-1.637584
H	0.858272	4.973241	-1.516347
C	4.418623	-0.053282	0.041940
C	0.590146	3.293350	0.798904
C	-0.110846	-0.542909	-3.008244
C	0.534014	2.975722	-0.555211
C	0.573192	3.206227	-2.794784
H	0.630985	3.575633	-3.810400
C	3.018525	0.322448	0.117809
H	2.796152	1.383359	0.308158
C	-0.459572	-2.390795	-1.857317
C	-0.304580	-1.603655	-3.961050
H	-0.272814	-1.480634	-5.035669
C	-0.521716	-2.743403	-3.251718
H	-0.701484	-3.741331	-3.629991
C	4.796789	-1.384011	-0.192170
C	6.141739	-1.706971	-0.266471
C	5.390020	0.943433	0.200401
H	5.086223	1.971565	0.380151
C	6.736348	0.613217	0.123376
H	7.494645	1.380096	0.241763
C	7.107572	-0.709846	-0.109203
H	8.160484	-0.968183	-0.170726
N	-0.325240	-1.711197	1.073127
N	0.219183	1.059892	1.743415
C	-0.117327	-0.780448	3.333974
C	-0.316114	-1.827561	2.438343
C	-0.677635	-3.902684	1.641511
H	-0.853607	-4.963584	1.519924
C	-0.612484	-3.276411	-0.795070
C	0.124579	0.551754	3.012483
C	-0.535670	-2.964706	0.560614
C	-0.542197	-3.201203	2.799806
H	-0.585496	-3.574230	3.814837
C	0.440048	2.406444	1.862521
C	0.304318	1.614286	3.965204
H	0.278314	1.489849	5.039860
C	0.498588	2.758955	3.256345
H	0.662272	3.759338	3.635440
O	-2.098946	0.458626	0.013041
C	-4.426017	0.034982	0.053584
C	-3.027146	-0.344426	0.130492
H	-2.805851	-1.408719	0.302578
C	-4.801799	1.367403	-0.174716
C	-6.146208	1.693244	-0.246288
C	-5.399242	-0.960339	0.209988
H	-5.097156	-1.989581	0.386346
C	-6.745026	-0.626818	0.138143
H	-7.504705	-1.392278	0.256850
C	-7.113835	0.697733	-0.089703
H	-8.166324	0.958538	-0.147977
H	0.759363	4.335897	1.049399
H	-0.153834	-1.026961	4.390457
H	-0.794356	-4.316714	-1.046237
H	0.196698	1.032271	-4.385276
H	6.445148	-2.733119	-0.446619
H	4.030979	-2.143537	-0.312050
H	-4.034722	2.125930	-0.292919
H	-6.447834	2.720442	-0.423396

[4]TS1A$_{endo}$

Fe	0.386487	0.389339	0.044235
N	0.496895	-1.436951	0.873742
N	0.601624	1.215379	1.853137
O	2.678025	0.389063	-0.033429
C	0.742776	-0.806862	3.228400
C	0.626037	-1.732676	2.207000
C	0.407079	-3.717320	1.184806
H	0.317061	-4.765582	0.932236
C	4.788862	-0.644204	-0.364431
C	0.215027	-2.817650	-1.129321
C	0.725461	0.565293	3.053428
C	0.359967	-2.643187	0.234385
C	0.578963	-3.153300	2.406909
H	0.664380	-3.640801	3.369092
C	3.334847	-0.549660	-0.468774
H	2.823753	-1.390491	-0.967476
C	0.623202	2.553898	2.146118
C	0.822899	1.516963	4.125054
H	0.932556	1.257153	5.169834
C	0.754225	2.749598	3.563072
H	0.797692	3.715741	4.048290
C	5.531683	0.356152	0.277080
C	6.908627	0.228556	0.375204
C	5.430241	-1.764030	-0.904557
H	4.844726	-2.535885	-1.398067

C	6.810503	-1.886797	-0.805291
H	7.312289	-2.753551	-1.222805
C	7.545082	-0.890895	-0.165368
H	8.623537	-0.987617	-0.083115
N	0.442488	2.195771	-0.794989
N	0.336518	-0.455076	-1.771892
C	0.319423	1.562800	-3.158506
C	0.389821	2.487473	-2.132942
C	0.432490	4.477767	-1.097835
H	0.449057	5.528490	-0.839553
C	0.547105	3.581114	1.223093
C	0.280628	0.192516	-2.978739
C	0.477454	3.402824	-0.146889
C	0.384431	3.910179	-2.329735
H	0.350677	4.396149	-3.295912
C	0.210995	-1.791164	-2.056145
C	0.133834	-0.757915	-4.045447
H	0.071288	-0.499703	-5.094498
C	0.084620	-1.986945	-3.472938
H	-0.026229	-2.950979	-3.951679
O	-1.634140	0.395355	0.140196
C	-3.077590	-1.466186	0.489543
C	-2.347292	-0.259598	0.986159
H	-1.940130	-0.378880	2.000949
C	-3.212161	-1.672485	-0.884418
C	-3.856590	-2.811016	-1.356924
C	-3.585440	-2.406893	1.385372
H	-3.465512	-2.252876	2.455564
C	-4.229338	-3.545512	0.911982
H	-4.618203	-4.278351	1.612558
C	-4.367666	-3.747483	-0.459811
H	-4.867829	-4.637577	-0.829735
H	7.492034	0.997062	0.872186
H	5.018822	1.217600	0.692745
H	-2.790860	-0.942082	-1.568233
H	-3.955682	-2.972193	-2.426417
C	-4.069757	2.000729	-0.532762
C	-4.490462	1.197035	0.597321
C	-3.669270	0.982532	1.700649
H	-2.929495	1.723728	1.986208
H	-4.098567	0.440632	2.539600
C	-5.750552	0.401885	0.472571
H	-6.588422	1.045361	0.182002
H	-5.642056	-0.354938	-0.316865
H	-6.005748	-0.110404	1.401511
C	-4.905570	1.996738	-1.789749
H	-5.057529	0.981942	-2.173943
H	-5.896679	2.430720	-1.614017
H	-4.422665	2.583597	-2.575202
C	-2.891286	2.661702	-0.492103
H	-2.293806	2.759877	0.403586
H	-2.509221	3.174198	-1.370871
H	0.102308	-3.830874	-1.499788
H	0.571224	4.598643	1.598622
H	0.842730	-1.184583	4.240555
H	0.272229	1.940598	-4.174364

^{6}TS1A$_{endo}$

Fe	0.375784	0.303119	0.042576
N	0.441486	-1.573911	0.922683
N	0.590888	1.158816	1.901758
O	2.622274	0.238884	0.069720
C	0.560299	-0.874498	3.271746
C	0.464324	-1.834306	2.265188
C	0.215563	-3.830180	1.251447
H	0.091897	-4.879328	1.016416
C	4.760211	-0.729428	-0.251587
C	0.208960	-2.936667	-1.103049
C	0.625730	0.507345	3.103328
C	0.286784	-2.770672	0.278415
C	0.330720	-3.253174	2.477206
H	0.321325	-3.736004	3.445715
C	3.305052	-0.692600	-0.345285
H	2.816344	-1.560554	-0.815999
C	0.696613	2.497536	2.158490
C	0.741633	1.475087	4.165325
H	0.793374	1.234981	5.219430
C	0.783151	2.702745	3.582543
H	0.877190	3.667441	4.063953
C	5.468553	0.318997	0.351730
C	6.850375	0.250354	0.436192
C	5.440484	-1.837600	-0.768357
H	4.882010	-2.646117	-1.233335
C	6.825656	-1.900437	-0.683214
H	7.357939	-2.757426	-1.082635
C	7.525885	-0.857433	-0.080643
H	8.608340	-0.907348	-0.010009
N	0.613367	2.133580	-0.835988
N	0.442250	-0.598843	-1.810083

C	0.554864	1.429025	-3.184897
C	0.637730	2.388690	-2.178982
C	0.795326	4.392962	-1.161244
H	0.885398	5.445418	-0.925331
C	0.742488	3.503386	1.194478
C	0.451285	0.050320	-3.013342
C	0.717375	3.333738	-0.187863
C	0.747060	3.810214	-2.389227
H	0.791453	4.290816	-3.357891
C	0.281746	-1.933435	-2.066875
C	0.308039	-0.914105	-4.075107
H	0.289097	-0.674858	-5.130512
C	0.199597	-2.137014	-3.491163
H	0.074319	-3.098303	-3.972372
O	-1.591355	0.316265	0.061051
C	-3.270340	-1.329889	0.412106
C	-2.371044	-0.254582	0.908383
H	-1.989683	-0.408721	1.925892
C	-3.412556	-1.532782	-0.962263
C	-4.240970	-2.547544	-1.429799
C	-3.948208	-2.152584	1.312623
H	-3.824020	-2.000008	2.382325
C	-4.776452	-3.165437	0.842700
H	-5.302557	-3.805248	1.544496
C	-4.926191	-3.361164	-0.529165
H	-5.573432	-4.152314	-0.895408
H	7.406911	1.056218	0.903737
H	4.925375	1.171045	0.747742
H	-2.858684	-0.899654	-1.648424
H	-4.349264	-2.708497	-2.498182
C	-3.862306	2.193660	-0.603393
C	-4.335574	1.469278	0.567803
C	-3.525475	1.211322	1.661418
H	-2.668663	1.837657	1.887136
H	-3.980908	0.738728	2.527738
C	-5.677892	0.813502	0.496384
H	-6.450049	1.541665	0.224342
H	-5.679990	0.041574	-0.285021
H	-5.955221	0.344025	1.441308
C	-4.712929	2.197309	-1.850383
H	-4.929336	1.179581	-2.193737
H	-5.674699	2.695029	-1.681945
H	-4.204369	2.724115	-2.661715
C	-2.648017	2.780361	-0.599275
H	-2.025746	2.854499	0.282902
H	-2.250127	3.247390	-1.496027
H	0.077427	-3.951522	-1.465517
H	0.830554	4.522304	1.559046
H	0.583109	-1.242299	4.293122
H	0.565728	1.794170	-4.207315

^{4}TS2A$_{endo}$

Fe	1.080676	0.799012	0.269785
N	1.948074	0.847093	-1.525851
N	2.453083	-0.517086	0.874501
C	3.645026	-0.897269	-1.232371
C	2.951966	0.026664	-1.988191
C	2.334448	1.244265	-3.766146
H	2.225201	1.702582	-4.740149
C	0.620478	2.609377	-2.593563
C	3.410834	-1.137361	0.107027
C	1.570738	1.607039	-2.608864
C	3.193840	0.267798	-3.380157
H	3.939813	-0.248024	-3.970316
C	2.548170	-1.098786	2.116704
C	4.113843	-2.118746	0.878863
H	4.919051	-2.734883	0.500927
C	3.573605	-2.099789	2.124057
H	3.844549	-2.693316	2.987370
N	0.512511	1.041193	2.170932
N	0.040736	2.435895	-0.219978
C	-1.071875	2.891000	1.915338
C	-0.438121	1.909130	2.649410
C	0.098167	0.620234	4.401857
H	0.191202	0.145986	5.369834
C	1.787755	-0.765981	3.220051
C	-0.834975	3.134394	0.578325
C	0.849497	0.245237	3.238690
C	-0.697470	1.655598	4.037189
H	-1.400772	2.214088	4.640507
C	-0.072924	3.004709	-1.467308
C	-1.496250	4.157404	-0.176960
H	-2.226299	4.844403	0.229943
C	-1.025542	4.075779	-1.446121
H	-1.285397	4.681953	-2.303987
O	-0.345308	-0.496145	-0.150929
C	-2.691743	-0.431909	-0.550552
C	-1.280052	-0.366311	-1.027802
H	-1.115005	0.356504	-1.837953

C	-2.965940	-0.872735	0.745282
C	-4.280690	-0.924359	1.196925
C	-3.736772	-0.035869	-1.386223
H	-3.521994	0.317648	-2.392324
C	-5.050575	-0.090100	-0.933587
H	-5.861281	0.220632	-1.585435
C	-5.323717	-0.537254	0.357779
H	-6.349267	-0.578068	0.712026
H	0.428297	3.138618	-3.520737
H	-1.802170	3.512400	2.422080
H	1.963013	-1.311816	4.140882
H	4.427456	-1.467861	-1.721232
H	-2.140993	-1.156392	1.391366
H	-4.492053	-1.260883	2.207480
C	-0.562667	-3.501986	-0.572677
C	-1.410940	-2.926464	-1.603549
C	-1.030653	-1.816153	-2.346726
H	0.018106	-1.585792	-2.505543
H	-1.683606	-1.517037	-3.162687
C	-2.813457	-3.427093	-1.726387
H	-2.821171	-4.510816	-1.890652
H	-3.364907	-3.242955	-0.794673
H	-3.348971	-2.943775	-2.544632
C	-1.141518	-4.535679	0.362252
H	-2.016872	-4.150367	0.896820
H	-1.460037	-5.434887	-0.176985
H	-0.399396	-4.840491	1.103982
C	0.698804	-3.054257	-0.404737
H	1.177533	-2.361956	-1.084154
H	1.312278	-3.413464	0.417091

^{6}TS2A$_{endo}$

Fe	-1.187448	-0.461289	-0.090139
N	-1.801894	0.630482	-1.731847
N	-2.361718	0.769413	1.073103
C	-2.991539	2.450571	-0.596618
C	-2.450009	1.842192	-1.721162
C	-1.849013	1.500276	-3.860837
H	-1.687422	1.566378	-4.928834
C	-0.833342	-0.763074	-3.502442
C	-2.953426	1.949906	0.697315
C	-1.444601	0.394106	-3.037278
C	-2.478326	2.390435	-3.049200
H	-2.935794	3.333969	-3.316422
C	-2.538098	0.662630	2.430271
C	-3.500327	2.610058	1.850947
H	-4.024841	3.556371	1.828638
C	-3.232671	1.820179	2.923980
H	-3.497045	1.984678	3.960393
N	-1.295257	-1.861409	1.420528
N	-0.744893	-2.005078	-1.391806
C	-0.360614	-3.795034	0.239343
C	-0.804217	-3.141021	1.380357
C	-1.296479	-2.755938	3.539300
H	-1.454098	-2.819980	4.607913
C	-2.159093	-0.425047	3.205935
C	-0.336785	-3.268920	-1.044714
C	-1.602315	-1.604895	2.732143
C	-0.808677	-3.707373	2.702479
H	-0.482294	-4.710314	2.944158
C	-0.537526	-1.888548	-2.743961
C	0.135334	-3.965488	-2.210345
H	0.505057	-4.982504	-2.212758
C	0.017359	-3.110345	-3.259565
H	0.265426	-3.285575	-4.298263
O	0.603805	0.193915	0.196259
C	2.939266	-0.191061	0.009485
C	1.620686	0.115630	-0.595079
H	1.454748	-0.304896	-1.593098
C	3.118455	-0.073667	1.389808
C	4.360580	-0.347100	1.951221
C	4.002021	-0.591441	-0.801890
H	3.856500	-0.689547	-1.875049
C	5.242636	-0.866522	-0.236879
H	6.067368	-1.181479	-0.868485
C	5.423635	-0.740344	1.138967
H	6.391998	-0.953883	1.580776
H	-0.614998	-0.812841	-4.564606
H	0.002226	-4.810416	0.362718
H	-2.358961	-0.364909	4.271074
H	-3.487990	3.404665	-0.742560
H	2.279137	0.225511	2.009446
H	4.500565	-0.258440	3.024133
C	1.633328	3.081485	0.711151
C	2.346759	2.650278	-0.487246
C	1.738524	1.931230	-1.501715
H	0.665315	1.968328	-1.662279
H	2.323038	1.723521	-2.394330
C	3.828750	2.838511	-0.526687

H	4.082487	3.891451	-0.357734
H	4.307851	2.264682	0.277608
H	4.256068	2.522395	-1.479257
C	2.429252	3.579555	1.892354
H	3.166617	2.840776	2.224185
H	2.974709	4.498739	1.649958
H	1.768392	3.799019	2.734481
C	0.291803	2.981078	0.777727
H	-0.317942	2.694420	-0.069003
H	-0.244554	3.234423	1.687905

B3LYP*-D3

[4]IA

Fe -0.00000100 0.00000000 -0.00005200
N -0.00921300 0.06099600 2.01292700
N -0.36933000 1.98252500 -0.06095900
O 2.20989200 0.50299000 -0.00137400
C -0.43895000 2.47817300 2.36825000
C -0.20564100 1.17998600 2.82374100
C 0.10594500 -0.54850700 4.25874800
H 0.22165500 -1.19102600 5.13090200
C 4.55916200 0.03443500 0.00121200
C 0.41784700 -2.33034500 2.51564200
C -0.50861200 2.84999800 1.02491900
C 0.18305100 -1.00694000 2.89070100
C -0.13460600 0.80508600 4.21723800
H -0.25409200 1.49925100 5.04833100
C 3.14334700 -0.32874700 0.00353700
H 2.90493100 -1.41293400 0.00940900
C -0.49799400 2.78301700 -1.19821100
C -0.73935300 4.19776300 0.55878200
H -0.88153400 5.05882900 1.21099000
C -0.73290200 4.15635000 -0.81670200
H -0.86816300 4.97663000 -1.52086400
C 4.96570100 1.39436500 -0.00293400
C 6.32759300 1.70955300 -0.00668100
C 5.53079200 -0.99792500 0.00157600
H 5.20759500 -2.04366000 0.00459000
C 6.89426900 -0.67453400 -0.00267900
H 7.64697600 -1.46716400 -0.00295200
C 7.29060300 0.67690600 -0.00674100
H 8.35480700 0.92924200 -0.00999100
N 0.00921300 -0.06099300 -2.01302900
N 0.36933100 -1.98252300 0.06085600
C 0.43894500 -2.47817200 -2.36835300
C 0.20563400 -1.17998500 -2.82384300
C -0.10596500 0.54850500 -4.25885000
H -0.22168700 1.19102200 -5.13100400
C -0.41784600 2.33034800 -2.51574500
C 0.50861000 -2.84999700 -1.02502100
C -0.18305200 1.00694200 -2.89080300
C 0.13460700 -0.80508300 -4.21734000
H 0.25409800 -1.49924800 -5.04843300
C 0.49799500 -2.78301400 1.19810800
C 0.73935100 -4.19776100 -0.55888500
H 0.88152900 -5.05882900 -1.21109100
C 0.73290100 -4.15634700 0.81659900
H 0.86816200 -4.97662800 1.52076200
O -2.20988600 -0.50298400 0.00129800
C -4.55915800 -0.03443600 -0.00110000
C -3.14334500 0.32875000 -0.00356900
H -2.90493200 1.41293800 -0.00935600
C -4.96569300 -1.39436700 0.00303800
C -6.32758400 -1.70955900 0.00691300
C -5.53079000 0.99792100 -0.00131500
H -5.20759600 2.04365800 -0.00431900
C -6.89426700 0.67452700 0.00306700
H -7.64697500 1.46715500 0.00345000
C -7.29059700 -0.67691400 0.00711500
H -8.35479900 -0.92925400 0.01046600
H 0.54417500 -3.06654100 3.31228600
H 0.57075300 -3.26098000 -3.11826800
H -0.54417600 3.06654300 -3.31238900
H -0.57075900 3.26098200 3.11816400
H 6.64838400 2.75458800 -0.00976300
H 4.20168700 2.17540500 -0.00307600
H -4.20167700 -2.17540500 0.00306600
H -6.64837200 -2.75459500 0.00998500

[6]IA

Fe 0.00001200 0.00000600 0.00005500
N 0.00199800 0.00538100 2.07076700
N -0.35900000 2.04156200 -0.00491700
O 2.17300300 0.46100700 -0.00243600
C -0.39610100 2.42741900 2.44819300
C -0.17928300 1.11167900 2.89563300
C 0.11746600 -0.66938100 4.28436600
H 0.22696700 -1.32876800 5.14494500
C 4.52232300 0.00851600 -0.00202000
C 0.40116600 -2.41460100 2.46001300

C -0.46964400 2.86509800 1.11328300
C 0.18551600 -1.09649300 2.90104500
C -0.10736300 0.69201100 4.28101400
H -0.21438900 1.35608000 5.13830400
C 3.11275600 -0.36728400 -0.00108700
H 2.87885400 -1.45121400 0.00087400
C -0.47181600 2.85926000 -1.12717000
C -0.67135100 4.23306400 0.67986500
H -0.78915400 5.08586800 1.34797400
C -0.67273500 4.22943700 -0.70051700
H -0.79179400 5.07872100 -1.37287500
C 4.91727100 1.37251600 -0.00407500
C 6.27619000 1.69871000 -0.00502100
C 5.50281800 -1.01630800 -0.00092200
H 5.18843400 -2.06458100 0.00068100
C 6.86309200 -0.68122500 -0.00191100
H 7.62265100 -1.46716900 -0.00111100
C 7.24763600 0.67378100 -0.00396400
H 8.30962400 0.93510700 -0.00473600
N -0.00200400 -0.00537700 -2.07067500
N 0.35899900 -2.04155800 0.00501100
C 0.39603400 -2.42742400 -2.44809800
C 0.17923900 -1.11167900 -2.89553900
C -0.11744900 0.66938900 -4.28427300
H -0.22692700 1.32878000 -5.14485200
C -0.40111200 2.41461400 -2.45991900
C 0.46960200 -2.86509900 -1.11318900
C -0.18548500 1.09650100 -2.90095100
C 0.10733000 -0.69201200 -4.28092100
H 0.21433100 -1.35608500 -5.13821000
C 0.47184900 -2.85925000 1.12726300
C 0.67131800 -4.23306500 -0.67977100
H 0.78909500 -5.08587200 -1.34787900
C 0.67275300 -4.22942900 0.70061100
H 0.79183400 -5.07871000 1.37296900
O -2.17299900 -0.46101500 0.00248700
C -4.52232000 -0.00852400 0.00189800
C -3.11275100 0.36727500 0.00106300
H -2.87885100 1.45120600 -0.00085200
C -4.91726800 -1.37252400 0.00392700
C -6.27618700 -1.69871900 0.00477500
C -5.50281400 1.01629900 0.00072700
H -5.18843000 2.06457200 -0.00085600
C -6.86308800 0.68121700 0.00161700
H -7.62264800 1.46716000 0.00076000
C -7.24763300 -0.67379000 0.00364500
H -8.30962100 -0.93511500 0.00434100
H 0.51798000 -3.17559900 3.23567600
H 0.51100300 -3.19248800 -3.22002500
H -0.51790600 3.17561500 -3.23558100
H -0.51109300 3.19247900 3.22012000
H 6.58845200 2.74624800 -0.00658900
H 4.14704000 2.14731200 -0.00483100
H -4.14703700 -2.14732000 0.00474200
H -6.58844900 -2.74625600 0.00632400

^{4}TS1A$_{endo}$

Fe 0.34965000 0.07453100 0.22470100
N 0.02997500 -1.88695900 0.59505400
N 0.48835700 0.41930500 2.21867500
O 2.64013200 -0.47803900 0.36347200
C -0.01415100 -1.85025100 3.07409700
C -0.11601000 -2.49614100 1.83985800
C -0.41484200 -4.15593400 0.32212200
H -0.59335700 -5.09995700 -0.19144100
C 4.89769900 -0.40389900 -0.44455100
C -0.07968600 -2.74047200 -1.72928500
C 0.27673000 -0.49381600 3.24722500
C -0.14530300 -2.90093800 -0.34415500
C -0.39819300 -3.90554400 1.67368600
H -0.55758100 -4.60335400 2.49507300
C 3.46406300 -0.09763700 -0.49086900
H 3.11696400 0.51386500 -1.35126400
C 0.76101400 1.63441000 2.84016700
C 0.41019200 0.16227700 4.53127100
H 0.29233700 -0.33546600 5.49330400
C 0.71174400 1.47937700 4.27923900
H 0.89188200 2.28295600 4.99268300
C 5.44469000 -1.18319400 0.60676800
C 6.81583000 -1.45803700 0.62564500
C 5.73938400 0.09287000 -1.46975300
H 5.30849700 0.69235300 -2.27804400
C 7.11318100 -0.18491300 -1.44498100
H 7.76518800 0.19752200 -2.23470200
C 7.64966900 -0.95937700 -0.39844700
H 8.72142100 -1.17696000 -0.37898300
N 0.88495600 1.99308600 -0.14756800
N 0.41737800 -0.31219300 -1.76422800
C 0.87531100 1.96595500 -2.62685300

C 1.01520400 2.60619500 -1.39240800
C 1.37512300 4.25203800 0.12919500
H 1.58269700 5.19037900 0.64272700
C 1.04412500 2.83362100 2.18172100
C 0.59569900 0.60764800 -2.79546100
C 1.10261800 2.99715600 0.79490800
C 1.31894200 4.01082800 -1.22451900
H 1.47432700 4.71132400 -2.04461400
C 0.17591200 -1.53543900 -2.38609200
C 0.46630300 -0.05175100 -4.07820200
H 0.56713100 0.44840000 -5.04099800
C 0.20885400 -1.37785400 -3.82450700
H 0.05307800 -2.18762400 -4.53661300
O -1.61049300 0.54772200 0.17510800
C -3.39539600 -1.08911000 0.08298900
C -2.56956400 -0.08017800 0.84689900
H -2.27223700 -0.44668200 1.84841000
C -3.38857400 -1.09493800 -1.32807900
C -4.14306600 -2.04695300 -2.03347000
C -4.16421100 -2.04676400 0.77722100
H -4.16152900 -2.05239500 1.87264300
C -4.91957600 -2.99733100 0.07102400
H -5.50824500 -3.74027600 0.61707400
C -4.91207800 -2.99819300 -1.33665800
H -5.49792100 -3.74037500 -1.88662800
H -0.25119700 -3.62507800 -2.34640400
H 0.99897600 2.57154900 -3.52763100
H 1.24077700 3.71298700 2.79941900
H -0.15663700 -2.45406000 3.97319400
H 7.24317100 -2.05847900 1.43325300
H 4.77948300 -1.55779800 1.38852900
H -2.76926400 -0.36616800 -1.85520000
H -4.12569900 -2.05113600 -3.12754800
C -3.58814200 2.76237800 -0.43431100
C -4.33304200 1.86463000 0.42196900
C -3.71548200 1.22217400 1.53834400
H -2.96188300 1.77983400 2.10297800
H -4.38699200 0.65219300 2.18925600
C -5.69067700 1.38985300 -0.02503100
H -6.34977100 2.23879500 -0.27750800
H -5.59338200 0.78398200 -0.94737200
H -6.18501700 0.76925800 0.73501500
C -4.20491600 3.23141200 -1.74514500
H -4.48182300 2.37987800 -2.39076600
H -5.12569700 3.81516800 -1.57071200
H -3.50537400 3.86984900 -2.30588500
C -2.29075800 3.09661800 -0.12417900
H -1.82316800 2.87771800 0.83198600
H -1.67356600 3.65522800 -0.83197700

^{6}TS1A$_{endo}$

Fe 0.29482000 0.08422200 0.19965700
N 0.05836000 -1.93896200 0.61731100
N 0.48842000 0.46339300 2.24468800
O 2.58509400 -0.45154200 0.38761500
C -0.01014500 -1.81160000 3.09492100
C -0.09600600 -2.50858700 1.87486000
C -0.32856900 -4.20963000 0.37721900
H -0.47619300 -5.17222000 -0.11165400
C 4.85479800 -0.38220400 -0.38478000
C 0.02526000 -2.79963600 -1.70958100
C 0.27586900 -0.44310900 3.27271300
C -0.06933400 -2.96056400 -0.31508300
C -0.34770300 -3.93112100 1.72689600
H -0.51149600 -4.62330800 2.55252800
C 3.42962200 -0.04050200 -0.43223400
H 3.10827400 0.62669300 -1.25940300
C 0.77757600 1.68857100 2.82788100
C 0.41876700 0.23823800 4.54851700
H 0.30342400 -0.23509600 5.52340000
C 0.73139100 1.55201000 4.27419500
H 0.92053300 2.35876700 4.98209400
C 5.37059300 -1.23324900 0.62588600
C 6.73453800 -1.54191700 0.64323800
C 5.71997500 0.15110000 -1.37140700
H 5.31273600 0.80612900 -2.14822000
C 7.08633100 -0.16127000 -1.34846300
H 7.75657000 0.24916700 -2.10831000
C 7.59189900 -1.00668000 -0.34223700
H 8.65788400 -1.25104800 -0.32404600
N 0.90684900 2.03609100 -0.21474100
N 0.47469000 -0.36104200 -1.83203300
C 0.90136400 1.92477700 -2.69380100
C 1.02864500 2.61478800 -1.47240400
C 1.38423100 4.28890300 0.03307300
H 1.58839900 5.24026000 0.52413300
C 1.06336500 2.87242000 2.12110200
C 0.64629200 0.55097800 -2.86515000
C 1.12291800 3.03946000 0.72372900

C 1.32225500 4.02843300 -1.32053900
H 1.47085800 4.72628200 -2.14449900
C 0.26813300 -1.60613300 -2.41371500
C 0.54490200 -0.14280000 -4.13821100
H 0.64297200 0.33147000 -5.11458200
C 0.31501400 -1.47220800 -3.85941900
H 0.18580200 -2.29336700 -4.56418700
O -1.63846200 0.49186700 0.13771600
C -3.44934400 -1.11062500 0.07601600
C -2.59302900 -0.12472700 0.82419800
H -2.29221200 -0.48255400 1.82675500
C -3.44600500 -1.13407500 -1.33581300
C -4.22333200 -2.07873600 -2.02546500
C -4.23803500 -2.04078100 0.78671000
H -4.23141500 -2.03215100 1.88185200
C -5.01720300 -2.98163500 0.09485800
H -5.62174900 -3.70329300 0.65167200
C -5.01272000 -3.00124500 -1.31295500
H -5.61705000 -3.73704200 -1.85118800
H -0.11614700 -3.70030400 -2.31242100
H 1.02545000 2.51587300 -3.60503800
H 1.26399500 3.76235200 2.72367600
H -0.15172700 -2.40135200 4.00443600
H 7.13788400 -2.19786900 1.41941900
H 4.68762800 -1.63586100 1.37782200
H -2.81148800 -0.42767300 -1.87550800
H -4.20853500 -2.09930000 -3.11917700
C -3.54403000 2.80603400 -0.39782900
C -4.30284100 1.89027400 0.43550300
C -3.71415600 1.23098800 1.55049800
H -2.93146200 1.74773900 2.11316200
H -4.39535500 0.66229300 2.19141000
C -5.65725000 1.43360800 -0.03648600
H -6.30731800 2.29108500 -0.28278700
H -5.54902300 0.84523600 -0.96914500
H -6.16497700 0.80164000 0.70481800
C -4.15291300 3.31090800 -1.69855100
H -4.43270600 2.47706100 -2.36546800
H -5.07026000 3.89671200 -1.51372900
H -3.44599400 3.95730200 -2.24041500
C -2.25221000 3.13420300 -0.06971100
H -1.78403600 2.87008100 0.87533700
H -1.63333300 3.72158600 -0.75212100

[4]TS2A$_{endo}$

Fe 0.92527800 0.76821000 0.57979700
N 1.53302900 1.95453000 -0.94020300
N 2.68200600 -0.22991300 0.47633600
C 3.62267500 0.78389900 -1.58303100
C 2.64708200 1.76422100 -1.76271100
C 1.54039000 3.52618700 -2.66472100
H 1.20947600 4.37044900 -3.26855700
C -0.34324400 3.57425100 -1.00076400
C 3.64387000 -0.13452600 -0.53261000
C 0.84703100 3.03865200 -1.49439800
C 2.65334700 2.73696200 -2.83131700
H 3.42281100 2.80119700 -3.60003500
C 3.11704900 -1.26649600 1.30474100
C 4.67740300 -1.12468500 -0.33535100
H 5.54169900 -1.25002200 -0.98666100
C 4.35302500 -1.82336900 0.80385400
H 4.89778900 -2.63849700 1.27909500
N 0.55586800 -0.12693900 2.35814400
N -0.59715600 2.04798900 0.93674900
C -1.55238200 1.01896100 2.98015600
C -0.55701900 0.06293900 3.17942500
C 0.59394300 -1.64213100 4.13138300
H 0.95610400 -2.44951900 4.76682200
C 2.45817200 -1.71329900 2.44969700
C -1.56326100 1.94899600 1.94223600
C 1.26727300 -1.17741900 2.93900100
C -0.53667800 -0.87575800 4.27871700
H -1.29354700 -0.92294000 5.06093500
C -1.00867200 3.11619100 0.13588800
C -2.58129200 2.95699800 1.76130200
H -3.44200100 3.08513200 2.41646500
C -2.23801200 3.68017400 0.64427200
H -2.75893800 4.52479900 0.19473900
O -0.10554500 -0.50745500 -0.50992900
C -2.48745300 -0.41306700 -0.91805900
C -1.06787200 -0.19309600 -1.36853300
H -0.92458100 0.76335700 -1.90683600
C -2.76195600 -1.24136100 0.19194400
C -4.08494400 -1.41857700 0.62761000
C -3.54989700 0.23399800 -1.58442800
H -3.33677600 0.88656000 -2.43786200
C -4.87253900 0.05368500 -1.14818900
H -5.69086900 0.56264000 -1.66537700
C -5.14261800 -0.77435600 -0.04232300

H -6.17244500 -0.91153800 0.29948500
H -0.77804600 4.42166500 -1.53520400
H -2.37418800 1.05258700 3.69848800
H 2.91200300 -2.53543500 3.00728000
H 4.44222600 0.74693700 -2.30410900
H -1.92929000 -1.71678700 0.71519200
H -4.29161800 -2.05377700 1.49402100
C 0.03197300 -3.30228400 -1.74601100
C -0.94342900 -2.59040800 -2.55289400
C -0.75516500 -1.23274200 -2.93443100
H 0.26132200 -0.87256100 -3.11503900
H -1.48417100 -0.82932600 -3.64420400
C -2.28838900 -3.22308200 -2.79162600
H -2.17812300 -4.20709800 -3.28256800
H -2.80284300 -3.41298100 -1.83028600
H -2.94067600 -2.59180700 -3.41038100
C -0.32310200 -4.67326200 -1.18770200
H -1.22006900 -4.62942700 -0.54555100
H -0.54225200 -5.39282500 -1.99561800
H 0.50482100 -5.08196300 -0.58953200
C 1.22248200 -2.70844000 -1.40707600
H 1.57273900 -1.77099300 -1.83201700
H 1.90358900 -3.18784600 -0.69946100

^{6}TS2A$_{endo}$

Fe 0.88514000 0.80989200 0.20456200
N 1.59415000 1.25130000 -1.72034000
N 2.70830300 -0.15869400 0.56342200
C 3.61300600 -0.19024200 -1.74755800
C 2.67871400 0.65581200 -2.36396400
C 1.62182100 1.89287600 -3.95423600
H 1.32028800 2.39698900 -4.87206000
C -0.18641900 2.82246600 -2.43674800
C 3.63864800 -0.56030300 -0.39309400
C 0.94279800 2.02372300 -2.67992800
C 2.69345600 1.05067400 -3.75880300
H 3.43860800 0.72902700 -4.48581700
C 3.09987500 -0.74694900 1.76499900
C 4.62148500 -1.43227200 0.21829700
H 5.46114800 -1.88584900 -0.30754800
C 4.28706800 -1.55012300 1.54974700
H 4.80214000 -2.11525300 2.32614500
N 0.66019300 0.98037100 2.27651200
N -0.44196800 2.40861100 -0.00478600
C -1.28264200 2.52055200 2.32758400
C -0.36889600 1.64590300 2.93632200
C 0.69832800 0.42834300 4.53328500
H 1.02669800 -0.04043700 5.46048700
C 2.44990000 -0.57413100 2.99702200
C -1.31715300 2.88176200 0.97298800
C 1.32831600 0.23272200 3.24136500
C -0.34848200 1.30184400 4.34496700
H -1.04553500 1.68932400 5.08736900
C -0.82019300 3.01749300 -1.19938100
C -2.26576900 3.79872200 0.37361100
H -3.05891800 4.31164700 0.91662300
C -1.96058800 3.88180400 -0.96610500
H -2.45361500 4.47758900 -1.73372300
O -0.32866500 -0.64872400 -0.18068300
C -2.71932400 -0.75614100 -0.53198800
C -1.32160800 -0.65172300 -1.06522900
H -1.21735500 0.07466100 -1.89198800
C -2.95185200 -1.14080800 0.80726900
C -4.26449400 -1.20270600 1.30172300
C -3.81251800 -0.43866300 -1.36807300
H -3.63255900 -0.13281000 -2.40406100
C -5.12338600 -0.50560200 -0.87146000
H -5.96575500 -0.25282400 -1.52140400
C -5.35166200 -0.88894700 0.46408600
H -6.37321300 -0.93765600 0.85134000
H -0.59122300 3.37354000 -3.28946400
H -2.03774100 2.97157800 2.97597400
H 2.87363900 -1.10143700 3.85548100
H 4.41246800 -0.58390200 -2.38021600
H -2.09795300 -1.36458700 1.45110000
H -4.44000500 -1.49067700 2.34220100
C 0.02122100 -3.66606200 -0.51907800
C -1.06776400 -3.30381100 -1.41526800
C -1.00882900 -2.15295700 -2.24447900
H -0.03644000 -1.81133300 -2.60757200
H -1.81215200 -2.03742800 -2.97896300
C -2.37884600 -4.02997100 -1.28610400
H -2.23849300 -5.12348700 -1.34660600
H -2.82231500 -3.82904500 -0.29071200
H -3.10462800 -3.72030500 -2.04994600
C -0.18868700 -4.78298300 0.49293800
H -1.05154700 -4.57697300 1.14958900
H -0.38775200 -5.74667900 -0.00745400
H 0.70083100 -4.91073900 1.12767600

C 1.19388900 -2.95620100 -0.52533700
H 1.43176000 -2.19330200 -1.26381300
H 1.97407900 -3.15271800 0.21435000

PW6B95D3

^{4}IA

Fe -0.00036800 0.18557900 -0.01262500
N -0.05839800 2.16362700 -0.14954500
N -0.33648000 0.03299500 -1.96823300
O 2.16350000 0.25521900 -0.42057400
C -0.56779800 2.40345100 -2.52945300
C -0.32151300 2.90782000 -1.26952200
C 0.02565500 4.40255300 0.35968400
H 0.14944300 5.29171500 0.95729800
C 4.41068700 -0.38267000 -0.07329600
C 0.43112700 2.75673100 2.17247400
C -0.55227800 1.06147100 -2.84966500
C 0.15427800 3.06813200 0.85740900
C -0.27758600 4.30320800 -0.95835000
H -0.45208200 5.09329400 -1.67134800
C 2.98195900 -0.46074700 0.15362600
H 2.63351800 -1.20676800 0.88134000
C -0.35821000 -1.11363200 -2.72153600
C -0.72832800 0.55069100 -4.17261100
H -0.91663000 1.15993000 -5.04241900
C -0.59855900 -0.79788000 -4.09521300
H -0.66020600 -1.52707000 -4.88762300
C 4.93869200 0.55897500 -0.96480200
C 6.30448900 0.61597400 -1.16562800
C 5.25516400 -1.26097500 0.61102000
H 4.83271600 -1.98354700 1.29911500
C 6.62345700 -1.20121500 0.40352400
H 7.28417200 -1.87862100 0.92732500
C 7.14369500 -0.26366500 -0.48285300
H 8.21289100 -0.21638200 -0.64461800
N 0.05628000 -1.80698600 0.12614800
N 0.34159300 0.32235700 1.94313500
C 0.34713500 -2.05074500 2.54435600
C 0.18272000 -2.55629800 1.27031400
C 0.04490100 -4.04546700 -0.39639200
H 0.00205100 -4.93409900 -1.00628700
C -0.20756900 -2.39667200 -2.23560500
C 0.43390300 -0.70651400 2.84600200
C -0.03217400 -2.71214600 -0.90303400
C 0.16801800 -3.94949200 0.95221900
H 0.24939500 -4.74268800 1.67864800
C 0.49958700 1.47282400 2.67269400
C 0.66198300 -0.19216700 4.16012600
H 0.77024600 -0.80086900 5.04388000
C 0.71126400 1.15939000 4.05126000
H 0.86527300 1.89226000 4.82736000
O -2.16414400 0.20692100 0.40133800
C -4.41433100 -0.44980800 0.11569000
C -2.98825200 -0.53833000 -0.12462800
H -2.65242200 -1.32059100 -0.81977800
C -4.93279900 0.53113000 0.96958500
C -6.29643700 0.59810500 1.18176100
C -5.26631000 -1.35665200 -0.52032700
H -4.85146000 -2.10892900 -1.18064900
C -6.63245300 -1.28616700 -0.30206800
H -7.29887100 -1.98493800 -0.78925000
C -7.14315800 -0.31006500 0.54736700
H -8.21063400 -0.25431400 0.71759600
H 0.57642900 3.57445100 2.86401000
H 0.43499600 -2.75650800 3.35843000
H -0.25190000 -3.21144800 -2.94462300
H -0.75564000 3.10880800 -3.32645800
H 6.72352300 1.34021700 -1.85122700
H 4.26432600 1.22915100 -1.48097000
H -4.25319500 1.22334200 1.44837200
H -6.70794100 1.35256100 1.83874100

^{6}IA

Fe 0.00001800 0.23206800 0.00001000
N 0.00023100 2.26208300 0.00073600
N -0.24150300 0.21208500 -2.02921200
O 2.12549300 0.25537300 -0.28992100
C -0.30516900 2.63515600 -2.39939700
C -0.14400100 3.07326600 -1.09062900
C 0.09064600 4.44365100 0.67540200
H 0.17833600 5.29177200 1.33615000
C 4.34707500 -0.47221500 0.00333100
C 0.30566900 2.63342900 2.40112500
C -0.33867900 1.31540600 -2.83402000
C 0.14461000 3.07248800 1.09265800
C -0.08975200 4.44413000 -0.67242400
H -0.17727100 5.29272600 -1.33258600
C 2.92120200 -0.53097400 0.22884100
H 2.54530100 -1.31958700 0.89315700
C -0.28234600 -0.88639900 -2.84417300

C -0.45832400 0.89688000 -4.20025200
H -0.55154800 1.56505900 -5.04197700
C -0.42045500 -0.46243000 -4.20712500
H -0.47740400 -1.12643400 -5.05547400
C 4.89510400 0.51751000 -0.82313100
C 6.26022700 0.55192700 -1.02994900
C 5.17156900 -1.42047400 0.61666000
H 4.73367100 -2.17948200 1.25383200
C 6.53922300 -1.38171900 0.40313700
H 7.18470900 -2.11188400 0.87194900
C 7.07895800 -0.39700800 -0.41839700
H 8.14797400 -0.36702000 -0.58519300
N -0.00020800 -1.82880300 -0.00073200
N 0.24149800 0.21063400 2.02921000
C 0.21227700 -2.20830000 2.41244900
C 0.08606700 -2.64397600 1.09763900
C -0.05189100 -4.01331600 -0.67982200
H -0.10238300 -4.86185500 -1.34418000
C -0.21275500 -2.20657700 -2.41417600
C 0.28213200 -0.88843900 2.84339100
C -0.08664700 -2.64320300 -1.09966900
C 0.05102000 -4.01379300 0.67684700
H 0.10133200 -4.86280300 1.34061800
C 0.33889900 1.31336200 2.83480400
C 0.42032500 -0.46547700 4.20664300
H 0.47715400 -1.13009400 5.05451700
C 0.45846600 0.89383200 4.20073500
H 0.55182400 1.56139000 5.04293900
O -2.12549800 0.25574900 0.28992800
C -4.34710200 -0.47182100 -0.00337600
C -2.92123500 -0.53051700 -0.22891600
H -2.54538800 -1.31904700 -0.89336500
C -4.89520100 0.51780400 0.82314900
C -6.26032200 0.55206700 1.03001600
C -5.17151900 -1.42013300 -0.61673200
H -4.73355900 -2.17905700 -1.25396100
C -6.53916900 -1.38154500 -0.40314500
H -7.18459500 -2.11176100 -0.87196200
C -7.07897500 -0.39693400 0.41846200
H -8.14798600 -0.36707300 0.58531100
H 0.39966100 3.39666500 3.16178100
H 0.26405900 -2.97321200 3.17549700
H -0.26468000 -2.97093200 -3.17777300
H -0.39899700 3.39896600 -3.15949900
H 6.69496900 1.31178800 -1.66521900
H 4.23666500 1.24048100 -1.28595800
H -4.23682900 1.24082700 1.28599500
H -6.69512200 1.31185500 1.66533300

^{4}TS1A$_{endo}$

Fe 0.39767000 0.37799900 0.07320200
N 0.45155000 -1.46890300 0.85882100
N 0.42005900 1.15410300 1.91466500
O 2.70200400 0.40170300 0.16904700
C 0.43986900 -0.90310900 3.23948400
C 0.43650900 -1.79977200 2.18858300
C 0.32291600 -3.75129400 1.09503000
H 0.25930100 -4.78821900 0.80527400
C 4.79169000 -0.66490800 -0.14992300
C 0.37554200 -2.79232300 -1.19752900
C 0.42726900 0.47239900 3.10162400
C 0.37975400 -2.65587000 0.17685600
C 0.36439500 -3.22151200 2.34311400
H 0.34242300 -3.73309900 3.29234200
C 3.34080700 -0.58382700 -0.18298100
H 2.80861300 -1.47445700 -0.54880600
C 0.38679500 2.48300600 2.24345800
C 0.39144100 1.39088000 4.19907900
H 0.39191400 1.10172300 5.23820200
C 0.36045900 2.63833000 3.66720100
H 0.33328300 3.58747200 4.17872800
C 5.56051900 0.41908400 0.28658700
C 6.93859900 0.31233300 0.31101400
C 5.40936700 -1.84877900 -0.55920500
H 4.80101600 -2.68053200 -0.89454400
C 6.79094200 -1.95186300 -0.53130800
H 7.27585600 -2.86628400 -0.84560500
C 7.55139100 -0.87172200 -0.09696700
H 8.63068000 -0.95099000 -0.07499000
N 0.52295800 2.20702500 -0.70642800
N 0.53655500 -0.41533200 -1.75881000
C 0.64504600 1.63811600 -3.08263600
C 0.59731300 2.53452800 -2.03327700
C 0.51358400 4.49107200 -0.94520800
H 0.49019800 5.53038900 -0.65762900
C 0.39754500 3.53301200 1.34590000
C 0.60256900 0.26496200 -2.94491200
C 0.47804000 3.39422100 -0.02667700
C 0.59281600 3.95811300 -2.19085200

H 0.64829500 4.46894000 -3.13920300
C 0.45909700 -1.74226600 -2.09135500
C 0.58543700 -0.65234100 -4.04352000
H 0.63476600 -0.36316700 -5.08148300
C 0.49001300 -1.89766800 -3.51471400
H 0.44577000 -2.84570200 -4.02708800
O -1.62768500 0.36158900 -0.00915900
C -3.14119200 -1.43370600 0.35617700
C -2.37624100 -0.25172300 0.85213200
H -1.95067700 -0.39731100 1.85331200
C -3.24676700 -1.64864400 -1.01666700
C -3.94364700 -2.74948500 -1.49582500
C -3.72378200 -2.33430300 1.24414800
H -3.62496300 -2.17549800 2.31264200
C -4.42006700 -3.43483900 0.76324800
H -4.86905300 -4.13450200 1.45651000
C -4.53428000 -3.64186700 -0.60731000
H -5.07593000 -4.50138700 -0.98083700
H 7.54209200 1.14526100 0.64645100
H 5.06038400 1.32610000 0.59903200
H -2.76158600 -0.95316300 -1.68979300
H -4.02021700 -2.91709500 -2.56281100
C -3.99256300 1.95473100 -0.72241500
C -4.44858800 1.23596200 0.43248400
C -3.60802100 1.00467000 1.52755300
H -2.85718700 1.73942700 1.79228500
H -4.05569100 0.51404500 2.38448100
C -5.74922400 0.51595200 0.35558300
H -6.55876700 1.22343600 0.15361200
H -5.74946200 -0.19735600 -0.47447500
H -5.97382900 -0.02154000 1.27384800
C -4.80009100 1.90378300 -1.98847700
H -4.92819100 0.87631900 -2.33820100
H -5.79927000 2.32097600 -1.84349800
H -4.31311300 2.47169600 -2.77972100
C -2.78167200 2.56102500 -0.69343900
H -2.21989400 2.70720700 0.21426800
H -2.34991600 2.99030600 -1.58972700
H 0.31029500 -3.79267800 -1.60176200
H 0.36683500 4.53654800 1.74666300
H 0.43309000 -1.30558000 4.24276100
H 0.69959100 2.04001700 -4.08466100

^{6}TS1A$_{endo}$

Fe 0.33998000 0.35285300 0.09087800
N 0.43095700 -1.55163700 0.89801000
N 0.50834800 1.13964600 1.98262800
O 2.59384800 0.33904000 0.14582600
C 0.44823700 -0.93925800 3.27200000
C 0.40496500 -1.85997000 2.22957900
C 0.25102600 -3.81936400 1.13661800
H 0.16646600 -4.85858000 0.85983100
C 4.68857600 -0.70123100 -0.21537500
C 0.32211400 -2.84700700 -1.17814400
C 0.50706200 0.44725900 3.15826300
C 0.33559700 -2.72823900 0.20800400
C 0.29546300 -3.28424400 2.38515000
H 0.25620500 -3.79956600 3.33208800
C 3.23848500 -0.64030700 -0.21832500
H 2.71056700 -1.53632700 -0.57112700
C 0.58100500 2.46819400 2.29023300
C 0.56919800 1.37321700 4.25472600
H 0.58356500 1.09492000 5.29693300
C 0.61468600 2.62163700 3.71908400
H 0.67429700 3.56626800 4.23670100
C 5.45469600 0.39176200 0.20382600
C 6.83393500 0.30024500 0.19546500
C 5.30973400 -1.87842300 -0.63977500
H 4.70274600 -2.71681600 -0.96066300
C 6.69240800 -1.96570800 -0.64482800
H 7.18020900 -2.87430400 -0.97120200
C 7.45015900 -0.87677000 -0.22791700
H 8.53051800 -0.94342900 -0.23205200
N 0.56775900 2.21536400 -0.71481600
N 0.48963300 -0.48077000 -1.78720600
C 0.57263000 1.59725300 -3.08329600
C 0.59941900 2.52193400 -2.04454600
C 0.68075100 4.48466200 -0.94861200
H 0.73439800 5.52596300 -0.67148800
C 0.62426300 3.50723900 1.36565800
C 0.51461600 0.21288700 -2.96410700
C 0.62694400 3.39040600 -0.02108300
C 0.66434600 3.94899100 -2.19909500
H 0.70403800 4.46621300 -3.14503500
C 0.39296100 -1.80756600 -2.10010500
C 0.44775600 -0.71107600 -4.06171800
H 0.45625100 -0.43104000 -5.10354000
C 0.37055800 -1.95889500 -3.52888900
H 0.30318300 -2.90230100 -4.04777100

O -1.62440700 0.37334700 0.08598000
C -3.16153100 -1.41993500 0.37846500
C -2.40690600 -0.25449000 0.90256000
H -2.05032300 -0.37977900 1.93102300
C -3.23539300 -1.63405700 -0.99728600
C -3.90354000 -2.74570200 -1.49052200
C -3.75460900 -2.32674700 1.25481000
H -3.67954100 -2.16714900 2.32481200
C -4.42455300 -3.43565500 0.75875600
H -4.88031300 -4.14087200 1.44155300
C -4.50132900 -3.64526000 -0.61420300
H -5.02129100 -4.51282000 -0.99951200
H 7.43596200 1.13972300 0.51680400
H 4.95172100 1.29291300 0.52841000
H -2.75062700 -0.93186000 -1.66341700
H -3.95321600 -2.91469800 -2.55859300
C -3.88731100 2.17829100 -0.66260700
C -4.42443700 1.33757300 0.38171200
C -3.70996000 1.04477800 1.53927900
H -2.96626500 1.73703000 1.91143700
H -4.22971600 0.49158700 2.31292700
C -5.68517800 0.59310200 0.11691500
H -6.48550700 1.27727800 -0.17581800
H -5.54404500 -0.10174700 -0.71823800
H -6.00853700 0.02197900 0.98401900
C -4.61523900 2.27526600 -1.97356600
H -4.74209200 1.29506300 -2.43886800
H -5.61353700 2.70052000 -1.84631900
H -4.06848600 2.90998200 -2.66903700
C -2.70037100 2.79923900 -0.48767900
H -2.17277300 2.81113000 0.45303800
H -2.23390100 3.35008800 -1.29575100
H 0.24059600 -3.84850200 -1.57891000
H 0.67982700 4.51057300 1.76672800
H 0.43529900 -1.34282600 4.27572700
H 0.59504600 1.99570600 -4.08881400

^{4}TS2A$_{endo}$

Fe 0.95146900 0.73650900 0.59592100
N 1.75539600 1.75766400 -0.92151700
N 2.57009200 -0.42361100 0.66858700
C 3.73036200 0.37185900 -1.33530300
C 2.88305300 1.41866400 -1.62804700
C 2.04328700 3.19664800 -2.69565200
H 1.84271000 4.02236500 -3.35992100
C 0.07048200 3.50540800 -1.21942200
C 3.58161100 -0.47557800 -0.25761800
C 1.22961100 2.84652600 -1.57349800
C 3.06830000 2.30933500 -2.73016900
H 3.88841300 2.25437000 -3.42863500
C 2.82623800 -1.44440500 1.55030600
C 4.47734600 -1.54740000 0.04195900
H 5.35554700 -1.78927600 -0.53566300
C 4.00893200 -2.14891300 1.16507400
H 4.42274000 -2.98706400 1.70317800
N 0.38140700 -0.00626400 2.35909000
N -0.45591600 2.14496700 0.74388400
C -1.62377400 1.34407600 2.74085000
C -0.74222600 0.33655100 3.06765600
C 0.14145700 -1.38925200 4.18352100
H 0.37116800 -2.18228400 4.87741300
C 2.06355200 -1.75466600 2.65664000
C -1.48170100 2.18168000 1.65636100
C 0.92741000 -1.07044500 3.03178000
C -0.89541400 -0.51614800 4.20481200
H -1.69883500 -0.43987500 4.92035700
C -0.71295300 3.16910600 -0.13506400
C -2.39709300 3.23033800 1.33687000
H -3.28460600 3.45889200 1.90519800
C -1.91947500 3.84492200 0.22614100
H -2.33095600 4.68611900 -0.30880200
O -0.11623200 -0.50130200 -0.49855400
C -2.46527100 -0.36685300 -0.87673200
C -1.06088000 -0.13832200 -1.30491400
H -0.91604100 0.80650800 -1.84204200
C -2.73132400 -1.17381300 0.22828500
C -4.03978800 -1.35444400 0.65476400
C -3.51413400 0.26080100 -1.54566000
H -3.30260700 0.89991000 -2.39613600
C -4.82138300 0.07638300 -1.11928800
H -5.63362600 0.56828200 -1.63849700
C -5.08564200 -0.73351100 -0.01967800
H -6.10523900 -0.87379100 0.31525500
H -0.23899600 4.34470800 -1.82609400
H -2.48086700 1.49205900 3.38223700
H 2.38185500 -2.58260700 3.27416500
H 4.58146400 0.21679000 -1.98341800
H -1.90266200 -1.62748200 0.75735500
H -4.24446200 -1.97395100 1.51866200

C -0.07717400 -3.18301000 -1.79953000
C -1.02403700 -2.41980900 -2.57577900
C -0.78093900 -1.10090100 -2.95197300
H 0.23543000 -0.75345400 -3.08505200
H -1.49829300 -0.64291700 -3.62321800
C -2.37875600 -2.99064000 -2.80273500
H -2.30285800 -3.95356000 -3.31586000
H -2.87813800 -3.18823100 -1.84879700
H -3.00770200 -2.32579900 -3.38991500
C -0.49384000 -4.51174600 -1.23561000
H -1.36746900 -4.41797500 -0.58668100
H -0.75570400 -5.21806500 -2.02664700
H 0.31386900 -4.94958600 -0.65138600
C 1.13796500 -2.66508600 -1.51478700
H 1.50817000 -1.75147400 -1.95196800
H 1.81968400 -3.18483600 -0.85227600

^{6}TS2A$_{endo}$

Fe 0.88046900 0.68444700 0.50195600
N 1.69737800 1.78253400 -1.04122200
N 2.65720900 -0.33294400 0.65184700
C 3.72188300 0.45300300 -1.40844300
C 2.82643400 1.45617600 -1.74679700
C 1.88795100 3.16192400 -2.86458700
H 1.64848400 3.95343100 -3.55720900
C -0.07015800 3.45027400 -1.32907400
C 3.65097700 -0.36608400 -0.29088200
C 1.10848100 2.82487600 -1.70919100
C 2.94979400 2.31540500 -2.88764100
H 3.75572900 2.27496000 -3.60359700
C 2.97485400 -1.28119400 1.58966300
C 4.61149500 -1.37126200 0.05459200
H 5.49700400 -1.59845900 -0.51810500
C 4.19413700 -1.93651500 1.21856500
H 4.66984400 -2.71903200 1.78860300
N 0.44041700 0.07817000 2.41655900
N -0.52541500 2.17663700 0.71086700
C -1.59849300 1.38521200 2.76633000
C -0.67751400 0.41587900 3.13172500
C 0.32031300 -1.21420700 4.30764500
H 0.60036000 -1.95608800 5.03876800
C 2.23324700 -1.55697400 2.72937600
C -1.52703300 2.19960700 1.64699100
C 1.06386900 -0.92074300 3.11679300
C -0.75811700 -0.38901600 4.31511600
H -1.54023600 -0.31819500 5.05465100
C -0.82904600 3.14892000 -0.20699900
C -2.48745800 3.20547200 1.30423900
H -3.37278200 3.42962700 1.87811900
C -2.05460200 3.79529900 0.15958700
H -2.51391700 4.60057900 -0.39181800
O -0.17935200 -0.54228300 -0.51823100
C -2.53334800 -0.46129100 -0.84529300
C -1.15371900 -0.21046100 -1.30201200
H -1.02468000 0.69661200 -1.90030600
C -2.76024800 -1.26184100 0.27527100
C -4.05247900 -1.44030200 0.74546600
C -3.60512600 0.15734800 -1.48859000
H -3.42240200 0.78847600 -2.35105200
C -4.89602100 -0.02677000 -1.01716100
H -5.72654500 0.45734500 -1.51373900
C -5.12031800 -0.82569200 0.09939100
H -6.12786800 -0.96431000 0.46932700
H -0.42454000 4.25406200 -1.96010900
H -2.44567900 1.52906600 3.42305300
H 2.60982000 -2.33152300 3.38361500
H 4.57046400 0.31365200 -2.06468700
H -1.91627000 -1.72202400 0.77355000
H -4.22834600 -2.05412200 1.61930800
C 0.02352200 -3.23538600 -1.74415800
C -0.99632100 -2.55306300 -2.51694900
C -0.83426800 -1.26228400 -2.99056800
H 0.15149200 -0.84084500 -3.13622500
H -1.60126100 -0.86500300 -3.64449300
C -2.33076200 -3.19821200 -2.65232200
H -2.23492300 -4.16761500 -3.14980200
H -2.76477100 -3.39657900 -1.66738800
H -3.02392000 -2.58025900 -3.21853300
C -0.31358700 -4.54954400 -1.09826300
H -1.14912800 -4.45472600 -0.40070200
H -0.59798900 -5.30125200 -1.83777100
H 0.54168400 -4.93271400 -0.54446000
C 1.23320600 -2.67128500 -1.54704700
H 1.55185000 -1.75436200 -2.01969900
H 1.96473500 -3.15245100 -0.90929500

Fe(III)Cl complex

^{2}IVA(Cl-)

Fe -0.000002 0.000092 -0.016968
N 1.413043 1.406087 -0.237655

N	-1.414794	1.404311	-0.237355
C	-0.002140	3.411778	-0.161965
C	1.226098	2.772126	-0.193056
C	3.457770	2.497446	-0.259613
H	4.533068	2.619613	-0.287673
C	3.426215	0.002112	-0.283198
C	-1.229531	2.770562	-0.192606
C	2.781779	1.227513	-0.265527
C	2.492710	3.455985	-0.209776
H	2.607921	4.532323	-0.190547
C	-2.783337	1.224105	-0.264991
C	-2.497006	3.452834	-0.208658
H	-2.613585	4.529023	-0.189160
C	-3.460902	2.493173	-0.258583
H	-4.536364	2.613966	-0.286411
N	-1.413066	-1.406092	-0.237640
N	1.414763	-1.404339	-0.237296
C	0.002068	-3.411710	-0.161968
C	-1.226116	-2.772037	-0.193165
C	-3.457731	-2.497395	-0.259767
H	-4.533036	-2.619522	-0.287859
C	-3.426145	-0.002147	-0.283096
C	1.229455	-2.770530	-0.192537
C	-2.781667	-1.227484	-0.265535
C	-2.492721	-3.455909	-0.209779
H	-2.607957	-4.532242	-0.190537
C	2.783296	-1.224126	-0.265091
C	2.496900	-3.452868	-0.208726
H	2.613459	-4.529051	-0.189217
C	3.460856	-2.493234	-0.258702
H	4.536310	-2.614060	-0.286630
H	4.511411	0.002860	-0.300665
H	0.002843	-4.496581	-0.129032
H	-4.511344	-0.002956	-0.300404
H	-0.002966	4.496657	-0.129045
Cl	0.000091	-0.000197	2.202118

^{4}IVA(Cl-)

Fe	-0.000001	0.000001	0.050677
N	-1.118891	1.654351	-0.241199
N	-1.654350	-1.118892	-0.241208
C	-3.362026	0.648930	-0.248610
C	-2.496441	1.730200	-0.250986
C	-1.793257	3.866587	-0.275496
H	-1.705585	4.945443	-0.292594
C	0.648935	3.362019	-0.248630
C	-2.961306	-0.676802	-0.251307
C	-0.676798	2.961307	-0.251316
C	-2.921470	3.103206	-0.275148
H	-3.955590	3.422991	-0.292025
C	-1.730194	-2.496437	-0.251016
C	-3.866583	-1.793263	-0.275486
H	-4.945440	-1.705595	-0.292566
C	-3.103197	-2.921472	-0.275190
H	-3.422978	-3.955592	-0.292092
N	1.118894	-1.654347	-0.241195
N	1.654355	1.118887	-0.241178
C	3.362019	-0.648930	-0.248569
C	2.496434	-1.730192	-0.250967
C	1.793258	-3.866574	-0.275499
H	1.705588	-4.945430	-0.292601
C	-0.648924	-3.362016	-0.248656
C	2.961305	0.676797	-0.251257
C	0.676802	-2.961294	-0.251329
C	2.921467	-3.103194	-0.275155
H	3.955586	-3.422978	-0.292036
C	1.730200	2.496431	-0.250983
C	3.866584	1.793253	-0.275438
H	4.945440	1.705584	-0.292518
C	3.103204	2.921464	-0.275112
H	3.422987	3.955585	-0.291988
H	0.854591	4.427336	-0.254427
H	4.427335	-0.854584	-0.254348
H	-0.854574	-4.427333	-0.254464
H	-4.427344	0.854581	-0.254394
Cl	-0.000007	-0.000008	2.354636

^{6}IVA(Cl-)

Fe	-0.000068	-0.000073	0.227385
N	1.727235	1.076884	-0.254020
N	-1.076869	1.727189	-0.254281
C	0.775459	3.344114	-0.289452
C	1.842344	2.448871	-0.284690
C	3.956218	1.662868	-0.333945
H	5.031575	1.541714	-0.369735
C	3.344181	-0.775439	-0.286801
C	-0.576350	3.009834	-0.286357
C	3.009889	0.576343	-0.284366
C	3.234465	2.820516	-0.334000
H	3.599113	3.839401	-0.369952

C	-2.448933	1.842339	-0.283650
C	-1.662911	3.956109	-0.335985
H	-1.541781	5.031433	-0.373070
C	-2.820596	3.234400	-0.333988
H	-3.839511	3.599035	-0.369268
N	-1.727235	-1.076875	-0.254167
N	1.077019	-1.727133	-0.254342
C	-0.775338	-3.344052	-0.289474
C	-1.842292	-2.448863	-0.284689
C	-3.956244	-1.663047	-0.333882
H	-5.031640	-1.541985	-0.369589
C	-3.344262	0.775449	-0.286887
C	0.576449	-3.009666	-0.286402
C	-3.009955	-0.576414	-0.284435
C	-3.234366	-2.820649	-0.333949
H	-3.598946	-3.839555	-0.369884
C	2.448909	-1.842259	-0.283622
C	1.662994	-3.956043	-0.336016
H	1.541820	-5.031373	-0.373107
C	2.820634	-3.234326	-0.333851
H	3.839548	-3.598956	-0.369037
H	4.401533	-1.020802	-0.311576
H	-1.020655	-4.401407	-0.315488
H	-4.401631	1.020855	-0.311689
H	1.020827	4.401437	-0.315512
Cl	-0.000075	0.000065	2.474248

^{2}IA(Cl-)

Fe	0.970536	-0.062828	-0.300866
N	0.073231	0.841190	-1.876912
N	1.448793	1.750734	0.437695
O	-0.997806	0.199230	0.845293
C	0.550397	3.189034	-1.337956
C	0.035269	2.188951	-2.151854
C	-0.920328	1.191782	-3.931185
H	-1.402733	0.954926	-4.871405
C	-3.371961	0.159113	0.943113
C	-0.679556	-1.158433	-3.093436
C	1.220181	2.976793	-0.140220
C	-0.495722	0.212962	-2.961099
C	-0.597403	2.415615	-3.426935
H	-0.756606	3.391414	-3.868498
C	-2.074693	-0.034685	0.296333
H	-2.089450	-0.408583	-0.737946
C	2.163005	1.994089	1.585371
C	1.794029	4.021028	0.672272
H	1.747053	5.077068	0.437808
C	2.383172	3.412026	1.738099
H	2.918762	3.864256	2.563402
C	-3.457625	0.628119	2.265797
C	-4.704037	0.804011	2.854410
C	-4.540279	-0.130209	0.220165
H	-4.462957	-0.491387	-0.802664
C	-5.787596	0.048782	0.814111
H	-6.693498	-0.172868	0.257534
C	-5.867441	0.514251	2.129408
H	-6.839954	0.652964	2.594184
N	1.652246	-0.946573	1.378846
N	0.267997	-1.852426	-0.932554
C	1.028768	-3.275769	0.919747
C	1.614124	-2.283453	1.694693
C	2.747953	-1.306665	3.377497
H	3.304695	-1.075982	4.277235
C	2.592018	1.031667	2.490537
C	0.418647	-3.070939	-0.310804
C	2.343128	-0.331384	2.393933
C	2.290446	-2.515002	2.947570
H	2.395442	-3.484141	3.418923
C	-0.329831	-2.111992	-2.144671
C	-0.120526	-4.118651	-1.140434
H	-0.130537	-5.168242	-0.874536
C	-0.579274	-3.526021	-2.278216
H	-1.045715	-3.987964	-3.139406
H	-1.147309	-1.513043	-4.006696
H	1.077508	-4.294881	1.290459
H	3.151503	1.375361	3.355004
H	0.448475	4.214360	-1.679616
H	-4.776965	1.165321	3.876115
H	-2.542509	0.846561	2.807374
Cl	2.915781	-0.294474	-1.379065

^{4}IA(Cl-)

Fe	1.054852	-0.111514	-0.320019
N	-0.069652	-0.012411	-1.999616
N	1.217022	1.898024	-0.328484
O	-1.064864	0.366089	0.875077
C	0.103317	2.395914	-2.461706
C	-0.296901	1.104723	-2.772054
C	-1.204158	-0.605104	-3.922098
H	-1.699054	-1.242110	-4.644332

C	-3.440474	0.209461	0.977072
C	-0.623114	-2.391984	-2.271964
C	0.810320	2.755387	-1.323913
C	-0.611903	-1.070698	-2.695810
C	-1.012949	0.743604	-3.967066
H	-1.317210	1.443831	-4.734788
C	-2.105236	-0.152343	0.485512
H	-2.070769	-0.938353	-0.288049
C	1.874345	2.675351	0.596729
C	1.218883	4.101749	-1.016221
H	1.016931	4.961987	-1.641900
C	1.880267	4.051626	0.173531
H	2.335399	4.861850	0.729139
C	-3.598234	1.182172	1.978812
C	-4.872871	1.519297	2.419165
C	-4.566319	-0.416451	0.421091
H	-4.434137	-1.166576	-0.355396
C	-5.842921	-0.073786	0.862309
H	-6.716355	-0.555222	0.431989
C	-5.994368	0.892867	1.860145
H	-6.989566	1.160808	2.204975
N	1.832413	-0.148519	1.538477
N	0.531172	-2.054891	-0.127749
C	1.573552	-2.538512	2.045790
C	2.013369	-1.255198	2.334513
C	2.976730	0.443764	3.453598
H	3.490055	1.079010	4.164343
C	2.442957	2.218590	1.776189
C	0.885995	-2.902359	0.897438
C	2.414026	0.901531	2.209517
C	2.727167	-0.893062	3.531869
H	2.993703	-1.585718	4.320178
C	-0.095360	-2.840994	-1.069621
C	0.456276	-4.244004	0.602204
H	0.612373	-5.094943	1.253202
C	-0.148832	-4.206727	-0.618307
H	-0.594614	-5.020167	-1.176851
H	-1.087001	-3.126029	-2.922929
H	1.789831	-3.317862	2.769320
H	2.936246	2.946777	2.411756
H	-0.148337	3.182171	-3.166073
H	-4.999968	2.269702	3.194311
H	-2.713343	1.655679	2.392935
Cl	3.078264	-0.526381	-1.425923

[6]IA(Cl-)

Fe	-1.164259	-0.125632	-0.402459
N	-0.497056	-2.093130	-0.181692
N	-1.846559	-0.191612	1.558847
O	1.007794	0.388190	0.845373
C	-1.563725	-2.597271	1.979193
C	-0.865422	-2.953984	0.824537
C	0.204058	-4.219767	-0.711696
H	0.663558	-5.022386	-1.275180
C	3.370577	0.163310	1.051213
C	0.708152	-2.334884	-2.312506
C	-2.012562	-1.320651	2.320976
C	0.152811	-2.840894	-1.135504
C	-0.422614	-4.288989	0.499412
H	-0.579850	-5.160397	1.122893
C	2.031815	-0.233171	0.591447
H	1.982797	-1.148852	-0.022434
C	-2.407568	0.852045	2.251732
C	-2.713033	-0.979941	3.537412
H	-2.974151	-1.683970	4.317673
C	-2.957306	0.362097	3.494315
H	-3.458238	0.976141	4.232298
C	3.549022	1.326445	1.818608
C	4.824427	1.690417	2.235203
C	4.476924	-0.626283	0.704191
H	4.329325	-1.523363	0.106833
C	5.754230	-0.259365	1.123976
H	6.612601	-0.869392	0.857355
C	5.926259	0.897982	1.887894
H	6.922088	1.185855	2.214471
N	-1.211659	1.948698	-0.295637
N	0.135733	0.040606	-2.030182
C	-0.045825	2.467872	-2.396780
C	-0.774472	2.821069	-1.260032
C	-1.835237	4.085047	0.282475
H	-2.273749	4.888915	0.860618
C	-2.419957	2.183366	1.833152
C	0.374261	1.185181	-2.752309
C	-1.856427	2.691535	0.661796
C	-1.166160	4.165402	-0.904247
H	-0.947307	5.048345	-1.491884
C	0.708655	-0.999988	-2.721427
C	1.131796	0.856374	-3.936669
H	1.458785	1.573748	-4.679064

C	1.335682	-0.493340	-3.919366
H	1.863530	-1.100512	-4.644240
H	1.198557	-3.048160	-2.968297
H	0.226842	3.274097	-3.071334
H	-2.899861	2.898716	2.494293
H	-1.778784	-3.394718	2.684216
H	4.968168	2.589573	2.827760
H	2.678529	1.924208	2.071368
Cl	-3.111637	-0.516267	-1.504572

^{4}TS1A$_{endo}$(Cl-)

Fe	-1.275793	-0.608138	0.313698
N	0.042669	-2.142943	0.331991
N	-1.618113	-0.950579	-1.651836
C	-0.306687	-3.009001	-1.940344
C	0.264293	-3.051037	-0.675472
C	1.578678	-3.716579	1.028224
H	2.279866	-4.219411	1.682111
C	0.917389	-1.897681	2.613886
C	-1.176015	-2.022883	-2.390193
C	0.834102	-2.535170	1.384287
C	1.221539	-4.039751	-0.245893
H	1.567701	-4.864266	-0.856398
C	-2.438393	-0.223803	-2.481520
C	-1.723568	-1.963226	-3.721921
H	-1.528823	-2.691826	-4.498878
C	-2.501712	-0.845731	-3.780062
H	-3.080859	-0.468579	-4.613667
N	-2.388311	1.073853	0.211257
N	-0.708024	-0.105206	2.185368
C	-2.111016	1.884010	2.514131
C	-2.661588	1.942474	1.240456
C	-3.830546	2.716830	-0.523289
H	-4.467527	3.269313	-1.202648
C	-3.117951	0.936143	-2.132279
C	-1.189540	0.936464	2.939347
C	-3.099814	1.525548	-0.874699
C	-3.563731	2.971871	0.788915
H	-3.933589	3.779062	1.408746
C	0.192885	-0.771733	2.979582
C	-0.574874	0.927214	4.243591
H	-0.785891	1.644861	5.026512
C	0.285536	-0.128673	4.266805
H	0.928266	-0.459768	5.072861
O	0.497813	0.682026	-0.320476
C	2.780507	-0.005343	-0.534871
C	1.417920	0.240172	-1.122664
H	1.105670	-0.537532	-1.833612
C	3.087818	0.502796	0.732701
C	4.347964	0.285246	1.291321
C	3.743300	-0.746862	-1.228981
H	3.500857	-1.157488	-2.207264
C	5.002580	-0.967552	-0.670351
H	5.742922	-1.548197	-1.214920
C	5.309836	-0.447740	0.590407
H	6.289943	-0.620938	1.027221
H	2.318235	1.047380	1.269612
H	4.577043	0.679362	2.278408
C	1.444407	3.308888	-0.855038
C	2.191226	2.636983	-1.861375
C	1.640302	1.517056	-2.524378
H	0.587584	1.525727	-2.793656
H	2.264785	1.040016	-3.277254
C	3.668022	2.894448	-1.973271
H	3.876931	3.964890	-2.087253
H	4.182451	2.563369	-1.060359
H	4.111465	2.362077	-2.817984
C	2.141652	4.249923	0.103877
H	2.975665	3.761936	0.623102
H	2.554996	5.120726	-0.420414
H	1.445642	4.623034	0.860911
C	0.145410	2.921069	-0.624691
H	-0.481167	2.459605	-1.372428
H	-0.372889	3.234152	0.275810
H	1.596138	-2.317199	3.349301
H	-3.730434	1.405934	-2.895372
H	-0.040206	-3.797709	-2.636656
H	-2.410658	2.646796	3.225717
Cl	-3.100185	-1.962474	1.013012

^{6}TS1A$_{endo}$(Cl-)

Fe	-1.251205	-0.699570	0.428479
N	0.175236	-2.206076	0.220968
N	-1.766376	-0.966824	-1.573945
C	-0.407596	-2.962755	-2.045398
C	0.299545	-3.061786	-0.842504
C	1.800198	-3.754718	0.700514
H	2.577309	-4.267532	1.253352
C	1.275678	-1.964450	2.407122
C	-1.354925	-1.990256	-2.387389

C	1.075150	-2.597808	1.177569
C	1.320215	-4.042752	-0.544629
H	1.625291	-4.839542	-1.211751
C	-2.689647	-0.227994	-2.267698
C	-2.037878	-1.889225	-3.658240
H	-1.900607	-2.569003	-4.490085
C	-2.860165	-0.801174	-3.585172
H	-3.529303	-0.416868	-4.345196
N	-2.377980	1.045265	0.472456
N	-0.394472	-0.164731	2.245052
C	-1.758963	1.825677	2.721543
C	-2.497145	1.901661	1.535795
C	-3.906683	2.671826	-0.057577
H	-4.632502	3.229432	-0.636574
C	-3.358472	0.899940	-1.779656
C	-0.777417	0.882364	3.041028
C	-3.221097	1.483774	-0.515015
C	-3.461769	2.928441	1.208810
H	-3.752373	3.737052	1.868244
C	0.603413	-0.840281	2.896279
C	0.014494	0.870915	4.252262
H	-0.078392	1.587936	5.058644
C	0.866945	-0.191636	4.162351
H	1.610327	-0.516179	4.879872
O	0.365968	0.650777	-0.392403
C	2.683686	0.098593	-0.627657
C	1.295758	0.227739	-1.197968
H	1.020441	-0.613860	-1.848094
C	2.979546	0.684398	0.608675
C	4.259210	0.569640	1.153283
C	3.679536	-0.614712	-1.304685
H	3.446653	-1.088379	-2.256453
C	4.958553	-0.732934	-0.759709
H	5.723808	-1.294312	-1.289847
C	5.252882	-0.136661	0.469895
H	6.248102	-0.230533	0.896779
H	2.186704	1.203349	1.136879
H	4.477654	1.021630	2.117751
C	1.122104	3.316848	-1.133827
C	1.895431	2.607287	-2.094884
C	1.402386	1.407530	-2.660330
H	0.348178	1.340576	-2.915969
H	2.042856	0.919715	-3.392630
C	3.351533	2.941181	-2.253993
H	3.494288	4.011502	-2.444732
H	3.898617	2.706258	-1.329921
H	3.813032	2.377732	-3.068193
C	1.775861	4.372208	-0.267614
H	2.648408	3.976776	0.266391
H	2.123773	5.223723	-0.866050
H	1.072538	4.758510	0.475895
C	-0.151775	2.885014	-0.858775
H	-0.748025	2.303633	-1.544881
H	-0.683192	3.253467	0.012208
H	2.043797	-2.385605	3.048758
H	-4.065208	1.372210	-2.455917
H	-0.189532	-3.713660	-2.799281
H	-1.956804	2.591587	3.466006
Cl	-2.899333	-2.073284	1.288670

Fe(III)OTf complex

^{2}IVA(OTf-)

Fe	0.562375	0.108229	-0.632612
N	2.446233	0.543857	-0.096696
N	0.219286	2.071594	-0.880949
C	2.364001	2.997661	-0.123812
C	2.998847	1.790984	0.117787
C	4.547602	0.336146	0.852074
H	5.424118	-0.152253	1.258454
C	3.260565	-1.734618	0.315235
C	1.058566	3.122724	-0.568248
C	3.384963	-0.355542	0.362931
C	4.313443	1.667319	0.687380
H	4.954476	2.503813	0.934731
C	-0.987233	2.652913	-1.209914
C	0.373843	4.377182	-0.726454
H	0.823821	5.346249	-0.552345
C	-0.899232	4.085501	-1.111788
H	-1.714549	4.764762	-1.325938
N	-1.138938	-0.289828	-1.593546
N	1.072112	-1.812698	-0.797579
C	-0.931504	-2.719162	-1.886547
C	-1.618389	-1.523264	-1.992484
C	-3.302354	-0.088864	-2.394870
H	-4.232662	0.390846	-2.671213
C	-2.113783	1.965886	-1.631969
C	0.316144	-2.851951	-1.305109
C	-2.169079	0.598251	-1.838132
C	-2.955189	-1.401094	-2.504076

H	-3.542151	-2.227098	-2.884654
C	2.195057	-2.408697	-0.257000
C	0.982459	-4.107397	-1.095265
H	0.593452	-5.066536	-1.411930
C	2.139384	-3.835210	-0.431425
H	2.903945	-4.522956	-0.093483
H	4.078031	-2.327810	0.711791
H	-1.424120	-3.618837	-2.240178
H	-3.002164	2.545791	-1.860110
H	2.912986	3.908607	0.092089
O	-0.023029	0.136225	1.159735
S	-0.917464	-0.868799	1.935215
O	-0.352128	-1.113264	3.262545
O	-1.402532	-1.995864	1.138782
C	-2.406100	0.219568	2.217731
F	-3.282322	-0.423441	2.999248
F	-3.001729	0.505879	1.051699
F	-2.049163	1.362560	2.811707

^{4}IVA(OTf-)

Fe	0.604824	0.111126	-0.610485
N	2.469751	0.576220	-0.012436
N	0.238779	2.071383	-0.881967
C	2.304015	3.028234	0.053190
C	2.966672	1.832831	0.273231
C	4.576931	0.402770	0.923023
H	5.474032	-0.069393	1.302538
C	3.381347	-1.687651	0.279191
C	1.031708	3.130414	-0.482804
C	3.451318	-0.309113	0.386499
C	4.278662	1.730451	0.848690
H	4.879662	2.577237	1.154553
C	-0.939913	2.639632	-1.325275
C	0.344380	4.373607	-0.692040
H	0.758489	5.347028	-0.462286
C	-0.879779	4.069120	-1.207980
H	-1.680172	4.739723	-1.493348
N	-1.061485	-0.310180	-1.642689
N	1.163771	-1.802625	-0.782529
C	-0.849638	-2.752859	-1.825314
C	-1.529066	-1.560997	-1.998681
C	-3.174182	-0.136732	-2.568084
H	-4.084275	0.332736	-2.918938
C	-2.025049	1.945844	-1.832223
C	0.404256	-2.856966	-1.252140
C	-2.067001	0.572339	-1.992898
C	-2.838258	-1.457475	-2.577412
H	-3.416212	-2.300099	-2.934543
C	2.317696	-2.374906	-0.279072
C	1.093478	-4.098593	-1.045675
H	0.703903	-5.067665	-1.329689
C	2.276079	-3.801128	-0.437759
H	3.062241	-4.474149	-0.120549
H	4.224622	-2.266732	0.639910
H	-1.338351	-3.666535	-2.145718
H	-2.892952	2.518615	-2.140916
H	2.812544	3.946543	0.327251
O	-0.093312	0.065107	1.272643
S	-1.043433	-0.878271	2.016699
O	-0.622625	-1.081134	3.405583
O	-1.459538	-2.042786	1.227608
C	-2.553973	0.208193	2.111268
F	-3.523606	-0.413816	2.795201
F	-3.011576	0.482084	0.878355
F	-2.267489	1.364019	2.723270

^{6}IVA(OTf-)

Fe	0.556909	0.095959	-0.431917
N	2.449416	0.847596	0.014133
N	0.030204	2.032392	-0.992105
C	1.941131	3.253429	-0.035669
C	2.763771	2.168290	0.253162
C	4.551537	0.983065	0.951639
H	5.505258	0.653931	1.344354
C	3.630654	-1.281393	0.368322
C	0.679856	3.191507	-0.621922
C	3.531639	0.105748	0.437645
C	4.077161	2.257586	0.837564
H	4.564701	3.182832	1.117619
C	-1.167950	2.419877	-1.554633
C	-0.133237	4.332459	-0.954846
H	0.146424	5.362172	-0.771154
C	-1.275103	3.855404	-1.530645
H	-2.118468	4.415864	-1.913699
N	-0.961871	-0.576561	-1.673140
N	1.450237	-1.765168	-0.664555
C	-0.429893	-2.979533	-1.680458
C	-1.252793	-1.894094	-1.961128
C	-3.005245	-0.703103	-2.735053
H	-3.935022	-0.370637	-3.178960

C	-2.122036	1.554790	-2.082797
C	0.826703	-2.920004	-1.086755
C	-2.021519	0.168962	-2.149248
C	-2.530338	-1.977227	-2.618570
H	-2.992918	-2.898802	-2.948256
C	2.672091	-2.147083	-0.149970
C	1.675589	-4.053836	-0.828085
H	1.421497	-5.079135	-1.065112
C	2.815252	-3.576606	-0.248773
H	3.683051	-4.132286	0.083132
H	4.545995	-1.728703	0.743196
H	-0.796809	-3.961757	-1.960855
H	-3.020458	2.003697	-2.494748
H	2.323224	4.240113	0.207154
O	-0.134155	0.033271	1.391454
S	-1.156996	-0.890094	2.094802
O	-0.831177	-1.044342	3.511539
O	-1.500971	-2.070050	1.298709
C	-2.656878	0.215589	2.030863
F	-3.696921	-0.402641	2.600653
F	-2.967396	0.496358	0.756501
F	-2.420349	1.361558	2.676460

^{2}IA(OTf-)

Fe	0.119570	0.060332	-0.153130
N	-0.674515	-0.218356	-2.002517
N	0.283076	2.037551	-0.546056
O	-2.024262	0.557296	0.570808
C	-0.571662	2.086152	-2.849989
C	-0.849110	0.731916	-2.983357
C	-1.406654	-1.229960	-3.940858
H	-1.714080	-2.023922	-4.609765
C	-4.359717	0.127369	0.568770
C	-0.967589	-2.658612	-1.927039
C	-0.029078	2.683553	-1.719505
C	-0.995222	-1.428622	-2.574079
C	-1.324656	0.107484	-4.190896
H	-1.547452	0.637422	-5.108404
C	-2.970168	-0.147415	0.212605
H	-2.783925	-1.033410	-0.411518
C	0.818809	2.987682	0.289489
C	0.312530	4.079925	-1.615968
H	0.164125	4.807475	-2.403932
C	0.843453	4.267040	-0.375418
H	1.219399	5.180717	0.067562
C	-4.697808	1.249513	1.346306
C	-6.028617	1.489920	1.664650
C	-5.361936	-0.745533	0.115114
H	-5.089968	-1.609407	-0.486863
C	-6.694412	-0.500743	0.438117
H	-7.472451	-1.173550	0.089823
C	-7.025302	0.615654	1.211123
H	-8.064876	0.807714	1.462682
N	0.694397	0.375402	1.748955
N	-0.253973	-1.872616	0.294350
C	0.380704	-1.881389	2.667886
C	0.753844	-0.548874	2.767327
C	1.540973	1.368328	3.649107
H	1.947017	2.144612	4.285187
C	1.241437	2.768471	1.594501
C	-0.065180	-2.497100	1.507758
C	1.163604	1.558373	2.271222
C	1.279528	0.067350	3.957778
H	1.428873	-0.447015	4.898574
C	-0.635362	-2.856766	-0.592020
C	-0.355920	-3.901187	1.386286
H	-0.285856	-4.613832	2.198273
C	-0.696780	-4.126604	0.086140
H	-0.969575	-5.061131	-0.387807
H	-1.245703	-3.533458	-2.506079
H	0.487490	-2.498820	3.553876
H	1.642707	3.618136	2.137714
H	-0.759215	2.721691	-3.709675
H	-6.296893	2.355034	2.264001
H	-3.908210	1.913017	1.684902
O	1.882902	-0.238039	-0.898610
S	3.082556	-1.113184	-0.468992
O	3.682229	-1.753218	-1.642248
O	2.871128	-1.903323	0.745009
C	4.280281	0.230628	0.018668
F	5.454430	-0.319394	0.357965
F	3.812448	0.917772	1.070231
F	4.477574	1.081193	-0.994686

^{4}IA(OTf-)

Fe	-0.112370	0.090734	0.203823
N	0.517556	0.237504	2.111809
N	-0.394699	2.078282	0.150170
O	2.108030	0.555685	-0.407511
C	0.210572	2.650901	2.463519

C	0.554786	1.375843	2.885772
C	1.165896	-0.298373	4.261047
H	1.471946	-0.914156	5.097241
C	4.424466	0.038678	-0.586037
C	0.967920	-2.134193	2.578189
C	-0.243167	2.966145	1.192031
C	0.878946	-0.799270	2.943733
C	0.970814	1.050170	4.223579
H	1.081309	1.771612	5.023206
C	3.026204	-0.250126	-0.266976
H	2.808693	-1.258237	0.122260
C	-0.865585	2.816996	-0.912723
C	-0.629293	4.287552	0.774736
H	-0.603074	5.162725	1.411496
C	-1.019208	4.194222	-0.527902
H	-1.377391	4.976782	-1.184728
C	4.806794	1.301419	-1.071899
C	6.142137	1.551182	-1.363948
C	5.387153	-0.965912	-0.398156
H	5.080799	-1.939423	-0.022370
C	6.724429	-0.711740	-0.693895
H	7.471838	-1.486690	-0.551203
C	7.099520	0.545630	-1.175211
H	8.142625	0.745362	-1.405457
N	-0.505196	-0.014701	-1.757427
N	0.364756	-1.862984	0.208341
C	-0.077963	-2.405260	-2.147033
C	-0.453334	-1.136476	-2.555921
C	-1.178610	0.516873	-3.900267
H	-1.535701	1.123743	-4.722502
C	-1.129336	2.325817	-2.181624
C	0.280417	-2.740559	-0.851549
C	-0.944749	1.008316	-2.569678
C	-0.867985	-0.810042	-3.893158
H	-0.920053	-1.521138	-4.707754
C	0.728570	-2.622208	1.302474
C	0.608983	-4.070291	-0.417633
H	0.613252	-4.940463	-1.061617
C	0.878669	-3.999033	0.917457
H	1.154238	-4.797470	1.594700
H	1.253207	-2.847624	3.344110
H	-0.097987	-3.198816	-2.886373
H	-1.494856	3.024189	-2.927020
H	0.280621	3.456299	3.187121
H	6.444874	2.524942	-1.738016
H	4.046212	2.063609	-1.209810
O	-2.034679	-0.233462	0.890518
S	-3.192990	-1.111297	0.437765
O	-3.928298	-1.684059	1.571658
O	-2.879139	-1.987364	-0.698508
C	-4.326620	0.189060	-0.265655
F	-5.455401	-0.375603	-0.720168
F	-3.733528	0.828762	-1.288999
F	-4.648612	1.096992	0.666269

^{6}IA(OTf-)

Fe	-0.161268	0.242370	0.071483
N	0.168985	0.311557	2.114928
N	-0.062787	2.298984	0.001577
O	2.165187	0.400926	-0.078444
C	0.113737	2.754266	2.413254
C	0.186093	1.444414	2.891566
C	0.312985	-0.297673	4.325457
H	0.375073	-0.937951	5.196448
C	4.412904	-0.369015	-0.112224
C	0.250705	-2.106102	2.566062
C	-0.001599	3.149556	1.078964
C	0.240319	-0.767277	2.962657
C	0.281586	1.066962	4.281410
H	0.313350	1.764416	5.109209
C	2.964210	-0.529546	0.009203
H	2.590095	-1.550800	0.185255
C	-0.163554	3.074434	-1.128533
C	-0.073062	4.512775	0.612039
H	-0.048699	5.386494	1.251278
C	-0.176117	4.466154	-0.749657
H	-0.250535	5.293926	-1.443880
C	4.979404	0.897700	-0.340001
C	6.358114	1.023598	-0.458554
C	5.235662	-1.501296	-0.005283
H	4.787048	-2.476448	0.169943
C	6.617426	-1.370340	-0.125209
H	7.257464	-2.243900	-0.043352
C	7.175724	-0.109315	-0.351918
H	8.253395	-0.006197	-0.446371
N	-0.053498	0.175339	-1.990921
N	0.126696	-1.808749	0.124124
C	0.083505	-2.261771	-2.295377
C	-0.010906	-0.953713	-2.771385

C	-0.191337	0.786242	-4.201385
H	-0.276794	1.425750	-5.070976
C	-0.218878	2.590453	-2.437316
C	0.135887	-2.659322	-0.959162
C	-0.163322	1.253437	-2.836009
C	-0.092968	-0.575114	-4.161463
H	-0.085990	-1.270820	-4.991184
C	0.198704	-2.588932	1.257512
C	0.215046	-4.020041	-0.491830
H	0.230271	-4.892267	-1.133186
C	0.250848	-3.976892	0.874114
H	0.301729	-4.806559	1.568038
H	0.304829	-2.848204	3.356653
H	0.095819	-3.052000	-3.039671
H	-0.302118	3.331007	-3.227015
H	0.142174	3.546088	3.155742
H	6.802906	1.998759	-0.634695
H	4.323931	1.759361	-0.420660
O	-2.146791	0.209597	0.206080
S	-3.116064	-0.963460	-0.025985
O	-3.198076	-1.867996	1.125373
O	-3.010240	-1.538822	-1.370615
C	-4.706619	0.008454	-0.024298
F	-5.736777	-0.823414	-0.230925
F	-4.692787	0.926036	-0.999420
F	-4.880141	0.627486	1.149535

^{4}TS1A$_{endo}$(OTf-)

Fe	0.463051	-0.217301	-0.103042
N	-0.315696	-2.074787	-0.266090
N	0.550549	-0.436580	1.894068
C	-0.220909	-2.765099	2.088941
C	-0.498549	-2.986251	0.746627
C	-1.207972	-4.020071	-1.123767
H	-1.595635	-4.711671	-1.860870
C	-0.704766	-2.135783	-2.691031
C	0.267400	-1.576454	2.614484
C	-0.736037	-2.692134	-1.420352
C	-1.054885	-4.204745	0.217541
H	-1.290614	-5.079410	0.810389
C	0.992298	0.493025	2.806851
C	0.517533	-1.348165	4.012922
H	0.377654	-2.089448	4.789443
C	0.957776	-0.063829	4.132765
H	1.259245	0.465680	5.027620
N	1.235184	1.631907	0.060958
N	0.323898	0.017978	-2.098319
C	1.311122	2.257080	-2.315788
C	1.534804	2.496950	-0.967525
C	2.107141	3.586145	0.919485
H	2.457761	4.294062	1.659769
C	1.447783	1.768616	2.506554
C	0.735117	1.104435	-2.832488
C	1.583863	2.280957	1.224495
C	2.082310	3.717641	-0.437412
H	2.406269	4.556716	-1.040028
C	-0.201185	-0.879633	-2.998281
C	0.459551	0.887429	-4.229358
H	0.693269	1.591956	-5.017546
C	-0.127621	-0.338365	-4.330898
H	-0.473642	-0.850367	-5.219922
O	-1.559374	0.539617	0.018274
C	-3.703420	-0.520139	0.180860
C	-2.443345	0.000656	0.816205
H	-2.041000	-0.680196	1.577046
C	-4.042746	-0.136556	-1.121732
C	-5.217735	-0.608712	-1.708695
C	-4.545334	-1.389999	0.882902
H	-4.275669	-1.699664	1.890778
C	-5.718608	-1.864624	0.296024
H	-6.363784	-2.543881	0.847308
C	-6.059783	-1.471438	-1.001184
H	-6.972182	-1.842822	-1.460355
H	-3.365693	0.515889	-1.663508
H	-5.472835	-0.311095	-2.722761
C	-3.075204	3.000359	0.342216
C	-3.688335	2.221276	1.366867
C	-2.932275	1.267808	2.093587
H	-1.914621	1.514756	2.385620
H	-3.468185	0.724972	2.869915
C	-5.186138	2.172191	1.446915
H	-5.614068	3.181178	1.472882
H	-5.595088	1.675865	0.555145
H	-5.530773	1.621788	2.325200
C	-3.933836	3.726287	-0.670933
H	-4.657355	3.055121	-1.148059
H	-4.506582	4.535788	-0.201017
H	-3.317259	4.173115	-1.456229
C	-1.716600	2.925820	0.178481

H	-1.041081	2.613562	0.958732
H	-1.240590	3.322353	-0.712839
H	-1.078995	-2.739542	-3.511325
H	1.767724	2.394589	3.333000
H	-0.403547	-3.580991	2.780621
H	1.597339	3.033719	-3.017584
O	4.441967	-2.424839	0.095153
S	3.709262	-1.178820	0.362452
O	3.628296	-0.717263	1.756756
O	2.407556	-1.100184	-0.406511
C	4.712272	0.136459	-0.499829
F	4.249592	1.365968	-0.209809
F	4.666055	-0.021327	-1.832111
F	5.996041	0.074673	-0.110073

^{6}TS1A$_{endo}$(OTf-)

Fe	0.425391	-0.200103	0.004020
N	-0.465920	-2.077687	-0.121878
N	0.637859	-0.448148	2.049966
C	-0.270332	-2.728664	2.242970
C	-0.632014	-2.963086	0.911269
C	-1.444112	-3.984847	-0.936101
H	-1.881178	-4.674115	-1.647634
C	-0.932940	-2.093852	-2.537818
C	0.322284	-1.573926	2.768593
C	-0.940106	-2.670277	-1.263464
C	-1.251880	-4.166786	0.403799
H	-1.499050	-5.034836	1.002237
C	1.207043	0.454521	2.911034
C	0.694066	-1.368156	4.149761
H	0.564922	-2.096879	4.940271
C	1.236203	-0.116969	4.237515
H	1.640556	0.374495	5.113679
N	1.278653	1.676274	0.128562
N	0.151918	0.059688	-2.038945
C	1.143035	2.301047	-2.249817
C	1.494446	2.538982	-0.916795
C	2.270025	3.576831	0.938986
H	2.708376	4.267552	1.648532
C	1.697930	1.715136	2.555431
C	0.521597	1.159383	-2.768100
C	1.743056	2.274626	1.273653
C	2.118098	3.739483	-0.410240
H	2.409366	4.588401	-1.016418
C	-0.428369	-0.839305	-2.896686
C	0.152966	0.952333	-4.150900
H	0.329809	1.660107	-4.951273
C	-0.434326	-0.278875	-4.229580
H	-0.832077	-0.774089	-5.106764
O	-1.456136	0.629442	0.298960
C	-3.555848	-0.518320	0.204932
C	-2.412586	0.064653	0.988875
H	-2.082807	-0.588132	1.809349
C	-3.675642	-0.235060	-1.160532
C	-4.722929	-0.783833	-1.901609
C	-4.487637	-1.364865	0.817202
H	-4.386136	-1.601244	1.874526
C	-5.534020	-1.913907	0.076440
H	-6.249774	-2.575046	0.557998
C	-5.656169	-1.621376	-1.285110
H	-6.468910	-2.052808	-1.863436
H	-2.923069	0.390234	-1.628956
H	-4.802378	-0.567202	-2.963891
C	-2.926914	3.163349	0.492284
C	-3.716175	2.302639	1.322083
C	-3.110043	1.336620	2.159148
H	-2.169121	1.584540	2.642744
H	-3.782275	0.777520	2.806661
C	-5.193267	2.222290	1.081890
H	-5.648654	3.219220	1.066437
H	-5.384928	1.770678	0.097228
H	-5.698949	1.614075	1.834330
C	-3.592378	3.974319	-0.597913
H	-4.178701	3.342114	-1.274926
H	-4.278058	4.722003	-0.180318
H	-2.847078	4.506257	-1.195736
C	-1.565054	3.126438	0.589055
H	-1.032458	2.680807	1.414961
H	-0.944226	3.628096	-0.146335
H	-1.357940	-2.690607	-3.339399
H	2.119730	2.313090	3.357889
H	-0.459675	-3.536094	2.944246
H	1.379596	3.088327	-2.959753
O	4.292701	-2.525757	-0.086242
S	3.652921	-1.234389	0.194510
O	3.755749	-0.689164	1.554341
O	2.263603	-1.146935	-0.416371
C	4.577335	-0.013495	-0.869771
F	4.183264	1.244299	-0.609757

F	4.367012	-0.259218	-2.171291
F	5.895415	-0.095909	-0.628429

The six-coordinate mode of IG with replacing PhCHO of acetone

^{2}IG

Fe	-0.000043	0.000093	-0.000063
N	-0.980580	-1.755829	0.024368
N	1.715447	-0.978822	0.365316
O	0.081092	-0.269844	-1.941992
C	0.890339	-3.293117	0.445021
C	-0.447030	-3.013136	0.207074
C	-2.628898	-3.354609	-0.229548
H	-3.614980	-3.762848	-0.412717
C	-3.232459	-0.949877	-0.554289
C	1.891661	-2.336506	0.523841
C	-2.312762	-1.948753	-0.266895
C	-1.473400	-4.013464	0.066896
H	-1.307128	-5.077958	0.174876
C	0.893320	-0.346398	-2.875948
C	2.946539	-0.407076	0.594885
C	3.270547	-2.626167	0.819750
H	3.665339	-3.621985	0.977361
C	3.923438	-1.430296	0.868416
H	4.969218	-1.236374	1.071667
N	0.980411	1.755973	-0.024374
N	-1.715568	0.978941	-0.365523
C	-0.890357	3.293170	-0.445967
C	0.446961	3.013261	-0.207702
C	2.628676	3.354807	0.229670
H	3.614686	3.763110	0.413081
C	3.232134	0.950109	0.555167
C	-1.891721	2.336575	-0.524393
C	2.312514	1.948978	0.267358
C	1.473297	4.013590	-0.067432
H	1.307121	5.078057	-0.175813
C	-2.946783	0.407245	-0.594413
C	-3.270671	2.626277	-0.819958
H	-3.665446	3.622077	-0.977738
C	-3.923676	1.430450	-0.867981
H	-4.969552	1.236562	-1.070772
O	-0.081092	0.269691	1.941847
C	-0.892971	0.346137	2.876139
H	-4.253890	-1.253234	-0.759836
H	-1.172898	4.332294	-0.579816
H	4.253470	1.253492	0.761152
H	1.172921	-4.332276	0.578501
C	2.359325	-0.114037	-2.710198
H	2.848435	0.021382	-3.676919
H	2.538187	0.749447	-2.067851
H	2.793432	-0.992577	-2.216746
C	0.373543	-0.706017	-4.230496
H	0.573371	0.125555	-4.918657
H	0.932572	-1.570797	-4.610284
H	-0.695626	-0.924046	-4.202166
C	-0.372442	0.704312	4.230754
H	-0.572892	-0.127570	4.918370
H	0.696931	0.921316	4.202281
H	-0.930535	1.569332	4.611368
C	-2.359137	0.114615	2.710623
H	-2.848363	-0.019690	3.677430
H	-2.792810	0.992749	2.216141
H	-2.538328	-0.749494	2.069137

^{4}IG

Fe	-0.000028	0.000341	0.000048
N	-0.968489	-1.751354	-0.089558
N	1.673292	-0.952074	0.552046
O	0.340404	-0.283154	-2.203225
C	0.854225	-3.273949	0.558136
C	-0.449048	-3.004316	0.170512
C	-2.582119	-3.365187	-0.454237
H	-3.544904	-3.784820	-0.717155
C	-3.173620	-0.974827	-0.861830
C	1.835612	-2.311133	0.735826
C	-2.274920	-1.961939	-0.487565
C	-1.452143	-4.010497	-0.043932
H	-1.291413	-5.071582	0.099279
C	1.282561	-0.353124	-2.997693
C	2.884952	-0.379879	0.886832
C	3.177042	-2.595268	1.165780
H	3.553809	-3.589326	1.370748
C	3.825677	-1.398601	1.262896
H	4.847989	-1.204007	1.561527
N	0.968370	1.751992	0.089855
N	-1.673228	0.952700	-0.552364
C	-0.854048	3.274537	-0.558812
C	0.449074	3.004938	-0.170648
C	2.582013	3.365829	0.454597
H	3.544763	3.785465	0.717634

C	3.173478	0.975480	0.862356
C	-1.835430	2.311711	-0.736552
C	2.274759	1.962583	0.488142
C	1.452171	4.011096	0.043861
H	1.291558	5.072156	-0.099653
C	-2.884945	0.380502	-0.886859
C	-3.176794	2.595822	-1.166757
H	-3.553449	3.589840	-1.372135
C	-3.825558	1.399159	-1.263394
H	-4.847882	1.204536	-1.561962
O	-0.340624	0.282444	2.203257
C	-1.282652	0.351504	2.998034
H	-4.172153	-1.283791	-1.152343
H	-1.124742	4.310242	-0.734850
H	4.171953	1.284475	1.153039
H	1.125004	-4.309698	0.733799
C	2.701199	-0.110899	-2.568917
H	3.368556	0.021366	-3.423645
H	2.748824	0.760190	-1.911593
H	3.038353	-0.980060	-1.990604
C	1.023798	-0.700854	-4.432661
H	1.331410	0.142185	-5.065265
H	1.648527	-1.555519	-4.721883
H	-0.031284	-0.927213	-4.599578
C	-1.023363	0.695160	4.433870
H	-1.332370	-0.149013	5.064292
H	0.032066	0.919444	4.601397
H	-1.646751	1.550073	4.725273
C	-2.701591	0.112052	2.568653
H	-3.369464	-0.019577	3.423063
H	-3.037166	0.981908	1.990429
H	-2.750357	-0.758709	1.910997

^{6}IG

Fe	0.000024	0.000469	0.000022
N	-1.021093	-1.785600	-0.022729
N	1.733578	-0.997490	0.477139
O	0.210097	-0.302092	-2.148768
C	0.846057	-3.296383	0.529637
C	-0.486522	-3.034874	0.203264
C	-2.657191	-3.370998	-0.333312
H	-3.630367	-3.789977	-0.556977
C	-3.236286	-0.942931	-0.701283
C	1.869008	-2.355276	0.665803
C	-2.343729	-1.964426	-0.367894
C	-1.512451	-4.030944	0.020816
H	-1.363328	-5.096677	0.141900
C	1.103665	-0.389113	-3.000413
C	2.954780	-0.422948	0.754105
C	3.228090	-2.648351	1.046928
H	3.609207	-3.641139	1.251154
C	3.896654	-1.456095	1.104608
H	4.933707	-1.281123	1.362551
N	1.021225	1.786358	0.022610
N	-1.733480	0.998298	-0.477206
C	-0.846167	3.297265	-0.528770
C	0.486474	3.035694	-0.202717
C	2.657360	3.371733	0.333048
H	3.630606	3.790696	0.556438
C	3.236639	0.943619	0.700294
C	-1.869046	2.356128	-0.665358
C	2.343983	1.965142	0.367281
C	1.512423	4.031751	-0.020310
H	1.363183	5.097521	-0.140926
C	-2.954526	0.423666	-0.754765
C	-3.228054	2.649161	-1.046765
H	-3.609262	3.641975	-1.250696
C	-3.896440	1.456833	-1.105112
H	-4.933398	1.281814	-1.363409
O	-0.210287	0.302480	2.148761
C	-1.103964	0.387136	3.000579
H	-4.250069	-1.241900	-0.949227
H	-1.113686	4.336619	-0.692796
H	4.250527	1.242549	0.947852
H	1.113507	-4.335685	0.694112
C	2.543269	-0.141232	-2.667380
H	3.154657	-0.031078	-3.565554
H	2.632776	0.744320	-2.034172
H	2.913160	-0.997466	-2.089732
C	0.747913	-0.764973	-4.404673
H	1.016385	0.063922	-5.072819
H	1.352077	-1.627017	-4.715150
H	-0.316035	-0.991569	-4.496975
C	-0.748580	0.761000	4.405432
H	-1.017362	-0.068707	5.072436
H	0.315340	0.987468	4.498350
H	-1.352798	1.622696	4.716812
C	-2.543261	0.138214	2.666986
H	-3.155354	0.029418	3.564824

H	-2.913031	0.992967	2.087129
H	-2.631739	-0.748700	2.035479

^{2}IG$_{4Ph}$

Fe	-0.001070	-0.000076	0.001577
N	0.571969	-1.934945	-0.058476
N	-1.931880	-0.584213	-0.040902
O	0.254176	-0.224053	2.112745
C	-1.641934	-3.045091	-0.188356
C	-0.244821	-3.048221	-0.127127
C	1.849423	-3.849581	0.089826
H	2.721497	-4.477977	0.194370
C	3.026190	-1.634685	0.164868
C	-2.410760	-1.878297	-0.126956
C	1.863080	-2.408876	0.081672
C	0.556062	-4.242073	-0.067361
H	0.172408	-5.250461	-0.116397
C	-0.431304	-0.149434	3.141333
C	-3.040849	0.237506	0.006338
C	-3.849644	-1.865804	-0.095702
H	-4.478848	-2.743315	-0.125348
C	-4.237381	-0.564369	-0.005469
H	-5.243920	-0.176994	0.053190
N	-0.573026	1.932959	0.071792
N	1.925267	0.588225	0.026942
C	1.639443	3.049033	-0.078902
C	0.243506	3.047671	0.015495
C	-1.861419	3.845813	0.110136
H	-2.741450	4.471402	0.139517
C	-3.038381	1.633632	0.090223
C	2.408362	1.880465	-0.066922
C	-1.870608	2.405316	0.105788
C	-0.560399	4.240420	0.057636
H	-0.174986	5.249253	0.044073
C	3.029627	-0.236095	0.096062
C	3.846111	1.856618	-0.117637
H	4.479180	2.725314	-0.222640
C	4.229100	0.555465	-0.000748
H	5.234171	0.159867	-0.001123
O	-0.217865	0.208269	-2.113177
C	0.497917	0.069599	-3.114766
C	-1.853567	0.314361	3.135703
H	-2.219033	0.505104	4.146650
H	-1.947723	1.213070	2.522190
H	-2.470001	-0.467374	2.676040
C	0.188044	-0.552438	4.442961
H	0.191079	0.312298	5.119280
H	-0.438413	-1.319847	4.915627
H	1.204547	-0.924403	4.301433
C	-0.065905	0.440208	-4.450753
H	-0.103569	-0.458689	-5.080019
H	-1.063187	0.873572	-4.354912
H	0.613808	1.144522	-4.946784
C	1.903434	-0.437536	-3.047392
H	2.266166	-0.740196	-4.031896
H	2.541585	0.372070	-2.671581
H	1.970097	-1.268714	-2.343551
C	4.342923	-2.329606	0.299791
C	4.849429	-3.124107	-0.740685
C	5.101137	-2.181907	1.471839
C	6.085009	-3.760324	-0.609217
H	4.272715	-3.237409	-1.654410
C	6.336169	-2.819177	1.602880
H	4.715718	-1.566972	2.280514
C	6.831040	-3.610490	0.562904
H	6.465824	-4.369619	-1.424627
H	6.909252	-2.698726	2.518410
H	7.792433	-4.106857	0.664698
C	2.340465	4.365794	-0.186165
C	2.195200	5.151432	-1.340352
C	3.149171	4.838014	0.859204
C	2.846322	6.381494	-1.447938
H	1.570757	4.792042	-2.153694
C	3.799334	6.068757	0.751084
H	3.263278	4.238864	1.758260
C	3.650382	6.843141	-0.402479
H	2.726811	6.976359	-2.349596
H	4.419691	6.423040	1.570100
H	4.157638	7.800594	-0.485931
C	-4.352567	2.342623	0.173680
C	-4.727926	2.989230	1.361517
C	-5.226434	2.374016	-0.923592
C	-5.953058	3.652175	1.450770
H	-4.054960	2.966976	2.214436
C	-6.451619	3.037418	-0.833493
H	-4.940177	1.878397	-1.847121
C	-6.817724	3.678169	0.353228
H	-6.231950	4.146279	2.377699
H	-7.117806	3.056629	-1.691962

H	-7.771292	4.194835	0.422283
C	-2.357580	-4.354284	-0.301687
C	-2.395004	-5.249964	0.777703
C	-3.014449	-4.696830	-1.493507
C	-3.071461	-6.466120	0.665063
H	-1.892882	-4.988322	1.705028
C	-3.690969	-5.912939	-1.605115
H	-2.990944	-4.006402	-2.332188
C	-3.721234	-6.800323	-0.526149
H	-3.094058	-7.149947	1.509492
H	-4.193337	-6.166309	-2.534941
H	-4.248378	-7.746684	-0.613028

^{4}IG$_{4Ph}$

Fe	-0.015582	0.018922	0.004336
N	0.905060	1.797683	0.033795
N	-1.789634	0.930874	0.066651
O	0.197906	0.199881	-2.229158
C	-1.053538	3.291851	0.309371
C	0.315674	3.039230	0.190960
C	2.506980	3.452220	-0.126178
H	3.473243	3.915002	-0.262311
C	3.250507	1.070069	-0.317847
C	-2.021024	2.289386	0.197155
C	2.254575	2.038024	-0.164040
C	1.319109	4.066262	0.131322
H	1.133085	5.123979	0.245024
C	-0.456496	0.107385	-3.271160
C	-3.038384	0.341489	-0.050732
C	-3.431833	2.551954	0.132974
H	-3.882695	3.531779	0.190254
C	-4.056884	1.354390	-0.041270
H	-5.115843	1.170924	-0.147726
N	-0.930777	-1.756373	-0.008298
N	1.757987	-0.897803	-0.062768
C	1.037252	-3.255290	0.202639
C	-0.333615	-2.999225	0.114659
C	-2.544770	-3.407294	-0.028879
H	-3.522907	-3.863612	-0.070572
C	-3.298758	-1.027131	-0.148223
C	2.002823	-2.251811	0.088920
C	-2.292780	-1.994293	-0.081618
C	-1.338029	-4.026221	0.098827
H	-1.141073	-5.085941	0.169054
C	2.995596	-0.300520	-0.216931
C	3.418930	-2.494059	0.075863
H	3.885220	-3.459633	0.205633
C	4.029359	-1.294944	-0.138472
H	5.088426	-1.094163	-0.207800
O	-0.132102	-0.153166	2.247768
C	0.657890	-0.207682	3.194997
C	-1.871440	-0.396629	-3.271823
H	-2.240218	-0.588268	-4.281855
H	-1.934947	-1.305207	-2.667775
H	-2.509942	0.358681	-2.798094
C	0.157104	0.515136	-4.576787
H	0.178607	-0.350635	-5.251580
H	-0.478548	1.270881	-5.056279
H	1.166860	0.905384	-4.433257
C	0.173961	-0.641479	4.546328
H	0.270577	0.197980	5.247389
H	-0.865538	-0.973145	4.504790
H	0.816728	-1.445119	4.927273
C	2.112144	0.130318	3.042430
H	2.561115	0.410198	3.998884
H	2.632615	-0.761874	2.669859
H	2.241934	0.927518	2.309250
C	4.663042	1.509153	-0.533026
C	5.373982	2.168460	0.481861
C	5.304229	1.247513	-1.753593
C	6.697259	2.562519	0.277837
H	4.885753	2.365330	1.432167
C	6.627344	1.643599	-1.957146
H	4.759763	0.735990	-2.542539
C	7.326900	2.301914	-0.942313
H	7.236811	3.068753	1.073854
H	7.110535	1.438822	-2.908793
H	8.357350	2.608598	-1.100717
C	1.485530	-4.670346	0.381401
C	1.241612	-5.340984	1.589907
C	2.147319	-5.350053	-0.652863
C	1.654909	-6.663350	1.761244
H	0.729574	-4.820313	2.394512
C	2.558823	-6.672704	-0.480937
H	2.333395	-4.837781	-1.592717
C	2.314151	-7.332326	0.726508
H	1.463154	-7.169793	2.703507
H	3.066782	-7.187996	-1.291785
H	2.634347	-8.362236	0.859862

C	-4.711252	-1.484297	-0.319211
C	-5.112686	-2.074564	-1.527933
C	-5.651964	-1.334672	0.710954
C	-6.429660	-2.502334	-1.703700
H	-4.388360	-2.190460	-2.329740
C	-6.968360	-1.764516	0.534612
H	-5.345866	-0.884058	1.650867
C	-7.360730	-2.348042	-0.673070
H	-6.728202	-2.953140	-2.646420
H	-7.685899	-1.646313	1.342197
H	-8.386010	-2.681444	-0.809623
C	-1.517693	4.699704	0.507847
C	-1.385280	5.654583	-0.512032
C	-2.108921	5.079889	1.722579
C	-1.828635	6.963935	-0.318048
H	-0.937131	5.365094	-1.458377
C	-2.550823	6.389875	1.916085
H	-2.217629	4.344910	2.515361
C	-2.410898	7.335362	0.896832
H	-1.722606	7.692082	-1.117832
H	-3.002415	6.671047	2.863752
H	-2.755119	8.355097	1.047344

^{6}IG$_{4Ph}$

Fe	0.000076	-0.002758	0.002390
N	0.843582	1.876406	0.059101
N	-1.875857	0.836830	0.066576
O	0.188525	0.196270	-2.159352
C	-1.235074	3.237845	0.094371
C	0.162062	3.076980	0.058242
C	2.355774	3.601323	-0.088252
H	3.300474	4.118089	-0.174551
C	3.242043	1.235215	-0.074215
C	-2.168267	2.185772	0.095134
C	2.190301	2.170513	-0.035737
C	1.111644	4.158066	-0.013983
H	0.861564	5.209004	-0.021829
C	-0.513468	0.192537	-3.178254
C	-3.077709	0.157236	0.072331
C	-3.599082	2.357505	0.092522
H	-4.112927	3.308022	0.092434
C	-4.157537	1.111636	0.078595
H	-5.208994	0.864021	0.063568
N	-0.841806	-1.881130	-0.062837
N	1.876145	-0.844051	-0.054324
C	1.234896	-3.246746	-0.043649
C	-0.162978	-3.083437	-0.075904
C	-2.363640	-3.603820	-0.064609
H	-3.313331	-4.118828	-0.054591
C	-3.242062	-1.239031	0.026534
C	2.168737	-2.192992	-0.016449
C	-2.191844	-2.173719	-0.037663
C	-1.118515	-4.162268	-0.100685
H	-0.873156	-5.213769	-0.132719
C	3.077389	-0.163171	-0.051046
C	3.597820	-2.360952	0.059356
H	4.113066	-3.307692	0.131692
C	4.155637	-1.114709	0.032936
H	5.205723	-0.866058	0.082758
O	-0.183014	-0.193985	2.165515
C	0.523023	-0.165434	3.181261
C	-1.960314	-0.195075	-3.143359
H	-2.350940	-0.394578	-4.143616
H	-2.099209	-1.065256	-2.498200
H	-2.526548	0.636381	-2.705032
C	0.086809	0.603007	-4.486481
H	0.048339	-0.247352	-5.180028
H	-0.524151	1.398517	-4.932135
H	1.118093	0.938819	-4.361417
C	-0.066669	-0.563078	4.498269
H	-0.042220	0.300786	5.175590
H	-1.092182	-0.919176	4.382092
H	0.559275	-1.339044	4.957260
C	1.964853	0.238477	3.135031
H	2.348229	0.483196	4.128102
H	2.542047	-0.605777	2.736424
H	2.097968	1.081880	2.454528
C	4.640263	1.762700	-0.127751
C	5.167426	2.504120	0.941813
C	5.450896	1.518651	-1.247908
C	6.474679	2.990749	0.891421
H	4.549193	2.692950	1.815103
C	6.757476	2.006987	-1.298128
H	5.050023	0.947206	-2.080414
C	7.272970	2.744248	-0.228793
H	6.870060	3.558824	1.729315
H	7.370637	1.813346	-2.174306
H	8.290655	3.123395	-0.267600
C	1.770214	-4.643864	-0.025805

C	1.541728	-5.480528	1.078158
C	2.511962	-5.138865	-1.110051
C	2.042496	-6.783347	1.096566
H	0.971674	-5.103260	1.922633
C	3.011844	-6.442125	-1.091143
H	2.691885	-4.498372	-1.969013
C	2.778570	-7.267671	0.011934
H	1.859838	-7.417660	1.959949
H	3.580579	-6.812628	-1.939905
H	3.167707	-8.282209	0.026017
C	-4.642238	-1.767661	0.031671
C	-5.179176	-2.368412	-1.117404
C	-5.441227	-1.661235	1.180178
C	-6.488654	-2.852471	-1.117230
H	-4.567283	-2.449323	-2.011752
C	-6.750174	-2.146870	1.179949
H	-5.030527	-1.198745	2.073462
C	-7.277308	-2.742908	0.031247
H	-6.893343	-3.311235	-2.015538
H	-7.356080	-2.060974	2.078094
H	-8.296655	-3.119526	0.030758
C	-1.771967	4.635758	0.120131
C	-1.701593	5.449492	-1.020631
C	-2.355412	5.149305	1.288134
C	-2.203475	6.752097	-0.993029
H	-1.253948	5.055799	-1.929070
C	-2.856263	6.452365	1.315163
H	-2.411703	4.523746	2.174854
C	-2.781222	7.256795	0.174990
H	-2.145073	7.370466	-1.884859
H	-3.302260	6.839106	2.227691
H	-3.170875	8.271012	0.196505

Reaction B-para

^{2}IIB$_{endo\text{-}p}$

Fe	1.130735	-0.022617	-0.110897
N	0.691124	1.306694	1.323269
N	1.075672	1.421844	-1.499972
O	3.016320	0.408070	0.169881
C	0.640894	3.351043	-0.043975
C	0.560811	2.667851	1.160125
C	0.259288	2.304906	3.363063
H	0.087546	2.387586	4.428437
C	5.325729	0.017195	0.470714
C	0.637649	-0.166217	3.290928
C	0.866945	2.762201	-1.278789
C	0.528360	1.071163	2.672368
C	0.288471	3.294951	2.428012
H	0.135556	4.357496	2.565749
C	3.955048	-0.400273	0.271967
H	3.747076	-1.475548	0.209273
C	1.248210	1.285005	-2.859371
C	0.896423	3.482305	-2.527282
H	0.752991	4.550815	-2.622001
C	1.126236	2.565649	-3.507257
H	1.217607	2.725437	-4.573734
C	5.663162	1.383328	0.554214
C	6.988217	1.750229	0.741644
C	6.324290	-0.968823	0.577259
H	6.054314	-2.019973	0.511287
C	7.650366	-0.593055	0.765672
H	8.425450	-1.348323	0.848570
C	7.979213	0.763666	0.846951
H	9.014821	1.057024	0.993424
N	1.598911	-1.356705	-1.538484
N	1.202025	-1.471488	1.286315
C	1.749428	-3.387317	-0.157750
C	1.799196	-2.708367	-1.367825
C	1.990348	-2.357494	-3.584134
H	2.122805	-2.443735	-4.654811
C	1.531642	0.098393	-3.517310
C	1.464296	-2.808504	1.071112
C	1.698088	-1.128894	-2.894280
C	2.056521	-3.335865	-2.638800
H	2.251867	-4.391960	-2.773342
C	0.962469	-1.347584	2.640065
C	1.397565	-3.531832	2.314140
H	1.567138	-4.596170	2.414307
C	1.083346	-2.627993	3.284256
H	0.945040	-2.797508	4.344255
O	-0.728218	-0.455157	-0.510240
C	-2.837258	-1.391939	-0.015232
C	-1.603600	-0.733169	0.339465
H	-1.408672	-0.531229	1.397684
C	-3.111561	-1.736659	-1.354125
C	-4.277968	-2.424282	-1.657665
C	-3.748898	-1.727482	1.003841
H	-3.537621	-1.441910	2.030728

C	-4.914327	-2.416181	0.691517
H	-5.627954	-2.661519	1.470550
C	-5.175505	-2.764989	-0.636343
H	-6.089595	-3.298364	-0.880361
H	0.477112	-0.209374	4.363188
H	1.931391	-4.456903	-0.176304
H	1.639998	0.133916	-4.596331
H	0.499656	4.426478	-0.019763
H	7.259049	2.799470	0.806640
H	4.877293	2.127015	0.469263
H	-2.398959	-1.461955	-2.124506
H	-4.497122	-2.696996	-2.685463
C	-3.592280	2.309981	-1.362431
C	-3.832650	1.813578	0.011652
C	-2.863214	1.900105	0.947069
H	-1.928854	2.402627	0.734512
H	-2.989539	1.507643	1.951610
C	-2.466557	2.109462	-2.057897
H	-1.640219	1.527997	-1.661961
H	-2.341688	2.516990	-3.056932
C	-5.159941	1.212585	0.292065
C	-5.919503	0.659661	-0.752994
C	-5.689523	1.159919	1.595571
C	-7.151677	0.057733	-0.504125
H	-5.526187	0.674043	-1.764474
C	-6.919087	0.556050	1.845853
H	-5.147904	1.624802	2.413950
C	-7.655551	-0.000677	0.796255
H	-7.718501	-0.367678	-1.327737
H	-7.313240	0.536185	2.858213
H	-8.619986	-0.461879	0.989886
H	-4.417379	2.851654	-1.824653

^{4}IIB$_{endo\text{-}p}$

Fe	1.143451	0.040543	-0.207345
N	0.719165	1.708489	0.811032
N	1.175646	1.084090	-1.910929
O	3.302095	0.506378	0.079209
C	0.675128	3.328132	-1.040687
C	0.576396	2.982325	0.297333
C	0.281151	3.201650	2.518303
H	0.103811	3.559196	3.524205
C	5.549969	-0.037742	0.626277
C	0.608067	0.796511	3.095442
C	0.953239	2.436968	-2.064146
C	0.545845	1.831691	2.175732
C	0.300156	3.914001	1.355530
H	0.140428	4.974654	1.212342
C	4.140456	-0.333092	0.424413
H	3.818865	-1.373624	0.595066
C	1.421282	0.597161	-3.178418
C	1.048050	2.800392	-3.451278
H	0.911919	3.803952	-3.832787
C	1.336859	1.660944	-4.141542
H	1.486779	1.537549	-5.206208
C	6.046777	1.265075	0.430836
C	7.396429	1.522268	0.630907
C	6.414826	-1.073049	1.022062
H	6.022266	-2.076380	1.170604
C	7.766824	-0.809430	1.221410
H	8.439411	-1.604807	1.527037
C	8.254369	0.486281	1.025544
H	9.309592	0.692424	1.180880
N	1.628927	-1.631187	-1.213550
N	1.131349	-1.010295	1.509784
C	1.632375	-3.256056	0.633975
C	1.766610	-2.905275	-0.701134
C	2.097065	-3.117948	-2.917621
H	2.286479	-3.472128	-3.922574
C	1.721906	-0.718072	-3.494800
C	1.336132	-2.369237	1.658798
C	1.814587	-1.750934	-2.575110
C	2.066140	-3.833274	-1.757472
H	2.224468	-4.894509	-1.615540
C	0.879375	-0.526063	2.779415
C	1.209339	-2.738230	3.041018
H	1.324770	-3.745330	3.420360
C	0.928059	-1.597431	3.734680
H	0.766963	-1.478949	4.798374
O	-0.931852	-0.525585	-0.569180
C	-3.033626	-1.515402	-0.085184
C	-1.774585	-0.884632	0.266846
H	-1.552465	-0.767823	1.338032
C	-3.357908	-1.760225	-1.432535
C	-4.549123	-2.401338	-1.746041
C	-3.919352	-1.900630	0.936097
H	-3.670456	-1.689910	1.972924
C	-5.112969	-2.537399	0.615988

H	-5.808127	-2.819218	1.399651
C	-5.423499	-2.790155	-0.722764
H	-6.357828	-3.284541	-0.972640
H	0.437093	1.039918	4.138714
H	1.766387	-4.300323	0.895941
H	1.892779	-0.957588	-4.538966
H	0.523183	4.368978	-1.304855
H	7.788502	2.523792	0.482503
H	5.360233	2.048275	0.125464
H	-2.663381	-1.444398	-2.204013
H	-4.805608	-2.597838	-2.782742
C	-3.665564	2.345377	-1.107205
C	-3.896976	1.767440	0.236434
C	-2.916641	1.782824	1.163034
H	-1.975138	2.279881	0.969039
H	-3.037808	1.327583	2.141575
C	-2.549442	2.175778	-1.825935
H	-1.729464	1.557470	-1.476370
H	-2.431257	2.639659	-2.801376
C	-5.233873	1.176945	0.497599
C	-6.007187	0.684514	-0.566875
C	-5.758388	1.077470	1.799700
C	-7.251236	0.098585	-0.338994
H	-5.615417	0.733194	-1.577898
C	-6.998962	0.487809	2.029033
H	-5.203236	1.494185	2.634893
C	-7.751385	-0.006115	0.959518
H	-7.828691	-0.280247	-1.177810
H	-7.388996	0.430406	3.041605
H	-8.724269	-0.455791	1.137460
H	-4.489249	2.923648	-1.525614

$^{6}IIB_{endo\text{-}p}$

Fe	0.774230	-0.024629	0.228208
N	0.847870	-0.862987	2.106007
N	0.730816	1.844220	1.047050
O	2.953557	0.087477	0.303965
C	0.802490	1.212127	3.428254
C	0.838343	-0.178681	3.299813
C	0.930597	-2.373438	3.834935
H	0.979572	-3.326158	4.346526
C	5.150872	-0.181052	-0.551556
C	0.954188	-3.239165	1.463090
C	0.770061	2.144507	2.391222
C	0.915201	-2.206743	2.403193
C	0.885312	-1.123087	4.387562
H	0.888166	-0.858489	5.437179
C	3.706310	-0.315082	-0.592913
H	3.270705	-0.802375	-1.478878
C	0.733695	3.039666	0.362073
C	0.784377	3.576271	2.556292
H	0.814793	4.087482	3.510022
C	0.755325	4.128140	1.306744
H	0.760073	5.177289	1.040671
C	5.786933	0.436473	0.543114
C	7.170024	0.553640	0.555500
C	5.911407	-0.676352	-1.626042
H	5.411389	-1.151213	-2.466883
C	7.297531	-0.556238	-1.607537
H	7.890185	-0.936601	-2.433573
C	7.923167	0.057827	-0.518118
H	9.005298	0.152060	-0.503493
N	0.800389	0.793140	-1.661448
N	0.942360	-1.917615	-0.616431
C	0.856811	-1.282223	-2.991731
C	0.821410	0.107255	-2.857126
C	0.807625	2.303502	-3.394374
H	0.804112	3.257541	-3.905771
C	0.749142	3.172823	-1.026807
C	0.900315	-2.221154	-1.957069
C	0.786903	2.136846	-1.962593
C	0.824042	1.052087	-3.945704
H	0.839693	0.786620	-4.995026
C	0.956622	-3.108986	0.071594
C	0.912925	-3.653824	-2.121879
H	0.891342	-4.167947	-3.074220
C	0.947275	-4.201648	-0.870102
H	0.960173	-5.250208	-0.601616
O	-1.308039	-0.392853	0.123375
C	-3.233230	-1.736912	0.419497
C	-2.028457	-1.073973	0.869074
H	-1.729633	-1.209765	1.919378
C	-3.674092	-1.598334	-0.910585
C	-4.835827	-2.243655	-1.314607
C	-3.957580	-2.527818	1.331503
H	-3.609129	-2.622925	2.357097
C	-5.116192	-3.173788	0.918310
H	-5.681014	-3.784619	1.615613

C	-5.554204	-3.028107	-0.404210
H	-6.463357	-3.528651	-0.725603
H	0.983261	-4.251196	1.855113
H	0.849878	-1.672799	-4.004671
H	0.740970	4.184598	-1.419708
H	0.816267	1.608250	4.438855
H	7.669616	1.028332	1.394402
H	5.179593	0.811957	1.360473
H	-3.111687	-0.970491	-1.592864
H	-5.191410	-2.126205	-2.333236
C	-3.120246	2.700279	1.660344
C	-4.028670	1.728418	1.012942
C	-4.831692	0.930840	1.745301
H	-4.892368	1.029493	2.824595
H	-5.451608	0.166794	1.287701
C	-2.519683	2.523516	2.842765
H	-2.622307	1.600139	3.407547
H	-1.901754	3.298888	3.282020
C	-4.019726	1.664782	-0.471802
C	-2.827587	1.870306	-1.186585
C	-5.187952	1.367745	-1.195665
C	-2.794500	1.735197	-2.574373
H	-1.913246	2.098393	-0.649126
C	-5.157982	1.240179	-2.582707
H	-6.127323	1.250301	-0.663917
C	-3.957719	1.413505	-3.277308
H	-1.858696	1.877991	-3.105240
H	-6.073910	1.016432	-3.122986
H	-3.933529	1.315818	-4.359307
H	-2.939659	3.623307	1.110160

^{2}TS1B$_{endo\text{-}p}$

Fe	0.982632	0.263568	-0.021892
N	0.850996	1.093881	1.803669
N	1.236478	2.076981	-0.852685
O	2.959715	0.208450	0.228803
C	0.969608	3.463570	1.158289
C	0.840225	2.439966	2.087642
C	0.676803	1.411216	4.085961
H	0.599355	1.159384	5.135784
C	5.129897	-0.731104	0.185902
C	0.707082	-0.928558	3.192199
C	1.180231	3.286959	-0.203291
C	0.758180	0.448382	3.017309
C	0.718286	2.644738	3.509760
H	0.685911	3.614328	3.989986
C	3.695068	-0.758232	-0.021107
H	3.251947	-1.681108	-0.417485
C	1.463493	2.365298	-2.178019
C	1.369762	4.363903	-1.144710
H	1.368629	5.414752	-0.885060
C	1.552633	3.791960	-2.367160
H	1.732666	4.276344	-3.318297
C	5.767284	0.419378	0.690703
C	7.142712	0.413998	0.877680
C	5.881385	-1.878120	-0.127000
H	5.380635	-2.761256	-0.516420
C	7.259875	-1.876659	0.063031
H	7.845092	-2.758898	-0.176647
C	7.887046	-0.732031	0.564334
H	8.963209	-0.730455	0.712576
N	1.189030	-0.578116	-1.838593
N	0.826141	-1.559673	0.822183
C	0.801535	-2.926404	-1.220830
C	1.026235	-1.909876	-2.140770
C	1.392144	-0.893106	-4.117772
H	1.544392	-0.643743	-5.159985
C	1.553000	1.430906	-3.200273
C	0.721385	-2.759434	0.155113
C	1.398744	0.061574	-3.037708
C	1.152813	-2.113684	-3.563206
H	1.071163	-3.074123	-4.055845
C	0.716142	-1.858991	2.162048
C	0.549320	-3.835983	1.098060
H	0.441351	-4.878223	0.826838
C	0.554254	-3.279361	2.341030
H	0.449929	-3.770070	3.300093
O	-0.867236	0.316422	-0.342823
C	-2.670285	-1.144027	0.216729
C	-1.742962	-0.032905	0.572414
H	-1.376768	-0.082450	1.601763
C	-2.689138	-1.648129	-1.090087
C	-3.547757	-2.696069	-1.419711
C	-3.515331	-1.698171	1.186791
H	-3.498221	-1.310182	2.202722
C	-4.379758	-2.738351	0.852517
H	-5.036844	-3.160108	1.607478
C	-4.398134	-3.238358	-0.452556

H	-5.068994	-4.052564	-0.711801
H	0.621439	-1.303041	4.207117
H	0.695240	-3.934876	-1.607168
H	1.725131	1.801389	-4.205786
H	0.943615	4.482467	1.531266
H	7.643104	1.295765	1.265994
H	5.168116	1.293467	0.924983
H	-2.013348	-1.221713	-1.823544
H	-3.552266	-3.092547	-2.431438
C	-3.228211	2.154055	-1.403258
C	-3.653114	1.652659	-0.118279
C	-2.758279	1.542238	0.968269
H	-1.916932	2.225622	1.035438
H	-3.188003	1.283102	1.931651
C	-1.977313	2.582507	-1.680043
H	-1.195011	2.640656	-0.937516
H	-1.707848	2.891161	-2.685259
C	-4.988855	1.062158	-0.000308
C	-5.555761	0.343018	-1.073730
C	-5.711589	1.149559	1.208946
C	-6.786342	-0.287743	-0.930116
H	-4.997724	0.218134	-1.994655
C	-6.957837	0.547318	1.336282
H	-5.311552	1.730693	2.034029
C	-7.493656	-0.181774	0.270380
H	-7.193640	-0.866332	-1.753412
H	-7.513099	0.645119	2.264211
H	-8.461395	-0.663771	0.374748
H	-3.970978	2.160080	-2.195743

^{4}TS1B$_{endo-p}$

Fe	0.924940	0.410242	0.045343
N	0.985195	1.111694	1.933201
N	1.240552	2.264596	-0.656910
O	3.220222	0.223452	0.202560
C	1.190946	3.514830	1.461380
C	1.071233	2.430688	2.319235
C	0.919372	1.257748	4.235848
H	0.864169	0.929509	5.265768
C	5.271143	-0.956160	-0.051949
C	0.745324	-0.999877	3.169305
C	1.280969	3.428940	0.078907
C	0.888730	0.379586	3.095438
C	1.027559	2.528493	3.754765
H	1.082573	3.456254	4.309547
C	3.820329	-0.810791	-0.094116
H	3.244060	-1.695605	-0.414524
C	1.382454	2.639816	-1.973971
C	1.442682	4.559594	-0.796864
H	1.505389	5.588463	-0.466628
C	1.511187	4.070438	-2.067853
H	1.641282	4.616437	-2.993392
C	6.089643	0.118747	0.340519
C	7.468779	-0.043113	0.372030
C	5.845374	-2.187245	-0.409893
H	5.204720	-3.012696	-0.711904
C	7.228283	-2.344962	-0.376325
H	7.676898	-3.294453	-0.651832
C	8.036649	-1.273381	0.014044
H	9.115844	-1.395485	0.040247
N	1.052021	-0.319979	-1.827461
N	0.782001	-1.473506	0.755569
C	0.683128	-2.708584	-1.367586
C	0.888543	-1.631988	-2.218536
C	1.176616	-0.472647	-4.127737
H	1.290598	-0.150561	-5.154760
C	1.383511	1.779457	-3.062869
C	0.631117	-2.627512	0.016406
C	1.220537	0.404257	-2.987247
C	0.966439	-1.732525	-3.652195
H	0.873744	-2.656178	-4.208862
C	0.679509	-1.853134	2.077342
C	0.432995	-3.751873	0.891149
H	0.285183	-4.770543	0.557113
C	0.466944	-3.273562	2.166891
H	0.354236	-3.820079	3.094234
O	-1.063828	0.598790	-0.053470
C	-2.666661	-1.099336	0.485590
C	-1.918480	0.153066	0.832016
H	-1.564302	0.162593	1.874448
C	-2.605001	-1.604143	-0.818196
C	-3.289378	-2.772847	-1.148501
C	-3.418316	-1.775287	1.453329
H	-3.461189	-1.389727	2.470073
C	-4.108952	-2.939767	1.120962
H	-4.692276	-3.458339	1.876538
C	-4.047015	-3.439894	-0.182637
H	-4.583190	-4.348621	-0.441518

H	0.662086	-1.442764	4.156360
H	0.553783	-3.687214	-1.817616
H	1.508443	2.216908	-4.048015
H	1.242580	4.503196	1.906255
H	8.107734	0.781459	0.673501
H	5.622975	1.060162	0.612828
H	-2.001260	-1.079468	-1.550982
H	-3.231302	-3.165016	-2.160470
C	-3.318984	2.234349	-1.215090
C	-3.896257	1.615724	-0.054238
C	-3.155912	1.510599	1.150344
H	-2.452255	2.303714	1.388879
H	-3.692053	1.138362	2.019029
C	-2.079276	2.780212	-1.249375
H	-1.456015	2.905782	-0.375314
H	-1.660479	3.140825	-2.183911
C	-5.164953	0.894325	-0.181008
C	-5.505557	0.228377	-1.377878
C	-6.058107	0.815137	0.908929
C	-6.684059	-0.503144	-1.470962
H	-4.814781	0.232162	-2.213700
C	-7.249317	0.106653	0.802397
H	-5.835577	1.350137	1.826821
C	-7.561551	-0.561537	-0.384942
H	-6.917642	-1.033178	-2.389270
H	-7.936155	0.075441	1.642767
H	-8.487081	-1.124388	-0.464081
H	-3.902038	2.212681	-2.131224

^{6}TS1B$_{endo\text{-}p}$

Fe	0.827298	0.403464	0.083158
N	1.140172	1.036442	2.031241
N	1.242780	2.296813	-0.606159
O	3.082936	0.134881	0.063149
C	1.501635	3.426705	1.567283
C	1.366780	2.332006	2.426828
C	1.275473	1.102599	4.322687
H	1.281120	0.752894	5.347202
C	5.090438	-1.057714	-0.377634
C	0.861198	-1.119121	3.183940
C	1.454629	3.411385	0.169908
C	1.083674	0.262303	3.163329
C	1.450107	2.379024	3.868207
H	1.626104	3.275195	4.449470
C	3.643684	-0.889109	-0.334429
H	3.032452	-1.738104	-0.681290
C	1.276242	2.693555	-1.920453
C	1.613502	4.560831	-0.689034
H	1.794930	5.571042	-0.344691
C	1.504468	4.118223	-1.978192
H	1.581945	4.696824	-2.889953
C	5.947133	-0.022588	0.040543
C	7.322735	-0.206805	-0.009640
C	5.623160	-2.271681	-0.842953
H	4.953092	-3.066033	-1.163663
C	7.002821	-2.451743	-0.890511
H	7.419696	-3.387916	-1.248876
C	7.849109	-1.419826	-0.474323
H	8.925849	-1.559526	-0.511368
N	0.814778	-0.303767	-1.861229
N	0.716551	-1.562286	0.765033
C	0.404814	-2.687839	-1.401276
C	0.567022	-1.596576	-2.259362
C	0.702395	-0.371562	-4.154875
H	0.721444	-0.024601	-5.180189
C	1.113033	1.848116	-3.021753
C	0.462752	-2.672503	-0.004787
C	0.895596	0.468214	-2.995906
C	0.497997	-1.643599	-3.701014
H	0.318790	-2.538671	-4.283077
C	0.680784	-1.958987	2.081505
C	0.268045	-3.814176	0.856516
H	0.049517	-4.817386	0.513982
C	0.402794	-3.374718	2.142587
H	0.319424	-3.949876	3.055855
O	-1.105101	0.646433	0.189837
C	-2.661220	-1.088346	0.757235
C	-1.898578	0.142815	1.097734
H	-1.510795	0.150840	2.126581
C	-2.785290	-1.492251	-0.578142
C	-3.487566	-2.653216	-0.892085
C	-3.238802	-1.861779	1.773301
H	-3.129334	-1.554881	2.811612
C	-3.936877	-3.025182	1.458424
H	-4.376215	-3.625787	2.249657
C	-4.067525	-3.418939	0.123179
H	-4.614542	-4.324530	-0.123626
H	0.821532	-1.588950	4.162163

H	0.201237	-3.646835	-1.867820
H	1.159415	2.313600	-4.001734
H	1.678427	4.390691	2.034946
H	7.991101	0.586767	0.310606
H	5.512082	0.905955	0.396484
H	-2.308349	-0.896636	-1.349106
H	-3.581766	-2.963294	-1.928974
C	-3.176541	2.767865	-0.604121
C	-3.812887	1.809263	0.275337
C	-3.231671	1.489743	1.519302
H	-2.588362	2.232474	1.981223
H	-3.820431	0.920369	2.230428
C	-1.940739	3.278992	-0.417421
H	-1.323868	3.048248	0.440559
H	-1.497405	3.943138	-1.153137
C	-4.947992	1.033280	-0.223748
C	-5.101976	0.797120	-1.609764
C	-5.867610	0.425253	0.659665
C	-6.116645	-0.024270	-2.085062
H	-4.388884	1.213039	-2.312775
C	-6.887224	-0.387156	0.180344
H	-5.804334	0.618626	1.724808
C	-7.011329	-0.620487	-1.192347
H	-6.205690	-0.208896	-3.151312
H	-7.589639	-0.836945	0.875219
H	-7.806420	-1.259857	-1.564754
H	-3.723796	3.055314	-1.496787

^{2}IIIB$_{endo\text{-}p}$

Fe	0.946673	0.387737	0.022210
N	0.893329	0.836133	1.976564
N	1.173783	2.325924	-0.434041
O	2.892832	0.285803	0.178695
C	0.917383	3.288181	1.813369
C	0.857735	2.098019	2.524064
C	0.850680	0.688198	4.281958
H	0.842712	0.231157	5.262908
C	5.055937	-0.615765	-0.114527
C	0.865744	-1.424245	2.937688
C	1.102408	3.385315	0.441689
C	0.893501	-0.040225	3.041493
C	0.813409	2.011775	3.961745
H	0.776589	2.865127	4.626543
C	3.620099	-0.611596	-0.282576
H	3.163114	-1.438306	-0.839783
C	1.421728	2.874044	-1.673962
C	1.294806	4.625636	-0.266498
H	1.280662	5.604437	0.195228
C	1.507421	4.307499	-1.573730
H	1.696695	4.971270	-2.407285
C	5.708041	0.414378	0.593525
C	7.087650	0.378060	0.737056
C	5.797506	-1.674452	-0.672095
H	5.285472	-2.464678	-1.215685
C	7.180240	-1.703654	-0.523765
H	7.758283	-2.516881	-0.950992
C	7.821458	-0.678872	0.179335
H	8.901296	-0.701847	0.295128
N	1.061074	-0.064773	-1.927407
N	0.811927	-1.556366	0.484224
C	0.696850	-2.492803	-1.785569
C	0.895425	-1.312653	-2.488723
C	1.301251	0.070980	-4.221159
H	1.472560	0.516318	-5.192576
C	1.508611	2.163677	-2.862284
C	0.680052	-2.601957	-0.402836
C	1.314627	0.794592	-2.977945
C	1.029786	-1.230388	-3.919977
H	0.938666	-2.072499	-4.593911
C	0.799716	-2.123434	1.742330
C	0.569081	-3.846238	0.312775
H	0.437453	-4.812460	-0.155874
C	0.656264	-3.551199	1.639135
H	0.611614	-4.225825	2.483973
O	-1.033921	0.532325	-0.125491
C	-2.363468	-1.444300	0.498898
C	-1.915308	-0.052733	0.890682
H	-1.303153	-0.105957	1.787970
C	-2.487584	-1.853159	-0.831714
C	-2.967082	-3.127974	-1.134998
C	-2.714212	-2.334388	1.520926
H	-2.603489	-2.030063	2.559672
C	-3.200277	-3.605784	1.219555
H	-3.471539	-4.284886	2.022831
C	-3.330156	-4.005470	-0.112173
H	-3.708493	-4.995567	-0.350052
H	0.853641	-1.998380	3.858249
H	0.577324	-3.405043	-2.360333

H	1.701340	2.723527	-3.771449
H	0.875653	4.212862	2.379634
H	7.599898	1.166038	1.280298
H	5.117197	1.220703	1.016062
H	-2.188568	-1.182555	-1.628640
H	-3.057171	-3.434519	-2.173633
C	-3.091645	1.411155	-1.195417
C	-3.857629	1.119508	-0.131771
C	-3.095335	0.898953	1.156498
H	-2.700145	1.843302	1.556927
H	-3.718411	0.445840	1.931045
C	-1.613280	1.545206	-1.035040
H	-1.338169	2.516737	-0.612059
H	-1.082199	1.416710	-1.976484
C	-5.318024	0.923625	-0.190026
C	-5.929004	0.384916	-1.335700
C	-6.130973	1.274683	0.901217
C	-7.310894	0.221640	-1.394932
H	-5.310663	0.059720	-2.167235
C	-7.514267	1.115853	0.839258
H	-5.682990	1.699168	1.795896
C	-8.109139	0.589528	-0.309007
H	-7.765030	-0.205514	-2.284623
H	-8.128094	1.404077	1.687913
H	-9.186414	0.458375	-0.354295
H	-3.506009	1.582215	-2.184455

^{4}IIIB$_{endo\text{-}p}$

Fe	0.979046	0.450850	0.087106
N	0.895161	0.852489	2.045138
N	1.152918	2.401978	-0.310936
O	3.192604	0.332337	0.245716
C	0.922130	3.310422	1.964074
C	0.862889	2.099793	2.636027
C	0.804204	0.632055	4.342321
H	0.770493	0.144536	5.307934
C	5.304252	-0.648398	-0.234298
C	0.820714	-1.436652	2.941200
C	1.082105	3.443049	0.593544
C	0.859252	-0.057479	3.083110
C	0.794733	1.967356	4.064967
H	0.757303	2.798476	4.757131
C	3.858416	-0.534979	-0.331090
H	3.340409	-1.276043	-0.961703
C	1.360991	2.987849	-1.543805
C	1.235119	4.699217	-0.086421
H	1.213623	5.666276	0.398891
C	1.420059	4.416816	-1.407753
H	1.575932	5.105287	-2.228077
C	6.047363	0.253195	0.551766
C	7.427415	0.121427	0.627152
C	5.955335	-1.676080	-0.939350
H	5.373131	-2.367851	-1.543711
C	7.338931	-1.802997	-0.860426
H	7.847105	-2.594275	-1.402648
C	8.071330	-0.904928	-0.078030
H	9.151394	-1.003531	-0.015899
N	1.089191	0.043930	-1.875284
N	0.843415	-1.504443	0.482056
C	0.768237	-2.393604	-1.811283
C	0.952168	-1.191600	-2.477621
C	1.303012	0.257942	-4.165263
H	1.450967	0.738122	-5.123865
C	1.451348	2.313721	-2.752054
C	0.730021	-2.532897	-0.433235
C	1.302931	0.943298	-2.902548
C	1.075276	-1.061412	-3.903012
H	1.003382	-1.883922	-4.602834
C	0.786665	-2.101665	1.726494
C	0.595356	-3.789304	0.249491
H	0.472791	-4.744283	-0.243692
C	0.640840	-3.523774	1.585215
H	0.564109	-4.217082	2.412295
O	-1.224272	0.582014	-0.101071
C	-2.434315	-1.432022	0.570501
C	-2.082031	0.001069	0.915823
H	-1.486483	0.012808	1.831382
C	-2.517581	-1.882348	-0.750136
C	-2.905854	-3.194692	-1.024194
C	-2.732283	-2.318995	1.611980
H	-2.654800	-1.980462	2.643405
C	-3.125405	-3.628756	1.340227
H	-3.357289	-4.304717	2.158559
C	-3.215284	-4.070023	0.018074
H	-3.522297	-5.089643	-0.197089
H	0.777566	-2.035386	3.844738
H	0.665338	-3.292049	-2.410186
H	1.614031	2.904232	-3.647395

H	0.881918	4.218308	2.556475
H	8.008867	0.811565	1.230782
H	5.523628	1.038712	1.087158
H	-2.264127	-1.207440	-1.559564
H	-2.968676	-3.532234	-2.055356
C	-3.252659	1.439538	-1.208366
C	-4.045612	1.075843	-0.186607
C	-3.332460	0.876714	1.133293
H	-3.012981	1.839349	1.557528
H	-3.965420	0.382941	1.874205
C	-1.786875	1.614220	-0.971545
H	-1.559747	2.582166	-0.504248
H	-1.211022	1.546934	-1.895501
C	-5.491384	0.810168	-0.313455
C	-6.024443	0.273791	-1.498541
C	-6.368720	1.089844	0.748127
C	-7.392166	0.042919	-1.624245
H	-5.354443	0.005827	-2.310292
C	-7.738099	0.862533	0.620442
H	-5.983319	1.512484	1.672220
C	-8.254901	0.338832	-0.566019
H	-7.784489	-0.380967	-2.544443
H	-8.402083	1.095867	1.448017
H	-9.320971	0.154508	-0.662666
H	-3.636612	1.622857	-2.207496

^{6}IIIB$_{endo\text{-}p}$

Fe	0.961027	0.440584	0.063861
N	0.916918	0.888747	2.066928
N	1.172974	2.429872	-0.391370
O	3.127890	0.323967	0.205175
C	1.036781	3.342753	1.893932
C	0.943764	2.151627	2.616406
C	0.843851	0.712269	4.356857
H	0.802109	0.253944	5.336561
C	5.247134	-0.671599	-0.183916
C	0.787010	-1.398001	2.969187
C	1.160357	3.473241	0.509029
C	0.860258	-0.009474	3.109664
C	0.889783	2.044532	4.052451
H	0.894809	2.883897	4.735908
C	3.802246	-0.592805	-0.284365
H	3.289362	-1.400902	-0.828375
C	1.351940	2.974173	-1.643865
C	1.322701	4.715404	-0.202783
H	1.346189	5.694286	0.258524
C	1.447233	4.407444	-1.529158
H	1.589547	5.086371	-2.360087
C	5.978859	0.321890	0.496134
C	7.360719	0.219875	0.577798
C	5.911622	-1.760480	-0.776979
H	5.338182	-2.522387	-1.299657
C	7.296949	-1.856983	-0.691382
H	7.815761	-2.694737	-1.146597
C	8.017661	-0.867727	-0.015009
H	9.099322	-0.942725	0.051978
N	1.072428	-0.022794	-1.943960
N	0.833679	-1.561841	0.510832
C	0.780346	-2.462738	-1.781481
C	0.947899	-1.277043	-2.500571
C	1.247943	0.148606	-4.229527
H	1.371591	0.601914	-5.204710
C	1.397190	2.250156	-2.837394
C	0.727416	-2.596924	-0.392974
C	1.257253	0.867437	-2.980177
C	1.051894	-1.172081	-3.934153
H	0.987613	-2.005643	-4.621799
C	0.756135	-2.114074	1.771649
C	0.576308	-3.838297	0.321704
H	0.456023	-4.807945	-0.143025
C	0.599788	-3.541624	1.655117
H	0.504422	-4.222878	2.490534
O	-1.186984	0.584497	-0.096308
C	-2.427034	-1.426415	0.551079
C	-2.049531	-0.006039	0.918258
H	-1.444939	-0.016101	1.826981
C	-2.501446	-1.863483	-0.774322
C	-2.912968	-3.165128	-1.064566
C	-2.757438	-2.314956	1.581363
H	-2.685647	-1.987141	2.616532
C	-3.174955	-3.613330	1.292953
H	-3.432086	-4.290863	2.102308
C	-3.255757	-4.041706	-0.034042
H	-3.581043	-5.052830	-0.261863
H	0.725394	-1.977840	3.884882
H	0.687432	-3.375566	-2.361390
H	1.535305	2.822358	-3.749546
H	1.045424	4.262242	2.471065

H	7.933741	0.980128	1.099611
H	5.445101	1.152876	0.946092
H	-2.222059	-1.189349	-1.575753
H	-2.967003	-3.493064	-2.099209
C	-3.227845	1.441210	-1.192187
C	-4.009579	1.096339	-0.155588
C	-3.279643	0.891117	1.154181
H	-2.937533	1.848432	1.572360
H	-3.908961	0.407868	1.905078
C	-1.757419	1.605194	-0.985637
H	-1.508399	2.576272	-0.538966
H	-1.198149	1.510011	-1.916809
C	-5.459667	0.846024	-0.259924
C	-6.014698	0.301507	-1.431076
C	-6.318530	1.148383	0.810519
C	-7.386572	0.084838	-1.534948
H	-5.358994	0.015020	-2.248115
C	-7.692004	0.935679	0.704272
H	-5.915238	1.577411	1.724032
C	-8.230966	0.403756	-0.468675
H	-7.796446	-0.345927	-2.444196
H	-8.341861	1.186685	1.537831
H	-9.300303	0.230712	-0.548454
H	-3.624539	1.619385	-2.187194

^{4}TS1B'$_{endo\text{-}p}$

Fe	0.903720	0.420167	0.106197
N	1.113066	1.150609	1.970834
N	1.101431	2.269588	-0.645526
O	3.198168	0.298462	0.051409
C	1.239968	3.549434	1.450636
C	1.206271	2.476805	2.330137
C	1.239029	1.331072	4.268675
H	1.277593	1.017510	5.303910
C	5.249484	-0.813637	-0.412347
C	1.050357	-0.947246	3.251783
C	1.204710	3.444027	0.067122
C	1.133306	0.434757	3.146766
C	1.280606	2.595868	3.762907
H	1.363033	3.532980	4.298212
C	3.797087	-0.703753	-0.343143
H	3.217635	-1.584369	-0.669011
C	1.102363	2.627359	-1.975311
C	1.264239	4.563459	-0.834804
H	1.350050	5.597387	-0.526275
C	1.204547	4.057464	-2.100289
H	1.232436	4.591902	-3.041166
C	6.070271	0.260883	-0.023063
C	7.451259	0.132953	-0.097402
C	5.823471	-2.010158	-0.873381
H	5.181123	-2.835540	-1.171892
C	7.208327	-2.133956	-0.945402
H	7.656784	-3.056630	-1.300651
C	8.018835	-1.062945	-0.557788
H	9.099558	-1.158523	-0.613879
N	0.860516	-0.337399	-1.758604
N	0.877557	-1.457669	0.851262
C	0.604297	-2.728030	-1.235562
C	0.681563	-1.657327	-2.114531
C	0.719575	-0.517098	-4.056116
H	0.705918	-0.205773	-5.092657
C	1.001059	1.752111	-3.047082
C	0.701769	-2.629086	0.144268
C	0.876154	0.375271	-2.937889
C	0.594896	-1.774866	-3.545978
H	0.458643	-2.707614	-4.077909
C	0.925209	-1.820958	2.181035
C	0.644414	-3.746567	1.046961
H	0.507127	-4.775485	0.741205
C	0.787704	-3.247278	2.307542
H	0.795595	-3.783764	3.247561
O	-1.103917	0.573655	0.187272
C	-2.484873	-1.344854	0.560326
C	-1.858413	-0.072934	1.025917
H	-1.496718	-0.122607	2.064363
C	-2.700687	-1.563010	-0.805688
C	-3.263754	-2.760641	-1.239509
C	-2.824745	-2.339192	1.485195
H	-2.636910	-2.175896	2.544329
C	-3.386098	-3.540084	1.049583
H	-3.642581	-4.311729	1.769981
C	-3.610215	-3.749901	-0.313547
H	-4.049558	-4.683411	-0.653873
H	1.075645	-1.376123	4.248163
H	0.454646	-3.715547	-1.658724
H	1.006866	2.177833	-4.045097
H	1.320252	4.544549	1.875746
H	8.091963	0.957398	0.200661

H	5.603980	1.175192	0.330177
H	-2.406533	-0.790786	-1.509460
H	-3.432016	-2.926273	-2.300001
C	-3.322547	2.337090	-0.634148
C	-3.977990	1.479757	0.322963
C	-3.304445	1.126700	1.512697
H	-2.630091	1.866335	1.932498
H	-3.865252	0.586307	2.269381
C	-2.126728	2.938786	-0.433610
H	-1.622769	2.972206	0.522902
H	-1.621480	3.451375	-1.246138
C	-5.255371	0.858435	-0.049992
C	-6.114740	1.491049	-0.976329
C	-5.672058	-0.369537	0.507988
C	-7.332072	0.919277	-1.328972
H	-5.850447	2.457586	-1.392253
C	-6.876406	-0.952229	0.131106
H	-5.033774	-0.897245	1.204976
C	-7.713420	-0.310416	-0.785457
H	-7.985302	1.433275	-2.027658
H	-7.162123	-1.910388	0.554470
H	-8.659237	-0.762027	-1.070315
H	-3.760021	2.399587	-1.625514

^{2}IIB$_{exo\text{-}p}$

Fe	-0.830010	0.643728	0.067395
N	-1.215357	2.124127	-1.238113
N	-1.247937	1.857650	1.607256
O	-2.714397	0.150520	-0.009470
C	-1.758519	3.912483	0.359255
C	-1.589962	3.410400	-0.922295
C	-1.553716	3.341004	-3.173733
H	-1.608795	3.553941	-4.233463
C	-4.594613	-1.269758	-0.175722
C	-0.845676	0.949046	-3.362905
C	-1.599873	3.183788	1.528835
C	-1.192306	2.064553	-2.614831
C	-1.797710	4.175332	-2.125559
H	-2.095907	5.215598	-2.146605
C	-3.174916	-0.997777	-0.133782
H	-2.484833	-1.846266	-0.214501
C	-1.214506	1.553497	2.949357
C	-1.795133	3.724341	2.851154
H	-2.078974	4.747859	3.059390
C	-1.558011	2.713076	3.731065
H	-1.605783	2.732688	4.812111
C	-5.538072	-0.226631	-0.080765
C	-6.892789	-0.524305	-0.121467
C	-5.021917	-2.603930	-0.311535
H	-4.286435	-3.401412	-0.383869
C	-6.381740	-2.894261	-0.351010
H	-6.718224	-3.920935	-0.454764
C	-7.312996	-1.855395	-0.256006
H	-8.374914	-2.082101	-0.286777
N	-0.455716	-0.841608	1.359757
N	-0.406566	-0.569165	-1.477292
C	0.184577	-2.608615	-0.230118
C	-0.049639	-2.121760	1.047334
C	-0.182783	-2.073722	3.295700
H	-0.168005	-2.296623	4.354681
C	-0.890087	0.316617	3.484016
C	0.017108	-1.881042	-1.398565
C	-0.535612	-0.795709	2.736438
C	0.118394	-2.894597	2.250545
H	0.443572	-3.925726	2.268395
C	-0.472593	-0.275001	-2.826548
C	0.232789	-2.410484	-2.718414
H	0.581976	-3.414148	-2.918999
C	-0.074698	-1.418865	-3.601800
H	-0.039751	-1.444024	-4.683348
O	1.027516	1.264718	0.242746
C	3.033378	2.194460	-0.590426
C	1.770395	1.510032	-0.726637
H	1.455458	1.208061	-1.732359
C	3.476680	2.656674	0.664777
C	4.704116	3.298838	0.760137
C	3.820857	2.390595	-1.740956
H	3.467421	2.028508	-2.703112
C	5.048986	3.032544	-1.637092
H	5.664429	3.182951	-2.518303
C	5.488673	3.482731	-0.386696
H	6.449042	3.983845	-0.304736
H	-0.859332	1.042086	-4.443871
H	0.544742	-3.627189	-0.320436
H	-0.906467	0.213231	4.563994
H	-2.048082	4.953942	0.454002
H	-7.627800	0.271293	-0.049062
H	-5.186817	0.794921	0.023251

H	2.854849	2.489295	1.536286
H	5.058309	3.655878	1.722180
C	3.307639	-0.801498	1.703490
C	3.952615	-1.358983	0.493280
C	4.863950	-0.657750	-0.207554
H	5.127648	0.358177	0.065222
H	5.385566	-1.089281	-1.056552
C	3.916443	-0.035869	2.616969
H	4.974271	0.204700	2.550562
H	3.372837	0.359373	3.470770
C	3.548186	-2.732171	0.086284
C	3.254221	-3.700585	1.060712
C	3.450100	-3.098883	-1.265785
C	2.887273	-4.996177	0.696486
H	3.327999	-3.435229	2.111447
C	3.086686	-4.394194	-1.632461
H	3.634261	-2.353211	-2.033674
C	2.801087	-5.349461	-0.652943
H	2.680559	-5.734787	1.466679
H	3.022213	-4.657536	-2.685113
H	2.520341	-6.359490	-0.938011
H	2.261254	-1.059795	1.847482

[4]IIB$_{exo-p}$

Fe	-0.798314	0.728463	0.105313
N	-1.080961	2.289211	-1.119580
N	-1.139335	1.898995	1.685219
O	-2.970973	0.305547	-0.039486
C	-1.493082	4.050140	0.544804
C	-1.360167	3.589270	-0.755503
C	-1.282271	3.608058	-3.004705
H	-1.297011	3.869991	-4.054711
C	-4.853629	-1.105786	-0.370299
C	-0.723501	1.192335	-3.292230
C	-1.388745	3.256192	1.675415
C	-1.022251	2.287998	-2.497722
C	-1.488802	4.414453	-1.925412
H	-1.709015	5.473896	-1.907815
C	-3.432159	-0.818617	-0.265038
H	-2.740119	-1.666235	-0.397599
C	-1.161329	1.522271	3.011937
C	-1.555738	3.738090	3.019292
H	-1.759082	4.768536	3.280251
C	-1.418405	2.663924	3.846710
H	-1.484819	2.631654	4.926447
C	-5.807494	-0.081002	-0.220268
C	-7.158993	-0.380431	-0.327094
C	-5.266462	-2.425004	-0.626942
H	-4.522584	-3.210025	-0.741743
C	-6.622589	-2.719406	-0.732700
H	-6.947333	-3.736155	-0.930595
C	-7.565116	-1.697549	-0.582787
H	-8.623780	-1.926723	-0.665507
N	-0.565024	-0.849078	1.317356
N	-0.439506	-0.452564	-1.482528
C	-0.002441	-2.586153	-0.335737
C	-0.250085	-2.144808	0.953948
C	-0.498026	-2.197724	3.191264
H	-0.558043	-2.475747	4.235569
C	-0.976103	0.234258	3.487483
C	-0.089442	-1.790708	-1.467300
C	-0.704796	-0.866805	2.690520
C	-0.211842	-2.987685	2.116262
H	0.020905	-4.043526	2.093762
C	-0.438776	-0.077686	-2.813546
C	0.144301	-2.253804	-2.805601
H	0.443335	-3.262583	-3.056171
C	-0.077692	-1.195906	-3.639247
H	-0.005654	-1.162617	-4.718719
O	1.332905	1.322505	0.140267
C	3.507004	1.691099	-0.731576
C	2.221558	1.011222	-0.656837
H	2.048308	0.182343	-1.361660
C	3.814611	2.739881	0.153644
C	5.059688	3.352945	0.086685
C	4.445447	1.278105	-1.691494
H	4.196032	0.469258	-2.374205
C	5.688963	1.898719	-1.759488
H	6.418896	1.581471	-2.497752
C	5.995840	2.930744	-0.866973
H	6.968525	3.411969	-0.915351
H	-0.697184	1.342012	-4.366422
H	0.294568	-3.620204	-0.466414
H	-1.041159	0.077443	4.558958
H	-1.703920	5.104708	0.687075
H	-7.901729	0.403245	-0.213106
H	-5.465244	0.929962	-0.023313
H	3.069680	3.046754	0.880806

H	5.308124	4.159080	0.770292
C	2.926839	-1.071017	2.269385
C	3.818748	-1.538648	1.185129
C	4.960155	-0.908438	0.851874
H	5.300840	-0.021688	1.376890
H	5.600080	-1.278778	0.057605
C	2.860165	0.172967	2.758514
H	3.481945	0.981209	2.383830
H	2.155312	0.426463	3.544123
C	3.405905	-2.768968	0.447678
C	2.972270	-3.918735	1.128669
C	3.454322	-2.809339	-0.954549
C	2.612115	-5.071786	0.431164
H	2.944852	-3.921271	2.214519
C	3.093283	-3.960447	-1.655382
H	3.770714	-1.922436	-1.496659
C	2.668200	-5.097857	-0.965558
H	2.302377	-5.957709	0.979406
H	3.143536	-3.967347	-2.741033
H	2.394519	-5.998571	-1.507881
H	2.231762	-1.808957	2.661117

^{6}IIB$_{exo\text{-}p}$

Fe	-0.759098	0.616054	0.085578
N	-0.516642	1.274881	-1.859534
N	-1.248305	2.516851	0.676061
O	-2.850607	0.274793	-0.393541
C	-1.021956	3.658734	-1.494065
C	-0.670877	2.571885	-2.297083
C	-0.097110	1.359206	-4.116603
H	0.154632	1.019275	-5.113060
C	-4.872047	-0.968800	-0.423670
C	0.079758	-0.861204	-2.922952
C	-1.286468	3.636322	-0.122842
C	-0.169674	0.514163	-2.950743
C	-0.406899	2.628345	-3.713108
H	-0.456124	3.525964	-4.316078
C	-3.471538	-0.758263	-0.108306
H	-2.946073	-1.572064	0.414279
C	-1.553026	2.916652	1.955868
C	-1.632820	4.781222	0.681628
H	-1.735130	5.791846	0.307628
C	-1.796334	4.337507	1.964367
H	-2.059324	4.915133	2.841212
C	-5.618079	0.020390	-1.093920
C	-6.956626	-0.208443	-1.381639
C	-5.478362	-2.181113	-0.047959
H	-4.894077	-2.938438	0.469355
C	-6.820542	-2.404248	-0.338900
H	-7.294407	-3.337510	-0.051278
C	-7.555965	-1.418483	-1.004360
H	-8.603930	-1.592170	-1.231755
N	-1.054119	-0.072805	1.994028
N	-0.300105	-1.297116	-0.524788
C	-0.581121	-2.460426	1.629661
C	-0.921461	-1.370383	2.433824
C	-1.461804	-0.144559	4.254458
H	-1.704075	0.199628	5.251755
C	-1.613498	2.075049	3.069148
C	-0.290091	-2.428383	0.265210
C	-1.384201	0.698784	3.089165
C	-1.181442	-1.421120	3.849590
H	-1.147587	-2.319001	4.453401
C	0.009279	-1.699210	-1.809158
C	0.042239	-3.571782	-0.544328
H	0.143239	-4.582726	-0.175056
C	0.231615	-3.122451	-1.820083
H	0.508720	-3.698911	-2.692059
O	1.290890	1.172814	0.443166
C	3.401530	1.841963	-0.401597
C	2.201856	1.022525	-0.382591
H	2.098104	0.254476	-1.163317
C	3.598031	2.846247	0.564178
C	4.757130	3.610620	0.533506
C	4.361735	1.626885	-1.405103
H	4.195489	0.854186	-2.151867
C	5.518911	2.398398	-1.434329
H	6.265415	2.235060	-2.205336
C	5.717073	3.384771	-0.462470
H	6.622688	3.984291	-0.482103
H	0.341105	-1.329006	-3.867161
H	-0.525989	-3.429220	2.116618
H	-1.866049	2.539433	4.017376
H	-1.096596	4.622904	-1.987589
H	-7.540363	0.547547	-1.897767
H	-5.129183	0.948056	-1.373791
H	2.835809	3.003732	1.320723
H	4.920248	4.383907	1.277972

C	2.613060	-1.415471	2.417081
C	3.614731	-1.554618	1.338260
C	4.641055	-0.694316	1.194908
H	4.793315	0.133795	1.879341
H	5.377111	-0.813122	0.406624
C	2.274241	-0.275262	3.030225
H	2.717845	0.680111	2.765771
H	1.515633	-0.260366	3.806046
C	3.472132	-2.705476	0.398926
C	3.187494	-3.999752	0.865316
C	3.663417	-2.528211	-0.980812
C	3.126045	-5.080890	-0.014374
H	3.056805	-4.172850	1.929824
C	3.596510	-3.606313	-1.862445
H	3.868425	-1.531809	-1.362398
C	3.331171	-4.890051	-1.382243
H	2.931611	-6.077632	0.372803
H	3.753344	-3.443281	-2.925572
H	3.287644	-5.733399	-2.065638
H	2.077771	-2.321433	2.690891

^{2}**TS1B$_{exo-p}$**

Fe	-0.622740	0.313122	0.261128
N	-0.290397	1.347289	-1.442808
N	-1.324454	1.970710	1.156177
O	-2.466482	0.014630	-0.411677
C	-0.966793	3.577361	-0.669221
C	-0.481698	2.695891	-1.623287
C	0.321548	1.943989	-3.589087
H	0.683314	1.828639	-4.602663
C	-4.427127	-1.179385	-0.975425
C	0.563896	-0.447667	-2.879532
C	-1.353123	3.233454	0.619173
C	0.200572	0.869885	-2.634851
C	-0.098460	3.076144	-2.960829
H	-0.150287	4.084452	-3.350478
C	-3.077044	-1.063613	-0.460268
H	-2.585390	-1.976951	-0.101245
C	-1.783094	2.093360	2.444756
C	-1.846567	4.175843	1.594798
H	-1.968181	5.236599	1.416848
C	-2.113924	3.468780	2.726671
H	-2.500935	3.827155	3.671799
C	-5.117181	-0.050101	-1.458614
C	-6.409410	-0.193493	-1.944813
C	-5.042194	-2.444230	-0.985479
H	-4.501495	-3.309878	-0.610519
C	-6.337973	-2.580589	-1.473880
H	-6.818355	-3.553927	-1.483736
C	-7.018116	-1.456413	-1.952012
H	-8.029693	-1.562684	-2.333463
N	-1.055385	-0.746521	1.916526
N	0.012380	-1.357283	-0.660184
C	-0.322868	-2.970166	1.168207
C	-0.858489	-2.094390	2.104600
C	-1.708897	-1.341705	4.051143
H	-2.098896	-1.228852	5.054584
C	-1.910573	1.053237	3.354273
C	0.091132	-2.619659	-0.109614
C	-1.574468	-0.268186	3.099441
C	-1.267550	-2.474576	3.433842
H	-1.220064	-3.481776	3.828142
C	0.479362	-1.479043	-1.954298
C	0.631504	-3.545290	-1.071285
H	0.805897	-4.596083	-0.881516
C	0.871292	-2.839654	-2.211681
H	1.278282	-3.194634	-3.149211
O	1.087315	0.781630	0.927176
C	2.601350	2.272794	-0.168624
C	2.100303	0.901366	0.113507
H	2.137967	0.217483	-0.737988
C	2.266687	3.337131	0.679945
C	2.691068	4.627426	0.374710
C	3.362284	2.511892	-1.321202
H	3.602350	1.687443	-1.989395
C	3.781129	3.804758	-1.626950
H	4.360265	3.988876	-2.527110
C	3.449428	4.863619	-0.776618
H	3.778146	5.871547	-1.013153
H	0.945836	-0.689514	-3.866174
H	-0.219582	-4.011267	1.457900
H	-2.298580	1.291606	4.339371
H	-1.047291	4.622007	-0.951381
H	-6.949520	0.670684	-2.319142
H	-4.623139	0.916105	-1.441740
H	1.650882	3.140685	1.551575
H	2.425512	5.452732	1.029221
C	2.472619	-1.516655	2.388996

C	3.348607	-1.171464	1.292455
C	3.697578	0.167846	1.063069
H	3.658368	0.862127	1.894026
H	4.494629	0.383981	0.360770
C	1.898432	-0.626540	3.227276
H	2.115320	0.433932	3.217313
H	1.158242	-0.958021	3.948163
C	3.730975	-2.220223	0.332843
C	3.774662	-3.577651	0.716280
C	4.070778	-1.900663	-0.999156
C	4.148307	-4.566516	-0.188525
H	3.559369	-3.861808	1.740480
C	4.423968	-2.892555	-1.907106
H	4.034384	-0.871824	-1.339306
C	4.467760	-4.229992	-1.505833
H	4.197054	-5.601340	0.137162
H	4.668529	-2.621770	-2.929982
H	4.754216	-5.003199	-2.212660
H	2.154262	-2.549282	2.465437

^{4}TS1B$_{exo\text{-}p}$

Fe	-0.557066	0.449475	0.313870
N	-0.182497	1.477484	-1.379063
N	-1.140534	2.142849	1.216041
O	-2.715269	0.279921	-0.472287
C	-0.619227	3.754217	-0.566287
C	-0.222949	2.844895	-1.534586
C	0.491317	2.043219	-3.512955
H	0.839945	1.909259	-4.528765
C	-4.696776	-0.920890	-1.013657
C	0.478426	-0.371097	-2.858178
C	-1.053979	3.418826	0.707311
C	0.255206	0.970460	-2.581797
C	0.200871	3.204118	-2.862038
H	0.263711	4.218911	-3.232253
C	-3.328020	-0.787435	-0.527953
H	-2.829469	-1.713923	-0.194966
C	-1.617577	2.276041	2.499582
C	-1.489038	4.374066	1.693053
H	-1.519683	5.444812	1.538376
C	-1.842424	3.666130	2.801947
H	-2.221092	4.035943	3.746018
C	-5.413855	0.204563	-1.459534
C	-6.716300	0.052611	-1.917593
C	-5.294460	-2.192049	-1.031965
H	-4.732384	-3.056394	-0.685581
C	-6.600154	-2.339492	-1.492102
H	-7.066642	-3.319704	-1.508181
C	-7.308004	-1.217730	-1.933553
H	-8.326940	-1.331879	-2.292678
N	-1.115809	-0.609073	1.930906
N	-0.132695	-1.274379	-0.651469
C	-0.561877	-2.880175	1.160043
C	-1.014998	-1.972809	2.108233
C	-1.801563	-1.171375	4.061724
H	-2.179332	-1.040232	5.067559
C	-1.837564	1.235891	3.390080
C	-0.148446	-2.548608	-0.122084
C	-1.596742	-0.103496	3.120501
C	-1.440379	-2.329087	3.434993
H	-1.462983	-3.339332	3.823382
C	0.304267	-1.410030	-1.954125
C	0.297193	-3.497440	-1.106343
H	0.390670	-4.561694	-0.936088
C	0.575234	-2.793202	-2.240252
H	0.938000	-3.164394	-3.189638
O	1.345140	0.728891	0.952705
C	2.962921	2.004358	-0.268036
C	2.349491	0.693518	0.121349
H	2.253600	-0.000878	-0.723304
C	2.748753	3.142209	0.519201
C	3.271643	4.371161	0.122892
C	3.702715	2.107729	-1.452185
H	3.850959	1.226220	-2.073149
C	4.222248	3.338330	-1.850345
H	4.787435	3.415829	-2.774738
C	4.009587	4.471702	-1.060834
H	4.415128	5.431233	-1.368910
H	0.827274	-0.626727	-3.853395
H	-0.534512	-3.928334	1.440281
H	-2.217070	1.489050	4.374545
H	-0.594522	4.806866	-0.827111
H	-7.276835	0.916015	-2.263079
H	-4.930999	1.176419	-1.436206
H	2.146244	3.050869	1.417267
H	3.099355	5.254141	0.732110
C	2.409208	-1.649652	2.388850
C	3.322906	-1.458757	1.293998

C	3.805922	-0.164335	1.000768
H	3.899918	0.535188	1.824580
H	4.622242	-0.079326	0.291048
C	1.952238	-0.655505	3.186657
H	2.343574	0.352919	3.175887
H	1.146962	-0.850559	3.887979
C	3.558524	-2.576092	0.367428
C	3.419451	-3.915248	0.792021
C	3.920400	-2.345532	-0.977220
C	3.636234	-4.972155	-0.086107
H	3.183042	-4.135542	1.827466
C	4.115347	-3.403190	-1.858678
H	4.026761	-1.332150	-1.347133
C	3.976833	-4.721440	-1.417632
H	3.546511	-5.993969	0.270598
H	4.380897	-3.198996	-2.891748
H	4.141134	-5.547055	-2.103852
H	1.938972	-2.620920	2.487244

^{6}TS1B$_{exo\text{-}p}$

Fe	-0.538878	0.432219	0.349567
N	-0.226611	1.484310	-1.410933
N	-1.259659	2.131737	1.250649
O	-2.649808	0.218130	-0.465282
C	-0.784973	3.726766	-0.563462
C	-0.329658	2.846772	-1.548508
C	0.460647	2.075063	-3.520776
H	0.832872	1.967156	-4.531528
C	-4.613172	-0.984487	-1.054219
C	0.523250	-0.363372	-2.847416
C	-1.222008	3.398997	0.722701
C	0.251879	0.984945	-2.595483
C	0.104783	3.223422	-2.873366
H	0.131604	4.237589	-3.250356
C	-3.262629	-0.850499	-0.524022
H	-2.777641	-1.771147	-0.160469
C	-1.750927	2.229958	2.527652
C	-1.708400	4.336398	1.708447
H	-1.789726	5.405366	1.558477
C	-2.037031	3.615535	2.820807
H	-2.437744	3.980366	3.757884
C	-5.306386	0.136353	-1.547823
C	-6.592206	-0.017440	-2.049714
C	-5.217911	-2.252612	-1.069075
H	-4.674166	-3.113035	-0.685875
C	-6.507131	-2.401460	-1.572786
H	-6.979229	-3.378989	-1.586210
C	-7.191097	-1.284546	-2.061791
H	-8.197111	-1.399939	-2.455304
N	-1.148637	-0.692753	1.971844
N	-0.094380	-1.332658	-0.665316
C	-0.528367	-2.932084	1.154986
C	-1.012477	-2.052322	2.128248
C	-1.830097	-1.271859	4.087501
H	-2.215085	-1.162860	5.093464
C	-1.929617	1.156602	3.404346
C	-0.098933	-2.601792	-0.133508
C	-1.649634	-0.189002	3.151314
C	-1.437697	-2.420814	3.456669
H	-1.441398	-3.429904	3.849218
C	0.366045	-1.432397	-1.959737
C	0.385742	-3.533477	-1.122833
H	0.499289	-4.598317	-0.967964
C	0.672629	-2.812827	-2.247553
H	1.060642	-3.177078	-3.189835
O	1.310885	0.769646	0.942625
C	2.924514	2.078563	-0.243399
C	2.319656	0.765873	0.116548
H	2.262538	0.057169	-0.716965
C	2.680857	3.209521	0.547361
C	3.200303	4.445130	0.169779
C	3.689944	2.195687	-1.411356
H	3.859587	1.319785	-2.034290
C	4.206082	3.433242	-1.788916
H	4.790750	3.522997	-2.699793
C	3.964086	4.559064	-0.996371
H	4.366994	5.524356	-1.289298
H	0.899709	-0.606813	-3.836496
H	-0.484065	-3.982596	1.427292
H	-2.324433	1.392685	4.387902
H	-0.806710	4.779413	-0.828377
H	-7.134323	0.841965	-2.432483
H	-4.818318	1.105619	-1.526483
H	2.061756	3.108009	1.432866
H	3.005861	5.322828	0.779599
C	2.405766	-1.629126	2.386089
C	3.335655	-1.390803	1.307035
C	3.818515	-0.093208	1.061456

H	3.848735	0.610039	1.886101
H	4.644056	0.027315	0.368592
C	1.929288	-0.678938	3.220989
H	2.290940	0.341273	3.238471
H	1.130754	-0.918268	3.916259
C	3.608026	-2.483927	0.360554
C	3.490476	-3.833992	0.755511
C	3.990645	-2.217946	-0.971772
C	3.750418	-4.867660	-0.138633
H	3.237619	-4.081054	1.780868
C	4.228057	-3.252551	-1.869716
H	4.080774	-1.195022	-1.319383
C	4.112229	-4.582326	-1.457178
H	3.676623	-5.898197	0.195797
H	4.508091	-3.021578	-2.893229
H	4.308978	-5.389891	-2.156187
H	1.954146	-2.612148	2.445067

$^{2}IIIB_{exo-p}$

Fe	-0.653564	0.218500	0.285606
N	-0.850706	1.882642	-0.801551
N	-1.417612	1.132572	1.891809
O	-2.479582	-0.251780	-0.232337
C	-1.603089	3.403844	0.974025
C	-1.224195	3.117427	-0.328575
C	-0.849501	3.414195	-2.529705
H	-0.727526	3.801328	-3.532698
C	-4.241336	-1.645652	-0.962889
C	-0.172122	1.053450	-3.010295
C	-1.719400	2.469828	1.993418
C	-0.620592	2.043702	-2.149464
C	-1.208560	4.081892	-1.400000
H	-1.446124	5.130923	-1.283360
C	-2.865929	-1.371210	-0.612834
H	-2.141243	-2.190994	-0.686937
C	-1.755120	0.570593	3.103439
C	-2.239958	2.758939	3.306582
H	-2.548469	3.740830	3.641479
C	-2.275523	1.580719	3.988620
H	-2.611204	1.396604	5.000906
C	-5.225216	-0.638241	-0.893118
C	-6.536578	-0.938794	-1.232666
C	-4.585161	-2.947345	-1.374025
H	-3.819389	-3.717538	-1.424329
C	-5.902102	-3.240802	-1.712017
H	-6.174382	-4.242492	-2.029014
C	-6.873609	-2.237420	-1.640843
H	-7.902063	-2.466679	-1.904863
N	-0.516767	-1.482764	1.343671
N	0.062025	-0.731359	-1.334599
C	0.620262	-2.900148	-0.316483
C	0.070685	-2.662863	0.934863
C	-0.652415	-3.052640	3.033618
H	-0.898651	-3.477394	3.998175
C	-1.568469	-0.761029	3.446990
C	0.593918	-2.003895	-1.374652
C	-0.964850	-1.707975	2.630797
C	-0.000250	-3.639376	1.989001
H	0.394876	-4.644703	1.920128
C	0.171689	-0.233608	-2.619869
C	1.061070	-2.301317	-2.702712
H	1.533952	-3.231493	-2.989162
C	0.784304	-1.212848	-3.476625
H	0.982673	-1.067573	-4.530679
O	1.152595	0.870944	0.857766
C	2.219705	2.728756	-0.331981
C	2.151425	1.230244	-0.155599
H	1.793617	0.769329	-1.071072
C	1.894356	3.619494	0.695423
C	2.017519	4.995779	0.498271
C	2.662599	3.234382	-1.560256
H	2.897713	2.547394	-2.370477
C	2.791676	4.608848	-1.755608
H	3.135167	4.988311	-2.713804
C	2.470352	5.494055	-0.724315
H	2.567219	6.565334	-0.875312
H	-0.028335	1.318710	-4.052504
H	1.079291	-3.867476	-0.489974
H	-1.871494	-1.072942	4.441012
H	-1.870692	4.430206	1.202181
H	-7.302032	-0.170700	-1.182580
H	-4.938770	0.358583	-0.573675
H	1.507606	3.241112	1.635760
H	1.755771	5.678948	1.301602
C	2.537323	-0.831447	1.851294
C	3.394664	-0.769099	0.816570
C	3.526592	0.602500	0.186305
H	4.052798	1.265806	0.885213

H	4.125552	0.596565	-0.727060
C	1.737147	0.397165	2.130959
H	2.332735	1.234223	2.514307
H	0.897546	0.236020	2.798623
C	4.097797	-1.946392	0.270028
C	4.536278	-2.980758	1.114800
C	4.281911	-2.091647	-1.115745
C	5.129777	-4.126795	0.590094
H	4.431044	-2.870466	2.190401
C	4.869411	-3.241892	-1.640975
H	3.930649	-1.319545	-1.794277
C	5.296207	-4.263799	-0.790628
H	5.474107	-4.909493	1.260246
H	4.996445	-3.338262	-2.715860
H	5.763910	-5.155130	-1.198757
H	2.301514	-1.752205	2.372545

[4]IIIB$_{exo\text{-}p}$

Fe	-0.618711	0.415872	0.329290
N	-0.486830	2.110854	-0.707153
N	-1.156453	1.433287	1.957772
O	-2.754567	0.234895	-0.230403
C	-0.908920	3.723819	1.099309
C	-0.586630	3.392543	-0.206627
C	-0.138807	3.644162	-2.397893
H	0.073293	4.018742	-3.390440
C	-4.649794	-0.984920	-0.986629
C	0.008131	1.211893	-2.941853
C	-1.206508	2.805045	2.094101
C	-0.214992	2.250688	-2.052229
C	-0.354511	4.350186	-1.253044
H	-0.360881	5.423092	-1.114498
C	-3.249266	-0.823880	-0.634374
H	-2.603004	-1.710620	-0.738298
C	-1.556891	0.915722	3.173096
C	-1.642212	3.152388	3.418916
H	-1.762028	4.165737	3.779445
C	-1.872931	1.983538	4.082276
H	-2.213960	1.842640	5.099668
C	-5.549366	0.093314	-0.880408
C	-6.882731	-0.089783	-1.221292
C	-5.098356	-2.239618	-1.435831
H	-4.396329	-3.066476	-1.514599
C	-6.435906	-2.417134	-1.776337
H	-6.787967	-3.383486	-2.123570
C	-7.324447	-1.342899	-1.668379
H	-8.368747	-1.480816	-1.933914
N	-0.739618	-1.298971	1.358588
N	-0.110267	-0.627303	-1.310085
C	0.084641	-2.880469	-0.340370
C	-0.367529	-2.560742	0.929862
C	-1.061431	-2.832671	3.055192
H	-1.335429	-3.216430	4.029377
C	-1.582592	-0.430928	3.503065
C	0.176163	-1.978036	-1.388012
C	-1.166510	-1.454861	2.664207
C	-0.553578	-3.514058	1.986560
H	-0.327292	-4.569261	1.905493
C	0.079313	-0.126997	-2.586436
C	0.551217	-2.325287	-2.729355
H	0.835529	-3.320385	-3.044910
C	0.474055	-1.184201	-3.474799
H	0.680915	-1.053743	-4.529151
O	1.516638	0.782607	0.942325
C	2.882620	2.240212	-0.454409
C	2.493014	0.814784	-0.134103
H	1.981087	0.387922	-0.995764
C	2.785603	3.265450	0.490789
C	3.170226	4.566377	0.160431
C	3.361654	2.536341	-1.736547
H	3.420026	1.746517	-2.482922
C	3.750097	3.834142	-2.066297
H	4.120429	4.050923	-3.064444
C	3.655706	4.853958	-1.116124
H	3.955164	5.866440	-1.371444
H	0.201644	1.468955	-3.977747
H	0.370605	-3.908511	-0.533007
H	-1.902618	-0.698823	4.504369
H	-0.967564	4.777163	1.351618
H	-7.583861	0.735400	-1.142418
H	-5.180912	1.052754	-0.531532
H	2.380775	3.049577	1.473862
H	3.088133	5.356314	0.902145
C	2.440203	-1.222999	1.901350
C	3.249871	-1.390621	0.840016
C	3.700697	-0.101690	0.181758
H	4.381414	0.431556	0.858519
H	4.259663	-0.263247	-0.742529

C	1.983533	0.172167	2.187015
H	2.774559	0.817228	2.592301
H	1.129191	0.212236	2.859922
C	3.587144	-2.715921	0.282070
C	3.726269	-3.837329	1.118818
C	3.693648	-2.904471	-1.106569
C	3.947915	-5.104751	0.584738
H	3.681158	-3.705983	2.196188
C	3.910189	-4.174000	-1.641709
H	3.570368	-2.061474	-1.780150
C	4.036788	-5.279991	-0.799037
H	4.064740	-5.956008	1.249689
H	3.984036	-4.298127	-2.718845
H	4.216057	-6.267283	-1.214896
H	1.995183	-2.051125	2.442270

^{6}IIIB$_{exo-p}$

Fe	-0.639484	0.371710	0.319785
N	-0.598318	2.061362	-0.821507
N	-1.262254	1.431774	1.949989
O	-2.709851	0.075899	-0.236252
C	-1.176033	3.679191	0.944089
C	-0.805910	3.341933	-0.358903
C	-0.290799	3.541654	-2.550407
H	-0.067495	3.903677	-3.545380
C	-4.561816	-1.207368	-0.982577
C	0.039003	1.075897	-2.982973
C	-1.406709	2.800339	2.005096
C	-0.284504	2.154427	-2.157929
C	-0.604984	4.273180	-1.440250
H	-0.689312	5.348409	-1.353380
C	-3.173100	-1.009121	-0.615966
H	-2.503806	-1.880332	-0.683107
C	-1.605423	0.931307	3.185247
C	-1.852667	3.174266	3.323781
H	-2.046245	4.189131	3.646557
C	-1.982879	2.022349	4.048897
H	-2.299270	1.915889	5.078539
C	-5.483680	-0.143342	-0.925262
C	-6.807018	-0.363542	-1.281383
C	-4.978623	-2.485041	-1.398591
H	-4.259621	-3.299804	-1.439759
C	-6.306523	-2.699070	-1.754139
H	-6.634644	-3.682489	-2.075720
C	-7.216798	-1.639087	-1.694864
H	-8.253705	-1.805793	-1.972538
N	-0.697679	-1.368731	1.433964
N	-0.053337	-0.746869	-1.322680
C	0.171278	-2.950193	-0.245469
C	-0.290126	-2.623452	1.030743
C	-0.941416	-2.839295	3.184654
H	-1.182634	-3.204304	4.174805
C	-1.539600	-0.415960	3.548794
C	0.270771	-2.086849	-1.337605
C	-1.100108	-1.475036	2.749303
C	-0.434744	-3.545205	2.127658
H	-0.182892	-4.597199	2.087703
C	0.161744	-0.262086	-2.597836
C	0.701843	-2.457283	-2.661008
H	1.027067	-3.448878	-2.947215
C	0.624806	-1.335599	-3.440249
H	0.869849	-1.235431	-4.489907
O	1.418908	0.822626	0.914399
C	2.709539	2.408552	-0.417380
C	2.409540	0.951915	-0.145760
H	1.938861	0.513984	-1.024208
C	2.466421	3.407571	0.529319
C	2.773873	4.738981	0.241734
C	3.255926	2.760342	-1.658126
H	3.428852	1.989790	-2.406748
C	3.566803	4.088759	-1.944212
H	3.990294	4.348990	-2.910248
C	3.326752	5.083056	-0.992306
H	3.566004	6.119128	-1.214172
H	0.264628	1.306142	-4.019525
H	0.481227	-3.977721	-0.406012
H	-1.824614	-0.660930	4.567249
H	-1.317679	4.735717	1.149418
H	-7.525150	0.449615	-1.240599
H	-5.139827	0.833958	-0.601935
H	2.007352	3.147798	1.477220
H	2.577639	5.508017	0.983925
C	2.488288	-1.098692	1.889260
C	3.309891	-1.207029	0.829272
C	3.671029	0.110111	0.171684
H	4.316392	0.684553	0.849128
H	4.239596	-0.014400	-0.752461
C	1.930667	0.259315	2.170396

H	2.671539	0.969820	2.558923
H	1.077704	0.245425	2.844843
C	3.752987	-2.503044	0.277111
C	3.991245	-3.602709	1.120053
C	3.870323	-2.689107	-1.110848
C	4.321825	-4.848765	0.592060
H	3.937896	-3.469228	2.196804
C	4.194754	-3.937982	-1.639656
H	3.668706	-1.864441	-1.788213
C	4.421386	-5.023196	-0.791016
H	4.514329	-5.682856	1.261099
H	4.273447	-4.061961	-2.716397
H	4.684697	-5.993536	-1.202031
H	2.102654	-1.956705	2.428771

^{4}TS1B'$_{exo\text{-}p}$

Fe	-0.616190	0.315953	0.294172
N	-0.292061	1.315122	-1.423931
N	-1.216263	2.020503	1.171381
O	-2.791732	0.125826	-0.457026
C	-0.851805	3.590126	-0.684033
C	-0.412789	2.671795	-1.624851
C	0.360652	1.850025	-3.572586
H	0.721651	1.703567	-4.582331
C	-4.809877	-1.052039	-0.904966
C	0.421591	-0.546714	-2.860939
C	-1.219599	3.279299	0.616316
C	0.174886	0.795148	-2.610319
C	-0.005901	3.011648	-2.962614
H	-0.001853	4.015773	-3.365694
C	-3.452512	-0.911391	-0.389146
H	-3.010132	-1.804246	0.085291
C	-1.615185	2.178548	2.478482
C	-1.635049	4.250074	1.595888
H	-1.724640	5.312116	1.407727
C	-1.876126	3.569235	2.750749
H	-2.206660	3.956676	3.705785
C	-5.455458	0.029621	-1.531679
C	-6.746386	-0.128966	-2.019096
C	-5.467458	-2.286260	-0.772289
H	-4.960788	-3.117040	-0.286373
C	-6.761481	-2.440521	-1.262230
H	-7.274020	-3.392353	-1.162161
C	-7.397913	-1.362456	-1.884288
H	-8.407623	-1.482013	-2.266958
N	-1.127723	-0.717040	1.946194
N	-0.216028	-1.424621	-0.652172
C	-0.676726	-3.009231	1.172374
C	-1.051304	-2.081239	2.134672
C	-1.713655	-1.238438	4.116588
H	-2.035464	-1.086737	5.138862
C	-1.756925	1.159087	3.408065
C	-0.297240	-2.696697	-0.126123
C	-1.535265	-0.186728	3.151771
C	-1.419185	-2.412422	3.485166
H	-1.448996	-3.417670	3.885808
C	0.216221	-1.576905	-1.952964
C	0.077667	-3.665704	-1.121832
H	0.085891	-4.736895	-0.964059
C	0.406570	-2.971388	-2.249032
H	0.740030	-3.356437	-3.203925
O	1.266612	0.603726	0.922886
C	2.711884	2.119352	-0.242576
C	2.305159	0.725027	0.140069
H	2.367221	0.015046	-0.697975
C	2.337767	3.203710	0.559578
C	2.674510	4.500580	0.178725
C	3.428003	2.342305	-1.424352
H	3.707374	1.498413	-2.051757
C	3.760216	3.641403	-1.807180
H	4.305926	3.811330	-2.730957
C	3.386242	4.722344	-1.004374
H	3.647259	5.734369	-1.300772
H	0.778732	-0.814855	-3.849802
H	-0.685759	-4.057109	1.454936
H	-2.078820	1.432402	4.407571
H	-0.892112	4.632398	-0.981644
H	-7.251731	0.700725	-2.504163
H	-4.927209	0.973511	-1.622439
H	1.758085	3.016633	1.457977
H	2.376644	5.340683	0.800069
C	2.667995	-1.656485	2.332214
C	3.591801	-1.202707	1.327705
C	3.822088	0.176110	1.120714
H	3.730535	0.850622	1.966782
H	4.624725	0.447676	0.442077
C	1.939199	-0.837093	3.126290
H	2.052038	0.238529	3.134129

H	1.193090	-1.248364	3.799011
C	4.141958	-2.172096	0.372104
C	3.382333	-3.286964	-0.038393
C	5.426372	-1.991742	-0.184419
C	3.882600	-4.177155	-0.983003
H	2.377053	-3.424499	0.342436
C	5.933125	-2.898587	-1.108946
H	6.045072	-1.162644	0.144405
C	5.160603	-3.990945	-1.515650
H	3.272414	-5.014892	-1.307232
H	6.932648	-2.758676	-1.509438
H	5.555548	-4.694674	-2.242662
H	2.510677	-2.727690	2.414277

^{2}VB'$_{endo-p}$

Fe	1.997096	-0.127482	-0.253261
N	1.455502	-1.310746	-1.762160
N	2.203576	-1.741778	0.895262
C	1.551889	-3.512110	-0.674256
C	1.308292	-2.688352	-1.759351
C	0.575461	-2.032283	-3.781782
H	0.190248	-1.972728	-4.791350
C	1.040286	0.400444	-3.478562
C	1.936018	-3.060204	0.575916
C	1.006240	-0.902084	-3.006322
C	0.785529	-3.140750	-3.018044
H	0.595799	-4.176747	-3.266304
C	2.486076	-1.747396	2.248365
C	2.069332	-3.897632	1.735987
H	1.928079	-4.970516	1.737510
C	2.389437	-3.081057	2.777651
H	2.574502	-3.346069	3.810467
N	2.958687	0.951597	1.116985
N	2.168221	1.393349	-1.536866
C	3.286256	3.054077	-0.111830
C	3.384915	2.264472	1.021109
C	3.718256	1.702099	3.171966
H	3.972323	1.672482	4.223499
C	2.892074	-0.645514	2.981216
C	2.693232	2.651561	-1.296726
C	3.150091	0.603799	2.439691
C	3.880166	2.725785	2.287885
H	4.286301	3.712998	2.465213
C	1.625249	1.462967	-2.806336
C	2.488967	3.505560	-2.432629
H	2.822316	4.533168	-2.495096
C	1.811693	2.774488	-3.362270
H	1.482567	3.076034	-4.348165
O	0.254371	0.386907	0.403578
C	-1.765994	1.598961	0.438087
C	-0.678804	0.928713	-0.229159
H	-0.661170	0.914698	-1.324984
C	-1.795027	1.698106	1.846090
C	-2.869717	2.315595	2.466899
C	-2.814790	2.137968	-0.329601
H	-2.790932	2.049937	-1.411978
C	-3.888002	2.757883	0.300744
H	-4.709336	3.152879	-0.286888
C	-3.918005	2.838951	1.694551
H	-4.762522	3.312332	2.186578
H	0.654265	0.585365	-4.475720
H	3.658481	4.071481	-0.053153
H	3.087052	-0.786518	4.039234
H	1.385898	-4.576918	-0.800034
H	-0.971921	1.284766	2.419745
H	-2.903032	2.393177	3.549197
C	-2.107116	-1.722360	1.271210
C	-2.781438	-1.498209	-0.028761
C	-2.131002	-1.803757	-1.172525
H	-1.115964	-2.178365	-1.145838
H	-2.594718	-1.729446	-2.150472
C	-1.344526	-2.781019	1.564304
H	-1.207821	-3.597723	0.861291
H	-0.836751	-2.868608	2.519907
C	-4.143299	-0.902462	-0.010627
C	-4.714323	-0.324093	-1.161164
C	-4.898498	-0.880607	1.174482
C	-5.983669	0.245242	-1.129226
H	-4.153311	-0.302051	-2.090368
C	-6.164848	-0.297125	1.212775
H	-4.502589	-1.341639	2.073594
C	-6.713966	0.269383	0.062095
H	-6.401327	0.679528	-2.033548
H	-6.727553	-0.297731	2.142084
H	-7.702466	0.718849	0.089786
H	-2.256409	-0.954558	2.028286

^{4}VB'$_{endo-p}$

Fe	2.036553	-0.147630	-0.292092
N	1.462555	-0.447847	-2.182218
N	1.928080	-2.114322	0.028800
C	1.120198	-2.884723	-2.165055
C	1.084325	-1.643017	-2.773947
C	0.713670	-0.071434	-4.340635
H	0.464561	0.476905	-5.239790
C	1.564157	1.864387	-3.022058
C	1.504021	-3.095561	-0.852542
C	1.251581	0.521537	-3.151272
C	0.623203	-1.413221	-4.111453
H	0.279034	-2.189830	-4.781915
C	2.159285	-2.773880	1.224791
C	1.474717	-4.373078	-0.201186
H	1.184363	-5.298435	-0.681207
C	1.869050	-4.171606	1.088767
H	1.972609	-4.898959	1.883277
N	2.967256	0.065783	1.460228
N	2.544974	1.727490	-0.769917
C	3.626047	2.432312	1.324341
C	3.519024	1.221217	1.984775
C	3.649056	-0.292481	3.643053
H	3.826592	-0.825054	4.568205
C	2.647829	-2.191813	2.381140
C	3.160647	2.664397	0.042242
C	3.045799	-0.870363	2.476319
C	3.952112	0.999993	3.334977
H	4.425569	1.748690	3.956492
C	2.197333	2.414083	-1.920901
C	3.215214	3.937741	-0.616596
H	3.659969	4.828175	-0.191961
C	2.608115	3.785791	-1.827503
H	2.456635	4.524842	-2.603376
O	0.217002	0.460858	0.476103
C	-1.808634	1.682999	0.539490
C	-0.726624	0.999434	-0.133848
H	-0.734327	0.981727	-1.232176
C	-1.834875	1.772222	1.946461
C	-2.902510	2.397686	2.574109
C	-2.854552	2.233751	-0.221920
H	-2.832379	2.152801	-1.305090
C	-3.923395	2.856756	0.413423
H	-4.742700	3.262198	-0.170718
C	-3.947695	2.934583	1.808037
H	-4.786011	3.414250	2.304863
H	1.346989	2.517802	-3.860216
H	4.086714	3.259348	1.853723
H	2.773834	-2.824285	3.253203
H	0.801276	-3.743973	-2.744920
H	-1.018293	1.339270	2.514583
H	-2.932723	2.468703	3.657069
C	-2.232105	-1.559193	1.462789
C	-2.823279	-1.411495	0.113913
C	-2.074956	-1.649920	-0.985229
H	-1.029563	-1.916350	-0.897449
H	-2.488556	-1.622398	-1.988146
C	-1.288193	-2.444761	1.802521
H	-0.910819	-3.185249	1.103245
H	-0.869200	-2.463003	2.804215
C	-4.230871	-0.943233	0.030759
C	-4.717747	-0.278958	-1.110525
C	-5.113899	-1.133296	1.107666
C	-6.032718	0.173327	-1.174780
H	-4.052425	-0.094327	-1.948793
C	-6.429841	-0.675762	1.047900
H	-4.775717	-1.663576	1.992777
C	-6.894864	-0.018498	-0.092076
H	-6.383889	0.684214	-2.067290
H	-7.094613	-0.840771	1.891114
H	-7.919696	0.338535	-0.138676
H	-2.605430	-0.872978	2.220573

^{6}VB’$_{endo-p}$

Fe	1.904912	-0.085347	-0.213958
N	1.565689	-0.021150	-2.253241
N	1.870411	-2.147007	-0.350352
C	1.095928	-2.400976	-2.674387
C	1.153580	-1.066104	-3.061276
C	1.024242	0.783394	-4.347740
H	0.889547	1.491830	-5.154916
C	1.908931	2.388943	-2.628645
C	1.434655	-2.904387	-1.421711
C	1.510519	1.118314	-3.036469
C	0.809684	-0.565794	-4.364534
H	0.461979	-1.177262	-5.187158
C	2.090198	-3.035137	0.686420
C	1.367022	-4.290226	-1.043237
H	1.057288	-5.093840	-1.698644

C	1.769089	-4.370412	0.259913
H	1.855097	-5.253161	0.880179
N	2.908063	-0.287945	1.574855
N	2.674402	1.828533	-0.358216
C	3.594981	2.050894	1.914392
C	3.443857	0.732682	2.335259
C	3.541664	-1.107511	3.636352
H	3.705409	-1.821472	4.433108
C	2.575135	-2.695777	1.946073
C	3.243935	2.558612	0.667271
C	2.970092	-1.425757	2.354821
C	3.836904	0.225417	3.623228
H	4.289525	0.818301	4.407420
C	2.467826	2.715646	-1.396586
C	3.406209	3.928823	0.257394
H	3.837893	4.708537	0.871413
C	2.923302	4.026894	-1.015297
H	2.882853	4.902551	-1.650104
O	0.111134	0.516103	0.455268
C	-1.945269	1.682518	0.478797
C	-0.840239	1.032530	-0.177016
H	-0.817324	1.018296	-1.273702
C	-2.003947	1.754642	1.887412
C	-3.097578	2.349884	2.497565
C	-2.986550	2.221427	-0.300226
H	-2.938575	2.154756	-1.383533
C	-4.079015	2.817551	0.319043
H	-4.892082	3.217146	-0.277555
C	-4.135509	2.876493	1.713907
H	-4.993535	3.334187	2.197491
H	1.809871	3.191608	-3.352637
H	4.034986	2.746097	2.622381
H	2.691561	-3.503169	2.662119
H	0.760136	-3.116365	-3.418344
H	-1.189625	1.336364	2.469466
H	-3.153531	2.407384	3.580163
C	-2.136367	-1.641929	1.384327
C	-2.774749	-1.452185	0.063000
C	-2.049506	-1.626579	-1.064701
H	-0.997479	-1.876820	-1.013313
H	-2.488993	-1.572138	-2.055309
C	-1.192199	-2.549726	1.659543
H	-0.856775	-3.274203	0.922854
H	-0.733423	-2.604721	2.642271
C	-4.194658	-1.017136	0.039002
C	-4.746884	-0.366946	-1.081114
C	-5.027273	-1.228725	1.151896
C	-6.074958	0.049345	-1.090708
H	-4.124734	-0.166352	-1.948070
C	-6.355508	-0.804518	1.147704
H	-4.640821	-1.751191	2.021505
C	-6.885301	-0.162140	0.027630
H	-6.476818	0.547639	-1.968776
H	-6.979683	-0.985666	2.018134
H	-7.920492	0.166836	0.023438
H	-2.472465	-0.974252	2.175730

^{2}TS2B'$_{endo-p}$

Fe	1.786681	0.200614	0.361939
N	3.001782	-0.294384	-1.142381
N	2.150785	-1.587420	1.200284
C	3.569900	-2.605501	-0.521682
C	3.611676	-1.515092	-1.374328
C	3.999896	-0.311657	-3.233742
H	4.315098	0.054458	-4.202235
C	2.789869	1.743905	-2.506786
C	2.870193	-2.640178	0.673130
C	3.224226	0.445375	-2.289967
C	4.251075	-1.521620	-2.660784
H	4.811298	-2.355559	-3.063167
C	1.579158	-2.077331	2.354578
C	2.754026	-3.802530	1.514287
H	3.242788	-4.749497	1.325069
C	1.939892	-3.457394	2.550675
H	1.626735	-4.061011	3.392743
N	0.992191	0.826956	2.078544
N	1.734743	2.084109	-0.318221
C	0.623925	3.203301	1.562287
C	0.526436	2.093326	2.383953
C	-0.135740	0.802737	4.102184
H	-0.549741	0.405353	5.019872
C	0.816758	-1.334630	3.241679
C	1.170857	3.189039	0.291177
C	0.573156	0.023696	3.124405
C	-0.152521	2.087430	3.649366
H	-0.591219	2.961369	4.112729
C	2.119572	2.516565	-1.572834
C	1.201413	4.321495	-0.593630

H	0.828898	5.305923	-0.342263
C	1.778731	3.902236	-1.754012
H	1.985317	4.473345	-2.649876
O	0.193160	-0.391339	-0.427037
C	-1.659313	1.139062	-0.471128
C	-0.601362	0.356563	-1.138416
H	-0.166869	0.826547	-2.029056
C	-2.130877	0.762584	0.794674
C	-3.099111	1.532087	1.432027
C	-2.166495	2.290557	-1.091391
H	-1.786442	2.590027	-2.065676
C	-3.137172	3.058688	-0.450514
H	-3.522587	3.954510	-0.928537
C	-3.605968	2.678031	0.810412
H	-4.362438	3.276532	1.309910
H	3.038008	2.203842	-3.457786
H	0.207475	4.137423	1.923819
H	0.426595	-1.841291	4.118443
H	4.095403	-3.504529	-0.826932
H	-1.717653	-0.121484	1.268676
H	-3.456339	1.243055	2.415829
C	-1.530509	-2.806529	-0.610452
C	-2.247283	-1.857572	-1.442584
C	-1.545647	-1.038149	-2.337769
H	-0.588280	-1.382141	-2.712234
H	-2.103314	-0.427568	-3.039965
C	-0.213871	-3.089476	-0.699371
H	0.430780	-2.713606	-1.484308
H	0.262308	-3.727426	0.038571
C	-3.685277	-1.671540	-1.208204
C	-4.338820	-0.468109	-1.554521
C	-4.462527	-2.702999	-0.633175
C	-5.693570	-0.291122	-1.301845
H	-3.781260	0.353530	-1.984880
C	-5.823759	-2.533321	-0.407903
H	-4.011224	-3.660876	-0.398603
C	-6.443031	-1.322253	-0.729204
H	-6.166155	0.653350	-1.553560
H	-6.403740	-3.346675	0.017403
H	-7.504101	-1.186456	-0.541533
H	-2.082269	-3.249259	0.212299

[4]TS2B'$_{endo-p}$

Fe	1.836507	0.233016	0.224687
N	2.532144	0.265255	-1.659819
N	2.652380	-1.580002	0.474395
C	3.356446	-2.045390	-1.837862
C	3.069479	-0.797170	-2.363834
C	2.896967	0.871159	-3.863432
H	2.922383	1.498773	-4.744731
C	1.900429	2.551602	-2.312106
C	3.171837	-2.399529	-0.511292
C	2.417458	1.293719	-2.577153
C	3.298738	-0.424443	-3.731721
H	3.723113	-1.078537	-4.482301
C	2.659680	-2.340902	1.628820
C	3.501732	-3.686308	0.033349
H	3.931526	-4.500875	-0.535045
C	3.187142	-3.649107	1.360047
H	3.306879	-4.426624	2.103393
N	1.555849	0.353069	2.208204
N	1.415748	2.191318	0.072441
C	0.644363	2.632343	2.365026
C	0.987230	1.403782	2.902675
C	1.243419	-0.231753	4.425429
H	1.267673	-0.841703	5.319045
C	2.219765	-1.918972	2.872092
C	0.853018	2.995623	1.046394
C	1.708147	-0.660099	3.134894
C	0.793801	1.045906	4.280583
H	0.373448	1.703086	5.030659
C	1.435431	2.963569	-1.074715
C	0.519254	4.281076	0.501616
H	0.068608	5.086692	1.066068
C	0.883150	4.262957	-0.811292
H	0.795032	5.051658	-1.547091
O	0.034326	-0.380959	-0.222551
C	-1.810287	1.132484	-0.417768
C	-0.775754	0.253358	-1.019037
H	-0.337755	0.651885	-1.945175
C	-2.222095	0.936084	0.907067
C	-3.168324	1.785536	1.474387
C	-2.347705	2.189316	-1.164793
H	-2.014347	2.350425	-2.187850
C	-3.294065	3.039897	-0.594277
H	-3.703251	3.862506	-1.173618
C	-3.707641	2.836209	0.725227
H	-4.445996	3.497371	1.169858

H	1.862638	3.264312	-3.129133
H	0.191712	3.364558	3.024781
H	2.284304	-2.621179	3.696474
H	3.776565	-2.790347	-2.505349
H	-1.780400	0.125604	1.477714
H	-3.487366	1.630801	2.501136
C	-1.556837	-2.876237	-0.271436
C	-2.362021	-1.988196	-1.083918
C	-1.739609	-1.165062	-2.040534
H	-0.820424	-1.526225	-2.489335
H	-2.367266	-0.608731	-2.729189
C	-0.227630	-3.069629	-0.418791
H	0.350222	-2.676787	-1.245597
H	0.330036	-3.655786	0.305008
C	-3.790452	-1.854826	-0.772597
C	-4.511518	-0.686531	-1.105037
C	-4.491193	-2.906575	-0.138947
C	-5.858463	-0.563351	-0.786191
H	-4.012193	0.149498	-1.577470
C	-5.845676	-2.791152	0.151971
H	-3.986893	-3.839164	0.090096
C	-6.532675	-1.614497	-0.159131
H	-6.383730	0.355188	-1.029401
H	-6.367324	-3.619846	0.621009
H	-7.588114	-1.520844	0.079282
H	-2.038960	-3.333282	0.586485

[6]TS2B'$_{endo-p}$

Fe	1.661129	0.059312	0.324460
N	3.250079	-0.170401	-0.996993
N	2.069949	-1.854457	1.015611
C	3.643303	-2.584076	-0.730840
C	3.846655	-1.359570	-1.360573
C	4.613920	0.174738	-2.825840
H	5.120295	0.704185	-3.622574
C	3.346591	2.126378	-1.872601
C	2.825061	-2.813981	0.373240
C	3.704440	0.780277	-1.885658
C	4.704153	-1.146407	-2.499560
H	5.298683	-1.915017	-2.976340
C	1.426181	-2.492992	2.055098
C	2.638677	-4.088589	1.016442
H	3.125354	-5.008139	0.717562
C	1.770374	-3.890996	2.053628
H	1.410128	-4.616961	2.771278
N	0.777721	0.443267	2.167798
N	1.885319	2.108767	0.107561
C	0.481764	2.878172	1.974501
C	0.285135	1.653888	2.606752
C	-0.487414	0.120273	4.070517
H	-0.960304	-0.400449	4.893296
C	0.595937	-1.871658	2.985291
C	1.219446	3.089723	0.814625
C	0.314858	-0.509937	3.052833
C	-0.499429	1.457618	3.798397
H	-0.989015	2.249494	4.350370
C	2.515861	2.745860	-0.942590
C	1.420861	4.367100	0.181219
H	1.010492	5.302942	0.537911
C	2.218679	4.154721	-0.905540
H	2.593080	4.882604	-1.613781
O	0.188064	-0.270905	-0.843016
C	-1.661512	1.270522	-0.728951
C	-0.676099	0.464493	-1.482979
H	-0.310495	0.933749	-2.405563
C	-2.104499	0.861303	0.536720
C	-3.022336	1.637342	1.237768
C	-2.150250	2.461734	-1.285564
H	-1.801703	2.783519	-2.264530
C	-3.068220	3.238434	-0.579901
H	-3.437096	4.165053	-1.009735
C	-3.506382	2.825297	0.681567
H	-4.223209	3.429272	1.230456
H	3.783114	2.758416	-2.639738
H	0.015260	3.746934	2.427652
H	0.159752	-2.502399	3.753799
H	4.181905	-3.438070	-1.129763
H	-1.715383	-0.056688	0.963690
H	-3.355680	1.317688	2.220219
C	-1.663766	-2.646774	-0.835750
C	-2.399159	-1.686151	-1.637139
C	-1.723320	-0.893304	-2.578945
H	-0.810447	-1.283326	-3.015407
H	-2.307514	-0.281665	-3.258605
C	-0.367641	-2.983945	-1.006768
H	0.235354	-2.643940	-1.839784
H	0.128724	-3.637442	-0.296526
C	-3.814968	-1.456599	-1.326312

C	-4.453503	-0.239910	-1.655094
C	-4.587019	-2.458307	-0.693964
C	-5.787327	-0.022484	-1.333558
H	-3.896456	0.561094	-2.123794
C	-5.928964	-2.247987	-0.398951
H	-4.149798	-3.425111	-0.469626
C	-6.531988	-1.025089	-0.706149
H	-6.247469	0.931080	-1.573478
H	-6.506951	-3.038877	0.069224
H	-7.577547	-0.857839	-0.464584
H	-2.177017	-3.059914	0.026484

^{2}VIB'$_{endo-p}$

Fe	1.845034	0.114026	0.086329
N	2.713604	-1.677462	-0.134102
N	1.915967	-0.143651	2.066216
C	2.858519	-2.408050	2.207858
C	3.024732	-2.603946	0.847254
C	3.423972	-3.665498	-1.093640
H	3.689792	-4.369753	-1.871121
C	2.819335	-1.764363	-2.588950
C	2.319545	-1.264768	2.772570
C	2.954739	-2.328731	-1.332109
C	3.482793	-3.830051	0.257271
H	3.801295	-4.699030	0.818005
C	1.399211	0.715790	3.019162
C	2.062032	-1.096373	4.175707
H	2.302757	-1.828428	4.935410
C	1.478346	0.124623	4.326727
H	1.145201	0.604949	5.237444
N	1.421421	2.033859	0.362922
N	2.196945	0.492375	-1.841509
C	1.622104	2.879861	-1.936326
C	1.328673	3.032445	-0.593864
C	0.594707	3.981705	1.306297
H	0.208188	4.644898	2.068490
C	0.930022	1.993274	2.769276
C	2.013053	1.685828	-2.520419
C	0.970702	2.614845	1.533583
C	0.829907	4.244349	-0.008668
H	0.673862	5.167418	-0.550996
C	2.500378	-0.436976	-2.821365
C	2.219167	1.500797	-3.928139
H	2.147447	2.287798	-4.667422
C	2.507249	0.181624	-4.116616
H	2.727087	-0.334643	-5.041956
O	-0.006123	-0.541575	-0.095917
C	-1.658336	1.263556	-0.298244
C	-0.937243	0.142140	-1.010255
H	-0.311297	0.547184	-1.806677
C	-1.994997	1.196123	1.056883
C	-2.751710	2.210945	1.643831
C	-2.077306	2.365461	-1.054353
H	-1.805528	2.431442	-2.105803
C	-2.841081	3.375653	-0.470138
H	-3.163173	4.223728	-1.067551
C	-3.182150	3.298624	0.882181
H	-3.776516	4.084283	1.339561
H	3.039064	-2.388725	-3.448681
H	1.507171	3.744856	-2.580985
H	0.548343	2.564795	3.608569
H	3.137116	-3.218286	2.873469
H	-1.655922	0.355366	1.650367
H	-3.009787	2.147282	2.697179
C	-1.774684	-2.165172	0.427431
C	-2.601016	-1.658258	-0.505622
C	-1.907496	-0.890980	-1.608895
H	-1.338147	-1.584347	-2.244421
H	-2.606742	-0.367021	-2.263898
C	-0.307481	-1.949814	0.277706
H	0.147365	-2.567004	-0.505414
H	0.236017	-2.105864	1.207107
C	-4.071235	-1.745500	-0.441175
C	-4.871453	-0.646488	-0.798959
C	-4.697151	-2.919440	0.012198
C	-6.259310	-0.719000	-0.695653
H	-4.407366	0.282391	-1.118856
C	-6.085037	-2.992571	0.104933
H	-4.089869	-3.784495	0.264019
C	-6.870414	-1.892016	-0.247595
H	-6.864273	0.142706	-0.962922
H	-6.555034	-3.911297	0.443871
H	-7.952689	-1.950139	-0.177095
H	-2.138332	-2.665865	1.319121

^{4}VIB'$_{endo-p}$

Fe	1.902259	0.120642	0.098134
N	2.756409	-1.671203	-0.124581

N	1.927557	-0.142350	2.076183
C	2.844116	-2.421688	2.218230
C	3.040929	-2.604310	0.860671
C	3.531483	-3.634486	-1.077679
H	3.835846	-4.327456	-1.851109
C	2.962462	-1.732161	-2.577974
C	2.313841	-1.273075	2.778660
C	3.060353	-2.302909	-1.321438
C	3.529974	-3.817160	0.273907
H	3.828929	-4.692166	0.836099
C	1.437352	0.731241	3.033323
C	2.060673	-1.100690	4.179901
H	2.282433	-1.840833	4.937586
C	1.508969	0.136292	4.336068
H	1.188711	0.621243	5.248897
N	1.422563	2.031434	0.366745
N	2.229441	0.497589	-1.836536
C	1.563115	2.863304	-1.945071
C	1.283813	3.014975	-0.600012
C	0.580423	3.971243	1.309324
H	0.196688	4.635368	2.072016
C	0.978045	2.012398	2.787770
C	2.000480	1.682204	-2.519111
C	0.992736	2.619081	1.545833
C	0.770034	4.219747	-0.016650
H	0.573239	5.130075	-0.567119
C	2.601899	-0.416859	-2.809691
C	2.237205	1.504198	-3.921651
H	2.135184	2.285014	-4.663940
C	2.599561	0.201728	-4.102790
H	2.859682	-0.303481	-5.023736
O	-0.069155	-0.566218	-0.134516
C	-1.690727	1.256259	-0.318637
C	-0.989935	0.123458	-1.036654
H	-0.365198	0.524504	-1.838973
C	-1.988650	1.201394	1.045937
C	-2.723739	2.224556	1.646702
C	-2.127167	2.354280	-1.070652
H	-1.887572	2.409386	-2.130646
C	-2.867684	3.373619	-0.472518
H	-3.204038	4.218154	-1.067281
C	-3.169759	3.309208	0.889942
H	-3.747202	4.101243	1.357999
H	3.230342	-2.342509	-3.433664
H	1.413521	3.718959	-2.594485
H	0.611936	2.590367	3.629150
H	3.106725	-3.236985	2.883606
H	-1.639363	0.360582	1.633729
H	-2.953261	2.169290	2.707210
C	-1.829194	-2.197432	0.377027
C	-2.666421	-1.667903	-0.532328
C	-1.987388	-0.886533	-1.635091
H	-1.438306	-1.575318	-2.293137
H	-2.694882	-0.345277	-2.266640
C	-0.362539	-1.971367	0.211565
H	0.081760	-2.584424	-0.583315
H	0.192591	-2.147767	1.132206
C	-4.136986	-1.752465	-0.448467
C	-4.938168	-0.641846	-0.764856
C	-4.761038	-2.935634	-0.018157
C	-6.324796	-0.712142	-0.643346
H	-4.474710	0.293000	-1.068185
C	-6.147902	-3.006263	0.093906
H	-4.153297	-3.809143	0.201446
C	-6.934220	-1.894247	-0.217383
H	-6.930147	0.158523	-0.879144
H	-6.616255	-3.932143	0.415444
H	-8.015603	-1.950355	-0.132473
H	-2.181955	-2.723171	1.258767

^{6}VIB'$_{endo\text{-}p}$

Fe	1.783441	0.081413	0.101318
N	2.806438	-1.704030	-0.060326
N	1.876307	-0.127443	2.146998
C	2.743429	-2.425780	2.294142
C	3.027006	-2.627472	0.945932
C	3.695842	-3.635238	-0.958660
H	4.079965	-4.320262	-1.703350
C	3.197366	-1.712195	-2.493639
C	2.215362	-1.265922	2.854424
C	3.219300	-2.307561	-1.235667
C	3.580030	-3.831606	0.388290
H	3.849475	-4.709299	0.961428
C	1.407871	0.782366	3.077108
C	1.941737	-1.068106	4.252796
H	2.120495	-1.803792	5.026215
C	1.440933	0.195143	4.389521
H	1.130744	0.696548	5.297105

N	1.416283	2.081422	0.362021
N	2.320925	0.496275	-1.848573
C	1.584274	2.841574	-1.973564
C	1.282599	3.035900	-0.630087
C	0.594836	4.036161	1.270718
H	0.221224	4.724156	2.017412
C	0.991212	2.078193	2.788399
C	2.075522	1.668073	-2.540547
C	1.002923	2.683876	1.536265
C	0.770765	4.254782	-0.065148
H	0.570394	5.157518	-0.627089
C	2.798609	-0.409516	-2.777992
C	2.397188	1.488167	-3.930480
H	2.301556	2.253410	-4.689859
C	2.842060	0.204799	-4.077242
H	3.181744	-0.285715	-4.980230
O	-0.108019	-0.611983	-0.204189
C	-1.709852	1.234879	-0.336885
C	-1.027670	0.114812	-1.089910
H	-0.395544	0.526120	-1.882402
C	-1.992044	1.151134	1.029516
C	-2.710322	2.167519	1.661062
C	-2.143671	2.352939	-1.060257
H	-1.913827	2.430855	-2.120879
C	-2.869124	3.364016	-0.430982
H	-3.203914	4.224808	-1.002688
C	-3.156012	3.271680	0.933141
H	-3.720432	4.058371	1.425327
H	3.554124	-2.310138	-3.326335
H	1.432219	3.684852	-2.639765
H	0.641553	2.679809	3.621213
H	2.965716	-3.243816	2.972036
H	-1.644861	0.294740	1.595805
H	-2.926182	2.091579	2.723002
C	-1.875254	-2.241974	0.278175
C	-2.711014	-1.678315	-0.612861
C	-2.031770	-0.878497	-1.702771
H	-1.490537	-1.556474	-2.378021
H	-2.738888	-0.319445	-2.318779
C	-0.408216	-2.032176	0.111405
H	0.031370	-2.619221	-0.704499
H	0.152777	-2.231837	1.024321
C	-4.181544	-1.745521	-0.519843
C	-4.970454	-0.618585	-0.808439
C	-4.817140	-2.929412	-0.108402
C	-6.356892	-0.673987	-0.678191
H	-4.497577	0.316622	-1.095685
C	-6.203857	-2.984801	0.011910
H	-4.219353	-3.814992	0.088907
C	-6.977994	-1.856823	-0.271560
H	-6.952852	0.208642	-0.892110
H	-6.681740	-3.910967	0.318012
H	-8.059347	-1.901138	-0.180065
H	-2.229507	-2.780538	1.151298

^{4}VB$_{endo\text{-}p}$

Fe	-2.060939	-0.122645	0.258502
N	-1.521478	-1.598150	1.493000
N	-2.429738	-1.474192	-1.162594
C	-1.695408	-3.528490	-0.026319
C	-1.397301	-2.946203	1.192219
C	-0.678398	-2.752337	3.314977
H	-0.298749	-2.918699	4.314537
C	-1.043713	-0.299534	3.530338
C	-2.170525	-2.834264	-1.123704
C	-1.080465	-1.475714	2.801832
C	-0.882152	-3.664068	2.321272
H	-0.699922	-4.730562	2.336904
C	-2.872837	-1.224811	-2.451056
C	-2.446842	-3.432518	-2.397241
H	-2.327816	-4.485333	-2.617283
C	-2.873567	-2.434157	-3.221990
H	-3.180390	-2.499673	-4.257630
N	-2.999659	1.259534	-0.837522
N	-2.034637	1.146887	1.804252
C	-2.975068	3.153618	0.732770
C	-3.248726	2.577094	-0.494628
C	-3.883616	2.404566	-2.645056
H	-4.251323	2.572204	-3.648903
C	-3.301225	0.000611	-2.929319
C	-2.403755	2.482808	1.799417
C	-3.379455	1.150340	-2.164077
C	-3.808913	3.286676	-1.608977
H	-4.100146	4.328660	-1.587419
C	-1.510585	0.918085	3.066763
C	-2.104750	3.087379	3.064707
H	-2.307259	4.121237	3.312086
C	-1.545599	2.120543	3.847239

H	-1.198120	2.197613	4.869249
O	-0.211391	0.457790	-0.468268
C	1.888100	1.556574	-0.417891
C	0.748764	0.909710	0.189719
H	0.708496	0.867946	1.286133
C	1.966268	1.707736	-1.816729
C	3.049210	2.372734	-2.373985
C	2.912174	2.058734	0.406892
H	2.854007	1.920993	1.483289
C	3.995824	2.719470	-0.158435
H	4.795869	3.093072	0.471501
C	4.060546	2.878516	-1.545661
H	4.909201	3.392788	-1.987165
H	-0.664133	-0.344214	4.545479
H	-3.218985	4.202045	0.865914
H	-3.627931	0.055616	-3.962107
H	-1.537248	-4.596247	-0.130177
H	1.170723	1.304252	-2.434329
H	3.116067	2.498882	-3.450254
C	2.464063	-2.382912	-1.175763
C	2.907905	-1.646026	0.029188
C	2.113854	-1.572543	1.118762
H	1.179541	-2.116687	1.165869
H	2.396484	-1.005520	2.000725
C	1.225342	-2.341769	-1.680581
H	0.448962	-1.721370	-1.246246
H	0.955013	-2.917856	-2.561001
C	4.239571	-0.993923	-0.028884
C	4.991385	-0.742432	1.134379
C	4.777415	-0.596804	-1.264837
C	6.224391	-0.099033	1.064400
H	4.622194	-1.085249	2.096435
C	6.012196	0.045750	-1.335782
H	4.209105	-0.766489	-2.173982
C	6.740012	0.299648	-0.172115
H	6.793968	0.074161	1.973282
H	6.405963	0.348899	-2.301928
H	7.707216	0.791245	-0.227568
H	3.228618	-2.972158	-1.681332

^{2}TS2B$_{endo-p}$

Fe	1.874742	0.244787	-0.228527
N	1.904973	-0.798237	-1.935516
N	2.762123	-1.290740	0.705466
C	2.597768	-2.989899	-1.064299
C	2.150339	-2.152398	-2.073339
C	1.554905	-1.459022	-4.129593
H	1.317716	-1.372436	-5.181985
C	1.156277	0.928415	-3.518853
C	2.910073	-2.577931	0.220884
C	1.534346	-0.366137	-3.197446
C	1.928304	-2.567872	-3.431375
H	2.064899	-3.578487	-3.793908
C	3.191940	-1.346705	2.018272
C	3.443917	-3.441663	1.239510
H	3.660367	-4.493005	1.100877
C	3.625549	-2.676385	2.351431
H	4.022510	-2.969219	3.314753
N	2.229088	1.382746	1.379354
N	1.389176	1.883612	-1.268209
C	1.273352	3.499451	0.577504
C	1.824213	2.690337	1.557281
C	2.647107	2.064408	3.555200
H	2.988401	2.005080	4.580468
C	3.182087	-0.284929	2.907395
C	1.092806	3.126190	-0.743707
C	2.719381	0.984581	2.607363
C	2.082430	3.119328	2.906859
H	1.866542	4.109086	3.287796
C	1.078819	1.973013	-2.612917
C	0.604385	4.003648	-1.773108
H	0.313462	5.033652	-1.613192
C	0.604711	3.291799	-2.933611
H	0.311853	3.614701	-3.924112
O	0.131323	-0.134735	0.307961
C	-1.987016	0.843157	-0.192235
C	-0.866112	-0.061910	-0.544525
H	-0.605552	-0.064864	-1.607122
C	-2.005354	1.482851	1.055180
C	-3.052177	2.343812	1.379408
C	-3.024237	1.071907	-1.106975
H	-3.009104	0.578629	-2.075920
C	-4.074732	1.923693	-0.775128
H	-4.878833	2.093804	-1.484801
C	-4.090741	2.559907	0.469652
H	-4.908089	3.227817	0.726002
H	0.882745	1.132478	-4.548906
H	0.996602	4.510795	0.856468

H	3.543902	-0.466230	3.914225
H	2.757697	-4.034460	-1.311060
H	-1.185781	1.309659	1.744167
H	-3.058596	2.848020	2.341700
C	-1.576389	-2.394772	1.732998
C	-2.253823	-2.088095	0.490562
C	-1.546744	-1.905048	-0.711682
H	-0.579819	-2.380926	-0.842370
H	-2.125353	-1.815176	-1.626152
C	-0.239520	-2.515890	1.872041
H	0.451897	-2.431983	1.045586
H	0.200117	-2.704373	2.846594
C	-3.685271	-1.781318	0.528584
C	-4.522947	-2.090266	-0.565268
C	-4.244664	-1.110816	1.637361
C	-5.869422	-1.746988	-0.546969
H	-4.123944	-2.643329	-1.409829
C	-5.584024	-0.738632	1.636609
H	-3.612958	-0.822690	2.470010
C	-6.400834	-1.060590	0.549306
H	-6.506901	-2.014659	-1.384088
H	-5.992264	-0.195037	2.482895
H	-7.450543	-0.781908	0.557741
H	-2.204591	-2.518544	2.610283

^{4}TS2B$_{endo\text{-}p}$

Fe	1.857003	0.350813	0.008907
N	2.375025	-0.315195	-1.814915
N	2.771873	-1.236289	0.825713
C	3.290691	-2.517036	-1.210738
C	2.895484	-1.557903	-2.128104
C	2.503972	-0.569862	-4.111325
H	2.418153	-0.314873	-5.159610
C	1.589171	1.563096	-3.195190
C	3.242105	-2.356106	0.164458
C	2.128506	0.297384	-3.029619
C	2.977810	-1.719368	-3.552910
H	3.361650	-2.601556	-4.048606
C	2.901106	-1.521668	2.172180
C	3.670022	-3.349895	1.108658
H	4.086427	-4.312047	0.839927
C	3.459710	-2.832368	2.352748
H	3.669973	-3.282167	3.314463
N	1.749839	1.193398	1.828462
N	1.352497	2.114203	-0.809548
C	0.788108	3.375211	1.225168
C	1.212305	2.427307	2.140557
C	1.643958	1.459555	4.124791
H	1.762195	1.217995	5.173082
C	2.545102	-0.677605	3.210630
C	0.862386	3.225721	-0.148296
C	2.011973	0.588638	3.042486
C	1.146960	2.598108	3.565718
H	0.773291	3.485563	4.059661
C	1.233843	2.406495	-2.155885
C	0.438239	4.221456	-1.091559
H	0.022403	5.183149	-0.821124
C	0.669306	3.715016	-2.335137
H	0.483987	4.175898	-3.296619
O	0.058715	-0.398618	0.018389
C	-1.996209	0.754273	-0.394684
C	-0.873912	-0.113696	-0.851764
H	-0.528165	0.110281	-1.871235
C	-2.103440	1.090835	0.961200
C	-3.136534	1.919565	1.394173
C	-2.931379	1.254856	-1.309341
H	-2.844430	1.002804	-2.364127
C	-3.969108	2.075865	-0.873259
H	-4.692884	2.459520	-1.586409
C	-4.074303	2.408051	0.480541
H	-4.881583	3.050889	0.819751
H	1.444264	1.921600	-4.208772
H	0.377422	4.302090	1.610671
H	2.701102	-1.030204	4.224635
H	3.683616	-3.452146	-1.595654
H	-1.359869	0.710830	1.653942
H	-3.211001	2.186135	2.444905
C	-1.505367	-2.919201	0.773933
C	-2.259348	-2.300100	-0.291107
C	-1.614605	-1.798273	-1.439764
H	-0.681662	-2.255904	-1.755294
H	-2.241866	-1.466751	-2.261805
C	-0.160458	-3.054798	0.784168
H	0.473819	-2.769463	-0.044101
H	0.348082	-3.470277	1.649013
C	-3.680541	-2.014282	-0.084150
C	-4.582207	-2.006629	-1.170517
C	-4.170854	-1.686931	1.198495

C	-5.922148	-1.691218	-0.979656
H	-4.237023	-2.294180	-2.158635
C	-5.505769	-1.344142	1.380306
H	-3.490855	-1.642420	2.042209
C	-6.385515	-1.350036	0.294470
H	-6.607497	-1.713062	-1.821451
H	-5.861333	-1.067790	2.368150
H	-7.430668	-1.093647	0.441145
H	-2.068346	-3.267151	1.635123

^{6}TS2B$_{endo\text{-}p}$

Fe	1.814387	0.051929	-0.202325
N	1.609229	-0.915240	-2.037377
N	2.572543	-1.729183	0.552172
C	1.988210	-3.235686	-1.305961
C	1.610478	-2.280780	-2.246801
C	0.916824	-1.368378	-4.190957
H	0.584795	-1.186957	-5.205126
C	1.080392	1.030501	-3.449834
C	2.451496	-2.981872	-0.016597
C	1.196629	-0.340039	-3.222581
C	1.173579	-2.566776	-3.588828
H	1.091952	-3.559415	-4.012721
C	3.056010	-1.926148	1.830254
C	2.859370	-3.986836	0.930113
H	2.868235	-5.051392	0.734564
C	3.231980	-3.334856	2.070883
H	3.608861	-3.761067	2.991739
N	2.769054	1.026507	1.350426
N	1.837128	1.840560	-1.254180
C	2.482453	3.346501	0.582998
C	2.837041	2.391479	1.532827
C	3.586267	1.484480	3.457912
H	3.981431	1.304990	4.449416
C	3.345943	-0.915138	2.743807
C	2.027857	3.093431	-0.708205
C	3.228266	0.453344	2.517317
C	3.345325	2.681637	2.849406
H	3.503856	3.677045	3.243639
C	1.391130	2.041477	-2.543620
C	1.693274	4.103399	-1.679847
H	1.761674	5.169892	-1.508745
C	1.298802	3.454173	-2.812856
H	0.980662	3.883308	-3.754193
O	-0.016747	0.056636	0.373972
C	-2.104876	1.180263	0.059198
C	-1.044787	0.257684	-0.422118
H	-0.809275	0.364489	-1.487900
C	-2.093514	1.635125	1.384629
C	-3.092399	2.497803	1.832813
C	-3.126663	1.591089	-0.807882
H	-3.137238	1.238874	-1.836883
C	-4.129997	2.444128	-0.353986
H	-4.921373	2.756299	-1.028873
C	-4.114156	2.898820	0.967598
H	-4.893137	3.568943	1.319645
H	0.741691	1.338770	-4.434075
H	2.587144	4.387833	0.870985
H	3.724466	-1.222897	3.713572
H	1.941623	-4.275315	-1.615018
H	-1.290616	1.317180	2.041497
H	-3.074125	2.859815	2.856942
C	-1.917310	-2.207421	1.631078
C	-2.562249	-1.757574	0.416797
C	-1.824534	-1.491641	-0.754838
H	-0.891372	-2.019097	-0.926977
H	-2.387821	-1.292907	-1.661708
C	-0.593085	-2.435437	1.761788
H	0.107791	-2.347425	0.944106
H	-0.175025	-2.731571	2.718954
C	-3.979626	-1.396236	0.465990
C	-4.805866	-1.556196	-0.668477
C	-4.539417	-0.829325	1.631499
C	-6.140492	-1.171069	-0.635028
H	-4.409501	-2.029145	-1.561442
C	-5.865387	-0.413227	1.648907
H	-3.915947	-0.650986	2.500098
C	-6.671015	-0.589110	0.520446
H	-6.769514	-1.324968	-1.506445
H	-6.272238	0.050821	2.541922
H	-7.711099	-0.277084	0.542239
H	-2.557412	-2.359296	2.495106

^{4}VIB$_{endo\text{-}p}$

Fe	-1.903481	0.096869	-0.038012
N	-2.213956	-0.013432	1.934433
N	-2.700556	-1.723270	-0.247204
C	-2.894590	-2.371565	2.120898

C	-2.559141	-1.142592	2.660384
C	-2.216770	0.460139	4.201000
H	-2.120429	1.040880	5.109012
C	-1.602140	2.273003	2.605704
C	-2.983014	-2.629146	0.764624
C	-2.000303	0.976082	2.881176
C	-2.555210	-0.853991	4.064648
H	-2.796642	-1.571524	4.837900
C	-2.952268	-2.407260	-1.426700
C	-3.411018	-3.879744	0.210308
H	-3.693208	-4.744618	0.796304
C	-3.400325	-3.739551	-1.146200
H	-3.668444	-4.466968	-1.901193
N	-1.947744	0.316347	-2.021218
N	-1.491293	2.033333	0.158265
C	-1.111157	2.622864	-2.201198
C	-1.517141	1.417724	-2.743635
C	-2.066172	-0.118013	-4.292716
H	-2.263482	-0.666495	-5.204475
C	-2.765166	-1.902863	-2.701558
C	-1.117115	2.908679	-0.848897
C	-2.289408	-0.632068	-2.972373
C	-1.581390	1.148298	-4.150541
H	-1.302304	1.853819	-4.922137
C	-1.359572	2.756897	1.333847
C	-0.747022	4.178130	-0.296990
H	-0.409389	5.023396	-0.881346
C	-0.904653	4.086985	1.053323
H	-0.724486	4.843092	1.805944
O	0.081658	-0.576011	0.029651
C	1.682278	1.229116	0.416546
C	1.021261	-0.006211	0.990559
H	0.409997	0.274434	1.852007
C	1.884578	1.396484	-0.956272
C	2.553119	2.523297	-1.436833
C	2.148824	2.209497	1.300249
H	1.983696	2.092572	2.369468
C	2.822424	3.332647	0.821936
H	3.182500	4.084263	1.518625
C	3.027572	3.491552	-0.550599
H	3.552564	4.365380	-0.925667
H	-1.462780	2.949952	3.441578
H	-0.788372	3.403017	-2.881584
H	-2.997322	-2.547981	-3.541960
H	-3.143436	-3.174763	2.806045
H	1.505968	0.651386	-1.646372
H	2.704802	2.642412	-2.506185
C	1.888810	-2.039450	-0.776349
C	2.718583	-1.659959	0.210643
C	2.036413	-1.078873	1.430894
H	1.501002	-1.857489	1.992286
H	2.745517	-0.607229	2.114846
C	0.416549	-1.863311	-0.600722
H	-0.029444	-2.641090	0.032870
H	-0.116295	-1.854291	-1.552256
C	4.189703	-1.712058	0.120668
C	4.969198	-1.975967	1.259852
C	4.843099	-1.501279	-1.106030
C	6.357994	-2.051893	1.169987
H	4.487248	-2.150643	2.218233
C	6.231225	-1.571517	-1.193792
H	4.258375	-1.245912	-1.984946
C	6.993558	-1.850357	-0.056778
H	6.944053	-2.269607	2.058266
H	6.720315	-1.396000	-2.147778
H	8.076320	-1.901518	-0.124979
H	2.246086	-2.472181	-1.705720

^{2}VB$_{exo\text{-}p}$

Fe	1.651814	0.111993	0.451269
N	1.704266	2.075594	0.870365
N	0.939411	-0.224292	2.291023
O	0.024024	0.316390	-0.553794
C	0.682239	2.083806	3.103052
C	1.209281	2.719063	1.990760
C	1.784977	4.372995	0.582281
H	1.985438	5.320353	0.099123
C	-2.013729	1.450692	-0.943574
C	2.602458	2.902061	-1.261004
C	0.562427	0.711029	3.242956
C	2.047984	3.087467	-0.004950
C	1.272651	4.144449	1.823317
H	0.961854	4.864444	2.569036
C	-0.956688	0.959625	-0.103107
H	-0.994106	1.198494	0.964131
C	0.618780	-1.450003	2.844760
C	0.015138	0.060429	4.399732
H	-0.334676	0.575115	5.285074

C	0.045925	-1.279383	4.151532
H	-0.274329	-2.091979	4.790539
C	-1.974999	1.268938	-2.342065
C	-2.991716	1.795075	-3.125481
C	-3.080008	2.150705	-0.343678
H	-3.107610	2.265376	0.735586
C	-4.096761	2.667789	-1.136639
H	-4.924097	3.203574	-0.682517
C	-4.049529	2.491773	-2.523769
H	-4.842579	2.900754	-3.143416
N	1.985021	-1.840458	0.239640
N	2.736987	0.449855	-1.190244
C	3.222293	-1.853748	-1.883594
C	2.632869	-2.482821	-0.802384
C	1.888575	-4.141352	0.519726
H	1.650768	-5.089109	0.984839
C	0.856565	-2.680204	2.254168
C	3.249311	-0.482561	-2.071935
C	1.519413	-2.857894	1.053007
C	2.591806	-3.907735	-0.621659
H	3.047611	-4.623796	-1.293022
C	2.939738	1.673048	-1.800594
C	3.800523	0.165122	-3.230210
H	4.276995	-0.351287	-4.053169
C	3.600108	1.501519	-3.066748
H	3.881111	2.312191	-3.726299
H	2.822935	3.787231	-1.848574
H	3.682129	-2.477797	-2.642736
H	0.549685	-3.567562	2.797738
H	0.334582	2.705630	3.921553
H	-2.971201	1.665292	-4.203021
H	-1.148495	0.720990	-2.780254
C	-2.646628	-2.148687	-1.589920
C	-0.447042	-3.110063	-2.260260
H	-0.315613	-2.370835	-3.046409
H	0.333939	-3.853691	-2.146960
C	-3.121273	-1.817286	-2.807067
H	-2.739589	-2.294557	-3.703650
H	-3.896630	-1.070883	-2.941939
C	-1.519015	-3.099300	-1.460980
H	-1.585116	-3.823872	-0.649229
C	-3.205195	-1.565810	-0.343403
C	-2.402656	-1.450386	0.807424
C	-4.519786	-1.072220	-0.280152
C	-2.885457	-0.836742	1.966457
H	-1.386821	-1.831126	0.789622
C	-4.999466	-0.452739	0.871489
H	-5.177555	-1.187755	-1.135614
C	-4.182882	-0.326034	2.000140
H	-2.244578	-0.761231	2.839335
H	-6.020319	-0.081541	0.894991
H	-4.564285	0.146049	2.901322

$^{4}VB_{exo\text{-}p}$

Fe	1.709063	0.077300	0.434366
N	1.958190	2.057121	0.629033
N	0.945366	0.020983	2.285389
O	-0.059617	0.347177	-0.617501
C	0.870790	2.427586	2.804947
C	1.494012	2.868555	1.650847
C	2.343843	4.283290	0.122994
H	2.682238	5.144701	-0.437756
C	-2.080148	1.522344	-1.030792
C	3.053027	2.550544	-1.518493
C	0.631596	1.097099	3.102849
C	2.477516	2.924704	-0.317135
C	1.737115	4.248630	1.343188
H	1.474947	5.075706	1.989969
C	-1.018576	1.020453	-0.190337
H	-1.052982	1.283053	0.874818
C	0.539961	-1.101296	2.990491
C	0.023334	0.640309	4.317764
H	-0.303699	1.288620	5.120190
C	-0.032349	-0.721309	4.248804
H	-0.415458	-1.418835	4.982237
C	-2.069717	1.295699	-2.421368
C	-3.092145	1.811630	-3.205464
C	-3.122535	2.259041	-0.437727
H	-3.130246	2.408813	0.637821
C	-4.146652	2.766499	-1.229083
H	-4.957406	3.329596	-0.777801
C	-4.128436	2.544331	-2.609748
H	-4.926846	2.944294	-3.228466
N	1.841294	-1.913460	0.451548
N	2.819616	0.118548	-1.223078
C	3.025633	-2.289799	-1.672809
C	2.396226	-2.727362	-0.521861
C	1.537601	-4.143331	0.999931

H	1.223313	-5.005589	1.573258
C	0.683064	-2.410304	2.566249
C	3.213406	-0.958487	-1.997282
C	1.309546	-2.782201	1.390706
C	2.220215	-4.109072	-0.178987
H	2.578350	-4.937830	-0.775791
C	3.219331	1.240209	-1.928987
C	3.863445	-0.504644	-3.193098
H	4.268399	-1.157424	-3.955257
C	3.863465	0.857518	-3.152799
H	4.269248	1.554054	-3.874752
H	3.417864	3.335978	-2.171509
H	3.401951	-3.038550	-2.361360
H	0.313096	-3.196743	3.215034
H	0.563544	3.172262	3.531323
H	-3.092789	1.645303	-4.278326
H	-1.261596	0.716439	-2.854010
C	-2.753096	-2.131399	-1.465419
C	-0.621561	-3.270782	-2.083410
H	-0.451579	-2.599777	-2.921448
H	0.108758	-4.056786	-1.925763
C	-3.197350	-1.828566	-2.701191
H	-2.832042	-2.361518	-3.572933
H	-3.931187	-1.049325	-2.875902
C	-1.680997	-3.134666	-1.278429
H	-1.782654	-3.796284	-0.418088
C	-3.302946	-1.473363	-0.252176
C	-2.508963	-1.328666	0.900582
C	-4.606040	-0.946592	-0.224623
C	-2.987850	-0.652914	2.025510
H	-1.501543	-1.732534	0.908367
C	-5.082787	-0.268322	0.894304
H	-5.256748	-1.083581	-1.082345
C	-4.273704	-0.112007	2.024323
H	-2.352480	-0.549416	2.899851
H	-6.094520	0.127650	0.890945
H	-4.651532	0.408001	2.900315

^{6}VB$_{exo\text{-}p}$

Fe	1.565318	0.122648	0.368174
N	1.821223	2.134878	0.802280
N	0.844884	-0.111065	2.300689
O	-0.150037	0.423121	-0.652711
C	0.605296	2.256827	2.937752
C	1.254108	2.834078	1.850100
C	2.116495	4.400694	0.475409
H	2.439661	5.319843	0.004147
C	-2.233919	1.505400	-0.989067
C	3.033691	2.805246	-1.233321
C	0.438505	0.892361	3.162200
C	2.355007	3.082423	-0.050267
C	1.437276	4.247638	1.649423
H	1.094200	5.016596	2.329493
C	-1.155894	1.005548	-0.180198
H	-1.206176	1.179561	0.901099
C	0.526209	-1.305113	2.921495
C	-0.160296	0.314624	4.334914
H	-0.554083	0.880893	5.169050
C	-0.103202	-1.042941	4.187790
H	-0.443724	-1.805502	4.876353
C	-2.206850	1.378730	-2.393691
C	-3.256049	1.888878	-3.144181
C	-3.319973	2.136581	-0.350195
H	-3.338891	2.205920	0.733256
C	-4.368446	2.640411	-1.110082
H	-5.211339	3.122990	-0.625884
C	-4.333501	2.517621	-2.503172
H	-5.152073	2.914410	-3.097046
N	1.980253	-1.897045	0.359811
N	2.923653	0.347658	-1.161863
C	3.314015	-2.018519	-1.707622
C	2.654947	-2.593010	-0.625372
C	1.856329	-4.162465	0.784686
H	1.602037	-5.082176	1.295376
C	0.800390	-2.570947	2.412515
C	3.442159	-0.655804	-1.956991
C	1.492466	-2.846429	1.237322
C	2.581591	-4.005301	-0.361264
H	3.036280	-4.771454	-0.975988
C	3.311342	1.539976	-1.741788
C	4.156345	-0.079637	-3.064976
H	4.662178	-0.647766	-3.834823
C	4.073267	1.276687	-2.933626
H	4.498383	2.038334	-3.574332
H	3.403490	3.654771	-1.798741
H	3.790932	-2.695261	-2.409522
H	0.483524	-3.420641	3.008832
H	0.221371	2.930988	3.696989

H	-3.245815	1.800045	-4.226062
H	-1.366110	0.879085	-2.861613
C	-2.560072	-2.156661	-1.636856
C	-0.321677	-3.086272	-2.227240
H	-0.170048	-2.347472	-3.010159
H	0.460830	-3.823707	-2.087223
C	-2.984195	-1.816480	-2.870236
H	-2.547802	-2.268093	-3.755045
H	-3.773497	-1.090008	-3.030415
C	-1.421064	-3.086652	-1.465693
H	-1.506476	-3.812325	-0.656772
C	-3.198637	-1.614432	-0.410444
C	-2.458177	-1.488998	0.780227
C	-4.534510	-1.176526	-0.403488
C	-3.021809	-0.918312	1.924581
H	-1.428619	-1.832155	0.807587
C	-5.095716	-0.602533	0.734264
H	-5.143774	-1.302136	-1.292735
C	-4.340502	-0.464543	1.903894
H	-2.425934	-0.830422	2.827960
H	-6.131498	-0.275108	0.714540
H	-4.784765	-0.026909	2.793583

^{2}TS2B$_{exo\text{-}p}$

Fe	0.979639	-0.845094	0.721211
N	1.243007	0.851295	1.761260
N	-0.753139	-1.123547	1.681287
O	0.396917	-0.010297	-0.858526
C	-0.947484	1.030136	2.855004
C	0.303077	1.521896	2.523404
C	2.073677	2.904150	2.440177
H	2.778282	3.718017	2.549237
C	0.441373	2.372854	-1.088932
C	3.518894	1.427061	1.043282
C	-1.422246	-0.220124	2.489461
C	2.333407	1.695904	1.705525
C	0.813179	2.798352	2.943751
H	0.269193	3.504841	3.557002
C	-0.277779	1.107027	-0.811071
H	-1.077788	1.177957	-0.065756
C	-1.588247	-2.224011	1.598330
C	-2.676752	-0.766496	2.923864
H	-3.375229	-0.256042	3.573889
C	-2.773290	-2.014228	2.383784
H	-3.568519	-2.739781	2.498757
C	1.690393	2.351969	-1.726477
C	2.383481	3.541780	-1.929791
C	-0.107765	3.591047	-0.662192
H	-1.072564	3.599973	-0.160006
C	0.591202	4.778540	-0.863492
H	0.170994	5.719956	-0.522253
C	1.836280	4.755156	-1.499810
H	2.380136	5.681874	-1.658620
N	0.877821	-2.721600	0.018823
N	2.900825	-0.780256	0.152820
C	3.030381	-2.855713	-1.153872
C	1.786245	-3.354732	-0.807378
C	0.056340	-4.782710	-0.650990
H	-0.623497	-5.622414	-0.716866
C	-1.336765	-3.368430	0.860237
C	3.552084	-1.660337	-0.689770
C	-0.188565	-3.592054	0.119598
C	1.278732	-4.631687	-1.232849
H	1.811875	-5.321983	-1.873576
C	3.776722	0.274385	0.321247
C	4.859248	-1.164318	-1.027325
H	5.567846	-1.682612	-1.660100
C	5.000636	0.033059	-0.395790
H	5.848896	0.705145	-0.403830
H	4.298319	2.180542	1.082589
H	3.648907	-3.453781	-1.815091
H	-2.086716	-4.152745	0.876699
H	-1.585819	1.649667	3.476421
H	3.353626	3.525583	-2.417993
H	2.111963	1.399068	-2.029089
C	-2.370470	0.053285	-2.104222
C	-0.847823	-1.831728	-2.647518
H	-0.018896	-1.195904	-2.931512
H	-0.666987	-2.901306	-2.679635
C	-1.468412	1.057100	-2.471935
H	-0.679486	0.844938	-3.185351
H	-1.804752	2.087524	-2.463254
C	-2.042179	-1.351851	-2.243049
H	-2.819618	-2.061023	-1.975662
C	-3.622350	0.423519	-1.428266
C	-4.130058	-0.371906	-0.381526
C	-4.322149	1.596466	-1.783712
C	-5.284583	0.002469	0.298410

H	-3.591579	-1.258007	-0.066727
C	-5.490057	1.953510	-1.118616
H	-3.973747	2.202576	-2.614187
C	-5.971966	1.160884	-0.071780
H	-5.645834	-0.609496	1.119333
H	-6.030876	2.845480	-1.420042
H	-6.882139	1.444175	0.448742

^{4}TS2B$_{exo-p}$

Fe	0.999004	-0.856074	0.699912
N	1.302879	0.824208	1.755862
N	-0.720161	-1.137268	1.705200
O	0.292817	0.044492	-0.883558
C	-0.886832	1.031398	2.855176
C	0.367968	1.505969	2.513322
C	2.160815	2.862490	2.436655
H	2.874440	3.668299	2.546300
C	0.383446	2.426938	-1.100167
C	3.589229	1.375737	1.038269
C	-1.372091	-0.219783	2.508566
C	2.408759	1.652392	1.704851
C	0.894614	2.775282	2.931701
H	0.356267	3.492149	3.537838
C	-0.376822	1.163812	-0.865921
H	-1.163626	1.253933	-0.104611
C	-1.554628	-2.238974	1.651171
C	-2.622481	-0.756842	2.966231
H	-3.312573	-0.233888	3.615303
C	-2.728791	-2.013472	2.447521
H	-3.524970	-2.734348	2.583026
C	1.649190	2.384441	-1.698892
C	2.380120	3.558684	-1.861098
C	-0.144700	3.651100	-0.669038
H	-1.124236	3.678294	-0.196212
C	0.590053	4.824221	-0.829470
H	0.182885	5.770682	-0.485922
C	1.852695	4.779335	-1.427953
H	2.424944	5.693870	-1.554793
N	0.869949	-2.732206	0.006998
N	2.915178	-0.795324	0.101613
C	2.994746	-2.862603	-1.225688
C	1.755947	-3.356411	-0.851098
C	0.026019	-4.782159	-0.653181
H	-0.656359	-5.620442	-0.709296
C	-1.321421	-3.387209	0.913242
C	3.538545	-1.678668	-0.757942
C	-0.194700	-3.604147	0.137917
C	1.230907	-4.624693	-1.273355
H	1.740358	-5.308011	-1.940343
C	3.817305	0.233225	0.290753
C	4.851453	-1.202486	-1.098522
H	5.542137	-1.722184	-1.749704
C	5.026900	-0.021728	-0.442448
H	5.890430	0.630552	-0.445513
H	4.381467	2.114750	1.086835
H	3.593405	-3.460991	-1.904220
H	-2.069953	-4.171867	0.947675
H	-1.514458	1.662831	3.475076
H	3.364306	3.523638	-2.319681
H	2.055166	1.425133	-2.003298
C	-2.417818	0.096229	-2.117329
C	-0.845733	-1.734023	-2.683219
H	-0.045992	-1.072608	-2.989930
H	-0.627131	-2.797259	-2.703665
C	-1.525454	1.131846	-2.446846
H	-0.757482	0.956732	-3.193602
H	-1.902080	2.149330	-2.431333
C	-2.055662	-1.293197	-2.273325
H	-2.806310	-2.027702	-1.997122
C	-3.666938	0.425920	-1.418388
C	-4.126897	-0.384009	-0.359914
C	-4.406731	1.578907	-1.756783
C	-5.276389	-0.042440	0.345422
H	-3.554143	-1.252684	-0.055558
C	-5.570572	1.901073	-1.066965
H	-4.091556	2.195953	-2.592597
C	-6.005738	1.094859	-0.010233
H	-5.601008	-0.663104	1.175097
H	-6.143463	2.777160	-1.355440
H	-6.911910	1.351802	0.530580

^{6}TS2B$_{exo-p}$

Fe	1.063606	-0.743199	0.551241
N	1.392943	0.926903	1.740055
N	-0.586406	-1.158279	1.746362
O	0.290404	0.182017	-0.928261
C	-0.757978	0.995358	2.928278
C	0.457876	1.550981	2.539251

C	2.171161	3.007981	2.358320
H	2.839821	3.855710	2.431169
C	0.038831	2.557195	-1.082125
C	3.613930	1.561979	0.892851
C	-1.225361	-0.274665	2.592705
C	2.451026	1.803432	1.619036
C	0.939686	2.853699	2.924394
H	0.401337	3.548799	3.555616
C	-0.522311	1.202284	-0.860181
H	-1.312428	1.151818	-0.101361
C	-1.363509	-2.299056	1.711695
C	-2.433585	-0.873415	3.097410
H	-3.116200	-0.395769	3.788291
C	-2.513192	-2.127651	2.562564
H	-3.274319	-2.879428	2.729431
C	1.314174	2.711655	-1.643756
C	1.857506	3.985673	-1.786645
C	-0.687101	3.684398	-0.668770
H	-1.672442	3.557690	-0.225386
C	-0.139698	4.956301	-0.812617
H	-0.698222	5.828064	-0.485006
C	1.132436	5.108021	-1.373889
H	1.559015	6.100606	-1.486032
N	1.065532	-2.737088	-0.009198
N	3.062523	-0.667788	0.009658
C	3.208459	-2.791870	-1.220134
C	1.985982	-3.342231	-0.840769
C	0.304966	-4.830259	-0.605925
H	-0.339085	-5.699521	-0.643044
C	-1.086820	-3.436512	0.957199
C	3.716289	-1.561344	-0.811589
C	0.035542	-3.639437	0.157649
C	1.508185	-4.644850	-1.226486
H	2.041629	-5.332480	-1.870105
C	3.902482	0.415667	0.156210
C	5.002792	-1.028602	-1.183966
H	5.720375	-1.534446	-1.816928
C	5.118237	0.191733	-0.584981
H	5.949808	0.883138	-0.629286
H	4.367545	2.343063	0.900552
H	3.834907	-3.390079	-1.874472
H	-1.801194	-4.251991	1.016877
H	-1.384060	1.591480	3.584913
H	2.848292	4.105895	-2.215095
H	1.873342	1.828489	-1.934685
C	-2.461579	-0.101338	-2.092142
C	-0.733516	-1.791650	-2.675070
H	-0.013373	-1.067280	-3.035354
H	-0.417227	-2.830359	-2.680886
C	-1.706261	1.011870	-2.484007
H	-0.913248	0.902818	-3.216233
H	-2.181405	1.986630	-2.475605
C	-1.958571	-1.454166	-2.219085
H	-2.619863	-2.249440	-1.888454
C	-3.722421	0.114773	-1.367719
C	-4.062712	-0.699416	-0.268113
C	-4.590626	1.169401	-1.721556
C	-5.220029	-0.452487	0.463785
H	-3.394797	-1.493579	0.045562
C	-5.761246	1.393481	-1.005141
H	-4.368193	1.783852	-2.588559
C	-6.076055	0.587654	0.093862
H	-5.450927	-1.071318	1.325482
H	-6.432742	2.192210	-1.305186
H	-6.987406	0.768734	0.656204

^{2}VIB$_{exo\text{-}p}$

Fe	0.856372	-0.976244	0.688642
N	2.449472	0.149644	1.042131
N	-0.136741	0.018960	2.103816
O	0.236638	0.016919	-0.917280
C	1.561756	1.673773	2.752313
C	2.552068	1.239569	1.890229
C	4.472392	1.232064	0.722350
H	5.448299	1.438624	0.303592
C	1.018316	2.329615	-1.029647
C	3.990563	-0.822231	-0.605115
C	0.296712	1.115442	2.831978
C	3.630373	0.139604	0.322157
C	3.811546	1.902986	1.705552
H	4.129378	2.778624	2.255558
C	-0.163012	1.429579	-0.762584
H	-0.479204	1.520304	0.275343
C	-1.478348	-0.116361	2.418751
C	-0.777085	1.652814	3.617715
H	-0.685464	2.502258	4.281894
C	-1.881770	0.900606	3.347747
H	-2.881690	1.002754	3.748595

C	1.988692	2.007076	-1.983857
C	3.029972	2.895248	-2.254326
C	1.106406	3.546827	-0.344961
H	0.363776	3.793444	0.410767
C	2.142479	4.438857	-0.621335
H	2.199903	5.382626	-0.086632
C	3.106435	4.114753	-1.578460
H	3.915306	4.807065	-1.793002
N	-0.599727	-2.348964	0.678065
N	1.994476	-2.232325	-0.366293
C	0.444921	-4.112896	-0.678699
C	-0.618789	-3.569682	0.024327
C	-2.704269	-3.308315	0.823239
H	-3.742875	-3.419963	1.106569
C	-2.306146	-1.123795	1.955222
C	1.657945	-3.475649	-0.876577
C	-1.881524	-2.184912	1.174599
C	-1.917132	-4.174262	0.123715
H	-2.178754	-5.138576	-0.291809
C	3.241873	-1.951534	-0.892214
C	2.715615	-3.981732	-1.706472
H	2.697561	-4.941816	-2.205417
C	3.688978	-3.029440	-1.731148
H	4.638929	-3.049813	-2.249111
H	4.953098	-0.716316	-1.094328
H	0.301390	-5.083241	-1.142445
H	-3.343640	-1.110156	2.270816
H	1.771667	2.541596	3.368443
H	3.781582	2.634251	-2.994067
H	1.944492	1.050185	-2.494289
C	-2.419516	0.646447	-1.589403
C	-0.459584	-0.716352	-1.997889
H	-0.094387	-0.274950	-2.933102
H	-0.099078	-1.738603	-1.935617
C	-1.380686	1.750796	-1.657346
H	-1.035808	1.896049	-2.689700
H	-1.766032	2.714570	-1.317315
C	-1.933350	-0.588553	-1.816096
H	-2.545835	-1.483644	-1.813647
C	-3.824866	0.936380	-1.246990
C	-4.616937	-0.027731	-0.594617
C	-4.409555	2.175121	-1.562487
C	-5.946725	0.236169	-0.278283
H	-4.176633	-0.979849	-0.315699
C	-5.742687	2.436850	-1.249358
H	-3.831008	2.932997	-2.083022
C	-6.515849	1.470146	-0.604334
H	-6.540568	-0.519150	0.229263
H	-6.178220	3.396249	-1.513013
H	-7.552590	1.676571	-0.355277

^{4}VIB$_{exo-p}$

Fe	0.959087	-0.981386	0.678366
N	2.592561	0.137086	0.842586
N	0.087054	0.118667	2.094323
O	0.174796	0.059806	-0.991068
C	1.851426	1.782167	2.513228
C	2.768958	1.274138	1.612697
C	4.626703	1.128467	0.355016
H	5.586117	1.276745	-0.122642
C	0.814933	2.407649	-1.010930
C	4.026364	-0.986151	-0.811907
C	0.592677	1.247918	2.721082
C	3.739210	0.038631	0.070909
C	4.031679	1.886171	1.318530
H	4.397541	2.788857	1.789074
C	-0.314879	1.429434	-0.796638
H	-0.649884	1.473539	0.240416
C	-1.226790	0.017413	2.528533
C	-0.406629	1.840763	3.560420
H	-0.252600	2.725666	4.163998
C	-1.537575	1.088918	3.428097
H	-2.499572	1.229107	3.903372
C	1.829901	2.163489	-1.941852
C	2.834735	3.108890	-2.148853
C	0.820448	3.605829	-0.287986
H	0.042914	3.792046	0.449879
C	1.821073	4.554294	-0.499491
H	1.814695	5.482294	0.065289
C	2.831161	4.307072	-1.431753
H	3.612943	5.043002	-1.596115
N	-0.544660	-2.288855	0.799683
N	1.984016	-2.298634	-0.412077
C	0.351435	-4.134416	-0.560704
C	-0.643323	-3.528720	0.188033
C	-2.653795	-3.180318	1.135893
H	-3.670889	-3.253295	1.498520
C	-2.113995	-0.982214	2.173211

C	1.572510	-3.549560	-0.847453
C	-1.778536	-2.074285	1.392802
C	-1.946548	-4.087352	0.401539
H	-2.267252	-5.052604	0.032050
C	3.220874	-2.095305	-1.002973
C	2.565889	-4.130296	-1.703048
H	2.483042	-5.106242	-2.163169
C	3.578802	-3.223458	-1.811672
H	4.499501	-3.306524	-2.374170
H	4.968801	-0.945503	-1.347192
H	0.147606	-5.114934	-0.977180
H	-3.121918	-0.930994	2.569669
H	2.117526	2.682696	3.055473
H	3.622203	2.908778	-2.870090
H	1.845641	1.222444	-2.482643
C	-2.536675	0.567385	-1.585201
C	-0.532798	-0.728245	-2.013759
H	-0.221996	-0.321311	-2.984397
H	-0.138114	-1.737379	-1.924911
C	-1.542859	1.708561	-1.689279
H	-1.207891	1.847751	-2.725960
H	-1.961497	2.662730	-1.361157
C	-2.007114	-0.652257	-1.792225
H	-2.588142	-1.568007	-1.759008
C	-3.944205	0.810242	-1.214972
C	-4.667746	-0.150103	-0.483551
C	-4.596153	2.000092	-1.581473
C	-6.000438	0.067261	-0.143918
H	-4.169174	-1.059364	-0.161304
C	-5.931645	2.215530	-1.243682
H	-4.067419	2.752873	-2.159624
C	-6.638570	1.251220	-0.523354
H	-6.542169	-0.683970	0.424484
H	-6.421154	3.136446	-1.547104
H	-7.677324	1.421776	-0.256037

^{6}VIB$_{exo\text{-}p}$

Fe	1.014081	-0.851008	0.541344
N	2.820487	0.129843	0.612061
N	0.368879	0.352456	2.083158
O	0.056563	0.040556	-1.044019
C	2.234840	1.959393	2.147199
C	3.087567	1.325171	1.251424
C	4.893057	0.910477	-0.035303
H	5.847743	0.940634	-0.544106
C	0.576541	2.414497	-1.045563
C	4.085437	-1.283325	-0.955222
C	0.975364	1.511227	2.534258
C	3.923037	-0.142252	-0.174887
C	4.377860	1.815322	0.847508
H	4.824733	2.735197	1.200725
C	-0.512819	1.389349	-0.850348
H	-0.861385	1.411690	0.183635
C	-0.880919	0.311829	2.677352
C	0.089522	2.205839	3.427143
H	0.323575	3.137461	3.925868
C	-1.059410	1.469494	3.509916
H	-1.948447	1.680503	4.089952
C	1.634779	2.203978	-1.935478
C	2.616152	3.181766	-2.100435
C	0.509897	3.612663	-0.325289
H	-0.299458	3.770048	0.384339
C	1.484399	4.594884	-0.499594
H	1.422986	5.522880	0.061715
C	2.541744	4.379441	-1.386379
H	3.305525	5.140362	-1.518256
N	-0.509492	-2.166666	1.018980
N	1.958031	-2.406267	-0.422531
C	0.174228	-4.103663	-0.336002
C	-0.727544	-3.417259	0.473808
C	-2.597786	-2.928040	1.637838
H	-3.567088	-2.941292	2.119428
C	-1.821093	-0.698976	2.496711
C	1.422644	-3.642089	-0.743574
C	-1.644544	-1.855965	1.744472
C	-2.029575	-3.894988	0.857194
H	-2.444494	-4.852734	0.570844
C	3.191144	-2.345616	-1.048404
C	2.335539	-4.361757	-1.590117
H	2.160244	-5.355934	-1.980339
C	3.423609	-3.559243	-1.783109
H	4.314777	-3.769136	-2.360238
H	5.013418	-1.371845	-1.511211
H	-0.124686	-5.087616	-0.683219
H	-2.767453	-0.589011	3.016519
H	2.574412	2.899148	2.569801
H	3.441080	3.006270	-2.784767
H	1.705010	1.265349	-2.475987

C	-2.694566	0.417526	-1.580363
C	-0.662433	-0.837235	-1.997385
H	-0.390526	-0.472579	-2.995058
H	-0.230050	-1.826170	-1.862479
C	-1.742441	1.585395	-1.757780
H	-1.410050	1.681688	-2.800155
H	-2.192969	2.540395	-1.477598
C	-2.127753	-0.794027	-1.729193
H	-2.674368	-1.724851	-1.620714
C	-4.099171	0.631455	-1.184790
C	-4.755855	-0.294513	-0.353163
C	-4.812183	1.758389	-1.628200
C	-6.087272	-0.105754	0.008288
H	-4.206669	-1.149715	0.030456
C	-6.146461	1.943311	-1.269225
H	-4.332591	2.481408	-2.282386
C	-6.788358	1.012908	-0.449704
H	-6.578269	-0.827373	0.655454
H	-6.685870	2.813146	-1.632677
H	-7.826151	1.160628	-0.165635

^{4}TS2B'$_{exo\text{-}p}$

Fe	-0.812569	0.917819	0.746549
N	-1.348140	-0.741661	1.752371
N	1.031243	0.754374	1.529656
O	-0.502350	-0.028237	-0.950954
C	0.843520	-1.445262	2.617662
C	-0.513155	-1.628302	2.406407
C	-2.534949	-2.614570	2.416121
H	-3.381153	-3.275799	2.548449
C	-0.888446	-2.359011	-1.331946
C	-3.740073	-0.814465	1.185885
C	1.555596	-0.326759	2.216824
C	-2.592959	-1.344245	1.749329
C	-1.246920	-2.790733	2.823579
H	-0.818005	-3.625449	3.362564
C	0.009884	-1.228010	-0.962141
H	0.711688	-1.468777	-0.155591
C	2.086454	1.635650	1.372935
C	2.953738	-0.124209	2.473317
H	3.590359	-0.829124	2.990726
C	3.281550	1.092056	1.953862
H	4.242646	1.588398	1.956478
C	-2.103294	-2.109779	-1.983871
C	-2.960668	-3.166037	-2.282469
C	-0.544087	-3.670019	-0.976346
H	0.390155	-3.859182	-0.451177
C	-1.404640	-4.724866	-1.272495
H	-1.138370	-5.738987	-0.989226
C	-2.612279	-4.474120	-1.930525
H	-3.282385	-5.296286	-2.164603
N	-0.373099	2.783276	0.160865
N	-2.747247	1.278432	0.358594
C	-2.553206	3.458388	-0.759257
C	-1.205941	3.659655	-0.511416
C	0.803333	4.671926	-0.471400
H	1.639271	5.352220	-0.571153
C	2.013724	2.870646	0.753484
C	-3.267849	2.348949	-0.341521
C	0.863746	3.399506	0.191060
C	-0.478242	4.830936	-0.911350
H	-0.909024	5.668165	-1.445022
C	-3.805993	0.411005	0.544729
C	-4.670944	2.154345	-0.583175
H	-5.306814	2.858588	-1.103625
C	-5.004368	0.953442	-0.033267
H	-5.970820	0.467109	-0.008611
H	-4.651556	-1.398441	1.254115
H	-3.088382	4.230108	-1.302243
H	2.918447	3.468189	0.709802
H	1.382016	-2.222921	3.148900
H	-3.903787	-2.971939	-2.785335
H	-2.368462	-1.085583	-2.225630
C	2.261970	-0.313935	-2.045472
C	0.843360	1.597954	-2.736985
H	0.073732	0.992057	-3.197954
H	0.656406	2.666810	-2.701610
C	1.308750	-1.275514	-2.428100
H	0.604833	-1.018085	-3.211569
H	1.612322	-2.316372	-2.455381
C	1.982661	1.092877	-2.213370
H	2.677538	1.793684	-1.765997
C	3.462041	-0.704824	-1.290128
C	4.597020	0.133608	-1.245739
C	3.515928	-1.924581	-0.581685
C	5.733256	-0.234426	-0.532197
H	4.611818	1.059902	-1.809959
C	4.643805	-2.279388	0.149996

H	2.662931	-2.593950	-0.580726
C	5.759020	-1.437778	0.176435
H	6.602661	0.416204	-0.533888
H	4.656133	-3.217825	0.696480
H	6.644044	-1.721744	0.738147

B-meta

^{4}TS1B$_{endo\text{-}m}$

Fe	-0.733984	-0.190166	0.408667
N	-1.303711	-0.279250	2.336483
N	-0.405799	-2.168523	0.408607
O	-2.898474	-0.672682	-0.105777
C	-0.919554	-2.669034	2.762326
C	-1.287993	-1.389764	3.150926
C	-1.978293	0.303164	4.465834
H	-2.322415	0.930529	5.277870
C	-4.920477	-0.379554	-1.321995
C	-1.879258	2.081259	2.708868
C	-0.514461	-3.024596	1.483189
C	-1.723876	0.766608	3.126615
C	-1.708534	-1.032393	4.480647
H	-1.786115	-1.726257	5.307709
C	-3.529915	-0.094377	-0.993361
H	-3.030490	0.690846	-1.585737
C	0.040144	-2.934485	-0.646713
C	-0.125920	-4.353475	1.094839
H	-0.125127	-5.211109	1.755092
C	0.217118	-4.298097	-0.224661
H	0.556403	-5.100725	-0.866671
C	-5.636987	-1.365256	-0.617918
C	-6.962053	-1.618636	-0.947307
C	-5.542290	0.344757	-2.353104
H	-4.981109	1.104753	-2.891969
C	-6.870904	0.087188	-2.679346
H	-7.356008	0.644213	-3.474937
C	-7.577612	-0.893153	-1.976464
H	-8.614449	-1.094677	-2.230511
N	-0.304047	-0.115409	-1.557204
N	-1.203183	1.769531	0.362831
C	-0.659484	2.281868	-1.980320
C	-0.293024	1.001100	-2.366829
C	0.453413	-0.681162	-3.664227
H	0.827284	-1.302203	-4.467873
C	0.310768	-2.468649	-1.925069
C	-1.076167	2.633758	-0.706173
C	0.154434	-1.155072	-2.339349
C	0.173987	0.653092	-3.681664
H	0.272972	1.351962	-4.502161
C	-1.637254	2.540309	1.422439
C	-1.438812	3.966521	-0.309182
H	-1.419168	4.827732	-0.964095
C	-1.786297	3.909144	1.007874
H	-2.115246	4.713248	1.653347
O	1.204073	0.170725	0.950321
C	2.143094	2.334290	0.528428
C	1.642770	1.285927	1.473716
H	1.037832	1.688655	2.299541
C	2.526468	1.974184	-0.768518
C	2.969726	2.948126	-1.661185
C	2.190583	3.676200	0.918566
H	1.873157	3.958681	1.920247
C	2.627590	4.652520	0.022052
H	2.658758	5.694195	0.328618
C	3.022618	4.289239	-1.267731
H	3.365427	5.048655	-1.964849
H	-2.217823	2.804351	3.443585
H	-0.592685	3.069727	-2.722662
H	0.675634	-3.184879	-2.653726
H	-0.955872	-3.452015	3.512620
H	-7.522077	-2.377368	-0.409083
H	-5.135956	-1.912036	0.174642
H	2.450150	0.934236	-1.067481
H	3.262389	2.662749	-2.668115
C	3.811107	-0.954480	1.056099
C	4.023513	0.241331	1.781730
C	3.071896	0.815573	2.642971
H	2.463959	0.158555	3.260915
H	3.356911	1.739189	3.141456
C	2.665001	-1.678126	1.306958
H	2.105499	-1.602644	2.228348
H	2.341564	-2.453477	0.622165
C	4.692451	-1.317713	-0.087010
C	5.233487	-0.339197	-0.937740
C	4.983472	-2.667483	-0.343053
C	6.040933	-0.703698	-2.014009
H	4.994566	0.708556	-0.781137
C	5.788879	-3.030925	-1.421257
H	4.596922	-3.434000	0.323737

C	6.320919	-2.049579	-2.259940
H	6.446096	0.065239	-2.665764
H	6.011811	-4.079270	-1.598216
H	6.952616	-2.331449	-3.097366
H	4.869288	0.858052	1.486022

^{2}IIB'$_{endo\text{-}m}$

Fe	-0.830162	0.048271	0.015373
N	-0.730747	-0.291216	1.987827
N	-0.499286	-1.890515	-0.365217
O	-2.738866	-0.367806	0.104029
C	-0.361509	-2.721861	1.941422
C	-0.544479	-1.512954	2.594047
C	-0.803269	-0.046600	4.285212
H	-0.899744	0.446478	5.243807
C	-5.048651	-0.103555	-0.313344
C	-1.175777	1.967937	2.851889
C	-0.341730	-2.889387	0.565550
C	-0.909153	0.615393	3.011886
C	-0.582110	-1.366120	4.026464
H	-0.457280	-2.179335	4.729875
C	-3.656280	0.263436	-0.448785
H	-3.410343	1.136507	-1.065686
C	-0.410213	-2.502395	-1.596094
C	-0.138768	-4.155132	-0.093698
H	0.015089	-5.095995	0.418711
C	-0.178105	-3.914611	-1.433413
H	-0.063802	-4.616336	-2.249263
C	-5.434832	-1.211787	0.467705
C	-6.779857	-1.533846	0.579840
C	-6.019139	0.671474	-0.975526
H	-5.711470	1.524547	-1.575335
C	-7.365556	0.342201	-0.858243
H	-8.119209	0.935693	-1.365974
C	-7.742587	-0.758091	-0.081928
H	-8.794019	-1.015145	0.009798
N	-0.941995	0.399128	-1.960480
N	-1.205636	1.993809	0.395273
C	-1.328493	2.829305	-1.915464
C	-1.119679	1.622245	-2.568688
C	-0.842654	0.156883	-4.256505
H	-0.739373	-0.336812	-5.214090
C	-0.520205	-1.863137	-2.821004
C	-1.370949	2.995467	-0.538129
C	-0.758881	-0.507144	-2.982914
C	-1.066167	1.476024	-4.000768
H	-1.182994	2.289479	-4.705216
C	-1.317267	2.604813	1.627892
C	-1.599727	4.254242	0.122454
H	-1.760715	5.194733	-0.388588
C	-1.566977	4.011864	1.463025
H	-1.696585	4.712055	2.278130
O	1.077571	0.440832	-0.172904
C	2.953113	1.837856	0.152226
C	1.693926	1.251681	0.547720
H	1.257669	1.562999	1.502386
C	3.544997	1.515151	-1.083448
C	4.745176	2.113854	-1.442683
C	3.570327	2.761599	1.018313
H	3.107023	2.994893	1.973409
C	4.770221	3.356871	0.651133
H	5.253087	4.068866	1.313112
C	5.355366	3.031429	-0.579346
H	6.295613	3.495101	-0.864468
H	-1.288386	2.568518	3.748518
H	-1.464458	3.713263	-2.529944
H	-0.413506	-2.466852	-3.716311
H	-0.223966	-3.605742	2.555074
H	-7.088227	-2.385076	1.178832
H	-4.670069	-1.795614	0.969774
H	3.066684	0.785200	-1.726221
H	5.214281	1.856553	-2.386473
C	3.541244	-1.747348	1.495486
C	3.779977	-0.555620	2.337691
C	2.973929	-0.123405	3.317343
H	2.048390	-0.631901	3.571905
H	3.225146	0.756971	3.902853
C	2.929212	-2.846252	1.969009
H	2.615828	-2.918760	3.005689
H	2.751495	-3.709179	1.335247
C	3.999387	-1.681695	0.076984
C	5.308325	-1.298707	-0.254104
C	3.111992	-2.004471	-0.961123
C	5.720801	-1.252073	-1.585834
H	6.016241	-1.055872	0.533796
C	3.520460	-1.948206	-2.293417
H	2.092721	-2.274016	-0.715932
C	4.827549	-1.571372	-2.611725

H	6.742859	-0.968859	-1.822285
H	2.811838	-2.192049	-3.080672
H	5.149587	-1.531379	-3.648702
H	4.660881	0.035661	2.091093

^{4}IIB'$_{endo\text{-}m}$

Fe	-0.815567	-0.011844	0.054370
N	-0.697520	-0.371148	2.018678
N	-0.519320	-1.948518	-0.327976
O	-3.000500	-0.430882	0.170644
C	-0.342676	-2.804204	1.970220
C	-0.500575	-1.594241	2.626050
C	-0.691430	-0.117640	4.314736
H	-0.751450	0.378984	5.274468
C	-5.289536	-0.014173	-0.312251
C	-1.084393	1.897168	2.895230
C	-0.351648	-2.961285	0.594236
C	-0.830477	0.542270	3.045607
C	-0.490861	-1.441625	4.054616
H	-0.351628	-2.252698	4.757551
C	-3.862084	0.251048	-0.394464
H	-3.548981	1.116820	-1.001102
C	-0.449595	-2.552021	-1.567550
C	-0.166135	-4.217065	-0.078808
H	-0.009660	-5.163974	0.421280
C	-0.223010	-3.963392	-1.417411
H	-0.123307	-4.659685	-2.239795
C	-5.777129	-1.100285	0.439573
C	-7.144580	-1.331709	0.504009
C	-6.181793	0.831787	-0.993892
H	-5.796497	1.668457	-1.572074
C	-7.551611	0.595330	-0.925453
H	-8.245267	1.245299	-1.449624
C	-8.029771	-0.484854	-0.177470
H	-9.098862	-0.669976	-0.123807
N	-0.964025	0.358337	-1.914545
N	-1.179320	1.934416	0.437794
C	-1.354655	2.788102	-1.863796
C	-1.158665	1.582921	-2.521425
C	-0.914889	0.115335	-4.210949
H	-0.831142	-0.377744	-5.170721
C	-0.570999	-1.906641	-2.787770
C	-1.365275	2.945060	-0.485551
C	-0.804526	-0.549025	-2.941469
C	-1.134658	1.435515	-3.951121
H	-1.266846	2.247805	-4.654036
C	-1.252754	2.538906	1.677480
C	-1.568498	4.197731	0.188042
H	-1.738691	5.142641	-0.311567
C	-1.499277	3.946169	1.527044
H	-1.601953	4.642643	2.349113
O	1.302536	0.474489	-0.156244
C	3.150197	1.954375	0.035592
C	1.887766	1.372955	0.461443
H	1.429012	1.788788	1.370857
C	3.796730	1.496062	-1.125690
C	5.001612	2.071536	-1.510395
C	3.717439	2.987178	0.803387
H	3.211889	3.327579	1.704025
C	4.923179	3.558906	0.413042
H	5.367286	4.355471	1.002058
C	5.563103	3.099606	-0.744365
H	6.506786	3.544215	-1.048079
H	-1.164452	2.496771	3.795774
H	-1.503752	3.674203	-2.471712
H	-0.480713	-2.507583	-3.686464
H	-0.196205	-3.690828	2.577343
H	-7.529592	-2.166945	1.081032
H	-5.069607	-1.739806	0.957689
H	3.355837	0.683117	-1.691796
H	5.511499	1.710313	-2.397702
C	3.569219	-1.677084	1.515318
C	3.847006	-0.444173	2.283046
C	3.057999	0.065566	3.238651
H	2.120756	-0.404453	3.524062
H	3.333686	0.972808	3.769246
C	2.940484	-2.732221	2.060826
H	2.639069	-2.736249	3.103594
H	2.737497	-3.627423	1.481684
C	4.010534	-1.706934	0.090354
C	5.326324	-1.385327	-0.276274
C	3.099524	-2.055917	-0.918107
C	5.722283	-1.423531	-1.613426
H	6.051288	-1.121038	0.488875
C	3.491048	-2.083384	-2.255960
H	2.074506	-2.273224	-0.646450
C	4.805317	-1.767221	-2.609790
H	6.749343	-1.185951	-1.876826

H	2.764182	-2.343480	-3.021107
H	5.113670	-1.791355	-3.651365
H	4.742439	0.106535	1.998418

^{6}IIB'$_{endo-m}$

Fe	-0.763324	0.094296	0.191402
N	-0.832440	1.456244	1.731722
N	-0.686039	-1.441172	1.532414
O	-2.946544	-0.012282	0.322883
C	-0.673666	-0.122254	3.614017
C	-0.761564	1.164093	3.074418
C	-0.951720	3.412337	2.931188
H	-1.037053	4.471891	3.135742
C	-5.142872	-0.074802	-0.575026
C	-1.061901	3.524800	0.411360
C	-0.661968	-1.324187	2.905200
C	-0.961189	2.823949	1.615304
C	-0.826531	2.389345	3.830335
H	-0.789934	2.452755	4.910311
C	-3.697886	0.034264	-0.659918
H	-3.260187	0.164133	-1.661861
C	-0.713903	-2.786792	1.237855
C	-0.658446	-2.640525	3.492874
H	-0.637851	-2.842609	4.556099
C	-0.695200	-3.541373	2.465963
H	-0.708720	-4.621953	2.527688
C	-5.781120	-0.244044	0.669185
C	-7.164493	-0.346337	0.721740
C	-5.901431	-0.009700	-1.757554
H	-5.399667	0.121353	-2.713403
C	-7.287831	-0.113083	-1.698334
H	-7.878943	-0.063686	-2.607408
C	-7.915685	-0.280839	-0.460214
H	-8.998013	-0.361460	-0.413521
N	-0.795517	-1.251729	-1.366380
N	-0.984068	1.645056	-1.178313
C	-0.833088	0.332169	-3.254213
C	-0.772061	-0.953194	-2.711702
C	-0.712234	-3.209150	-2.567861
H	-0.678394	-4.271924	-2.770522
C	-0.744640	-3.330705	-0.046676
C	-0.931219	1.535008	-2.547852
C	-0.760073	-2.623048	-1.251589
C	-0.713125	-2.179331	-3.467846
H	-0.684259	-2.238992	-4.548339
C	-1.057183	2.985143	-0.878249
C	-0.997217	2.851349	-3.134122
H	-0.978830	3.057872	-4.196554
C	-1.078613	3.745972	-2.103938
H	-1.138269	4.825254	-2.161619
O	1.309705	0.404670	-0.080004
C	3.233844	1.776398	-0.274707
C	1.991554	1.353975	0.339361
H	1.626027	1.932327	1.198075
C	3.735126	1.120362	-1.414064
C	4.923795	1.556513	-1.985273
C	3.929188	2.865734	0.282672
H	3.534562	3.359293	1.167501
C	5.118099	3.296129	-0.293890
H	5.661097	4.134303	0.131655
C	5.612822	2.640223	-1.427886
H	6.543399	2.975119	-1.877664
H	-1.136151	4.605538	0.484482
H	-0.806502	0.404596	-4.337193
H	-0.736351	-4.413862	-0.118041
H	-0.644496	-0.195095	4.696904
H	-7.665767	-0.477082	1.675853
H	-5.175293	-0.292137	1.568401
H	3.195640	0.270815	-1.817184
H	5.322199	1.045040	-2.855513
C	3.394430	-1.620164	1.662929
C	3.849832	-0.358420	2.285644
C	3.148063	0.358820	3.174375
H	2.156233	0.054982	3.499226
H	3.549800	1.271669	3.605462
C	2.643714	-2.509287	2.335381
H	2.365050	-2.350784	3.371779
H	2.304207	-3.429403	1.871580
C	3.791710	-1.871979	0.247368
C	5.115634	-1.705216	-0.187451
C	2.827726	-2.282146	-0.687233
C	5.465441	-1.950611	-1.515710
H	5.884041	-1.401166	0.518036
C	3.173233	-2.518730	-2.017025
H	1.797848	-2.382887	-0.368181
C	4.495822	-2.352561	-2.437340
H	6.498502	-1.829373	-1.829975
H	2.404445	-2.822696	-2.722732

H	4.769675	-2.538417	-3.472279
H	4.810514	0.028377	1.948610

[2]TS1B'$_{endo\text{-}m}$

Fe	-0.772195	-0.008513	0.418064
N	-1.349961	1.125470	1.963216
N	-0.815850	-1.652006	1.573940
O	-2.687293	-0.347862	0.027730
C	-1.278716	-0.577980	3.734326
C	-1.467762	0.717736	3.271361
C	-2.014373	2.904456	3.278056
H	-2.312620	3.912854	3.534289
C	-4.623165	-0.553106	-1.315441
C	-1.623188	3.289147	0.833013
C	-1.013229	-1.681545	2.933523
C	-1.677830	2.463382	1.948309
C	-1.870902	1.827304	4.099571
H	-2.034948	1.768618	5.167906
C	-3.214622	-0.296503	-1.094589
H	-2.595913	-0.046481	-1.966035
C	-0.652402	-2.961015	1.182725
C	-0.952076	-3.041103	3.413302
H	-1.073065	-3.334358	4.448214
C	-0.745168	-3.834927	2.325906
H	-0.653092	-4.912651	2.285731
C	-5.478826	-0.875294	-0.243329
C	-6.823449	-1.116180	-0.489196
C	-5.127198	-0.476176	-2.626658
H	-4.459161	-0.226827	-3.447558
C	-6.476113	-0.719378	-2.865791
H	-6.870843	-0.661567	-3.875263
C	-7.320458	-1.038491	-1.797946
H	-8.373695	-1.228298	-1.984285
N	-0.251353	-1.162793	-1.148841
N	-0.818115	1.611802	-0.767686
C	0.081311	0.606330	-2.822680
C	0.123530	-0.714731	-2.394554
C	0.335773	-2.957697	-2.478210
H	0.511127	-3.987502	-2.761751
C	-0.350989	-3.382568	-0.105139
C	-0.374795	1.679203	-2.068716
C	-0.125147	-2.532655	-1.179931
C	0.505294	-1.830048	-3.225388
H	0.840764	-1.745900	-4.251117
C	-1.189595	2.892393	-0.424164
C	-0.479044	3.031883	-2.555663
H	-0.186567	3.350920	-3.547481
C	-0.995256	3.780343	-1.542057
H	-1.213055	4.840337	-1.530085
O	1.057099	0.277797	0.764512
C	2.284441	2.227991	0.070948
C	1.575244	1.436918	1.127071
H	0.937233	2.052357	1.771547
C	2.854061	1.590354	-1.036981
C	3.502088	2.340996	-2.014709
C	2.357867	3.619855	0.185290
H	1.899863	4.116543	1.038106
C	3.002614	4.371903	-0.798283
H	3.053442	5.453037	-0.706313
C	3.579105	3.732979	-1.897408
H	4.085468	4.316459	-2.661091
H	-1.893694	4.331616	0.965831
H	0.407769	0.813635	-3.836516
H	-0.232501	-4.448358	-0.272062
H	-1.408799	-0.751752	4.797737
H	-7.490972	-1.364872	0.330058
H	-5.069019	-0.929641	0.760147
H	2.771851	0.513214	-1.130354
H	3.945584	1.840776	-2.871216
C	3.482585	-1.052632	1.362467
C	3.744289	0.262362	1.827655
C	2.801104	1.067687	2.493284
H	2.093599	0.599780	3.174355
H	3.143646	2.045965	2.822654
C	2.339986	-1.687730	1.770466
H	1.738006	-1.345012	2.600085
H	2.024549	-2.616286	1.309248
C	4.312763	-1.606619	0.255824
C	5.712114	-1.593946	0.317662
C	3.680776	-2.109437	-0.892309
C	6.467735	-2.088890	-0.745759
H	6.211357	-1.214564	1.206092
C	4.438486	-2.598788	-1.954860
H	2.597423	-2.087231	-0.965330
C	5.833400	-2.592057	-1.883766
H	7.552035	-2.084577	-0.683265
H	3.934880	-2.974834	-2.841054
H	6.424156	-2.973236	-2.711648

H	4.619208	0.760013	1.415059

^{4}TS1B'$_{endo-m}$

Fe	-0.714833	0.108380	0.517000
N	-1.245462	1.655020	1.682554
N	-0.893263	-1.136051	2.079782
O	-2.936069	-0.236108	0.155012
C	-1.344753	0.507840	3.853742
C	-1.426332	1.634101	3.048021
C	-1.749830	3.777312	2.436075
H	-1.949544	4.840318	2.396860
C	-4.801371	-0.714430	-1.241163
C	-1.291043	3.445971	-0.000851
C	-1.120186	-0.781266	3.392903
C	-1.434004	2.962718	1.292348
C	-1.736425	2.956146	3.524262
H	-1.927352	3.207198	4.559617
C	-3.396967	-0.437871	-0.971328
H	-2.719296	-0.413811	-1.841395
C	-0.785963	-2.510306	2.070499
C	-1.136215	-1.954842	4.224823
H	-1.293986	-1.946717	5.295543
C	-0.941346	-3.026212	3.404467
H	-0.900316	-4.075757	3.665617
C	-5.738265	-0.766302	-0.192139
C	-7.071078	-1.035752	-0.474091
C	-5.212047	-0.933636	-2.567155
H	-4.481188	-0.891614	-3.371522
C	-6.549207	-1.203508	-2.844497
H	-6.871691	-1.373877	-3.867008
C	-7.475255	-1.254018	-1.798340
H	-8.518962	-1.464593	-2.014021
N	-0.321435	-1.462114	-0.679337
N	-0.686107	1.323359	-1.085917
C	0.043612	-0.275933	-2.804411
C	0.005792	-1.419917	-2.019890
C	0.071918	-3.600698	-1.456049
H	0.168199	-4.678531	-1.437652
C	-0.518857	-3.290907	0.954581
C	-0.285314	0.997203	-2.364259
C	-0.285142	-2.794188	-0.319831
C	0.262123	-2.749233	-2.505288
H	0.538645	-2.987991	-3.524167
C	-0.917972	2.682574	-1.096396
C	-0.274516	2.172801	-3.193068
H	0.012009	2.184865	-4.236364
C	-0.672385	3.214407	-2.410576
H	-0.778944	4.256887	-2.680117
O	1.273041	0.480447	0.864984
C	2.445911	2.217423	-0.313977
C	1.862993	1.646713	0.941297
H	1.347847	2.389168	1.566534
C	2.854693	1.374791	-1.353910
C	3.392461	1.915611	-2.519670
C	2.569514	3.603580	-0.457077
H	2.240982	4.259641	0.346563
C	3.101840	4.145155	-1.627929
H	3.193085	5.222527	-1.733539
C	3.517175	3.301400	-2.660220
H	3.936522	3.721345	-3.570124
H	-1.450569	4.507111	-0.160544
H	0.334402	-0.388353	-3.843521
H	-0.460719	-4.365299	1.094050
H	-1.509088	0.638823	4.918233
H	-7.800548	-1.077690	0.329052
H	-5.397931	-0.593583	0.823956
H	2.742116	0.301984	-1.242252
H	3.713144	1.255495	-3.321041
C	3.633865	-0.854270	1.373784
C	4.098374	0.463308	1.586783
C	3.317575	1.462556	2.192867
H	2.682582	1.198293	3.035617
H	3.752451	2.456249	2.268226
C	2.421984	-1.215157	1.919247
H	1.988332	-0.716717	2.774290
H	1.918318	-2.121659	1.605206
C	4.288356	-1.706449	0.342436
C	5.680539	-1.861679	0.313023
C	3.510310	-2.340965	-0.638565
C	6.282876	-2.644661	-0.672548
H	6.292480	-1.384456	1.074561
C	4.114174	-3.120401	-1.623237
H	2.434143	-2.200301	-0.647311
C	5.502135	-3.276503	-1.642663
H	7.362366	-2.764930	-0.678634
H	3.496745	-3.598638	-2.378667
H	5.973237	-3.884902	-2.409237
H	4.963812	0.780385	1.009465

^{6}**TS1B'$_{endo\text{-}m}$**

Fe	-0.672197	0.081675	0.501810
N	-1.327002	1.597449	1.740045
N	-0.939155	-1.287670	2.018864
O	-2.860909	-0.258665	0.096546
C	-1.538663	0.291698	3.814476
C	-1.584754	1.482814	3.084228
C	-1.861675	3.668544	2.574106
H	-2.056732	4.733179	2.593227
C	-4.735982	-0.658536	-1.306430
C	-1.282749	3.448122	0.120483
C	-1.261245	-0.986770	3.322582
C	-1.486802	2.918013	1.398429
C	-1.919967	2.783773	3.613883
H	-2.172207	2.985739	4.647060
C	-3.329091	-0.400777	-1.037002
H	-2.657730	-0.332517	-1.908093
C	-0.801933	-2.652507	1.933639
C	-1.303558	-2.206971	4.092779
H	-1.531508	-2.264174	5.149480
C	-1.026437	-3.234318	3.235497
H	-0.980451	-4.292990	3.456794
C	-5.662882	-0.762155	-0.252128
C	-6.999586	-1.010016	-0.534840
C	-5.160530	-0.804729	-2.638297
H	-4.437319	-0.722886	-3.446433
C	-6.501697	-1.052964	-2.916129
H	-6.835073	-1.166599	-3.943001
C	-7.417696	-1.155033	-1.864866
H	-8.464568	-1.348721	-2.081046
N	-0.278966	-1.476538	-0.807742
N	-0.684409	1.394111	-1.101827
C	0.069127	-0.155055	-2.858510
C	0.050409	-1.353569	-2.139250
C	0.165463	-3.562323	-1.664310
H	0.288971	-4.637192	-1.699808
C	-0.470640	-3.357090	0.772600
C	-0.262890	1.115103	-2.380080
C	-0.220403	-2.814918	-0.491204
C	0.338960	-2.660952	-2.677927
H	0.624330	-2.858453	-3.703415
C	-0.894433	2.751351	-1.026314
C	-0.219594	2.339008	-3.144257
H	0.084057	2.410641	-4.180523
C	-0.610804	3.346863	-2.310806
H	-0.690201	4.402992	-2.533375
O	1.222277	0.469003	0.898604
C	2.439701	2.249473	-0.165252
C	1.824297	1.628005	1.049589
H	1.282842	2.343520	1.683378
C	2.858786	1.451632	-1.235916
C	3.427366	2.041346	-2.362425
C	2.585331	3.638927	-0.235477
H	2.247955	4.259671	0.592051
C	3.149988	4.229009	-1.367011
H	3.258152	5.308677	-1.417591
C	3.574730	3.430467	-2.430792
H	4.018365	3.888293	-3.310324
H	-1.421697	4.519466	0.011878
H	0.368285	-0.218382	-3.900278
H	-0.391958	-4.436268	0.863150
H	-1.774876	0.362243	4.871996
H	-7.721468	-1.091535	0.272096
H	-5.311860	-0.645377	0.768264
H	2.728873	0.376181	-1.181189
H	3.753369	1.417041	-3.189693
C	3.592392	-0.922046	1.414381
C	4.012360	0.403461	1.687469
C	3.203353	1.363860	2.321638
H	2.551803	1.048256	3.133546
H	3.636329	2.350535	2.468678
C	2.407663	-1.364878	1.949100
H	1.919392	-0.885930	2.786211
H	1.951165	-2.287007	1.607041
C	4.287196	-1.700045	0.350602
C	5.682962	-1.819536	0.336003
C	3.537657	-2.294498	-0.676206
C	6.317811	-2.532881	-0.681417
H	6.272443	-1.371398	1.132294
C	4.174585	-3.003722	-1.692755
H	2.458828	-2.176885	-0.693940
C	5.565925	-3.126903	-1.697372
H	7.399718	-2.628367	-0.677065
H	3.579604	-3.452032	-2.483464
H	6.062754	-3.680216	-2.489008
H	4.880992	0.763113	1.140399

^{2}IIIB'$_{endo\text{-}m}$

Fe	-0.719959	-0.071461	0.417975
N	-1.298124	1.029067	1.986443
N	-0.697740	-1.720287	1.559767
O	-2.607185	-0.447821	0.072535
C	-1.135754	-0.681692	3.743348
C	-1.372987	0.609773	3.294821
C	-2.027241	2.766115	3.323313
H	-2.370334	3.757103	3.590704
C	-4.571131	-0.721822	-1.209618
C	-1.724183	3.175006	0.871201
C	-0.864577	-1.768863	2.924493
C	-1.701223	2.347617	1.985223
C	-1.808263	1.695317	4.136700
H	-1.942979	1.624614	5.208295
C	-3.168164	-0.419103	-1.037187
H	-2.582928	-0.154479	-1.926205
C	-0.513539	-3.024195	1.152137
C	-0.768981	-3.130931	3.385849
H	-0.860760	-3.436589	4.420044
C	-0.568515	-3.909366	2.286208
H	-0.453644	-4.984145	2.232653
C	-5.381272	-1.063571	-0.107493
C	-6.723817	-1.350364	-0.308655
C	-5.118675	-0.671811	-2.505538
H	-4.485920	-0.407354	-3.349255
C	-6.465330	-0.961540	-2.699008
H	-6.894045	-0.925334	-3.695443
C	-7.263899	-1.299588	-1.601887
H	-8.315581	-1.525575	-1.753137
N	-0.189269	-1.189475	-1.162763
N	-0.839266	1.549227	-0.743707
C	0.034847	0.597328	-2.835708
C	0.136578	-0.722810	-2.417813
C	0.389999	-2.960868	-2.527164
H	0.579278	-3.983534	-2.826488
C	-0.230577	-3.426640	-0.145308
C	-0.449226	1.641570	-2.061337
C	-0.045084	-2.561871	-1.215174
C	0.517213	-1.822063	-3.267095
H	0.823913	-1.721658	-4.300264
C	-1.294628	2.803168	-0.393159
C	-0.657276	2.982311	-2.543983
H	-0.413160	3.318406	-3.542955
C	-1.194569	3.696238	-1.516793
H	-1.480563	4.739581	-1.498329
O	1.204216	0.284292	0.798150
C	2.077720	2.466324	0.065163
C	1.692921	1.599471	1.244218
H	0.853523	2.047002	1.773790
C	2.649531	1.946717	-1.100855
C	3.069746	2.802170	-2.119927
C	1.923639	3.852653	0.188706
H	1.466376	4.263921	1.086277
C	2.349841	4.708685	-0.826309
H	2.226610	5.782277	-0.714814
C	2.927388	4.184234	-1.984320
H	3.260634	4.848482	-2.776676
H	-2.057186	4.198280	1.009538
H	0.320224	0.823432	-3.857638
H	-0.089322	-4.487223	-0.324896
H	-1.233297	-0.868218	4.807816
H	-7.356652	-1.614333	0.532927
H	-4.938909	-1.096762	0.882949
H	2.755341	0.874978	-1.218984
H	3.512993	2.385109	-3.020086
C	3.530502	-0.550692	0.939422
C	3.931024	0.565896	1.568463
C	2.867084	1.397108	2.229529
H	2.480254	0.912259	3.138683
H	3.235169	2.383947	2.521600
C	2.069798	-0.877130	1.077589
H	1.835577	-1.206261	2.095116
H	1.744664	-1.646342	0.388415
C	4.391840	-1.427745	0.119901
C	5.721405	-1.682287	0.494957
C	3.909613	-1.994119	-1.074013
C	6.547479	-2.469959	-0.304204
H	6.097527	-1.275246	1.429353
C	4.738981	-2.778765	-1.873574
H	2.891027	-1.802353	-1.400944
C	6.059817	-3.021217	-1.491880
H	7.570978	-2.661894	0.005064
H	4.351213	-3.198800	-2.797726
H	6.703605	-3.638574	-2.111675
H	4.951508	0.929659	1.501862

^{4}IIIB'$_{endo\text{-}m}$

Fe	-0.710747	-0.019010	0.498258
N	-1.196362	1.330356	1.887027
N	-0.641152	-1.445946	1.897881
O	-2.868510	-0.479617	0.229217
C	-1.021024	-0.070597	3.901398
C	-1.245560	1.133319	3.252885
C	-1.800966	3.293378	2.941650
H	-2.090597	4.330140	3.053455
C	-4.806159	-0.868630	-1.091068
C	-1.569008	3.292301	0.453478
C	-0.769978	-1.274769	3.261343
C	-1.536953	2.652394	1.682951
C	-1.607737	2.356608	3.914045
H	-1.711800	2.467408	4.985580
C	-3.398386	-0.572457	-0.883795
H	-2.780805	-0.423430	-1.784747
C	-0.451821	-2.799904	1.703921
C	-0.646887	-2.539599	3.931819
H	-0.706876	-2.674564	5.003912
C	-0.464239	-3.485546	2.966633
H	-0.337334	-4.553657	3.086044
C	-5.668250	-1.071413	0.003774
C	-7.009176	-1.351910	-0.221454
C	-5.299720	-0.949618	-2.405211
H	-4.626704	-0.791161	-3.244685
C	-6.644761	-1.231220	-2.624909
H	-7.031618	-1.294819	-3.637162
C	-7.495829	-1.431535	-1.533740
H	-8.545894	-1.651113	-1.704768
N	-0.241493	-1.380615	-0.902296
N	-0.836387	1.383977	-0.915559
C	-0.085497	0.088308	-2.868529
C	0.016796	-1.143338	-2.238414
C	0.266162	-3.366020	-1.968352
H	0.442655	-4.426163	-2.095421
C	-0.218413	-3.414645	0.482981
C	-0.502408	1.255627	-2.250250
C	-0.094336	-2.744351	-0.724757
C	0.347866	-2.373552	-2.902269
H	0.597764	-2.456362	-3.952057
C	-1.213697	2.702012	-0.748027
C	-0.676526	2.511753	-2.925146
H	-0.467246	2.678030	-3.973438
C	-1.127348	3.403121	-1.998874

H	-1.360706	4.451172	-2.131022
O	1.450612	0.399010	0.810587
C	2.158924	2.529865	-0.144058
C	1.968649	1.723159	1.123954
H	1.208280	2.199765	1.749129
C	2.588380	1.943469	-1.339509
C	2.845869	2.736139	-2.458933
C	1.976246	3.916957	-0.093235
H	1.627309	4.377956	0.828648
C	2.237970	4.710742	-1.210099
H	2.096918	5.786618	-1.154255
C	2.678040	4.120672	-2.396809
H	2.884605	4.735923	-3.268039
H	-1.844910	4.341148	0.437387
H	0.149330	0.134775	-3.926526
H	-0.076275	-4.489993	0.479131
H	-1.092177	-0.080849	4.983830
H	-7.681444	-1.509637	0.616349
H	-5.265301	-1.003897	1.009290
H	2.713139	0.868156	-1.393401
H	3.182649	2.269189	-3.380646
C	3.732592	-0.506200	0.815220
C	4.229862	0.670036	1.232510
C	3.284342	1.603974	1.936004
H	3.036492	1.231649	2.941633
H	3.704496	2.605395	2.058633
C	2.288158	-0.740204	1.167915
H	2.168765	-0.921844	2.244192
H	1.857495	-1.584919	0.640409
C	4.465733	-1.510837	0.018743
C	5.827546	-1.763375	0.253576
C	3.821756	-2.213471	-1.015724
C	6.525972	-2.683045	-0.526424
H	6.330565	-1.248741	1.067468
C	4.522458	-3.131004	-1.796395
H	2.774701	-2.024738	-1.235422
C	5.876425	-3.371218	-1.554350
H	7.576943	-2.870538	-0.325315
H	4.008510	-3.656497	-2.596974
H	6.420863	-4.091420	-2.158261
H	5.238170	0.989474	0.987723

^{6}**IIIB'**$_{\mathbf{endo\text{-}m}}$

Fe	-0.711148	-0.012238	0.488025

N	-1.259314	1.270145	1.986951
N	-0.697502	-1.583261	1.814371
O	-2.819835	-0.442053	0.161300
C	-1.209487	-0.319640	3.867526
C	-1.389274	0.952432	3.321330
C	-1.886434	3.151274	3.147167
H	-2.164343	4.180665	3.333634
C	-4.747344	-0.797495	-1.176536
C	-1.527892	3.320300	0.652027
C	-0.911719	-1.494186	3.172387
C	-1.563977	2.606642	1.852404
C	-1.772808	2.133150	4.053092
H	-1.941971	2.170909	5.121562
C	-3.345332	-0.495205	-0.959189
H	-2.725463	-0.304557	-1.848908
C	-0.482068	-2.911777	1.522172
C	-0.813070	-2.808294	3.755666
H	-0.934777	-3.028633	4.808383
C	-0.555494	-3.683343	2.737278
H	-0.422974	-4.755905	2.797871
C	-5.607324	-1.049995	-0.089562
C	-6.944347	-1.338183	-0.325900
C	-5.239359	-0.837209	-2.493818
H	-4.567981	-0.640933	-3.326456
C	-6.580533	-1.127313	-2.724028
H	-6.966502	-1.159665	-3.738032
C	-7.429173	-1.376807	-1.640919
H	-8.476385	-1.603086	-1.820441
N	-0.223636	-1.321833	-1.037580
N	-0.826783	1.516275	-0.873110
C	-0.080930	0.302555	-2.883183
C	0.034496	-0.983217	-2.348224
C	0.328154	-3.221439	-2.208868
H	0.528965	-4.266369	-2.407550
C	-0.192808	-3.420373	0.253821
C	-0.484857	1.454479	-2.206893
C	-0.055648	-2.686394	-0.926948
C	0.391445	-2.170664	-3.083064
H	0.648510	-2.193915	-4.134336
C	-1.171070	2.823143	-0.601910
C	-0.623820	2.762998	-2.793500
H	-0.406167	3.003242	-3.825786
C	-1.052504	3.604431	-1.806716
H	-1.252797	4.665469	-1.876764

O	1.388198	0.367087	0.842024
C	2.194653	2.485174	-0.082622
C	1.917711	1.687834	1.175031
H	1.131540	2.176377	1.756085
C	2.638012	1.884266	-1.265325
C	2.972033	2.668744	-2.370177
C	2.077376	3.879431	-0.028787
H	1.718009	4.352620	0.882799
C	2.417673	4.664497	-1.130088
H	2.326786	5.745597	-1.072286
C	2.869720	4.059295	-2.304790
H	3.136346	4.668055	-3.164155
H	-1.776272	4.375705	0.704355
H	0.157093	0.415361	-3.936276
H	-0.028572	-4.491071	0.183475
H	-1.350300	-0.412459	4.940015
H	-7.615119	-1.533937	0.504986
H	-5.205717	-1.014272	0.918058
H	2.713790	0.804754	-1.323995
H	3.317600	2.189877	-3.282372
C	3.674653	-0.542306	0.893350
C	4.157519	0.616442	1.371722
C	3.183247	1.535559	2.055572
H	2.878193	1.137411	3.035134
H	3.602312	2.530100	2.227717
C	2.219824	-0.789971	1.183078
H	2.057394	-1.006464	2.246006
H	1.809574	-1.612952	0.607981
C	4.434017	-1.517792	0.085021
C	5.784536	-1.787427	0.361108
C	3.827494	-2.169685	-1.003909
C	6.509004	-2.675434	-0.431747
H	6.257750	-1.311756	1.215586
C	4.554642	-3.055065	-1.797395
H	2.790318	-1.966007	-1.255439
C	5.897202	-3.313256	-1.513933
H	7.550681	-2.877478	-0.198965
H	4.070070	-3.540869	-2.640155
H	6.462141	-4.008532	-2.128109
H	5.175031	0.940318	1.176396

^{2}IIB$_{exo\text{-}m}$

Fe	0.926287	-0.603730	0.101349
N	1.320636	-2.125971	-1.152584

N	1.454874	-1.731460	1.676068
O	2.792408	-0.068165	-0.056797
C	2.050253	-3.800641	0.491445
C	1.783926	-3.371132	-0.799798
C	1.561522	-3.436291	-3.040396
H	1.539208	-3.707411	-4.087911
C	4.637649	1.375710	-0.346332
C	0.712606	-1.097642	-3.296304
C	1.888467	-3.034483	1.636477
C	1.175192	-2.149765	-2.520730
C	1.941340	-4.192563	-1.974330
H	2.294709	-5.215579	-1.964002
C	3.224788	1.080951	-0.252042
H	2.515244	1.910791	-0.355903
C	1.448737	-1.374918	3.004614
C	2.158059	-3.508772	2.971502
H	2.511442	-4.503892	3.209011
C	1.883732	-2.480075	3.819527
H	1.965056	-2.453018	4.898391
C	5.601542	0.354585	-0.221631
C	6.949344	0.671618	-0.312828
C	5.037879	2.707689	-0.562154
H	4.287068	3.488392	-0.656739
C	6.391035	3.017525	-0.652026
H	6.706712	4.042660	-0.817808
C	7.342608	2.000425	-0.527289
H	8.399272	2.242453	-0.597252
N	0.551892	0.923897	1.342978
N	0.399334	0.524475	-1.472668
C	-0.171544	2.610814	-0.297462
C	0.122218	2.186146	0.990186
C	0.340049	2.237972	3.232275
H	0.363134	2.507755	4.280138
C	1.092060	-0.129944	3.498757
C	-0.056095	1.828659	-1.435969
C	0.686177	0.940206	2.717200
C	-0.005106	3.010118	2.163495
H	-0.332280	4.040775	2.150078
C	0.347156	0.144805	-2.801581
C	-0.403015	2.268335	-2.760477
H	-0.798416	3.248534	-2.990820
C	-0.152732	1.227038	-3.604706
H	-0.288211	1.181518	-4.677619
O	-0.874130	-1.334814	0.279428

C	-3.039015	-1.935850	-0.434462
C	-1.900832	-1.045079	-0.362104
H	-1.933879	-0.114512	-0.939194
C	-3.078867	-3.124689	0.321564
C	-4.154618	-3.989644	0.180299
C	-4.081077	-1.629049	-1.326546
H	-4.044361	-0.705822	-1.898334
C	-5.144943	-2.513948	-1.481587
H	-5.947219	-2.284491	-2.175579
C	-5.181949	-3.688250	-0.727045
H	-6.015930	-4.374879	-0.841005
H	0.631788	-1.256989	-4.366503
H	-0.552716	3.617618	-0.418384
H	1.140531	0.020533	4.572170
H	2.407045	-4.817794	0.616463
H	7.699746	-0.107090	-0.217983
H	5.271049	-0.665644	-0.055411
H	-2.262597	-3.343354	1.002561
H	-4.197835	-4.904836	0.762749
C	-4.048715	1.285610	1.153922
C	-5.418686	0.771941	0.945240
C	-5.833064	-0.486332	1.142130
H	-5.160399	-1.280294	1.453985
H	-6.871202	-0.764619	0.988654
C	-3.154822	0.702598	1.977390
H	-3.412799	-0.168472	2.572041
H	-2.150855	1.095302	2.093452
C	-3.699352	2.536820	0.421183
C	-3.914494	2.639569	-0.962087
C	-3.178458	3.646823	1.102845
C	-3.591909	3.809056	-1.651057
H	-4.339400	1.794548	-1.498822
C	-2.873406	4.824632	0.419770
H	-3.033262	3.582545	2.176845
C	-3.072436	4.907836	-0.961202
H	-3.760243	3.868241	-2.722971
H	-2.493115	5.683448	0.967034
H	-2.843449	5.827682	-1.492178
H	-6.137903	1.509638	0.592102

$^{4}IIB_{exo\text{-}m}$

Fe	0.672321	-0.533451	0.380722
N	0.599658	-2.336940	-0.503769
N	1.109060	-1.400081	2.120814

O	2.817940	-0.438725	-0.110504
C	1.052023	-3.765977	1.442906
C	0.771092	-3.563768	0.100876
C	0.321499	-4.023950	-2.056035
H	0.127426	-4.491045	-3.012758
C	4.766839	0.621490	-0.959292
C	0.022961	-1.658449	-2.795890
C	1.212667	-2.753107	2.374723
C	0.314757	-2.604590	-1.826111
C	0.602682	-4.618202	-0.862099
H	0.689921	-5.673423	-0.637979
C	3.352262	0.545042	-0.635559
H	2.734475	1.424399	-0.879210
C	1.374978	-0.771713	3.320241
C	1.537074	-2.973286	3.757199
H	1.670636	-3.948156	4.207842
C	1.642128	-1.746447	4.341596
H	1.877512	-1.507565	5.370555
C	5.627889	-0.458449	-0.684426
C	6.975168	-0.360256	-1.004750
C	5.267734	1.792693	-1.554730
H	4.595248	2.621438	-1.763577
C	6.619214	1.885437	-1.873371
H	7.011812	2.787031	-2.333084
C	7.469196	0.809854	-1.598100
H	8.524234	0.881622	-1.846823
N	0.769789	1.268477	1.242217
N	0.210999	0.341714	-1.371313
C	0.137158	2.696398	-0.662418
C	0.515517	2.493408	0.654508
C	1.071936	2.960105	2.786199
H	1.296798	3.431137	3.734337
C	1.386445	0.597851	3.527686
C	-0.005051	1.687545	-1.601117
C	1.100657	1.542221	2.554989
C	0.702951	3.547757	1.611527
H	0.555516	4.597463	1.396813
C	-0.035751	-0.290697	-2.576026
C	-0.388857	1.899270	-2.968017
H	-0.623179	2.866870	-3.390531
C	-0.403030	0.676136	-3.572882
H	-0.643596	0.435445	-4.600352
O	-1.506071	-0.887191	0.724797
C	-3.169806	-1.958806	-0.585920
C	-2.348442	-0.827215	-0.177278
H	-2.467399	0.109006	-0.744808
C	-2.974149	-3.222620	-0.000664
C	-3.732005	-4.303995	-0.432089
C	-4.121943	-1.789965	-1.604659
H	-4.261798	-0.808406	-2.051274
C	-4.881691	-2.874934	-2.030519
H	-5.622886	-2.749875	-2.813946
C	-4.686250	-4.129515	-1.442951
H	-5.278936	-4.977005	-1.775741
H	-0.196620	-2.015892	-3.796288
H	-0.088104	3.710069	-0.972371
H	1.620511	0.955539	4.524662
H	1.163535	-4.788846	1.786774
H	7.646766	-1.187553	-0.796692
H	5.219069	-1.352468	-0.224427
H	-2.222038	-3.329541	0.774247
H	-3.585836	-5.284102	0.011974
C	-3.421727	2.078107	1.428174
C	-4.638396	1.264704	1.210295
C	-4.807546	-0.009162	1.590474
H	-4.021247	-0.571632	2.086250
H	-5.745568	-0.528795	1.420245
C	-2.605003	1.868646	2.477860
H	-2.838846	1.129037	3.236487
H	-1.682723	2.424238	2.604074
C	-3.137005	3.144398	0.428850
C	-3.430571	2.949048	-0.930937
C	-2.569259	4.371914	0.811889
C	-3.140012	3.929820	-1.879831
H	-3.885735	2.014891	-1.249784
C	-2.281376	5.355646	-0.132719
H	-2.377302	4.559914	1.863849
C	-2.560040	5.137298	-1.485789
H	-3.375063	3.753103	-2.926021
H	-1.859267	6.304251	0.188962
H	-2.349535	5.910069	-2.219900
H	-5.451107	1.765533	0.684855

^{6}IIB$_{exo\text{-}m}$

Fe	-0.683134	0.509066	0.388915
N	-0.685166	2.345196	-0.561787
N	-1.171496	1.402149	2.152333
O	-2.784145	0.359774	-0.100468

C	-1.199903	3.747847	1.393588
C	-0.898340	3.561330	0.041827
C	-0.417531	4.011253	-2.120397
H	-0.218343	4.482606	-3.074227
C	-4.716431	-0.709924	-0.966623
C	-0.049169	1.613858	-2.822797
C	-1.331090	2.754811	2.365571
C	-0.380346	2.589136	-1.879340
C	-0.738567	4.611054	-0.935225
H	-0.854013	5.668084	-0.732338
C	-3.306145	-0.626859	-0.638263
H	-2.677387	-1.495052	-0.887456
C	-1.409240	0.768734	3.351191
C	-1.668629	2.978379	3.748506
H	-1.847820	3.949527	4.191738
C	-1.719349	1.754551	4.355355
H	-1.946793	1.532749	5.390084
C	-5.587130	0.361456	-0.685596
C	-6.931658	0.255784	-1.013720
C	-5.205270	-1.879795	-1.576002
H	-4.525293	-2.701071	-1.789554
C	-6.554144	-1.979452	-1.902144
H	-6.937780	-2.879340	-2.372508
C	-7.413388	-0.912658	-1.620764
H	-8.466552	-0.989953	-1.875660
N	-0.745131	-1.336718	1.280305
N	-0.211927	-0.405788	-1.415145
C	-0.102658	-2.740611	-0.641467
C	-0.472412	-2.553032	0.690355
C	-0.990813	-3.002760	2.844797
H	-1.190624	-3.475238	3.798001
C	-1.360732	-0.611290	3.554575
C	0.028996	-1.747543	-1.614091
C	-1.056183	-1.583699	2.600607
C	-0.626762	-3.598931	1.668950
H	-0.465263	-4.650768	1.474247
C	0.037879	0.235491	-2.610568
C	0.434868	-1.960274	-2.979654
H	0.694750	-2.922862	-3.399059
C	0.439150	-0.738456	-3.594537
H	0.693481	-0.510064	-4.621740
O	1.407468	0.925202	0.672105
C	3.104437	1.993153	-0.590333
C	2.273625	0.864302	-0.213258
H	2.397573	-0.070966	-0.777839
C	2.889668	3.256819	-0.008907
C	3.661997	4.337553	-0.413964
C	4.088449	1.824834	-1.579992
H	4.240899	0.844172	-2.024022
C	4.861928	2.909538	-1.978373
H	5.627560	2.786118	-2.738048
C	4.648415	4.163039	-1.393809
H	5.252215	5.010606	-1.705713
H	0.182195	1.965426	-3.823586
H	0.139806	-3.753716	-0.942391
H	-1.575701	-0.964326	4.558338
H	-1.350896	4.771678	1.721910
H	-7.610777	1.075786	-0.801515
H	-5.187729	1.254412	-0.215458
H	2.112102	3.364262	0.740310
H	3.502039	5.317308	0.025780
C	3.454738	-1.964267	1.457105
C	4.643082	-1.104578	1.261307
C	4.751353	0.175881	1.640930
H	3.930551	0.706126	2.116229
H	5.670172	0.734360	1.489828
C	2.621859	-1.794482	2.501324
H	2.824289	-1.055879	3.270029
H	1.718903	-2.383138	2.614736
C	3.220026	-3.029450	0.444029
C	3.531169	-2.813331	-0.908746
C	2.684530	-4.277481	0.806737
C	3.287035	-3.793756	-1.870932
H	3.964734	-1.863688	-1.212016
C	2.444546	-5.261218	-0.150861
H	2.479446	-4.480675	1.853279
C	2.738893	-5.022087	-1.496987
H	3.533202	-3.600038	-2.911471
H	2.046564	-6.225177	0.155479
H	2.564786	-5.794246	-2.241210
H	5.486114	-1.573615	0.754586

^{2}TS1B$_{exo\text{-}m}$

Fe	-0.546863	-0.079974	0.187577
N	-1.885660	0.808837	-1.033194
N	-1.682360	0.359365	1.787325
O	-1.645735	-1.717765	-0.008414
C	-3.524603	1.522173	0.650371

C	-3.060577	1.409076	-0.651183
C	-2.977420	1.647518	-2.889659
H	-3.161115	1.880888	-3.930443
C	-2.156984	-3.986683	-0.423108
C	-0.794827	0.458707	-3.201536
C	-2.870900	1.046293	1.778216
C	-1.820985	0.938478	-2.400946
C	-3.747560	1.935408	-1.805606
H	-4.692490	2.461576	-1.769017
C	-1.253655	-2.861908	-0.282533
H	-0.181190	-3.042855	-0.429526
C	-1.405559	0.093079	3.107092
C	-3.351614	1.223248	3.127563
H	-4.270079	1.732295	3.390070
C	-2.440259	0.637938	3.951711
H	-2.456392	0.561841	5.031276
C	-3.546134	-3.824331	-0.252584
C	-4.386407	-4.919395	-0.398552
C	-1.624003	-5.249446	-0.739073
H	-0.550652	-5.364728	-0.869281
C	-2.472355	-6.342817	-0.884035
H	-2.067223	-7.319795	-1.128168
C	-3.850096	-6.175844	-0.713782
H	-4.512620	-7.029213	-0.827335
N	0.724336	-1.076463	1.390323
N	0.540754	-0.590282	-1.421388
C	2.312619	-1.885981	-0.303710
C	1.855525	-1.759799	1.000580
C	1.790758	-1.946050	3.245463
H	1.975345	-2.174403	4.287394
C	-0.311667	-0.622522	3.570272
C	1.703266	-1.332578	-1.421369
C	0.668557	-1.181626	2.761546
C	2.524403	-2.307324	2.153483
H	3.441106	-2.881165	2.113559
C	0.299165	-0.261576	-2.739362
C	2.202518	-1.464865	-2.765302
H	3.103352	-2.002095	-3.031514
C	1.337771	-0.796075	-3.580259
H	1.378007	-0.679707	-4.655699
O	0.402190	1.539909	0.475033
C	-0.527429	3.591684	-0.366133
C	0.482651	2.481637	-0.448835
H	0.654952	2.133812	-1.473087
C	-1.125411	3.909931	0.859027
C	-2.082576	4.919069	0.923274
C	-0.885793	4.294647	-1.521710
H	-0.429141	4.036737	-2.474441
C	-1.849015	5.300923	-1.455894
H	-2.135408	5.834920	-2.357289
C	-2.445366	5.617435	-0.232991
H	-3.192068	6.404633	-0.181056
H	-0.862578	0.638403	-4.269649
H	3.239178	-2.428332	-0.458338
H	-0.224403	-0.771073	4.641773
H	-4.463710	2.044233	0.800834
H	-5.458227	-4.804592	-0.269417
H	-3.935676	-2.840864	-0.009712
H	-0.858551	3.337014	1.741343
H	-2.552928	5.156775	1.873238
C	3.462491	1.484850	0.553957
C	3.095855	2.557078	-0.303067
C	2.023747	3.446947	-0.097145
H	1.777523	3.723876	0.924734
H	1.970879	4.287297	-0.784419
C	2.752926	1.296262	1.714291
H	2.053701	2.023664	2.099540
H	2.890091	0.402689	2.311863
C	4.530856	0.530509	0.144246
C	4.744468	0.203056	-1.203952
C	5.334890	-0.084288	1.117349
C	5.720502	-0.723691	-1.566434
H	4.110888	0.626205	-1.977313
C	6.305830	-1.017139	0.756534
H	5.212296	0.180793	2.163694
C	6.501438	-1.341928	-0.587698
H	5.864915	-0.968491	-2.615051
H	6.920360	-1.478202	1.524347
H	7.263082	-2.062710	-0.870459
H	3.601840	2.612054	-1.264964

^{4}TS1B$_{exo\text{-}m}$

Fe	-0.257781	0.478993	0.198897
N	-0.009007	2.068668	-1.003130
N	-0.358641	1.698042	1.788923
O	-2.501282	0.843056	-0.053180
C	-0.019070	3.863235	0.676292
C	0.077980	3.390245	-0.622303

C	0.351966	3.398893	-2.856122
H	0.506511	3.659575	-3.895101
C	-4.746783	0.177309	-0.466881
C	0.125353	0.932712	-3.180312
C	-0.214064	3.067781	1.794063
C	0.151696	2.059062	-2.372719
C	0.303568	4.223080	-1.772359
H	0.416678	5.298529	-1.737065
C	-3.311089	-0.040883	-0.339909
H	-2.949014	-1.068263	-0.513883
C	-0.523887	1.329006	3.105802
C	-0.281645	3.567135	3.142194
H	-0.197579	4.611248	3.414469
C	-0.469020	2.490158	3.955284
H	-0.572197	2.468139	5.032452
C	-5.300663	1.454012	-0.258559
C	-6.671943	1.636617	-0.381520
C	-5.576204	-0.907323	-0.797962
H	-5.139450	-1.890697	-0.956624
C	-6.950145	-0.719217	-0.920369
H	-7.595473	-1.553998	-1.175927
C	-7.494905	0.551248	-0.711749
H	-8.567028	0.698789	-0.806558
N	-0.700341	-1.090103	1.374431
N	-0.321850	-0.724644	-1.418592
C	-0.760748	-2.879565	-0.316514
C	-0.839700	-2.407813	0.984740
C	-1.043020	-2.421713	3.228317
H	-1.174551	-2.686349	4.269640
C	-0.733640	0.037024	3.561271
C	-0.513447	-2.090972	-1.429860
C	-0.823939	-1.083918	2.748189
C	-1.053821	-3.240715	2.135856
H	-1.182921	-4.314229	2.097053
C	-0.102846	-0.360726	-2.730585
C	-0.414289	-2.590953	-2.774154
H	-0.525387	-3.631697	-3.048104
C	-0.156417	-1.520665	-3.579646
H	-0.021079	-1.502335	-4.653411
O	1.770435	0.187440	0.441582
C	3.178301	1.949963	-0.390705
C	2.655439	0.539586	-0.463987
H	2.431198	0.221108	-1.491422
C	3.151828	2.646227	0.822707
C	3.610265	3.960298	0.882103
C	3.670132	2.575521	-1.540820
H	3.680115	2.036631	-2.485731
C	4.124061	3.893367	-1.481200
H	4.495272	4.378520	-2.379419
C	4.097767	4.586826	-0.269085
H	4.454382	5.611825	-0.221747
H	0.275330	1.074789	-4.245498
H	-0.858417	-3.948665	-0.469373
H	-0.847551	-0.107497	4.630542
H	0.083039	4.931927	0.830305
H	-7.107632	2.618337	-0.222145
H	-4.639965	2.276534	-0.003462
H	2.737733	2.158620	1.699699
H	3.579649	4.500251	1.824468
C	2.970156	-2.447242	0.566656
C	3.877691	-1.762639	-0.272784
C	4.224382	-0.404436	-0.120799
H	4.364327	-0.018963	0.886582
H	4.959276	-0.013547	-0.820389
C	2.471679	-1.779803	1.666751
H	2.964765	-0.926435	2.107920
H	1.615394	-2.163834	2.208130
C	2.435504	-3.781442	0.180140
C	2.234376	-4.130270	-1.164828
C	2.093822	-4.715539	1.171360
C	1.700024	-5.371370	-1.507438
H	2.446347	-3.412166	-1.951178
C	1.549290	-5.952823	0.830598
H	2.269580	-4.476822	2.216494
C	1.349641	-6.285544	-0.511373
H	1.549073	-5.620687	-2.554128
H	1.297205	-6.663834	1.612261
H	0.935166	-7.253288	-0.778661
H	4.173329	-2.255445	-1.196843

^{6}TS1B$_{exo\text{-}m}$

Fe	-0.377653	0.365058	0.220787
N	-0.815042	1.969359	-1.002056
N	-1.086251	1.403823	1.849312
O	-2.569604	-0.155254	-0.101659
C	-1.581931	3.539667	0.731682
C	-1.266414	3.198326	-0.585180
C	-0.960915	3.369131	-2.817919

H	-0.901299	3.703608	-3.845683
C	-4.390901	-1.613734	-0.551371
C	-0.192685	0.988637	-3.170297
C	-1.499054	2.714243	1.854708
C	-0.628636	2.040295	-2.361886
C	-1.355569	4.082236	-1.722340
H	-1.676670	5.114785	-1.678233
C	-2.980846	-1.278660	-0.401159
H	-2.255745	-2.091718	-0.567696
C	-1.153144	0.945459	3.142106
C	-1.827709	3.102163	3.206861
H	-2.181733	4.082160	3.500292
C	-1.611827	2.012896	4.001266
H	-1.756165	1.927852	5.070734
C	-5.386449	-0.639834	-0.349463
C	-6.723892	-0.984085	-0.496020
C	-4.747413	-2.927453	-0.899557
H	-3.970990	-3.673380	-1.053274
C	-6.089464	-3.267370	-1.045834
H	-6.370327	-4.280974	-1.314794
C	-7.074216	-2.295858	-0.843822
H	-8.121786	-2.560389	-0.957169
N	-0.268448	-1.351337	1.373590
N	0.021627	-0.781185	-1.469974
C	0.426874	-2.947497	-0.370810
C	0.138999	-2.597227	0.949988
C	-0.115533	-2.745314	3.192431
H	-0.163190	-3.075788	4.222334
C	-0.841677	-0.352028	3.552714
C	0.375196	-2.110718	-1.488381
C	-0.436274	-1.413849	2.737736
C	0.238014	-3.474794	2.089469
H	0.545491	-4.511455	2.043501
C	0.099589	-0.317366	-2.762211
C	0.688394	-2.499066	-2.842138
H	0.996233	-3.492146	-3.142607
C	0.521668	-1.392675	-3.627791
H	0.661095	-1.307302	-4.698011
O	1.511911	0.882692	0.487276
C	2.197681	3.030873	-0.351425
C	2.248784	1.528515	-0.397971
H	2.198650	1.133232	-1.420855
C	1.854280	3.686238	0.836527
C	1.781357	5.076693	0.866549
C	2.471150	3.773797	-1.505054
H	2.724516	3.261360	-2.430471
C	2.392719	5.165775	-1.473980
H	2.593789	5.738985	-2.374363
C	2.051860	5.819201	-0.287256
H	1.994143	6.903609	-0.262170
H	-0.089218	1.200516	-4.230239
H	0.757073	-3.967072	-0.541610
H	-0.935645	-0.563021	4.613659
H	-1.921068	4.556957	0.900360
H	-7.498625	-0.239150	-0.341523
H	-5.086675	0.368197	-0.080874
H	1.613713	3.095841	1.714997
H	1.505206	5.582655	1.787352
C	3.640798	-1.177074	0.665794
C	4.210846	-0.150851	-0.130159
C	4.010737	1.232920	0.057813
H	3.941886	1.609456	1.075667
H	4.587589	1.887194	-0.591240
C	2.916180	-0.824169	1.780552
H	2.961634	0.160549	2.222529
H	2.305059	-1.553408	2.300073
C	3.715792	-2.596646	0.223071
C	3.681928	-2.941098	-1.137391
C	3.791805	-3.626374	1.174268
C	3.714376	-4.276955	-1.534112
H	3.574710	-2.169317	-1.893836
C	3.815142	-4.962790	0.778800
H	3.849912	-3.379186	2.230589
C	3.775971	-5.292993	-0.577964
H	3.681638	-4.524036	-2.591535
H	3.880545	-5.745321	1.529246
H	3.803128	-6.333659	-0.887698
H	4.696923	-0.458767	-1.054045

^{2}IIIB$_{exo-m}$

Fe	-0.337475	0.288847	0.122841
N	-0.310704	2.285017	0.087967
N	-0.267341	0.291675	2.124300
O	-2.281834	0.402405	0.254517
C	-0.000126	2.705779	2.488680
C	-0.145506	3.114062	1.171456
C	-0.386870	4.489196	-0.595713
H	-0.465974	5.333369	-1.267871

C	-4.533507	-0.105324	-0.241481
C	-0.598077	2.687480	-2.316832
C	-0.081233	1.392809	2.927561
C	-0.462118	3.110829	-1.003743
C	-0.176179	4.491183	0.748738
H	-0.050832	5.337740	1.410671
C	-3.105198	-0.252384	-0.409017
H	-2.743326	-0.973357	-1.151914
C	-0.408298	-0.782258	2.975419
C	-0.068908	0.995612	4.312986
H	0.067696	1.674900	5.144508
C	-0.285430	-0.348958	4.342938
H	-0.356150	-1.001987	5.203091
C	-5.065603	0.803807	0.695644
C	-6.441700	0.918005	0.831734
C	-5.390338	-0.892267	-1.034064
H	-4.970091	-1.590747	-1.753503
C	-6.768735	-0.771986	-0.891466
H	-7.435527	-1.375623	-1.498926
C	-7.290650	0.131498	0.039663
H	-8.367140	0.225301	0.150980
N	-0.469515	-1.713945	0.153621
N	-0.454647	0.272331	-1.879352
C	-0.410467	-2.157076	-2.263650
C	-0.490892	-2.556145	-0.937996
C	-0.769458	-3.898560	0.849342
H	-0.893190	-4.732259	1.527089
C	-0.611784	-2.097112	2.577868
C	-0.411386	-0.837737	-2.696581
C	-0.635448	-2.526532	1.259919
C	-0.668867	-3.918756	-0.507827
H	-0.694438	-4.772238	-1.171188
C	-0.565200	1.361711	-2.723934
C	-0.472169	-0.435586	-4.076792
H	-0.452078	-1.118943	-4.915997
C	-0.581697	0.923715	-4.092664
H	-0.662007	1.582969	-4.947250
O	1.676428	0.268947	0.094192
C	2.794126	2.403583	-0.428596
C	2.437175	1.018755	-0.916037
H	1.763567	1.080824	-1.767017
C	3.087285	2.668747	0.913163
C	3.506523	3.940777	1.303938
C	2.924685	3.429887	-1.371350
H	2.684521	3.233760	-2.413962
C	3.352053	4.699399	-0.982799
H	3.449245	5.486848	-1.724825
C	3.645758	4.957584	0.357355
H	3.976901	5.946010	0.662602
H	-0.694471	3.448208	-3.084443
H	-0.407119	-2.934130	-3.020874
H	-0.726766	-2.846792	3.353683
H	0.140259	3.477613	3.238179
H	-6.862297	1.614804	1.550056
H	-4.386941	1.400649	1.296440
H	2.957524	1.891891	1.659535
H	3.724893	4.136917	2.350088
C	2.894287	-1.810197	-0.278109
C	3.509937	-1.254704	-1.335092
C	3.702298	0.234628	-1.347620
H	4.524937	0.501580	-0.667970
H	3.995636	0.606338	-2.333815
C	2.410827	-0.815769	0.750818
H	3.240434	-0.348052	1.294268
H	1.720758	-1.237047	1.472000
C	2.696866	-3.257998	-0.071517
C	2.586393	-4.136555	-1.164142
C	2.632376	-3.799215	1.223122
C	2.430506	-5.506215	-0.968327
H	2.593561	-3.735201	-2.173234
C	2.472871	-5.171011	1.419824
H	2.729068	-3.151325	2.089969
C	2.371866	-6.030616	0.325834
H	2.346987	-6.166322	-1.827462
H	2.437165	-5.567953	2.430603
H	2.249709	-7.099075	0.478236
H	3.918626	-1.851587	-2.144941

^{4}IIIB$_{exo\text{-}m}$

Fe	-0.322097	0.345351	0.206830
N	-0.038185	2.316610	0.191495
N	-0.192387	0.327887	2.199816
O	-2.503705	0.699568	0.373933
C	0.351852	2.693372	2.590231
C	0.249426	3.119254	1.276250
C	0.209918	4.507570	-0.493926
H	0.256897	5.353720	-1.166424
C	-4.770723	0.400139	-0.272137

C	-0.259024	2.764952	-2.216341
C	0.126755	1.394252	3.016225
C	-0.062474	3.157166	-0.902319
C	0.414488	4.481901	0.852703
H	0.660468	5.303071	1.512561
C	-3.338524	0.171805	-0.369393
H	-2.994243	-0.515376	-1.159665
C	-0.403688	-0.743971	3.043491
C	0.138506	0.976499	4.390800
H	0.363907	1.625104	5.227334
C	-0.201402	-0.344251	4.408745
H	-0.306921	-1.001105	5.262409
C	-5.297993	1.259499	0.710950
C	-6.669748	1.461840	0.781129
C	-5.627757	-0.249456	-1.177931
H	-5.211653	-0.911446	-1.933825
C	-7.001879	-0.042063	-1.103431
H	-7.668400	-0.540301	-1.800478
C	-7.519541	0.812138	-0.124918
H	-8.592100	0.974231	-0.066170
N	-0.661862	-1.630013	0.216530
N	-0.458398	0.348851	-1.794214
C	-0.724369	-2.062972	-2.204686
C	-0.803735	-2.460230	-0.879882
C	-1.124983	-3.785773	0.910663
H	-1.294443	-4.612153	1.587317
C	-0.737936	-2.029550	2.642918
C	-0.575730	-0.749772	-2.625482
C	-0.851430	-2.437660	1.323849
C	-1.085651	-3.801683	-0.451114
H	-1.215334	-4.643426	-1.116951
C	-0.417420	1.451347	-2.629553
C	-0.594759	-0.328722	-3.997754
H	-0.677445	-0.998527	-4.843915
C	-0.508962	1.033661	-3.999697
H	-0.501103	1.706018	-4.847804
O	1.913564	0.070977	0.129164
C	3.149838	2.067251	-0.540960
C	2.684385	0.685012	-0.941403
H	1.991887	0.754512	-1.782302
C	3.455710	2.380346	0.787216
C	3.959719	3.639377	1.116360
C	3.349859	3.033856	-1.533207
H	3.100445	2.801003	-2.566308
C	3.860006	4.290500	-1.206620
H	4.012914	5.031228	-1.986603
C	4.168228	4.595752	0.120814
H	4.564818	5.573902	0.377596
H	-0.244400	3.535520	-2.979632
H	-0.827312	-2.828722	-2.966128
H	-0.890292	-2.775402	3.415695
H	0.594159	3.436225	3.342619
H	-7.085464	2.122503	1.535762
H	-4.617497	1.750633	1.399228
H	3.275314	1.645875	1.565489
H	4.190622	3.872577	2.152327
C	2.811026	-2.151706	-0.318241
C	3.492810	-1.669653	-1.371090
C	3.868774	-0.215116	-1.367478
H	4.702038	-0.045474	-0.669267
H	4.218087	0.127211	-2.345924
C	2.482045	-1.119554	0.736783
H	3.373740	-0.816453	1.301999
H	1.728235	-1.458153	1.442729
C	2.401064	-3.558690	-0.144079
C	2.128749	-4.377829	-1.254416
C	2.287063	-4.122093	1.138020
C	1.774053	-5.714518	-1.088871
H	2.172157	-3.952457	-2.252867
C	1.929366	-5.460084	1.304646
H	2.503028	-3.520399	2.016814
C	1.672941	-6.262962	0.192584
H	1.570271	-6.329119	-1.961664
H	1.861660	-5.877582	2.305561
H	1.397747	-7.305793	0.321498
H	3.806572	-2.303361	-2.195450

^{6}IIIB$_{exo\text{-}m}$

Fe	-0.268416	0.410426	0.132022
N	-0.004470	1.982409	-1.149277
N	-0.233586	1.685674	1.730080
O	-2.403748	0.791493	-0.033508
C	0.081126	3.800852	0.510755
C	0.090349	3.312194	-0.794071
C	0.193661	3.258167	-3.051177
H	0.264461	3.503531	-4.103092
C	-4.682759	0.207627	-0.342127
C	-0.016430	0.754892	-3.284819

C	-0.058820	3.049881	1.678426
C	0.048598	1.924715	-2.525907
C	0.217590	4.112101	-1.983887
H	0.320007	5.189293	-1.993607
C	-3.262707	-0.074617	-0.247513
H	-2.947349	-1.121428	-0.374274
C	-0.360566	1.340475	3.057437
C	-0.050419	3.578790	3.018366
H	0.075560	4.624235	3.268541
C	-0.232063	2.524698	3.869605
H	-0.287238	2.542127	4.950498
C	-5.169240	1.520906	-0.190339
C	-6.533143	1.761599	-0.282889
C	-5.572490	-0.854147	-0.586241
H	-5.187466	-1.864607	-0.701505
C	-6.938571	-0.606238	-0.677987
H	-7.630512	-1.421180	-0.865954
C	-7.415462	0.699481	-0.526104
H	-8.481992	0.893210	-0.597274
N	-0.696442	-1.166782	1.392381
N	-0.386734	-0.879695	-1.478446
C	-0.817936	-2.982639	-0.269371
C	-0.882145	-2.486570	1.033732
C	-1.035380	-2.421413	3.288126
H	-1.146940	-2.661162	4.337816
C	-0.610836	0.053233	3.534256
C	-0.573209	-2.244372	-1.427963
C	-0.781222	-1.102900	2.768929
C	-1.098430	-3.273219	2.218688
H	-1.257314	-4.343289	2.224813
C	-0.205533	-0.542697	-2.802039
C	-0.492261	-2.784567	-2.761108
H	-0.594501	-3.833459	-3.006788
C	-0.266129	-1.735920	-3.608765
H	-0.154289	-1.760568	-4.685183
O	1.914026	-0.008646	0.301601
C	3.125253	2.008639	-0.320438
C	2.798779	0.583457	-0.700324
H	2.231356	0.558649	-1.632864
C	3.191216	2.408883	1.017750
C	3.543160	3.718923	1.344107
C	3.419736	2.934206	-1.327269
H	3.351610	2.634645	-2.370479
C	3.774911	4.242996	-1.002150
H	3.996085	4.954303	-1.792947
C	3.837889	4.638968	0.336032
H	4.112961	5.658656	0.590399
H	0.076341	0.865791	-4.360786
H	-0.925101	-4.055859	-0.387071
H	-0.692053	-0.060507	4.610856
H	0.205553	4.871884	0.630838
H	-6.917976	2.770134	-0.167088
H	-4.464161	2.324271	-0.002283
H	2.930990	1.704127	1.800732
H	3.579813	4.021342	2.387014
C	2.905037	-2.219316	-0.017463
C	3.737864	-1.733556	-0.953018
C	4.072304	-0.270625	-0.897032
H	4.765759	-0.062312	-0.068748
H	4.569809	0.078795	-1.806243
C	2.431230	-1.191000	0.985015
H	3.242485	-0.856818	1.644194
H	1.607301	-1.540049	1.598235
C	2.429732	-3.614890	0.056757
C	2.236782	-4.370443	-1.113661
C	2.129900	-4.213640	1.291422
C	1.760596	-5.677757	-1.050331
H	2.421464	-3.910714	-2.079839
C	1.652209	-5.522478	1.355830
H	2.277133	-3.659162	2.214216
C	1.461834	-6.259492	0.185562
H	1.614058	-6.242275	-1.967285
H	1.434854	-5.968042	2.322675
H	1.089283	-7.278656	0.234703
H	4.161218	-2.359118	-1.733513

^{4}TS1B'$_{exo\text{-}m}$

Fe	-0.469718	0.051661	0.315330
N	-1.271670	0.981333	-1.272353
N	-1.358476	1.366140	1.532722
O	-2.424320	-1.164345	0.468578
C	-2.615105	2.684399	-0.115129
C	-2.137834	2.055255	-1.254560
C	-1.715836	1.659519	-3.432342
H	-1.677476	1.672503	-4.514015
C	-4.220567	-2.400474	-0.483155
C	-0.237784	-0.332539	-3.075187
C	-2.220170	2.377985	1.179093

C	-1.009816	0.721302	-2.601383
C	-2.429040	2.471071	-2.599407
H	-3.082679	3.293658	-2.858339
C	-2.968659	-1.654096	-0.522369
H	-2.496330	-1.529970	-1.512149
C	-1.155658	1.488336	2.888837
C	-2.579947	3.145975	2.342518
H	-3.250914	3.995025	2.333525
C	-1.902237	2.610692	3.397074
H	-1.914307	2.922023	4.433611
C	-4.898315	-2.602253	0.733371
C	-6.091619	-3.312834	0.745542
C	-4.747396	-2.915673	-1.679314
H	-4.216675	-2.754584	-2.614934
C	-5.943614	-3.627768	-1.662005
H	-6.354913	-4.027786	-2.583656
C	-6.612973	-3.824506	-0.450573
H	-7.546744	-4.379559	-0.436041
N	0.165049	-0.981411	1.900130
N	0.210745	-1.391103	-0.903635
C	0.941972	-3.025289	0.776954
C	0.713119	-2.244769	1.902745
C	0.781494	-1.551402	4.046607
H	0.917063	-1.458190	5.116379
C	-0.400429	0.624944	3.670098
C	0.759532	-2.597607	-0.530797
C	0.174723	-0.551845	3.208590
C	1.091161	-2.610462	3.242259
H	1.541335	-3.555278	3.518573
C	0.296232	-1.337848	-2.278285
C	1.195103	-3.320185	-1.696477
H	1.663529	-4.295475	-1.675285
C	0.930335	-2.530331	-2.776287
H	1.123381	-2.733769	-3.821672
O	1.268897	1.119610	0.248718
C	0.951447	3.338658	-0.635343
C	1.607365	1.979682	-0.693619
H	1.618471	1.570827	-1.714118
C	0.581619	3.890154	0.595407
C	-0.042410	5.134485	0.643036
C	0.701512	4.043654	-1.816412
H	0.979075	3.609257	-2.774007
C	0.070024	5.287161	-1.768865
H	-0.132931	5.825837	-2.690170
C	-0.300275	5.835682	-0.538924
H	-0.787519	6.805916	-0.501130
H	-0.108001	-0.418684	-4.149016
H	1.370728	-4.010771	0.928069
H	-0.322244	0.842147	4.730265
H	-3.288265	3.523948	-0.249691
H	-6.621424	-3.472623	1.679783
H	-4.472042	-2.194842	1.644674
H	0.754551	3.320058	1.502902
H	-0.339983	5.550800	1.601149
C	4.083105	0.157940	0.301568
C	4.094414	1.247225	-0.601479
C	3.344398	2.436933	-0.446226
H	3.259299	2.849427	0.557534
H	3.532833	3.207204	-1.190818
C	3.288410	0.254532	1.423324
H	2.906867	1.194075	1.791507
H	3.078741	-0.611310	2.042847
C	4.819945	-1.089428	-0.050953
C	6.058327	-1.008686	-0.707335
C	4.314516	-2.356136	0.273652
C	6.764052	-2.164008	-1.042508
H	6.491419	-0.037532	-0.933386
C	5.018537	-3.510266	-0.063019
H	3.356336	-2.441134	0.771676
C	6.244751	-3.419992	-0.725076
H	7.724547	-2.080631	-1.542606
H	4.606681	-4.483685	0.190003
H	6.794308	-4.319970	-0.984970
H	4.551352	1.069952	-1.573383

^{2}VB$_{endo\text{-}m}$

Fe	1.189226	-0.897384	-0.603560
N	0.772529	0.160257	-2.252185
N	-0.506496	-1.908590	-0.863208
C	-1.458086	-0.716966	-2.787525
C	-0.387429	0.133126	-3.004274
C	0.843747	1.782095	-3.909416
H	1.238438	2.590644	-4.510907
C	2.799601	1.548071	-2.380178
C	-1.514749	-1.661677	-1.781476
C	1.533431	1.179469	-2.802376
C	-0.342689	1.125000	-4.040981
H	-1.127505	1.285526	-4.768470

C	-1.005493	-2.912863	-0.050720
C	-2.637586	-2.525514	-1.547605
H	-3.547518	-2.518279	-2.131894
C	-2.324753	-3.297455	-0.473111
H	-2.924117	-4.057784	0.008536
N	1.762354	-2.230344	0.766918
N	3.055292	-0.161280	-0.633285
C	4.066452	-1.480277	1.178655
C	2.972256	-2.285596	1.438449
C	1.687298	-3.837610	2.433679
H	1.281761	-4.627902	3.051825
C	-0.331322	-3.489533	1.009181
C	4.099907	-0.484560	0.215632
C	0.967588	-3.177696	1.379488
C	2.933217	-3.288412	2.465559
H	3.761691	-3.530656	3.118202
C	3.517062	0.906341	-1.381255
C	5.223744	0.375864	-0.017488
H	6.165280	0.312703	0.512177
C	4.860004	1.244135	-1.003798
H	5.443793	2.036496	-1.453958
O	0.530786	0.408872	0.692442
C	0.395468	2.484948	1.813352
C	0.780482	1.631134	0.713981
H	1.325777	2.078627	-0.125492
C	-0.295756	1.970226	2.929337
C	-0.675566	2.825892	3.953906
C	0.711693	3.855611	1.745964
H	1.250042	4.242917	0.884505
C	0.330384	4.706019	2.777563
H	0.569473	5.763570	2.730049
C	-0.365369	4.190972	3.876760
H	-0.666826	4.856085	4.680774
H	3.283510	2.369532	-2.898662
H	4.957211	-1.629630	1.779888
H	-0.843032	-4.264952	1.569721
H	-2.315389	-0.625321	-3.443835
H	-0.523587	0.910712	2.971629
H	-1.212570	2.439387	4.814339
C	-3.329453	1.478428	-0.866899
C	-2.368505	2.491857	-0.377828
C	-1.443727	3.112950	-1.123140
H	-1.294015	2.879688	-2.172807
H	-0.819253	3.893820	-0.699553
C	-3.850820	1.560336	-2.106639
H	-3.627621	2.398256	-2.759151
H	-4.527557	0.804582	-2.493909
H	-2.436698	2.732933	0.682842
C	-3.705467	0.393758	0.077697
C	-4.987668	-0.182900	0.066306
C	-2.774732	-0.077702	1.017907
C	-5.319945	-1.203533	0.955219
H	-5.738464	0.194944	-0.621435
C	-3.107758	-1.093901	1.911305
H	-1.769899	0.326180	1.021260
C	-4.382491	-1.662247	1.884463
H	-6.319742	-1.628270	0.936143
H	-2.364710	-1.451309	2.619784
H	-4.648222	-2.448558	2.585588

$^{4}VB_{endo\text{-}m}$

Fe	1.238766	-0.932757	-0.549997
N	0.861350	0.055144	-2.246395
N	-0.448849	-1.962810	-0.791328
C	-1.375142	-0.830203	-2.769263
C	-0.293095	-0.003655	-3.010583
C	0.987623	1.554334	-4.006793
H	1.404217	2.313330	-4.656131
C	2.918102	1.396395	-2.442502
C	-1.440887	-1.742244	-1.734344
C	1.654465	1.014404	-2.858843
C	-0.216477	0.919460	-4.103463
H	-0.992320	1.052813	-4.846014
C	-0.944363	-2.967551	0.024150
C	-2.556757	-2.611147	-1.500634
H	-3.460252	-2.618824	-2.094756
C	-2.248977	-3.370848	-0.413242
H	-2.847876	-4.131110	0.068923
N	1.769174	-2.178683	0.913979
N	3.085385	-0.159298	-0.542374
C	4.027027	-1.331101	1.407409
C	2.944982	-2.158088	1.646434
C	1.676653	-3.737891	2.622948
H	1.275174	-4.525805	3.246653
C	-0.286320	-3.516605	1.108707
C	4.089789	-0.405224	0.380903
C	0.983607	-3.148459	1.514569
C	2.891718	-3.125524	2.704012

H	3.692628	-3.306015	3.409036
C	3.586923	0.835438	-1.368365
C	5.220184	0.438115	0.125878
H	6.134086	0.428613	0.705375
C	4.908280	1.207695	-0.956301
H	5.514615	1.957588	-1.447519
O	0.506413	0.508367	0.788577
C	0.277723	2.683080	1.704848
C	0.732762	1.728732	0.715774
H	1.317339	2.113360	-0.132251
C	-0.484947	2.268615	2.815146
C	-0.933098	3.212025	3.729639
C	0.593784	4.043527	1.531082
H	1.185892	4.354063	0.673535
C	0.144104	4.983204	2.452348
H	0.383071	6.033891	2.322232
C	-0.620420	4.566476	3.547341
H	-0.975080	5.300618	4.265015
H	3.428223	2.166025	-3.011939
H	4.887920	-1.421426	2.061000
H	-0.792312	-4.296285	1.667551
H	-2.225091	-0.759057	-3.436919
H	-0.716673	1.216047	2.936450
H	-1.526003	2.902428	4.584561
C	-3.385341	1.347168	-0.972881
C	-2.437223	2.398861	-0.541552
C	-1.517121	2.981532	-1.321672
H	-1.364118	2.683350	-2.354406
H	-0.896818	3.790047	-0.946393
C	-3.923835	1.367218	-2.207572
H	-3.726186	2.182881	-2.895659
H	-4.588759	0.582401	-2.555805
H	-2.508999	2.701042	0.503042
C	-3.723126	0.294362	0.021234
C	-4.986718	-0.321666	0.044859
C	-2.771689	-0.107659	0.973314
C	-5.280463	-1.314113	0.978369
H	-5.754144	0.003221	-0.651540
C	-3.066470	-1.095447	1.911097
H	-1.779567	0.326036	0.953573
C	-4.322717	-1.704478	1.918042
H	-6.266725	-1.769828	0.985331
H	-2.307180	-1.398742	2.627408
H	-4.558944	-2.468827	2.653222

$^{6}VB_{endo\text{-}m}$

Fe	1.146437	-0.874095	-0.439032
N	0.805052	-0.001080	-2.280547
N	-0.525529	-2.028416	-0.736410
C	-1.427731	-0.932035	-2.745582
C	-0.351726	-0.097521	-3.031668
C	0.929326	1.434109	-4.083037
H	1.338288	2.170068	-4.763252
C	2.882672	1.301915	-2.507825
C	-1.511331	-1.825860	-1.684997
C	1.604686	0.930141	-2.918399
C	-0.276543	0.794238	-4.155998
H	-1.049507	0.906361	-4.905258
C	-0.999368	-3.017308	0.105720
C	-2.626980	-2.692736	-1.420650
H	-3.535927	-2.722503	-2.006067
C	-2.311510	-3.427884	-0.315154
H	-2.910892	-4.178948	0.181212
N	1.768779	-2.234577	0.971851
N	3.101020	-0.206414	-0.574746
C	4.051417	-1.398576	1.360137
C	2.969677	-2.222035	1.655891
C	1.708535	-3.792712	2.672344
H	1.324253	-4.577578	3.310835
C	-0.302622	-3.547221	1.187065
C	4.117750	-0.469980	0.325006
C	0.985080	-3.196508	1.581766
C	2.934388	-3.192644	2.716976
H	3.750386	-3.388461	3.400469
C	3.586007	0.763509	-1.433259
C	5.256944	0.354940	0.026167
H	6.189695	0.339906	0.574929
C	4.927896	1.118618	-1.057599
H	5.539243	1.849794	-1.570633
O	0.520953	0.606960	0.819671
C	0.331831	2.820645	1.639737
C	0.756031	1.826742	0.685001
H	1.327020	2.162385	-0.191474
C	-0.429289	2.462030	2.772249
C	-0.843561	3.446887	3.657475
C	0.676992	4.167875	1.413673
H	1.265507	4.434390	0.539201
C	0.261653	5.148017	2.307275

H	0.523634	6.187621	2.139075
C	-0.499141	4.786296	3.424747
H	-0.827268	5.552762	4.120892
H	3.392543	2.052188	-3.104170
H	4.932052	-1.499753	1.986571
H	-0.799992	-4.322944	1.760631
H	-2.277795	-0.883894	-3.416607
H	-0.687011	1.420496	2.931025
H	-1.434425	3.182309	4.528648
C	-3.352873	1.340739	-0.974435
C	-2.399985	2.400429	-0.574710
C	-1.472724	2.951100	-1.370556
H	-1.314866	2.615722	-2.391160
H	-0.854998	3.773832	-1.022661
C	-3.888718	1.327371	-2.210336
H	-3.682941	2.119415	-2.923082
H	-4.558037	0.536519	-2.535525
H	-2.476851	2.740542	0.458041
C	-3.699585	0.319345	0.048766
C	-4.968352	-0.284900	0.088953
C	-2.751152	-0.066620	1.010488
C	-5.269263	-1.251914	1.046507
H	-5.733570	0.029223	-0.614749
C	-3.051468	-1.031687	1.970094
H	-1.757824	0.364751	0.983606
C	-4.312720	-1.630001	1.992211
H	-6.259390	-1.698778	1.065645
H	-2.293804	-1.325393	2.692106
H	-4.553242	-2.376644	2.743943

^{2}TS2B$_{endo\text{-}m}$

Fe	1.298152	-0.643618	0.701906
N	2.391027	-1.992538	-0.279405
N	0.052645	-2.115585	1.268726
C	1.040957	-4.017915	0.076248
C	2.111020	-3.334380	-0.475683
C	3.889152	-2.915491	-1.787213
H	4.724284	-2.966945	-2.473668
C	4.102221	-0.495468	-1.223794
C	0.068934	-3.438555	0.875462
C	3.474794	-1.725379	-1.096768
C	3.052882	-3.915378	-1.391420
H	3.056767	-4.955743	-1.689565
C	-1.110965	-1.964069	1.992594
C	-1.097437	-4.124587	1.364851
H	-1.305685	-5.174499	1.204461
C	-1.838707	-3.204605	2.044823
H	-2.778853	-3.343957	2.562507
N	0.484315	0.544191	2.078726
N	2.739154	0.712804	0.419271
C	1.973189	2.495995	1.920417
C	0.868520	1.828390	2.421322
C	-1.098469	1.515263	3.465517
H	-2.001671	1.605346	4.054949
C	-1.500209	-0.800680	2.637003
C	2.829895	1.979618	0.964956
C	-0.736234	0.351691	2.704447
C	-0.095919	2.423463	3.303163
H	-0.010054	3.416970	3.723322
C	3.774610	0.634001	-0.492191
C	3.929157	2.700295	0.382714
H	4.211131	3.709802	0.651688
C	4.507693	1.871225	-0.529690
H	5.368300	2.055967	-1.159429
O	0.206287	-0.150554	-0.737951
C	0.040597	2.191129	-1.275718
C	0.463989	0.801452	-1.606741
H	1.443487	0.738923	-2.097904
C	-0.928229	2.415969	-0.290045
C	-1.292266	3.718781	0.042319
C	0.646942	3.276462	-1.918605
H	1.413109	3.101158	-2.670837
C	0.282385	4.580553	-1.582965
H	0.757431	5.420652	-2.080980
C	-0.689988	4.803003	-0.604802
H	-0.973565	5.818375	-0.343266
H	4.943991	-0.428920	-1.905359
H	2.151502	3.508384	2.266538
H	-2.445005	-0.810626	3.170435
H	0.938989	-5.071774	-0.161439
H	-1.368529	1.567604	0.222286
H	-2.033767	3.888330	0.817671
C	-2.402107	-0.678700	-1.913144
C	-1.800220	0.245324	-2.815131
C	-0.453580	0.241769	-3.204549
H	0.071271	-0.706821	-3.283020
H	-0.156089	0.976714	-3.948444
C	-1.679141	-1.759613	-1.478183

H	-0.757501	-2.081056	-1.943723
H	-2.036340	-2.370533	-0.656397
H	-2.385287	1.125640	-3.072490
C	-3.747033	-0.392161	-1.342207
C	-4.646663	-1.444046	-1.105925
C	-4.135980	0.916461	-1.011054
C	-5.899655	-1.196332	-0.547802
H	-4.373067	-2.457608	-1.386984
C	-5.390001	1.162630	-0.453996
H	-3.447226	1.743702	-1.156611
C	-6.274961	0.108157	-0.219965
H	-6.587811	-2.020121	-0.382061
H	-5.673817	2.179474	-0.198128
H	-7.253277	0.302337	0.209824

^{4}TS2B$_{endo\text{-}m}$

Fe	0.999399	-0.778436	0.684347
N	2.165197	-2.251938	-0.014553
N	-0.453841	-2.115959	1.002971
C	0.602683	-4.145520	0.097190
C	1.819910	-3.572773	-0.228740
C	3.863920	-3.344718	-1.141690
H	4.817582	-3.488025	-1.632778
C	4.160862	-0.931381	-0.574681
C	-0.463344	-3.453471	0.650297
C	3.416249	-2.100278	-0.579348
C	2.882690	-4.260889	-0.909065
H	2.861931	-5.309467	-1.176082
C	-1.736371	-1.836791	1.441385
C	-1.765641	-4.014913	0.873515
H	-2.027641	-5.048636	0.688526
C	-2.559389	-3.007594	1.343529
H	-3.603748	-3.045014	1.623577
N	0.023401	0.502563	1.877092
N	2.601554	0.407547	0.775322
C	1.731486	2.248806	2.151648
C	0.472237	1.714586	2.365034
C	-1.711313	1.637913	2.904368
H	-2.711263	1.831496	3.270034
C	-2.162194	-0.622826	1.954243
C	2.705922	1.650285	1.374343
C	-1.320244	0.446190	2.205034
C	-0.594381	2.409758	3.029229
H	-0.490582	3.374399	3.508518
C	3.790060	0.225200	0.091175
C	3.968103	2.252606	1.050680
H	4.290495	3.223464	1.403290
C	4.631632	1.379238	0.241388
H	5.615432	1.481402	-0.197880
O	0.359853	-0.202556	-1.099008
C	0.659127	2.180207	-1.441830
C	0.748079	0.758209	-1.909551
H	1.684141	0.546948	-2.448182
C	-0.366676	2.586015	-0.582940
C	-0.447226	3.911801	-0.165406
C	1.612443	3.109668	-1.868835
H	2.419237	2.794395	-2.526820
C	1.539207	4.437367	-1.441547
H	2.285809	5.153433	-1.772498
C	0.506126	4.841671	-0.594376
H	0.443824	5.875781	-0.267839
H	5.129079	-0.945552	-1.063734
H	1.945580	3.226042	2.569746
H	-3.206889	-0.525636	2.228315
H	0.459467	-5.196260	-0.131459
H	-1.077488	1.849532	-0.226106
H	-1.242896	4.215524	0.508730
C	-2.273371	-0.247576	-2.168006
C	-1.650546	0.740951	-2.970594
C	-0.325366	0.647070	-3.444129
H	0.031198	-0.329010	-3.766061
H	0.018913	1.458664	-4.080925
C	-1.600338	-1.431134	-1.963665
H	-0.784230	-1.760636	-2.590297
H	-1.928652	-2.135548	-1.210352
H	-2.144084	1.706562	-3.049956
C	-3.511857	0.042143	-1.392382
C	-4.411457	-0.995682	-1.095709
C	-3.782687	1.325366	-0.889088
C	-5.530371	-0.765675	-0.297549
H	-4.242802	-1.987436	-1.505755
C	-4.903344	1.556026	-0.091891
H	-3.103396	2.147025	-1.091202
C	-5.777349	0.510910	0.213277
H	-6.217978	-1.579910	-0.087167
H	-5.090627	2.554116	0.293695
H	-6.651409	0.692058	0.831999

^{6}TS2B$_{endo\text{-}m}$

Fe	1.004643	-0.693893	0.506928
N	2.352392	-2.184595	-0.028331
N	-0.368705	-2.207362	0.902398
C	0.830992	-4.115395	-0.089303
C	2.055621	-3.498766	-0.323839
C	4.180132	-3.188309	-1.016863
H	5.181865	-3.296818	-1.412116
C	4.349727	-0.778870	-0.314836
C	-0.292976	-3.518683	0.479039
C	3.648493	-1.975390	-0.447265
C	3.198423	-4.131233	-0.934207
H	3.234939	-5.165764	-1.250402
C	-1.651636	-2.023160	1.378915
C	-1.561113	-4.168905	0.685046
H	-1.768904	-5.204954	0.449890
C	-2.403977	-3.240586	1.231594
H	-3.436998	-3.365132	1.528928
N	-0.056010	0.470142	1.870010
N	2.634072	0.525213	0.872101
C	1.552519	2.305697	2.178415
C	0.309306	1.706098	2.359255
C	-1.867352	1.460113	2.902867
H	-2.876390	1.579163	3.275655
C	-2.134936	-0.845236	1.942793
C	2.624022	1.766842	1.476327
C	-1.386117	0.297597	2.202310
C	-0.814391	2.321733	3.016926
H	-0.790056	3.290679	3.498869
C	3.889286	0.373179	0.316768
C	3.893244	2.416009	1.273735
H	4.149006	3.398346	1.649101
C	4.671824	1.559437	0.551201
H	5.693297	1.699151	0.221640
O	0.442847	-0.132441	-1.239119
C	0.540249	2.274964	-1.562554
C	0.681290	0.870859	-2.063900
H	1.578472	0.729633	-2.683235
C	-0.432494	2.601442	-0.612737
C	-0.564584	3.914532	-0.169355
C	1.387202	3.271623	-2.058874
H	2.150142	3.020099	-2.792297
C	1.261455	4.587021	-1.607236
H	1.925494	5.355660	-1.991369
C	0.282004	4.911085	-0.666864
H	0.178911	5.935433	-0.320773
H	5.366890	-0.759806	-0.693504
H	1.688861	3.294897	2.603213
H	-3.178793	-0.831112	2.238818
H	0.741385	-5.158438	-0.376730
H	-1.060403	1.816796	-0.205996
H	-1.315903	4.155314	0.576926
C	-2.344037	-0.320130	-2.106048
C	-1.787124	0.712293	-2.910733
C	-0.501861	0.678085	-3.489342
H	-0.128152	-0.281319	-3.840176
H	-0.253776	1.503837	-4.152341
C	-1.663469	-1.508451	-2.001052
H	-0.842978	-1.774686	-2.652650
H	-1.959628	-2.260317	-1.279970
H	-2.313295	1.663853	-2.928642
C	-3.553955	-0.075925	-1.270997
C	-4.435998	-1.131937	-0.987020
C	-3.811404	1.182818	-0.704011
C	-5.526240	-0.942563	-0.140309
H	-4.278095	-2.104493	-1.444385
C	-4.902821	1.372340	0.142839
H	-3.144711	2.017221	-0.895018
C	-5.760014	0.309628	0.433691
H	-6.201739	-1.769191	0.060122
H	-5.079290	2.351430	0.578709
H	-6.611644	0.458352	1.091042

^{2}VIB$_{endo\text{-}m}$

Fe	0.770359	-0.923859	0.728802
N	0.615208	-2.787797	0.022137
N	-0.978765	-1.186960	1.672780
C	-1.560084	-3.460962	0.946041
C	-0.434187	-3.677486	0.167125
C	0.929582	-4.630888	-1.345831
H	1.410083	-5.281813	-2.064548
C	2.680554	-2.851866	-1.308518
C	-1.825036	-2.284464	1.625743
C	1.458222	-3.368354	-0.909027
C	-0.232387	-4.832026	-0.662889
H	-0.905440	-5.677956	-0.711893
C	-1.689058	-0.213152	2.349345
C	-3.064117	-1.992024	2.291423

H	-3.881724	-2.692422	2.400639
C	-2.989896	-0.700365	2.717210
H	-3.731895	-0.123134	3.252743
N	1.086800	0.688343	1.836856
N	2.677248	-0.893734	0.174934
C	3.458829	1.112112	1.356633
C	2.254845	1.423768	1.959737
C	0.707541	2.602428	3.086920
H	0.163046	3.328970	3.675710
C	-1.185431	1.024995	2.712215
C	3.643842	0.042570	0.496956
C	0.129691	1.414557	2.524334
C	2.027740	2.597816	2.752874
H	2.789218	3.325264	3.000472
C	3.269771	-1.722523	-0.762697
C	4.849082	-0.208703	-0.238997
H	5.754252	0.377820	-0.151629
C	4.611492	-1.290651	-1.035312
H	5.285074	-1.778530	-1.727762
O	-0.020265	-0.149001	-0.936956
C	1.190781	1.925271	-1.361424
C	0.791740	0.586312	-1.928953
H	1.675976	-0.029865	-2.102207
C	0.299893	2.701678	-0.611571
C	0.676015	3.972210	-0.176658
C	2.459696	2.430899	-1.662588
H	3.162104	1.822900	-2.228310
C	2.832383	3.704864	-1.233406
H	3.818923	4.089829	-1.475518
C	1.939147	4.479162	-0.490167
H	2.227673	5.470435	-0.152496
H	3.249449	-3.412886	-2.042699
H	4.296923	1.779799	1.523071
H	-1.840505	1.698927	3.254071
H	-2.310045	-4.244336	0.978486
H	-0.673930	2.300705	-0.353885
H	-0.016902	4.564823	0.413468
C	-2.090374	0.445964	-2.037490
C	-1.422174	1.130729	-2.982752
C	-0.001979	0.719343	-3.254544
H	0.028628	-0.244340	-3.783819
H	0.525248	1.440293	-3.884142
C	-1.302151	-0.684171	-1.430707
H	-1.069682	-1.488106	-2.137684
H	-1.779813	-1.110827	-0.558638
H	-1.851506	1.983485	-3.498843
C	-3.422384	0.779809	-1.496994
C	-4.258210	-0.216355	-0.962224
C	-3.873667	2.112085	-1.475858
C	-5.507920	0.106094	-0.435870
H	-3.943957	-1.256699	-0.975155
C	-5.123791	2.432952	-0.952548
H	-3.227165	2.901925	-1.847518
C	-5.946312	1.431332	-0.430202
H	-6.142289	-0.680012	-0.036207
H	-5.452789	3.468179	-0.942234
H	-6.919746	1.683182	-0.019599

^{4}VIB$_{endo\text{-}m}$

Fe	0.836920	-0.962611	0.735321
N	0.797029	-2.791381	-0.066080
N	-0.898573	-1.353311	1.642079
C	-1.351589	-3.621479	0.798263
C	-0.201836	-3.744899	0.036824
C	1.260351	-4.585908	-1.451941
H	1.796584	-5.193155	-2.169431
C	2.891907	-2.704840	-1.353610
C	-1.682422	-2.493067	1.527371
C	1.701863	-3.305456	-0.980565
C	0.090276	-4.865565	-0.809366
H	-0.532577	-5.746147	-0.896782
C	-1.657755	-0.461787	2.384617
C	-2.932685	-2.305976	2.203898
H	-3.713454	-3.052887	2.264062
C	-2.925909	-1.041535	2.715568
H	-3.698059	-0.540886	3.284479
N	1.038869	0.655421	1.875668
N	2.724521	-0.760070	0.147408
C	3.357150	1.287625	1.354654
C	2.146724	1.478343	1.992723
C	0.543327	2.481628	3.209148
H	-0.040686	3.140071	3.838684
C	-1.232058	0.781866	2.815834
C	3.612459	0.252430	0.473234
C	0.046953	1.271840	2.620511
C	1.847602	2.600941	2.833455
H	2.552946	3.382662	3.081790
C	3.382746	-1.537351	-0.794524

C	4.827235	0.106632	-0.271649
H	5.678961	0.768933	-0.188518
C	4.678767	-0.990797	-1.069900
H	5.387086	-1.415809	-1.769048
O	-0.022569	-0.095180	-0.996968
C	1.067665	2.051202	-1.332309
C	0.732291	0.717049	-1.956900
H	1.650754	0.160890	-2.163667
C	0.152097	2.732155	-0.521776
C	0.463160	3.997718	-0.025240
C	2.297535	2.648069	-1.629260
H	3.021038	2.114686	-2.242082
C	2.606445	3.916463	-1.136575
H	3.563412	4.371356	-1.376492
C	1.687371	4.595395	-0.334204
H	1.925086	5.582648	0.051646
H	3.510333	-3.215541	-2.083853
H	4.139185	2.020143	1.519200
H	-1.921552	1.379100	3.402395
H	-2.056865	-4.445482	0.790186
H	-0.791555	2.262266	-0.268655
H	-0.250172	4.515641	0.609333
C	-2.138142	0.475984	-2.039973
C	-1.508245	1.212459	-2.972060
C	-0.075634	0.868667	-3.272234
H	-0.012566	-0.075504	-3.833230
H	0.414164	1.631316	-3.882791
C	-1.299900	-0.638646	-1.469254
H	-1.077592	-1.427267	-2.198424
H	-1.747363	-1.092011	-0.592140
H	-1.978325	2.060409	-3.459917
C	-3.480987	0.737470	-1.485688
C	-4.267773	-0.306664	-0.967653
C	-3.992884	2.046716	-1.433474
C	-5.528250	-0.052993	-0.428977
H	-3.906839	-1.331220	-1.004994
C	-5.253668	2.298926	-0.898185
H	-3.384610	2.873187	-1.789595
C	-6.027019	1.250199	-0.393459
H	-6.124249	-0.875766	-0.044058
H	-5.629479	3.317628	-0.864445
H	-7.008916	1.448564	0.026291

$^{6}VIB_{endo\text{-}m}$

Fe	0.782628	-0.877476	0.630551
N	0.751568	-2.842517	-0.014927
N	-0.949932	-1.283456	1.684582
C	-1.460090	-3.530936	0.816857
C	-0.293986	-3.740334	0.085414
C	1.200757	-4.704163	-1.302954
H	1.737725	-5.366744	-1.969331
C	2.901127	-2.856494	-1.216948
C	-1.768780	-2.392008	1.555170
C	1.678104	-3.419933	-0.863530
C	-0.013070	-4.905449	-0.711007
H	-0.666067	-5.763977	-0.799760
C	-1.639687	-0.385744	2.480170
C	-2.999151	-2.172749	2.266697
H	-3.811383	-2.885498	2.326994
C	-2.923293	-0.930386	2.829193
H	-3.661012	-0.426971	3.440137
N	1.093883	0.716491	1.894545
N	2.771835	-0.803397	0.136891
C	3.404568	1.287590	1.269453
C	2.216832	1.519939	1.952827
C	0.679448	2.533475	3.255692
H	0.139476	3.196780	3.918794
C	-1.137775	0.832735	2.927086
C	3.662163	0.215900	0.420327
C	0.141565	1.326063	2.689061
C	1.962963	2.649181	2.806531
H	2.680689	3.429223	3.023521
C	3.422168	-1.657531	-0.736809
C	4.883938	0.006488	-0.305558
H	5.747496	0.657245	-0.258945
C	4.732994	-1.145068	-1.027443
H	5.450291	-1.621279	-1.683310
O	-0.019327	-0.123791	-1.100654
C	1.059250	2.039127	-1.401033
C	0.725091	0.719074	-2.054042
H	1.642920	0.166597	-2.274417
C	0.147645	2.700984	-0.569295
C	0.463335	3.953350	-0.043331
C	2.288948	2.640774	-1.689571
H	3.009873	2.121080	-2.316833
C	2.599802	3.897914	-1.170309
H	3.555707	4.357758	-1.404377
C	1.685730	4.557393	-0.346478

H	1.926805	5.534454	0.062336
H	3.527921	-3.427797	-1.894513
H	4.197139	2.017353	1.396832
H	-1.785724	1.428843	3.561780
H	-2.200230	-4.324767	0.802600
H	-0.797080	2.229386	-0.322697
H	-0.244735	4.454969	0.609563
C	-2.153581	0.468926	-2.091228
C	-1.534853	1.222190	-3.018151
C	-0.107229	0.884454	-3.350465
H	-0.051926	-0.051233	-3.925941
H	0.369797	1.657267	-3.958225
C	-1.312915	-0.661330	-1.559296
H	-1.094779	-1.433144	-2.305900
H	-1.742530	-1.132783	-0.681523
H	-2.009954	2.082870	-3.477759
C	-3.483185	0.726630	-1.504347
C	-4.263823	-0.323016	-0.988207
C	-3.986629	2.037415	-1.418811
C	-5.511012	-0.072561	-0.418341
H	-3.909396	-1.348656	-1.050360
C	-5.234404	2.286137	-0.852510
H	-3.382202	2.867363	-1.773343
C	-6.001974	1.232231	-0.349779
H	-6.102482	-0.898834	-0.034261
H	-5.604079	3.305826	-0.792779
H	-6.973401	1.427913	0.094760

^{4}TS2B'$_{endo-m}$

Fe	1.380594	-0.527963	0.648025
N	2.638626	-1.730939	-0.357008
N	0.354937	-2.142597	1.243158
C	1.487002	-3.885223	-0.075358
C	2.488918	-3.084203	-0.596081
C	4.273454	-2.469507	-1.819617
H	5.137163	-2.425412	-2.470179
C	4.229204	-0.055112	-1.199214
C	0.493315	-3.439133	0.779496
C	3.726868	-1.343042	-1.115257
C	3.508773	-3.549239	-1.494783
H	3.613902	-4.574629	-1.824517
C	-0.755944	-2.161572	2.066551
C	-0.551502	-4.270085	1.306907
H	-0.658991	-5.326716	1.098828
C	-1.328125	-3.477349	2.101229
H	-2.202352	-3.751227	2.677437
N	0.432913	0.560286	2.047271
N	2.661099	0.990966	0.382677
C	1.607754	2.705445	1.797876
C	0.625498	1.897078	2.340136
C	-1.182352	1.294457	3.534609
H	-2.042299	1.249953	4.190129
C	-1.249620	-1.078288	2.775410
C	2.550599	2.277275	0.880757
C	-0.682514	0.183802	2.771917
C	-0.369081	2.354321	3.270172
H	-0.425472	3.363053	3.657446
C	3.734616	1.026388	-0.490439
C	3.559233	3.122204	0.308110
H	3.683840	4.171094	0.543094
C	4.290281	2.349140	-0.544051
H	5.141569	2.631748	-1.149643
O	0.208754	-0.120436	-0.870794
C	-0.323706	2.194933	-1.230379
C	0.271931	0.901867	-1.697071
H	1.242941	1.026469	-2.197974
C	-1.369638	2.191277	-0.300312
C	-1.896571	3.395564	0.159105
C	0.193509	3.409241	-1.691588
H	1.017944	3.411728	-2.401635
C	-0.331549	4.616353	-1.226232
H	0.075747	5.557580	-1.584115
C	-1.378252	4.610543	-0.301985
H	-1.789613	5.549146	0.058316
H	5.085235	0.112412	-1.844151
H	1.631272	3.746493	2.100028
H	-2.128715	-1.236146	3.391434
H	1.482081	-4.934069	-0.352467
H	-1.750342	1.244536	0.068351
H	-2.704910	3.387318	0.884433
C	-2.351334	-0.858349	-1.974549
C	-1.923682	0.171985	-2.851963
C	-0.592912	0.368083	-3.267311
H	0.025305	-0.505868	-3.462072
H	-0.421361	1.184647	-3.965141
C	-1.457353	-1.828813	-1.596170
H	-0.526925	-2.006695	-2.116049
H	-1.691754	-2.521536	-0.794252

H	-2.619861	0.992921	-3.008694
C	-3.689766	-0.741605	-1.329578
C	-3.826846	-0.910359	0.056300
C	-4.824086	-0.437283	-2.095017
C	-5.075980	-0.781555	0.661592
H	-2.950648	-1.117750	0.663103
C	-6.073565	-0.312662	-1.487492
H	-4.734338	-0.320086	-3.172340
C	-6.202824	-0.483960	-0.107828
H	-5.168372	-0.905992	1.737116
H	-6.946318	-0.087397	-2.093274
H	-7.175713	-0.384763	0.364435

$^{2}VB_{exo-m}$

Fe	1.556916	-0.825106	0.353805
N	2.957494	0.230792	1.313518
N	0.422352	-0.757163	2.001388
O	0.758955	0.720344	-0.528169
C	1.779384	0.728584	3.413349
C	2.862275	0.831069	2.557989
C	4.834002	1.524291	1.732374
H	5.800687	1.979791	1.561711
C	-0.471208	2.733857	-0.569530
C	4.671864	0.319342	-0.443787
C	0.633400	-0.000367	3.142751
C	4.161642	0.666858	0.796215
C	4.030035	1.621652	2.827393
H	4.197627	2.176316	3.741336
C	-0.244028	1.369523	-0.158547
H	-0.950803	0.912039	0.542300
C	-0.846364	-1.287233	2.150143
C	-0.511873	-0.061127	4.005052
H	-0.585740	0.439460	4.961709
C	-1.433865	-0.852053	3.384973
H	-2.423422	-1.132965	3.719591
C	0.384574	3.364871	-1.496971
C	0.173767	4.695354	-1.826646
C	-1.531501	3.449602	0.016224
H	-2.188857	2.952290	0.723453
C	-1.721739	4.791239	-0.301772
H	-2.532851	5.349375	0.154612
C	-0.873514	5.409443	-1.223144
H	-1.026494	6.454203	-1.477970
N	0.357921	-2.232111	-0.398489
N	2.855315	-1.192802	-1.115817
C	1.573079	-2.798742	-2.458069
C	0.498967	-2.917399	-1.593161
C	-1.494666	-3.571175	-0.786969
H	-2.458948	-4.026497	-0.609859
C	-1.459252	-2.152234	1.258920
C	2.668134	-1.984506	-2.235575
C	-0.878387	-2.618103	0.093844
C	-0.640153	-3.758889	-1.830817
H	-0.754892	-4.406184	-2.690363
C	4.064902	-0.556000	-1.326358
C	3.773583	-1.849456	-3.142854
H	3.864243	-2.379644	-4.081804
C	4.637027	-0.959399	-2.582153
H	5.586249	-0.607062	-2.964206
H	5.629782	0.740700	-0.730169
H	1.552067	-3.381120	-3.373265
H	-2.460061	-2.493367	1.495538
H	1.825586	1.266809	4.354315
H	0.823289	5.188853	-2.542909
H	1.197948	2.796131	-1.935852
C	-3.923726	1.778509	-1.929751
C	-2.178291	0.023076	-2.199247
H	-1.724594	0.547148	-3.034913
H	-1.725244	-0.918205	-1.906748
C	-3.348812	2.811597	-2.559752
H	-2.302373	2.808877	-2.851727
H	-3.915578	3.705267	-2.801743
C	-3.278653	0.496736	-1.578571
H	-4.967578	1.864329	-1.631975
C	-3.936003	-0.295832	-0.499846
C	-4.274749	-1.642121	-0.703712
C	-4.254422	0.296930	0.732437
C	-4.904779	-2.379083	0.299897
H	-4.058330	-2.098108	-1.664959
C	-4.864344	-0.443596	1.745593
H	-4.023706	1.347127	0.895060
C	-5.194264	-1.784759	1.531521
H	-5.183053	-3.413728	0.117105
H	-5.098843	0.030363	2.694862
H	-5.691400	-2.355937	2.310417

$^{4}VB_{exo-m}$

Fe	0.721336	1.148363	-0.673722

N	2.466179	0.355741	-1.279567
N	-0.237707	-0.062375	-1.943699
O	0.688463	-0.217601	0.886444
C	1.634893	-1.471354	-2.701576
C	2.644630	-0.764323	-2.073311
C	4.707748	-0.217705	-1.364542
H	5.770502	-0.158696	-1.168919
C	1.403133	-2.467683	1.079465
C	4.027082	1.725127	0.038054
C	0.293568	-1.137421	-2.641234
C	3.731912	0.687003	-0.827533
C	4.033854	-1.117604	-2.135195
H	4.428770	-1.950849	-2.701538
C	0.444760	-1.431474	0.756624
H	-0.521638	-1.738815	0.334308
C	-1.603143	-0.117882	-2.173236
C	-0.750079	-1.866711	-3.301440
H	-0.589104	-2.743305	-3.915399
C	-1.923763	-1.235918	-3.010585
H	-2.926747	-1.490642	-3.324370
C	2.718277	-2.125941	1.450715
C	3.641699	-3.131725	1.702028
C	1.025571	-3.817869	0.961473
H	0.006162	-4.067013	0.677961
C	1.953214	-4.820029	1.220767
H	1.668387	-5.864212	1.138255
C	3.258629	-4.475283	1.590576
H	3.983719	-5.258872	1.791385
N	-0.955136	2.196993	-0.436186
N	1.725752	2.560379	0.307068
C	-0.134596	4.001702	1.022257
C	-1.131479	3.335817	0.333203
C	-3.185919	2.816386	-0.421202
H	-4.242851	2.771518	-0.645816
C	-2.534708	0.778195	-1.684909
C	1.198326	3.633497	1.006115
C	-2.220931	1.868138	-0.895401
C	-2.511579	3.727473	0.336909
H	-2.900426	4.591937	0.859046
C	3.087835	2.597261	0.558824
C	2.236036	4.336382	1.702164
H	2.076377	5.214367	2.314216
C	3.406142	3.693677	1.426303
H	4.404946	3.936235	1.764846
H	5.064900	1.873967	0.316230
H	-0.414012	4.874139	1.602811
H	-3.577674	0.606647	-1.922972
H	1.914130	-2.341055	-3.286430
H	4.659541	-2.879854	1.983446
H	2.989450	-1.077514	1.518957
C	-2.089691	-1.870942	2.903594
C	-2.226125	0.566822	2.523041
H	-1.542599	0.751577	3.345319
H	-2.575534	1.433858	1.974266
C	-0.887184	-1.967048	3.489091
H	-0.173095	-1.147857	3.480250
H	-0.585313	-2.869532	4.011795
C	-2.622225	-0.680460	2.204897
H	-2.750574	-2.736828	2.926226
C	-3.626081	-0.922073	1.131985
C	-4.700147	-0.037934	0.929863
C	-3.527979	-2.044327	0.292861
C	-5.625382	-0.253714	-0.089958
H	-4.820537	0.809975	1.597195
C	-4.450904	-2.262048	-0.730888
H	-2.717222	-2.753927	0.438716
C	-5.502450	-1.365167	-0.929794
H	-6.458012	0.433242	-0.216296
H	-4.352800	-3.137588	-1.366959
H	-6.232476	-1.541777	-1.714653

^{6}VB$_{exo-m}$

Fe	-0.873624	1.002730	0.496525
N	-2.669179	0.135173	1.086598
N	0.064991	-0.208510	1.895171
O	-0.609564	-0.252852	-1.045553
C	-1.792548	-1.733243	2.427766
C	-2.820389	-1.042824	1.790229
C	-4.873085	-0.542902	0.999288
H	-5.926880	-0.524673	0.752759
C	-0.961970	-2.594971	-1.122764
C	-4.208574	1.567853	-0.193029
C	-0.455759	-1.350817	2.477580
C	-3.917454	0.452753	0.589116
C	-4.195055	-1.466421	1.742127
H	-4.582931	-2.355627	2.221953
C	-0.160053	-1.416975	-0.916483
H	0.877252	-1.540603	-0.586952

C	1.415520	-0.205689	2.187014
C	0.594979	-2.081052	3.135915
H	0.455476	-3.009054	3.675318
C	1.750514	-1.375924	2.952985
H	2.746125	-1.613309	3.302921
C	-2.344897	-2.479915	-1.375484
C	-3.113887	-3.627448	-1.504015
C	-0.362865	-3.864405	-1.000144
H	0.703589	-3.938235	-0.803817
C	-1.138332	-5.008652	-1.137598
H	-0.685128	-5.991007	-1.050453
C	-2.510886	-4.887943	-1.389253
H	-3.116551	-5.783671	-1.493832
N	0.757317	2.254045	0.586978
N	-1.958252	2.561094	-0.285916
C	-0.127135	4.148527	-0.712200
C	0.892647	3.474129	-0.047968
C	2.935445	2.989819	0.779174
H	3.978544	2.988829	1.065099
C	2.312655	0.789500	1.812494
C	-1.450473	3.730531	-0.819208
C	2.002762	1.942122	1.099022
C	2.250093	3.937598	0.074350
H	2.620472	4.871747	-0.327667
C	-3.307648	2.553072	-0.586104
C	-2.499228	4.460072	-1.479384
H	-2.368981	5.412019	-1.977453
C	-3.645644	3.731815	-1.337521
H	-4.638481	3.971059	-1.695769
H	-5.240416	1.692746	-0.506195
H	0.128977	5.096385	-1.174910
H	3.348924	0.651843	2.100354
H	-2.059915	-2.655309	2.934190
H	-4.180569	-3.550294	-1.689330
H	-2.788049	-1.492174	-1.446383
C	2.406520	-1.439060	-3.022502
C	2.306137	0.948998	-2.401198
H	1.655288	1.155241	-3.244718
H	2.544600	1.784158	-1.751982
C	1.230011	-1.588162	-3.649562
H	0.439156	-0.846309	-3.574717
H	1.031473	-2.451208	-4.277806
C	2.797587	-0.285944	-2.181051
H	3.151552	-2.228658	-3.115795
C	3.754652	-0.552489	-1.071638
C	4.716877	0.400683	-0.695287
C	3.715898	-1.765539	-0.363549
C	5.586079	0.161645	0.367473
H	4.795823	1.324559	-1.259750
C	4.579604	-2.005326	0.705817
H	2.999742	-2.532067	-0.650143
C	5.516952	-1.040345	1.079122
H	6.333688	0.905634	0.629443
H	4.526829	-2.950228	1.239685
H	6.202680	-1.230997	1.899715

^{2}TS2B$_{exo-m}$

Fe	0.481365	-0.652429	0.921714
N	2.065906	0.432271	1.507470
N	-0.714813	0.698264	1.780932
O	0.424743	-0.039587	-0.856549
C	0.929690	2.434352	2.360093
C	2.063407	1.717086	2.021111
C	4.234160	1.222829	1.714636
H	5.314954	1.219903	1.663661
C	2.186640	1.405263	-1.619474
C	3.875565	-1.071230	0.806225
C	-0.364875	1.948024	2.263683
C	3.397829	0.124812	1.312556
C	3.406661	2.211013	2.152578
H	3.666207	3.187403	2.540453
C	0.781134	1.197289	-1.151508
H	0.426772	1.974710	-0.465760
C	-2.094124	0.644693	1.880125
C	-1.534322	2.676868	2.668637
H	-1.520109	3.672958	3.091764
C	-2.604984	1.865911	2.436966
H	-3.653209	2.059777	2.621254
C	2.909300	0.341977	-2.176368
C	4.228969	0.535307	-2.575464
C	2.789475	2.660277	-1.470726
H	2.229573	3.479844	-1.026038
C	4.112911	2.848617	-1.865440
H	4.583091	3.819058	-1.737041
C	4.832391	1.788068	-2.422437
H	5.861920	1.936744	-2.734945
N	-1.076348	-1.907291	0.749674
N	1.703367	-2.180142	0.491028

C	0.058781	-3.882655	-0.164860
C	-1.073599	-3.178910	0.209924
C	-3.244207	-2.706671	0.565088
H	-4.323321	-2.713612	0.644926
C	-2.886281	-0.422482	1.495675
C	1.353364	-3.414697	-0.023037
C	-2.407166	-1.608967	0.969024
C	-2.416683	-3.680869	0.091766
H	-2.674672	-4.660577	-0.289140
C	3.082854	-2.139820	0.424616
C	2.526072	-4.159396	-0.394543
H	2.512592	-5.160485	-0.805469
C	3.598731	-3.369798	-0.114275
H	4.650526	-3.586419	-0.248849
H	4.949639	-1.177198	0.696974
H	-0.077271	-4.874582	-0.583211
H	-3.960402	-0.314157	1.599496
H	1.064048	3.436472	2.754060
H	4.791231	-0.291141	-3.000530
H	2.437571	-0.631711	-2.262346
C	-1.504836	1.666969	-2.357970
C	-1.817018	-0.717898	-2.192557
H	-0.874741	-0.915382	-2.683420
H	-2.352389	-1.583199	-1.817481
C	-0.155555	1.625317	-2.742308
H	0.202098	0.773662	-3.314295
H	0.284335	2.568375	-3.054623
C	-2.331249	0.544946	-2.054666
H	-1.921237	2.649559	-2.144589
C	-3.699024	0.761532	-1.506214
C	-4.726201	-0.150163	-1.798944
C	-3.989635	1.852056	-0.671357
C	-5.999520	0.010158	-1.255081
H	-4.533964	-0.980195	-2.472985
C	-5.264889	2.017293	-0.133157
H	-3.207601	2.550497	-0.388785
C	-6.273522	1.094708	-0.418270
H	-6.782038	-0.702613	-1.498357
H	-5.467720	2.864444	0.515973
H	-7.267555	1.225115	-0.000858

^{4}TS2B$_{exo\text{-}m}$

Fe	0.435858	-0.673183	0.907925
N	2.018927	0.389771	1.535719
N	-0.769822	0.658376	1.803765
O	0.354335	0.081549	-0.914882
C	0.873258	2.401297	2.365914
C	2.007370	1.679331	2.037539
C	4.183614	1.168023	1.793101
H	5.265223	1.161249	1.768426
C	2.200379	1.450950	-1.610937
C	3.844198	-1.125816	0.887432
C	-0.418735	1.907480	2.282741
C	3.356122	0.070252	1.381266
C	3.348525	2.167227	2.193591
H	3.603129	3.148422	2.572490
C	0.757880	1.299167	-1.217943
H	0.414312	2.090558	-0.538946
C	-2.145363	0.587575	1.933947
C	-1.586806	2.622863	2.715088
H	-1.573258	3.618684	3.138864
C	-2.654396	1.800943	2.507532
H	-3.700092	1.984924	2.714329
C	2.912414	0.351091	-2.104077
C	4.259804	0.484777	-2.430006
C	2.842925	2.683743	-1.451738
H	2.291047	3.534532	-1.058156
C	4.193551	2.814157	-1.774125
H	4.692001	3.769698	-1.639578
C	4.902782	1.716011	-2.266707
H	5.953699	1.818799	-2.521306
N	-1.120583	-1.918639	0.714069
N	1.669660	-2.190072	0.456391
C	0.020654	-3.858638	-0.279656
C	-1.111486	-3.166263	0.117217
C	-3.282823	-2.712589	0.501818
H	-4.361818	-2.726885	0.580128
C	-2.936465	-0.474995	1.536913
C	1.315629	-3.405891	-0.098051
C	-2.452938	-1.634722	0.958138
C	-2.452623	-3.660136	-0.023436
H	-2.710749	-4.618516	-0.455109
C	3.051662	-2.173699	0.453363
C	2.489727	-4.156702	-0.447866
H	2.476199	-5.144262	-0.890327
C	3.565005	-3.396330	-0.099083
H	4.617205	-3.629076	-0.198492
H	4.920151	-1.241947	0.817531

H	-0.116025	-4.834097	-0.734168
H	-4.009948	-0.378045	1.654616
H	1.007359	3.404802	2.755630
H	4.812088	-0.372495	-2.804432
H	2.409018	-0.606272	-2.194899
C	-1.464726	1.792114	-2.481381
C	-1.674574	-0.591504	-2.262398
H	-0.775182	-0.775592	-2.831923
H	-2.153195	-1.463655	-1.831054
C	-0.093159	1.763858	-2.805286
H	0.284207	0.926427	-3.387251
H	0.349619	2.712341	-3.099094
C	-2.245495	0.654850	-2.149810
H	-1.911081	2.767466	-2.297094
C	-3.598476	0.819507	-1.551147
C	-4.602901	-0.126584	-1.812171
C	-3.894426	1.892709	-0.696152
C	-5.861151	-0.012821	-1.223250
H	-4.403675	-0.947025	-2.495996
C	-5.155529	2.012175	-0.113978
H	-3.125214	2.614692	-0.438124
C	-6.142212	1.058094	-0.371063
H	-6.627433	-0.750439	-1.443481
H	-5.363815	2.847782	0.548256
H	-7.125346	1.153209	0.080517

^{6}TS2B$_{exo-m}$

Fe	0.459238	-0.780364	0.680537
N	2.184968	0.042344	1.480690
N	-0.618257	0.527674	1.877214
O	0.424829	0.171036	-0.982656
C	1.209494	2.042183	2.528481
C	2.285568	1.277951	2.087295
C	4.407041	0.629477	1.677207
H	5.483272	0.551337	1.596002
C	2.151001	1.775565	-1.473808
C	3.826908	-1.569848	0.609494
C	-0.136193	1.684157	2.456002
C	3.475801	-0.371260	1.223296
C	3.672595	1.649430	2.206851
H	4.028195	2.570911	2.649229
C	0.725912	1.448908	-1.146958
H	0.296269	2.127743	-0.400193
C	-1.989633	0.551544	2.037231
C	-1.231897	2.455544	2.984412
H	-1.128278	3.402352	3.498812
C	-2.375148	1.752824	2.730155
H	-3.393377	2.012438	2.988391
C	3.007559	0.786349	-1.971141
C	4.342436	1.091915	-2.227523
C	2.634848	3.069676	-1.241423
H	1.968515	3.833378	-0.845853
C	3.970177	3.371816	-1.499166
H	4.345338	4.373276	-1.310347
C	4.824883	2.383401	-1.995746
H	5.865651	2.618897	-2.198182
N	-1.231724	-1.975925	0.588769
N	1.571436	-2.463244	0.197049
C	-0.255211	-3.949197	-0.512064
C	-1.332689	-3.190859	-0.057932
C	-3.452075	-2.573647	0.417615
H	-4.526448	-2.508095	0.528395
C	-2.868456	-0.422633	1.574887
C	1.090852	-3.622952	-0.375025
C	-2.520184	-1.588883	0.900044
C	-2.719052	-3.563440	-0.175722
H	-3.075924	-4.475352	-0.637146
C	2.947336	-2.542785	0.141698
C	2.192358	-4.448594	-0.801304
H	2.090630	-5.416616	-1.274707
C	3.338770	-3.782279	-0.480422
H	4.362318	-4.096768	-0.638434
H	4.887327	-1.764846	0.484269
H	-0.486701	-4.894561	-0.992905
H	-3.926599	-0.247134	1.737603
H	1.441170	2.991485	3.001461
H	5.009351	0.321181	-2.603017
H	2.626766	-0.219145	-2.117873
C	-1.502153	1.969251	-2.343750
C	-1.815144	-0.423039	-2.303683
H	-0.916224	-0.593041	-2.879978
H	-2.329987	-1.306535	-1.940869
C	-0.144308	1.954399	-2.716232
H	0.228699	1.134170	-3.324296
H	0.283869	2.915765	-2.987389
C	-2.312533	0.831994	-2.061016
H	-1.925433	2.939252	-2.088922
C	-3.637915	1.017245	-1.407673

C	-4.698366	0.146451	-1.704880
C	-3.848440	2.034209	-0.463076
C	-5.929384	0.278273	-1.064691
H	-4.565057	-0.627689	-2.455407
C	-5.082280	2.172094	0.170964
H	-3.033873	2.693068	-0.175857
C	-6.125908	1.292473	-0.124016
H	-6.740406	-0.400130	-1.312897
H	-5.224473	2.962083	0.902856
H	-7.087796	1.401508	0.368216

^{2}VIB$_{exo\text{-}m}$

Fe	0.361404	0.538227	-1.030516
N	2.017472	-0.528555	-1.469435
N	-0.776907	-0.967894	-1.745585
O	0.155428	-0.157046	1.073905
C	0.974550	-2.605964	-2.253965
C	2.086892	-1.804363	-1.958820
C	4.230365	-1.197740	-1.670783
H	5.309119	-1.140587	-1.614092
C	2.263011	-1.155300	1.719816
C	3.726741	1.086103	-0.782700
C	-0.351665	-2.225921	-2.141286
C	3.331405	-0.140426	-1.281818
C	3.455305	-2.233317	-2.097341
H	3.766283	-3.200457	-2.469853
C	0.806660	-1.412753	1.404305
H	0.726247	-2.029216	0.504397
C	-2.139067	-1.012371	-1.803801
C	-1.481673	-3.068563	-2.437480
H	-1.417000	-4.095641	-2.771945
C	-2.595805	-2.312973	-2.223357
H	-3.634247	-2.591699	-2.343561
C	2.684872	0.065345	2.255880
C	4.031838	0.276068	2.554642
C	3.205976	-2.163805	1.493484
H	2.888977	-3.109266	1.059304
C	4.552225	-1.957586	1.797463
H	5.274365	-2.747853	1.613091
C	4.969447	-0.734822	2.326824
H	6.017212	-0.571260	2.562340
N	-1.272511	1.722296	-0.947345
N	1.516117	2.126897	-0.574692
C	-0.227970	3.804041	-0.176243
C	-1.336298	3.031331	-0.552636
C	-3.476030	2.446954	-0.914594
H	-4.552204	2.399552	-1.009461
C	-2.987983	0.071355	-1.529556
C	1.089947	3.384103	-0.176227
C	-2.583927	1.343594	-1.164941
C	-2.698873	3.499689	-0.533822
H	-3.004982	4.502010	-0.264480
C	2.873085	2.149873	-0.447811
C	2.213610	4.195806	0.214757
H	2.147784	5.218906	0.560879
C	3.325476	3.425780	0.046073
H	4.359995	3.687726	0.225608
H	4.791115	1.248096	-0.647303
H	-0.424970	4.823383	0.142266
H	-4.055373	-0.106969	-1.605921
H	1.174527	-3.614243	-2.604394
H	4.348784	1.231299	2.964279
H	1.962314	0.861107	2.402742
C	-1.416289	-2.121423	2.308032
C	-0.991995	0.203788	1.889149
H	-0.623601	0.487089	2.884190
H	-1.404944	1.086718	1.409208
C	0.067920	-2.150173	2.544359
H	0.320699	-1.668201	3.500486
H	0.460373	-3.169393	2.601900
C	-1.971949	-0.946897	1.963410
H	-2.000162	-3.032313	2.404166
C	-3.394406	-0.746097	1.626388
C	-4.038730	0.472926	1.896247
C	-4.134707	-1.764109	0.995917
C	-5.375821	0.668360	1.552150
H	-3.497226	1.274676	2.390769
C	-5.471355	-1.569848	0.652340
H	-3.644125	-2.699452	0.743680
C	-6.097848	-0.350513	0.925822
H	-5.856405	1.615570	1.780388
H	-6.023835	-2.368863	0.165346
H	-7.139443	-0.198388	0.658289

^{4}VIB$_{exo\text{-}m}$

Fe	0.348512	0.494623	-0.991033
N	1.887898	-0.659953	-1.521985
N	-0.903364	-0.890285	-1.693767

O	0.163733	-0.135391	1.052224
C	0.684286	-2.711581	-2.157867
C	1.839036	-1.976060	-1.957736
C	4.029283	-1.480033	-1.847341
H	5.110795	-1.488074	-1.872717
C	2.281849	-1.112265	1.727153
C	3.767789	0.839712	-0.996455
C	-0.594579	-2.197418	-2.034958
C	3.239550	-0.355966	-1.441398
C	3.163400	-2.485177	-2.161109
H	3.387640	-3.486189	-2.505856
C	0.825766	-1.386301	1.436452
H	0.728496	-2.041830	0.567862
C	-2.284266	-0.808897	-1.777082
C	-1.792158	-2.939153	-2.305754
H	-1.813518	-3.981355	-2.596508
C	-2.837316	-2.077687	-2.150469
H	-3.894870	-2.266820	-2.275884
C	2.697564	0.108933	2.268166
C	4.044129	0.326454	2.560646
C	3.228648	-2.113120	1.485713
H	2.914784	-3.057635	1.047705
C	4.574593	-1.898055	1.781944
H	5.301688	-2.681314	1.587392
C	4.985795	-0.676343	2.319740
H	6.033977	-0.507261	2.549252
N	-1.162264	1.799569	-0.931110
N	1.622747	1.983784	-0.624292
C	0.031557	3.784791	-0.095824
C	-1.113474	3.114137	-0.487460
C	-3.290351	2.709437	-0.886238
H	-4.365806	2.757930	-0.990861
C	-3.037064	0.326521	-1.547455
C	1.306563	3.253704	-0.168276
C	-2.506157	1.544713	-1.166557
C	-2.429823	3.679729	-0.461627
H	-2.654023	4.693935	-0.157834
C	3.005559	1.922349	-0.598613
C	2.499766	3.975666	0.166299
H	2.516124	4.990397	0.542054
C	3.552125	3.153261	-0.108187
H	4.610263	3.351760	0.000358
H	4.846607	0.929264	-0.941439
H	-0.074542	4.803655	0.260740
H	-4.114671	0.247811	-1.633968
H	0.788165	-3.746500	-2.465519
H	4.357534	1.282099	2.971473
H	1.971463	0.899532	2.428424
C	-1.397782	-2.043787	2.388132
C	-0.987667	0.258940	1.865887
H	-0.596041	0.578163	2.839332
H	-1.402362	1.123474	1.357391
C	0.087941	-2.055137	2.616950
H	0.347957	-1.522231	3.543504
H	0.484780	-3.068437	2.725801
C	-1.961321	-0.889581	1.991849
H	-1.975692	-2.952713	2.526912
C	-3.384091	-0.703822	1.644640
C	-4.023298	0.531873	1.840246
C	-4.125742	-1.754204	1.074951
C	-5.358061	0.712979	1.478376
H	-3.482155	1.359463	2.290887
C	-5.458889	-1.574421	0.713927
H	-3.638350	-2.703875	0.876387
C	-6.080913	-0.337665	0.910090
H	-5.835147	1.674197	1.648177
H	-6.012640	-2.398212	0.271818
H	-7.120262	-0.197244	0.627741

^{6}VIB$_{exo-m}$

Fe	0.363166	0.502221	-0.839050
N	1.908282	-0.662243	-1.545600
N	-0.947045	-0.801489	-1.759751
O	0.134264	-0.192974	1.100498
C	0.618459	-2.622811	-2.293928
C	1.811130	-1.953170	-2.034026
C	4.020768	-1.530346	-1.876586
H	5.101578	-1.577454	-1.895974
C	2.245756	-1.221367	1.722462
C	3.808202	0.802377	-0.984842
C	-0.661464	-2.088479	-2.175545
C	3.262022	-0.389229	-1.445292
C	3.126171	-2.497525	-2.236426
H	3.330381	-3.490330	-2.616058
C	0.785273	-1.472841	1.438027
H	0.670965	-2.094175	0.545623
C	-2.320738	-0.666869	-1.834346
C	-1.882645	-2.777715	-2.499653

H	-1.938338	-3.798560	-2.854967
C	-2.906300	-1.898968	-2.290022
H	-3.967135	-2.057567	-2.430513
C	2.681858	-0.018709	2.288774
C	4.034527	0.174630	2.569873
C	3.176385	-2.227490	1.442305
H	2.846329	-3.154953	0.980684
C	4.527448	-2.037026	1.730435
H	5.242443	-2.823512	1.506894
C	4.959702	-0.834430	2.294368
H	6.012444	-0.684206	2.515694
N	-1.136639	1.915427	-0.853120
N	1.717489	2.035910	-0.587925
C	0.150738	3.851655	-0.036541
C	-1.037392	3.215272	-0.384603
C	-3.238643	2.857685	-0.730352
H	-4.315003	2.936886	-0.804703
C	-3.030435	0.484775	-1.516070
C	1.429199	3.311967	-0.140628
C	-2.484215	1.684645	-1.072514
C	-2.346777	3.802378	-0.304931
H	-2.550174	4.811799	0.028500
C	3.094240	1.926890	-0.583917
C	2.650371	4.007099	0.167169
H	2.705549	5.024145	0.533207
C	3.678737	3.152460	-0.110942
H	4.741829	3.331056	-0.015157
H	4.890224	0.860357	-0.933479
H	0.074543	4.872355	0.324823
H	-4.111068	0.436178	-1.596612
H	0.695085	-3.643536	-2.655142
H	4.364969	1.116070	2.999292
H	1.968497	0.776874	2.479168
C	-1.449084	-2.134973	2.354710
C	-1.036256	0.183829	1.909961
H	-0.650919	0.475292	2.893525
H	-1.441228	1.061503	1.414601
C	0.035044	-2.159585	2.597555
H	0.293222	-1.648582	3.536809
H	0.425045	-3.177445	2.685935
C	-2.007381	-0.969216	1.984975
H	-2.030013	-3.047034	2.453633
C	-3.422352	-0.770662	1.613590
C	-4.067492	0.455562	1.846678
C	-4.146719	-1.796848	0.981942
C	-5.394010	0.649953	1.462968
H	-3.538100	1.264337	2.343438
C	-5.472123	-1.603538	0.599600
H	-3.651892	-2.735582	0.752339
C	-6.101244	-0.377532	0.835373
H	-5.877122	1.602414	1.662044
H	-6.013368	-2.407790	0.108903
H	-7.134344	-0.226843	0.535969

^{4}TS2B'$_{exo\text{-}m}$

Fe	-0.601738	0.840443	0.804152
N	-1.857525	-0.418025	1.717777
N	0.854266	0.222916	2.029714
O	-0.183374	-0.284517	-0.745122
C	-0.217678	-1.803424	2.915594
C	-1.500808	-1.518300	2.475113
C	-3.739194	-1.638303	2.279548
H	-4.785494	-1.908618	2.332900
C	-1.952887	-1.814380	-1.313504
C	-4.014772	0.397970	0.867139
C	0.867928	-0.958241	2.746838
C	-3.233462	-0.484776	1.590784
C	-2.665256	-2.289828	2.809286
H	-2.651094	-3.197385	3.398725
C	-0.524408	-1.551807	-0.913129
H	-0.182320	-2.231782	-0.120976
C	2.106857	0.778361	2.205562
C	2.149983	-1.147950	3.368119
H	2.413057	-1.996808	3.985649
C	2.908672	-0.060796	3.052508
H	3.926391	0.159194	3.347050
C	-2.652834	-0.875125	-2.078892
C	-3.984369	-1.104765	-2.415389
C	-2.588872	-2.988447	-0.897333
H	-2.047314	-3.712880	-0.293484
C	-3.925060	-3.214120	-1.229464
H	-4.419659	-4.120856	-0.893408
C	-4.623379	-2.274252	-1.990966
H	-5.662188	-2.452373	-2.253789
N	0.552261	2.373054	0.248919
N	-2.177303	1.776373	-0.004308
C	-0.974657	3.443471	-1.352505
C	0.263322	3.285287	-0.748873

C	2.378300	3.738874	-0.123856
H	3.369955	4.153821	0.002414
C	2.561729	1.934857	1.594625
C	-2.121306	2.770345	-0.962766
C	1.847751	2.646939	0.643329
C	1.405329	4.117438	-1.003810
H	1.435066	4.911418	-1.738705
C	-3.510600	1.435826	0.101041
C	-3.439955	3.056182	-1.459105
H	-3.664097	3.801628	-2.211030
C	-4.302492	2.246251	-0.782299
H	-5.378891	2.181447	-0.873033
H	-5.085534	0.228168	0.848050
H	-1.069321	4.201729	-2.122612
H	3.567238	2.269401	1.828147
H	-0.072695	-2.702242	3.505485
H	-4.527511	-0.366670	-2.998443
H	-2.156619	0.044740	-2.372630
C	1.744795	-2.102828	-2.033510
C	1.933242	0.293131	-2.143192
H	0.959739	0.394453	-2.598962
H	2.443582	1.221089	-1.903326
C	0.372709	-2.157299	-2.370809
H	0.001905	-1.440404	-3.100595
H	-0.025435	-3.156546	-2.532337
C	2.528231	-0.923879	-1.914097
H	2.197630	-3.019248	-1.658377
C	3.934938	-1.041368	-1.435115
C	4.477615	-0.110460	-0.538316
C	4.744889	-2.094177	-1.888520
C	5.793911	-0.234097	-0.098550
H	3.859870	0.697927	-0.164581
C	6.063087	-2.216018	-1.449615
H	4.355630	-2.807703	-2.610871
C	6.591151	-1.287769	-0.550777
H	6.196998	0.491266	0.603047
H	6.679184	-3.031032	-1.817766
H	7.617513	-1.382481	-0.208795

Reaction C

^{4}IIC$_{endo\text{-}p}$

Fe	0.431100	0.324645	0.286824
N	0.318805	-0.272702	2.194808
N	0.803186	2.197599	0.886963
O	2.607265	-0.038841	0.345991
C	0.721660	1.875706	3.323107
C	0.458299	0.515135	3.318879
C	0.034484	-1.555337	4.093731
H	-0.146960	-2.434434	4.698362
C	4.669723	-0.847439	-0.512786
C	-0.141883	-2.665631	1.864625
C	0.878895	2.651915	2.186147
C	0.060731	-1.546494	2.656650
C	0.282733	-0.279012	4.503758
H	0.345352	0.104266	5.513921
C	3.230688	-0.646355	-0.532123
H	2.679081	-1.065392	-1.389039
C	0.998377	3.303904	0.089492
C	1.134110	4.067216	2.202129
H	1.238207	4.661645	3.100457
C	1.207067	4.471513	0.903148
H	1.383791	5.466101	0.514898
C	5.454462	-0.340095	0.540771
C	6.827710	-0.544084	0.532978
C	5.272606	-1.556960	-1.566521
H	4.658754	-1.945079	-2.375999
C	6.649505	-1.758718	-1.568710
H	7.120526	-2.305590	-2.379518
C	7.423445	-1.252211	-0.520098
H	8.498391	-1.408986	-0.521454
N	0.551563	0.909767	-1.625201
N	0.114692	-1.564107	-0.321537
C	0.181861	-1.243744	-2.760304
C	0.412891	0.123468	-2.753267
C	0.794478	2.204449	-3.523206
H	0.956845	3.087784	-4.126971
C	0.993311	3.309426	-1.296126
C	0.047161	-2.025473	-1.623067
C	0.788173	2.190635	-2.087337
C	0.563254	0.924702	-3.935683
H	0.499087	0.544341	-4.946954
C	-0.108697	-2.667842	0.479819
C	-0.210229	-3.438190	-1.633509
H	-0.304828	-4.037819	-2.529650
C	-0.310661	-3.834490	-0.332092
H	-0.505567	-4.825314	0.056917
O	-1.734111	0.802596	0.460581
C	-3.460947	-0.841668	0.576097

C	-2.758369	0.365654	0.994453
H	-3.172547	0.897302	1.867425
C	-3.146961	-1.460761	-0.645560
C	-3.821076	-2.615520	-1.025025
C	-4.466597	-1.374240	1.399323
H	-4.717777	-0.877298	2.333409
C	-5.132508	-2.535958	1.019752
H	-5.905632	-2.954906	1.656530
C	-4.807383	-3.156514	-0.191208
H	-5.331837	-4.059558	-0.490852
H	-0.336872	-3.608255	2.364665
H	0.099376	-1.735900	-3.723613
H	1.162722	4.256237	-1.797588
H	0.806293	2.369098	4.285529
H	7.441045	-0.156279	1.340538
H	4.967665	0.204475	1.343549
H	-2.397922	-1.008695	-1.282401
H	-3.582808	-3.096304	-1.968910
C	-3.964411	1.544550	-2.166590
C	-4.869399	1.681051	-1.008196
C	-4.642858	2.533267	0.008521
H	-3.793146	3.209416	0.017119
H	-5.330537	2.606633	0.846517
C	-6.087724	0.788849	-1.033216
H	-6.698986	0.995417	-1.921447
H	-5.795531	-0.267552	-1.085091
H	-6.715474	0.928661	-0.148810
C	-2.683078	1.931847	-2.234406
H	-2.168623	2.396426	-1.400165
H	-2.099230	1.784597	-3.137508
H	-4.401481	1.050917	-3.035198

^{4}TS1C$_{endo-p}$

Fe	0.258984	0.289747	0.209969
N	-0.139007	-0.968919	1.722776
N	0.561259	1.778315	1.529060
O	2.474709	-0.233197	0.527767
C	-0.057797	0.614384	3.602756
C	-0.265296	-0.643948	3.056177
C	-0.715476	-2.847012	2.934627
H	-0.961283	-3.882015	3.133494
C	4.640239	-0.826868	-0.258520
C	-0.404999	-3.066108	0.465940
C	0.345412	1.733528	2.887511
C	-0.408252	-2.317846	1.632679
C	-0.632936	-1.809993	3.815237
H	-0.792765	-1.820542	4.885679
C	3.227983	-0.498537	-0.410981
H	2.829065	-0.491680	-1.439769
C	0.962303	3.065251	1.248740
C	0.615322	3.020832	3.473582
H	0.524189	3.247187	4.528046
C	1.004117	3.843852	2.459694
H	1.294920	4.885163	2.510709
C	5.236070	-0.861804	1.015785
C	6.583434	-1.176683	1.135916
C	5.403784	-1.109349	-1.403537
H	4.934580	-1.080280	-2.384341
C	6.754091	-1.424215	-1.278051
H	7.348536	-1.643166	-2.159705
C	7.340725	-1.457200	-0.009559
H	8.394317	-1.702907	0.089409
N	0.814937	1.507476	-1.293960
N	0.150830	-1.247823	-1.092290
C	0.533945	-0.030217	-3.194297
C	0.815372	1.212029	-2.641206
C	1.412652	3.380449	-2.501718
H	1.716022	4.401039	-2.696771
C	1.258838	3.561228	-0.012605
C	0.235699	-1.173359	-2.466295
C	1.176063	2.834678	-1.192659
C	1.181125	2.377866	-3.399416
H	1.261085	2.409625	-4.478500
C	-0.158873	-2.557837	-0.800433
C	-0.012958	-2.467659	-3.046083
H	0.000275	-2.677894	-4.107829
C	-0.249564	-3.325792	-2.013814
H	-0.477499	-4.382935	-2.054352
O	-1.674438	0.774582	-0.071982
C	-3.421062	-0.843065	0.222907
C	-2.704380	0.403961	0.648589
H	-2.554761	0.451563	1.738314
C	-3.241429	-1.342330	-1.073094
C	-3.876097	-2.521330	-1.462309
C	-4.242501	-1.532253	1.122383
H	-4.372157	-1.152535	2.133984
C	-4.877197	-2.711480	0.732642
H	-5.508482	-3.245470	1.437186

C	-4.695550	-3.207467	-0.561510
H	-5.187660	-4.127389	-0.864258
H	-0.646852	-4.120484	0.545111
H	0.569978	-0.118155	-4.275247
H	1.560717	4.600972	-0.084570
H	-0.192573	0.724763	4.673718
H	7.051202	-1.206028	2.115377
H	4.626157	-0.640356	1.885916
H	-2.579548	-0.811554	-1.749239
H	-3.720564	-2.910886	-2.464457
C	-3.478816	2.438144	-1.697786
C	-4.352759	1.950256	-0.685315
C	-3.915536	1.809911	0.653857
H	-3.231498	2.560982	1.041445
H	-4.674615	1.513724	1.375091
C	-5.643596	1.318779	-1.105702
H	-6.026605	1.752647	-2.034186
H	-5.462310	0.248044	-1.290197
H	-6.408580	1.388494	-0.327884
C	-2.206288	2.856780	-1.456436
H	-1.825970	3.072892	-0.467717
H	-1.520145	3.046636	-2.276455
H	-3.813942	2.345231	-2.728744

^{4}IIIC$_{\text{endo-p}}$

Fe	0.298605	0.293843	0.132760
N	-0.248853	-1.455804	0.930062
N	0.255047	1.129598	1.950482
O	2.404538	-0.298543	0.503092
C	-0.369748	-0.825235	3.304761
C	-0.457028	-1.738493	2.265889
C	-0.823002	-3.678607	1.186833
H	-1.056880	-4.696900	0.906378
C	4.669188	-0.686092	-0.104368
C	-0.354331	-2.829049	-1.108148
C	-0.046960	0.514038	3.148250
C	-0.465359	-2.642941	0.258782
C	-0.811964	-3.120436	2.430195
H	-1.030594	-3.588216	3.381249
C	3.297371	-0.273924	-0.351328
H	3.057453	0.077797	-1.367986
C	0.513206	2.449088	2.260364
C	0.012017	1.466619	4.223037
H	-0.184097	1.232975	5.261413
C	0.354555	2.665647	3.672616
H	0.499218	3.617300	4.167257
C	5.065277	-1.148010	1.165547
C	6.381319	-1.537920	1.374043
C	5.600458	-0.620462	-1.155804
H	5.285700	-0.263150	-2.133559
C	6.918212	-1.012998	-0.941048
H	7.641459	-0.964764	-1.749090
C	7.305582	-1.470428	0.322163
H	8.334044	-1.777106	0.490358
N	0.915740	2.028440	-0.664322
N	0.349417	-0.543915	-1.690207
C	1.100709	1.379767	-3.030828
C	1.203751	2.290876	-1.990668
C	1.515358	4.244713	-0.916154
H	1.725604	5.270069	-0.641111
C	0.899490	3.429197	1.360482
C	0.688044	0.063033	-2.886113
C	1.097146	3.224464	0.003809
C	1.585860	3.666021	-2.149704
H	1.862519	4.121190	-3.091867
C	0.023949	-1.848098	-2.010255
C	0.561536	-0.873301	-3.967754
H	0.766579	-0.643629	-5.005378
C	0.148183	-2.055129	-3.425201
H	-0.053613	-2.992260	-3.927509
O	-1.797790	0.964922	-0.177850
C	-3.368322	-0.915353	0.142528
C	-2.848917	0.414536	0.655216
H	-2.345641	0.242398	1.608668
C	-3.342736	-1.275124	-1.207987
C	-3.854169	-2.506226	-1.622490
C	-3.909634	-1.814255	1.070410
H	-3.917013	-1.553746	2.126825
C	-4.423839	-3.042949	0.659255
H	-4.837806	-3.728239	1.393633
C	-4.398157	-3.393068	-0.692813
H	-4.798654	-4.349302	-1.017378
H	-0.581787	-3.813617	-1.501258
H	1.345228	1.724660	-4.029926
H	1.073336	4.427382	1.748009
H	-0.574476	-1.181377	4.308766
H	6.696034	-1.895198	2.349780
H	4.329425	-1.190646	1.962239

H	-2.905321	-0.608118	-1.940153
H	-3.824749	-2.770793	-2.676082
C	-3.590189	2.125320	-1.448844
C	-4.462619	1.993362	-0.446703
C	-3.953688	1.459077	0.870275
H	-3.552318	2.277749	1.484040
H	-4.761192	0.994789	1.447159
C	-5.921856	2.327309	-0.556684
H	-6.174021	2.749072	-1.534276
H	-6.534452	1.429427	-0.399481
H	-6.217072	3.049709	0.216009
C	-2.135852	1.798848	-1.318029
H	-1.539457	2.710863	-1.198541
H	-1.763249	1.287442	-2.213355
H	-3.898417	2.502184	-2.421816

^{4}TS2C$_{endo\text{-}p}$

Fe	0.875730	0.097475	-0.848899
N	1.570247	-1.787113	-0.898057
N	2.623811	0.699943	-0.070689
C	3.678991	-1.486714	0.333380
C	2.719760	-2.264039	-0.293810
C	1.677564	-4.090612	-1.094205
H	1.385621	-5.091724	-1.383677
C	-0.289211	-2.899468	-2.063132
C	3.635652	-0.104602	0.421535
C	0.922076	-2.905403	-1.389784
C	2.789932	-3.693543	-0.413956
H	3.599274	-4.301864	-0.031551
C	2.994243	1.994046	0.245703
C	4.646373	0.696120	1.052929
H	5.545992	0.302019	1.507599
C	4.249904	1.996605	0.942373
H	4.758521	2.887877	1.286409
N	0.395816	2.015961	-1.191026
N	-0.658107	-0.467793	-2.016246
C	-1.741874	1.711222	-2.369364
C	-0.763969	2.489622	-1.774171
C	0.306466	4.323063	-1.030897
H	0.619955	5.327987	-0.779794
C	2.266854	3.133921	-0.052997
C	-1.682985	0.333861	-2.485453
C	1.057133	3.136309	-0.725594
C	-0.822765	3.921975	-1.678761
H	-1.628160	4.529365	-2.070675
C	-1.018869	-1.759262	-2.354660
C	-2.689926	-0.465252	-3.124628
H	-3.590092	-0.069612	-3.576305
C	-2.278228	-1.761605	-3.045265
H	-2.769864	-2.649959	-3.419804
O	-0.080155	-0.067170	0.846885
C	-2.441639	-0.417433	1.058895
C	-1.039070	-0.935327	1.070849
H	-0.935945	-1.896543	0.546584
C	-2.676627	0.963912	1.068736
C	-3.983284	1.449347	1.016484
C	-3.523195	-1.305079	0.993852
H	-3.341375	-2.377825	0.972370
C	-4.828033	-0.818255	0.942006
H	-5.663052	-1.510568	0.886359
C	-5.060063	0.560529	0.955588
H	-6.077100	0.939540	0.913912
H	-0.688953	-3.852952	-2.391718
H	-2.608616	2.216226	-2.781728
H	2.676150	4.090094	0.255365
H	4.535565	-1.993238	0.765478
H	-1.826393	1.637963	1.090692
H	-4.161643	2.521023	1.016973
C	0.248752	0.319987	3.781605
C	-0.780682	-0.667766	3.671566
C	-0.696004	-1.724941	2.741043
H	0.289844	-2.111114	2.495280
H	-1.456036	-2.498975	2.818067
C	-2.074570	-0.405134	4.373623
H	-1.919342	0.094832	5.334947
H	-2.672511	0.274566	3.744436
H	-2.662673	-1.313384	4.522486
C	1.386846	0.310314	3.042972
H	1.691248	-0.527130	2.429704
H	2.068692	1.155381	3.056946
H	0.043461	1.175650	4.421296

^{4}TS1C$_{exo\text{-}p}$

Fe	0.126847	-0.001511	0.269261
N	-0.536935	-1.470615	-0.936494
N	0.038206	-1.246716	1.839768
O	2.223435	-0.878487	-0.124785
C	-0.950787	-3.212796	0.744952

C	-0.971067	-2.718069	-0.549808
C	-1.303719	-2.639111	-2.774469
H	-1.554792	-2.840638	-3.807722
C	4.539141	-0.745288	-0.651121
C	-0.453981	-0.310312	-3.101638
C	-0.475082	-2.523695	1.850098
C	-0.733543	-1.405251	-2.297853
C	-1.454144	-3.450306	-1.691125
H	-1.856664	-4.453804	-1.650529
C	3.210775	-0.196580	-0.403427
H	3.106742	0.899975	-0.475382
C	0.389360	-0.966982	3.140380
C	-0.446361	-3.057215	3.187764
H	-0.791518	-4.045709	3.461755
C	0.091391	-2.094348	3.986702
H	0.277952	-2.128645	5.052257
C	4.765216	-2.132591	-0.585828
C	6.037991	-2.633823	-0.826168
C	5.595411	0.128771	-0.957559
H	5.411704	1.199731	-1.005846
C	6.869693	-0.378287	-1.197230
H	7.689403	0.292994	-1.434058
C	7.088319	-1.757475	-1.131166
H	8.082354	-2.153966	-1.317987
N	0.994631	1.400441	1.429267
N	0.395297	1.189123	-1.343634
C	1.342911	3.174092	-0.241760
C	1.403190	2.661552	1.046878
C	1.787886	2.553389	3.264578
H	2.065655	2.744684	4.293116
C	0.945419	0.221514	3.588318
C	0.871499	2.483735	-1.350163
C	1.225331	1.319140	2.786196
C	1.897896	3.384904	2.187287
H	2.284322	4.395625	2.154760
C	0.067985	0.895848	-2.651220
C	0.832450	3.015506	-2.686116
H	1.158164	4.010284	-2.962391
C	0.335020	2.031576	-3.492005
H	0.169020	2.057758	-4.561319
O	-1.741368	0.664999	0.604805
C	-3.645540	-0.467567	-0.315715
C	-2.669425	0.681660	-0.331181
H	-2.307975	0.893284	-1.347832
C	-3.829821	-1.214457	0.852678
C	-4.697631	-2.304969	0.852881
C	-4.335298	-0.819009	-1.481346
H	-4.179812	-0.246069	-2.393434
C	-5.200234	-1.912720	-1.481721
H	-5.726132	-2.187516	-2.391645
C	-5.385371	-2.655468	-0.312701
H	-6.060936	-3.506167	-0.311363
H	-0.663472	-0.400083	-4.162479
H	1.702416	4.186435	-0.396664
H	1.176803	0.300978	4.645395
H	-1.334508	-4.214682	0.904934
H	6.220896	-3.703051	-0.777980
H	3.933734	-2.788290	-0.347335
H	-3.262491	-0.949048	1.738985
H	-4.833889	-2.886751	1.760377
C	-1.969885	3.613002	0.808373
C	-2.896435	3.243046	-0.205720
C	-3.744991	2.115138	-0.059313
H	-4.106859	1.884402	0.939653
H	-4.521973	2.008180	-0.813044
C	-2.756620	3.888191	-1.550145
H	-2.473227	4.942107	-1.462424
H	-1.942322	3.389164	-2.101093
H	-3.666723	3.806186	-2.149561
C	-1.816805	2.923694	1.970717
H	-2.505286	2.159232	2.303507
H	-0.984563	3.143175	2.632114
H	-1.259827	4.401165	0.572121

Reaction D

^{4}IID$_{endo\text{-}cis\text{-}p}$

Fe	1.164765	0.049487	-0.015107
N	0.710492	0.348766	1.912717
N	1.068370	2.016287	-0.348137
O	3.317853	0.302909	0.513785
C	0.519232	2.801244	1.917664
C	0.481092	1.560084	2.533665
C	0.260699	0.013133	4.154061
H	0.109991	-0.520630	5.083449
C	5.622350	-0.266840	0.389465
C	0.775481	-1.972764	2.730782
C	0.794792	3.005257	0.575340
C	0.593622	-0.609014	2.902208

C	0.193291	1.356045	3.926008
H	-0.027445	2.148717	4.628930
C	4.210307	-0.436549	0.085479
H	3.939266	-1.281056	-0.569200
C	1.291380	2.669006	-1.543939
C	0.839895	4.296070	-0.054250
H	0.657008	5.233209	0.454971
C	1.149891	4.088244	-1.365480
H	1.275010	4.820280	-2.152663
C	6.059492	0.784849	1.217235
C	7.413592	0.926227	1.489692
C	6.550902	-1.169133	-0.158302
H	6.204066	-1.978453	-0.796650
C	7.907068	-1.022517	0.117915
H	8.628625	-1.716055	-0.302515
C	8.335190	0.023873	0.940488
H	9.393610	0.139054	1.156375
N	1.654826	-0.258432	-1.937771
N	1.319308	-1.929363	0.332117
C	1.853400	-2.711502	-1.938767
C	1.873755	-1.471456	-2.559277
C	2.069762	0.072959	-4.185434
H	2.209942	0.606555	-5.116511
C	1.605185	2.062127	-2.749209
C	1.594689	-2.918007	-0.591702
C	1.764808	0.696532	-2.926890
C	2.136881	-1.269584	-3.958030
H	2.342727	-2.063339	-4.664413
C	1.113906	-2.581315	1.531342
C	1.575433	-4.206781	0.043433
H	1.763022	-5.144064	-0.464165
C	1.278063	-3.998341	1.358026
H	1.172915	-4.729637	2.148921
O	-0.900379	-0.306295	-0.606016
C	-2.948373	-1.492518	-0.420565
C	-1.744146	-0.875684	0.104913
H	-1.556141	-0.979182	1.182876
C	-3.219719	-1.467398	-1.800831
C	-4.347080	-2.114203	-2.290464
C	-3.824696	-2.149619	0.460280
H	-3.618928	-2.145020	1.527455
C	-4.953827	-2.791993	-0.034730
H	-5.641560	-3.286157	0.643415
C	-5.209995	-2.777703	-1.408573
H	-6.093343	-3.278008	-1.795041
H	0.646220	-2.610187	3.599104
H	2.048526	-3.584373	-2.552837
H	1.736326	2.701838	-3.615411
H	0.318290	3.674088	2.529576
H	7.760140	1.734249	2.126729
H	5.324444	1.469374	1.628509
H	-2.533908	-0.944453	-2.459283
H	-4.561947	-2.104289	-3.354876
C	-3.647395	2.405341	-0.612693
C	-3.899439	1.585356	0.590398
C	-2.962709	1.451684	1.554657
H	-2.033583	2.006861	1.511796
H	-3.104792	0.805601	2.415651
C	-2.462142	2.537470	-1.228024
H	-1.599791	1.993883	-0.848923
C	-5.217272	0.903618	0.678198
C	-5.921439	0.568012	-0.490196
C	-5.790211	0.561155	1.917140
C	-7.144504	-0.097367	-0.425855
H	-5.490596	0.799960	-1.458614
C	-7.009574	-0.108540	1.982634
H	-5.291196	0.852134	2.836729
C	-7.693161	-0.441865	0.809995
H	-7.666668	-0.351396	-1.344196
H	-7.437727	-0.353557	2.950852
H	-8.650086	-0.953734	0.861840
H	-4.514593	2.915878	-1.031900
C	-2.227296	3.383241	-2.443319
H	-3.153726	3.831193	-2.817614
H	-1.520546	4.193391	-2.220072
H	-1.775304	2.791947	-3.251072

^{4}TS1D$_{endo\text{-}cis\text{-}p}$

Fe	0.988330	0.329026	0.051493
N	0.873722	0.765708	2.010436
N	1.267946	2.268440	-0.365405
O	3.275005	0.203357	0.372039
C	0.887129	3.219253	1.870709
C	0.791484	2.022123	2.568029
C	0.670834	0.589992	4.302318
H	0.600181	0.122114	5.275748
C	5.378823	-0.878266	0.110100
C	0.797593	-1.508754	2.943915

C	1.152190	3.324955	0.512326
C	0.802115	-0.124392	3.059612
C	0.649467	1.918598	3.996588
H	0.565536	2.762710	4.668855
C	3.937929	-0.737764	-0.067074
H	3.428743	-1.537731	-0.631747
C	1.594050	2.817743	-1.585430
C	1.394617	4.565120	-0.177048
H	1.364584	5.542442	0.287179
C	1.686121	4.249477	-1.471520
H	1.935056	4.915970	-2.287249
C	6.113927	0.091496	0.816410
C	7.484745	-0.066109	0.975274
C	6.027620	-2.000329	-0.431708
H	5.451211	-2.745119	-0.975804
C	7.401806	-2.154274	-0.269533
H	7.907753	-3.019837	-0.686039
C	8.127084	-1.187571	0.433060
H	9.199418	-1.306771	0.560133
N	1.281337	-0.130683	-1.880655
N	0.911504	-1.640209	0.491581
C	0.890035	-2.557890	-1.787155
C	1.132775	-1.371096	-2.464486
C	1.606472	0.035193	-4.159517
H	1.817478	0.493154	-5.117256
C	1.747686	2.112320	-2.771120
C	0.809772	-2.680102	-0.406774
C	1.564869	0.743096	-2.906819
C	1.324713	-1.270510	-3.887412
H	1.262197	-2.103393	-4.575849
C	0.819032	-2.207044	1.744132
C	0.655256	-3.925579	0.296821
H	0.550597	-4.891512	-0.179825
C	0.674780	-3.634624	1.628527
H	0.586411	-4.313100	2.467138
O	-1.014002	0.383555	-0.187963
C	-2.683581	-1.313564	0.036141
C	-1.831886	-0.233658	0.620448
H	-1.434084	-0.485478	1.612110
C	-2.707652	-1.501613	-1.350925
C	-3.494051	-2.510975	-1.905571
C	-3.449507	-2.146352	0.860628
H	-3.429490	-2.003562	1.938894
C	-4.242439	-3.148506	0.305085
H	-4.840007	-3.786480	0.949896
C	-4.267267	-3.331600	-1.080475
H	-4.883299	-4.114736	-1.513462
H	0.726774	-2.087312	3.859142
H	0.791306	-3.462479	-2.377836
H	1.990524	2.678201	-3.664593
H	0.812228	4.139669	2.440392
H	8.059608	0.677527	1.518961
H	5.590694	0.949559	1.226475
H	-2.093063	-0.858633	-1.972173
H	-3.504295	-2.658640	-2.982279
C	-3.472797	2.197804	-0.781364
C	-3.873108	1.413487	0.348154
C	-2.973704	1.105225	1.392513
H	-2.186723	1.812354	1.638097
H	-3.383708	0.591592	2.257476
C	-2.233347	2.729845	-0.956264
H	-1.495337	2.674800	-0.164511
C	-5.178593	0.737583	0.315136
C	-5.704558	0.238435	-0.893421
C	-5.910921	0.533347	1.502432
C	-6.908015	-0.458848	-0.907881
H	-5.136469	0.339516	-1.811147
C	-7.128776	-0.138170	1.479971
H	-5.541062	0.939977	2.438774
C	-7.626603	-0.643565	0.275884
H	-7.284016	-0.863195	-1.842754
H	-7.691531	-0.266768	2.399772
H	-8.571869	-1.178409	0.260584
H	-4.203186	2.330924	-1.575487
C	-1.792673	3.403868	-2.208314
H	-2.608722	3.534829	-2.925040
H	-1.336668	4.376852	-1.987503
H	-1.003606	2.801338	-2.678621

^{6}TS1D$_{endo\text{-}cis\text{-}p}$

Fe	0.945081	0.242269	-0.002955
N	0.817702	0.737380	2.004372
N	1.287851	2.217380	-0.468144
O	3.190306	0.187836	0.406055
C	0.940425	3.185037	1.770315
C	0.781332	2.012034	2.515817
C	0.550151	0.619992	4.282465
H	0.436187	0.186342	5.267798

C	5.362604	-0.763924	0.239228
C	0.677433	-1.529393	2.955566
C	1.211172	3.280103	0.402539
C	0.690956	-0.134060	3.059168
C	0.600449	1.943701	3.946717
H	0.537180	2.800915	4.604817
C	3.923419	-0.723416	0.014647
H	3.483756	-1.568138	-0.540592
C	1.624537	2.718482	-1.702760
C	1.486280	4.500894	-0.317023
H	1.493733	5.490310	0.122217
C	1.750975	4.153540	-1.612475
H	2.011851	4.804993	-2.436830
C	6.010292	0.268600	0.942486
C	7.383167	0.207046	1.143108
C	6.100252	-1.851867	-0.257260
H	5.590856	-2.645474	-0.799082
C	7.476303	-1.908771	-0.053911
H	8.050887	-2.747014	-0.435769
C	8.114468	-0.880095	0.645284
H	9.188220	-0.923874	0.804595
N	1.389006	-0.291494	-1.947396
N	0.955336	-1.774655	0.521611
C	1.106521	-2.729301	-1.741058
C	1.322815	-1.559982	-2.475483
C	1.768228	-0.180696	-4.210824
H	1.979827	0.247987	-5.182096
C	1.785798	1.958131	-2.864756
C	0.944313	-2.832212	-0.355808
C	1.656895	0.571477	-2.983032
C	1.554838	-1.493287	-3.899165
H	1.560764	-2.346220	-4.565785
C	0.783211	-2.287202	1.785030
C	0.768276	-4.060712	0.382411
H	0.723121	-5.047765	-0.059604
C	0.675101	-3.725437	1.703096
H	0.537788	-4.385163	2.550296
O	-1.001161	0.218331	-0.280536
C	-2.798661	-1.339690	-0.055454
C	-1.847855	-0.358282	0.531635
H	-1.459837	-0.645866	1.514797
C	-2.867914	-1.499772	-1.445372
C	-3.756014	-2.422535	-1.996726
C	-3.622364	-2.112283	0.773975
H	-3.568171	-1.989333	1.853231
C	-4.515594	-3.026548	0.220670
H	-5.157089	-3.616869	0.868340
C	-4.584863	-3.182016	-1.166901
H	-5.279120	-3.897422	-1.598526
H	0.552638	-2.082647	3.881544
H	1.080559	-3.657859	-2.303186
H	2.028081	2.503016	-3.772252
H	0.899690	4.119252	2.322222
H	7.891088	0.999267	1.684789
H	5.419274	1.098366	1.317111
H	-2.209170	-0.908263	-2.072446
H	-3.800983	-2.551683	-3.074711
C	-3.392935	2.234849	-0.776031
C	-3.784825	1.459273	0.367520
C	-2.868897	1.099574	1.375081
H	-2.011037	1.734576	1.573280
H	-3.264804	0.604268	2.256722
C	-2.140224	2.706033	-1.006626
H	-1.362110	2.582362	-0.262171
C	-5.127997	0.861411	0.384220
C	-5.732146	0.411512	-0.807193
C	-5.819862	0.680013	1.599474
C	-6.971991	-0.218088	-0.778728
H	-5.198708	0.493372	-1.747347
C	-7.072752	0.076715	1.620882
H	-5.389164	1.052411	2.523891
C	-7.648285	-0.382105	0.432678
H	-7.409421	-0.586301	-1.701667
H	-7.602616	-0.034425	2.562205
H	-8.621813	-0.863457	0.451232
H	-4.156161	2.424770	-1.526328
C	-1.732966	3.398698	-2.259685
H	-2.575838	3.581084	-2.932687
H	-1.231706	4.347494	-2.031247
H	-0.986687	2.785757	-2.783641

^{4}IIID$_{endo\text{-}cis\text{-}p}$

Fe	1.016353	0.437242	-0.018436
N	0.672268	1.050928	1.844859
N	1.241978	2.319284	-0.599806
O	3.195546	0.395130	0.462754
C	0.509083	3.469789	1.446078
C	0.435374	2.345423	2.256802

C	0.331675	1.091435	4.125733
H	0.236865	0.722383	5.138713
C	5.350555	-0.606139	0.401281
C	0.706503	-1.116781	3.006812
C	0.945501	3.454090	0.129216
C	0.611577	0.268847	2.980901
C	0.196442	2.372221	3.673934
H	-0.019701	3.266476	4.243892
C	3.920432	-0.554909	0.146743
H	3.471518	-1.424167	-0.361817
C	1.774021	2.760676	-1.795583
C	1.258589	4.625796	-0.641185
H	1.108142	5.642265	-0.301573
C	1.797778	4.196941	-1.819147
H	2.167392	4.791241	-2.644609
C	6.006694	0.460866	1.044634
C	7.374404	0.388107	1.272057
C	6.075394	-1.739113	-0.008570
H	5.560238	-2.558213	-0.505009
C	7.446221	-1.806442	0.221885
H	8.011139	-2.678419	-0.092556
C	8.092213	-0.743760	0.861011
H	9.162248	-0.795686	1.041089
N	1.448216	-0.187572	-1.856755
N	0.868035	-1.460029	0.576349
C	0.949790	-2.582065	-1.607924
C	1.302047	-1.467683	-2.355356
C	2.111529	-0.243631	-4.065441
H	2.486355	0.113003	-5.016013
C	2.149902	1.946846	-2.855180
C	0.799178	-2.579364	-0.228845
C	1.943248	0.574795	-2.896866
C	1.687131	-1.499328	-3.739120
H	1.656946	-2.382094	-4.364527
C	0.778850	-1.919688	1.877225
C	0.648641	-3.756635	0.579292
H	0.547870	-4.759968	0.187682
C	0.664991	-3.351654	1.882055
H	0.578097	-3.957191	2.774700
O	-1.281573	0.461990	-0.397217
C	-2.431240	-1.628565	0.173905
C	-2.024092	-0.241132	0.632094
H	-1.325092	-0.349564	1.457519
C	-2.493486	-1.989283	-1.173534
C	-2.872356	-3.281016	-1.544208
C	-2.746496	-2.585350	1.146834
H	-2.683318	-2.320622	2.200453
C	-3.129027	-3.874581	0.780660
H	-3.372417	-4.604710	1.547577
C	-3.192854	-4.227146	-0.569802
H	-3.491326	-5.231068	-0.858392
H	0.638650	-1.608790	3.971271
H	0.860973	-3.531853	-2.123693
H	2.550278	2.425253	-3.742567
H	0.303318	4.433799	1.898888
H	7.889027	1.205801	1.767344
H	5.426924	1.325330	1.352181
H	-2.226134	-1.262689	-1.930346
H	-2.918483	-3.546352	-2.597144
C	-3.475083	1.388536	-1.108234
C	-4.104436	0.959411	-0.004752
C	-3.201615	0.602425	1.149490
H	-2.793092	1.501586	1.632116
H	-3.724459	0.026009	1.916788
C	-1.979395	1.512013	-1.171059
H	-1.655360	2.455256	-0.708633
C	-5.565096	0.769170	0.090536
C	-6.316062	0.363971	-1.026503
C	-6.237695	0.989686	1.304545
C	-7.696834	0.203964	-0.936654
H	-5.805419	0.140392	-1.958698
C	-7.620131	0.833600	1.393006
H	-5.680309	1.310004	2.180969
C	-8.354862	0.440930	0.272681
H	-8.259159	-0.119406	-1.808218
H	-8.124018	1.020040	2.337310
H	-9.431071	0.311771	0.343278
H	-4.022751	1.680046	-2.000265
C	-1.486097	1.459879	-2.609817
H	-1.790717	0.527033	-3.093772
H	-1.923936	2.294792	-3.167608
H	-0.405105	1.543871	-2.663600

^{4}VD$_{endo\text{-}cis\text{-}p}$

Fe	-2.057651	0.086884	0.362440
N	-1.452065	-0.166356	2.251635
N	-2.247138	-1.891953	0.180199
C	-1.413025	-2.624244	2.377150

C	-1.202995	-1.362891	2.904989
C	-0.572822	0.236194	4.356109
H	-0.218837	0.799816	5.209444
C	-1.199192	2.185258	2.936338
C	-1.894767	-2.863891	1.102895
C	-1.075916	0.822709	3.148058
C	-0.662032	-1.116789	4.209570
H	-0.389060	-1.890853	4.914738
C	-2.658573	-2.589629	-0.944296
C	-2.090107	-4.172437	0.549747
H	-1.891590	-5.095846	1.077716
C	-2.557908	-4.002556	-0.719734
H	-2.825907	-4.758016	-1.446679
N	-3.029953	0.319733	-1.365883
N	-2.243776	2.048638	0.712646
C	-3.273087	2.768001	-1.403485
C	-3.404445	1.510187	-1.964245
C	-3.882428	-0.080151	-3.480726
H	-4.198543	-0.642989	-4.349253
C	-3.132602	-2.021331	-2.112843
C	-2.727465	3.012882	-0.155869
C	-3.320475	-0.662721	-2.295947
C	-3.941275	1.265371	-3.272309
H	-4.312494	2.035530	-3.935698
C	-1.769469	2.750123	1.808418
C	-2.563826	4.321155	0.408843
H	-2.873186	5.239197	-0.073342
C	-1.963358	4.159215	1.622030
H	-1.681845	4.916707	2.341818
O	-0.211727	0.309080	-0.528612
C	1.885210	1.378766	-0.807891
C	0.764833	0.907869	-0.027857
H	0.746436	1.155816	1.040852
C	1.935077	1.156642	-2.198187
C	2.992739	1.670408	-2.935924
C	2.912647	2.096972	-0.167749
H	2.878181	2.243581	0.908353
C	3.969008	2.606883	-0.912408
H	4.770284	3.148923	-0.421720
C	4.004462	2.397523	-2.294194
H	4.831160	2.795230	-2.875770
H	-0.865674	2.852509	3.723843
H	-3.611683	3.619406	-1.983898
H	-3.411619	-2.686348	-2.922966
H	-1.173925	-3.480457	2.998411
H	1.136918	0.592692	-2.669340
H	3.037585	1.509695	-4.008769
C	2.406749	-2.506577	-0.659318
C	2.835664	-1.580874	0.408848
C	2.025487	-1.301842	1.455136
H	1.082213	-1.818373	1.580787
H	2.295397	-0.587307	2.226459
C	1.149449	-2.644598	-1.108484
H	0.360467	-2.025929	-0.686289
C	4.177000	-0.958813	0.267472
C	4.893061	-0.485337	1.383471
C	4.760151	-0.809719	-1.002392
C	6.132407	0.130990	1.231703
H	4.490381	-0.632209	2.381224
C	6.003265	-0.198365	-1.154933
H	4.220645	-1.144467	-1.882101
C	6.693880	0.278230	-0.039902
H	6.671538	0.478794	2.108561
H	6.430805	-0.090854	-2.147933
H	7.666436	0.747961	-0.156877
H	3.195080	-3.099625	-1.122444
C	0.734056	-3.596949	-2.188807
H	1.588357	-4.132231	-2.615614
H	0.024553	-4.338083	-1.798222
H	0.214947	-3.068331	-2.999679

^{4}TS2D$_{endo\text{-}cis\text{-}p}$

Fe	1.932589	0.231153	-0.323620
N	1.604734	-0.207810	-2.259230
N	2.587607	-1.648883	-0.040344
C	1.945794	-2.644255	-2.198272
C	1.589144	-1.466054	-2.834517
C	0.962577	-0.061145	-4.477045
H	0.656165	0.396325	-5.409033
C	1.071854	2.033906	-3.127130
C	2.441509	-2.717768	-0.906339
C	1.223332	0.662858	-3.265046
C	1.179983	-1.380255	-4.208069
H	1.092719	-2.228295	-4.875093
C	3.149655	-2.175736	1.107509
C	2.907167	-3.926844	-0.286570
H	2.901089	-4.899397	-0.761689
C	3.355106	-3.589294	0.956659

H	3.790602	-4.228616	1.713804
N	2.677625	0.734707	1.467134
N	1.701885	2.177631	-0.754331
C	2.138881	3.134858	1.468590
C	2.593885	1.974172	2.071884
C	3.473177	0.616162	3.635443
H	3.901079	0.178982	4.528441
C	3.444933	-1.463536	2.258628
C	1.746853	3.226720	0.143610
C	3.211745	-0.107795	2.421949
C	3.081333	1.903542	3.421774
H	3.122409	2.744116	4.102507
C	1.289184	2.732282	-1.950706
C	1.358812	4.450957	-0.500503
H	1.316719	5.413922	-0.008349
C	1.083237	4.146540	-1.799446
H	0.764904	4.806933	-2.595696
O	0.073637	0.003624	0.314427
C	-2.023347	1.152917	0.246925
C	-0.961137	0.340669	-0.408697
H	-0.758805	0.639328	-1.442876
C	-2.005333	1.350921	1.633403
C	-2.999292	2.117458	2.241580
C	-3.044677	1.726311	-0.522008
H	-3.059836	1.570368	-1.598175
C	-4.040954	2.485496	0.088169
H	-4.832823	2.923640	-0.512679
C	-4.020318	2.681388	1.472141
H	-4.796026	3.275232	1.947730
H	0.754136	2.596878	-3.998312
H	2.123818	4.041774	2.063630
H	3.879696	-2.005638	3.091619
H	1.883532	-3.565671	-2.767599
H	-1.200304	0.913112	2.214328
H	-2.977108	2.277438	3.316304
C	-1.881131	-2.394241	1.191176
C	-2.498288	-1.795185	0.039190
C	-1.743885	-1.333979	-1.057060
H	-0.797272	-1.811954	-1.289224
H	-2.290241	-0.995558	-1.932437
C	-0.547599	-2.601169	1.341325
H	0.133359	-2.376298	0.529047
C	-3.936372	-1.487735	0.091890
C	-4.721547	-1.524992	-1.079354
C	-4.552981	-1.106222	1.301241
C	-6.072334	-1.195469	-1.041606
H	-4.278472	-1.852904	-2.014480
C	-5.898398	-0.753090	1.330226
H	-3.963335	-1.025164	2.207398
C	-6.662928	-0.800441	0.161871
H	-6.665974	-1.248697	-1.949621
H	-6.349838	-0.433217	2.264631
H	-7.715293	-0.532205	0.189075
H	-2.536815	-2.675460	2.011113
C	0.074795	-3.133345	2.583616
H	-0.663958	-3.383015	3.351367
H	0.677093	-4.023503	2.358295
H	0.774801	-2.392578	2.993477

[6]TS2D$_{endo\text{-}cis\text{-}p}$

Fe	1.805289	0.175714	-0.278751
N	1.563426	-0.658614	-2.171919
N	2.690603	-1.615775	0.298857
C	2.082175	-3.005997	-1.640201
C	1.602809	-2.004541	-2.481221
C	0.779475	-0.982750	-4.317178
H	0.391374	-0.742229	-5.298599
C	0.905541	1.362224	-3.414434
C	2.619401	-2.822963	-0.368143
C	1.074311	-0.016071	-3.291395
C	1.105125	-2.210858	-3.816658
H	1.035194	-3.173156	-4.307268
C	3.301896	-1.875756	1.509526
C	3.188182	-3.864165	0.447845
H	3.265553	-4.903693	0.156134
C	3.613092	-3.278134	1.605962
H	4.103927	-3.744181	2.450645
N	2.785136	1.076806	1.304153
N	1.709630	2.039377	-1.190471
C	2.329792	3.428863	0.744261
C	2.775184	2.423774	1.599164
C	3.684850	1.416152	3.401038
H	4.145905	1.183772	4.352288
C	3.580748	-0.925215	2.489163
C	1.850839	3.254023	-0.551035
C	3.347005	0.443754	2.392794
C	3.329787	2.639026	2.911553
H	3.442720	3.607177	3.381985

C	1.213475	2.316355	-2.447266
C	1.436960	4.317664	-1.430321
H	1.455327	5.369593	-1.176456
C	1.044064	3.739089	-2.601630
H	0.676988	4.223060	-3.497466
O	-0.012685	0.086627	0.350248
C	-2.111332	1.232103	0.310605
C	-1.048879	0.453339	-0.364776
H	-0.846759	0.753486	-1.398306
C	-2.088328	1.401335	1.701940
C	-3.090825	2.139473	2.329191
C	-3.148469	1.800959	-0.442310
H	-3.168615	1.666299	-1.521242
C	-4.154053	2.528637	0.188465
H	-4.957643	2.963303	-0.398385
C	-4.126240	2.699121	1.575787
H	-4.907798	3.271527	2.067034
H	0.516131	1.727504	-4.359644
H	2.385083	4.448040	1.113659
H	4.052111	-1.281537	3.399821
H	2.072194	-4.019736	-2.028409
H	-1.273797	0.964640	2.270174
H	-3.064388	2.280884	3.405996
C	-1.913195	-2.350702	1.169748
C	-2.568609	-1.724543	0.051059
C	-1.855138	-1.257503	-1.066218
H	-0.908343	-1.713167	-1.336665
H	-2.425411	-0.882006	-1.910268
C	-0.579526	-2.580124	1.262498
H	0.069194	-2.361166	0.422411
C	-3.997121	-1.398994	0.167500
C	-4.830189	-1.402389	-0.971128
C	-4.556640	-1.029726	1.408011
C	-6.172588	-1.054237	-0.871266
H	-4.430869	-1.721996	-1.928735
C	-5.892263	-0.653174	1.498823
H	-3.928442	-0.972300	2.289452
C	-6.705099	-0.669241	0.362609
H	-6.805655	-1.085192	-1.752909
H	-6.299485	-0.342636	2.456096
H	-7.751024	-0.386430	0.438347
H	-2.540543	-2.634574	2.010529
C	0.094840	-3.130835	2.469804
H	-0.609687	-3.386057	3.266466
H	0.687830	-4.016326	2.208426
H	0.815002	-2.394881	2.853088

^{4}VID$_{endo\text{-}cis\text{-}p}$

Fe	-1.885777	-0.011179	0.278968
N	-1.644766	-0.816215	2.085311
N	-2.734885	-1.698040	-0.325604
C	-1.997519	-3.176955	1.496977
C	-1.615500	-2.171027	2.370257
C	-1.066420	-1.148735	4.299016
H	-0.796550	-0.905214	5.318334
C	-1.114285	1.185696	3.411669
C	-2.586343	-2.943540	0.264886
C	-1.300407	-0.180492	3.267011
C	-1.233719	-2.381155	3.736873
H	-1.139786	-3.352200	4.205254
C	-3.503213	-1.914816	-1.459212
C	-3.237337	-3.946495	-0.526379
H	-3.261386	-5.000328	-0.281699
C	-3.831216	-3.305602	-1.574794
H	-4.430364	-3.729653	-2.369892
N	-2.568957	0.885762	-1.351214
N	-1.471156	1.771930	1.049389
C	-1.583140	3.111757	-1.007719
C	-2.239236	2.163432	-1.773748
C	-3.500505	1.300965	-3.426213
H	-4.072404	1.125308	-4.327915
C	-3.829471	-0.948729	-2.396646
C	-1.289084	2.943657	0.334708
C	-3.346386	0.349142	-2.364188
C	-2.788917	2.410862	-3.075347
H	-2.669894	3.335120	-3.625439
C	-1.162036	2.088760	2.362926
C	-0.847369	3.993145	1.205365
H	-0.620520	5.000455	0.883240
C	-0.798987	3.472583	2.465070
H	-0.521723	3.966323	3.387160
O	0.145653	-0.419842	-0.330491
C	1.719265	1.379193	0.226446
C	1.143190	0.039466	0.634948
H	0.594724	0.169429	1.565717
C	1.714962	1.832946	-1.094712
C	2.267998	3.073516	-1.417077
C	2.281625	2.187866	1.221687

H	2.275779	1.850151	2.256070
C	2.838908	3.424844	0.901921
H	3.272750	4.040797	1.684516
C	2.831861	3.872441	-0.421381
H	3.264754	4.836259	-0.673545
H	-0.850218	1.561349	4.394369
H	-1.376331	4.077021	-1.455987
H	-4.435150	-1.246475	-3.245538
H	-1.932987	-4.202714	1.843511
H	1.266005	1.221576	-1.867662
H	2.259315	3.414147	-2.448896
C	2.013799	-1.617324	-1.448231
C	2.858583	-1.412724	-0.426403
C	2.203779	-1.044448	0.881898
H	1.710500	-1.911995	1.342421
H	2.921995	-0.651326	1.605553
C	0.526979	-1.504388	-1.283276
H	0.115317	-2.414109	-0.825844
C	4.328200	-1.460558	-0.548709
C	5.120587	-1.901827	0.524837
C	4.967749	-1.069692	-1.737993
C	6.507418	-1.973610	0.404568
H	4.649581	-2.216927	1.452322
C	6.353864	-1.136753	-1.855662
H	4.374549	-0.678455	-2.559357
C	7.128775	-1.591796	-0.785972
H	7.102965	-2.328907	1.240704
H	6.831770	-0.821039	-2.778725
H	8.210007	-1.639932	-0.877015
H	2.370505	-1.885811	-2.438571
C	-0.160037	-1.237131	-2.613070
H	0.074597	-2.055398	-3.301955
H	-1.239528	-1.184887	-2.506672
H	0.196564	-0.302079	-3.054859

[4]TS1D$_{endo\text{-}trans\text{-}p}$

Fe	0.907186	0.380677	0.091783
N	1.145576	0.841342	2.038401
N	1.375957	2.270920	-0.410577
O	3.211495	0.049455	0.100044
C	1.749521	3.214059	1.829773
C	1.474203	2.070141	2.565203
C	1.155418	0.725225	4.343555
H	1.067121	0.292346	5.331674
C	5.215735	-1.174335	-0.281068
C	0.660854	-1.352207	3.035731
C	1.701915	3.300012	0.445394
C	0.958649	0.002709	3.113559
C	1.484179	2.003496	4.003289
H	1.717101	2.835277	4.655545
C	3.767346	-0.995820	-0.236397
H	3.153085	-1.865672	-0.526528
C	1.391700	2.807482	-1.677175
C	1.930189	4.508597	-0.301819
H	2.206891	5.458975	0.136193
C	1.725445	4.206920	-1.616013
H	1.809570	4.857254	-2.477285
C	6.079022	-0.119329	0.065979
C	7.453893	-0.310696	0.015836
C	5.741137	-2.415574	-0.675810
H	5.066187	-3.225897	-0.942071
C	7.120043	-2.603210	-0.724058
H	7.530874	-3.561022	-1.028308
C	7.973145	-1.551179	-0.378623
H	9.049124	-1.696586	-0.416112
N	0.887305	-0.125097	-1.855374
N	0.706292	-1.564985	0.586052
C	0.567242	-2.556118	-1.658369
C	0.707674	-1.383847	-2.387372
C	0.793517	0.005101	-4.158360
H	0.807463	0.451308	-5.144435
C	1.161666	2.105264	-2.852665
C	0.542453	-2.629878	-0.273048
C	0.945986	0.737183	-2.928058
C	0.655906	-1.308942	-3.824225
H	0.528665	-2.161154	-4.479351
C	0.560286	-2.077067	1.857916
C	0.301038	-3.835302	0.474482
H	0.135821	-4.809022	0.032310
C	0.301102	-3.491355	1.792767
H	0.144187	-4.126920	2.654493
O	-1.061555	0.718495	0.142753
C	-2.556469	-1.094173	0.677343
C	-1.917586	0.223884	1.007991
H	-1.597040	0.272011	2.061776
C	-2.517590	-1.580106	-0.633486
C	-3.109310	-2.802896	-0.945401
C	-3.198145	-1.842646	1.670874

H	-3.221852	-1.471142	2.693623
C	-3.797896	-3.061645	1.358198
H	-4.292938	-3.637681	2.134945
C	-3.755123	-3.543834	0.047108
H	-4.219596	-4.495096	-0.197235
H	0.528440	-1.890697	3.968343
H	0.424804	-3.478148	-2.212130
H	1.198720	2.659972	-3.784639
H	2.008159	4.113371	2.379131
H	8.127115	0.498719	0.281881
H	5.649371	0.830546	0.368330
H	-1.998740	-0.997454	-1.387409
H	-3.066422	-3.178739	-1.964320
C	-3.315314	2.195510	-1.131766
C	-3.923954	1.523770	-0.023943
C	-3.281273	1.443837	1.244329
H	-2.687646	2.287490	1.569810
H	-3.859798	0.996738	2.048609
C	-2.288861	3.094490	-1.129950
C	-5.113944	0.698856	-0.263357
C	-5.324049	0.034600	-1.491831
C	-6.078422	0.535539	0.755058
C	-6.442060	-0.767819	-1.684405
H	-4.578344	0.094400	-2.276131
C	-7.208827	-0.247073	0.550098
H	-5.962893	1.065106	1.695103
C	-7.390851	-0.907445	-0.667849
H	-6.571912	-1.291930	-2.626385
H	-7.949889	-0.340886	1.338062
H	-8.268776	-1.527329	-0.824689
H	-3.717743	1.952678	-2.110861
C	-1.638581	3.827763	0.000292
H	-2.374609	4.180195	0.731760
H	-0.929089	3.174997	0.515050
H	-1.080541	4.688026	-0.375565
H	-1.933205	3.377865	-2.119769

[4]TS1D$_{exo\text{-}cis\text{-}p}$

Fe	-0.633173	0.271684	0.208589
N	-0.375942	1.242582	-1.537116
N	-1.182791	1.995990	1.085854
O	-2.852254	0.118673	-0.452570
C	-0.905519	3.529835	-0.813472
C	-0.507716	2.594680	-1.756651
C	0.201890	1.741318	-3.715422
H	0.528450	1.578220	-4.734316
C	-4.921008	-1.003559	-0.801053
C	0.284402	-0.644194	-2.966297
C	-1.206412	3.244606	0.509922
C	0.051709	0.702786	-2.728894
C	-0.149853	2.912046	-3.114319
H	-0.164499	3.908688	-3.535369
C	-3.533380	-0.902752	-0.360165
H	-3.088277	-1.812410	0.079223
C	-1.500512	2.179415	2.411361
C	-1.552403	4.236534	1.496043
H	-1.643057	5.296202	1.295386
C	-1.727120	3.578245	2.675688
H	-1.995241	3.985500	3.642008
C	-5.572955	0.102943	-1.375362
C	-6.893235	-0.015777	-1.790168
C	-5.601675	-2.222602	-0.647670
H	-5.089910	-3.073013	-0.202738
C	-6.925085	-2.337063	-1.064909
H	-7.455482	-3.277199	-0.948560
C	-7.567840	-1.234318	-1.634947
H	-8.600435	-1.322798	-1.960782
N	-1.101397	-0.733171	1.892362
N	-0.308796	-1.488460	-0.732754
C	-0.751343	-3.043786	1.120876
C	-1.063723	-2.097649	2.088088
C	-1.614056	-1.219200	4.089143
H	-1.888568	-1.049624	5.122379
C	-1.609751	1.173957	3.361187
C	-0.403957	-2.752856	-0.191922
C	-1.440262	-0.180858	3.108799
C	-1.389719	-2.407302	3.455010
H	-1.440167	-3.408363	3.864083
C	0.088779	-1.661421	-2.041134
C	-0.074156	-3.739803	-1.186962
H	-0.084704	-4.809366	-1.018891
C	0.244016	-3.062698	-2.327897
H	0.545377	-3.463295	-3.287089
O	1.257878	0.533118	0.753009
C	2.645599	2.094899	-0.428819
C	2.286468	0.690345	-0.038598
H	2.364800	-0.016366	-0.879165
C	2.277466	3.167259	0.392228

C	2.571806	4.473069	0.008374
C	3.314909	2.340118	-1.633329
H	3.589720	1.506047	-2.275451
C	3.606242	3.648346	-2.019095
H	4.116139	3.834950	-2.959951
C	3.237336	4.716579	-1.197405
H	3.466928	5.735571	-1.495962
H	0.612007	-0.928690	-3.960821
H	-0.779426	-4.088643	1.413141
H	-1.873053	1.465699	4.372608
H	-0.957060	4.566838	-1.127367
H	-7.403606	0.833549	-2.234299
H	-5.026409	1.034517	-1.483394
H	1.734590	2.964254	1.310014
H	2.277228	5.302946	0.644867
C	2.642554	-1.658634	2.139260
C	3.577473	-1.174907	1.167146
C	3.815919	0.201503	0.943248
H	3.706726	0.901080	1.761020
H	4.621668	0.454988	0.259889
C	2.003977	-0.996426	3.146709
C	4.124116	-2.133450	0.190790
C	3.327086	-3.175567	-0.323440
C	5.445115	-1.998287	-0.283512
C	3.830962	-4.042645	-1.287964
H	2.294931	-3.273336	-0.008523
C	5.955155	-2.887010	-1.224778
H	6.084820	-1.219708	0.120830
C	5.147751	-3.908162	-1.734700
H	3.191799	-4.821189	-1.693812
H	6.982887	-2.785677	-1.560349
H	5.544567	-4.595115	-2.476587
H	2.378479	-2.707701	2.043244
C	2.166785	0.382135	3.703750
H	1.842999	0.401455	4.748477
H	3.197235	0.744904	3.655678
H	1.532907	1.078133	3.143152
H	1.260594	-1.595882	3.669745

[4]IID$_{exo\text{-}trans\text{-}p}$

Fe	-0.938990	0.372478	0.343591
N	-1.201036	2.156633	1.220736
N	-0.944667	-0.512304	2.135250
O	-3.153945	0.153327	0.289834
C	-1.256007	1.455305	3.576260
C	-1.307911	2.408989	2.571760
C	-1.450832	4.424334	1.580045
H	-1.542984	5.477427	1.348185
C	-5.233000	-0.407530	-0.715548
C	-1.219555	3.605033	-0.767108
C	-1.081057	0.097293	3.364266
C	-1.285156	3.385031	0.600009
C	-1.467980	3.819233	2.801059
H	-1.575415	4.273844	3.777264
C	-3.789481	-0.231005	-0.697374
H	-3.247603	-0.460913	-1.629424
C	-0.789284	-1.856165	2.396004
C	-1.009990	-0.883588	4.413676
H	-1.090320	-0.659222	5.469343
C	-0.827482	-2.093412	3.814014
H	-0.728354	-3.067184	4.275795
C	-6.005547	-0.135915	0.429806
C	-7.381785	-0.314670	0.387623
C	-5.850877	-0.857037	-1.895628
H	-5.246233	-1.064501	-2.775488
C	-7.230738	-1.034892	-1.932112
H	-7.713226	-1.382153	-2.840416
C	-7.992641	-0.763565	-0.791562
H	-9.069740	-0.902143	-0.819432
N	-0.723304	-1.410506	-0.548627
N	-0.959073	1.257341	-1.463993
C	-0.717328	-0.718946	-2.911015
C	-0.648892	-1.669327	-1.904345
C	-0.436419	-3.674932	-0.903045
H	-0.308230	-4.722477	-0.667314
C	-0.626565	-2.849202	1.444216
C	-0.849936	0.645914	-2.699147
C	-0.596653	-2.635941	0.075731
C	-0.472340	-3.077889	-2.128633
H	-0.379388	-3.536444	-3.104566
C	-1.074215	2.611551	-1.723331
C	-0.890457	1.630838	-3.742659
H	-0.823930	1.408233	-4.799833
C	-1.024704	2.847566	-3.138450
H	-1.092721	3.824036	-3.600257
O	1.206708	0.676591	0.564266
C	3.271600	1.803760	0.261140
C	1.932994	1.399754	-0.129497

H	1.548071	1.789122	-1.083406
C	3.837634	1.340082	1.462934
C	5.110871	1.757404	1.827027
C	3.990870	2.679636	-0.568307
H	3.549760	3.018710	-1.501462
C	5.265882	3.096883	-0.197268
H	5.826164	3.773482	-0.835104
C	5.823028	2.635608	0.998246
H	6.818455	2.960137	1.288372
H	-1.297090	4.629479	-1.115609
H	-0.645711	-1.065527	-3.936681
H	-0.519723	-3.868664	1.795938
H	-1.351120	1.797455	4.601282
H	-7.985659	-0.107774	1.265884
H	-5.506665	0.210068	1.329498
H	3.270344	0.649929	2.077992
H	5.555958	1.404092	2.752448
C	3.311868	-3.510670	0.099986
C	4.107351	-2.268901	0.206885
C	4.862927	-2.002402	1.290249
H	4.951411	-2.713034	2.105817
H	5.415437	-1.073722	1.384569
C	2.726041	-4.155448	1.119327
H	2.762642	-3.712534	2.114897
C	4.079491	-1.347888	-0.962171
C	2.898405	-1.174876	-1.703803
C	5.225973	-0.644125	-1.366912
C	2.861724	-0.315893	-2.803792
H	2.000073	-1.702887	-1.401174
C	5.190972	0.211795	-2.466375
H	6.156621	-0.789479	-0.827130
C	4.008771	0.379261	-3.191760
H	1.934737	-0.192902	-3.355908
H	6.092349	0.740082	-2.764258
H	3.985500	1.036860	-4.056648
H	3.223287	-3.929614	-0.903229
C	2.045864	-5.487566	1.004013
H	1.032589	-5.469912	1.427136
H	1.979339	-5.819550	-0.037714
H	2.596972	-6.255164	1.564298

^{4}TS1D$_{exo\text{-}trans\text{-}p}$

Fe	-0.635473	0.367962	0.265746
N	-0.525368	1.836996	-1.105088
N	-1.310574	1.683393	1.620234
O	-2.841236	0.167118	-0.403631
C	-1.092945	3.756657	0.317096
C	-0.704584	3.182592	-0.883818
C	-0.081851	3.016034	-3.039915
H	0.198745	3.193449	-4.069836
C	-4.756369	-1.078639	-1.070025
C	0.180504	0.530620	-3.063667
C	-1.384715	3.051336	1.474059
C	-0.140640	1.716720	-2.421459
C	-0.427185	3.924089	-2.086032
H	-0.487898	5.001023	-2.170322
C	-3.357147	-0.913550	-0.691257
H	-2.742008	-1.829543	-0.666499
C	-1.750066	1.413647	2.895847
C	-1.857667	3.653448	2.693388
H	-2.002225	4.716365	2.837287
C	-2.092072	2.638680	3.571365
H	-2.465613	2.697126	4.585548
C	-5.620546	0.030007	-1.128697
C	-6.949323	-0.152622	-1.489793
C	-5.234500	-2.363670	-1.375416
H	-4.558986	-3.214815	-1.327476
C	-6.567184	-2.542004	-1.736722
H	-6.941419	-3.533255	-1.973505
C	-7.421375	-1.436850	-1.793182
H	-8.461453	-1.574797	-2.075052
N	-0.985291	-1.136415	1.556877
N	-0.154252	-0.985136	-1.154819
C	-0.265236	-3.044884	0.181327
C	-0.735311	-2.478258	1.357151
C	-1.561661	-2.330238	3.447093
H	-1.928232	-2.518128	4.448082
C	-1.857475	0.151613	3.461730
C	-0.001008	-2.345142	-0.988306
C	-1.494246	-1.030921	2.833295
C	-1.082183	-3.225430	2.536893
H	-0.983439	-4.298899	2.636083
C	0.177981	-0.723339	-2.468414
C	0.441296	-2.945915	-2.217477
H	0.641494	-4.001069	-2.348638
C	0.549024	-1.942431	-3.134764
H	0.852413	-2.007917	-4.171619
O	1.310349	0.657312	0.788306

C	2.667214	2.178255	-0.483995
C	2.260783	0.789829	-0.091654
H	2.249338	0.084932	-0.934319
C	2.385869	3.260144	0.358532
C	2.739474	4.552506	-0.023756
C	3.307473	2.401995	-1.709555
H	3.515192	1.560270	-2.367179
C	3.658637	3.695260	-2.092637
H	4.147458	3.864511	-3.047909
C	3.377192	4.772856	-1.248219
H	3.652355	5.781063	-1.544759
H	0.469407	0.588593	-4.107937
H	-0.121573	-4.120660	0.165167
H	-2.241581	0.085294	4.474450
H	-1.192410	4.836510	0.347849
H	-7.623048	0.697607	-1.537482
H	-5.228334	1.013139	-0.888241
H	1.864786	3.075041	1.292503
H	2.514818	5.390609	0.630284
C	2.956989	-1.451910	2.262981
C	3.678772	-1.147106	1.059735
C	3.933023	0.188556	0.688566
H	4.025430	0.934117	1.471313
H	4.597734	0.365053	-0.150324
C	2.412957	-0.520912	3.089481
H	2.542956	0.534516	2.876478
C	4.020113	-2.238542	0.133310
C	3.223121	-3.399250	0.055909
C	5.147970	-2.148624	-0.709890
C	3.531748	-4.418563	-0.839109
H	2.331926	-3.479764	0.666801
C	5.461962	-3.175098	-1.594685
H	5.807701	-1.289950	-0.639540
C	4.652315	-4.312088	-1.666284
H	2.897722	-5.299173	-0.892182
H	6.344933	-3.094448	-2.221569
H	4.898485	-5.112955	-2.357475
H	2.818190	-2.499560	2.515286
C	1.647547	-0.838931	4.325820
H	0.692339	-0.302016	4.324886
H	1.450310	-1.908753	4.435605
H	2.202228	-0.487401	5.207905

^{6}**TS1D**$_{\mathbf{exo\text{-}trans\text{-}p}}$

Fe	-0.587171	0.323105	0.302557
N	-0.611386	1.879419	-1.065013
N	-1.411435	1.557772	1.725978
O	-2.766002	0.095949	-0.373924
C	-1.328057	3.669517	0.461321
C	-0.880025	3.192538	-0.773277
C	-0.195635	3.159669	-2.926618
H	0.093949	3.408507	-3.939333
C	-4.673012	-1.106133	-1.126277
C	0.191466	0.664888	-3.045445
C	-1.588019	2.916536	1.608102
C	-0.193221	1.825708	-2.370677
C	-0.619925	4.002575	-1.940969
H	-0.743184	5.076698	-1.988514
C	-3.274508	-0.965687	-0.738760
H	-2.653609	-1.875758	-0.780918
C	-1.845424	1.194312	2.976009
C	-2.124938	3.433427	2.845218
H	-2.359174	4.473842	3.030932
C	-2.288467	2.371042	3.687209
H	-2.680057	2.373562	4.696445
C	-5.540745	0.001154	-1.097554
C	-6.869382	-0.157248	-1.469870
C	-5.147401	-2.365278	-1.530017
H	-4.469185	-3.215315	-1.549329
C	-6.479948	-2.519002	-1.902763
H	-6.851419	-3.489972	-2.215623
C	-7.337664	-1.415553	-1.871955
H	-8.377690	-1.534510	-2.162527
N	-0.950103	-1.310541	1.522887
N	-0.110430	-0.990408	-1.243798
C	-0.199026	-3.104211	0.011685
C	-0.671740	-2.627661	1.236802
C	-1.483792	-2.603588	3.346427
H	-1.836949	-2.860019	4.337170
C	-1.869554	-0.112376	3.470256
C	0.061699	-2.350150	-1.136983
C	-1.457605	-1.267795	2.800706
C	-0.993537	-3.441520	2.384647
H	-0.874689	-4.516534	2.434828
C	0.226778	-0.634507	-2.530655
C	0.529424	-2.871226	-2.398157
H	0.751184	-3.912160	-2.594346
C	0.632772	-1.813223	-3.257095

H	0.950234	-1.823668	-4.291979
O	1.299678	0.675370	0.768076
C	2.619653	2.263265	-0.452351
C	2.251127	0.864733	-0.100193
H	2.302726	0.154013	-0.933568
C	2.245907	3.324849	0.381912
C	2.565318	4.633395	0.027498
C	3.315375	2.523872	-1.641550
H	3.593628	1.697896	-2.292823
C	3.632710	3.833267	-1.994358
H	4.165246	4.032106	-2.919895
C	3.260561	4.890126	-1.157891
H	3.509007	5.911459	-1.431919
H	0.497171	0.784180	-4.080525
H	-0.036224	-4.175616	-0.062980
H	-2.253093	-0.245422	4.477477
H	-1.509165	4.737807	0.531196
H	-7.545926	0.691846	-1.450243
H	-5.151053	0.963926	-0.782297
H	1.682669	3.111330	1.284754
H	2.268370	5.455633	0.672401
C	2.993164	-1.394936	2.267041
C	3.728812	-1.043840	1.079652
C	3.984826	0.295851	0.750035
H	4.014417	1.040859	1.537374
H	4.646606	0.508628	-0.082132
C	2.419739	-0.503991	3.114447
H	2.518661	0.560771	2.928078
C	4.092407	-2.108259	0.129187
C	3.289201	-3.258714	-0.006344
C	5.243349	-1.997901	-0.679368
C	3.615066	-4.250188	-0.926455
H	2.380934	-3.351779	0.577260
C	5.574880	-2.998097	-1.587490
H	5.905505	-1.145978	-0.562524
C	4.759166	-4.125806	-1.717847
H	2.975667	-5.122641	-1.026768
H	6.475390	-2.904707	-2.187034
H	5.018896	-4.905764	-2.427738
H	2.877168	-2.452811	2.486102
C	1.651942	-0.876741	4.334223
H	0.677533	-0.375130	4.333404
H	1.491683	-1.955354	4.415288
H	2.182987	-0.527473	5.231483

^{4}IIID$_{exo\text{-}trans\text{-}p}$

Fe	-0.710416	0.354570	0.317711
N	-0.561974	1.972810	-0.825654
N	-1.351577	1.466847	1.826148
O	-2.819089	0.127377	-0.337217
C	-0.979471	3.700372	0.869423
C	-0.654246	3.285197	-0.412184
C	-0.248664	3.385346	-2.623961
H	-0.055696	3.691958	-3.643350
C	-4.663355	-1.147823	-1.125575
C	-0.073142	0.919505	-2.993476
C	-1.348954	2.844945	1.896445
C	-0.313643	2.018160	-2.182655
C	-0.435757	4.168420	-1.524849
H	-0.432643	5.248073	-1.459307
C	-3.282217	-0.953979	-0.717727
H	-2.620983	-1.835132	-0.755110
C	-1.899068	1.024283	3.013229
C	-1.874712	3.271761	3.164081
H	-1.970934	4.304621	3.472526
C	-2.240191	2.146440	3.843584
H	-2.685473	2.069286	4.826953
C	-5.577997	-0.077149	-1.107106
C	-6.892221	-0.290940	-1.500341
C	-5.077627	-2.425880	-1.540394
H	-4.364122	-3.246565	-1.551714
C	-6.396163	-2.634108	-1.933649
H	-6.721787	-3.618557	-2.254833
C	-7.299827	-1.567285	-1.912757
H	-8.329261	-1.729189	-2.219576
N	-0.896179	-1.281603	1.438715
N	-0.134083	-0.787599	-1.221733
C	0.093344	-2.949757	-0.076183
C	-0.466269	-2.559545	1.131322
C	-1.443684	-2.724571	3.154798
H	-1.847179	-3.060082	4.101316
C	-2.018695	-0.302566	3.396949
C	0.218637	-2.123282	-1.181563
C	-1.504161	-1.372272	2.677606
C	-0.779639	-3.453561	2.209687
H	-0.541649	-4.509416	2.220162
C	0.054046	-0.383923	-2.531846
C	0.652067	-2.556280	-2.479516

H	0.999423	-3.555702	-2.706358
C	0.525139	-1.487866	-3.320507
H	0.750289	-1.434604	-4.377775
O	1.555592	0.819497	0.898502
C	2.735561	2.347145	-0.604982
C	2.396938	0.907564	-0.277002
H	1.793743	0.500552	-1.082392
C	2.592650	3.378596	0.326950
C	2.925956	4.691270	-0.015466
C	3.206908	2.651414	-1.889079
H	3.302881	1.857571	-2.627074
C	3.539464	3.960745	-2.232446
H	3.902269	4.182100	-3.232426
C	3.401377	4.986408	-1.293464
H	3.660560	6.007469	-1.558596
H	0.110835	1.101122	-4.046934
H	0.408446	-3.981273	-0.181290
H	-2.470343	-0.512092	4.360672
H	-1.015690	4.767316	1.061031
H	-7.604681	0.528217	-1.489287
H	-5.235952	0.900841	-0.783764
H	2.190431	3.158193	1.309694
H	2.810755	5.484025	0.718983
C	2.796365	-1.004449	1.831497
C	3.416663	-1.236639	0.659682
C	3.664340	0.007501	-0.172340
H	4.479630	0.590665	0.274803
H	3.973215	-0.226237	-1.193239
C	2.310979	0.391690	2.088645
H	3.158235	1.089697	2.156796
C	3.834839	-2.572777	0.185685
C	3.236242	-3.740874	0.697617
C	4.837273	-2.723770	-0.788016
C	3.622337	-5.002194	0.253818
H	2.443986	-3.655196	1.434247
C	5.225209	-3.987680	-1.233792
H	5.342166	-1.850164	-1.188670
C	4.618172	-5.132908	-0.718731
H	3.144096	-5.887423	0.665040
H	6.008598	-4.075217	-1.981359
H	4.918052	-6.116865	-1.067499
H	2.587155	-1.779434	2.562651
C	1.455477	0.559179	3.326597
H	1.033907	1.566576	3.373959
H	0.641612	-0.164344	3.348639
H	2.079894	0.399053	4.211926

$^{4}VD_{exo\text{-}trans\text{-}p}$

Fe	-0.911554	0.899532	0.917363
N	-0.249307	-0.389094	2.299729
N	0.667684	2.064675	1.280516
O	-0.264989	-0.209693	-0.678819
C	1.899503	0.657223	2.880248
C	0.968163	-0.362010	2.959948
C	0.019503	-2.309080	3.565911
H	-0.220361	-3.269808	4.002541
C	-0.555433	-2.531518	-1.076096
C	-2.097908	-2.016094	2.289681
C	1.751280	1.787243	2.097879
C	-0.842562	-1.584366	2.678519
C	1.137231	-1.548763	3.746130
H	2.004959	-1.759199	4.357465
C	0.189660	-1.371817	-0.627764
H	1.164524	-1.539414	-0.150768
C	0.974744	3.266134	0.663379
C	2.734859	2.826272	1.992138
H	3.666909	2.845945	2.541607
C	2.259896	3.735728	1.094649
H	2.720621	4.655197	0.758192
C	-1.857492	-2.386195	-1.590908
C	-2.582773	-3.514319	-1.950732
C	0.008954	-3.811445	-0.922932
H	1.014688	-3.909362	-0.521047
C	-0.721007	-4.936698	-1.288415
H	-0.291369	-5.927297	-1.176422
C	-2.014637	-4.786546	-1.802498
H	-2.584260	-5.666492	-2.087669
N	-1.733291	2.372292	-0.142596
N	-2.678341	-0.058860	0.917761
C	-3.953750	1.406260	-0.589744
C	-2.986294	2.384575	-0.733285
C	-1.998051	4.284714	-1.419700
H	-1.750190	5.236772	-1.870562
C	0.152285	3.951396	-0.211449
C	-3.803985	0.268182	0.182954
C	-1.120193	3.539761	-0.563892
C	-3.153924	3.569347	-1.524084
H	-4.050682	3.814105	-2.078084

C	-2.956788	-1.290677	1.483240
C	-4.799787	-0.757971	0.309490
H	-5.774118	-0.727024	-0.160545
C	-4.272311	-1.726332	1.110680
H	-4.723305	-2.655457	1.433480
H	-2.440358	-2.976282	2.659873
H	-4.892814	1.542548	-1.115214
H	0.515330	4.886496	-0.623659
H	2.810182	0.556894	3.459839
H	-3.589637	-3.411623	-2.343627
H	-2.277256	-1.390210	-1.684736
C	2.460554	-0.596288	-2.497584
C	0.919549	1.222495	-3.247368
H	0.087510	0.519272	-3.243949
C	1.854564	-1.645117	-3.097830
H	1.165848	-1.503719	-3.923999
H	2.047821	-2.669370	-2.797320
C	2.131979	0.794070	-2.862589
H	2.951276	1.510687	-2.803778
C	3.471477	-0.804737	-1.427077
C	3.588167	0.120122	-0.374920
C	4.315926	-1.929825	-1.423118
C	4.513292	-0.079989	0.649688
H	2.943458	0.993980	-0.356629
C	5.234869	-2.130983	-0.395592
H	4.274699	-2.631747	-2.250815
C	5.338006	-1.205479	0.646404
H	4.592762	0.651123	1.448466
H	5.886011	-3.000350	-0.418189
H	6.065060	-1.354912	1.439664
C	0.609400	2.614639	-3.705695
H	-0.240807	3.033946	-3.155488
H	1.467819	3.284132	-3.585700
H	0.326979	2.616307	-4.767656

^{4}TS2D$_{exo\text{-}trans\text{-}p}$

Fe	-0.805150	0.812193	0.868877
N	-0.681350	-0.845096	2.002967
N	0.982313	1.394958	1.589100
O	-0.228085	-0.025415	-0.813499
C	1.649900	-0.659445	2.766050
C	0.449390	-1.335378	2.632326
C	-1.099139	-2.950392	2.868222
H	-1.654536	-3.846136	3.113327
C	-0.877050	-2.296799	-1.216293
C	-2.933606	-1.774509	1.662282
C	1.877916	0.628143	2.309717
C	-1.639193	-1.833131	2.146818
C	0.195793	-2.644972	3.164407
H	0.921253	-3.236104	3.708071
C	0.150747	-1.269471	-0.892898
H	0.914934	-1.600611	-0.178510
C	1.624806	2.598013	1.363609
C	3.088660	1.363790	2.545183
H	3.935251	0.984584	3.102170
C	2.932327	2.585232	1.959703
H	3.624310	3.417195	1.937584
C	-2.086298	-1.917928	-1.813791
C	-3.071716	-2.871594	-2.055074
C	-0.660355	-3.637384	-0.869144
H	0.273917	-3.925753	-0.392178
C	-1.649163	-4.589523	-1.107859
H	-1.483094	-5.626029	-0.828889
C	-2.855410	-4.207852	-1.703523
H	-3.625587	-4.950536	-1.891309
N	-1.092892	2.667342	0.166990
N	-2.765236	0.439131	0.606887
C	-3.391683	2.437109	-0.680476
C	-2.209201	3.135921	-0.500672
C	-0.736414	4.836379	-0.554823
H	-0.222768	5.777362	-0.703194
C	1.092280	3.676331	0.677793
C	-3.649624	1.186720	-0.146240
C	-0.180281	3.705920	0.133989
C	-1.990162	4.480204	-0.956582
H	-2.718746	5.070793	-1.496650
C	-3.450793	-0.711086	0.944224
C	-4.904699	0.496946	-0.274924
H	-5.763083	0.881592	-0.810136
C	-4.783373	-0.675280	0.407504
H	-5.518449	-1.457645	0.543264
H	-3.579430	-2.627083	1.840333
H	-4.184404	2.921207	-1.240945
H	1.704949	4.566137	0.578707
H	2.453498	-1.156246	3.299012
H	-4.013350	-2.572749	-2.506403
H	-2.250961	-0.871470	-2.049514
C	2.306017	-0.494067	-2.225376

C	0.940309	1.437354	-2.964708
H	0.090225	0.792522	-3.165090
C	1.324823	-1.455812	-2.507754
H	0.588588	-1.258336	-3.279400
H	1.579887	-2.504578	-2.396892
C	2.099982	0.902674	-2.507459
H	2.932550	1.573535	-2.312294
C	3.503086	-0.888113	-1.466455
C	4.025624	-0.037134	-0.472203
C	4.130063	-2.131342	-1.688617
C	5.132155	-0.422055	0.278649
H	3.537439	0.906684	-0.255473
C	5.248261	-2.503612	-0.949107
H	3.765509	-2.786037	-2.474242
C	5.750692	-1.651516	0.039044
H	5.510141	0.240070	1.051671
H	5.734557	-3.453921	-1.147838
H	6.623416	-1.944055	0.615747
C	0.730570	2.885950	-3.236460
H	-0.161294	3.249171	-2.713332
H	1.587592	3.496283	-2.937655
H	0.544964	3.042391	-4.308860

^{6}TS2D$_{exo\text{-}trans\text{-}p}$

Fe	-0.800406	0.792503	0.712438
N	-0.753020	-0.808263	2.043524
N	0.959058	1.460385	1.600706
O	-0.230124	-0.166444	-0.852468
C	1.542030	-0.552420	2.891823
C	0.354667	-1.261516	2.731761
C	-1.190850	-2.893983	2.926730
H	-1.742496	-3.793288	3.168001
C	-0.733908	-2.484099	-1.155037
C	-2.995782	-1.730548	1.622990
C	1.807805	0.723169	2.399929
C	-1.713529	-1.793559	2.159331
C	0.086189	-2.566167	3.280203
H	0.786536	-3.142717	3.870726
C	0.207143	-1.389377	-0.853380
H	1.020864	-1.630227	-0.161200
C	1.596738	2.660024	1.355706
C	3.005989	1.477545	2.662637
H	3.826085	1.134105	3.279682
C	2.877700	2.673219	2.015658
H	3.572726	3.502865	1.997306
C	-1.984523	-2.204138	-1.726949
C	-2.895694	-3.234193	-1.939047
C	-0.404293	-3.801908	-0.801207
H	0.560030	-4.011944	-0.344241
C	-1.319746	-4.828997	-1.012912
H	-1.068026	-5.847072	-0.731190
C	-2.564972	-4.546428	-1.584557
H	-3.278051	-5.348866	-1.750212
N	-1.125234	2.733583	0.075344
N	-2.850623	0.479057	0.552711
C	-3.444661	2.499580	-0.718126
C	-2.251493	3.203336	-0.571105
C	-0.751026	4.886872	-0.661066
H	-0.233908	5.823327	-0.825883
C	1.069630	3.711375	0.610629
C	-3.725802	1.241838	-0.191396
C	-0.198421	3.756066	0.037363
C	-2.017887	4.544574	-1.040306
H	-2.742467	5.146818	-1.573060
C	-3.525716	-0.675216	0.885912
C	-4.982648	0.548575	-0.326545
H	-5.845102	0.932724	-0.855825
C	-4.858892	-0.635154	0.340408
H	-5.599245	-1.414768	0.464816
H	-3.642670	-2.584865	1.794008
H	-4.241045	2.992258	-1.267241
H	1.688372	4.596918	0.503380
H	2.321088	-1.023478	3.482922
H	-3.867419	-3.015127	-2.371652
H	-2.236818	-1.176675	-1.967828
C	2.341460	-0.519125	-2.240242
C	0.866122	1.318353	-3.031432
H	0.071236	0.615163	-3.264507
C	1.466277	-1.553497	-2.559461
H	0.697334	-1.417759	-3.311729
H	1.772638	-2.581034	-2.397157
C	2.040366	0.864462	-2.532480
H	2.815840	1.590952	-2.302950
C	3.541432	-0.816803	-1.437120
C	3.938211	0.051378	-0.401335
C	4.292363	-1.987539	-1.663796
C	5.046912	-0.248655	0.385394
H	3.356023	0.940985	-0.185219

C	5.410233	-2.274238	-0.886216
H	4.022790	-2.650053	-2.480854
C	5.788987	-1.406857	0.142697
H	5.328981	0.425179	1.188247
H	5.992579	-3.168429	-1.087373
H	6.662021	-1.631457	0.748642
C	0.557522	2.750917	-3.298296
H	-0.359436	3.047131	-2.775280
H	1.367211	3.416867	-2.987106
H	0.366993	2.904403	-4.369821

^{4}VID$_{exo-trans-p}$

Fe	-0.780537	1.002297	0.740305
N	0.473246	0.080121	1.990498
N	0.526173	2.488592	0.584849
O	-0.319151	-0.201901	-1.019239
C	2.508966	1.379196	1.529483
C	1.835677	0.311736	2.094823
C	1.446732	-1.492147	3.381741
H	1.518811	-2.342922	4.046647
C	-1.123850	-2.514189	-0.973458
C	-0.983179	-1.702824	2.860147
C	1.881427	2.422145	0.870194
C	0.229538	-1.038381	2.773718
C	2.444677	-0.673331	2.939106
H	3.500997	-0.711405	3.170333
C	0.034455	-1.581243	-0.696767
H	0.239726	-1.574694	0.369766
C	0.318456	3.762171	0.076875
C	2.527144	3.645039	0.494577
H	3.584718	3.841298	0.614000
C	1.555436	4.484403	0.030382
H	1.657299	5.502773	-0.321138
C	-2.178342	-2.168828	-1.823149
C	-3.213073	-3.074917	-2.066805
C	-1.125235	-3.774886	-0.361891
H	-0.317633	-4.044655	0.315574
C	-2.156862	-4.679998	-0.605403
H	-2.145609	-5.654310	-0.125032
C	-3.204734	-4.332001	-1.461948
H	-4.009317	-5.036078	-1.653306
N	-2.162789	2.164962	-0.075952
N	-2.200891	-0.284709	1.263168
C	-4.016125	0.549963	-0.167379
C	-3.429508	1.767085	-0.471842
C	-3.240770	3.903529	-1.153772
H	-3.398237	4.892415	-1.564091
C	-0.891973	4.246545	-0.391323
C	-3.454809	-0.384372	0.684788
C	-2.038735	3.479340	-0.496962
C	-4.089866	2.835346	-1.164288
H	-5.089414	2.774553	-1.574427
C	-2.101800	-1.370428	2.116969
C	-4.131693	-1.554830	1.164283
H	-5.120602	-1.861302	0.851145
C	-3.304778	-2.151323	2.068138
H	-3.473526	-3.051498	2.643855
H	-1.038639	-2.578491	3.497648
H	-5.011797	0.354784	-0.550446
H	-0.924517	5.265726	-0.760747
H	3.581006	1.437327	1.676216
H	-4.027306	-2.794879	-2.729619
H	-2.206593	-1.180958	-2.270231
C	2.324464	-0.878229	-1.574349
C	0.298212	0.250554	-2.296727
H	-0.006789	-0.514891	-3.022947
C	1.348138	-2.026595	-1.402009
H	1.099173	-2.451265	-2.382908
H	1.762385	-2.842417	-0.806067
C	1.780695	0.239970	-2.091499
H	2.351054	1.136382	-2.314065
C	3.743257	-1.011777	-1.186780
C	4.513818	0.124708	-0.872741
C	4.367965	-2.269371	-1.124954
C	5.853789	0.007293	-0.515047
H	4.049283	1.105386	-0.889140
C	5.711187	-2.387467	-0.767897
H	3.813546	-3.166216	-1.384633
C	6.459409	-1.251343	-0.458583
H	6.428181	0.898430	-0.276333
H	6.173921	-3.369712	-0.737515
H	7.504375	-1.343687	-0.177467
C	-0.295397	1.576415	-2.718920
H	-1.387528	1.534757	-2.718142
H	0.029986	2.386579	-2.067459
H	0.043769	1.799907	-3.735801

^{4}TS2D$_{cc-endo-cis-p}$

Fe	1.964347	0.001410	0.172136
N	1.771769	0.595099	2.085458
N	2.144027	1.933951	-0.374487
C	1.635236	3.032443	1.766972
C	1.573439	1.886795	2.542462
C	1.324845	0.577006	4.355764
H	1.174194	0.176512	5.349876
C	1.759472	-1.594654	3.208547
C	1.921221	3.048996	0.411729
C	1.626688	-0.215142	3.196892
C	1.293225	1.878781	3.950870
H	1.110366	2.764988	4.544651
C	2.380537	2.419357	-1.645825
C	2.022367	4.244551	-0.378324
H	1.902049	5.247924	0.009335
C	2.306606	3.854137	-1.654143
H	2.469271	4.471555	-2.528149
N	2.637611	-0.539448	-1.640671
N	2.273277	-1.876166	0.820882
C	2.802503	-2.971231	-1.315958
C	2.848010	-1.826302	-2.093373
C	3.096054	-0.522345	-3.909058
H	3.267550	-0.119107	-4.898614
C	2.660896	1.647833	-2.761749
C	2.545380	-2.985971	0.044141
C	2.782960	0.269258	-2.750177
C	3.135922	-1.821022	-3.502035
H	3.346173	-2.706394	-4.087718
C	2.077616	-2.361526	2.099920
C	2.527706	-4.176140	0.849195
H	2.720065	-5.172639	0.473609
C	2.237862	-3.789731	2.122756
H	2.143410	-4.403133	3.009353
O	0.083346	-0.220813	-0.248072
C	-1.841845	-1.638506	-0.303151
C	-0.818638	-0.862909	0.470260
H	-0.424437	-1.424975	1.329764
C	-1.899865	-1.518832	-1.696821
C	-2.859398	-2.226683	-2.420256
C	-2.752317	-2.473671	0.356402
H	-2.707041	-2.576464	1.438503
C	-3.717628	-3.172395	-0.366297
H	-4.421803	-3.816184	0.152803
C	-3.774508	-3.047944	-1.757172
H	-4.523517	-3.596450	-2.321212
H	1.624374	-2.106658	4.155420
H	2.993045	-3.921009	-1.804056
H	2.815290	2.161092	-3.704982
H	1.468588	3.984504	2.259680
H	-1.177956	-0.880243	-2.195215
H	-2.892629	-2.139498	-3.502889
C	-1.759969	2.143447	-0.150262
C	-2.455652	1.151949	0.626398
C	-1.689435	0.370221	1.532172
H	-0.816205	0.846529	1.969617
H	-2.245280	-0.253216	2.228349
C	-2.361081	3.164412	-0.808969
H	-3.443274	3.269084	-0.743422
C	-3.850895	0.802655	0.376288
C	-4.696622	0.430817	1.445187
C	-4.362137	0.755253	-0.939641
C	-6.010989	0.050304	1.207741
H	-4.328292	0.487404	2.464772
C	-5.668626	0.340196	-1.173846
H	-3.705761	0.975580	-1.772788
C	-6.496856	-0.005207	-0.103423
H	-6.658619	-0.207148	2.040236
H	-6.038665	0.275686	-2.192367
H	-7.519133	-0.321799	-0.288899
H	-0.673994	2.075188	-0.143612
C	-1.638034	4.204801	-1.592634
H	-0.557525	4.040304	-1.606227
H	-2.010026	4.222878	-2.626568
H	-1.841121	5.203503	-1.181960

^{6}TS2D$_{cc\text{-}endo\text{-}cis\text{-}p}$

Fe	1.845488	-0.013788	0.159328
N	1.676683	0.565010	2.153420
N	2.155252	1.993515	-0.301572
C	1.538886	3.006308	1.855118
C	1.448687	1.845985	2.619443
C	1.129645	0.486334	4.392909
H	0.938174	0.067741	5.372609
C	1.658468	-1.664022	3.200738
C	1.888007	3.078157	0.508584
C	1.495811	-0.279891	3.230240
C	1.103441	1.799044	4.016609
H	0.883748	2.665316	4.627352

C	2.476410	2.502005	-1.542975
C	2.031757	4.295984	-0.246845
H	1.889269	5.291431	0.153916
C	2.393642	3.939712	-1.515305
H	2.607802	4.586462	-2.356522
N	2.814214	-0.484745	-1.609618
N	2.325312	-1.912830	0.847068
C	3.061319	-2.910492	-1.278597
C	3.122413	-1.753315	-2.051406
C	3.473815	-0.395021	-3.818205
H	3.718585	0.030105	-4.783003
C	2.856215	1.743577	-2.648417
C	2.701834	-2.984568	0.064317
C	3.025850	0.362341	-2.676550
C	3.533971	-1.701739	-3.431825
H	3.837676	-2.559954	-4.017204
C	2.063007	-2.419532	2.102654
C	2.674020	-4.192889	0.849031
H	2.934022	-5.175509	0.477178
C	2.277322	-3.844775	2.107088
H	2.149289	-4.485356	2.970115
O	0.016306	-0.303275	-0.299322
C	-1.929049	-1.686511	-0.391545
C	-0.891155	-0.962530	0.387402
H	-0.525880	-1.505040	1.269127
C	-1.989893	-1.539532	-1.784208
C	-2.963943	-2.217509	-2.514900
C	-2.855210	-2.513822	0.259158
H	-2.808440	-2.634469	1.339035
C	-3.836140	-3.179148	-0.471850
H	-4.552688	-3.816089	0.038186
C	-3.892784	-3.030929	-1.860829
H	-4.654164	-3.555029	-2.431273
H	1.484622	-2.198970	4.129259
H	3.336140	-3.840966	-1.765362
H	3.073361	2.284541	-3.564212
H	1.342687	3.945383	2.363150
H	-1.259032	-0.906868	-2.276897
H	-2.999251	-2.112298	-3.595593
C	-1.774895	2.135520	-0.115755
C	-2.505212	1.129246	0.615386
C	-1.791344	0.336093	1.535579
H	-0.891339	0.753357	1.977004
H	-2.359007	-0.320024	2.189183
C	-2.341999	3.161790	-0.793705
H	-3.426549	3.260954	-0.790309
C	-3.896784	0.795693	0.308198
C	-4.784616	0.421661	1.340590
C	-4.358906	0.765699	-1.025166
C	-6.091861	0.051677	1.050458
H	-4.454319	0.466502	2.373830
C	-5.659153	0.363263	-1.312597
H	-3.672709	0.995281	-1.831359
C	-6.529244	0.012288	-0.278023
H	-6.771694	-0.208707	1.855982
H	-5.991682	0.314661	-2.344891
H	-7.546439	-0.293743	-0.504582
H	-0.690501	2.078409	-0.053097
C	-1.579747	4.214545	-1.522977
H	-0.499395	4.053577	-1.479635
H	-1.893370	4.246198	-2.575671
H	-1.807780	5.206984	-1.110307

[4]TS2D$_{co\text{-}endo\text{-}cis\text{-}p}$

Fe	-1.822692	0.331550	0.234125
N	-1.983268	-0.417125	2.090081
N	-2.982765	-1.162440	-0.432565
C	-2.865308	-2.641974	1.529786
C	-2.331647	-1.714880	2.411507
C	-1.679760	-0.780732	4.352995
H	-1.432451	-0.564592	5.384224
C	-1.055160	1.443927	3.400600
C	-3.220554	-2.358561	0.219424
C	-1.571313	0.162625	3.274105
C	-2.131749	-1.949255	3.815415
H	-2.340535	-2.885130	4.317361
C	-3.597599	-1.280476	-1.664827
C	-3.972806	-3.242885	-0.626787
H	-4.290553	-4.238769	-0.346324
C	-4.228701	-2.564908	-1.783227
H	-4.787721	-2.896679	-2.648595
N	-2.116187	1.284080	-1.502749
N	-1.132460	2.042727	1.016859
C	-0.803010	3.314329	-1.059075
C	-1.521047	2.469602	-1.889548
C	-2.641992	1.754968	-3.705428
H	-3.094929	1.635231	-4.681042
C	-3.535378	-0.341060	-2.681879

C	-0.661799	3.129506	0.305708
C	-2.810691	0.838239	-2.611108
C	-1.826867	2.754176	-3.264474
H	-1.479454	3.627103	-3.801607
C	-0.829415	2.305847	2.339343
C	-0.052752	4.080134	1.193412
H	0.394198	5.013501	0.877869
C	-0.173176	3.579346	2.454849
H	0.157176	4.016244	3.388189
O	-0.077115	-0.469797	-0.124771
C	1.929598	0.822356	0.247303
C	0.975209	-0.249851	0.741903
H	0.607355	0.022624	1.739020
C	1.941087	1.196816	-1.099248
C	2.843346	2.159652	-1.554746
C	2.821119	1.432393	1.135301
H	2.805976	1.158112	2.188430
C	3.724882	2.393385	0.682042
H	4.413339	2.861021	1.380404
C	3.740313	2.757017	-0.666983
H	4.442881	3.506235	-1.020897
H	-0.766890	1.776903	4.392043
H	-0.378196	4.212346	-1.493991
H	-4.051346	-0.563638	-3.609849
H	-3.104155	-3.627390	1.915913
H	1.224379	0.742524	-1.775018
H	2.842164	2.447409	-2.602937
C	1.665873	-2.385670	-1.297537
C	2.442170	-2.051711	-0.191923
C	1.750117	-1.631081	1.045518
H	0.977390	-2.329467	1.380028
H	2.439230	-1.441932	1.868264
C	0.276828	-2.437826	-1.245275
H	-0.231431	-2.665582	-0.317923
C	3.887280	-1.860902	-0.312321
C	4.732572	-2.118751	0.787994
C	4.469279	-1.410391	-1.517027
C	6.109692	-1.961920	0.677085
H	4.312399	-2.486431	1.718931
C	5.843374	-1.227568	-1.614460
H	3.834752	-1.139597	-2.353728
C	6.668435	-1.510644	-0.521875
H	6.748554	-2.189047	1.525062
H	6.272947	-0.855125	-2.539399
H	7.742705	-1.373019	-0.602354
H	2.143674	-2.491932	-2.268080
C	-0.561872	-2.412435	-2.469494
H	0.023599	-2.577630	-3.378639
H	-1.384670	-3.131923	-2.409446
H	-1.022164	-1.419022	-2.538644

[6]TS2D$_{co\text{-}endo\text{-}cis\text{-}p}$

Fe	-1.717204	0.225817	0.220984
N	-1.982428	-0.570636	2.127984
N	-3.162854	-1.118608	-0.437933
C	-3.174961	-2.630392	1.506772
C	-2.491157	-1.821488	2.410868
C	-1.604285	-1.075500	4.346829
H	-1.274967	-0.948603	5.370107
C	-0.782864	1.126493	3.445299
C	-3.522253	-2.289720	0.201411
C	-1.434421	-0.097883	3.302230
C	-2.250701	-2.143268	3.794363
H	-2.557597	-3.062385	4.276721
C	-3.809859	-1.129341	-1.658391
C	-4.381422	-3.066850	-0.653051
H	-4.807432	-4.026783	-0.390904
C	-4.569048	-2.344397	-1.796457
H	-5.173735	-2.599433	-2.657262
N	-2.206399	1.398869	-1.422498
N	-1.024473	1.950846	1.140608
C	-0.877784	3.389286	-0.849000
C	-1.646426	2.624567	-1.722385
C	-2.863576	2.055335	-3.534732
H	-3.369295	2.022953	-4.491196
C	-3.717083	-0.127346	-2.622101
C	-0.606626	3.088657	0.482453
C	-2.967638	1.041503	-2.516568
C	-2.038615	3.027610	-3.048557
H	-1.739316	3.951708	-3.526062
C	-0.585245	2.071633	2.442913
C	0.122912	3.936543	1.389323
H	0.564338	4.887729	1.122708
C	0.130093	3.312370	2.601856
H	0.575972	3.652636	3.527397
O	-0.032688	-0.599040	-0.213764
C	1.953068	0.641365	0.438943
C	1.031501	-0.538051	0.697549

H	0.632136	-0.476054	1.716790
C	1.940338	1.309539	-0.787925
C	2.829797	2.358504	-1.027852
C	2.856559	1.044113	1.428423
H	2.861854	0.538834	2.392174
C	3.749160	2.087846	1.188528
H	4.447801	2.390487	1.963383
C	3.739828	2.746751	-0.043617
H	4.433222	3.561714	-0.230720
H	-0.387150	1.361516	4.428457
H	-0.493101	4.331111	-1.227243
H	-4.276985	-0.273580	-3.540514
H	-3.529402	-3.588202	1.875125
H	1.216664	1.017166	-1.540916
H	2.807232	2.875454	-1.983598
C	1.767821	-2.035199	-1.754570
C	2.544923	-2.013560	-0.612070
C	1.833191	-1.913080	0.691849
H	1.094651	-2.705539	0.850546
H	2.517447	-1.901372	1.540186
C	0.368124	-2.104187	-1.696640
H	-0.111562	-2.688014	-0.920461
C	3.993249	-1.812883	-0.667569
C	4.822790	-2.359163	0.333891
C	4.591581	-1.072779	-1.709185
C	6.203074	-2.198706	0.276861
H	4.388740	-2.948050	1.136121
C	5.969161	-0.891370	-1.746913
H	3.968632	-0.584007	-2.450435
C	6.779635	-1.460129	-0.760122
H	6.830118	-2.646495	1.041785
H	6.412529	-0.297903	-2.540666
H	7.856172	-1.321205	-0.794520
H	2.227382	-1.862001	-2.724436
C	-0.476310	-1.774898	-2.872340
H	-0.787433	-2.710513	-3.360011
H	-1.391321	-1.273239	-2.555311
H	0.047104	-1.151702	-3.601711

[4]TS2D$_{cc\text{-}exo\text{-}trans\text{-}p}$

Fe	-0.851398	0.910137	0.802701
N	-1.267633	-0.750165	1.852645
N	0.916499	1.031854	1.767506
O	-0.279744	0.033143	-0.835248
C	0.920881	-1.150028	2.902090
C	-0.375611	-1.516183	2.581327
C	-2.286336	-2.704029	2.558447
H	-3.067922	-3.440086	2.693145
C	-1.148659	-2.149377	-1.313200
C	-3.625810	-1.075847	1.232067
C	1.500674	0.060023	2.556926
C	-2.448248	-1.471097	1.841165
C	-1.001278	-2.735650	3.011188
H	-0.512524	-3.500185	3.601050
C	-0.021353	-1.245802	-0.946759
H	0.662392	-1.675659	-0.201537
C	1.845552	2.051792	1.687131
C	2.803861	0.484364	2.987790
H	3.454649	-0.094365	3.630346
C	3.015926	1.721324	2.453554
H	3.878246	2.365910	2.565548
C	-2.284920	-1.628737	-1.946963
C	-3.364942	-2.460521	-2.229806
C	-1.098194	-3.508460	-0.974956
H	-0.218810	-3.906601	-0.473337
C	-2.181132	-4.338665	-1.257135
H	-2.145099	-5.389417	-0.984540
C	-3.315145	-3.815431	-1.886194
H	-4.158866	-4.463038	-2.107190
N	-0.576682	2.773649	0.123545
N	-2.794450	1.037791	0.294597
C	-2.724585	3.109462	-1.026401
C	-1.430888	3.481659	-0.699582
C	0.427678	4.741823	-0.558512
H	1.181562	5.514752	-0.634432
C	1.694687	3.217961	0.955740
C	-3.361986	1.980984	-0.539026
C	0.568193	3.541183	0.218177
C	-0.806932	4.700107	-1.135373
H	-1.274609	5.431656	-1.781386
C	-3.779358	0.095993	0.511315
C	-4.726099	1.632132	-0.832046
H	-5.388459	2.218715	-1.455300
C	-4.986908	0.468461	-0.173770
H	-5.906292	-0.101718	-0.148135
H	-4.479297	-1.740925	1.304065
H	-3.288360	3.765277	-1.681324
H	2.512458	3.930872	0.966524

H	1.505760	-1.837418	3.503910
H	-4.249625	-2.052195	-2.709908
H	-2.317178	-0.569180	-2.179556
C	2.244246	-0.688242	-2.064323
C	3.278986	1.560802	-2.128557
H	4.260075	1.121983	-1.953536
C	1.130333	-1.433899	-2.494197
H	0.474063	-0.993560	-3.240420
H	1.223802	-2.514483	-2.548455
C	2.199964	0.746781	-2.201730
H	1.226627	1.171802	-2.435478
C	3.313638	-1.358915	-1.317502
C	3.810692	-0.792633	-0.125382
C	3.810845	-2.611057	-1.735350
C	4.776478	-1.460094	0.622502
H	3.401709	0.144900	0.231469
C	4.794341	-3.260430	-0.997238
H	3.453427	-3.049180	-2.662411
C	5.276512	-2.688888	0.185643
H	5.133326	-1.019606	1.548593
H	5.188952	-4.211246	-1.342520
H	6.038557	-3.203258	0.763983
C	3.237488	3.038023	-2.323644
H	3.840715	3.326187	-3.195403
H	2.218536	3.411515	-2.455599
H	3.686787	3.544618	-1.458959

^{6}TS2D$_{\text{cc-exo-trans-p}}$

Fe	-0.783691	0.889935	0.666960
N	-1.289582	-0.701932	1.907236
N	0.974036	1.067906	1.776312
O	-0.276413	-0.092232	-0.880626
C	0.882203	-1.063928	3.003321
C	-0.418734	-1.442002	2.680652
C	-2.342887	-2.619800	2.636145
H	-3.130023	-3.349505	2.774666
C	-1.154365	-2.283838	-1.280071
C	-3.635977	-0.993111	1.223744
C	1.512864	0.116877	2.617442
C	-2.477864	-1.403406	1.877239
C	-1.071321	-2.644673	3.131648
H	-0.612176	-3.397454	3.759470
C	-0.035850	-1.378381	-0.938050
H	0.676670	-1.778821	-0.206942
C	1.913346	2.072944	1.667572
C	2.821922	0.535717	3.048738
H	3.453666	-0.020942	3.728997
C	3.069552	1.744637	2.463111
H	3.945583	2.372015	2.567345
C	-2.307224	-1.777674	-1.899313
C	-3.382208	-2.622728	-2.157886
C	-1.085569	-3.640435	-0.928103
H	-0.195116	-4.025248	-0.436080
C	-2.163906	-4.482223	-1.187159
H	-2.114579	-5.529920	-0.905859
C	-3.311904	-3.974403	-1.804480
H	-4.152208	-4.632173	-2.007310
N	-0.520968	2.855723	0.081896
N	-2.812532	1.121477	0.276691
C	-2.705361	3.226838	-0.990447
C	-1.393079	3.589823	-0.695451
C	0.498512	4.815035	-0.578917
H	1.259901	5.579826	-0.663457
C	1.766150	3.230421	0.906921
C	-3.367822	2.091335	-0.530001
C	0.639636	3.595188	0.174136
C	-0.754990	4.809391	-1.120321
H	-1.221649	5.568349	-1.734932
C	-3.794282	0.178862	0.488617
C	-4.738441	1.752899	-0.823971
H	-5.405936	2.352701	-1.429142
C	-5.002111	0.572469	-0.192549
H	-5.928393	0.012757	-0.178280
H	-4.495919	-1.651790	1.289819
H	-3.273726	3.907207	-1.616958
H	2.597128	3.929261	0.910565
H	1.443972	-1.733540	3.647062
H	-4.278492	-2.228008	-2.627331
H	-2.355153	-0.721032	-2.141470
C	2.226169	-0.816718	-2.129231
C	3.162218	1.478954	-2.237276
H	4.147474	1.101329	-1.968804
C	1.163804	-1.627546	-2.550190
H	0.478522	-1.249831	-3.304173
H	1.277882	-2.706714	-2.520724
C	2.132516	0.607368	-2.347504
H	1.162878	0.976252	-2.673566
C	3.300359	-1.406213	-1.317595

C	3.716753	-0.780980	-0.124580
C	3.877773	-2.640557	-1.678808
C	4.686532	-1.374984	0.678772
H	3.247043	0.144474	0.187344
C	4.863510	-3.216293	-0.884018
H	3.578504	-3.121804	-2.605187
C	5.267327	-2.586672	0.298412
H	4.981987	-0.889312	1.603405
H	5.320443	-4.154153	-1.185092
H	6.031909	-3.042721	0.920669
C	3.062356	2.941396	-2.508839
H	3.690535	3.213525	-3.368318
H	2.034883	3.260299	-2.703670
H	3.447328	3.510775	-1.653213

^{4}VIIID$_{exo\text{-}trans\text{-}p}$

Fe	-0.866069	0.870920	0.775238
N	-1.328942	-0.808765	1.779328
N	0.903552	0.920304	1.758341
O	-0.288637	0.077254	-0.857222
C	0.851227	-1.304036	2.804105
C	-0.458354	-1.620452	2.481399
C	-2.401562	-2.753681	2.426794
H	-3.203050	-3.472071	2.537394
C	-1.158847	-2.119556	-1.339516
C	-3.690987	-1.053025	1.140215
C	1.465155	-0.097822	2.501678
C	-2.527387	-1.495597	1.744450
C	-1.118584	-2.834835	2.877285
H	-0.651339	-3.630349	3.443235
C	0.027105	-1.233408	-1.023065
H	0.591765	-1.653289	-0.175290
C	1.848217	1.926731	1.730527
C	2.776427	0.279661	2.953832
H	3.414185	-0.340401	3.570575
C	3.010657	1.539166	2.484105
H	3.880595	2.165451	2.636689
C	-2.272405	-1.582992	-1.994566
C	-3.381793	-2.384754	-2.257300
C	-1.159981	-3.466133	-0.960128
H	-0.300741	-3.879943	-0.436240
C	-2.270383	-4.268677	-1.222231
H	-2.271032	-5.310482	-0.914080
C	-3.383258	-3.728654	-1.872585
H	-4.248762	-4.352758	-2.076823
N	-0.553160	2.768941	0.188663
N	-2.807204	1.068170	0.273429
C	-2.684384	3.188045	-0.963125
C	-1.387856	3.526295	-0.607227
C	0.486701	4.751966	-0.387647
H	1.250564	5.518542	-0.415804
C	1.718403	3.131826	1.058406
C	-3.347179	2.052329	-0.528465
C	0.601457	3.511953	0.331581
C	-0.742051	4.755717	-0.978798
H	-1.192799	5.524871	-1.592460
C	-3.812436	0.142538	0.452086
C	-4.717179	1.743045	-0.844469
H	-5.362176	2.365360	-1.451118
C	-5.007754	0.563852	-0.228894
H	-5.939190	0.012951	-0.229019
H	-4.560283	-1.699652	1.187053
H	-3.230699	3.880624	-1.594730
H	2.546361	3.831457	1.112180
H	1.417702	-2.028106	3.380290
H	-4.249674	-1.958588	-2.752894
H	-2.270398	-0.529383	-2.255349
C	2.264077	-0.609063	-1.928089
C	3.333816	1.608513	-2.083099
H	4.318824	1.145487	-2.043770
C	1.064780	-1.342695	-2.312413
H	0.517865	-0.891078	-3.141354
H	1.227682	-2.407037	-2.479345
C	2.229667	0.814789	-2.044277
H	1.244278	1.255017	-2.176924
C	3.355795	-1.307015	-1.275244
C	4.015531	-0.723344	-0.166542
C	3.742295	-2.605278	-1.689380
C	5.026506	-1.411534	0.494206
H	3.677094	0.232077	0.214864
C	4.774938	-3.270830	-1.044110
H	3.266457	-3.061488	-2.551244
C	5.415893	-2.678317	0.052057
H	5.500712	-0.963873	1.362103
H	5.084919	-4.251935	-1.390103
H	6.212249	-3.210891	0.563831
C	3.298343	3.086043	-2.244608
H	3.801635	3.379394	-3.176497

H	2.280302	3.482403	-2.241035
H	3.866467	3.560538	-1.432577

^{4}TS2D$_{\text{co-exo-trans-p}}$

Fe	-0.868328	0.804050	0.762691
N	0.826848	0.486190	1.801625
N	-0.274443	2.704118	0.451202
O	-0.299995	-0.039799	-0.899927
C	2.055711	2.534643	1.222998
C	1.962962	1.271875	1.784884
C	2.530055	-0.553760	2.972955
H	3.028900	-1.305155	3.571008
C	-0.822863	-2.385219	-1.145308
C	0.314320	-1.692582	2.822123
C	0.995082	3.215072	0.646107
C	1.163434	-0.634898	2.537537
C	3.027084	0.624072	2.501418
H	4.017101	1.038705	2.636766
C	0.224421	-1.314042	-0.883382
H	0.737654	-1.525038	0.064293
C	-1.012021	3.742870	-0.085705
C	1.052875	4.585013	0.216298
H	1.934143	5.210717	0.275976
C	-0.195239	4.916302	-0.222687
H	-0.544609	5.865396	-0.608145
C	-1.950481	-2.096825	-1.919994
C	-2.898153	-3.087653	-2.176237
C	-0.653450	-3.673939	-0.630877
H	0.215085	-3.897248	-0.014507
C	-1.601762	-4.665898	-0.884610
H	-1.465801	-5.662260	-0.473343
C	-2.726346	-4.374532	-1.659561
H	-3.465397	-5.145471	-1.858492
N	-2.710783	1.322868	0.160722
N	-1.647354	-0.851398	1.602974
C	-3.888433	-0.789837	0.597217
C	-3.803767	0.484119	0.062305
C	-4.459813	2.413803	-0.889981
H	-5.002290	3.218062	-1.369789
C	-2.330128	3.657906	-0.503616
C	-2.892798	-1.390772	1.347450
C	-3.107668	2.514804	-0.411449
C	-4.886632	1.151475	-0.606997
H	-5.853261	0.706235	-0.803286
C	-0.996604	-1.785634	2.385506
C	-3.026062	-2.669562	1.987783
H	-3.909373	-3.291714	1.933522
C	-1.855643	-2.909105	2.641278
H	-1.578848	-3.769165	3.236893
H	0.705655	-2.506747	3.422959
H	-4.812736	-1.338688	0.454192
H	-2.781146	4.543338	-0.939146
H	3.002522	3.058147	1.310578
H	-3.774889	-2.854282	-2.774462
H	-2.093324	-1.085567	-2.287996
C	2.396267	-0.455161	-1.891667
C	0.759968	1.148315	-2.706866
H	0.145765	0.365211	-3.131045
C	1.372655	-1.512388	-2.011415
H	0.858605	-1.494522	-2.975085
H	1.763499	-2.516881	-1.858879
C	2.020111	0.852550	-2.221286
H	2.688987	1.680634	-2.009523
C	3.690151	-0.737371	-1.274758
C	4.415085	0.268866	-0.594408
C	4.265442	-2.026444	-1.358161
C	5.653726	-0.002682	-0.027880
H	3.979262	1.251929	-0.462543
C	5.511697	-2.290519	-0.801755
H	3.757909	-2.815241	-1.902305
C	6.209323	-1.281421	-0.132306
H	6.187638	0.780704	0.501888
H	5.944029	-3.281902	-0.895868
H	7.180845	-1.490380	0.305821
C	0.238470	2.521436	-2.888541
H	-0.797437	2.572535	-2.536995
H	0.831865	3.278911	-2.372854
H	0.210409	2.753124	-3.964350

^{6}TS2D$_{\text{co-exo-trans-p}}$

Fe	-0.866526	0.764706	0.589815
N	0.840295	0.636719	1.785903
N	-0.426058	2.787499	0.356478
O	-0.243859	-0.109799	-1.009872
C	1.899392	2.768441	1.170156
C	1.905807	1.512085	1.770417
C	2.589356	-0.253107	2.999226
H	3.137471	-0.951405	3.618372

C	-0.719789	-2.481485	-1.103784
C	0.441054	-1.552497	2.835270
C	0.808815	3.374820	0.549584
C	1.236095	-0.445544	2.544348
C	3.004322	0.954557	2.518340
H	3.959358	1.440827	2.668322
C	0.316659	-1.391989	-0.891187
H	0.777859	-1.508412	0.096837
C	-1.231210	3.752891	-0.215165
C	0.783461	4.730651	0.065512
H	1.620005	5.416608	0.104939
C	-0.480273	4.967087	-0.397091
H	-0.878213	5.881475	-0.818048
C	-1.862295	-2.243062	-1.873090
C	-2.792417	-3.261927	-2.082023
C	-0.518638	-3.746943	-0.543734
H	0.359077	-3.930270	0.072528
C	-1.447860	-4.766222	-0.752694
H	-1.286518	-5.744007	-0.307703
C	-2.587200	-4.525671	-1.523884
H	-3.312358	-5.318035	-1.685798
N	-2.835112	1.239377	0.131796
N	-1.597034	-0.878222	1.633480
C	-3.876033	-0.912955	0.705915
C	-3.889858	0.351253	0.122914
C	-4.669229	2.225913	-0.860971
H	-5.271615	2.987410	-1.339287
C	-2.555596	3.567540	-0.604433
C	-2.827019	-1.469611	1.430898
C	-3.297823	2.399319	-0.452568
C	-5.032279	0.957789	-0.512304
H	-5.992089	0.475731	-0.645780
C	-0.873647	-1.749085	2.421761
C	-2.875221	-2.740001	2.107342
H	-3.727261	-3.406827	2.104407
C	-1.671864	-2.909152	2.726259
H	-1.342478	-3.741656	3.334381
H	0.884910	-2.329182	3.450307
H	-4.783172	-1.502871	0.624054
H	-3.057264	4.414824	-1.061527
H	2.806278	3.359725	1.256245
H	-3.681117	-3.066741	-2.675918
H	-2.033563	-1.249910	-2.275980
C	2.476069	-0.493798	-1.861586
C	0.690656	0.917940	-2.676170
H	0.203783	0.103901	-3.198891
C	1.503932	-1.619187	-1.933198
H	1.049156	-1.710283	-2.924355
H	1.943981	-2.584419	-1.685828
C	2.006610	0.757689	-2.228049
H	2.596886	1.649884	-2.042681
C	3.788508	-0.677390	-1.242624
C	4.431254	0.381389	-0.562787
C	4.455423	-1.919487	-1.325377
C	5.687588	0.204013	0.003825
H	3.918821	1.327517	-0.431139
C	5.720137	-2.087436	-0.771906
H	4.003624	-2.745396	-1.864794
C	6.339361	-1.028012	-0.103106
H	6.159500	1.024623	0.536076
H	6.226146	-3.043454	-0.865091
H	7.323681	-1.162949	0.335474
C	0.033416	2.236626	-2.834839
H	-1.010750	2.174953	-2.513722
H	0.535577	3.033834	-2.284866
H	0.018214	2.490288	-3.905529

Reaction E1

^{4}IE1

Fe	-0.000049	-0.000042	0.000008
N	-0.810580	1.416645	-1.166637
N	-0.812723	-1.413542	-1.169009
O	1.779822	-0.000190	-1.304248
C	-1.927508	0.003793	-2.842560
C	-1.565712	1.231296	-2.307212
C	-1.397381	3.462672	-2.063045
H	-1.457993	4.538391	-2.165326
C	4.105503	-0.000183	-1.657558
C	0.002836	3.423123	0.002684
C	-1.567560	-1.225148	-2.309287
C	-0.693404	2.782941	-1.010579
C	-1.937639	2.502121	-2.865893
H	-2.531001	2.628371	-3.762230
C	2.927203	-0.000523	-0.831226
H	3.042982	-0.001079	0.259987
C	-0.697644	-2.780279	-1.015215
C	-1.941433	-2.494469	-2.870064
H	-2.534973	-2.618327	-3.766613

C	-1.402664	-3.457180	-2.068801
H	-1.464913	-4.532637	-2.172853
C	5.380249	-0.000253	-1.186660
N	0.810582	-1.416669	1.166605
N	0.812760	1.413479	1.168967
C	1.927075	-0.003831	2.842791
C	1.565477	-1.231319	2.307336
C	1.397626	-3.462692	2.062856
H	1.458430	-4.538413	2.165008
C	-0.002380	-3.423191	-0.003008
C	1.567280	1.225085	2.309407
C	0.693684	-2.782978	1.010362
C	1.937511	-2.502128	2.865935
H	2.530722	-2.628387	3.762360
C	0.697893	2.780188	1.015007
C	1.941196	2.494393	2.870162
H	2.534548	2.618261	3.766824
C	1.402768	3.457099	2.068685
H	1.465155	4.532554	2.172638
O	-1.779782	0.000694	1.304261
C	-4.105475	0.001435	1.657638
C	-2.927186	0.000152	0.831283
H	-3.043003	-0.001258	-0.259933
C	-5.380230	0.000493	1.186745
H	0.003749	4.507956	0.003520
H	2.524104	-0.005045	3.748625
H	-0.003090	-4.508027	-0.003963
H	-2.524749	0.005017	-3.748270
H	3.920938	0.000245	-2.729060
H	-3.920904	0.003253	2.729135
C	-6.537764	0.002130	2.144698
H	-7.173010	0.880646	1.970285
H	-7.173116	-0.876888	1.973252
H	-6.218229	0.003881	3.189603
C	-5.769839	-0.002243	-0.267218
H	-4.931529	-0.003174	-0.965411
H	-6.392531	-0.881049	-0.478178
H	-6.393314	0.875229	-0.481357
C	6.537791	0.000231	-2.144602
H	6.218271	0.000547	-3.189513
H	7.172960	0.879034	-1.971367
H	7.173221	-0.878499	-1.971970
C	5.769832	-0.000771	0.267306
H	4.931509	-0.000866	0.965474
H	6.392788	-0.879062	0.479590
H	6.393036	0.877212	0.480141

^{6}IE1

Fe	0.002759	-0.256677	0.013438
N	-0.376643	-2.193642	0.546328
N	-1.011601	0.411568	1.678316
O	1.804645	-0.291178	1.193830
C	-1.663833	-1.776914	2.603952
C	-1.081022	-2.615114	1.651682
C	-0.421991	-4.490079	0.575089
H	-0.236221	-5.510392	0.265204
C	4.086113	0.141445	1.550628
C	0.786969	-3.321464	-1.307074
C	-1.627284	-0.380070	2.623108
C	0.039382	-3.318646	-0.127546
C	-1.114234	-4.056420	1.670790
H	-1.602700	-4.653722	2.429942
C	2.876025	0.186919	0.779274
H	2.888339	0.656955	-0.210701
C	-1.193859	1.723003	2.058949
C	-2.228241	0.462106	3.625851
H	-2.776244	0.103422	4.487752
C	-1.959961	1.758493	3.279117
H	-2.246990	2.660886	3.803737
C	5.281241	0.643101	1.137977
N	0.391108	1.706528	-0.526560
N	1.037831	-0.893337	-1.646843
C	1.624933	1.292167	-2.617826
C	1.057348	2.131181	-1.653910
C	0.440084	4.004574	-0.550670
H	0.267346	5.024614	-0.232020
C	-0.727690	2.837211	1.356298
C	1.613063	-0.104916	-2.618104
C	-0.001721	2.832264	0.163104
C	1.092496	3.572443	-1.671513
H	1.554880	4.170794	-2.446160
C	1.240602	-2.205578	-2.013298
C	2.208287	-0.949126	-3.624264
H	2.727444	-0.591138	-4.504124
C	1.979625	-2.243653	-3.250568
H	2.275823	-3.148241	-3.765970
O	-1.807540	-0.184333	-1.148954
C	-4.069194	0.266270	-1.597266

C	-2.908487	0.238229	-0.752851
H	-2.981987	0.598034	0.280071
C	-5.292763	0.716600	-1.208766
H	1.032753	-4.293120	-1.724182
H	2.119134	1.775756	-3.454901
H	-0.958836	3.809817	1.779880
H	-2.189094	-2.260643	3.421745
H	4.001988	-0.335742	2.523799
H	-3.921465	-0.105606	-2.608064
C	-6.440876	0.700309	-2.176890
H	-6.840562	1.714658	-2.307968
H	-6.159558	0.305764	-3.156180
H	-7.264730	0.094127	-1.777301
C	-5.628601	1.253298	0.156137
H	-4.795757	1.271370	0.860568
H	-6.019819	2.274349	0.062102
H	-6.436043	0.654208	0.596204
C	5.529154	1.323137	-0.181127
H	5.911805	2.336409	-0.004127
H	6.316676	0.787457	-0.726367
H	4.655386	1.397469	-0.830207
C	6.487446	0.538650	2.026650
H	7.287056	-0.014334	1.516080
H	6.890488	1.538160	2.237356
H	6.269369	0.042033	2.975150

^{4}IIE1O$_{endo\text{-}cis\text{-}p}$

C	-3.698585	2.301229	-0.273925
C	-4.001411	1.119573	0.560436
C	-3.168609	0.708188	1.539392
H	-2.284300	1.277775	1.796600
H	-3.352511	-0.195236	2.113216
C	-2.472357	2.697689	-0.647442
H	-1.606669	2.109207	-0.353030
C	-5.266753	0.396049	0.265881
C	-5.781304	0.364314	-1.041591
C	-5.982124	-0.274178	1.275243
C	-6.959026	-0.324125	-1.334496
H	-5.243510	0.871293	-1.837298
C	-7.152071	-0.972605	0.982536
H	-5.631192	-0.220480	2.301694
C	-7.645905	-1.002157	-0.325641
H	-7.338064	-0.333368	-2.352886
H	-7.692832	-1.475769	1.779462
H	-8.566074	-1.533817	-0.550804
H	-4.561537	2.869993	-0.621331
C	-2.189643	3.909953	-1.482509
H	-1.607373	3.642140	-2.374424
H	-3.108204	4.411464	-1.804716
H	-1.582005	4.632184	-0.921632
Fe	1.255828	0.187811	0.111424
N	1.516773	-1.799901	-0.121567
N	0.501640	-0.131559	1.940939
O	3.267448	0.344506	1.035374
C	0.601128	-2.590310	2.020648
C	1.173642	-2.792993	0.773128
C	2.008302	-3.869985	-1.019744
H	2.355415	-4.600730	-1.738817
C	5.574220	-0.103174	1.130974
C	2.478566	-1.830463	-2.386517
C	0.301658	-1.348942	2.562067
C	2.022683	-2.448323	-1.230393
C	1.480338	-4.083447	0.219746
H	1.308953	-5.024860	0.725570
C	4.261005	-0.217974	0.551121
H	4.129773	-0.824342	-0.354401
C	0.112351	0.825910	2.854982
C	-0.248583	-1.148289	3.874958
H	-0.499561	-1.944694	4.563545
C	-0.359445	0.198329	4.058403
H	-0.726204	0.732447	4.925183
C	6.693098	-0.697184	0.640119
N	1.041588	2.157501	0.360328
N	2.055639	0.496484	-1.706425
C	1.898097	2.947823	-1.806779
C	1.363709	3.148438	-0.543990
C	0.597702	4.230787	1.276065
H	0.279165	4.962301	2.007136
C	0.146975	2.196621	2.650880
C	2.216955	1.709195	-2.342067
C	0.576443	2.808641	1.484153
C	1.083229	4.441194	0.019707
H	1.246318	5.381196	-0.491139
C	2.481236	-0.460255	-2.604539
C	2.768788	1.510362	-3.654683
H	2.992330	2.306006	-4.353422
C	2.934522	0.167025	-3.816659
H	3.320270	-0.365536	-4.676332

O	-0.656724	-0.028450	-0.897991
C	-2.547247	-1.304962	-1.459536
C	-1.510628	-0.872740	-0.565277
H	-1.432648	-1.331748	0.426346
C	-3.477486	-2.251215	-1.166168
H	2.845424	-2.467001	-3.184940
H	2.089251	3.824291	-2.416759
H	-0.193675	2.835063	3.458930
H	0.385821	-3.466487	2.623183
H	5.633352	0.513734	2.024426
H	-2.536962	-0.836680	-2.440382
C	-4.465650	-2.692716	-2.205678
H	-4.314380	-3.756534	-2.434483
H	-5.487578	-2.590595	-1.822402
H	-4.381051	-2.120792	-3.133078
C	-3.634044	-2.923374	0.169326
H	-3.696538	-4.010673	0.040503
H	-2.839252	-2.703813	0.883698
H	-4.585611	-2.598193	0.608947
C	8.013881	-0.502305	1.329659
H	8.428269	-1.471476	1.637675
H	8.742693	-0.061642	0.636494
H	7.936518	0.140281	2.210050
C	6.746588	-1.572905	-0.582938
H	7.140732	-2.560273	-0.310405
H	5.788162	-1.715562	-1.084383
H	7.453016	-1.148466	-1.307737
H	6.787788	0.550186	-2.968677
H	7.610311	1.220920	1.202137
H	8.212343	1.544080	-1.189382
H	3.977908	-2.586028	-1.521698
C	1.549404	-3.793308	-1.617305
H	0.702682	-3.324843	-2.137979
H	2.294111	-4.096571	-2.358978
H	1.140559	-4.680190	-1.117893
Fe	-1.062114	-0.341255	0.201229
N	-1.055640	1.681329	0.214549
N	-0.671572	-0.347347	2.177332
O	-3.290146	-0.220374	0.815703
C	-0.598096	2.075127	2.597452
C	-0.824168	2.502192	1.295160
C	-0.971978	3.879617	-0.481610
H	-0.999699	4.725657	-1.156155
C	-5.463259	0.637730	0.515276
C	-1.414857	2.092577	-2.180535
C	-0.511882	0.747268	2.997776
C	-1.145212	2.507702	-0.882899
C	-0.784728	3.877359	0.868774
H	-0.623562	4.720564	1.528041
C	-4.053518	0.543334	0.213757
H	-3.640856	1.176581	-0.584127
C	-0.456601	-1.453326	2.968041
C	-0.182533	0.318006	4.332202
H	-0.011127	0.984437	5.167703
C	-0.135082	-1.044378	4.310731
H	0.076096	-1.723523	5.126625
C	-6.342679	1.447522	-0.126233
N	-1.296676	-2.334289	0.243742
N	-1.654413	-0.311745	-1.719273
C	-2.102307	-2.705419	-2.048409
C	-1.755092	-3.137818	-0.775511
C	-1.298744	-4.537791	0.930057
H	-1.167556	-5.390941	1.583142
C	-0.587098	-2.773770	2.557063
C	-2.022511	-1.391459	-2.489373
C	-1.023341	-3.173877	1.301290
C	-1.768128	-4.513294	-0.350099
H	-2.091624	-5.343736	-0.964386
C	-1.671773	0.781159	-2.558144
C	-2.287679	-0.966407	-3.839510
H	-2.600660	-1.623774	-4.640439

^{4}TS1E1O$_{endo\text{-}cis\text{-}p}$

C	3.325258	-2.261538	-0.715047
C	3.790872	-1.204540	0.123565
C	2.973205	-0.660133	1.151562
H	2.284150	-1.328020	1.662522
H	3.459102	0.040049	1.825792
C	2.090143	-2.828882	-0.622420
H	1.432200	-2.580054	0.201420
C	5.030392	-0.499977	-0.233210
C	5.401450	-0.328954	-1.583389
C	5.843739	0.079902	0.762092
C	6.530510	0.409636	-1.923104
H	4.771959	-0.730358	-2.370636
C	6.984535	0.799636	0.420988
H	5.601404	-0.068921	1.809590
C	7.326740	0.974484	-0.923042

C	-2.058573	0.376393	-3.884931
H	-2.148842	1.046643	-4.730057
O	0.877582	-0.370035	-0.315281
C	2.471427	1.368979	-0.598696
C	1.758759	0.411882	0.286493
H	1.409402	0.857332	1.223525
C	3.105421	2.491133	-0.220961
H	-1.462003	2.854755	-2.951451
H	-2.432831	-3.454933	-2.760172
H	-0.397204	-3.548724	3.292461
H	-0.437681	2.836902	3.353402
H	-5.806087	-0.006579	1.321552
H	2.460250	1.083397	-1.648639
C	3.817505	3.353329	-1.230268
H	3.441389	4.384756	-1.204128
H	4.891029	3.402081	-1.000455
H	3.707916	2.970132	-2.249077
C	3.214757	2.963103	1.207440
H	2.965620	4.029206	1.280700
H	2.565189	2.420852	1.899346
H	4.250348	2.856680	1.558962
C	-7.791355	1.459608	0.275740
H	-8.096427	2.472319	0.571120
H	-8.425519	1.182425	-0.576909
H	-8.001840	0.776950	1.102813
C	-5.998844	2.380709	-1.257221
H	-6.257135	3.410035	-0.976826
H	-4.950608	2.362579	-1.559120
H	-6.611389	2.136836	-2.134696

^{6}TS1E1O$_{endo\text{-}cis\text{-}p}$

C	3.352892	-2.290673	-0.496919
C	3.753826	-1.155003	0.280887
C	2.872891	-0.520164	1.187561
H	2.100225	-1.111684	1.669562
H	3.304786	0.236156	1.836234
C	2.111221	-2.842435	-0.471441
H	1.370450	-2.483032	0.232135
C	5.033624	-0.500806	-0.027633
C	5.526365	-0.466500	-1.349169
C	5.765929	0.166039	0.976235
C	6.695010	0.223588	-1.655499
H	4.963220	-0.937706	-2.147952
C	6.945911	0.837204	0.671540
H	5.429400	0.122158	2.007198
C	7.410009	0.875191	-0.646731
H	7.047239	0.257628	-2.682061
H	7.508129	1.325965	1.461640
H	8.327383	1.405356	-0.885468
H	4.083719	-2.703356	-1.187824
C	1.661676	-3.926972	-1.384293
H	0.849329	-3.546841	-2.019304
H	2.465777	-4.303202	-2.023315
H	1.225493	-4.755515	-0.812503
Fe	-1.009205	-0.315135	0.080706
N	-1.129652	1.769543	0.178169
N	-0.601010	-0.350203	2.118528
O	-3.178486	-0.291412	0.809089
C	-0.502307	2.073330	2.539751
C	-0.830207	2.552137	1.266616
C	-1.173724	3.978111	-0.454496
H	-1.284756	4.847108	-1.090520
C	-5.397967	0.489058	0.745273
C	-1.692026	2.200800	-2.178369
C	-0.399878	0.734842	2.935632
C	-1.340180	2.610062	-0.887729
C	-0.863056	3.942822	0.875185
H	-0.669476	4.777125	1.537418
C	-4.014852	0.485660	0.331990
H	-3.690913	1.200254	-0.435847
C	-0.431351	-1.478943	2.882531
C	-0.060092	0.273859	4.261882
H	0.148519	0.917318	5.107130
C	-0.076260	-1.091864	4.228367
H	0.116291	-1.780952	5.040767
C	-6.353758	1.313716	0.247136
N	-1.278045	-2.359556	0.104339
N	-1.771738	-0.236642	-1.838263
C	-2.096223	-2.649821	-2.199045
C	-1.736523	-3.130426	-0.936078
C	-1.352890	-4.553770	0.779398
H	-1.265088	-5.418430	1.424904
C	-0.621154	-2.791817	2.437837
C	-2.100669	-1.317208	-2.620303
C	-1.051807	-3.196641	1.171131
C	-1.781751	-4.511777	-0.517714
H	-2.107135	-5.336554	-1.138864
C	-1.900071	0.889119	-2.616134

C	-2.452518	-0.855930	-3.943338
H	-2.756070	-1.496377	-4.761629
C	-2.324092	0.503538	-3.942257
H	-2.503881	1.190931	-4.759054
O	0.870411	-0.261035	-0.470430
C	2.581246	1.340448	-0.838643
C	1.751274	0.549407	0.091660
H	1.385144	1.099008	0.961628
C	3.259193	2.463539	-0.542145
H	-1.831804	2.986035	-2.915282
H	-2.414079	-3.389998	-2.927308
H	-0.467493	-3.577742	3.171174
H	-0.300210	2.820637	3.301328
H	-5.653002	-0.238505	1.512236
H	2.638134	0.923442	-1.841726
C	4.108224	3.146769	-1.581070
H	3.822360	4.200639	-1.694294
H	5.163297	3.135083	-1.273820
H	4.035896	2.660272	-2.558042
C	3.282048	3.107753	0.820793
H	3.060283	4.179301	0.740475
H	2.570691	2.672735	1.527178
H	4.287680	3.025975	1.255190
C	-7.766713	1.225959	0.752858
H	-8.084148	2.192645	1.165976
H	-8.453629	1.003361	-0.074529
H	-7.889302	0.460493	1.523184
C	-6.132400	2.358539	-0.814454
H	-6.414395	3.344960	-0.424339
H	-5.108738	2.419239	-1.186637
H	-6.795482	2.162738	-1.666885

^{4}IIIE1O$_{endo\text{-}cis\text{-}p}$

C	-2.948027	-0.101185	-1.525204
C	-3.556781	-0.113942	-0.328522
C	-2.851406	-0.721811	0.866425
H	-2.451322	0.067419	1.507677
H	-3.568111	-1.284719	1.476434
C	-1.549870	-0.583881	-1.778278
H	-0.976416	0.276614	-2.123597
C	-4.899970	0.475465	-0.113548
C	-5.879080	0.461475	-1.121872
C	-5.222965	1.074934	1.116395
C	-7.127349	1.044730	-0.916078
H	-5.668165	-0.035683	-2.064517
C	-6.471386	1.661604	1.321688
H	-4.488033	1.098566	1.916261
C	-7.428550	1.650802	0.306042
H	-7.871952	1.014163	-1.706727
H	-6.697442	2.125779	2.277922
H	-8.403939	2.100459	0.468096
H	-3.434567	0.347300	-2.388230
C	-1.419045	-1.667484	-2.852888
H	-0.366748	-1.947812	-2.960639
H	-1.998799	-2.558932	-2.611223
H	-1.770840	-1.269050	-3.811500
Fe	0.847730	0.323970	0.102425
N	1.771189	-1.299804	0.833579
N	0.076498	0.699257	1.904248
O	2.605905	1.548726	0.666231
C	1.119453	-1.116050	3.198033
C	1.802784	-1.715780	2.150634
C	3.048666	-3.213763	1.021265
H	3.671308	-4.044648	0.715855
C	4.916533	1.978477	0.753459
C	2.794106	-2.162600	-1.231676
C	0.302717	-0.002897	3.071654
C	2.529928	-2.213520	0.129556
C	2.607183	-2.900228	2.273450
H	2.790567	-3.423972	3.202572
C	3.764085	1.193411	0.397570
H	3.909171	0.243076	-0.132451
C	-0.789704	1.720940	2.240206
C	-0.446289	0.580092	4.150706
H	-0.440964	0.213979	5.169095
C	-1.128579	1.642088	3.634470
H	-1.795220	2.326081	4.143327
C	6.202255	1.637185	0.475189
N	-0.000904	1.987672	-0.612205
N	1.678737	-0.019059	-1.693548
C	0.933283	1.997842	-2.889099
C	0.170564	2.540785	-1.866243
C	-1.274754	3.904740	-0.807714
H	-1.962941	4.690226	-0.524344
C	-1.264541	2.693494	1.374092
C	1.606015	0.786553	-2.812654
C	-0.873482	2.824569	0.051659
C	-0.616807	3.737782	-1.989336

H	-0.658228	4.356022	-2.876605
C	2.404766	-1.130787	-2.073258
C	2.294750	0.167051	-3.911043
H	2.382612	0.595984	-4.900767
C	2.777918	-1.025492	-3.457375
H	3.347185	-1.770287	-3.998366
O	-0.877635	-0.967117	-0.547319
C	-2.261445	-2.973045	-0.068610
C	-1.724011	-1.661822	0.441254
H	-1.033501	-1.817957	1.270064
C	-1.644359	-4.163942	-0.032171
H	3.385945	-2.964946	-1.659573
H	0.968002	2.534627	-3.831090
H	-1.957785	3.427600	1.770176
H	1.214744	-1.561586	4.182486
H	4.701755	2.905233	1.279979
H	-3.258956	-2.921320	-0.502316
C	-2.315969	-5.399849	-0.575488
H	-1.714504	-5.844769	-1.380152
H	-2.408696	-6.167748	0.204130
H	-3.314709	-5.190952	-0.970369
C	-0.254699	-4.382022	0.502574
H	0.430318	-4.635544	-0.317577
H	0.152444	-3.507829	1.008890
H	-0.234244	-5.227538	1.201881
C	7.328211	2.538703	0.895379
H	8.020416	1.999001	1.555185
H	7.914607	2.847350	0.019727
H	6.977625	3.434079	1.414326
C	6.633319	0.389091	-0.246981
H	7.319986	-0.184324	0.388727
H	5.817392	-0.269544	-0.548329
H	7.201412	0.662767	-1.145291

^{4}TS2E1O$_{endo\text{-}cis\text{-}p}$

C	1.809833	-2.741656	-0.646811
C	2.551078	-1.791992	0.129847
C	1.922807	-0.957374	1.086378
H	1.040768	-1.328590	1.599977
H	2.579046	-0.376330	1.727949
C	0.465745	-2.925386	-0.563716
H	-0.108555	-2.402196	0.190864
C	3.949823	-1.520818	-0.227357
C	4.378808	-1.591754	-1.569839
C	4.882836	-1.121643	0.752422
C	5.683037	-1.256789	-1.919134
H	3.672777	-1.858777	-2.349130
C	6.192748	-0.808828	0.403827
H	4.590304	-1.098512	1.797405
C	6.594819	-0.866824	-0.934102
H	5.988980	-1.295517	-2.960205
H	6.903332	-0.525726	1.174581
H	7.615610	-0.615141	-1.206497
H	2.360593	-3.318567	-1.385325
C	-0.311840	-3.808549	-1.474194
H	-1.027980	-3.198234	-2.041742
H	0.323111	-4.355343	-2.177088
H	-0.917865	-4.518472	-0.897226
Fe	-1.773995	0.355886	0.226269
N	-1.418509	2.337244	0.271423
N	-1.341584	0.271271	2.190630
C	-0.586877	2.582431	2.572124
C	-0.875726	3.071025	1.308120
C	-0.967290	4.495489	-0.430466
H	-0.913631	5.347803	-1.095121
C	-1.960321	2.890671	-2.061048
C	-0.800777	1.273453	2.975773
C	-1.472465	3.202884	-0.803567
C	-0.603902	4.416046	0.879928
H	-0.188260	5.188681	1.513582
C	-1.358612	-0.855945	2.993023
C	-0.468385	0.763691	4.276427
H	-0.040725	1.354979	5.075677
C	-0.806527	-0.557149	4.284628
H	-0.716102	-1.271122	5.092958
N	-2.612356	-1.468009	0.368162
N	-2.631958	0.576471	-1.570396
C	-3.620825	-1.653678	-1.867598
C	-3.312067	-2.148531	-0.611066
C	-3.133042	-3.602088	1.097834
H	-3.187865	-4.454389	1.762625
C	-1.876563	-2.089402	2.632572
C	-3.287502	-0.385427	-2.312471
C	-2.498935	-2.355087	1.423755
C	-3.646719	-3.469835	-0.158324
H	-4.204512	-4.193536	-0.738408
C	-2.515883	1.670049	-2.405511
C	-3.585944	0.113825	-3.627183

H	-4.105690	-0.447526	-4.392642
C	-3.100640	1.384724	-3.687329
H	-3.140948	2.084447	-4.511872
O	0.001254	-0.117353	-0.455379
C	2.070658	0.959363	-0.894110
C	1.098426	0.402230	0.070713
H	0.940690	1.017548	0.961082
C	3.068800	1.816151	-0.614151
H	-1.938899	3.667140	-2.818256
H	-4.160662	-2.303243	-2.548531
H	-1.839840	-2.886595	3.367460
H	-0.157579	3.269497	3.293682
H	1.945318	0.588097	-1.909013
C	4.027133	2.260846	-1.686811
H	4.061158	3.355779	-1.757835
H	5.045636	1.926664	-1.444445
H	3.764182	1.856595	-2.668521
C	3.359058	2.360037	0.762099
H	3.505266	3.446497	0.721953
H	2.572436	2.155474	1.493238
H	4.295998	1.930622	1.142040

^{6}TS2E1O$_{endo\text{-}cis\text{-}p}$

C	1.896655	-2.673762	-0.712079
C	2.641862	-1.707218	0.047342
C	2.033968	-0.896764	1.027586
H	1.147228	-1.255243	1.540453
H	2.687043	-0.293837	1.650840
C	0.563880	-2.896510	-0.585721
H	-0.000229	-2.385547	0.185734
C	4.025349	-1.410185	-0.349688
C	4.415470	-1.468478	-1.704445
C	4.979253	-0.997323	0.603778
C	5.702519	-1.107995	-2.090818
H	3.692561	-1.748430	-2.463546
C	6.272138	-0.658337	0.217998
H	4.717722	-0.984737	1.657171
C	6.635534	-0.704127	-1.131596
H	5.978441	-1.138374	-3.140494
H	7.000065	-0.365488	0.968662
H	7.643230	-0.433344	-1.432862
H	2.440343	-3.237099	-1.466004
C	-0.220429	-3.809641	-1.459617
H	-0.983896	-3.230493	-1.997170
H	0.403191	-4.336612	-2.187223
H	-0.774902	-4.538778	-0.855615
Fe	-1.663247	0.268860	0.185568
N	-1.458235	2.293909	0.626984
N	-1.285070	-0.102532	2.205215
C	-0.468992	2.121949	2.872577
C	-0.844673	2.836013	1.736237
C	-1.120859	4.552505	0.298571
H	-1.144430	5.516449	-0.193110
C	-2.226433	3.216850	-1.521563
C	-0.681346	0.762069	3.095307
C	-1.634337	3.330222	-0.266604
C	-0.632758	4.247630	1.535474
H	-0.177910	4.912284	2.258642
C	-1.339786	-1.332062	2.832502
C	-0.324448	0.052817	4.297063
H	0.155391	0.499834	5.158255
C	-0.731016	-1.240552	4.134825
H	-0.648598	-2.060606	4.836517
N	-2.652045	-1.564615	0.133336
N	-2.774777	0.819277	-1.472233
C	-3.716662	-1.368593	-2.079379
C	-3.378769	-2.072357	-0.925205
C	-3.253574	-3.746695	0.582832
H	-3.348675	-4.676902	1.128301
C	-1.929376	-2.477267	2.302405
C	-3.444009	-0.026872	-2.330454
C	-2.571731	-2.575382	1.070092
C	-3.753139	-3.435183	-0.649319
H	-4.335309	-4.061301	-1.313122
C	-2.765938	2.058363	-2.075334
C	-3.852622	0.696439	-3.508369
H	-4.401388	0.267110	-4.336592
C	-3.432562	1.984433	-3.351270
H	-3.569480	2.820413	-4.024895
O	0.084083	-0.033532	-0.543124
C	2.142013	1.066041	-0.970367
C	1.171532	0.510028	-0.018201
H	1.011701	1.092690	0.892064
C	3.143914	1.916421	-0.673535
H	-2.296980	4.123186	-2.114813
H	-4.276883	-1.907529	-2.837101
H	-1.922131	-3.366347	2.925330
H	0.010892	2.681810	3.669386

H	2.024984	0.701779	-1.988384
C	4.112911	2.361222	-1.734860
H	4.163137	3.456191	-1.789845
H	5.124140	2.007912	-1.488970
H	3.850914	1.973413	-2.723152
C	3.424356	2.448118	0.708627
H	3.574211	3.534110	0.676802
H	2.632817	2.241181	1.433469
H	4.358070	2.013023	1.089637

^{6}TS1E1O$_{endo\text{-}trans\text{-}p}$

C	1.935916	-1.447074	2.698213
C	3.008059	-0.746621	2.052591
C	3.186235	0.658181	2.151135
H	2.791707	1.155913	3.028456
H	4.145607	1.054960	1.830590
C	0.973498	-0.990149	3.551203
C	3.818811	-1.476850	1.071891
C	3.218691	-2.453385	0.247396
C	5.170447	-1.143468	0.843269
C	3.938755	-3.052904	-0.779630
H	2.167735	-2.691373	0.362294
C	5.896795	-1.770430	-0.163713
H	5.662881	-0.418213	1.483230
C	5.280685	-2.719796	-0.984698
H	3.445948	-3.774838	-1.423627
H	6.943119	-1.519892	-0.310389
H	5.845601	-3.198256	-1.779523
H	1.888679	-2.510373	2.484123
Fe	-0.751675	0.563584	0.045544
N	-0.143604	1.388114	-1.750783
N	-1.579368	2.381347	0.597096
O	-2.749107	0.377592	-1.107732
C	-0.677421	3.737936	-1.241513
C	-0.136817	2.729237	-2.044131
C	0.805396	1.692918	-3.819080
H	1.267823	1.447211	-4.766561
C	-4.686825	-0.768863	-1.796127
C	0.590081	-0.654302	-2.903568
C	-1.356646	3.577575	-0.029630
C	0.412011	0.729035	-2.816564
C	0.474995	2.927605	-3.338716
H	0.608926	3.888424	-3.819163
C	-3.428311	-0.654947	-1.095531
H	-3.061271	-1.520240	-0.528446
C	-2.277576	2.637827	1.746156
C	-1.945730	4.642357	0.755096
H	-1.925133	5.691674	0.489924
C	-2.517228	4.062111	1.849311
H	-3.054908	4.543452	2.656196
C	-5.468840	-1.877234	-1.809780
N	-1.756937	-0.357702	1.619653
N	-0.333731	-1.337720	-0.722125
C	-1.189601	-2.704908	1.139871
C	-1.761921	-1.697732	1.924128
C	-2.893894	-0.669631	3.590922
H	-3.467907	-0.432298	4.477729
C	-2.679938	1.673337	2.673743
C	-0.544960	-2.541199	-0.090554
C	-2.447098	0.295096	2.613618
C	-2.473035	-1.899390	3.165318
H	-2.637404	-2.860047	3.637419
C	0.245159	-1.607574	-1.940135
C	-0.057417	-3.610854	-0.931612
H	-0.101263	-4.663962	-0.683283
C	0.427800	-3.034189	-2.072533
H	0.855761	-3.525076	-2.937593
O	0.856209	0.919082	1.028631
C	2.705966	0.939636	-0.484893
C	2.090984	1.337036	0.797541
H	2.261431	2.381237	1.090327
C	3.693387	1.567476	-1.149701
H	1.040537	-1.029855	-3.817522
H	-1.285050	-3.722337	1.508021
H	-3.235543	2.031242	3.535433
H	-0.588028	4.753404	-1.615764
H	-4.995629	0.121817	-2.338350
H	2.293693	0.025954	-0.898202
C	0.709044	0.368930	4.116288
H	0.516187	1.084918	3.311943
H	-0.164466	0.341847	4.770555
H	1.563060	0.732645	4.703902
H	0.277516	-1.757623	3.882556
C	4.383546	2.821182	-0.681752
H	3.971224	3.237304	0.240774
H	5.451805	2.624973	-0.516406
H	4.328622	3.598185	-1.455031
C	4.195911	1.009799	-2.455190

H	4.073098	1.743660	-3.262431
H	5.268902	0.783490	-2.389878
H	3.668756	0.094368	-2.737040
C	-6.764107	-1.880518	-2.573194
H	-6.757541	-2.675075	-3.331223
H	-7.602856	-2.102667	-1.900071
H	-6.959367	-0.926846	-3.069944
C	-5.156678	-3.169320	-1.101265
H	-5.133483	-3.991580	-1.827924
H	-4.212723	-3.171594	-0.554145
H	-5.962590	-3.404991	-0.394266

^{4}TS1E1O$_{exo\text{-}cis\text{-}p}$

Fe	0.700832	0.418165	-0.059887
N	0.860772	2.378601	-0.500297
N	-0.414598	0.144870	-1.718617
O	2.508346	0.155181	-1.484263
C	-0.517330	2.464012	-2.530266
C	0.262003	3.045884	-1.541320
C	1.292294	4.638855	-0.328499
H	1.693202	5.560559	0.072976
C	4.610109	-0.756332	-2.033325
C	2.237906	3.095377	1.401274
C	-0.834963	1.114786	-2.601707
C	1.494235	3.335179	0.254266
C	0.533332	4.458650	-1.444526
H	0.180983	5.201816	-2.148195
C	3.455032	-0.579488	-1.182672
H	3.416791	-1.120846	-0.227309
C	-0.983274	-1.034113	-2.145119
C	-1.693998	0.532355	-3.599634
H	-2.153297	1.082246	-4.410980
C	-1.792338	-0.797108	-3.311619
H	-2.349497	-1.562328	-3.835951
C	5.671684	-1.550905	-1.747629
N	0.734956	-1.582401	0.237072
N	1.981525	0.648917	1.474890
C	2.201704	-1.687677	2.208199
C	1.384204	-2.263401	1.242300
C	0.209350	-3.827005	0.122496
H	-0.248338	-4.737852	-0.241320
C	-0.803459	-2.276414	-1.554433
C	2.463027	-0.327823	2.318203
C	0.008510	-2.525629	-0.458170
C	1.067886	-3.666759	1.171510
H	1.458448	-4.420360	1.843631
C	2.468387	1.844442	1.956480
C	3.277281	0.266433	3.346416
H	3.778194	-0.286485	4.130810
C	3.275595	1.611607	3.125574
H	3.776088	2.387171	3.690856
O	-0.856495	0.773029	1.125404
C	-2.419238	2.530939	0.872613
C	-2.065730	1.094550	0.696112
H	-2.318281	0.690042	-0.289108
C	-3.151605	3.291740	0.040658
H	2.687323	3.951067	1.894531
H	2.654498	-2.347203	2.941921
H	-1.335918	-3.117443	-1.985336
H	-0.926102	3.115425	-3.295697
H	4.587869	-0.192299	-2.962927
H	-1.953316	2.982723	1.746989
C	-1.858562	-1.682390	2.368930
C	-2.964078	-1.149878	1.628505
C	-3.306040	0.231252	1.708176
H	-4.261001	0.519833	1.277121
H	-3.135748	0.714885	2.660364
C	-1.120724	-1.114141	3.368088
C	-3.569202	-2.000524	0.594615
C	-3.513640	-3.409496	0.688223
C	-4.202515	-1.440633	-0.536638
C	-4.053467	-4.216536	-0.306587
H	-3.073363	-3.876908	1.562100
C	-4.721477	-2.249799	-1.541755
H	-4.266670	-0.365647	-0.653036
C	-4.649486	-3.640728	-1.432899
H	-4.015627	-5.296811	-0.202230
H	-5.184542	-1.793017	-2.411258
H	-5.065056	-4.271896	-2.213036
H	-1.472227	-2.632052	2.015641
H	-0.240649	-1.693937	3.640346
C	-1.292740	0.136044	4.169895
H	-2.338903	0.351819	4.409002
H	-0.734218	0.063269	5.107176
H	-0.890304	0.981458	3.599488
C	-3.310125	4.769429	0.292787
H	-4.370672	5.053451	0.319619
H	-2.844862	5.081878	1.232045

H	-2.851559	5.345222	-0.522908
C	-3.852256	2.787638	-1.196342
H	-3.582446	1.766029	-1.472821
H	-4.941653	2.832248	-1.061174
H	-3.619596	3.432775	-2.053167
C	5.825004	-2.380891	-0.500165
H	5.951920	-3.436372	-0.773378
H	6.741253	-2.086604	0.027778
H	4.991051	-2.308637	0.199751
C	6.820233	-1.655680	-2.712220
H	7.755907	-1.350047	-2.225298
H	6.961225	-2.698878	-3.025020
H	6.674884	-1.041120	-3.604159

^{6}TS1E1O$_{exo\text{-}cis\text{-}p}$

Fe	0.678578	0.426085	-0.026010
N	0.923244	2.426197	-0.505448
N	-0.446165	0.165454	-1.750039
O	2.431645	0.151895	-1.459709
C	-0.529903	2.516241	-2.484402
C	0.296402	3.100055	-1.520431
C	1.426856	4.659342	-0.336698
H	1.866719	5.571541	0.045761
C	4.523395	-0.742652	-2.062587
C	2.388548	3.050291	1.365121
C	-0.872029	1.164820	-2.592740
C	1.617848	3.344124	0.235754
C	0.611709	4.508334	-1.420834
H	0.255057	5.272753	-2.099414
C	3.392147	-0.576700	-1.181349
H	3.381951	-1.116581	-0.225977
C	-1.005824	-1.009877	-2.185025
C	-1.747635	0.597757	-3.591924
H	-2.224280	1.157745	-4.386497
C	-1.832756	-0.742848	-3.337984
H	-2.395461	-1.491468	-3.880395
C	5.601434	-1.524181	-1.801103
N	0.733913	-1.641045	0.223511
N	2.060473	0.608592	1.496475
C	2.197872	-1.745049	2.200162
C	1.371092	-2.324357	1.230069
C	0.186916	-3.867623	0.075304
H	-0.272394	-4.774484	-0.296836
C	-0.804774	-2.262995	-1.599735
C	2.512027	-0.387694	2.328001
C	0.002089	-2.555549	-0.498412
C	1.034910	-3.726872	1.139100
H	1.404936	-4.497609	1.803674
C	2.595505	1.794824	1.943928
C	3.364865	0.189621	3.340215
H	3.859986	-0.367815	4.125316
C	3.414490	1.534504	3.104247
H	3.958017	2.288406	3.659364
O	-0.854834	0.813692	1.114332
C	-2.448129	2.547909	0.902612
C	-2.055360	1.140651	0.685700
H	-2.332564	0.713441	-0.280211
C	-3.241645	3.287842	0.104282
H	2.884991	3.889133	1.843775
H	2.636735	-2.417626	2.931579
H	-1.338007	-3.096902	-2.045065
H	-0.954454	3.183276	-3.228734
H	4.470700	-0.181743	-2.992716
H	-1.972171	3.006389	1.767320
C	-1.827282	-1.682972	2.369427
C	-2.948135	-1.149974	1.640573
C	-3.339221	0.201635	1.764756
H	-4.290120	0.494350	1.330353
H	-3.129596	0.704593	2.697236
C	-1.110572	-1.142330	3.394862
C	-3.540733	-1.994713	0.589940
C	-3.466462	-3.403043	0.663742
C	-4.184998	-1.427974	-0.530692
C	-4.002127	-4.203425	-0.339368
H	-3.014389	-3.877083	1.527844
C	-4.700840	-2.229376	-1.543703
H	-4.262426	-0.352311	-0.632665
C	-4.611921	-3.620582	-1.454059
H	-3.949201	-5.284410	-0.249873
H	-5.172745	-1.766065	-2.404956
H	-5.023624	-4.246187	-2.240684
H	-1.429733	-2.617794	1.990671
H	-0.235436	-1.726947	3.672039
C	-1.296753	0.101645	4.204462
H	-2.344929	0.292576	4.458056
H	-0.726164	0.038980	5.134860
H	-0.920867	0.961023	3.635933
C	-3.462433	4.749948	0.390748

H	-4.534644	4.975873	0.465184
H	-2.976172	5.070284	1.316379
H	-3.070436	5.362430	-0.432522
C	-3.957163	2.769825	-1.117415
H	-3.635161	1.775510	-1.433909
H	-5.040632	2.741502	-0.937405
H	-3.801504	3.450939	-1.963337
C	5.797072	-2.346445	-0.554735
H	5.931828	-3.401174	-0.826975
H	6.722323	-2.036942	-0.051882
H	4.980522	-2.282723	0.166083
C	6.725632	-1.618845	-2.794661
H	7.670053	-1.302388	-2.332187
H	6.869553	-2.661401	-3.108246
H	6.551300	-1.008148	-3.684031

^{4}TS1E1O$_{exo\text{-}trans\text{-}p}$

Fe	-0.664097	-0.352993	-0.059336
N	-1.270789	1.564929	0.168933
N	0.595032	-0.130720	1.489496
O	-2.255074	-0.900946	1.525636
C	0.092972	2.196515	2.111788
C	-0.833891	2.465490	1.113026
C	-2.206248	3.633528	-0.239930
H	-2.805241	4.390993	-0.728920
C	-4.443537	-0.736832	2.382890
C	-2.800233	1.720998	-1.746800
C	0.766062	0.992533	2.270648
C	-2.107402	2.263780	-0.672018
C	-1.428771	3.754986	0.874189
H	-1.257145	4.633441	1.482936
C	-3.392209	-0.422426	1.443658
H	-3.610835	0.272189	0.620831
C	1.481256	-1.068287	1.968809
C	1.790750	0.754774	3.252984
H	2.117605	1.479611	3.987286
C	2.244590	-0.515229	3.055498
H	3.023948	-1.042772	3.588449
C	-5.706083	-0.242642	2.328344
N	-0.253486	-2.312878	-0.108491
N	-2.075130	-0.613054	-1.464186
C	-1.934809	-3.049590	-1.743281
C	-0.908564	-3.279846	-0.837442
C	0.700993	-4.374008	0.297545
H	1.368110	-5.109396	0.728713
C	1.574342	-2.382704	1.533726
C	-2.444341	-1.797511	-2.060344
C	0.726665	-2.967107	0.603805
C	-0.325159	-4.570531	-0.578773
H	-0.661821	-5.497545	-1.024758
C	-2.799017	0.376226	-2.094061
C	-3.424297	-1.548282	-3.085206
H	-3.880498	-2.314376	-3.698822
C	-3.631891	-0.201236	-3.116823
H	-4.297398	0.362156	-3.758166
O	0.683201	0.258661	-1.451880
C	1.089755	2.526851	-1.994823
C	1.433577	1.308443	-1.222943
H	1.705563	1.475754	-0.176541
C	1.301086	3.802290	-1.622845
H	-3.425980	2.388870	-2.329816
H	-2.343068	-3.906659	-2.268867
H	2.315780	-3.016016	2.009211
H	0.338672	3.001048	2.797264
H	-4.162338	-1.424161	3.177365
H	0.563584	2.313408	-2.923439
C	3.033188	-1.498901	-1.681029
C	3.581096	-0.253808	-1.228466
C	3.211680	0.949682	-1.868212
H	3.759641	1.851103	-1.615307
H	2.969532	0.901133	-2.923902
C	2.193592	-1.620755	-2.743971
H	1.995332	-0.766901	-3.382440
C	4.370982	-0.226622	0.014545
C	5.048460	-1.377271	0.472173
C	4.484952	0.951262	0.782409
C	5.804556	-1.348738	1.639778
H	5.019985	-2.292209	-0.109437
C	5.230262	0.974512	1.956447
H	3.968692	1.853685	0.476500
C	5.895812	-0.173650	2.390696
H	6.333128	-2.242133	1.959158
H	5.290432	1.891518	2.535124
H	6.486301	-0.151930	3.302035
H	3.193413	-2.380597	-1.069678
C	1.517460	-2.881535	-3.148139
H	1.851105	-3.183448	-4.151106
H	1.698908	-3.700386	-2.447942

H	0.435792	-2.714069	-3.217190
C	0.796678	4.943243	-2.467042
H	1.612790	5.629531	-2.730496
H	0.325760	4.597441	-3.391790
H	0.059592	5.533037	-1.905191
C	2.024248	4.218726	-0.366684
H	1.446719	4.983593	0.167373
H	2.203438	3.395504	0.328325
H	2.991311	4.676219	-0.617479
C	-6.221954	0.709098	1.281230
H	-7.077659	0.256907	0.763462
H	-6.599563	1.619354	1.764401
H	-5.486291	1.001425	0.530600
C	-6.719433	-0.640503	3.365187
H	-7.099375	0.247959	3.886966
H	-7.588077	-1.113136	2.887703
H	-6.310761	-1.331525	4.106755

^{6}TS1E1O$_{exo\text{-}trans\text{-}p}$

Fe	-0.641081	-0.338072	-0.181379
N	-1.132163	1.640718	0.310793
N	0.654104	-0.378753	1.434188
O	-2.188449	-0.904642	1.411457
C	0.429032	1.954519	2.187572
C	-0.533266	2.408041	1.278535
C	-1.899529	3.799055	0.132286
H	-2.461282	4.646650	-0.239461
C	-4.302973	-0.725635	2.428046
C	-2.776338	2.052098	-1.472948
C	0.972213	0.668887	2.265347
C	-1.965938	2.461570	-0.408980
C	-1.020214	3.764585	1.177850
H	-0.722037	4.578999	1.825961
C	-3.289806	-0.344263	1.473206
H	-3.502419	0.474220	0.773488
C	1.365975	-1.472528	1.862055
C	1.951199	0.227102	3.231233
H	2.386620	0.850608	4.001174
C	2.195762	-1.092349	2.980674
H	2.874332	-1.754821	3.501083
C	-5.527785	-0.150302	2.530222
N	-0.472443	-2.379691	-0.373285
N	-2.217340	-0.345520	-1.524871
C	-2.224992	-2.747098	-2.062533
C	-1.230492	-3.188392	-1.185659
C	0.161674	-4.564549	-0.054140
H	0.691991	-5.415823	0.353606
C	1.250204	-2.765067	1.343714
C	-2.668674	-1.432392	-2.233321
C	0.370934	-3.192294	0.345347
C	-0.830372	-4.562567	-0.993587
H	-1.263136	-5.410830	-1.508443
C	-2.903114	0.755398	-1.980426
C	-3.676326	-1.001870	-3.174244
H	-4.203031	-1.654316	-3.858928
C	-3.815901	0.348274	-3.023204
H	-4.481480	1.013785	-3.558110
O	0.678666	0.289895	-1.501775
C	1.183271	2.538245	-2.035594
C	1.449068	1.320809	-1.249296
H	1.722543	1.466268	-0.202281
C	1.458940	3.804293	-1.665481
H	-3.389433	2.817647	-1.939153
H	-2.710838	-3.506269	-2.668183
H	1.861988	-3.530391	1.812091
H	0.795399	2.680200	2.907665
H	-4.027274	-1.539535	3.094536
H	0.659097	2.347699	-2.969864
C	2.985676	-1.575821	-1.595470
C	3.558921	-0.324003	-1.175718
C	3.275245	0.858873	-1.880878
H	3.846463	1.751316	-1.650587
H	3.010709	0.783926	-2.928898
C	2.249640	-1.746327	-2.722959
H	2.135444	-0.926905	-3.426197
C	4.311229	-0.272733	0.090037
C	4.949443	-1.423004	0.600232
C	4.440069	0.928596	0.818357
C	5.689031	-1.370444	1.777917
H	4.905766	-2.357130	0.050949
C	5.166555	0.975653	2.003095
H	3.955628	1.832473	0.468446
C	5.797904	-0.172203	2.487367
H	6.189804	-2.264521	2.137556
H	5.241946	1.911244	2.549499
H	6.374240	-0.132237	3.407051
H	3.059909	-2.418418	-0.915947
C	1.574749	-3.012658	-3.111660

H	1.970783	-3.373723	-4.071193
H	1.684872	-3.796612	-2.359271
H	0.504078	-2.829743	-3.266217
C	1.028304	4.963837	-2.523384
H	1.883319	5.606381	-2.773357
H	0.556685	4.638950	-3.455094
H	0.313696	5.593101	-1.975581
C	2.190821	4.187345	-0.404633
H	1.673376	5.013387	0.097730
H	2.282109	3.370823	0.314619
H	3.199228	4.550323	-0.647682
C	-6.032030	0.978959	1.671204
H	-6.956384	0.671895	1.165385
H	-6.297315	1.833965	2.306359
H	-5.327504	1.323866	0.912915
C	-6.506005	-0.635114	3.563769
H	-6.796987	0.188499	4.229241
H	-7.428949	-0.982350	3.080442
H	-6.103331	-1.449171	4.171561

^{4}TS1E1C$_{endo\text{-}cis\text{-}p}$

C	3.997983	-1.643900	0.158777
C	4.884451	-0.647557	-0.357889
C	4.369542	0.402203	-1.146735
H	5.081996	1.071996	-1.616767
H	3.518021	0.195305	-1.784263
C	2.696644	-1.832555	-0.214852
H	2.297443	-1.331246	-1.092392
C	6.296978	-0.648701	0.078473
C	6.904952	-1.838578	0.530656
C	7.099142	0.510416	0.019236
C	8.241329	-1.863119	0.921076
H	6.342604	-2.766243	0.542211
C	8.430366	0.488537	0.420542
H	6.678652	1.447431	-0.328975
C	9.009327	-0.698718	0.876707
H	8.684774	-2.796817	1.254377
H	9.018893	1.400279	0.376301
H	10.050317	-0.716683	1.185430
H	4.361304	-2.254262	0.981625
C	1.779911	-2.803806	0.442940
H	1.410062	-3.536469	-0.284458
H	2.260773	-3.332428	1.271695
H	0.891991	-2.277377	0.822403
Fe	-1.422585	0.143380	0.036639
N	-0.625168	-0.286458	-1.756979
N	-1.122985	2.097889	-0.307698
O	-3.394993	0.411021	-1.077696
C	-0.118453	1.987220	-2.548936
C	-0.131362	0.606013	-2.685751
C	0.177156	-1.431695	-3.594658
H	0.417857	-2.271748	-4.233375
C	-5.720302	0.111520	-1.295056
C	-0.914787	-2.730406	-1.761227
C	-0.576853	2.674193	-1.433885
C	-0.470925	-1.538798	-2.314777
C	0.382769	-0.103228	-3.826545
H	0.826883	0.367952	-4.693954
C	-4.451480	-0.048171	-0.625863
H	-4.420272	-0.598855	0.323750
C	-1.423080	3.139089	0.543857
C	-0.514697	4.103132	-1.277447
H	-0.127165	4.786006	-2.022439
C	-1.031205	4.390343	-0.049300
H	-1.156791	5.357778	0.419424
C	-6.905249	-0.365240	-0.836077
N	-2.388458	0.588450	1.751546
N	-1.891614	-1.800495	0.294327
C	-3.005299	-1.674922	2.484819
C	-2.931958	-0.299094	2.652935
C	-3.147925	1.727183	3.610346
H	-3.359056	2.564645	4.262605
C	-2.047270	3.021966	1.776591
C	-2.519173	-2.366681	1.383912
C	-2.497923	1.832402	2.330101
C	-3.418981	0.406798	3.809495
H	-3.896906	-0.062643	4.659638
C	-1.583727	-2.844116	-0.551199
C	-2.611615	-3.792108	1.213802
H	-3.062950	-4.468034	1.928596
C	-2.033030	-4.087556	0.014961
H	-1.913387	-5.055337	-0.454731
O	0.275373	-0.118051	1.215667
C	2.049239	1.034558	0.141906
C	1.467966	0.330868	1.199008
H	2.052007	0.162172	2.112755
C	3.341013	1.631929	0.113815
H	-0.736073	-3.641275	-2.323307

H	-3.467935	-2.254057	3.277258
H	-2.210216	3.932193	2.344206
H	0.289646	2.570601	-3.367728
H	-5.682039	0.663402	-2.231272
H	1.432392	1.159697	-0.737433
C	4.136822	1.703347	1.405651
H	4.220413	0.732417	1.899969
H	5.147363	2.078717	1.232857
H	3.638772	2.394669	2.096562
C	3.431742	2.897437	-0.735767
H	2.907897	3.709570	-0.215710
H	4.467711	3.213607	-0.891727
H	2.950685	2.765592	-1.710041
C	-7.094234	-1.138412	0.442242
H	-7.801461	-0.606912	1.091953
H	-7.550943	-2.111303	0.219937
H	-6.178236	-1.313466	1.008462
C	-8.167255	-0.134015	-1.619321
H	-8.633027	-1.093135	-1.882160
H	-8.900852	0.408038	-1.007827
H	-7.992687	0.432186	-2.537605

[6]TS1E1C$_{endo\text{-}cis\text{-}p}$

C	3.851767	-1.646579	0.442901
C	4.728850	-0.795380	-0.305451
C	4.192195	0.175519	-1.176503
H	4.883143	0.708381	-1.820852
H	3.250518	-0.035853	-1.668283
C	2.511476	-1.802554	0.233173
H	2.042645	-1.396382	-0.659145
C	6.178676	-0.845620	-0.029290
C	6.762811	-2.009391	0.513758
C	7.034823	0.234789	-0.332398
C	8.131767	-2.082724	0.756537
H	6.150870	-2.882565	0.713530
C	8.399641	0.166252	-0.076277
H	6.632232	1.148317	-0.756526
C	8.956045	-0.992969	0.471688
H	8.555596	-2.995651	1.164240
H	9.032252	1.018656	-0.305619
H	10.022921	-1.047734	0.666613
H	4.274240	-2.158465	1.303723
C	1.622395	-2.610624	1.111997
H	1.161785	-3.426016	0.540869
H	2.154059	-3.027701	1.972448
H	0.789033	-1.988456	1.466145
Fe	-1.386005	0.342021	0.222584
N	-0.588033	0.748703	-1.647910
N	-1.709649	2.355439	0.509490
O	-3.349690	0.474420	-0.937888
C	-0.683496	3.209937	-1.554489
C	-0.355188	2.000340	-2.172022
C	0.489294	0.524596	-3.664099
H	0.943052	0.045176	-4.522313
C	-5.486513	-0.292674	-1.557658
C	-0.198450	-1.559264	-2.411559
C	-1.313541	3.375215	-0.318701
C	-0.102137	-0.170667	-2.546304
C	0.327417	1.863869	-3.435591
H	0.628183	2.688990	-4.068784
C	-4.244881	-0.372333	-0.825802
H	-4.088054	-1.216226	-0.142491
C	-2.317063	2.922931	1.601102
C	-1.664925	4.640258	0.283930
H	-1.469299	5.610526	-0.154375
C	-2.282226	4.361317	1.469486
H	-2.690514	5.058874	2.189492
C	-6.495398	-1.196286	-1.471365
N	-2.556558	-0.091993	1.875251
N	-1.385861	-1.686604	-0.257073
C	-2.549993	-2.547022	1.737457
C	-2.854420	-1.337497	2.369727
C	-3.640946	0.140810	3.887442
H	-4.092773	0.625243	4.743568
C	-2.906666	2.218329	2.654428
C	-1.883406	-2.708630	0.518635
C	-3.017489	0.831472	2.782240
C	-3.541545	-1.196864	3.632900
H	-3.895412	-2.020008	4.240451
C	-0.815593	-2.257951	-1.370861
C	-1.617953	-3.970327	-0.130198
H	-1.905564	-4.938931	0.258679
C	-0.957515	-3.692716	-1.294526
H	-0.602885	-4.390556	-2.042541
O	0.264639	0.279483	1.337262
C	2.090869	1.233668	0.160562
C	1.496942	0.641773	1.267033
H	2.077262	0.488230	2.182902

C	3.447201	1.654376	0.028271
H	0.219054	-2.155918	-3.217212
H	-2.864697	-3.454565	2.243784
H	-3.338253	2.813644	3.453367
H	-0.430296	4.115936	-2.096763
H	-5.581616	0.568132	-2.215288
H	1.450059	1.368523	-0.700784
C	4.314898	1.720478	1.271263
H	4.278929	0.800366	1.859296
H	5.358859	1.921240	1.023260
H	3.959006	2.541918	1.905444
C	3.658815	2.817728	-0.934470
H	3.293706	3.736855	-0.458228
H	4.716759	2.966129	-1.171319
H	3.100394	2.678675	-1.865404
C	-6.488514	-2.426337	-0.602597
H	-7.337886	-2.390822	0.091744
H	-6.636564	-3.317620	-1.225788
H	-5.577601	-2.566782	-0.018868
C	-7.747400	-1.011322	-2.282631
H	-7.907243	-1.877376	-2.938661
H	-8.623859	-0.957638	-1.623222
H	-7.718557	-0.108670	-2.898014

^{4}TS1E1C$_{endo\text{-}trans\text{-}p}$

C	4.081929	-1.454864	1.069205
C	4.909437	-1.093663	-0.042825
C	4.324783	-0.762359	-1.289282
H	5.006981	-0.546270	-2.105795
H	3.470075	-1.333576	-1.619996
C	2.790340	-1.908052	1.101637
H	2.330833	-1.885617	2.090112
C	6.340581	-0.841943	0.211836
C	6.990145	-1.495142	1.281176
C	7.118978	0.003599	-0.607546
C	8.346143	-1.300134	1.528452
H	6.438329	-2.193338	1.902337
C	8.470086	0.208398	-0.351509
H	6.665112	0.525472	-1.442906
C	9.091457	-0.441202	0.719026
H	8.822543	-1.825689	2.350771
H	9.041705	0.876328	-0.989168
H	10.148298	-0.284907	0.913850
H	4.498735	-1.208564	2.043598
C	1.942693	-2.626619	0.101569
H	0.976867	-2.120324	0.004555
H	2.393257	-2.745981	-0.883244
H	1.725617	-3.628521	0.497171
Fe	-1.489377	0.409847	0.259944
N	-0.916673	1.602898	-1.251238
N	-1.832499	2.027139	1.402059
O	-3.617293	0.697665	-0.531423
C	-0.984364	3.774364	-0.099622
C	-0.728066	2.966124	-1.197510
C	-0.209480	2.375213	-3.307938
H	0.077316	2.347376	-4.351308
C	-5.750709	-0.118112	-1.103823
C	-0.633980	-0.072793	-3.029057
C	-1.518899	3.331176	1.101063
C	-0.609309	1.225812	-2.539956
C	-0.272233	3.451700	-2.473138
H	-0.054600	4.488853	-2.693465
C	-4.396841	-0.258443	-0.621427
H	-4.044007	-1.254395	-0.321066
C	-2.339480	2.042128	2.679651
C	-1.842600	4.187936	2.213531
H	-1.694425	5.259956	2.226719
C	-2.358788	3.390190	3.188707
H	-2.718384	3.670871	4.170153
C	-6.643079	-1.133956	-1.218383
N	-2.236863	-0.776741	1.702910
N	-1.341209	-1.195077	-0.956250
C	-1.998669	-2.969905	0.615700
C	-2.318376	-2.152276	1.691571
C	-3.061934	-1.528377	3.723291
H	-3.448920	-1.484851	4.733136
C	-2.748571	0.929344	3.399246
C	-1.562547	-2.516110	-0.621684
C	-2.683272	-0.379537	2.944361
C	-2.829907	-2.626862	2.950142
H	-2.990796	-3.669043	3.194457
C	-0.959873	-1.196895	-2.282540
C	-1.299835	-3.361123	-1.755173
H	-1.396933	-4.439253	-1.753221
C	-0.931904	-2.544255	-2.784255
H	-0.666745	-2.816853	-3.797737
O	0.377959	0.263902	1.104738
C	2.053601	0.490518	-0.551964

C	1.556688	0.601654	0.747799
H	2.194017	1.034558	1.529385
C	3.327841	0.922376	-1.036599
H	-0.361627	-0.222798	-4.068765
H	-2.122261	-4.040422	0.743420
H	-3.126786	1.092489	4.403030
H	-0.786983	4.836469	-0.200612
H	-6.038017	0.892524	-1.384358
H	1.386942	0.024389	-1.270280
C	4.164250	1.823613	-0.141052
H	4.305989	1.408032	0.859460
H	5.151828	2.006690	-0.570040
H	3.658965	2.791183	-0.037176
C	3.309999	1.380336	-2.496272
H	2.755823	2.324104	-2.562386
H	4.319911	1.550679	-2.882062
H	2.802012	0.656352	-3.141177
C	-8.030554	-0.867273	-1.732002
H	-8.190480	0.185100	-1.979513
H	-8.228257	-1.471771	-2.627219
H	-8.777175	-1.168520	-0.985133
C	-6.372280	-2.572530	-0.864781
H	-6.545199	-3.206444	-1.743982
H	-5.364221	-2.768582	-0.496527
H	-7.086576	-2.904132	-0.100197

^{4}TS1E1C$_{exo-cis-p}$

C	-4.653494	1.646115	-0.409883
C	-5.205550	0.459435	0.174451
C	-4.440205	-0.491343	0.898086
H	-3.593141	-0.136656	1.469832
H	-5.016879	-1.248507	1.423771
C	-3.456563	2.286671	-0.238325
C	-6.607265	0.129168	-0.150654
C	-7.162368	0.409119	-1.417999
C	-7.431742	-0.493348	0.810167
C	-8.477553	0.066235	-1.713973
H	-6.546504	0.852640	-2.193805
C	-8.753056	-0.818684	0.517616
H	-7.047410	-0.680409	1.808016
C	-9.280024	-0.545054	-0.746683
H	-8.876646	0.271201	-2.702928
H	-9.375037	-1.278933	1.279628
H	-10.309436	-0.803795	-0.976351
H	-5.314920	2.144091	-1.113897
Fe	1.571861	0.284713	0.030071
N	0.926427	0.019484	1.919611
N	1.460721	2.272752	0.270847
O	3.672585	0.411996	0.915615
C	0.577037	2.366410	2.563123
C	0.538114	1.000960	2.805207
C	0.243134	-0.942580	3.903925
H	0.028987	-1.719149	4.626948
C	5.960084	-0.144993	0.933130
C	1.053539	-2.433490	2.067297
C	1.016697	2.950510	1.384621
C	0.764709	-1.177481	2.583586
C	0.101890	0.406818	4.040558
H	-0.251066	0.963678	4.898998
C	4.615034	-0.211466	0.412221
H	4.419718	-0.841320	-0.466306
C	1.793381	3.237177	-0.654073
C	1.062248	4.369667	1.149706
H	0.768718	5.121261	1.871227
C	1.542651	4.547262	-0.113264
H	1.723188	5.474436	-0.641645
C	7.025455	-0.810012	0.419086
N	2.363550	0.544473	-1.800220
N	1.835063	-1.711155	-0.150066
C	2.673983	-1.804969	-2.460177
C	2.718430	-0.438314	-2.698670
C	3.068527	1.507577	-3.776134
H	3.310606	2.286221	-4.487835
C	2.304264	2.998800	-1.921416
C	2.264915	-2.391128	-1.269776
C	2.561882	1.742622	-2.449836
C	3.163618	0.157059	-3.931129
H	3.499487	-0.400149	-4.796143
C	1.548754	-2.674933	0.793385
C	2.253835	-3.808025	-1.021045
H	2.548434	-4.560406	-1.741289
C	1.812602	-3.983648	0.257151
H	1.672393	-4.909622	0.799864
O	-0.318485	0.162124	-0.780005
C	-2.462837	-0.802261	-0.911256
C	-1.193499	-0.681336	-0.373501
H	-0.911696	-1.324460	0.465663
C	-3.497908	-1.620391	-0.335354

H	0.874755	-3.291646	2.706793
H	2.985048	-2.464873	-3.263404
H	2.515312	3.859482	-2.547415
H	0.251368	3.026904	3.360004
H	6.086919	0.496263	1.802246
H	-2.704355	-0.176556	-1.765857
C	-2.319737	2.143588	0.709625
H	-2.450541	1.395989	1.491638
H	-1.403743	1.889554	0.155336
H	-2.126829	3.115991	1.181010
H	-3.330632	3.146024	-0.898874
C	-4.551984	-2.109564	-1.319312
H	-5.446955	-2.481657	-0.811266
H	-4.855422	-1.324968	-2.017348
H	-4.126365	-2.933549	-1.905362
C	-3.067858	-2.727564	0.634330
H	-2.431489	-3.444186	0.102825
H	-2.505515	-2.350082	1.492783
H	-3.940033	-3.269257	1.012192
C	6.984016	-1.725945	-0.775509
H	5.998865	-1.834508	-1.231609
H	7.678463	-1.361203	-1.543242
H	7.339375	-2.723472	-0.486805
C	8.383120	-0.662858	1.047701
H	8.760600	-1.641707	1.372316
H	9.104121	-0.282387	0.311957
H	8.375063	0.010521	1.908390

^{6}TS1E1C$_{exo\text{-}cis\text{-}p}$

C	-4.542580	1.766482	-0.402856
C	-5.202000	0.610566	0.139562
C	-4.559906	-0.371586	0.929337
H	-3.716756	-0.073690	1.537040
H	-5.213309	-1.088173	1.420031
C	-3.328020	2.338677	-0.144848
C	-6.593035	0.362978	-0.287802
C	-7.028068	0.655569	-1.598531
C	-7.525036	-0.195735	0.611964
C	-8.332963	0.381473	-1.994683
H	-6.328156	1.052988	-2.326668
C	-8.835954	-0.448900	0.219102
H	-7.231434	-0.388195	1.639147
C	-9.243275	-0.167147	-1.087120
H	-8.640634	0.592427	-3.014521
H	-9.542380	-0.859612	0.934262
H	-10.264423	-0.370629	-1.395677
H	-5.131487	2.307375	-1.138669
Fe	1.514771	0.107051	0.087173
N	1.053963	-0.903221	1.836137
N	1.240213	1.905966	1.062624
O	3.598980	0.196032	0.966458
C	0.467031	1.038486	3.231959
C	0.597024	-0.334200	3.000382
C	0.586412	-2.569396	3.345650
H	0.496398	-3.566115	3.758559
C	5.940779	0.033671	0.801646
C	1.476950	-3.203009	1.065447
C	0.782383	2.072714	2.346878
C	1.069902	-2.265186	2.019986
C	0.293432	-1.378247	3.949679
H	-0.080567	-1.214171	4.952328
C	4.598693	-0.051883	0.279218
H	4.456118	-0.347519	-0.767570
C	1.433824	3.154902	0.526578
C	0.671995	3.484702	2.630062
H	0.339511	3.903250	3.571566
C	1.073492	4.152223	1.507150
H	1.133022	5.222124	1.352935
C	7.066313	-0.220213	0.086817
N	2.254617	1.108939	-1.558793
N	2.087322	-1.692799	-0.781481
C	2.821101	-0.830806	-2.966442
C	2.687157	0.541727	-2.734261
C	2.757277	2.778488	-3.054428
H	2.880496	3.777358	-3.453202
C	1.907202	3.414364	-0.763014
C	2.544580	-1.864480	-2.065465
C	2.282722	2.473634	-1.725545
C	3.005179	1.587983	-3.676770
H	3.370281	1.424737	-4.682760
C	1.944404	-2.941395	-0.225192
C	2.707806	-3.275607	-2.327413
H	3.058040	-3.695457	-3.261795
C	2.337890	-3.939457	-1.192652
H	2.327736	-5.007905	-1.018517
O	-0.335632	-0.010461	-0.645994
C	-2.522621	-0.858735	-0.807002
C	-1.268085	-0.801563	-0.239128

H	-1.030832	-1.435501	0.618788
C	-3.613577	-1.615229	-0.252954
H	1.425145	-4.247072	1.359480
H	3.178197	-1.124712	-3.948896
H	1.996479	4.458940	-1.045883
H	0.105011	1.332542	4.212643
H	6.013279	0.327894	1.845978
H	-2.710165	-0.231485	-1.673876
C	-2.250263	2.097020	0.850514
H	-2.461771	1.330551	1.594791
H	-1.325869	1.809160	0.329108
H	-2.019458	3.037846	1.366341
H	-3.118107	3.206203	-0.772381
C	-4.663868	-2.068928	-1.254048
H	-5.596311	-2.368819	-0.766175
H	-4.894436	-1.292897	-1.988215
H	-4.270958	-2.938177	-1.796566
C	-3.283168	-2.702038	0.772306
H	-2.676244	-3.475525	0.287384
H	-2.723186	-2.326641	1.632982
H	-4.196665	-3.177073	1.141214
C	7.101365	-0.632101	-1.361187
H	6.121972	-0.722145	-1.833193
H	7.692547	0.092952	-1.935234
H	7.617773	-1.595864	-1.456563
C	8.417085	-0.096537	0.734758
H	8.958673	-1.049604	0.670279
H	9.028724	0.642884	0.200750
H	8.352615	0.198000	1.785231

^{4}TS1E1C$_{exo\text{-}trans\text{-}p}$

C	-4.141652	-1.741884	-0.116648
C	-5.062912	-0.706405	-0.454231
C	-4.605344	0.490537	-1.041233
H	-3.739933	0.451464	-1.695007
H	-5.352807	1.214369	-1.352478
C	-2.811239	-1.736317	-0.426416
H	-2.430215	-1.036801	-1.165118
C	-6.460052	-0.799772	0.015355
C	-6.784915	-1.412063	1.242665
C	-7.507710	-0.259352	-0.756144
C	-8.104008	-1.470454	1.683991
H	-5.996958	-1.808067	1.875968
C	-8.827888	-0.331109	-0.319732
H	-7.288052	0.183969	-1.722744
C	-9.130831	-0.933355	0.903727
H	-8.330981	-1.931090	2.641037
H	-9.622159	0.076899	-0.938000
H	-10.160172	-0.985400	1.246141
H	-4.508531	-2.559521	0.499481
C	-1.809573	-2.687164	0.122977
H	-1.012078	-2.109377	0.615184
H	-2.246396	-3.386577	0.841758
H	-1.319559	-3.252058	-0.679303
Fe	1.541773	0.246810	-0.001958
N	1.462249	2.255730	-0.058292
N	0.816504	0.178336	-1.878721
O	3.605822	0.435012	-0.939368
C	0.493686	2.581188	-2.294337
C	0.991631	3.044897	-1.084800
C	1.608360	4.482174	0.534625
H	1.827148	5.351306	1.141351
C	5.890020	-0.125408	-1.050994
C	2.384405	2.754983	2.165457
C	0.418086	1.243614	-2.658241
C	1.847155	3.122261	0.940660
C	1.076644	4.434222	-0.719197
H	0.771106	5.256083	-1.353731
C	4.563485	-0.206938	-0.488985
H	4.394962	-0.865061	0.374092
C	0.607342	-0.949913	-2.642382
C	-0.079936	0.771269	-3.922387
H	-0.453907	1.409533	-4.712555
C	0.039637	-0.587157	-3.913639
H	-0.217651	-1.290986	-4.694673
C	6.970752	-0.805550	-0.590665
N	1.767358	-1.753007	-0.011343
N	2.380265	0.319003	1.822555
C	2.663002	-2.084474	2.255601
C	2.219852	-2.545040	1.023633
C	1.725899	-3.971750	-0.647392
H	1.568110	-4.837260	-1.277998
C	0.913723	-2.249126	-2.264615
C	2.735245	-0.747237	2.620360
C	1.454958	-2.615284	-1.040939
C	2.193092	-3.928928	0.633141
H	2.498605	-4.751892	1.266335
C	2.621365	1.450292	2.571695

C	3.220404	-0.274501	3.889738
H	3.563718	-0.913861	4.692785
C	3.152274	1.086351	3.858420
H	3.427851	1.792706	4.630743
O	-0.349097	0.121044	0.862824
C	-2.621063	0.777066	0.924676
C	-1.328092	0.792405	0.400430
H	-1.136676	1.419843	-0.475552
C	-3.701868	1.566960	0.421259
H	2.632833	3.549000	2.861879
H	2.982428	-2.822838	2.983649
H	0.709945	-3.039927	-2.979237
H	0.152086	3.318243	-3.013568
H	5.989958	0.541611	-1.904024
H	-2.794180	0.135795	1.783699
C	-3.378134	2.805610	-0.414856
H	-2.704772	2.600880	-1.251524
H	-4.291674	3.251829	-0.818293
H	-2.896175	3.554393	0.225180
C	-4.838383	1.802938	1.404446
H	-4.528393	2.563269	2.133005
H	-5.741942	2.165245	0.904978
H	-5.096832	0.894334	1.954601
C	6.966882	-1.755272	0.577862
H	7.311769	-2.744125	0.249420
H	7.686470	-1.412493	1.332397
H	5.997222	-1.876796	1.063020
C	8.307758	-0.639446	-1.257306
H	9.051122	-0.278553	-0.534064
H	8.675271	-1.608612	-1.620083
H	8.272528	0.057374	-2.098369

^{6}TS1E1C$_{exo-trans-p}$

C	-4.099351	-1.763255	-0.073051
C	-5.020278	-0.735341	-0.449646
C	-4.568222	0.437541	-1.081010
H	-3.683833	0.388065	-1.707483
H	-5.314899	1.144880	-1.428431
C	-2.771364	-1.779418	-0.382022
H	-2.378223	-1.091457	-1.125604
C	-6.419367	-0.818514	0.013905
C	-6.749247	-1.397186	1.256513
C	-7.464026	-0.300695	-0.777647
C	-8.070075	-1.443171	1.693613
H	-5.964563	-1.775508	1.904393
C	-8.785879	-0.361730	-0.345436
H	-7.241230	0.114102	-1.756033
C	-9.093529	-0.929065	0.893559
H	-8.301234	-1.876743	2.662143
H	-9.577823	0.027051	-0.978768
H	-10.124359	-0.972392	1.232593
H	-4.473847	-2.560496	0.564592
C	-1.779768	-2.723703	0.195595
H	-0.981988	-2.140086	0.679497
H	-2.225371	-3.404259	0.926777
H	-1.286261	-3.307736	-0.590464
Fe	1.485367	0.246040	0.057177
N	1.481108	2.313280	0.009007
N	0.783274	0.223918	-1.894492
O	3.510918	0.444585	-0.908410
C	0.525240	2.650188	-2.232364
C	1.024344	3.111792	-1.010405
C	1.672823	4.512833	0.640758
H	1.907498	5.372923	1.254738
C	5.786348	-0.117772	-1.113945
C	2.434988	2.712771	2.242357
C	0.422182	1.317554	-2.645496
C	1.890226	3.138259	1.027426
C	1.137612	4.496579	-0.615813
H	0.850403	5.340901	-1.229442
C	4.481859	-0.212494	-0.507539
H	4.340343	-0.892010	0.342154
C	0.575008	-0.892738	-2.667546
C	-0.065208	0.871480	-3.928996
H	-0.415966	1.523462	-4.718913
C	0.030334	-0.491934	-3.943208
H	-0.228064	-1.169847	-4.746773
C	6.878845	-0.815948	-0.710879
N	1.751606	-1.804979	-0.020855
N	2.422789	0.273006	1.891866
C	2.693425	-2.149022	2.228798
C	2.223810	-2.606325	0.993569
C	1.693002	-3.995785	-0.709420
H	1.526555	-4.851747	-1.351152
C	0.870969	-2.205080	-2.287478
C	2.784931	-0.817492	2.646321
C	1.419131	-2.626540	-1.073182
C	2.184263	-3.983882	0.566108

H	2.498315	-4.827812	1.167068
C	2.675781	1.396484	2.644283
C	3.293274	-0.366034	3.919575
H	3.650785	-1.015067	4.708795
C	3.226773	0.998492	3.917640
H	3.519617	1.681079	4.705113
O	-0.355636	0.164546	0.873275
C	-2.622460	0.834158	0.916749
C	-1.335225	0.840970	0.396264
H	-1.131474	1.457585	-0.482325
C	-3.704348	1.598179	0.377222
H	2.700589	3.490409	2.952114
H	3.026596	-2.904945	2.933475
H	0.659382	-2.980216	-3.018088
H	0.202606	3.405473	-2.942693
H	5.859298	0.572490	-1.950959
H	-2.803648	0.203150	1.781589
C	-3.389690	2.805400	-0.503691
H	-2.703873	2.579335	-1.324217
H	-4.304825	3.223636	-0.931981
H	-2.925135	3.583751	0.113830
C	-4.863162	1.844616	1.328135
H	-4.580632	2.639674	2.030647
H	-5.764307	2.169563	0.799666
H	-5.112623	0.953761	1.910312
C	6.910044	-1.797969	0.430062
H	7.240896	-2.777886	0.062721
H	7.655396	-1.477859	1.169389
H	5.956721	-1.931015	0.943603
C	8.192566	-0.637198	-1.418828
H	8.962454	-0.300544	-0.711836
H	8.541664	-1.597566	-1.821003
H	8.132350	0.083194	-2.238348

^{4}TS2E1C$_{exo\text{-}trans\text{-}p}$

C	3.377027	-1.564276	0.060296
C	4.236704	-0.443988	0.296101
C	3.739962	0.732146	0.886656
H	2.910755	0.660856	1.582567
H	4.452377	1.514404	1.130091
C	2.080590	-1.656970	0.469736
H	1.686449	-0.941262	1.186193
C	5.599202	-0.453608	-0.270343
C	5.880239	-1.090240	-1.496784
C	6.654954	0.195683	0.400682
C	7.163304	-1.064410	-2.035704
H	5.082421	-1.571428	-2.054033
C	7.940860	0.205807	-0.132056
H	6.473819	0.657971	1.366233
C	8.198784	-0.419696	-1.354419
H	7.355383	-1.544486	-2.990688
H	8.744358	0.695746	0.409858
H	9.201067	-0.407325	-1.772306
H	3.769641	-2.375844	-0.547438
C	1.127666	-2.711439	0.035240
H	0.265178	-2.222959	-0.443449
H	1.576851	-3.419737	-0.666824
H	0.724238	-3.260541	0.894348
Fe	-2.329735	-0.033865	0.177043
N	-2.723082	1.921078	-0.059462
N	-1.643541	0.364792	2.026721
C	-1.697128	2.824912	1.986067
C	-2.334683	2.961938	0.764546
C	-3.270680	3.952271	-1.025052
H	-3.667412	4.646061	-1.754670
C	-3.822121	1.858791	-2.258735
C	-1.380834	1.609772	2.572110
C	-3.295208	2.523357	-1.164920
C	-2.675285	4.224142	0.169960
H	-2.483398	5.187287	0.624725
C	-1.175760	-0.548307	2.955880
C	-0.728732	1.470670	3.843117
H	-0.428572	2.298982	4.471809
C	-0.600907	0.133594	4.080349
H	-0.175093	-0.360099	4.944275
N	-2.372585	-1.984642	0.646759
N	-3.404832	-0.430119	-1.461498
C	-3.388381	-2.887688	-1.403600
C	-2.798372	-3.025099	-0.159003
C	-1.972860	-4.014513	1.684660
H	-1.651452	-4.708117	2.450870
C	-1.274530	-1.925023	2.847718
C	-3.673775	-1.673023	-2.002221
C	-1.856301	-2.586288	1.779781
C	-2.551479	-4.286390	0.480994
H	-2.804549	-5.248891	0.055964
C	-3.873146	0.481820	-2.388370
C	-4.318138	-1.534860	-3.278537

H	-4.635945	-2.363954	-3.897125
C	-4.442457	-0.199659	-3.517349
H	-4.882897	0.295083	-4.373076
O	-0.556768	0.000906	-0.751234
C	1.634228	0.852934	-0.984373
C	0.382718	0.796811	-0.387430
H	0.169622	1.466153	0.450739
C	2.684922	1.730808	-0.570475
H	-4.235047	2.457746	-3.063320
H	-3.663004	-3.791842	-1.936191
H	-0.888909	-2.525346	3.665001
H	-1.442342	3.729447	2.528099
H	1.815723	0.173508	-1.811697
C	2.336152	2.962870	0.260705
H	1.717993	2.738323	1.134014
H	3.241484	3.468137	0.608222
H	1.782593	3.670207	-0.368959
C	3.754555	2.001738	-1.614010
H	3.362711	2.724902	-2.341388
H	4.659506	2.428696	-1.172156
H	4.035436	1.096277	-2.157863

^{6}TS2E1C$_{exo\text{-}trans\text{-}p}$

C	3.396190	-1.554604	0.057930
C	4.280323	-0.444058	0.281037
C	3.828764	0.728867	0.898550
H	2.986907	0.683652	1.580481
H	4.555317	1.499881	1.132774
C	2.111173	-1.638169	0.495146
H	1.728546	-0.904826	1.200730
C	5.630321	-0.477616	-0.314003
C	5.870559	-1.112257	-1.550198
C	6.713147	0.147716	0.336593
C	7.141016	-1.106443	-2.118878
H	5.051651	-1.576801	-2.090786
C	7.986197	0.136892	-0.225815
H	6.563687	0.606333	1.309293
C	8.203710	-0.485417	-1.457755
H	7.302153	-1.584559	-3.080486
H	8.811360	0.607437	0.300405
H	9.196236	-0.489561	-1.898403
H	3.771519	-2.371867	-0.552814
C	1.146253	-2.694604	0.093174
H	0.296764	-2.214072	-0.414327
H	1.590457	-3.435120	-0.578201
H	0.726850	-3.202169	0.969747
Fe	-2.210467	-0.037741	0.092031
N	-3.186640	1.599232	-0.725588
N	-1.781935	1.155701	1.744742
C	-2.404416	3.349656	0.815452
C	-3.051922	2.916163	-0.337658
C	-4.177356	2.973645	-2.291838
H	-4.716617	3.263522	-3.184314
C	-4.233720	0.486946	-2.653923
C	-1.812386	2.536392	1.779387
C	-3.873045	1.614718	-1.921792
C	-3.671731	3.777375	-1.312669
H	-3.714944	4.856639	-1.243233
C	-1.099477	0.751376	2.875501
C	-1.124723	3.007361	2.953262
H	-1.014367	4.048714	3.227451
C	-0.680087	1.904937	3.627739
H	-0.137249	1.867776	4.563613
N	-1.980034	-1.685063	1.346778
N	-3.343892	-1.241941	-1.145462
C	-2.900460	-3.433888	-0.119771
C	-2.258124	-3.000290	1.037110
C	-1.187929	-3.058300	3.023249
H	-0.722085	-3.349126	3.956074
C	-0.879179	-0.573033	3.246626
C	-3.406698	-2.620633	-1.129461
C	-1.324787	-1.700735	2.561086
C	-1.770696	-3.860726	2.084197
H	-1.872128	-4.938324	2.094364
C	-4.005017	-0.836618	-2.287008
C	-4.104219	-3.089973	-2.298667
H	-4.295233	-4.130700	-2.526290
C	-4.470540	-1.988251	-3.015456
H	-5.021961	-1.947512	-3.945834
O	-0.474297	0.094867	-0.777246
C	1.720905	0.937291	-1.012772
C	0.482580	0.879928	-0.402799
H	0.275857	1.526599	0.452689
C	2.776510	1.801494	-0.590966
H	-4.774578	0.653910	-3.580079
H	-3.038041	-4.504458	-0.234714
H	-0.354040	-0.741124	4.181911
H	-2.362940	4.421317	0.983589

H	1.896901	0.263923	-1.846271
C	2.456869	3.014033	0.274082
H	1.827152	2.785650	1.137919
H	3.372679	3.487843	0.637070
H	1.925718	3.751240	-0.341230
C	3.866846	2.057407	-1.611824
H	3.502116	2.802225	-2.332064
H	4.774957	2.456545	-1.151501
H	4.132259	1.154342	-2.166926

^{4}TS1E1C$_{endo\text{-}cis\text{-}m}$

C	-3.925904	0.751225	-0.956665
C	-4.882824	-0.256288	-1.166853
C	-4.580548	-1.551419	-1.621705
C	-2.601679	0.481065	-1.287830
H	-1.809672	1.173912	-1.033562
Fe	1.216832	-0.310934	0.484131
N	1.111452	-2.302366	0.303289
N	1.782350	-0.509813	2.394527
O	3.390501	-0.462248	-0.077705
C	1.573747	-2.945516	2.631583
C	1.251807	-3.230811	1.313577
C	0.855809	-4.432869	-0.549235
H	0.692286	-5.213107	-1.281336
C	5.291634	0.264922	-1.259844
C	0.652723	-2.488482	-2.106639
C	1.842643	-1.676112	3.121369
C	0.879638	-3.026195	-0.847641
C	1.073916	-4.558757	0.790947
H	1.132457	-5.463804	1.381506
C	3.899914	0.323592	-0.888773
H	3.262921	1.094988	-1.341205
C	2.122333	0.499440	3.265689
C	2.240088	-1.395017	4.476271
H	2.364057	-2.143739	5.247798
C	2.422147	-0.047966	4.563331
H	2.722327	0.537802	5.422390
C	5.894739	1.104602	-2.140443
N	1.413866	1.687718	0.622979
N	0.773774	-0.112883	-1.471858
C	0.726637	2.342934	-1.645252
C	1.110341	2.625129	-0.341094
C	1.717932	3.812115	1.473116
H	1.961491	4.585981	2.189456
C	2.136737	1.853683	2.967723
C	0.597897	1.065097	-2.172669
C	1.781021	2.400775	1.743818
C	1.293786	3.951681	0.185018
H	1.120787	4.862923	-0.372784
C	0.589447	-1.130693	-2.387679
C	0.277706	0.778502	-3.543511
H	0.085242	1.529337	-4.298998
C	0.280702	-0.580124	-3.678579
H	0.093538	-1.167681	-4.568220
O	-0.760269	-0.046025	1.212358
C	-2.209045	-1.495358	-0.018674
C	-1.894085	-0.533038	0.979859
H	-2.711333	-0.180497	1.623489
C	-3.384610	-2.305897	-0.031116
H	0.486664	-3.179850	-2.926230
H	0.534435	3.181563	-2.306277
H	2.415363	2.538902	3.761373
H	1.655259	-3.778954	3.321265
H	5.866677	-0.521621	-0.777096
H	-1.376503	-1.813656	-0.632904
C	-4.349896	-2.236828	1.142224
H	-4.636310	-1.211570	1.389414
H	-5.263797	-2.796655	0.924594
H	-3.894627	-2.687015	2.034281
C	-3.124646	-3.719902	-0.539805
H	-2.476855	-4.248416	0.171417
H	-4.044892	-4.300709	-0.632780
H	-2.610325	-3.716192	-1.507159
C	7.357241	0.949994	-2.449991
H	7.816777	0.123713	-1.902110
H	7.502676	0.781646	-3.525355
H	7.896915	1.874915	-2.206772
C	5.214405	2.229706	-2.874034
H	5.322333	2.080715	-3.956051
H	4.152754	2.344372	-2.650245
H	5.717390	3.177107	-2.641712
H	-2.348382	-0.214873	-2.076552
C	-4.281264	1.986334	-0.202169
C	-5.541651	2.581442	-0.374876
C	-3.374500	2.589672	0.688174
C	-5.890598	3.735656	0.325125
H	-6.246244	2.153718	-1.082925
C	-3.726882	3.741835	1.389400

H	-2.393145	2.154596	0.855717
C	-4.985707	4.319507	1.213201
H	-6.868497	4.182647	0.170000
H	-3.015179	4.185242	2.080521
H	-5.257804	5.217741	1.759888
H	-5.879032	-0.097395	-0.758113
H	-3.746861	-1.661092	-2.312428
C	-5.726575	-2.502553	-1.889427
H	-5.391653	-3.532342	-2.029569
H	-6.467079	-2.484391	-1.082759
H	-6.241187	-2.206899	-2.812693

^{4}TS1E1C$_{endo\text{-}trans\text{-}m}$

C	-4.030665	0.617993	-0.973880
C	-4.778506	-0.506610	-1.359424
C	-4.263548	-1.727040	-1.855926
H	-5.038833	-2.489824	-1.931934
C	-2.648988	0.621468	-1.132769
H	-2.043108	1.374470	-0.640972
Fe	1.206008	-0.294502	0.534596
N	1.224639	-2.272414	0.208607
N	1.797335	-0.597631	2.423483
O	3.395455	-0.301533	-0.046486
C	1.710040	-3.051350	2.489324
C	1.415287	-3.260672	1.150423
C	1.106891	-4.346944	-0.798080
H	0.997088	-5.080537	-1.586354
C	5.253756	0.604678	-1.171502
C	0.780632	-2.309705	-2.210546
C	1.909320	-1.807020	3.068708
C	1.043461	-2.922951	-0.993515
C	1.323925	-4.556067	0.531572
H	1.434594	-5.496213	1.056167
C	3.858768	0.558465	-0.807053
H	3.177727	1.315511	-1.218732
C	2.097671	0.363485	3.360569
C	2.293525	-1.601760	4.441414
H	2.449792	-2.395221	5.160597
C	2.420215	-0.257520	4.619474
H	2.697140	0.280791	5.516546
C	5.811454	1.531945	-1.991428
N	1.328492	1.697359	0.805683
N	0.756704	0.017525	-1.406609
C	0.582650	2.473039	-1.404973
C	0.976680	2.683542	-0.090247
C	1.567080	3.770887	1.791039
H	1.792050	4.504174	2.554582
C	2.063608	1.734113	3.151721
C	0.507495	1.230013	-2.019252
C	1.682440	2.347500	1.967336
C	1.121051	3.979024	0.520040
H	0.909670	4.918383	0.025581
C	0.629035	-0.941339	-2.392321
C	0.192380	1.023877	-3.406017
H	-0.048950	1.813918	-4.105473
C	0.277388	-0.319090	-3.639414
H	0.118441	-0.851621	-4.568286
O	-0.761312	-0.188510	1.246967
C	-2.123133	-1.646874	-0.054179
C	-1.884010	-0.687467	0.962138
H	-2.723248	-0.382075	1.601469
C	-3.289764	-2.451017	-0.214107
H	0.656777	-2.948156	-3.079131
H	0.347289	3.345535	-2.005773
H	2.321255	2.375473	3.987999
H	1.833491	-3.925553	3.119825
H	5.871351	-0.175785	-0.733403
H	-1.257103	-1.899838	-0.650687
C	-4.355528	-2.423075	0.874847
H	-4.620136	-1.409262	1.181811
H	-5.268340	-2.924456	0.537519
H	-3.990926	-2.957825	1.761107
C	-3.002101	-3.866541	-0.715464
H	-2.597319	-4.461367	0.112901
H	-3.910314	-4.365836	-1.067514
H	-2.259055	-3.877560	-1.517311
C	7.282008	1.482191	-2.298048
H	7.786811	0.652594	-1.796860
H	7.441631	1.387095	-3.380358
H	7.765757	2.420599	-1.995958
C	5.070419	2.662370	-2.654658
H	5.202437	2.601305	-3.742468
H	4.000826	2.692121	-2.441799
H	5.507777	3.620058	-2.344322
C	-3.263204	-1.827000	-3.008780
H	-3.445243	-1.028818	-3.736593
H	-2.216114	-1.759063	-2.706093
H	-3.388604	-2.783300	-3.523538

H	-2.146034	0.060689	-1.902879
C	-4.665638	1.708390	-0.179224
C	-5.673285	1.451181	0.765662
C	-4.241494	3.036238	-0.354961
C	-6.236139	2.486657	1.510259
H	-6.010498	0.433118	0.937632
C	-4.803201	4.072209	0.389421
H	-3.481932	3.258644	-1.099757
C	-5.802863	3.800930	1.325409
H	-7.008919	2.264987	2.240805
H	-4.467599	5.093253	0.231005
H	-6.243011	4.607469	1.904560
H	-5.816821	-0.525256	-1.036928

^{4}TS1E1C$_{exo\text{-}cis\text{-}m}$

C	-4.201160	-0.592684	-0.637395
C	-4.986557	0.515129	-1.001736
C	-4.503940	1.804682	-1.327902
H	-5.311262	2.529358	-1.435439
C	-2.816417	-0.493826	-0.664043
H	-2.293381	0.130630	-1.366945
Fe	1.403921	0.486169	0.266796
N	2.272027	1.918632	1.359308
N	1.106029	1.800878	-1.224522
O	3.420854	0.068528	-0.642109
C	1.974337	3.835502	-0.150975
C	2.401837	3.250115	1.030869
C	3.340507	3.047148	3.066920
H	3.834055	3.203332	4.017300
C	5.117246	-1.397685	-1.356713
C	2.916594	0.599221	3.330702
C	1.378935	3.152693	-1.201859
C	2.841437	1.778443	2.605219
C	3.062860	3.959657	2.094651
H	3.284547	5.018867	2.080877
C	3.824693	-1.092403	-0.795077
H	3.170501	-1.919486	-0.488473
C	0.543856	1.546629	-2.458211
C	0.964079	3.759878	-2.437561
H	1.068850	4.811575	-2.670670
C	0.451709	2.764622	-3.217405
H	0.048186	2.834451	-4.219297
C	5.603130	-2.650810	-1.549976
N	0.627355	-0.985540	-0.881384
N	1.782661	-0.858816	1.707905
C	0.756034	-2.858902	0.707695
C	0.403598	-2.295872	-0.511715
C	-0.388842	-2.123200	-2.613533
H	-0.827306	-2.293845	-3.588276
C	0.106031	0.308875	-2.907309
C	1.405960	-2.185351	1.731767
C	0.141831	-0.864018	-2.166331
C	-0.230972	-3.009150	-1.586770
H	-0.509247	-4.054646	-1.550818
C	2.407654	-0.621280	2.913127
C	1.811220	-2.791390	2.972060
H	1.632624	-3.825464	3.237177
C	2.437309	-1.824649	3.700559
H	2.875991	-1.902056	4.686877
O	-0.498013	0.846053	1.083536
C	-2.753959	1.507892	0.888163
C	-1.374750	1.607552	0.594798
H	-1.042103	2.376348	-0.113953
C	-3.756553	2.443872	0.467043
H	3.389252	0.638785	4.306406
H	0.516184	-3.905064	0.866645
H	-0.313989	0.258296	-3.906540
H	2.135967	4.901501	-0.271485
H	5.716440	-0.533330	-1.632959
H	-3.017351	0.760788	1.630501
C	-3.330499	3.856262	0.066681
H	-2.471396	3.887861	-0.605980
H	-4.155534	4.390649	-0.414532
H	-3.063037	4.414373	0.972507
C	-4.999906	2.455531	1.350675
H	-4.776109	2.979149	2.289157
H	-5.834203	2.978122	0.871695
H	-5.323105	1.442506	1.606028
C	4.876045	-3.925645	-1.214014
H	4.774854	-4.539851	-2.118002
H	5.473856	-4.514534	-0.506612
H	3.882940	-3.783768	-0.785415
C	6.969825	-2.847232	-2.143571
H	7.605130	-3.418663	-1.453851
H	6.902998	-3.440738	-3.065044
H	7.467017	-1.901578	-2.373275
C	-4.823544	-1.783115	0.006441
C	-4.287286	-3.059207	-0.236406

C	-5.929201	-1.676310	0.866444
C	-4.838273	-4.191979	0.360126
H	-3.445637	-3.160240	-0.916984
C	-6.481596	-2.809182	1.462072
H	-6.350060	-0.700301	1.091665
C	-5.938548	-4.070881	1.211083
H	-4.416361	-5.171336	0.151640
H	-7.332277	-2.705273	2.129589
H	-6.371305	-4.953003	1.674042
C	-3.387786	2.052044	-2.341086
H	-2.380502	1.987569	-1.923709
H	-3.447506	1.323464	-3.157332
H	-3.491836	3.050392	-2.773638
H	-6.050123	0.442239	-0.786802
H	-2.198571	-1.206042	-0.128383

^{4}TS1E1C$_{exo\text{-}trans\text{-}m}$

C	-4.098950	-0.824635	-0.599184
C	-5.198516	0.045434	-0.651320
C	-5.081119	1.438116	-0.797577
C	-2.824893	-0.301378	-0.814385
H	-2.660552	0.526689	-1.495240
Fe	1.260318	0.515067	0.062469
N	1.599168	2.470837	0.332745
N	0.430237	0.910290	-1.724381
O	3.243067	0.384975	-0.982148
C	0.536238	3.368105	-1.694674
C	1.210722	3.506231	-0.489474
C	2.285184	4.483310	1.230870
H	2.747401	5.171513	1.926557
C	5.313717	-0.664317	-1.370050
C	2.805679	2.378748	2.473682
C	0.195624	2.154227	-2.274373
C	2.259903	3.053936	1.392108
C	1.627382	4.764196	0.070686
H	1.443551	5.729477	-0.382941
C	4.025734	-0.543539	-0.734820
H	3.722346	-1.302886	-0.001937
C	-0.007808	-0.006365	-2.657975
C	-0.430537	2.013791	-3.560960
H	-0.720085	2.840548	-4.196707
C	-0.545692	0.676020	-3.803339
H	-0.956105	0.182669	-4.674888
C	6.206436	-1.659049	-1.129127
N	0.981856	-1.454866	-0.249129
N	2.166041	0.100864	1.803746
C	1.823158	-2.332734	1.889134
C	1.234150	-2.478873	0.641227
C	0.380661	-3.476422	-1.188759
H	0.006528	-4.173352	-1.927715
C	0.017983	-1.385515	-2.510828
C	2.267862	-1.128742	2.417877
C	0.456696	-2.052406	-1.376222
C	0.851011	-3.739023	0.064182
H	0.941637	-4.695370	0.562670
C	2.740713	1.006992	2.668610
C	2.934343	-0.992831	3.685408
H	3.138269	-1.811918	4.362853
C	3.234576	0.328341	3.836755
H	3.733031	0.815203	4.664897
O	-0.585001	0.624826	1.089153
C	-2.890380	1.113958	1.057588
C	-1.530778	1.367025	0.727731
H	-1.295276	2.237137	0.100926
C	-3.960595	2.053262	0.917757
H	3.294701	2.971362	3.239656
H	1.963997	-3.227141	2.486894
H	-0.366938	-1.984947	-3.329214
H	0.292537	4.272966	-2.241447
H	5.551077	0.117877	-2.087145
H	-3.040923	0.254659	1.705073
C	-3.618216	3.476046	0.493265
H	-2.990956	3.516267	-0.403026
H	-4.521136	4.057750	0.293952
H	-3.080490	3.988816	1.300875
C	-5.026790	1.968062	1.998803
H	-4.642637	2.395501	2.935067
H	-5.925809	2.529773	1.730251
H	-5.310498	0.929705	2.196874
C	6.002408	-2.794969	-0.162284
H	6.087435	-3.750893	-0.694749
H	6.804406	-2.787103	0.586914
H	5.046173	-2.781301	0.362803
C	7.523120	-1.682482	-1.853236
H	8.352916	-1.663266	-1.134295
H	7.628111	-2.616877	-2.420527
H	7.637947	-0.841563	-2.541501
C	-4.228191	-2.209166	-0.074223

C	-3.372507	-3.221794	-0.542290
C	-5.169959	-2.538761	0.915378
C	-3.453675	-4.517827	-0.038086
H	-2.647993	-2.991591	-1.318386
C	-5.256458	-3.837310	1.415159
H	-5.822057	-1.768758	1.318160
C	-4.398468	-4.831802	0.941069
H	-2.784370	-5.285689	-0.416565
H	-5.987613	-4.070014	2.184199
H	-4.466191	-5.842947	1.332093
H	-6.177979	-0.352157	-0.391616
H	-1.945217	-0.887385	-0.580591
C	-6.344408	2.260389	-0.912748
H	-6.188447	3.311305	-0.654649
H	-6.709480	2.238797	-1.947714
H	-7.141350	1.866491	-0.273562
H	-4.256046	1.803128	-1.405543

^{4}TS2E1C$_{endo\text{-}cis\text{-}m}$

C	3.502703	0.026411	0.151795
C	4.128475	-1.209082	-0.086380
C	3.544994	-2.466970	0.175645
C	2.270640	0.032663	0.793499
H	1.692404	0.943349	0.892474
Fe	-1.867476	0.205141	0.375131
N	-2.273251	-1.753783	0.226078
N	-3.222753	0.565238	-1.053439
C	-3.621704	-1.732164	-1.832172
C	-2.945191	-2.377495	-0.811340
C	-2.217560	-4.051603	0.504128
H	-1.995479	-5.005931	0.963819
C	-1.059852	-2.619376	2.184115
C	-3.772233	-0.359150	-1.921070
C	-1.825848	-2.780113	1.041783
C	-2.898689	-3.802835	-0.650695
H	-3.355368	-4.511169	-1.329583
C	-3.650437	1.796714	-1.510935
C	-4.558765	0.302199	-2.924959
H	-5.097123	-0.205631	-3.714468
C	-4.491491	1.637405	-2.664698
H	-4.959995	2.453764	-3.198621
N	-1.851189	2.158738	0.820231
N	-0.894965	-0.163981	2.091379
C	-0.302992	2.138418	2.730267
C	-1.069739	2.782534	1.774467
C	-2.055398	4.456968	0.642241
H	-2.401629	5.410837	0.266350
C	-3.303824	3.020734	-0.966414
C	-0.252872	0.764321	2.894091
C	-2.453766	3.181999	0.113994
C	-1.190128	4.209861	1.664948
H	-0.681715	4.918678	2.305409
C	-0.617733	-1.396154	2.660970
C	0.454153	0.103642	3.952621
H	1.034218	0.611363	4.712149
C	0.219672	-1.233833	3.814722
H	0.567245	-2.046474	4.439501
O	-0.321040	0.387148	-0.901295
C	1.035674	-1.564738	-0.655549
C	0.698432	-0.309953	-1.199406
H	1.333053	0.104587	-1.990990
C	1.997155	-2.483654	-1.186166
H	-0.774868	-3.510461	2.733423
H	0.258210	2.751775	3.427042
H	-3.711045	3.913795	-1.428082
H	-4.107073	-2.344774	-2.584321
H	0.370031	-1.934875	0.114433
C	2.663943	-2.159989	-2.515318
H	3.064387	-1.143882	-2.550355
H	3.486399	-2.853115	-2.713846
H	1.938703	-2.268463	-3.331924
C	1.542298	-3.937672	-1.072622
H	0.666657	-4.089062	-1.715660
H	2.314989	-4.633860	-1.405170
H	1.246633	-4.195517	-0.050001
H	1.975943	-0.760754	1.466543
C	4.053646	1.273233	-0.452018
C	5.441395	1.477945	-0.509978
C	3.207008	2.266638	-0.973746
C	5.968764	2.636202	-1.079326
H	6.113985	0.739290	-0.082207
C	3.736321	3.422132	-1.546558
H	2.128410	2.137864	-0.948249
C	5.118444	3.611343	-1.603456
H	7.045141	2.779212	-1.106422
H	3.066021	4.173812	-1.953944
H	5.529178	4.512623	-2.048859
H	5.022505	-1.205356	-0.707182

H	2.874109	-2.536186	1.030113
C	4.409620	-3.697206	-0.014958
H	3.849203	-4.623447	0.123914
H	4.877466	-3.718005	-1.005248
H	5.216508	-3.694568	0.728028

^{4}TS2E1C$_{endo\text{-}trans\text{-}m}$

C	3.539999	-0.371753	0.058113
C	3.959412	-1.680057	-0.236560
C	3.244004	-2.877029	0.014113
H	3.743611	-3.738589	-0.429475
C	2.333448	-0.159644	0.714272
H	1.893980	0.831542	0.747005
Fe	-1.792414	0.376004	0.351010
N	-2.515855	-1.470009	0.046438
N	-3.069322	1.072950	-1.026012
C	-3.840023	-1.054932	-1.985188
C	-3.278230	-1.885906	-1.031204
C	-2.837462	-3.761257	0.131022
H	-2.776087	-4.774141	0.507421
C	-1.478025	-2.680767	1.920217
C	-3.762781	0.326955	-1.958880
C	-2.245776	-2.619808	0.769251
C	-3.465974	-3.308411	-0.990782
H	-4.030399	-3.872670	-1.721681
C	-3.281002	2.391172	-1.379503
C	-4.425645	1.189689	-2.897461
H	-5.039764	0.844905	-3.719063
C	-4.134799	2.469188	-2.532534
H	-4.457368	3.393084	-2.994455
N	-1.451465	2.258193	0.953489
N	-0.911956	-0.286390	2.031383
C	0.048259	1.826172	2.852977
C	-0.586513	2.663897	1.951824
C	-1.264940	4.564745	0.960071
H	-1.442281	5.590000	0.662737
C	-2.730748	3.491521	-0.745616
C	-0.135883	0.454989	2.906745
C	-1.868385	3.420773	0.334713
C	-0.463415	4.095346	1.956313
H	0.149713	4.655725	2.650016
C	-0.850663	-1.589158	2.498879
C	0.436498	-0.396804	3.909118
H	1.080732	-0.055376	4.708924
C	-0.014639	-1.661145	3.663051
H	0.185783	-2.568272	4.218365
O	-0.253591	0.388020	-0.928648
C	0.838116	-1.726274	-0.775400
C	0.687030	-0.407892	-1.248122
H	1.380236	-0.030621	-2.008711
C	1.717247	-2.730512	-1.288896
H	-1.348385	-3.648686	2.392792
H	0.693860	2.279298	3.597822
H	-2.978357	4.474391	-1.131867
H	-4.418458	-1.514568	-2.779403
H	0.121356	-2.026362	-0.021906
C	2.398940	-2.485359	-2.630469
H	2.853274	-1.495206	-2.700781
H	3.179525	-3.230695	-2.812004
H	1.660888	-2.574034	-3.437273
C	1.155513	-4.148669	-1.172184
H	0.378186	-4.285144	-1.934074
H	1.926067	-4.905827	-1.346460
H	0.694820	-4.331000	-0.197698
C	2.624469	-3.226687	1.368589
H	3.239588	-2.822790	2.179569
H	1.608726	-2.852029	1.511207
H	2.587296	-4.312746	1.486568
H	1.890844	-0.880680	1.381934
C	4.257740	0.797398	-0.524969
C	4.817267	0.758535	-1.813213
C	4.369948	1.984629	0.217521
C	5.466487	1.873010	-2.342119
H	4.731561	-0.139839	-2.418035
C	5.019915	3.098793	-0.310467
H	3.967220	2.022921	1.226217
C	5.569953	3.046545	-1.592697
H	5.886425	1.826366	-3.342735
H	5.106713	4.004150	0.283412
H	6.078775	3.913250	-2.004253
H	4.807543	-1.767588	-0.911433

^{4}TS2E1C$_{exo\text{-}cis\text{-}m}$

C	-3.632744	-0.594691	0.081684
C	-3.956831	-1.936797	-0.194506
C	-3.064448	-3.039092	-0.183400
C	-2.353358	-0.261786	0.496141
H	-1.716552	-0.938930	1.037315

Fe	1.938951	0.490780	0.364169
N	3.541008	0.303596	-0.819783
N	2.292428	-1.346482	1.092526
C	4.070505	-2.069703	-0.446772
C	4.256574	-0.851781	-1.076329
C	5.121241	0.689488	-2.467201
H	5.692571	1.236298	-3.205817
C	3.629846	2.564005	-1.783764
C	3.158148	-2.292782	0.570696
C	4.062184	1.253942	-1.678035
C	5.239028	-0.615890	-2.097350
H	5.928321	-1.363182	-2.468156
C	1.594350	-2.000147	2.094649
C	2.991382	-3.547333	1.246482
H	3.559078	-4.442057	1.026496
C	2.024616	-3.365511	2.191831
H	1.637084	-4.080819	2.905638
N	0.650795	0.808476	1.878013
N	1.877181	2.443973	-0.063509
C	0.046527	3.148954	1.421007
C	-0.086217	1.955387	2.110688
C	-0.853803	0.452806	3.599717
H	-1.373084	-0.069975	4.392411
C	0.610975	-1.434417	2.888774
C	0.973201	3.372923	0.418227
C	0.182393	-0.121264	2.788922
C	-1.021985	1.739356	3.177518
H	-1.704510	2.489315	3.555898
C	2.607264	3.111309	-1.028703
C	1.142271	4.630954	-0.253712
H	0.556999	5.517971	-0.049508
C	2.157823	4.470172	-1.146974
H	2.578103	5.196965	-1.829683
O	0.539901	-0.058764	-0.957221
C	-1.277131	-1.407321	-1.607952
C	-0.033835	-1.194407	-1.000015
H	0.483597	-2.035003	-0.524686
C	-1.912440	-2.689381	-1.778303
H	4.124990	3.204307	-2.505721
H	-0.602220	3.971259	1.703215
H	0.149159	-2.061350	3.644133
H	4.697133	-2.899707	-0.755115
H	-1.715070	-0.540429	-2.093119
C	-1.038210	-3.944372	-1.749351
H	-0.334173	-3.976090	-0.915570
H	-1.654087	-4.847754	-1.706239
H	-0.455996	-3.987524	-2.677646
C	-2.907514	-2.723946	-2.936169
H	-2.358677	-2.738111	-3.886209
H	-3.536686	-3.619275	-2.904728
H	-3.554260	-1.842148	-2.940306
C	-4.579502	0.497817	-0.278235
C	-4.667464	1.632978	0.544977
C	-5.383515	0.439805	-1.428343
C	-5.532659	2.678827	0.228561
H	-4.069640	1.679910	1.451892
C	-6.249028	1.485674	-1.744952
H	-5.318984	-0.415519	-2.095437
C	-6.326738	2.607987	-0.917662
H	-5.596281	3.543854	0.882658
H	-6.857740	1.427276	-2.642630
H	-7.003655	3.420768	-1.164005
H	-4.918973	-2.105988	-0.672572
H	-2.003196	0.763016	0.438441
C	-2.111827	-3.320930	0.978869
H	-1.156211	-2.795720	0.909773
H	-2.575281	-3.032056	1.928451
H	-1.889418	-4.389834	1.025057
H	-3.548662	-3.951559	-0.531683

^{4}TS2E1C$_{exo\text{-}trans\text{-}m}$

C	-3.556728	-0.448941	0.174470
C	-4.243072	-1.608797	-0.216176
C	-3.660845	-2.892557	-0.238887
C	-2.266337	-0.571799	0.681504
H	-1.947262	-1.453873	1.224321
Fe	1.910254	0.394024	0.362972
N	2.311348	-1.400120	1.162689
N	0.703325	0.786853	1.922887
C	0.734015	-1.399606	3.052654
C	1.676819	-2.002163	2.237441
C	3.054329	-3.577910	1.410640
H	3.625023	-4.476245	1.214520
C	4.025037	-2.188954	-0.416782
C	0.293411	-0.094676	2.908224
C	3.165405	-2.364187	0.654153
C	2.134237	-3.353351	2.392185
H	1.796540	-4.029941	3.166492

C	-0.024853	1.944553	2.139387
C	-0.690441	0.521924	3.751090
H	-1.157738	0.041740	4.600950
C	-0.887519	1.785312	3.274641
H	-1.547566	2.553969	3.655350
N	1.795761	2.317137	-0.175305
N	3.446758	0.154327	-0.893864
C	3.449543	2.353694	-1.994477
C	2.459343	2.932552	-1.219990
C	1.013774	4.477582	-0.458962
H	0.425325	5.367350	-0.277285
C	0.046485	3.093447	1.370282
C	3.908655	1.058741	-1.832794
C	0.905241	3.263209	0.299323
C	1.980211	4.274486	-1.397191
H	2.348916	4.962424	-2.146683
C	4.163993	-1.006351	-1.121306
C	4.933815	0.461311	-2.641528
H	5.458044	0.970136	-3.439826
C	5.089653	-0.818813	-2.203119
H	5.769559	-1.578590	-2.565771
O	0.453971	-0.240024	-0.872034
C	-1.450268	-1.507716	-1.408806
C	-0.202656	-1.320367	-0.781380
H	0.228361	-2.130525	-0.181036
C	-2.106856	-2.766159	-1.622695
H	4.648053	-3.027771	-0.708075
H	3.894721	2.954849	-2.779832
H	-0.599057	3.922745	1.638391
H	0.322075	-1.984982	3.867906
H	-1.808920	-0.643298	-1.961152
C	-1.334945	-4.034836	-1.275206
H	-0.905766	-4.013107	-0.268418
H	-1.972222	-4.919091	-1.346021
H	-0.512548	-4.174983	-1.987417
C	-2.863636	-2.850751	-2.942150
H	-2.149426	-2.911505	-3.773737
H	-3.496889	-3.740213	-2.992179
H	-3.489022	-1.966889	-3.101174
C	-4.078368	0.904485	-0.149889
C	-3.841157	1.976604	0.727368
C	-4.785848	1.153837	-1.337477
C	-4.297666	3.258189	0.426537
H	-3.315907	1.793612	1.660821
C	-5.244264	2.435930	-1.637358
H	-4.956582	0.346453	-2.044660
C	-5.001418	3.492224	-0.756929
H	-4.117175	4.073134	1.122426
H	-5.784380	2.611746	-2.563218
H	-5.361046	4.490109	-0.990546
H	-5.219435	-1.489390	-0.682300
H	-1.638715	0.301659	0.802318
C	-4.551942	-4.070603	-0.569355
H	-3.987039	-4.950904	-0.886555
H	-5.122014	-4.362040	0.321694
H	-5.271803	-3.823475	-1.356010
H	-2.925708	-3.102182	0.536204

Reaction E2

^{4}IE2

Fe	0.000000	-0.019887	0.000001
N	1.178744	-1.432134	-0.789412
N	1.177084	1.394742	-0.792618
O	-1.197814	-0.019707	-1.882365
C	2.826935	-0.018898	-1.947946
C	2.299444	-1.245626	-1.575993
C	2.058509	-3.477095	-1.408133
H	2.155451	-4.552582	-1.479859
C	-3.199914	0.047580	-3.134978
C	0.000049	-3.443146	-0.000033
C	2.294826	1.208474	-1.583936
C	1.017484	-2.800238	-0.686434
C	2.852011	-2.514765	-1.959192
H	3.732491	-2.639226	-2.576096
C	-2.428137	0.056644	-1.902237
H	-2.986750	0.135576	-0.954356
C	1.013478	2.763094	-0.692490
C	2.842226	2.477555	-1.973896
H	3.719530	2.602306	-2.595264
C	2.049188	3.439857	-1.421577
H	2.143640	4.515336	-1.496918
C	-4.538486	0.138223	-3.095755
H	-5.074507	0.219554	-2.152148
N	-1.177126	1.394692	0.792648
N	-1.178703	-1.432183	0.789385
C	-2.826937	-0.019018	1.947946
C	-2.294862	1.208377	1.583962
C	-2.049287	3.439770	1.421648

H	-2.143769	4.515245	1.497011
C	-0.000049	3.405786	0.000035
C	-2.299410	-1.245723	1.575968
C	-1.013558	2.763051	0.692548
C	-2.842298	2.477434	1.973947
H	-3.719606	2.602148	2.595318
C	-1.017404	-2.800281	0.686380
C	-2.851941	-2.514886	1.959141
H	-3.732418	-2.639384	2.576042
C	-2.058410	-3.477182	1.408065
H	-2.155322	-4.552672	1.479769
O	1.197814	-0.019709	1.882366
C	3.199918	0.047575	3.134974
C	2.428137	0.056647	1.902235
H	2.986747	0.135592	0.954353
C	4.538489	0.138222	3.095748
H	5.074507	0.219563	2.152140
H	0.000065	-4.527889	-0.000044
H	-3.712936	-0.019504	2.573876
H	-0.000064	4.490554	0.000047
H	3.712934	-0.019346	-2.573877
H	-2.644176	-0.033552	-4.065298
H	-5.141306	0.134924	-3.998663
H	2.644182	-0.033567	4.065294
H	5.141311	0.134915	3.998654

^{6}IE2

Fe	-0.000081	0.002680	-0.000118
N	1.239166	-1.447711	-0.764663
N	1.241018	1.450511	-0.765087
O	-1.102581	0.002544	-1.879871
C	2.907154	0.000208	-1.855759
C	2.377676	-1.246800	-1.516067
C	2.117265	-3.486283	-1.362914
H	2.218645	-4.560958	-1.443149
C	-3.045157	-0.005768	-3.219057
C	-0.001631	-3.434631	0.001369
C	2.379581	1.247919	-1.515921
C	1.054664	-2.810200	-0.664091
C	2.932669	-2.522620	-1.888628
H	3.828437	-2.658170	-2.481032
C	-2.336003	-0.006858	-1.952354
H	-2.936492	-0.016382	-1.028771
C	1.058141	2.813243	-0.665040
C	2.936399	2.522957	-1.888537
H	3.832607	2.657193	-2.480572
C	2.121965	3.487786	-1.363507
H	2.224721	4.562303	-1.444024
C	-4.387846	-0.017019	-3.239610
H	-4.970016	-0.027027	-2.320307
N	-1.239413	1.452307	0.764268
N	-1.240779	-1.445939	0.765462
C	-2.907497	0.004439	1.855233
C	-2.378431	1.251378	1.514862
C	-2.117962	3.490879	1.361814
H	-2.219386	4.565550	1.441963
C	0.002091	3.439198	-0.000655
C	-2.379230	-1.243351	1.516510
C	-1.054805	2.814776	0.663833
C	-2.933775	2.527230	1.886890
H	-3.829989	2.662778	2.478616
C	-1.057420	-2.808674	0.666192
C	-2.935425	-2.518349	1.890093
H	-3.831424	-2.652582	2.482448
C	-2.120722	-3.483204	1.365464
H	-2.223021	-4.557716	1.446688
O	1.102404	0.002085	1.879899
C	3.045138	-0.006598	3.218852
C	2.335834	-0.006879	1.952226
H	2.936223	-0.015495	1.028562
C	4.387829	-0.017112	3.239278
H	4.969928	-0.025884	2.319915
H	-0.002086	-4.520192	0.001883
H	-3.813513	0.005130	2.453242
H	0.002784	4.524750	-0.000816
H	3.813032	-0.000451	-2.453981
H	-2.443752	0.004149	-4.124001
H	-4.947459	-0.016781	-4.169917
H	2.443808	0.002211	4.123859
H	4.947525	-0.017307	4.169537

^{4}TS1E2O$_{endo\text{-}cis\text{-}p}$

C	2.985799	-2.242722	-0.351908
C	3.524407	-1.086970	0.302975
C	2.721328	-0.251427	1.114524
H	1.901110	-0.697543	1.669448
H	3.232725	0.537774	1.658208
C	1.690341	-2.646083	-0.271394

H	1.002656	-2.145615	0.398926
C	4.893917	-0.664873	-0.029316
C	5.426320	-0.869682	-1.319797
C	5.702442	-0.027746	0.934123
C	6.712157	-0.442900	-1.635167
H	4.813658	-1.325028	-2.091262
C	6.996010	0.378478	0.622303
H	5.330144	0.108371	1.944549
C	7.502979	0.177412	-0.664111
H	7.098230	-0.591597	-2.639166
H	7.611965	0.847664	1.383624
H	8.510686	0.500647	-0.908303
H	3.657383	-2.807295	-0.993571
C	1.121209	-3.760535	-1.076970
H	0.339621	-3.362598	-1.738469
H	1.875003	-4.268332	-1.685719
H	0.621216	-4.490080	-0.427764
Fe	-1.218391	0.222089	0.082283
N	-1.105746	2.210794	-0.216124
N	-0.723056	0.497029	2.015651
O	-3.403213	0.683282	0.800882
C	-0.438776	2.940283	2.033657
C	-0.755484	3.173958	0.702997
C	-1.035938	4.279381	-1.238340
H	-1.109664	5.016587	-2.027199
C	-5.749417	0.371592	0.823286
C	-1.662028	2.282524	-2.607273
C	-0.416267	1.689416	2.635916
C	-1.281708	2.871156	-1.410227
C	-0.716781	4.468277	0.072497
H	-0.473222	5.392208	0.580866
C	-4.388804	0.079771	0.384388
H	-4.266601	-0.727777	-0.359253
C	-0.579204	-0.478998	2.977991
C	-0.051238	1.453008	4.007117
H	0.230686	2.226419	4.709800
C	-0.144838	0.109058	4.217246
H	0.041522	-0.444906	5.128227
C	-6.785999	-0.321223	0.329481
H	-6.640472	-1.111615	-0.404472
N	-1.596371	-1.721304	0.460245
N	-1.919515	-0.021806	-1.783135
C	-2.432800	-2.425597	-1.743329
C	-2.100353	-2.660500	-0.415893
C	-1.737125	-3.775019	1.507913
H	-1.658086	-4.513213	2.295563
C	-0.859907	-1.827730	2.806702
C	-2.324060	-1.196822	-2.379373
C	-1.367708	-2.392051	1.645490
C	-2.200322	-3.938740	0.234938
H	-2.572621	-4.839966	-0.235108
C	-1.963347	0.939042	-2.771150
C	-2.633519	-0.967015	-3.766143
H	-2.979281	-1.728846	-4.452888
C	-2.403951	0.353819	-4.009712
H	-2.524575	0.898731	-4.937003
O	0.698797	-0.028910	-0.535141
C	2.556414	1.329389	-1.139960
C	1.659218	0.750716	-0.104915
H	1.387579	1.472200	0.674609
C	3.057540	2.562769	-1.053499
H	2.844343	3.204079	-0.200326
H	-1.751849	2.925704	-3.476361
H	-2.794842	-3.265238	-2.327658
H	-0.712592	-2.482718	3.659080
H	-0.176703	3.796815	2.645947
H	-5.870463	1.165333	1.555865
H	-7.808727	-0.125735	0.636890
H	2.754134	0.691983	-1.998669
H	3.690316	2.980829	-1.830588

^{6}TS1E2O$_{endo\text{-}cis\text{-}p}$

C	2.991355	-2.252844	-0.393397
C	3.525694	-1.099015	0.279118
C	2.734894	-0.289697	1.116932
H	1.885931	-0.725165	1.634167
H	3.235856	0.497783	1.671615
C	1.701161	-2.667783	-0.323661
H	0.999915	-2.165042	0.331909
C	4.892230	-0.666049	-0.054043
C	5.413577	-0.842752	-1.353102
C	5.704857	-0.040942	0.913745
C	6.692600	-0.398779	-1.672910
H	4.798117	-1.291003	-2.126546
C	6.991755	0.382334	0.597018
H	5.341921	0.071714	1.930463
C	7.487445	0.210259	-0.698071
H	7.070428	-0.526201	-2.682938

H	7.611592	0.841594	1.361209
H	8.490086	0.546310	-0.945744
H	3.673201	-2.810080	-1.030547
C	1.148543	-3.796017	-1.121894
H	0.358716	-3.418073	-1.785375
H	1.910137	-4.297664	-1.725956
H	0.660598	-4.527508	-0.465596
Fe	-1.156677	0.218208	0.040057
N	-1.133301	2.275833	-0.186036
N	-0.670945	0.461443	2.045050
O	-3.298357	0.652115	0.842132
C	-0.374913	2.906837	2.066854
C	-0.742601	3.200380	0.750807
C	-1.158988	4.365197	-1.140736
H	-1.291686	5.132818	-1.892244
C	-5.634268	0.317632	0.994885
C	-1.849066	2.375311	-2.536696
C	-0.348615	1.644227	2.668048
C	-1.399366	2.956024	-1.347989
C	-0.755834	4.516507	0.154899
H	-0.494097	5.431975	0.669892
C	-4.298033	0.031751	0.485741
H	-4.206496	-0.783636	-0.252216
C	-0.556767	-0.538109	2.981361
C	0.012436	1.376217	4.039498
H	0.308553	2.128014	4.759882
C	-0.114088	0.029422	4.232592
H	0.058850	-0.533340	5.141029
C	-6.688667	-0.394996	0.571658
H	-6.575284	-1.196427	-0.155915
N	-1.588841	-1.777473	0.410167
N	-2.012155	0.020433	-1.825656
C	-2.455054	-2.399180	-1.810863
C	-2.093030	-2.687858	-0.491435
C	-1.730383	-3.837535	1.421752
H	-1.657863	-4.594940	2.191862
C	-0.860408	-1.885340	2.763449
C	-2.409501	-1.148180	-2.431946
C	-1.362938	-2.452897	1.590190
C	-2.185283	-3.981115	0.140219
H	-2.553416	-4.879186	-0.339479
C	-2.132925	1.026064	-2.758003
C	-2.793783	-0.870963	-3.795563
H	-3.150159	-1.610836	-4.500893
C	-2.621152	0.468651	-3.996630
H	-2.809833	1.037341	-4.898143
O	0.709084	0.022499	-0.580684
C	2.597400	1.347354	-1.160327
C	1.673322	0.793681	-0.146895
H	1.420520	1.492818	0.656597
C	3.168662	2.547085	-1.029384
H	2.990326	3.169523	-0.154794
H	-2.007857	3.047042	-3.374783
H	-2.818378	-3.229055	-2.409528
H	-0.726313	-2.555666	3.607018
H	-0.091052	3.748074	2.691920
H	-5.723127	1.122145	1.720215
H	-7.694649	-0.205392	0.933241
H	2.762041	0.727176	-2.037914
H	3.826298	2.953605	-1.791457

[4]IIIE2O$_{endo\text{-}cis\text{-}p}$

C	2.672450	-0.342256	-1.604898
C	3.369818	0.134013	-0.561499
C	2.664869	0.999268	0.464443
H	2.351635	0.392502	1.322805
H	3.346840	1.765516	0.850472
C	1.209965	-0.105342	-1.843611
H	0.697382	-1.068983	-1.781038
C	4.806438	-0.167487	-0.357422
C	5.674815	-0.369482	-1.444455
C	5.336177	-0.259270	0.941305
C	7.018503	-0.674130	-1.239993
H	5.299604	-0.257238	-2.457780
C	6.680744	-0.567119	1.146368
H	4.690642	-0.106554	1.801851
C	7.527288	-0.777538	0.056997
H	7.673379	-0.817565	-2.094939
H	7.067404	-0.641486	2.159068
H	8.576339	-1.009834	0.216038
H	3.147601	-0.985683	-2.341345
C	0.897146	0.503974	-3.213436
H	-0.181248	0.638354	-3.326081
H	1.386375	1.470421	-3.347984
H	1.246570	-0.173740	-4.000469
Fe	-1.227542	-0.017115	0.227539
N	-1.857849	1.876175	0.356699
N	-0.404950	0.140849	2.043176

O	-3.137883	-0.611829	1.262613
C	-1.096184	2.428113	2.628713
C	-1.726063	2.732013	1.431086
C	-2.757051	3.936786	-0.167501
H	-3.246416	4.703472	-0.753871
C	-5.083312	-1.905574	1.620591
C	-2.883531	2.111335	-1.865675
C	-0.466117	1.223384	2.899221
C	-2.497818	2.599980	-0.627405
C	-2.290578	4.015065	1.111930
H	-2.315247	4.860221	1.787447
C	-3.849708	-1.560586	0.930183
H	-3.557314	-2.190066	0.072712
C	0.349312	-0.813391	2.696115
C	0.268157	0.943470	4.102147
H	0.372807	1.631307	4.931037
C	0.782596	-0.312681	3.971822
H	1.391638	-0.867274	4.673759
C	-5.818734	-2.950961	1.211402
H	-5.515588	-3.561226	0.362811
N	-0.667445	-1.940927	0.133462
N	-2.122484	-0.209424	-1.553556
C	-1.685565	-2.587778	-2.011763
C	-0.952935	-2.855839	-0.864845
C	0.356243	-3.959718	0.596894
H	0.954996	-4.687833	1.128378
C	0.629936	-2.084645	2.220007
C	-2.206580	-1.343338	-2.338106
C	0.135379	-2.610649	1.037215
C	-0.327488	-4.115684	-0.572863
H	-0.398603	-4.995778	-1.198778
C	-2.726341	0.798662	-2.281115
C	-2.881610	-1.042100	-3.569800
H	-3.075960	-1.760920	-4.355197
C	-3.193742	0.285711	-3.538799
H	-3.699860	0.875388	-4.291984
O	0.598315	0.671096	-0.775416
C	1.775823	2.820032	-1.066163
C	1.439095	1.687527	-0.132276
H	0.800247	2.068345	0.661348
C	1.094787	3.965868	-1.084016
H	0.241849	4.135054	-0.429683
H	-3.375672	2.797804	-2.546253
H	-1.825017	-3.395621	-2.722256
H	1.249305	-2.727749	2.836011
H	-1.062373	3.198508	3.391491
H	-5.367639	-1.283186	2.464887
H	-6.743311	-3.231914	1.706260
H	2.630829	2.672903	-1.723224
H	1.363438	4.779204	-1.752353

^{4}TS2E2O$_{endo\text{-}cis\text{-}p}$

C	2.434944	-2.236916	-0.539376
C	3.037220	-1.109424	0.118588
C	2.323398	-0.313193	1.036441
H	1.518697	-0.759454	1.612973
H	2.878040	0.452686	1.569988
C	1.148566	-2.637116	-0.375134
H	0.516028	-2.150229	0.356983
C	4.377957	-0.682316	-0.310925
C	4.793760	-0.833052	-1.650914
C	5.272407	-0.090773	0.604599
C	6.050116	-0.397193	-2.059125
H	4.113897	-1.254095	-2.384723
C	6.536585	0.324220	0.198275
H	4.992188	0.000343	1.649227
C	6.927426	0.177354	-1.135288
H	6.346188	-0.504099	-3.098361
H	7.220583	0.756775	0.922210
H	7.912482	0.506586	-1.452742
H	3.052693	-2.781710	-1.248511
C	0.509763	-3.727376	-1.162243
H	-0.320358	-3.310954	-1.749105
H	1.209674	-4.222111	-1.841714
H	0.059768	-4.472178	-0.493825
Fe	-1.635735	0.227754	0.226566
N	-1.675481	2.229030	0.037981
N	-1.116234	0.454796	2.155566
C	-0.835422	2.898002	2.250058
C	-1.260643	3.170875	0.960346
C	-1.698543	4.339414	-0.911253
H	-1.841542	5.103521	-1.664060
C	-2.397206	2.394662	-2.306101
C	-0.762417	1.627630	2.799277
C	-1.941051	2.938644	-1.117687
C	-1.285019	4.484728	0.378625
H	-1.016136	5.392380	0.903139
C	-0.876091	-0.554003	3.072582

C	-0.286261	1.345629	4.123856
H	0.041609	2.097120	4.830277
C	-0.347930	-0.006387	4.289901
H	-0.084550	-0.591152	5.161659
N	-2.091759	-1.692163	0.612035
N	-2.579671	0.068026	-1.530381
C	-3.134556	-2.326476	-1.523808
C	-2.687945	-2.602307	-0.242225
C	-2.158981	-3.785996	1.597303
H	-2.019193	-4.549922	2.351097
C	-1.154452	-1.897089	2.878273
C	-3.065753	-1.080702	-2.123753
C	-1.763868	-2.413649	1.746940
C	-2.742371	-3.899843	0.369984
H	-3.174242	-4.777858	-0.092515
C	-2.710150	1.058129	-2.485260
C	-3.502448	-0.805063	-3.464821
H	-3.932250	-1.540169	-4.132697
C	-3.273920	0.518252	-3.691668
H	-3.479151	1.095465	-4.583726
O	0.194368	0.040415	-0.512212
C	2.034281	1.411140	-1.142158
C	1.176181	0.800681	-0.100727
H	0.963174	1.467031	0.743005
C	2.598345	2.612282	-0.996309
H	2.465136	3.197405	-0.088345
H	-2.556998	3.067622	-3.141680
H	-3.566081	-3.141100	-2.095733
H	-0.931564	-2.581895	3.689570
H	-0.531007	3.734566	2.870036
H	2.156907	0.828625	-2.051954
H	3.206663	3.057453	-1.777541

^{6}TS2E2O$_{endo\text{-}cis\text{-}p}$

C	2.475517	-2.169733	-0.611119
C	3.092130	-1.036669	0.031512
C	2.408018	-0.248639	0.970823
H	1.589086	-0.673087	1.542618
H	2.963278	0.527954	1.486802
C	1.202123	-2.585488	-0.404211
H	0.589451	-2.104013	0.348988
C	4.420718	-0.607647	-0.434107
C	4.799861	-0.760276	-1.784657
C	5.336708	-0.007917	0.454388
C	6.041964	-0.318712	-2.229022
H	4.103136	-1.188497	-2.498225
C	6.586571	0.413296	0.011658
H	5.085681	0.084022	1.506364
C	6.941174	0.264055	-1.331796
H	6.309911	-0.428216	-3.275573
H	7.288016	0.851732	0.715040
H	7.915351	0.597207	-1.677413
H	3.076930	-2.710132	-1.337205
C	0.548935	-3.689948	-1.157990
H	-0.315401	-3.294762	-1.708917
H	1.227701	-4.175631	-1.864687
H	0.141919	-4.439814	-0.468379
Fe	-1.507194	0.178385	0.183881
N	-1.633741	2.231094	0.494202
N	-1.001786	0.003457	2.196322
C	-0.560855	2.372205	2.703356
C	-1.092496	2.939286	1.547173
C	-1.704302	4.489490	0.026745
H	-1.908488	5.402578	-0.517323
C	-2.605388	2.874673	-1.674289
C	-0.525543	1.013024	3.009277
C	-2.011482	3.165037	-0.449275
C	-1.137372	4.350635	1.260049
H	-0.785467	5.127488	1.926604
C	-0.834129	-1.174213	2.900054
C	-0.017753	0.452497	4.234402
H	0.407033	1.027943	5.046851
C	-0.208064	-0.898180	4.166986
H	0.031141	-1.645270	4.912781
N	-2.175619	-1.791447	0.278160
N	-2.735976	0.427587	-1.462436
C	-3.320933	-1.919625	-1.899365
C	-2.845045	-2.478987	-0.715677
C	-2.417923	-4.003101	0.892532
H	-2.351169	-4.896183	1.500577
C	-1.252139	-2.430715	2.469053
C	-3.276305	-0.572106	-2.243827
C	-1.911519	-2.710606	1.274594
C	-2.994988	-3.859594	-0.336726
H	-3.491506	-4.612621	-0.934990
C	-2.951414	1.608895	-2.139819
C	-3.828098	-0.005811	-3.448442
H	-4.317341	-0.573656	-4.229198

C	-3.626286	1.341659	-3.384686
H	-3.917948	2.097198	-4.102616
O	0.256114	0.151702	-0.621029
C	2.112749	1.501351	-1.242734
C	1.237328	0.915689	-0.212312
H	1.037429	1.562591	0.648292
C	2.745053	2.665689	-1.068790
H	2.650731	3.234051	-0.145682
H	-2.844329	3.716811	-2.316084
H	-3.806733	-2.591400	-2.600131
H	-1.084553	-3.263184	3.145254
H	-0.159840	3.054132	3.446766
H	2.200403	0.934596	-2.166017
H	3.372701	3.094609	-1.843459

^{4}TS1E2O$_{exo\text{-}trans\text{-}p}$

Fe	-0.901920	-0.103227	0.163004
N	-1.498730	1.806802	-0.129149
N	0.609771	0.554205	1.307640
O	-2.171297	-0.041763	2.130496
C	0.199745	2.979595	1.202785
C	-0.908753	2.947443	0.367623
C	-2.552644	3.669503	-0.992672
H	-3.255699	4.251798	-1.574098
C	-4.130435	0.474372	3.354264
C	-3.372098	1.401575	-1.666008
C	0.907692	1.863583	1.626697
C	-2.503164	2.231902	-0.971401
C	-1.573332	4.112903	-0.154039
H	-1.307254	5.133248	0.090253
C	-3.306152	0.428871	2.151547
H	-3.745887	0.844115	1.227227
C	1.574292	-0.214521	1.921430
C	2.091187	1.912931	2.442616
H	2.537435	2.822765	2.822442
C	2.511737	0.627842	2.612927
H	3.376539	0.268255	3.153713
C	-5.361681	1.004405	3.313630
H	-5.773276	1.409381	2.391051
N	-0.479195	-1.988557	0.696020
N	-2.544932	-0.744952	-0.792604
C	-2.402318	-3.148877	-0.304478
C	-1.236001	-3.112863	0.445993
C	0.546653	-3.842091	1.613548
H	1.281863	-4.422971	2.155569
C	1.600037	-1.600903	1.949835
C	-2.984401	-2.045212	-0.911966
C	0.611063	-2.417240	1.421850
C	-0.606561	-4.270065	1.024794
H	-1.003592	-5.275642	0.972708
C	-3.398144	0.017791	-1.562274
C	-4.136158	-2.099942	-1.772598
H	-4.673756	-3.005441	-2.022660
C	-4.383580	-0.824851	-2.186428
H	-5.168759	-0.470235	-2.841608
O	0.143929	-0.020353	-1.592101
C	0.423821	1.884116	-2.989594
C	0.881240	1.015369	-1.872139
H	1.307073	1.566597	-1.024296
C	0.605501	3.206997	-2.999440
H	1.125773	3.713793	-2.189269
H	-4.114859	1.871239	-2.302416
H	-2.878408	-4.113146	-0.448896
H	2.414865	-2.076234	2.485743
H	0.555113	3.951904	1.528065
H	-3.698351	0.064503	4.263468
H	-5.997663	1.052950	4.192205
H	-0.116712	1.371630	-3.782951
H	0.226759	3.833033	-3.801488
C	2.504632	-1.804606	-1.524480
C	3.074609	-0.493269	-1.650359
C	2.584405	0.398287	-2.623882
H	3.128470	1.319386	-2.800958
H	2.171570	-0.023182	-3.533685
C	1.531488	-2.295600	-2.336390
H	1.215380	-1.732600	-3.208401
C	4.048909	-0.035699	-0.644000
C	4.847446	-0.959304	0.063036
C	4.227365	1.336030	-0.368936
C	5.786717	-0.528885	0.995311
H	4.767100	-2.020260	-0.147759
C	5.157441	1.763580	0.572772
H	3.616980	2.076996	-0.873271
C	5.944411	0.834610	1.256885
H	6.405585	-1.257928	1.510111
H	5.267907	2.824984	0.774144
H	6.678796	1.169922	1.983443
H	2.781716	-2.398693	-0.659992

C	0.853711	-3.606030	-2.153364
H	1.044501	-4.252479	-3.021566
H	1.171244	-4.122696	-1.244840
H	-0.232076	-3.457131	-2.109182

^{6}TS1E2O$_{exo\text{-}trans\text{-}p}$

Fe	-0.874910	-0.113324	0.064084
N	-1.291541	1.939952	0.046307
N	0.720014	0.265439	1.325346
O	-2.059646	-0.108339	2.070737
C	0.625489	2.726338	1.374046
C	-0.516870	2.933393	0.594456
C	-2.126940	3.983256	-0.595407
H	-2.772533	4.707689	-1.075232
C	-3.897019	0.435618	3.456044
C	-3.282495	1.885652	-1.399448
C	1.190338	1.496828	1.720796
C	-2.277886	2.551304	-0.691635
C	-1.043573	4.219567	0.202846
H	-0.631216	5.174857	0.501975
C	-3.138733	0.463751	2.210826
H	-3.574172	1.029989	1.369770
C	1.513178	-0.684682	1.924609
C	2.346920	1.314793	2.564620
H	2.922320	2.117260	3.006985
C	2.547312	-0.029877	2.688136
H	3.320741	-0.539827	3.246500
C	-5.071477	1.076400	3.550006
H	-5.483706	1.628996	2.707911
N	-0.733202	-2.121180	0.486263
N	-2.701904	-0.443085	-0.848147
C	-2.783999	-2.893748	-0.636686
C	-1.629072	-3.099708	0.120726
C	-0.026306	-4.138933	1.329298
H	0.583479	-4.853867	1.866967
C	1.304762	-2.064208	1.867401
C	-3.270735	-1.669032	-1.100679
C	0.245202	-2.724160	1.241562
C	-1.184350	-4.369687	0.641667
H	-1.701655	-5.310380	0.502823
C	-3.485824	0.504876	-1.464627
C	-4.453444	-1.488923	-1.907840
H	-5.096695	-2.289377	-2.250322
C	-4.582170	-0.148820	-2.138264
H	-5.353256	0.359920	-2.702596
O	0.113377	0.044320	-1.650920
C	0.493388	1.969629	-2.993627
C	0.879626	1.068649	-1.886296
H	1.338128	1.560389	-1.022406
C	0.745547	3.282167	-2.968245
H	1.274255	3.742546	-2.136543
H	-3.993799	2.507449	-1.934608
H	-3.370570	-3.774523	-0.879800
H	2.004193	-2.683279	2.421297
H	1.123071	3.613859	1.753403
H	-3.465962	-0.122651	4.282975
H	-5.658619	1.072093	4.463223
H	-0.059627	1.503500	-3.806108
H	0.414485	3.944277	-3.762154
C	2.451387	-1.819098	-1.529582
C	3.051820	-0.515880	-1.664451
C	2.627220	0.364264	-2.668296
H	3.190697	1.272822	-2.845997
H	2.168702	-0.042300	-3.562084
C	1.528431	-2.335097	-2.379526
H	1.255208	-1.791731	-3.279523
C	4.016365	-0.066555	-0.643286
C	4.793944	-0.999137	0.073682
C	4.214882	1.303640	-0.374421
C	5.738911	-0.578549	1.005412
H	4.694583	-2.059888	-0.130104
C	5.148384	1.721243	0.568212
H	3.620819	2.051677	-0.888109
C	5.918925	0.782793	1.258826
H	6.343021	-1.315024	1.527066
H	5.276927	2.781908	0.763186
H	6.656462	1.110100	1.985777
H	2.681871	-2.388298	-0.635132
C	0.841700	-3.641383	-2.197141
H	1.056231	-4.303136	-3.047762
H	1.128919	-4.142883	-1.270621
H	-0.245099	-3.491548	-2.186134

^{4}IIIE2O$_{exo\text{-}trans\text{-}p}$

Fe	0.886473	0.192940	0.233562
N	1.923405	-1.501883	0.044967
N	-0.426458	-0.739934	1.403201
O	2.078934	0.651485	2.100397

C	0.703149	-2.913105	1.645588
C	1.722329	-2.682584	0.731455
C	3.431811	-3.090035	-0.678085
H	4.242196	-3.526814	-1.247033
C	4.091201	0.793522	3.332928
C	3.579999	-0.777178	-1.619534
C	-0.314818	-2.014028	1.929039
C	2.975317	-1.735371	-0.818188
C	2.675115	-3.667652	0.299981
H	2.733871	-4.676569	0.687385
C	3.299537	0.492145	2.149821
H	3.835107	0.104401	1.266405
C	-1.620004	-0.235191	1.877351
C	-1.460824	-2.307765	2.745390
H	-1.614851	-3.239736	3.273663
C	-2.278623	-1.216806	2.693387
H	-3.240504	-1.069699	3.165114
C	5.419394	0.601459	3.324570
H	5.931792	0.219318	2.443765
N	-0.090862	1.893818	0.499223
N	2.276072	1.148759	-0.819915
C	1.545339	3.466205	-0.453433
C	0.348619	3.165188	0.181449
C	-1.716411	3.439517	1.040814
H	-2.657883	3.825251	1.409464
C	-2.086994	1.052117	1.664908
C	2.415954	2.514676	-0.965422
C	-1.343628	2.053121	1.060999
C	-0.657985	4.130762	0.525597
H	-0.560880	5.198113	0.375390
C	3.280340	0.577173	-1.577587
C	3.530498	2.803289	-1.823701
H	3.853999	3.799399	-2.096447
C	4.046740	1.604824	-2.225979
H	4.886244	1.422172	-2.884155
O	-0.190921	-0.469830	-1.752405
C	-0.226238	-2.729073	-2.609800
C	-0.856300	-1.766293	-1.645837
H	-0.675371	-2.093296	-0.625533
C	0.231914	-3.930764	-2.262737
H	0.204815	-4.271746	-1.230806
H	4.399231	-1.094087	-2.256066
H	1.769498	4.510753	-0.641235
H	-3.065479	1.307381	2.055653
H	0.663316	-3.887008	2.121944
H	3.558794	1.174198	4.200466
H	6.037068	0.817818	4.190783
H	-0.190731	-2.404147	-3.649447
H	0.647417	-4.619503	-2.992428
C	-2.229979	0.749631	-1.951309
C	-2.951084	-0.289774	-1.490160
C	-2.396613	-1.651326	-1.861045
H	-2.870320	-2.472498	-1.320347
H	-2.619065	-1.832654	-2.921517
C	-0.940714	0.432395	-2.649290
H	-1.122663	-0.155795	-3.560275
C	-4.164846	-0.139441	-0.659294
C	-4.940499	1.033859	-0.726353
C	-4.547746	-1.126692	0.264515
C	-6.031070	1.224801	0.117924
H	-4.697764	1.793993	-1.462732
C	-5.640469	-0.937350	1.110749
H	-3.968575	-2.039326	0.357030
C	-6.384209	0.241703	1.047231
H	-6.617178	2.136380	0.040751
H	-5.911565	-1.715721	1.819099
H	-7.239331	0.387251	1.700878
H	-2.469726	1.785107	-1.730490
C	-0.092111	1.626796	-3.029899
H	-0.582124	2.157410	-3.853329
H	0.017769	2.321725	-2.199866
H	0.898831	1.306405	-3.361219

^{4}TS1E2C$_{endo-cis-p}$

C	-3.896691	-1.487810	0.114547
C	-4.816544	-0.499886	0.609790
C	-4.458516	0.368190	1.639875
H	-5.215026	1.024141	2.058661
H	-3.678049	0.089282	2.337377
C	-2.655497	-1.752786	0.608544
H	-2.328747	-1.305327	1.544047
C	-6.090431	-0.304596	-0.119384
C	-6.752816	-1.399353	-0.707305
C	-6.679117	0.969399	-0.238695
C	-7.958375	-1.226747	-1.383156
H	-6.342988	-2.399352	-0.602238
C	-7.876546	1.142425	-0.927606
H	-6.189009	1.837836	0.190900

C	-8.522401	0.044973	-1.501904
H	-8.460150	-2.088206	-1.814097
H	-8.305154	2.136266	-1.018886
H	-9.458814	0.179873	-2.035103
H	-4.180682	-2.000123	-0.801682
C	-1.684624	-2.676658	-0.041148
H	-1.278651	-3.395953	0.679114
H	-2.130772	-3.218801	-0.880636
H	-0.827743	-2.099238	-0.417510
Fe	1.622187	0.212132	-0.054882
N	1.146777	0.917728	1.761466
N	1.839447	2.087091	-0.725952
O	3.811995	0.375743	0.661505
C	1.250964	3.333810	1.308155
C	1.032453	2.242119	2.133678
C	0.462083	1.057096	3.961884
H	0.163513	0.726010	4.948211
C	6.030112	-0.398351	0.937532
C	0.817763	-1.204332	2.962641
C	1.622601	3.252845	-0.024577
C	0.809915	0.179920	2.877749
C	0.606109	2.333898	3.502728
H	0.445090	3.262838	4.034321
C	4.650207	-0.505183	0.479246
H	4.368943	-1.429902	-0.054028
C	2.194261	2.472761	-1.999876
C	1.838331	4.391084	-0.877246
H	1.734465	5.421604	-0.563426
C	2.189052	3.907775	-2.101963
H	2.432954	4.459806	-3.000316
C	6.899378	-1.394286	0.710315
H	6.599998	-2.298092	0.183074
N	2.309229	-0.499422	-1.809088
N	1.580170	-1.666846	0.669332
C	2.283835	-2.911652	-1.334518
C	2.473460	-1.820000	-2.168874
C	2.944746	-0.637680	-4.025308
H	3.210562	-0.304535	-5.020098
C	2.533014	1.618507	-3.037930
C	1.872976	-2.832485	-0.011726
C	2.585531	0.236830	-2.941074
C	2.877802	-1.911635	-3.546598
H	3.076168	-2.839272	-4.067732
C	1.198928	-2.056536	1.937541
C	1.681136	-3.967783	0.848082
H	1.848604	-4.995803	0.553731
C	1.260750	-3.487663	2.053972
H	1.017543	-4.041639	2.951561
O	-0.312443	0.115766	-0.857671
C	-1.928094	1.217167	0.524167
C	-1.465611	0.589718	-0.652918
H	-2.174647	0.527934	-1.493346
C	-3.211968	1.751461	0.619766
H	-3.809821	1.861051	-0.281245
H	0.535265	-1.651882	3.909851
H	2.462296	-3.898907	-1.747519
H	2.785298	2.065071	-3.993908
H	1.117801	4.322276	1.734909
H	6.306324	0.513023	1.461299
H	7.932639	-1.343253	1.039637
H	-1.258868	1.263077	1.374343
H	-3.410038	2.495472	1.386264

^{6}TS1E2C$_{endo\text{-}cis\text{-}p}$

C	-3.831718	-1.377013	-0.310085
C	-4.645942	-0.455868	0.443983
C	-4.121945	0.280248	1.501419
H	-4.791466	0.876461	2.112701
H	-3.218252	-0.042492	2.003504
C	-2.552783	-1.745426	-0.031868
H	-2.083826	-1.450181	0.904384
C	-6.016700	-0.182074	-0.043719
C	-6.779951	-1.206038	-0.636949
C	-6.597591	1.095230	0.079239
C	-8.076897	-0.962910	-1.082629
H	-6.371316	-2.209015	-0.715324
C	-7.888166	1.340226	-0.381435
H	-6.030153	1.911601	0.515812
C	-8.634066	0.311770	-0.962553
H	-8.654367	-1.771315	-1.521272
H	-8.311617	2.336205	-0.290176
H	-9.642474	0.502670	-1.317482
H	-4.258981	-1.743678	-1.240603
C	-1.726035	-2.602170	-0.929758
H	-1.364872	-3.489145	-0.396126
H	-2.278719	-2.920746	-1.818809
H	-0.829185	-2.052688	-1.248011
Fe	1.588403	0.202138	-0.222363

N	1.657244	2.191808	0.296413
N	2.589594	0.609660	-1.968557
O	3.703928	0.183579	0.692927
C	2.601921	3.063046	-1.806530
C	2.077802	3.216051	-0.521584
C	1.467884	4.182788	1.428535
H	1.256183	4.871904	2.236062
C	5.449522	-0.687824	2.028043
C	0.823116	2.042551	2.605602
C	2.848244	1.859952	-2.472639
C	1.295200	2.751978	1.498399
C	1.943654	4.469272	0.180617
H	2.199055	5.437852	-0.229571
C	4.107636	-0.689658	1.459328
H	3.441633	-1.521850	1.742902
C	2.994674	-0.304114	-2.908837
C	3.438160	1.731462	-3.784422
H	3.742882	2.560655	-4.409810
C	3.531314	0.395822	-4.052390
H	3.926450	-0.080830	-4.940240
C	5.834899	-1.669569	2.856897
H	5.162017	-2.482610	3.122593
N	1.851634	-1.830121	-0.547329
N	0.924358	-0.249136	1.697390
C	0.847184	-2.705959	1.521008
C	1.439141	-2.861165	0.264644
C	2.281958	-3.831294	-1.593912
H	2.619988	-4.524636	-2.353365
C	2.896340	-1.690947	-2.777429
C	0.612589	-1.499963	2.186429
C	2.370912	-2.395106	-1.691697
C	1.707944	-4.119053	-0.387330
H	1.485133	-5.093078	0.029517
C	0.638925	0.659811	2.694522
C	0.077854	-1.370008	3.519275
H	-0.249097	-2.195943	4.138290
C	0.101097	-0.039228	3.834805
H	-0.207544	0.431463	4.759653
O	-0.180118	0.256560	-1.190601
C	-1.782761	1.349834	0.195823
C	-1.347937	0.714101	-0.978994
H	-2.054727	0.629684	-1.814692
C	-3.086917	1.803593	0.363787
H	-3.758419	1.848861	-0.488930
H	0.565556	2.625225	3.484833
H	0.566520	-3.614889	2.044693
H	3.262387	-2.285280	-3.609040
H	2.878420	3.973490	-2.329338
H	6.103419	0.133627	1.747454
H	6.827734	-1.696650	3.295330
H	-1.075791	1.443477	1.009396
H	-3.296726	2.525579	1.147515

^{4}TS2E2C$_{endo\text{-}cis\text{-}p}$

C	-3.410720	-1.331021	-0.098624
C	-4.278614	-0.248604	0.297241
C	-3.875347	0.703274	1.225752
H	-4.592709	1.436943	1.578322
H	-3.066245	0.493842	1.914193
C	-2.195882	-1.631820	0.432159
H	-1.841795	-1.119373	1.324246
C	-5.554981	-0.079905	-0.434078
C	-6.275706	-1.204511	-0.879688
C	-6.088039	1.197830	-0.692738
C	-7.487008	-1.056128	-1.551060
H	-5.907131	-2.203215	-0.665470
C	-7.290932	1.344494	-1.377913
H	-5.549534	2.086440	-0.377335
C	-7.996588	0.218144	-1.808274
H	-8.035358	-1.937476	-1.870303
H	-7.677145	2.339412	-1.579334
H	-8.937253	0.333385	-2.338493
H	-3.726219	-1.907865	-0.964924
C	-1.290622	-2.683801	-0.112159
H	-0.996727	-3.391287	0.671771
H	-1.749180	-3.234211	-0.939214
H	-0.360912	-2.220922	-0.471407
Fe	2.049029	0.045830	0.146218
N	1.449738	0.945615	1.836152
N	2.666587	1.820077	-0.543711
C	1.889206	3.299111	1.259627
C	1.412423	2.306598	2.098104
C	0.360889	1.343882	3.839537
H	-0.157559	1.129303	4.765108
C	0.663590	-1.015812	3.099502
C	2.463687	3.065133	0.022842
C	0.811724	0.348044	2.911237
C	0.744759	2.555477	3.342557

H	0.597539	3.537618	3.772735
C	3.245199	2.068203	-1.775005
C	2.920493	4.098213	-0.863240
H	2.878961	5.156904	-0.642904
C	3.395152	3.480927	-1.981483
H	3.826782	3.927855	-2.867475
N	3.113314	-0.855045	-1.293669
N	1.854496	-1.727510	1.068727
C	2.911396	-3.202711	-0.593044
C	3.304743	-2.211397	-1.474681
C	4.150234	-1.252594	-3.324599
H	4.612247	-1.035201	-4.278689
C	3.669970	1.103173	-2.671622
C	2.239142	-2.969352	0.594046
C	3.626529	-0.258583	-2.429413
C	3.959700	-2.462184	-2.728485
H	4.230086	-3.444722	-3.092561
C	1.193712	-1.978648	2.257797
C	1.820049	-4.001540	1.498921
H	2.013244	-5.056701	1.355962
C	1.164109	-3.388797	2.525331
H	0.712954	-3.837237	3.400999
O	0.354262	-0.013253	-0.956040
C	-1.314577	1.384296	0.034581
C	-0.798566	0.520106	-0.948531
H	-1.430446	0.304821	-1.821210
C	-2.594498	1.920099	-0.046327
H	-3.163986	1.825452	-0.966536
H	0.156459	-1.351337	3.997978
H	3.138207	-4.231146	-0.852729
H	4.104787	1.437766	-3.607288
H	1.792038	4.328654	1.587054
H	-0.691709	1.601242	0.892915
H	-2.844949	2.785946	0.559143

^{6}TS2E2C$_{endo\text{-}cis\text{-}p}$

C	-3.368499	-1.312063	-0.304610
C	-4.204349	-0.212860	0.132598
C	-3.742929	0.745610	1.016906
H	-4.422393	1.501632	1.395713
H	-2.855092	0.580907	1.614980
C	-2.142646	-1.641483	0.175338
H	-1.738984	-1.134138	1.049210
C	-5.530681	-0.058514	-0.509451
C	-6.282470	-1.192551	-0.870832
C	-6.078625	1.213495	-0.763404
C	-7.539391	-1.058408	-1.456087
H	-5.899621	-2.186192	-0.657873
C	-7.328465	1.345686	-1.362222
H	-5.516367	2.108010	-0.512267
C	-8.064768	0.210210	-1.709298
H	-8.110393	-1.946131	-1.711446
H	-7.727105	2.335949	-1.562171
H	-9.041143	0.314189	-2.173088
H	-3.738894	-1.883991	-1.152545
C	-1.294084	-2.729663	-0.392717
H	-1.071365	-3.484661	0.370574
H	-1.769612	-3.220034	-1.247475
H	-0.323978	-2.324715	-0.710562
Fe	1.910638	0.039535	0.100162
N	1.021518	0.107984	1.983736
N	2.266067	2.072942	0.308147
C	0.917939	2.546652	2.312002
C	0.600876	1.236297	2.662633
C	-0.236658	-0.512223	3.817640
H	-0.721722	-1.156377	4.539833
C	0.816332	-2.308990	2.415043
C	1.700702	2.934893	1.228572
C	0.541567	-0.974358	2.698674
C	-0.197196	0.853685	3.797099
H	-0.643502	1.546142	4.499321
C	2.959825	2.865579	-0.586914
C	2.034608	4.294417	0.893654
H	1.721289	5.163194	1.458076
C	2.807848	4.251813	-0.229770
H	3.254356	5.078582	-0.766872
N	3.456221	-0.016351	-1.270759
N	2.238290	-1.983935	0.434841
C	3.709702	-2.452737	-1.483589
C	3.963419	-1.143980	-1.882269
C	4.810507	0.600763	-3.033625
H	5.339072	1.245491	-3.723722
C	3.725013	2.397508	-1.650743
C	2.925666	-2.841919	-0.401048
C	3.966918	1.062281	-1.961318
C	4.809085	-0.762633	-2.984095
H	5.336222	-1.456696	-3.625744
C	1.623314	-2.776689	1.382131

C	2.727850	-4.201902	0.027570
H	3.162937	-5.068860	-0.452485
C	1.925800	-4.161460	1.131378
H	1.572817	-4.989129	1.733068
O	0.419017	-0.045518	-1.181121
C	-1.261037	1.451236	-0.399146
C	-0.747529	0.467807	-1.259957
H	-1.359682	0.131076	-2.104160
C	-2.552873	1.933774	-0.510670
H	-3.164089	1.686987	-1.373196
H	0.388146	-3.051882	3.080883
H	4.184603	-3.243080	-2.056281
H	4.206464	3.143319	-2.275338
H	0.532880	3.335426	2.950735
H	-0.625394	1.797033	0.406931
H	-2.841455	2.834076	0.022061

^{4}TS1E2C$_{exo\text{-}trans\text{-}p}$

C	4.089059	-1.384029	0.127405
C	4.927191	-0.253076	0.414218
C	4.409837	0.897190	1.009553
H	3.530439	0.837312	1.642347
H	5.080782	1.719891	1.238336
C	2.768732	-1.485698	0.438020
H	2.311604	-0.767716	1.114487
C	6.301518	-0.226989	-0.132529
C	6.595306	-0.779210	-1.394343
C	7.346914	0.374068	0.594344
C	7.887324	-0.724509	-1.912001
H	5.802367	-1.221868	-1.989870
C	8.640576	0.415787	0.080932
H	7.148461	0.775722	1.583793
C	8.914630	-0.130740	-1.175228
H	8.091821	-1.142033	-2.893628
H	9.437259	0.868251	0.664170
H	9.923224	-0.095958	-1.576614
H	4.531078	-2.187025	-0.458173
C	1.862464	-2.563002	-0.048166
H	1.008497	-2.102069	-0.565755
H	2.366149	-3.254899	-0.729698
H	1.439441	-3.131507	0.788753
Fe	-1.731540	0.179372	0.008976
N	-1.941929	2.158638	-0.245561
N	-0.966787	0.505850	1.839647
O	-3.806837	0.226670	1.007460
C	-0.986209	2.965466	1.871759
C	-1.570611	3.157961	0.628845
C	-2.426715	4.223155	-1.159910
H	-2.782692	4.947613	-1.880810
C	-6.019747	-0.548577	1.322076
C	-2.972908	2.173673	-2.478097
C	-0.709226	1.726157	2.430496
C	-2.470365	2.797713	-1.346679
C	-1.868549	4.446399	0.063059
H	-1.673809	5.391762	0.552658
C	-4.702853	-0.556891	0.697579
H	-4.524342	-1.311090	-0.088545
C	-0.568268	-0.447763	2.753640
C	-0.113887	1.532773	3.724565
H	0.181260	2.333420	4.390407
C	-0.026737	0.186471	3.924876
H	0.354294	-0.342609	4.788824
C	-6.956952	-1.428548	0.939132
H	-6.759745	-2.162986	0.160558
N	-1.678105	-1.802808	0.339936
N	-2.613912	-0.152406	-1.762306
C	-2.571885	-2.610680	-1.805626
C	-2.047357	-2.804184	-0.536018
C	-1.323213	-3.867209	1.312125
H	-1.035232	-4.592428	2.062323
C	-0.693422	-1.819638	2.594657
C	-2.831791	-1.371091	-2.371080
C	-1.221818	-2.442553	1.474263
C	-1.826783	-4.091326	0.064604
H	-2.038334	-5.037621	-0.416196
C	-3.027063	0.801557	-2.668864
C	-3.400619	-1.176469	-3.677338
H	-3.666050	-1.976158	-4.356639
C	-3.524270	0.168616	-3.860285
H	-3.911186	0.698729	-4.720823
O	0.150541	0.177267	-0.913782
C	2.382243	0.943611	-1.116966
C	1.092541	0.938597	-0.553797
H	0.903900	1.646870	0.267178
C	3.377491	1.787895	-0.615218
H	3.077376	2.623136	0.014617
H	-3.346497	2.806387	-3.276249
H	-2.804023	-3.491937	-2.394282

H	-0.355623	-2.452405	3.408763
H	-0.738988	3.846955	2.453879
H	-6.193672	0.193981	2.096380
H	-7.946541	-1.446564	1.385296
H	2.587230	0.252361	-1.927298
H	4.269663	1.964948	-1.207036

^{6}**TS1E2C**$_{exo\text{-}trans\text{-}p}$

C	4.052269	-1.402219	0.027646
C	4.876497	-0.271938	0.376566
C	4.353183	0.839311	1.025288
H	3.443869	0.763663	1.612294
H	5.011192	1.659944	1.293904
C	2.733304	-1.541166	0.316787
H	2.247478	-0.848125	0.999870
C	6.259351	-0.217352	-0.147726
C	6.570910	-0.707642	-1.430504
C	7.293104	0.348376	0.622572
C	7.869776	-0.626176	-1.927213
H	5.787169	-1.123743	-2.056707
C	8.593671	0.415796	0.129715
H	7.080289	0.700686	1.627702
C	8.885735	-0.068335	-1.147835
H	8.088615	-0.995699	-2.924805
H	9.381723	0.839199	0.745532
H	9.899853	-0.013579	-1.532663
H	4.517540	-2.174416	-0.580847
C	1.858751	-2.621903	-0.219197
H	1.000117	-2.166415	-0.732840
H	2.388274	-3.277962	-0.916336
H	1.438632	-3.228113	0.592023
Fe	-1.675508	0.174864	-0.035793
N	-1.945742	2.211346	-0.209773
N	-0.865893	0.464312	1.848960
O	-3.674076	0.234900	1.056749
C	-0.914193	2.926804	1.906780
C	-1.544582	3.175168	0.684866
C	-2.483511	4.282034	-1.047441
H	-2.872861	5.029009	-1.727210
C	-5.863010	-0.518398	1.532043
C	-3.082672	2.231798	-2.393172
C	-0.605930	1.675619	2.448867
C	-2.528639	2.857566	-1.273865
C	-1.876203	4.478098	0.160391
H	-1.672944	5.416553	0.660128
C	-4.581818	-0.568439	0.841428
H	-4.440549	-1.369165	0.096334
C	-0.451628	-0.519029	2.717180
C	0.024392	1.447543	3.725967
H	0.336205	2.226310	4.410241
C	0.119569	0.093699	3.891674
H	0.523959	-0.447406	4.737613
C	-6.816494	-1.421027	1.256298
H	-6.658576	-2.204879	0.518044
N	-1.665903	-1.866252	0.287867
N	-2.718959	-0.127824	-1.781335
C	-2.687208	-2.587751	-1.835374
C	-2.092778	-2.834277	-0.594808
C	-1.286795	-3.939239	1.204962
H	-0.974300	-4.687636	1.922174
C	-0.593358	-1.892900	2.506375
C	-2.976275	-1.336874	-2.386373
C	-1.164486	-2.514991	1.394206
C	-1.852686	-4.136251	-0.023821
H	-2.094009	-5.076217	-0.503463
C	-3.164813	0.858504	-2.632641
C	-3.611118	-1.105082	-3.660507
H	-3.923422	-1.881871	-4.346676
C	-3.728746	0.247912	-3.811212
H	-4.155434	0.790989	-4.644668
O	0.131228	0.218126	-0.973880
C	2.359805	0.991199	-1.182022
C	1.077823	0.981415	-0.610659
H	0.889811	1.678221	0.217376
C	3.354023	1.813679	-0.654295
H	3.067467	2.625635	0.010080
H	-3.493990	2.882491	-3.158744
H	-2.960260	-3.456591	-2.426314
H	-0.230501	-2.545468	3.294711
H	-0.653105	3.794254	2.505237
H	-5.998124	0.272911	2.264614
H	-7.781189	-1.409108	1.754198
H	2.565042	0.309242	-1.999913
H	4.272169	1.974366	-1.208940

^{4}**TS2E2O**$_{cc\text{-}endo\text{-}cis\text{-}p}$

C	-2.418755	1.454392	0.176758
C	-2.918622	0.113443	0.341391

C	-2.076578	-0.821794	0.996812
H	-1.393610	-0.417614	1.739758
H	-2.517826	-1.776957	1.269547
C	-3.192634	2.537875	-0.075471
H	-4.271881	2.409015	-0.145858
C	-4.163764	-0.331596	-0.281713
C	-4.555543	0.150966	-1.551373
C	-4.977223	-1.295958	0.353166
C	-5.717461	-0.314626	-2.158042
H	-3.914636	0.851640	-2.075429
C	-6.149694	-1.739309	-0.246721
H	-4.709430	-1.658803	1.340294
C	-6.520452	-1.253816	-1.505168
H	-5.994379	0.048578	-3.143070
H	-6.777879	-2.462284	0.264581
H	-7.431404	-1.610440	-1.976879
H	-1.349523	1.582603	0.333454
C	-2.680666	3.929811	-0.217571
H	-3.130866	4.583205	0.542483
H	-1.592557	3.984971	-0.133054
H	-2.984406	4.343668	-1.189205
Fe	1.661154	0.105993	0.208744
N	2.368659	-1.776595	0.131116
N	1.189738	-0.190801	2.143462
C	1.636056	-2.604614	2.326825
C	2.208505	-2.771555	1.076765
C	3.170490	-3.788871	-0.683702
H	3.625291	-4.485504	-1.375791
C	3.302326	-1.772000	-2.143204
C	1.169180	-1.396778	2.820959
C	2.956242	-2.394328	-0.956129
C	2.707925	-4.022782	0.575973
H	2.704871	-4.951021	1.132458
C	0.651621	0.734131	3.022232
C	0.600145	-1.225360	4.127868
H	0.481248	-2.019312	4.853700
C	0.279907	0.094319	4.252732
H	-0.155870	0.604794	5.101786
N	1.419994	2.087593	0.489980
N	2.586246	0.498616	-1.528131
C	2.185980	2.917994	-1.694472
C	1.641537	3.091662	-0.432641
C	0.768703	4.128738	1.364002
H	0.388394	4.840956	2.084873
C	0.498535	2.087262	2.770866
C	2.627217	1.706129	-2.196328
C	0.882088	2.715968	1.597824
C	1.238961	4.361529	0.104833
H	1.325137	5.304519	-0.419412
C	3.133111	-0.421876	-2.399360
C	3.213639	1.538592	-3.498391
H	3.365727	2.339863	-4.209726
C	3.526193	0.219602	-3.624584
H	3.988267	-0.288197	-4.461082
O	-0.080640	-0.287431	-0.578207
C	-1.555982	-1.960058	-1.402342
C	-0.823143	-1.333796	-0.267882
H	-0.352948	-2.076363	0.392474
C	-1.788161	-3.271767	-1.482084
H	-1.460248	-3.957511	-0.702950
H	3.752837	-2.380723	-2.919867
H	2.293166	3.794995	-2.323984
H	0.073285	2.701442	3.557589
H	1.564885	-3.474681	2.970972
H	-1.876452	-1.276727	-2.185259
H	-2.304612	-3.711349	-2.330044

^{6}TS2E2O$_{cc\text{-}endo\text{-}cis\text{-}p}$

C	-2.420207	1.482195	0.217049
C	-2.965014	0.152429	0.351176
C	-2.195721	-0.808570	1.034381
H	-1.469071	-0.453311	1.759789
H	-2.657008	-1.762974	1.270358
C	-3.143487	2.583369	-0.097109
H	-4.216519	2.482002	-0.253129
C	-4.199512	-0.251220	-0.328345
C	-4.499928	0.218542	-1.626063
C	-5.083314	-1.168585	0.278558
C	-5.643338	-0.216245	-2.289227
H	-3.805791	0.886650	-2.124637
C	-6.237036	-1.581186	-0.378769
H	-4.883914	-1.519967	1.286088
C	-6.517396	-1.110500	-1.665466
H	-5.850940	0.137469	-3.294597
H	-6.920452	-2.268459	0.110556
H	-7.413822	-1.442441	-2.180961
H	-1.364363	1.592038	0.453793
C	-2.585801	3.961031	-0.205696

H	-3.076647	4.630404	0.514177
H	-1.506211	3.990499	-0.038210
H	-2.802355	4.376127	-1.199833
Fe	1.540457	0.058288	0.148665
N	2.382008	-1.842114	0.136558
N	1.128627	-0.244599	2.170094
C	1.542881	-2.669237	2.294690
C	2.167955	-2.842000	1.062030
C	3.227269	-3.832993	-0.665698
H	3.720473	-4.523449	-1.337594
C	3.446626	-1.767196	-2.081160
C	1.075193	-1.463868	2.815767
C	3.031858	-2.429838	-0.929133
C	2.692172	-4.088299	0.563024
H	2.661549	-5.028947	1.097678
C	0.600062	0.684131	3.046754
C	0.480989	-1.299711	4.116872
H	0.328416	-2.098850	4.830847
C	0.189706	0.026940	4.260426
H	-0.249767	0.526545	5.114205
N	1.442703	2.102835	0.530786
N	2.700316	0.505751	-1.506502
C	2.334807	2.935249	-1.607749
C	1.731767	3.112113	-0.364232
C	0.840343	4.135551	1.439448
H	0.468434	4.848275	2.164394
C	0.489159	2.049745	2.799114
C	2.795024	1.730909	-2.131890
C	0.903328	2.711617	1.645727
C	1.350380	4.382741	0.196218
H	1.479658	5.338110	-0.296015
C	3.302822	-0.407274	-2.344901
C	3.468355	1.581219	-3.397407
H	3.678191	2.391151	-4.083985
C	3.782178	0.260486	-3.528892
H	4.300523	-0.226875	-4.344481
O	-0.159199	-0.357329	-0.635713
C	-1.692050	-1.994885	-1.420274
C	-0.906176	-1.392865	-0.322878
H	-0.468135	-2.119776	0.373096
C	-2.023593	-3.289058	-1.444570
H	-1.740177	-3.967103	-0.641891
H	3.947898	-2.363530	-2.837072
H	2.491890	3.825407	-2.208877
H	0.066746	2.659721	3.591574
H	1.439327	-3.548982	2.922315
H	-1.971510	-1.319163	-2.224209
H	-2.582092	-3.718940	-2.270153

^{4}TS2E2O$_{co\text{-}endo\text{-}cis\text{-}p}$

C	2.389592	-2.090705	-0.611552
C	3.073446	-1.073641	0.063054
C	2.317026	-0.192653	0.974494
H	1.700106	-0.733925	1.697518
H	2.956306	0.511741	1.505361
C	1.060592	-2.391766	-0.376896
H	0.590663	-2.115665	0.556808
C	4.451996	-0.741413	-0.295755
C	4.958582	-0.994000	-1.591564
C	5.319813	-0.165998	0.659124
C	6.273095	-0.681905	-1.915064
H	4.304831	-1.386512	-2.363158
C	6.642050	0.120502	0.338766
H	4.970753	0.013648	1.670448
C	7.121480	-0.130623	-0.949606
H	6.637448	-0.861530	-2.921884
H	7.300250	0.540353	1.093150
H	8.150693	0.106384	-1.202404
H	2.888896	-2.614691	-1.422110
C	0.249325	-3.217296	-1.301203
H	-0.508826	-2.563577	-1.753178
H	0.843928	-3.664499	-2.102403
H	-0.304757	-3.991249	-0.758086
Fe	-1.595583	0.208976	0.199673
N	-1.519773	2.217716	0.164759
N	-1.217082	0.264889	2.173549
C	-0.875700	2.678924	2.492209
C	-1.169488	3.068546	1.194910
C	-1.336712	4.384601	-0.622930
H	-1.356487	5.204767	-1.328592
C	-1.984807	2.580701	-2.220378
C	-0.873972	1.364998	2.935490
C	-1.620212	3.015979	-0.957205
C	-1.072450	4.420308	0.713358
H	-0.827416	5.275170	1.330281
C	-1.045683	-0.828584	3.001496
C	-0.477391	0.953058	4.253889
H	-0.175104	1.629283	5.042978

C	-0.567223	-0.406947	4.289603
H	-0.362134	-1.074107	5.116798
N	-2.235132	-1.689146	0.418004
N	-2.447353	0.244698	-1.613598
C	-3.278147	-2.062510	-1.776173
C	-2.910511	-2.467997	-0.502831
C	-2.507479	-3.834501	1.240115
H	-2.448131	-4.663505	1.933512
C	-1.371988	-2.136902	2.678060
C	-3.027211	-0.804093	-2.298624
C	-1.988699	-2.519160	1.496178
C	-3.097521	-3.796233	0.010345
H	-3.611008	-4.590788	-0.515430
C	-2.398351	1.291490	-2.512340
C	-3.336305	-0.409853	-3.646766
H	-3.806676	-1.051393	-4.380548
C	-2.932117	0.883404	-3.783699
H	-3.006465	1.525084	-4.651913
O	0.242286	-0.099927	-0.366645
C	1.932759	1.463942	-0.987524
C	1.264628	0.692275	0.119861
H	0.904837	1.393258	0.882236
C	2.094615	2.785897	-0.965928
H	1.749881	3.388515	-0.127912
H	-2.009723	3.313234	-3.020000
H	-3.775343	-2.785845	-2.413759
H	-1.212540	-2.898732	3.433971
H	-0.597542	3.451492	3.201232
H	2.256217	0.875246	-1.844250
H	2.567408	3.319720	-1.785470

^{6}TS2E2O$_{\text{co-endo-cis-p}}$

C	2.458039	-1.841508	-0.852320
C	3.178785	-0.891904	-0.154393
C	2.423641	-0.024165	0.793891
H	1.889197	-0.595886	1.561319
H	3.062230	0.706856	1.289798
C	1.100558	-2.095236	-0.591965
H	0.736081	-2.063752	0.426988
C	4.570062	-0.580544	-0.493489
C	5.081923	-0.803696	-1.790567
C	5.441496	-0.064499	0.489196
C	6.410672	-0.525270	-2.088630
H	4.424797	-1.151416	-2.581073
C	6.776420	0.191644	0.193153
H	5.084389	0.096246	1.501367
C	7.264177	-0.032829	-1.096661
H	6.781236	-0.683926	-3.096770
H	7.437153	0.567941	0.968128
H	8.302921	0.181036	-1.330236
H	2.904770	-2.340350	-1.708242
C	0.244427	-2.807600	-1.577700
H	-0.566000	-2.140344	-1.890174
H	0.802193	-3.108399	-2.468541
H	-0.233365	-3.679931	-1.117753
Fe	-1.478293	0.110009	0.201503
N	-1.630357	2.176161	0.374449
N	-1.028210	0.074013	2.240519
C	-0.711288	2.480556	2.637681
C	-1.178726	2.960389	1.416163
C	-1.689719	4.396875	-0.247707
H	-1.862007	5.268724	-0.865432
C	-2.493884	2.662043	-1.878370
C	-0.631709	1.141877	3.017541
C	-1.952957	3.039753	-0.652122
C	-1.219586	4.349129	1.032919
H	-0.930133	5.174115	1.671099
C	-0.783602	-1.062205	2.984073
C	-0.111667	0.663356	4.273517
H	0.256715	1.296497	5.070417
C	-0.196615	-0.698969	4.249319
H	0.084950	-1.400758	5.023860
N	-2.163244	-1.844128	0.425689
N	-2.701316	0.246360	-1.467069
C	-3.395939	-2.101224	-1.690141
C	-2.878011	-2.587816	-0.492422
C	-2.276995	-4.039197	1.128664
H	-2.133190	-4.908127	1.757983
C	-1.116388	-2.358460	2.595449
C	-3.306174	-0.789376	-2.147540
C	-1.800193	-2.715428	1.434498
C	-2.955250	-3.956735	-0.054775
H	-3.468968	-4.746753	-0.587379
C	-2.868544	1.372374	-2.245487
C	-3.844687	-0.305717	-3.394138
H	-4.376499	-0.914484	-4.113882
C	-3.566304	1.027904	-3.458688
H	-3.827178	1.729379	-4.240492

O	0.316653	-0.103099	-0.477920
C	1.858305	1.603179	-1.135971
C	1.314045	0.779769	-0.000573
H	0.888438	1.447035	0.754367
C	1.760730	2.931276	-1.177131
H	1.278090	3.492909	-0.379789
H	-2.695906	3.454955	-2.591551
H	-3.919244	-2.809976	-2.324251
H	-0.883253	-3.156538	3.293567
H	-0.371887	3.217844	3.358534
H	2.317178	1.050504	-1.953567
H	2.151343	3.507835	-2.010771

^{4}TS2E2C$_{cc1\text{-}endo\text{-}cis\text{-}p}$

C	3.032342	0.668843	0.674150
C	4.010710	-0.367537	0.433695
C	3.673550	-1.656971	0.878900
H	4.437276	-2.429728	0.872316
H	2.963961	-1.741450	1.697242
C	2.726280	1.647558	-0.216237
H	3.312300	1.721735	-1.130652
C	5.196035	-0.127595	-0.398320
C	5.820525	1.138880	-0.402486
C	5.770093	-1.155055	-1.178402
C	6.961661	1.369953	-1.165790
H	5.428103	1.928623	0.229091
C	6.901483	-0.915956	-1.950093
H	5.318706	-2.141623	-1.199103
C	7.502048	0.347241	-1.947649
H	7.435015	2.346955	-1.142271
H	7.318044	-1.715304	-2.555689
H	8.390661	0.528385	-2.544959
H	2.385397	0.547454	1.541602
C	1.581966	2.588948	-0.073888
H	0.727829	2.213865	-0.659824
H	1.249285	2.677870	0.962305
H	1.833991	3.581785	-0.462107
Fe	-1.941750	0.025651	0.189399
N	-1.389107	-1.223911	1.661366
N	-2.847596	-1.507972	-0.728686
C	-2.191516	-3.363284	0.744024
C	-1.529692	-2.602016	1.692081
C	-0.251863	-2.101565	3.475825
H	0.342677	-2.119259	4.380174
C	-0.278821	0.371459	3.170197
C	-2.794998	-2.846650	-0.389220
C	-0.613256	-0.909561	2.763576
C	-0.831445	-3.148410	2.819973
H	-0.799750	-4.201067	3.069552
C	-3.517130	-1.461991	-1.936752
C	-3.441417	-3.643400	-1.394356
H	-3.540456	-4.720234	-1.352682
C	-3.879480	-2.786353	-2.358466
H	-4.415814	-3.013465	-3.270518
N	-2.949347	1.293102	-0.996051
N	-1.472177	1.572683	1.386762
C	-2.399271	3.435873	0.074593
C	-2.962169	2.672599	-0.933417
C	-4.020234	2.166758	-2.852265
H	-4.555285	2.179462	-3.792788
C	-3.842938	-0.309587	-2.631056
C	-1.711890	2.915206	1.157792
C	-3.595198	0.973270	-2.173900
C	-3.636454	3.219713	-2.078579
H	-3.788178	4.276904	-2.253111
C	-0.727309	1.525676	2.550426
C	-1.120138	3.712209	2.195437
H	-1.180831	4.791400	2.249026
C	-0.502379	2.851928	3.054291
H	0.044185	3.079467	3.960365
O	-0.328257	0.066858	-0.989969
C	1.156722	-1.721024	-0.409075
C	0.759204	-0.594572	-1.123999
H	1.428125	-0.234144	-1.918317
C	2.437464	-2.290356	-0.565878
H	3.011424	-2.014815	-1.448612
H	0.314144	0.478145	4.072582
H	-2.498482	4.514103	0.009107
H	-4.367412	-0.418498	-3.574249
H	-2.224107	-4.437620	0.890376
H	0.490939	-2.111297	0.349683
H	2.562502	-3.332115	-0.277669

^{6}TS2E2C$_{cc1\text{-}endo\text{-}cis\text{-}p}$

C	2.989043	0.677758	0.522785
C	3.985189	-0.357070	0.338737
C	3.658528	-1.632602	0.807739
H	4.418719	-2.407697	0.829716

H	2.884682	-1.728162	1.563797
C	2.720297	1.646758	-0.388681
H	3.338955	1.707917	-1.282611
C	5.204883	-0.116861	-0.446936
C	5.821328	1.152726	-0.435979
C	5.817321	-1.148574	-1.190138
C	6.995382	1.382081	-1.148745
H	5.396074	1.947219	0.167770
C	6.982144	-0.911814	-1.911505
H	5.371322	-2.137510	-1.220856
C	7.576051	0.354349	-1.894036
H	7.461999	2.361966	-1.114288
H	7.429467	-1.714863	-2.489592
H	8.489986	0.534271	-2.452194
H	2.315095	0.570481	1.371510
C	1.584395	2.605703	-0.290010
H	0.745296	2.250034	-0.907396
H	1.218570	2.701935	0.733707
H	1.868586	3.593770	-0.668363
Fe	-1.819016	0.021028	0.109570
N	-1.277659	-1.319735	1.609472
N	-3.023284	-1.479201	-0.666396
C	-2.217393	-3.388657	0.656116
C	-1.442384	-2.692439	1.579835
C	-0.029481	-2.286509	3.292671
H	0.623197	-2.358472	4.153474
C	-0.051567	0.217710	3.091082
C	-2.954816	-2.827265	-0.381476
C	-0.426681	-1.054157	2.664644
C	-0.663188	-3.298004	2.626965
H	-0.623144	-4.360531	2.829936
C	-3.860365	-1.355962	-1.756133
C	-3.761008	-3.566424	-1.318890
H	-3.885334	-4.641561	-1.311929
C	-4.316817	-2.657733	-2.171037
H	-4.988134	-2.840166	-3.000125
N	-3.060988	1.388720	-0.832618
N	-1.312203	1.542502	1.446018
C	-2.318765	3.457306	0.270081
C	-3.021649	2.761528	-0.708581
C	-4.362618	2.354130	-2.475608
H	-5.029592	2.422878	-3.325179
C	-4.239289	-0.152420	-2.344003
C	-1.544460	2.892588	1.280045
C	-3.884762	1.120147	-1.905020
C	-3.832840	3.368274	-1.733449
H	-3.978452	4.433916	-1.854372
C	-0.499381	1.421860	2.554227
C	-0.858702	3.635186	2.306014
H	-0.886236	4.712172	2.410512
C	-0.207374	2.726635	3.090543
H	0.400619	2.912551	3.966650
O	-0.335040	0.021528	-1.148249
C	1.171743	-1.757312	-0.621083
C	0.771772	-0.614748	-1.304897
H	1.429254	-0.214791	-2.085953
C	2.458739	-2.296975	-0.768640
H	3.070928	-1.969829	-1.605680
H	0.598329	0.275218	3.959047
H	-2.396458	4.539931	0.256476
H	-4.904909	-0.210586	-3.199411
H	-2.253549	-4.468699	0.758684
H	0.495263	-2.182382	0.109901
H	2.623489	-3.329222	-0.471217

^{6}TS2E2C$_{cc2\text{-}endo\text{-}cis\text{-}p}$

C	-3.581674	-1.170167	-0.193983
C	-4.441577	-0.123656	0.088230
C	-3.900949	1.027430	0.869465
H	-4.671501	1.766718	1.090535
H	-3.494634	0.700795	1.833672
C	-2.295556	-1.297982	0.369445
H	-2.123155	-0.921671	1.372730
C	-5.766540	-0.050598	-0.535348
C	-6.471467	-1.232844	-0.849898
C	-6.369437	1.186825	-0.844680
C	-7.724654	-1.177950	-1.449274
H	-6.050137	-2.197146	-0.582617
C	-7.615153	1.237737	-1.462890
H	-5.849956	2.116361	-0.634383
C	-8.299063	0.057305	-1.763805
H	-8.258976	-2.098186	-1.665531
H	-8.054324	2.199794	-1.709178
H	-9.276612	0.099278	-2.234866
H	-3.845392	-1.871731	-0.981388
C	-1.341413	-2.350381	-0.083387
H	-1.238693	-3.115284	0.697406
H	-1.663368	-2.824065	-1.014891

H	-0.337059	-1.940036	-0.232953
Fe	1.992224	0.032928	0.060141
N	1.409914	1.047947	1.783657
N	2.888487	1.795111	-0.561841
C	1.867186	3.352618	1.041768
C	1.321719	2.420244	1.920686
C	0.246345	1.562003	3.709720
H	-0.292347	1.410830	4.636527
C	0.618068	-0.855077	3.132871
C	2.610418	3.064064	-0.098234
C	0.762706	0.506372	2.879319
C	0.590860	2.743848	3.116706
H	0.389165	3.749403	3.463015
C	3.663729	1.956324	-1.691212
C	3.219550	4.043834	-0.961130
H	3.159962	5.114884	-0.817069
C	3.871535	3.359277	-1.944492
H	4.451828	3.757657	-2.766632
N	3.316462	-0.984234	-1.167273
N	1.815331	-1.726500	1.167847
C	2.890097	-3.283845	-0.407551
C	3.443302	-2.352796	-1.281105
C	4.617247	-1.493851	-3.004333
H	5.233644	-1.337017	-3.879985
C	4.182991	0.920220	-2.461998
C	2.151644	-2.994785	0.736950
C	4.031981	-0.441020	-2.213795
C	4.252880	-2.675182	-2.428593
H	4.511170	-3.679503	-2.738443
C	1.123349	-1.890335	2.351170
C	1.644649	-3.974304	1.661856
H	1.778841	-5.043735	1.562401
C	1.009899	-3.291877	2.660259
H	0.524024	-3.692588	3.540788
O	0.414645	0.027835	-1.081236
C	-1.459056	0.925049	0.081524
C	-0.774144	0.523729	-1.053927
H	-1.283423	0.572951	-2.024331
C	-2.727314	1.704664	0.055328
H	-3.066155	1.887243	-0.970359
H	0.092667	-1.134003	4.041228
H	3.077225	-4.331567	-0.620915
H	4.778692	1.199911	-3.325168
H	1.722975	4.400769	1.284531
H	-0.906142	0.912248	1.014456
H	-2.547618	2.683253	0.516959

^{4}TS1E2C$_{endo\text{-}cis\text{-}m}$

C	3.990973	0.217147	-0.427016
C	4.814745	1.334937	-0.168346
C	4.429369	2.667513	-0.368243
C	2.754458	0.395090	-1.019798
H	2.041611	-0.417497	-1.063204
Fe	-1.385775	0.136101	0.123640
N	-1.394459	2.034902	-0.513744
N	-2.396477	0.679251	1.760902
O	-3.421766	-0.066891	-0.928024
C	-2.407977	3.116443	1.448633
C	-1.824153	3.140093	0.190989
C	-1.079356	3.941489	-1.777169
H	-0.799414	4.556497	-2.622763
C	-4.996619	-1.200970	-2.280633
C	-0.382676	1.742715	-2.737657
C	-2.692966	1.962982	2.162330
C	-0.940250	2.511306	-1.727213
C	-1.616073	4.331465	-0.585257
H	-1.869785	5.330683	-0.255868
C	-3.709829	-1.046393	-1.613966
H	-2.974743	-1.859640	-1.744661
C	-2.889593	-0.148880	2.745346
C	-3.380969	1.939978	3.425979
H	-3.722878	2.820212	3.954633
C	-3.512048	0.631616	3.781869
H	-3.979400	0.216708	4.665399
C	-5.252748	-2.293936	-3.014985
H	-4.513993	-3.084726	-3.130749
N	-1.538516	-1.797061	0.664465
N	-0.512631	-0.433825	-1.596880
C	-0.257593	-2.842972	-1.153869
C	-0.950412	-2.880275	0.046706
C	-1.935375	-3.711917	1.892337
H	-2.310664	-4.336001	2.692904
C	-2.784803	-1.531017	2.766440
C	-0.081931	-1.704950	-1.928847
C	-2.131150	-2.289166	1.806714
C	-1.194054	-4.076265	0.808474
H	-0.839758	-5.061539	0.534446
C	-0.173604	0.372871	-2.665382

C	0.547331	-1.688285	-3.219864
H	0.969610	-2.556930	-3.708546
C	0.480314	-0.404241	-3.680341
H	0.842414	-0.007771	-4.620126
O	0.418366	0.199952	1.188512
C	1.793028	2.095575	0.649762
C	1.484759	0.853151	1.276448
H	2.258800	0.441036	1.944484
C	2.920444	2.851524	1.015851
H	3.470956	2.559919	1.906651
H	-0.067514	2.252408	-3.642243
H	0.148596	-3.775744	-1.530629
H	-3.214147	-2.054653	3.613983
H	-2.698262	4.065262	1.887160
H	-5.718917	-0.399441	-2.150278
H	-6.201840	-2.437269	-3.522304
H	1.061012	2.505506	-0.036314
H	2.863993	3.926785	0.869996
C	4.340866	-1.120712	0.125178
C	4.040529	-2.282945	-0.604432
C	4.940338	-1.258277	1.387887
C	4.333198	-3.544038	-0.088324
H	3.594956	-2.190893	-1.591332
C	5.232799	-2.519922	1.904620
H	5.158102	-0.375041	1.982640
C	4.931147	-3.666774	1.167803
H	4.105925	-4.431903	-0.671974
H	5.690580	-2.607129	2.885856
H	5.162976	-4.649194	1.568877
H	5.730777	1.163336	0.394927
H	2.519606	1.255152	-1.632639
C	5.408957	3.784298	-0.082451
H	5.981160	3.592564	0.831526
H	6.122950	3.880904	-0.909696
H	4.903838	4.748998	0.026285
H	3.740934	2.873875	-1.185623

^{4}TS1E2C$_{endo\text{-}trans\text{-}m}$

C	4.021892	0.511578	-0.262652
C	4.643533	1.746254	0.029384
C	4.098466	3.042229	-0.072811
H	4.759028	3.793483	0.362358
C	2.753992	0.453944	-0.805959
H	2.209604	-0.482600	-0.822613
Fe	-1.385957	0.039223	0.131137
N	-1.363603	1.916573	-0.572222
N	-2.504835	0.631561	1.678956
O	-3.358608	-0.205173	-1.045497
C	-2.506849	3.056851	1.283130
C	-1.842579	3.042495	0.065937
C	-0.953790	3.783776	-1.866268
H	-0.613004	4.372777	-2.708065
C	-4.838265	-1.395616	-2.457087
C	-0.206745	1.555215	-2.714534
C	-2.831163	1.926266	2.016735
C	-0.823443	2.355514	-1.764099
C	-1.575922	4.209752	-0.728900
H	-1.851290	5.218555	-0.449876
C	-3.597029	-1.208026	-1.716058
H	-2.850315	-2.019189	-1.773921
C	-3.059142	-0.165985	2.655967
C	-3.598162	1.941898	3.234266
H	-3.976080	2.837406	3.710149
C	-3.747862	0.645468	3.624717
H	-4.269987	0.257773	4.489740
C	-5.040518	-2.515378	-3.167413
H	-4.290663	-3.303160	-3.208208
N	-1.583141	-1.877611	0.716562
N	-0.412321	-0.583871	-1.517508
C	-0.183150	-2.975683	-0.979127
C	-0.958015	-2.978280	0.171123
C	-2.062327	-3.754738	1.972844
H	-2.489933	-4.354342	2.765873
C	-2.957952	-1.546889	2.726449
C	0.046805	-1.861592	-1.774580
C	-2.248186	-2.334647	1.832618
C	-1.254004	-4.151587	0.950145
H	-0.885534	-5.144887	0.728802
C	-0.007352	0.187867	-2.587555
C	0.762090	-1.884754	-3.020429
H	1.220943	-2.766456	-3.449047
C	0.717654	-0.618432	-3.529651
H	1.139318	-0.251411	-4.456487
O	0.356305	0.116536	1.270472
C	1.602697	2.143881	0.975743
C	1.385014	0.819916	1.444234
H	2.172548	0.386654	2.081705
C	2.700362	2.921052	1.386049

H	3.301534	2.558674	2.215894
H	0.166773	2.037004	-3.612268
H	0.248208	-3.918757	-1.297863
H	-3.440777	-2.043960	3.561139
H	-2.828353	4.018006	1.670012
H	-5.572443	-0.596280	-2.400462
H	-5.954647	-2.684625	-3.728131
H	0.839922	2.577971	0.341410
H	2.565764	4.000732	1.388762
C	4.640287	-0.755864	0.223798
C	4.577208	-1.911345	-0.572180
C	5.259977	-0.840783	1.481241
C	5.120112	-3.115132	-0.125980
H	4.116548	-1.855472	-1.555157
C	5.801465	-2.045322	1.928564
H	5.298417	0.032262	2.127578
C	5.734599	-3.185817	1.125905
H	5.073268	-3.996015	-0.760196
H	6.270001	-2.094272	2.907355
H	6.160562	-4.122628	1.473218
C	3.313806	3.621684	-1.241645
H	3.917343	3.553828	-2.154276
H	2.357358	3.134507	-1.434780
H	3.107268	4.680256	-1.056132
H	5.561115	1.679531	0.610705
H	2.313110	1.258753	-1.368608

^{4}TS1E2C$_{exo\text{-}cis\text{-}m}$

C	4.205970	0.669187	0.203789
C	4.633989	2.010873	0.074936
C	3.835232	3.170700	0.122364
H	4.396593	4.069818	-0.132559
C	2.892756	0.365617	0.498875
H	2.236103	1.039086	1.022043
Fe	-1.581270	-0.019368	-0.133713
N	-2.885738	0.439077	-1.578136
N	-1.774880	1.827890	0.630850
O	-3.385263	-0.597154	1.157361
C	-3.290981	2.804111	-1.041107
C	-3.472680	1.664598	-1.808712
C	-4.249506	0.320275	-3.438304
H	-4.750578	-0.112741	-4.294203
C	-4.571589	-2.023337	2.624337
C	-3.007194	-1.727840	-2.732232
C	-2.505308	2.871082	0.099856
C	-3.351461	-0.394921	-2.570807
C	-4.321220	1.596841	-2.968782
H	-4.895079	2.427499	-3.358506
C	-3.421724	-1.634222	1.818087
H	-2.554738	-2.317717	1.815210
C	-1.173406	2.327070	1.768289
C	-2.347198	4.047336	0.910632
H	-2.818755	5.000222	0.708306
C	-1.524986	3.709870	1.945309
H	-1.184364	4.329964	2.764460
C	-4.553303	-3.167467	3.324436
H	-3.688395	-3.827913	3.308682
N	-0.381451	-0.526760	1.409598
N	-1.481871	-1.905270	-0.811211
C	0.127175	-2.853054	0.791693
C	0.257578	-1.734865	1.603594
C	0.934455	-0.421711	3.304008
H	1.395645	-0.005061	4.190122
C	-0.336677	1.617065	2.617047
C	-0.688274	-2.925661	-0.327792
C	0.028818	0.289721	2.444092
C	1.078783	-1.674620	2.781489
H	1.679963	-2.494073	3.154027
C	-2.125780	-2.420484	-1.916625
C	-0.845770	-4.101743	-1.140330
H	-0.332150	-5.038991	-0.969535
C	-1.739721	-3.790390	-2.120799
H	-2.108642	-4.418960	-2.920814
O	0.079168	0.519891	-1.282315
C	2.080745	1.720837	-1.656092
C	0.761641	1.570181	-1.168222
H	0.308507	2.432393	-0.653764
C	2.762445	2.955537	-1.583559
H	2.165182	3.843283	-1.378885
H	-3.446724	-2.263690	-3.566767
H	0.694824	-3.738251	1.058892
H	0.065834	2.140677	3.477911
H	-3.815218	3.704125	-1.344482
H	-5.423894	-1.348990	2.624825
H	-5.394886	-3.483155	3.933345
H	2.511211	0.878771	-2.187182
H	3.559957	3.127787	-2.299503
C	5.122767	-0.435932	-0.198400

C	5.140302	-1.627952	0.543964
C	5.961337	-0.331170	-1.319833
C	5.973965	-2.683731	0.178676
H	4.510987	-1.712836	1.426367
C	6.794309	-1.387412	-1.686373
H	5.944248	0.570244	-1.926708
C	6.804383	-2.566109	-0.937654
H	5.983830	-3.594214	0.771391
H	7.430612	-1.292456	-2.561619
H	7.456787	-3.386706	-1.221720
C	2.750422	3.458223	1.154067
H	1.825603	2.899483	0.998536
H	3.113556	3.219055	2.160193
H	2.493085	4.521381	1.130932
H	5.636703	2.148800	-0.325156
H	2.511778	-0.638744	0.351130

^{4}TS1E2C$_{exo\text{-}trans\text{-}m}$

C	-4.213695	0.550754	-0.142798
C	-4.826846	1.785525	0.175217
C	-4.136216	2.998330	0.284061
C	-2.877222	0.518549	-0.491968
H	-2.376549	1.370952	-0.933883
Fe	1.487294	-0.041178	-0.060163
N	1.393603	1.747839	-0.970481
N	0.258722	-0.807523	-1.453021
O	3.206215	-0.529953	-1.486481
C	-0.127342	1.196693	-2.824564
C	0.665684	2.072029	-2.097835
C	1.686840	3.981330	-1.478693
H	2.057855	4.994507	-1.393675
C	5.385005	-1.277394	-2.021400
C	2.894760	3.035509	0.493236
C	-0.311627	-0.143114	-2.517755
C	2.033405	2.908917	-0.586649
C	0.841797	3.462687	-2.415439
H	0.379350	3.963944	-3.255928
C	4.303667	-0.936374	-1.106331
H	4.501492	-1.056346	-0.027017
C	-0.222945	-2.098533	-1.509160
C	-1.170678	-1.032154	-3.251917
H	-1.733567	-0.753523	-4.133335
C	-1.119247	-2.242642	-2.624206
H	-1.628206	-3.160747	-2.887703
C	6.561798	-1.713856	-1.547682
H	6.737238	-1.823169	-0.479162
N	1.674087	-1.852812	0.778071
N	2.815821	0.696777	1.253975
C	3.244656	-1.319999	2.594739
C	2.426191	-2.186181	1.885013
C	1.348897	-4.076982	1.310036
H	0.956479	-5.083113	1.239791
C	0.111993	-3.122856	-0.636301
C	3.414729	0.024647	2.300211
C	0.999991	-3.002352	0.420647
C	2.234552	-3.572765	2.214705
H	2.716483	-4.079557	3.040656
C	3.256220	2.001791	1.343201
C	4.253606	0.921078	3.048495
H	4.834240	0.637417	3.916705
C	4.153935	2.145208	2.457164
H	4.637729	3.071169	2.739812
O	-0.135476	0.386546	1.197122
C	-2.161417	1.456480	1.771983
C	-0.870967	1.402368	1.184196
H	-0.504757	2.311288	0.677903
C	-2.875699	2.663831	1.886356
H	-2.331535	3.591310	1.716735
H	3.323653	4.014057	0.681673
H	3.778768	-1.719532	3.450338
H	-0.348515	-4.091678	-0.798029
H	-0.642091	1.588424	-3.695676
H	5.187480	-1.160013	-3.083638
H	7.385584	-1.975139	-2.204783
H	-2.521871	0.548993	2.244990
H	-3.632149	2.735213	2.661488
C	-4.933517	-0.730276	0.093413
C	-4.727620	-1.818222	-0.771963
C	-5.809022	-0.893922	1.179357
C	-5.378788	-3.031698	-0.558678
H	-4.068033	-1.700169	-1.627676
C	-6.461230	-2.107775	1.391951
H	-5.962042	-0.076492	1.878716
C	-6.249052	-3.180243	0.523380
H	-5.216851	-3.858915	-1.244373
H	-7.129730	-2.218206	2.241022
H	-6.760258	-4.124415	0.687730
H	-5.852648	1.759086	0.539354

H	-2.332304	-0.416624	-0.537932
C	-4.877212	4.274304	0.610624
H	-5.322366	4.700452	-0.297055
H	-5.685780	4.095441	1.327394
H	-4.209184	5.032168	1.032679
H	-3.273404	3.134964	-0.365698

[4]TS2E2C$_{endo\text{-}cis\text{-}m}$

C	-3.560423	0.656714	-0.104650
C	-4.047470	1.891154	-0.593859
C	-3.381732	3.120691	-0.480262
C	-2.408706	0.619871	0.657228
H	-1.932667	-0.324113	0.895061
Fe	1.687929	-0.221866	0.353899
N	2.620206	1.462166	-0.201986
N	2.881708	-1.260618	-0.875102
C	3.973000	0.592894	-2.065166
C	3.481107	1.616863	-1.275138
C	3.200427	3.690186	-0.449471
H	3.235776	4.751275	-0.239436
C	1.656467	3.060020	1.402029
C	3.701296	-0.748846	-1.862532
C	2.448136	2.735601	0.313726
C	3.834969	2.998201	-1.437268
H	4.499441	3.373780	-2.204418
C	2.900968	-2.630120	-1.057382
C	4.250631	-1.810829	-2.658543
H	4.929774	-1.666113	-3.488494
C	3.757080	-2.977131	-2.157312
H	3.945518	-3.989107	-2.491091
N	1.113312	-1.943236	1.210280
N	0.874893	0.777451	1.896410
C	-0.267656	-1.074049	3.049710
C	0.237982	-2.098975	2.269031
C	0.633381	-4.185221	1.529273
H	0.668195	-5.254875	1.369181
C	2.192278	-3.551011	-0.306280
C	0.050569	0.262713	2.883897
C	1.360302	-3.222992	0.749628
C	-0.064191	-3.488696	2.469402
H	-0.718748	-3.867922	3.243327
C	0.925644	2.141378	2.137044
C	-0.435320	1.315806	3.727520
H	-1.093879	1.168417	4.573635
C	0.108254	2.479520	3.266192
H	-0.010684	3.481188	3.658646
O	0.176683	-0.236970	-0.973624
C	-0.857476	1.924167	-1.052939
C	-0.729163	0.551242	-1.371106
H	-1.452235	0.129941	-2.084396
C	-1.783603	2.769626	-1.690962
H	-2.286647	2.405471	-2.583121
H	1.612353	4.100963	1.704339
H	-0.936774	-1.338021	3.861768
H	2.297237	-4.600132	-0.560873
H	4.639271	0.857418	-2.879173
H	-0.162691	2.336649	-0.330705
H	-1.534668	3.826715	-1.734915
C	-4.163212	-0.619758	-0.577638
C	-4.287176	-1.709012	0.300033
C	-4.588685	-0.775991	-1.907217
C	-4.822706	-2.918815	-0.138040
H	-3.984034	-1.595622	1.337468
C	-5.123145	-1.986733	-2.345909
H	-4.479812	0.045141	-2.611207
C	-5.242626	-3.061157	-1.462277
H	-4.922402	-3.747561	0.557086
H	-5.440906	-2.092486	-3.379154
H	-5.662993	-4.002787	-1.803073
H	-4.905498	1.855819	-1.263675
H	-2.037380	1.479279	1.201099
C	-4.044822	4.380015	-0.994723
H	-4.530119	4.212749	-1.962092
H	-4.814108	4.713836	-0.287827
H	-3.327561	5.198582	-1.107295
H	-2.750488	3.271478	0.393486

[4]TS2E2C$_{endo\text{-}trans\text{-}m}$

C	3.522122	-0.911586	-0.335033
C	3.824382	-2.157919	-0.933571
C	3.056942	-3.341142	-0.944825
H	3.495880	-4.098428	-1.595747
C	2.393744	-0.737866	0.438472
H	2.068561	0.257641	0.719112
Fe	-1.651925	0.310456	0.374107
N	-1.889467	-1.638337	0.791678
N	-3.209525	0.200614	-0.877463
C	-3.514274	-2.237495	-0.958213

C	-2.646258	-2.550547	0.073650
C	-1.540979	-3.778445	1.601589
H	-1.148860	-4.566122	2.231932
C	-0.310612	-1.890115	2.664379
C	-3.791429	-0.948746	-1.378926
C	-1.209922	-2.389064	1.737509
C	-2.422902	-3.879256	0.565257
H	-2.903089	-4.766286	0.172987
C	-3.816158	1.242661	-1.554149
C	-4.765961	-0.620485	-2.381588
H	-5.360863	-1.347368	-2.919003
C	-4.787200	0.737617	-2.483987
H	-5.400508	1.356873	-3.125369
N	-1.788826	2.312179	0.326465
N	-0.429942	0.463164	1.959422
C	0.015047	2.883193	1.895598
C	-0.951333	3.209284	0.960508
C	-2.253196	4.469833	-0.370781
H	-2.744169	5.269957	-0.909023
C	-3.534704	2.586332	-1.380777
C	0.234320	1.603658	2.377565
C	-2.580821	3.077180	-0.506394
C	-1.236303	4.550780	0.531134
H	-0.720914	5.431742	0.890746
C	0.052067	-0.557101	2.759641
C	1.154272	1.285664	3.431936
H	1.787555	2.007343	3.931344
C	1.032290	-0.051166	3.676955
H	1.550524	-0.651426	4.413447
O	-0.226905	0.299992	-1.034576
C	0.656606	-1.896120	-1.404832
C	0.629796	-0.489629	-1.534977
H	1.375033	-0.029328	-2.199723
C	1.550020	-2.712181	-2.124573
H	2.095644	-2.267093	-2.952434
H	0.145929	-2.590799	3.355578
H	0.609130	3.689463	2.312215
H	-4.088275	3.300932	-1.980263
H	-4.044116	-3.052748	-1.439039
H	-0.079173	-2.348490	-0.751282
H	1.231584	-3.734211	-2.320139
C	4.321901	0.290358	-0.709274
C	4.639092	1.246857	0.268827
C	4.746567	0.511200	-2.029560
C	5.364853	2.389720	-0.062758
H	4.333564	1.077718	1.298114
C	5.469749	1.656038	-2.361711
H	4.490983	-0.202026	-2.809113
C	5.782459	2.597353	-1.378960
H	5.613996	3.113274	0.708227
H	5.784234	1.815349	-3.389050
H	6.350640	3.486165	-1.637223
C	2.338096	-3.985323	0.231594
H	3.065846	-4.211594	1.019530
H	1.545573	-3.377911	0.668924
H	1.886450	-4.929793	-0.086662
H	4.641714	-2.138676	-1.652160
H	1.873100	-1.550533	0.914855

^{4}TS2E2C$_{exo\text{-}cis\text{-}m}$

C	-3.707082	-0.952523	-0.343738
C	-3.915695	-2.226598	-0.928456
C	-2.980979	-3.270950	-1.079223
H	-3.383802	-4.105057	-1.653731
C	-2.506175	-0.629090	0.248182
H	-1.816310	-1.364681	0.623729
Fe	1.814992	0.349408	0.370916
N	3.489695	0.236886	-0.716393
N	2.060612	-1.568962	0.904503
C	3.916943	-2.182635	-0.590074
C	4.184388	-0.908986	-1.059869
C	5.197390	0.748021	-2.193861
H	5.838475	1.353491	-2.821065
C	3.720425	2.588648	-1.397356
C	2.933722	-2.482749	0.337592
C	4.102946	1.259296	-1.417393
C	5.244566	-0.595713	-1.976626
H	5.933982	-1.322041	-2.386776
C	1.290278	-2.300697	1.793787
C	2.696019	-3.794372	0.867820
H	3.253565	-4.678673	0.587863
C	1.681835	-3.680814	1.773049
H	1.237077	-4.453111	2.387147
N	0.430668	0.541612	1.817644
N	1.835448	2.337048	0.161665
C	-0.089679	2.933687	1.572168
C	-0.297521	1.678806	2.119418
C	-1.181646	0.057589	3.405203

H	-1.757574	-0.527365	4.110464
C	0.280314	-1.789073	2.590793
C	0.917798	3.236216	0.673528
C	-0.110634	-0.460526	2.602160
C	-1.300695	1.382651	3.102159
H	-1.991509	2.108653	3.510840
C	2.657574	3.083226	-0.661672
C	1.171563	4.554709	0.164269
H	0.593151	5.432454	0.421223
C	2.253289	4.461017	-0.658219
H	2.745587	5.245018	-1.218518
O	0.492523	0.015033	-1.104317
C	-1.247533	-1.217116	-2.113443
C	-0.038358	-1.094745	-1.408597
H	0.481256	-2.015023	-1.104470
C	-1.773647	-2.477102	-2.482614
H	-1.094636	-3.328492	-2.451236
H	4.287918	3.288331	-2.001336
H	-0.740148	3.740050	1.893521
H	-0.237167	-2.474721	3.253448
H	4.538335	-2.995739	-0.949697
H	-1.733800	-0.299409	-2.426408
H	-2.455234	-2.502714	-3.327208
C	-4.721163	0.121485	-0.546127
C	-5.008608	1.009904	0.502856
C	-5.387507	0.287357	-1.770925
C	-5.939438	2.033583	0.333555
H	-4.513601	0.878738	1.461830
C	-6.317020	1.312490	-1.940783
H	-5.160318	-0.370140	-2.606115
C	-6.596881	2.187102	-0.888764
H	-6.159943	2.704886	1.158690
H	-6.817905	1.432333	-2.897071
H	-7.324641	2.982442	-1.020538
C	-1.998993	-3.739378	-0.011417
H	-1.140020	-3.079392	0.124923
H	-2.505089	-3.823149	0.957020
H	-1.609780	-4.726667	-0.276649
H	-4.828831	-2.330135	-1.511711
H	-2.262740	0.402843	0.476338

[4]TS2E2C$_{exo\text{-}trans\text{-}m}$

C	3.680168	0.782393	-0.153041
C	4.068329	2.076956	-0.578914
C	3.202823	3.175531	-0.654045
C	2.419211	0.577331	0.367027
H	1.845014	1.370646	0.829188
Fe	-1.846998	-0.305449	0.357838
N	-2.750523	1.481717	0.272573
N	-0.750418	0.357098	1.907542
C	-1.385228	2.735974	1.891493
C	-2.384777	2.640510	0.937845
C	-4.062144	3.255339	-0.428918
H	-4.832118	3.784499	-0.974996
C	-4.459252	1.011210	-1.434189
C	-0.638322	1.664195	2.351863
C	-3.782574	1.855386	-0.570981
C	-3.194338	3.742510	0.502795
H	-3.107661	4.753150	0.880121
C	0.159610	-0.365236	2.659817
C	0.357187	1.756922	3.381169
H	0.624030	2.670852	3.895943
C	0.847473	0.498348	3.575445
H	1.602808	0.170418	4.277657
N	-1.228279	-2.167613	0.750660
N	-3.235855	-1.047317	-0.870674
C	-2.549573	-3.408560	-0.911221
C	-1.578523	-3.319522	0.070566
C	0.018635	-3.961657	1.517245
H	0.741428	-4.504934	2.111816
C	0.404728	-1.722378	2.536604
C	-3.328328	-2.346511	-1.335339
C	-0.244279	-2.555476	1.642614
C	-0.805513	-4.434151	0.541043
H	-0.899786	-5.445510	0.167939
C	-4.200715	-0.341421	-1.566577
C	-4.364386	-2.452550	-2.323585
H	-4.634195	-3.369034	-2.831752
C	-4.906972	-1.211058	-2.464569
H	-5.713991	-0.896671	-3.113305
O	-0.454651	0.066272	-1.058254
C	1.351330	1.330475	-1.912960
C	0.156504	1.166060	-1.184199
H	-0.265648	2.056310	-0.693076
C	1.909347	2.607581	-2.131633
H	1.267101	3.470481	-1.963943
H	-5.248878	1.437761	-2.043285
H	-2.725535	-4.378467	-1.363678

H	1.152189	-2.162828	3.187848
H	-1.198421	3.709338	2.332717
H	1.781854	0.446220	-2.370740
H	2.585956	2.726809	-2.972228
C	4.549111	-0.388620	-0.449203
C	4.646183	-1.442351	0.474556
C	5.268724	-0.480989	-1.651847
C	5.440115	-2.555435	0.204768
H	4.114434	-1.372033	1.420010
C	6.062262	-1.594876	-1.922270
H	5.188610	0.309635	-2.393277
C	6.151074	-2.634745	-0.994639
H	5.513725	-3.356038	0.935362
H	6.606269	-1.653560	-2.860559
H	6.772843	-3.500251	-1.204047
H	5.043634	2.175289	-1.053205
H	2.026342	-0.423776	0.501717
C	3.724697	4.531229	-1.072131
H	4.192824	5.037423	-0.219058
H	4.476463	4.444881	-1.863686
H	2.920101	5.178745	-1.435066
H	2.389757	3.210479	0.069561

Reaction F

^{4}IF

Fe	0.000745	0.158909	-0.148338
N	0.147708	-1.838550	-0.007699
N	-1.025317	0.200998	1.575525
O	1.790653	0.575821	1.113241
C	-1.072684	-2.203778	2.097349
C	-0.371386	-2.644482	0.985871
C	0.578549	-4.056203	-0.488416
H	0.948737	-4.916959	-1.030084
C	1.351093	-2.314564	-2.099974
C	-1.361883	-0.876252	2.371627
C	0.718194	-2.695471	-0.927513
C	-0.094657	-4.024909	0.697537
H	-0.389849	-4.854619	1.326675
C	-1.462920	1.321389	2.254073
C	-2.040519	-0.423580	3.554577
H	-2.411754	-1.073960	4.335958
C	-2.098859	0.937706	3.483760
H	-2.530054	1.631290	4.193913
N	-0.163362	2.148814	-0.293334
N	1.031765	0.114538	-1.862452
C	1.047165	2.514332	-2.402940
C	0.338419	2.953088	-1.296184
C	-0.605799	4.367116	0.178380
H	-0.976033	5.227995	0.719738
C	-1.333118	2.630496	1.817018
C	1.364740	1.190246	-2.660534
C	-0.730208	3.007383	0.627238
C	0.053049	4.333264	-1.014275
H	0.335286	5.160713	-1.652122
C	1.494478	-1.004071	-2.528127
C	2.072382	0.740804	-3.827491
H	2.446746	1.392108	-4.606512
C	2.151426	-0.617869	-3.746251
H	2.604300	-1.309493	-4.444728
O	-1.800751	-0.321927	-1.382727
H	1.759937	-3.097716	-2.729551
H	1.378397	3.260197	-3.117562
H	-1.731888	3.415218	2.451003
H	-1.411635	-2.950292	2.807817
C	2.880373	0.058897	1.367879
C	-3.022050	-0.188275	-1.296329
C	-3.694821	0.882201	-0.489738
H	-4.325731	1.443104	-1.193637
H	-2.959744	1.569523	-0.073366
C	-4.605878	0.252729	0.607745
H	-5.422996	0.952978	0.808976
H	-4.033815	0.155905	1.534359
C	-5.169705	-1.124848	0.188030
H	-6.121230	-1.311419	0.696103
H	-4.480279	-1.917386	0.513418
C	-5.332803	-1.221007	-1.336544
H	-5.967455	-0.400472	-1.694924
H	-5.834312	-2.150594	-1.623643
C	-3.943319	-1.158921	-2.004867
H	-3.446490	-2.135682	-2.003370
H	-4.013590	-0.847973	-3.057090
C	3.812812	0.677852	2.370748
H	3.866570	-0.036135	3.206425
H	3.382276	1.606079	2.753146
C	5.243661	0.879574	1.794430
H	5.941499	0.900515	2.637330
H	5.300286	1.863655	1.318596
C	5.650696	-0.220956	0.781272

H	6.722805	-0.426054	0.861099
H	5.479350	0.138667	-0.242646
C	4.835064	-1.507315	0.976975
H	4.970559	-1.890385	1.996760
H	5.181381	-2.295777	0.300744
C	3.350716	-1.215937	0.706966
H	3.157016	-1.111065	-0.366646
H	2.694301	-2.027197	1.048471

^{6}IF

Fe	0.074626	-0.461967	0.147087
N	0.033975	-2.478807	-0.195950
N	1.053221	-0.770589	1.917370
O	1.848404	-0.406721	-1.074549
C	1.267394	-3.222856	1.797411
C	0.600235	-3.452138	0.592204
C	-0.325593	-4.531863	-1.163658
H	-0.672339	-5.267875	-1.877543
C	-1.190953	-2.470296	-2.332209
C	1.457346	-1.988781	2.421001
C	-0.533864	-3.109337	-1.279376
C	0.378186	-4.743037	-0.011758
H	0.717008	-5.685061	0.399728
C	1.353332	0.182205	2.868456
C	2.057501	-1.791209	3.715975
H	2.464592	-2.580107	4.335226
C	1.986931	-0.454810	3.994797
H	2.328421	0.059181	4.884106
N	0.074549	1.575603	0.506272
N	-0.938645	-0.124246	-1.620315
C	-1.057504	2.332494	-1.548063
C	-0.444018	2.554898	-0.312941
C	0.270873	3.612713	1.553462
H	0.517608	4.339708	2.316638
C	1.095065	1.549199	2.749056
C	-1.294815	1.093975	-2.150087
C	0.505395	2.194667	1.659940
C	-0.308207	3.835986	0.334403
H	-0.629048	4.779826	-0.087688
C	-1.370356	-1.094728	-2.496379
C	-1.984752	0.885203	-3.399324
H	-2.377459	1.672871	-4.029609
C	-2.034245	-0.465428	-3.610527
H	-2.475024	-0.992971	-4.446656
O	-1.829163	-0.495105	1.162789
H	-1.588366	-3.108316	-3.115592
H	-1.410976	3.209802	-2.081233
H	1.388200	2.175125	3.586240
H	1.649782	-4.095590	2.317494
C	2.893887	0.247817	-1.146502
C	-2.930500	0.038334	1.000338
C	-3.339039	1.248291	1.796108
H	-2.444404	1.711279	2.222072
H	-3.958517	0.896454	2.635819
C	-4.152835	2.211562	0.908243
H	-3.501457	2.542085	0.090322
H	-4.400739	3.108136	1.484523
C	-5.435892	1.553334	0.337492
H	-5.624244	1.955442	-0.664277
H	-6.303781	1.826790	0.946803
C	-5.339834	0.011148	0.270701
H	-6.025773	-0.381617	-0.486115
H	-5.658586	-0.424802	1.225687
C	-3.910364	-0.460368	-0.029610
H	-3.815725	-1.548297	-0.110363
H	-3.565055	-0.053968	-0.993165
C	3.289421	1.278793	-0.123062
H	3.005984	0.914612	0.868175
H	2.654349	2.158138	-0.310796
C	4.776475	1.641810	-0.221897
H	5.369466	0.752446	0.026110
H	5.022405	2.391159	0.536979
C	5.159695	2.153925	-1.628018
H	6.239629	2.034904	-1.764296
H	4.957604	3.228581	-1.696301
C	4.391875	1.423003	-2.762212
H	5.037183	1.270198	-3.632296
H	3.554532	2.040600	-3.109359
C	3.826018	0.059561	-2.312345
H	3.301268	-0.470589	-3.111600
H	4.646416	-0.590509	-1.970574

^{4}TS1F$_{1}$

Fe	-0.801321	0.510948	0.046963
N	-0.052022	-0.300328	1.721360
N	-0.687651	2.325841	0.888876
O	-3.007397	0.556420	1.018749
C	0.110501	1.707314	3.128911

C	0.274246	0.348997	2.891663
C	0.967259	-1.776905	3.170939
H	1.369777	-2.710218	3.543091
C	0.066731	-2.644975	1.001129
C	-0.310385	2.628170	2.178217
C	0.347116	-1.608262	1.882905
C	0.897857	-0.571524	3.805729
H	1.244851	-0.312766	4.797728
C	-0.985936	3.524587	0.283972
C	-0.351202	4.053650	2.379808
H	-0.100288	4.551021	3.307752
C	-0.746922	4.610326	1.199467
H	-0.895286	5.656418	0.965132
N	-1.865521	1.289101	-1.467911
N	-1.252439	-1.335450	-0.604670
C	-2.742253	-0.768627	-2.475595
C	-2.588908	0.610457	-2.416954
C	-2.757767	2.785232	-2.980403
H	-2.989394	3.737555	-3.439514
C	-1.529253	3.671928	-0.986356
C	-2.091431	-1.671266	-1.643518
C	-1.980101	2.623123	-1.776412
C	-3.155200	1.540297	-3.363591
H	-3.771715	1.260018	-4.207969
C	-0.736242	-2.519558	-0.125435
C	-2.116250	-3.101530	-1.809199
H	-2.704774	-3.624573	-2.552109
C	-1.253422	-3.624411	-0.889492
H	-1.008949	-4.664013	-0.712457
O	0.912219	0.646032	-0.965002
H	0.404628	-3.640523	1.271247
H	-3.368635	-1.173310	-3.264263
H	-1.703623	4.681671	-1.343096
H	0.393630	2.085980	4.105483
C	-3.784130	-0.298550	1.432416
C	2.071285	1.244342	-0.771648
C	2.120615	2.715202	-1.291114
H	2.695574	2.795455	-2.219887
H	1.089934	2.986401	-1.534110
C	2.724501	3.682106	-0.259808
H	2.810500	4.678048	-0.710358
H	2.049787	3.788765	0.598420
C	4.100567	3.182473	0.201182
H	4.755752	3.124617	-0.679054

H	4.565805	3.909788	0.875125
C	4.007135	1.794138	0.889311
H	4.843949	1.159257	0.579265
H	4.110781	1.905145	1.974930
C	2.667238	1.084684	0.629738
H	1.912628	1.528235	1.281309
H	2.711616	0.026550	0.899080
C	-5.245814	-0.284518	1.071487
H	-5.823852	-0.227839	2.003938
H	-5.470766	0.601446	0.473314
C	-5.614462	-1.599040	0.325028
H	-6.701839	-1.717488	0.369672
H	-5.352054	-1.484889	-0.732305
C	-4.901361	-2.849475	0.909358
H	-5.581523	-3.707601	0.903480
H	-4.052773	-3.120698	0.268033
C	-4.364564	-2.599241	2.327418
H	-5.190548	-2.347362	3.004746
H	-3.900873	-3.506159	2.730464
C	-3.330640	-1.460877	2.293197
H	-2.371986	-1.804543	1.896430
H	-3.119965	-1.065741	3.296935
C	2.237128	-1.762379	-2.402534
C	3.233816	-0.989308	-1.719830
C	3.325311	0.409351	-1.912700
H	2.987190	0.781698	-2.873378
H	4.237739	0.892418	-1.577589
C	1.280478	-1.238219	-3.213265
H	1.206720	-0.161952	-3.332367
C	4.084527	-1.658733	-0.729934
C	5.427075	-1.271639	-0.540284
C	3.561256	-2.692117	0.075249
C	6.216072	-1.895031	0.420983
H	5.861921	-0.507925	-1.177322
C	4.345543	-3.295695	1.052583
H	2.520643	-2.976877	-0.034124
C	5.675820	-2.902854	1.225306
H	7.253888	-1.599597	0.541477
H	3.921697	-4.073739	1.680876
H	6.291441	-3.383070	1.980280
C	0.332614	-2.051177	-4.022276
H	-0.680710	-1.644067	-3.960703
H	0.627668	-1.992087	-5.081060
H	0.317178	-3.102509	-3.723709

H	2.272765	-2.842141	-2.281317

^{6}TS1F$_{1}$

Fe	-0.696726	0.481906	0.015243
N	0.008245	-0.384044	1.766374
N	-0.610876	2.331338	0.943160
O	-2.885124	0.575990	1.038357
C	0.288328	1.647622	3.128042
C	0.395242	0.271805	2.911150
C	0.944913	-1.899442	3.219865
H	1.286803	-2.849331	3.610932
C	-0.041366	-2.735825	1.049421
C	-0.168628	2.602382	2.215070
C	0.313100	-1.713216	1.934848
C	0.980238	-0.677294	3.828997
H	1.366598	-0.431678	4.809959
C	-0.980718	3.525379	0.374929
C	-0.222143	4.027537	2.444116
H	0.070452	4.518558	3.363408
C	-0.710372	4.598641	1.303052
H	-0.899400	5.646808	1.109934
N	-1.898418	1.302614	-1.463205
N	-1.280937	-1.407933	-0.609864
C	-2.746146	-0.763430	-2.482675
C	-2.631753	0.626845	-2.401818
C	-2.916305	2.822625	-2.854924
H	-3.213502	3.780419	-3.262393
C	-1.610523	3.669713	-0.865241
C	-2.110574	-1.702887	-1.665524
C	-2.075114	2.643013	-1.691340
C	-3.272115	1.577910	-3.284818
H	-3.913202	1.316574	-4.117089
C	-0.820190	-2.600818	-0.103756
C	-2.175316	-3.136033	-1.831836
H	-2.761718	-3.645737	-2.585878
C	-1.362682	-3.689281	-0.881569
H	-1.169321	-4.738802	-0.699016
O	0.953979	0.619393	-0.975538
H	0.238375	-3.746194	1.333995
H	-3.374863	-1.156282	-3.276558
H	-1.843133	4.684334	-1.174297
H	0.624507	2.016826	4.092196
C	-3.717278	-0.224621	1.452283
C	2.094497	1.254692	-0.811443
C	2.108348	2.701367	-1.371954
H	2.639940	2.759412	-2.327025
H	1.064982	2.960969	-1.574739
C	2.749017	3.693816	-0.386784
H	2.815491	4.677108	-0.866467
H	2.103205	3.819777	0.491083
C	4.141801	3.207496	0.035894
H	4.763798	3.124471	-0.865999
H	4.630262	3.953621	0.671331
C	4.077047	1.839912	0.766685
H	4.910584	1.201589	0.455098
H	4.204436	1.983193	1.845628
C	2.739002	1.109653	0.560980
H	1.996950	1.549059	1.232405
H	2.802577	0.055457	0.839310
C	-5.176235	-0.100250	1.101911
H	-5.746198	-0.026353	2.037991
H	-5.341812	0.811879	0.524090
C	-5.634277	-1.370539	0.327066
H	-6.725928	-1.426413	0.388055
H	-5.381640	-1.243966	-0.731095
C	-4.987444	-2.674888	0.867420
H	-5.709351	-3.497294	0.827333
H	-4.149308	-2.964257	0.220105
C	-4.447193	-2.500727	2.295477
H	-5.261790	-2.220351	2.975307
H	-4.040205	-3.444821	2.673532
C	-3.346825	-1.424718	2.299495
H	-2.402807	-1.818340	1.916426
H	-3.135641	-1.058012	3.314048
C	2.224488	-1.783212	-2.454872
C	3.239771	-1.011681	-1.787053
C	3.388911	0.365892	-2.026039
H	3.030629	0.748868	-2.974476
H	4.295185	0.849169	-1.677279
C	1.282482	-1.269015	-3.284880
H	1.229271	-0.194958	-3.438527
C	4.045463	-1.671118	-0.752272
C	5.399938	-1.333915	-0.555160
C	3.454933	-2.627242	0.099998
C	6.138123	-1.933549	0.460084
H	5.882085	-0.629601	-1.226035
C	4.188846	-3.202569	1.132389
H	2.403771	-2.869554	-0.014016

C	5.532325	-2.862230	1.312042
H	7.186261	-1.680373	0.587240
H	3.713672	-3.916328	1.798900
H	6.107976	-3.321784	2.110177
C	0.312923	-2.086754	-4.063458
H	-0.693842	-1.664277	-3.994541
H	0.591842	-2.058023	-5.127620
H	0.286701	-3.129777	-3.737594
H	2.236033	-2.858292	-2.294741

^{4}TS1F$_2$

Fe	0.695583	-0.125943	0.004276
N	1.894438	0.077615	-1.600531
N	1.066847	1.793741	0.488486
O	2.634596	-0.782648	1.260968
C	2.177771	2.517469	-1.582986
C	2.310803	1.263737	-2.163923
C	3.052069	-0.348897	-3.550607
H	3.510298	-0.921969	-4.346353
C	2.136621	-2.279986	-2.243822
C	1.649786	2.747782	-0.318599
C	2.340239	-0.919793	-2.435575
C	3.017863	1.004270	-3.392037
H	3.448649	1.767987	-4.026654
C	0.790090	2.420857	1.684738
C	1.720183	4.004447	0.377665
H	2.132318	4.915309	-0.037066
C	1.206406	3.796852	1.625211
H	1.102042	4.506322	2.435936
N	-0.180602	-0.392723	1.798739
N	0.656171	-2.117512	-0.290629
C	-0.765812	-2.768985	1.603243
C	-0.804236	-1.535394	2.239313
C	-1.086544	-0.029431	3.890606
H	-1.339582	0.495881	4.802417
C	0.154372	1.837084	2.773058
C	-0.019416	-3.044592	0.465393
C	-0.326261	0.535666	2.804646
C	-1.399301	-1.305903	3.532075
H	-1.948190	-2.048472	4.096960
C	1.358838	-2.829601	-1.234098
C	0.242360	-4.372491	-0.030586
H	-0.168260	-5.279980	0.393246
C	1.117573	-4.241109	-1.066650
H	1.557937	-5.017387	-1.679310
O	-0.954541	0.216547	-1.051791
H	2.601314	-2.961680	-2.949057
H	-1.263921	-3.600981	2.091203
H	-0.015155	2.457467	3.646887
H	2.585272	3.367462	-2.120407
C	3.770470	-0.373505	1.480422
C	-1.842788	1.190749	-1.174624
C	-2.327347	1.857819	0.140009
H	-3.348188	1.556542	0.390185
H	-1.699581	1.479219	0.942377
C	-2.274907	3.393005	0.075677
H	-2.712706	3.803453	0.993498
H	-1.233610	3.735814	0.052119
C	-3.031653	3.913868	-1.153630
H	-4.082198	3.603317	-1.067651
H	-3.042399	5.009064	-1.159161
C	-2.424423	3.375008	-2.479112
H	-3.224705	3.064351	-3.161283
H	-1.886608	4.174196	-3.001750
C	-1.438975	2.217382	-2.250750
H	-0.485645	2.614685	-1.886661
H	-1.203920	1.691247	-3.181766
C	4.765372	-1.193027	2.262200
H	4.960593	-0.635690	3.190273
H	4.316547	-2.150422	2.537333
C	6.104287	-1.369987	1.492514
H	6.888410	-1.581739	2.226630
H	6.032624	-2.254166	0.850722
C	6.482712	-0.130433	0.640011
H	7.569318	0.003075	0.632316
H	6.183012	-0.295579	-0.404256
C	5.780205	1.140832	1.138863
H	6.042917	1.330937	2.187832
H	6.112265	2.017367	0.572116
C	4.260753	0.973273	0.986317
H	3.963113	1.054498	-0.064232
H	3.697970	1.751290	1.518604
C	-3.695504	-0.712059	-1.247718
C	-3.081869	-1.954706	-1.610219
C	-2.105940	-2.077695	-2.548088
H	-1.706930	-1.187951	-3.024940
C	-3.378075	0.487545	-1.938580
H	-3.084194	0.372370	-2.977188

H	-4.069304	1.313206	-1.800379
C	-4.566105	-0.674845	-0.071197
C	-5.684341	0.184333	-0.012714
C	-4.265795	-1.470365	1.054987
C	-6.475059	0.240742	1.130090
H	-5.953701	0.778353	-0.880377
C	-5.042325	-1.388835	2.205326
H	-3.383891	-2.101365	1.043576
C	-6.152193	-0.539829	2.244247
H	-7.344750	0.890312	1.153381
H	-4.780869	-1.983700	3.075382
H	-6.765330	-0.487104	3.139209
H	-3.440847	-2.854336	-1.116836
C	-1.559327	-3.374880	-3.026016
H	-1.928312	-3.559547	-4.046589
H	-0.468545	-3.337216	-3.094339
H	-1.850279	-4.217189	-2.393471

^{6}TS1F$_2$

Fe	0.613527	-0.122473	-0.097755
N	1.909436	0.388029	-1.645862
N	0.917946	1.781019	0.692892
O	2.569087	-0.776873	1.127712
C	2.052072	2.807980	-1.238002
C	2.271497	1.662699	-2.008373
C	3.090543	0.304159	-3.617193
H	3.579673	-0.116386	-4.486573
C	2.270468	-1.853346	-2.592054
C	1.472297	2.854056	0.032885
C	2.399789	-0.462959	-2.605770
C	3.001798	1.617116	-3.254437
H	3.407924	2.480377	-3.766043
C	0.555016	2.216791	1.946962
C	1.440231	4.011766	0.893626
H	1.812330	4.992630	0.626482
C	0.885632	3.615333	2.078737
H	0.709273	4.212060	2.964572
N	-0.183952	-0.708478	1.732997
N	0.827766	-2.120189	-0.615756
C	-0.392703	-3.116662	1.276145
C	-0.597418	-1.976147	2.056420
C	-1.053891	-0.693175	3.860853
H	-1.349526	-0.312225	4.830074
C	-0.068142	1.438087	2.926067
C	0.326174	-3.191343	0.079457
C	-0.418778	0.089422	2.824146
C	-1.170310	-1.967062	3.384818
H	-1.571412	-2.835894	3.891538
C	1.577323	-2.619847	-1.651761
C	0.742651	-4.416335	-0.562962
H	0.489195	-5.412062	-0.221880
C	1.527702	-4.064134	-1.623571
H	2.033923	-4.715094	-2.325115
O	-1.017667	0.235634	-1.061382
H	2.771534	-2.396196	-3.388078
H	-0.739248	-4.057894	1.692888
H	-0.307000	1.934278	3.861793
H	2.430098	3.744864	-1.635912
C	3.670798	-0.347903	1.457303
C	-1.893948	1.213401	-1.156329
C	-2.395379	1.841476	0.160178
H	-3.418451	1.533233	0.388791
H	-1.778377	1.447282	0.965619
C	-2.347436	3.377823	0.115769
H	-2.780550	3.772116	1.042255
H	-1.307800	3.723598	0.090188
C	-3.119233	3.909255	-1.100055
H	-4.169352	3.601949	-1.001811
H	-3.124899	5.004243	-1.096222
C	-2.536529	3.380129	-2.440824
H	-3.348400	3.060408	-3.104156
H	-2.018905	4.184684	-2.974620
C	-1.534502	2.232006	-2.242858
H	-0.572857	2.635443	-1.899266
H	-1.315726	1.708532	-3.178627
C	4.630528	-1.188770	2.259724
H	4.744192	-0.681526	3.229222
H	4.190021	-2.170866	2.447179
C	6.024378	-1.288320	1.578301
H	6.763580	-1.511308	2.354531
H	6.026211	-2.141721	0.892447
C	6.417293	0.001809	0.813004
H	7.499165	0.159007	0.871987
H	6.178393	-0.114859	-0.253273
C	5.657536	1.228874	1.337782
H	5.857095	1.367041	2.408588
H	5.999791	2.141832	0.838701
C	4.152684	1.040220	1.089102

H	3.908695	1.193270	0.033110
H	3.543700	1.763970	1.646316
C	-3.737957	-0.741319	-1.243425
C	-3.094969	-1.971299	-1.621542
C	-2.170084	-2.084132	-2.606619
H	-1.835341	-1.193380	-3.130946
C	-3.527066	0.452797	-1.958119
H	-3.226572	0.373277	-2.997317
H	-4.203061	1.279106	-1.766031
C	-4.519899	-0.718717	-0.002923
C	-5.682790	0.070525	0.116197
C	-4.067771	-1.436859	1.123417
C	-6.373310	0.133327	1.322441
H	-6.062708	0.603323	-0.750162
C	-4.742273	-1.342855	2.336104
H	-3.152926	-2.016188	1.060818
C	-5.899691	-0.565717	2.437009
H	-7.280164	0.725939	1.395607
H	-4.358279	-1.868800	3.204625
H	-6.432929	-0.504815	3.381309
H	-3.389563	-2.868828	-1.083706
C	-1.584720	-3.369107	-3.070962
H	-1.951883	-3.587380	-4.085012
H	-0.495892	-3.294869	-3.145129
H	-1.840073	-4.208901	-2.419677

^{4}TS2F$_1$

Fe	1.327324	0.142350	0.705324
N	0.015859	-0.272404	2.169162
N	2.007969	-1.743719	0.846424
C	0.511193	-2.646110	2.576054
C	-0.217019	-1.486441	2.787121
C	-1.864569	-0.133396	3.508155
H	-2.723646	0.307748	3.996630
C	-1.057619	1.929263	2.356911
C	1.533339	-2.766062	1.647653
C	-0.979511	0.571004	2.622156
C	-1.377541	-1.400818	3.629863
H	-1.761763	-2.216241	4.228715
C	2.976889	-2.318476	0.046415
C	2.208920	-3.995177	1.336375
H	2.022777	-4.939679	1.830851
C	3.087212	-3.722590	0.329139
H	3.774373	-4.396053	-0.166248
N	3.011589	0.626417	-0.275674
N	1.013808	2.095201	1.047247
C	2.924868	3.076771	-0.145715
C	3.476309	1.885955	-0.590601
C	4.809936	0.469757	-1.722024
H	5.570120	0.007126	-2.337892
C	3.791133	-1.633183	-0.842525
C	1.763855	3.166422	0.605598
C	3.830745	-0.252724	-0.954899
C	4.604320	1.794072	-1.478808
H	5.156388	2.643424	-1.859764
C	-0.088027	2.643726	1.672018
C	1.124877	4.402883	0.966712
H	1.521895	5.385444	0.746785
C	-0.036285	4.079053	1.602978
H	-0.779788	4.741763	2.026744
O	0.216306	-0.052067	-0.888763
H	-1.876047	2.485365	2.802755
H	3.411764	4.001285	-0.438393
H	4.505367	-2.209320	-1.421170
H	0.229317	-3.531139	3.136429
C	-0.505759	-1.031050	-1.389309
C	0.274201	-1.967864	-2.353934
H	0.035070	-1.759962	-3.401714
H	1.332709	-1.727121	-2.218986
C	-0.003056	-3.453610	-2.070436
H	0.499038	-4.061911	-2.831652
H	0.434723	-3.741035	-1.106161
C	-1.512796	-3.726823	-2.075813
H	-1.905474	-3.454767	-3.065239
H	-1.708396	-4.797766	-1.957890
C	-2.247330	-2.921611	-0.970972
H	-3.199998	-2.537127	-1.349828
H	-2.508293	-3.580859	-0.135263
C	-1.397993	-1.773478	-0.397817
H	-0.686843	-2.193277	0.317959
H	-2.005381	-1.062335	0.167079
C	-1.754864	2.151059	-1.817599
C	-2.381087	0.859890	-1.915189
C	-1.771459	-0.195789	-2.621398
H	-1.085656	0.075510	-3.415853
H	-2.380302	-1.062086	-2.857744
C	-0.529651	2.455426	-2.316195
H	0.067931	1.680249	-2.786275

C	-3.619519	0.627336	-1.162622
C	-4.632152	-0.214589	-1.666419
C	-3.803025	1.220074	0.104195
C	-5.786376	-0.455437	-0.928213
H	-4.529079	-0.646473	-2.656984
C	-4.946342	0.955265	0.850947
H	-3.019272	1.843384	0.520679
C	-5.942899	0.121675	0.335695
H	-6.567010	-1.088909	-1.338201
H	-5.063329	1.399221	1.835305
H	-6.841304	-0.073535	0.913866
C	0.060486	3.822105	-2.317386
H	1.106700	3.793382	-1.998626
H	0.058773	4.212692	-3.346200
H	-0.489306	4.518952	-1.679520
H	-2.321908	2.941973	-1.333312

^{6}TS2F$_1$

Fe	1.170135	0.142979	0.615348
N	-0.055606	1.352130	1.807353
N	0.700261	-1.419497	1.917102
C	-1.206372	-0.397091	3.094904
C	-1.039597	0.917571	2.668801
C	-1.301996	3.154675	2.532746
H	-1.612974	4.184288	2.655301
C	0.684080	3.570469	1.048140
C	-0.389681	-1.473639	2.764161
C	-0.185032	2.722842	1.729287
C	-1.829145	2.037601	3.114624
H	-2.655611	1.970981	3.810563
C	1.200936	-2.705297	1.848491
C	-0.574461	-2.821266	3.236129
H	-1.353075	-3.133650	3.920072
C	0.406688	-3.582566	2.669462
H	0.591796	-4.641173	2.798748
N	2.912649	-0.917350	0.158953
N	2.229141	1.880487	0.143256
C	4.167948	0.857888	-0.983053
C	3.941505	-0.471770	-0.641365
C	4.257566	-2.696043	-0.436455
H	4.626639	-3.712579	-0.480478
C	2.295465	-3.105032	1.085895
C	3.404219	1.949846	-0.576955
C	3.095592	-2.274143	0.306992
C	4.781448	-1.581230	-1.021581
H	5.665293	-1.502255	-1.641323
C	1.828581	3.183232	0.357266
C	3.747181	3.328201	-0.821235
H	4.629939	3.659279	-1.352983
C	2.776350	4.090319	-0.238072
H	2.702594	5.169691	-0.203451
O	0.166796	-0.272656	-0.935312
H	0.483167	4.635635	1.109810
H	5.048547	1.069506	-1.581671
H	2.564173	-4.156098	1.124013
H	-2.028162	-0.594238	3.776196
C	-0.561422	-1.258076	-1.411851
C	0.223118	-2.252316	-2.299363
H	0.094511	-2.029987	-3.363056
H	1.282566	-2.090314	-2.071741
C	-0.187814	-3.710948	-2.033139
H	0.301106	-4.358706	-2.769191
H	0.179199	-4.024827	-1.047174
C	-1.712231	-3.867059	-2.103740
H	-2.044713	-3.554531	-3.103040
H	-1.993182	-4.920945	-2.008741
C	-2.424533	-3.024962	-1.013487
H	-3.358345	-2.606241	-1.403429
H	-2.716616	-3.666829	-0.175143
C	-1.535249	-1.906704	-0.439471
H	-0.879223	-2.340518	0.321350
H	-2.120035	-1.140385	0.075128
C	-1.474375	1.991978	-2.157848
C	-2.242876	0.772977	-2.112105
C	-1.808398	-0.389453	-2.767900
H	-1.122505	-0.274687	-3.598988
H	-2.507176	-1.209350	-2.885784
C	-0.236045	2.104875	-2.698491
H	0.258537	1.222061	-3.094230
C	-3.430710	0.734392	-1.252260
C	-4.561183	-0.033778	-1.599646
C	-3.435248	1.431948	-0.026072
C	-5.660822	-0.098300	-0.750511
H	-4.590852	-0.545454	-2.556679
C	-4.527811	1.344420	0.830623
H	-2.562527	1.999107	0.279313
C	-5.644164	0.585218	0.469726
H	-6.533771	-0.675290	-1.040101

H	-4.503873	1.869278	1.780454
H	-6.501829	0.529158	1.133774
C	0.517036	3.380639	-2.827167
H	1.540485	3.270686	-2.455138
H	0.602696	3.645308	-3.891628
H	0.032939	4.208579	-2.302095
H	-1.934031	2.881405	-1.734729

^{4}TS2F$_{2}$

Fe	-1.264510	0.167001	0.728698
N	-3.034202	0.002190	-0.216429
N	-1.473197	-1.743097	1.343383
C	-3.322623	-2.435375	-0.124067
C	-3.654032	-1.173301	-0.588818
C	-4.836186	0.453491	-1.595974
H	-5.554296	1.038736	-2.155480
C	-3.515499	2.364879	-0.683394
C	-2.339406	-2.685743	0.819769
C	-3.758922	1.008379	-0.821345
C	-4.763701	-0.899723	-1.461726
H	-5.411983	-1.655859	-1.885059
C	-0.719128	-2.419058	2.284956
C	-2.112061	-3.966232	1.429913
H	-2.664824	-4.865775	1.192265
C	-1.118476	-3.797396	2.348020
H	-0.683492	-4.531638	3.013463
N	0.153043	0.414396	2.140249
N	-1.458294	2.173162	0.649254
C	0.561202	2.824708	1.890222
C	0.875653	1.567066	2.378133
C	1.841399	-0.004934	3.666759
H	2.450915	-0.561637	4.366553
C	0.311934	-1.871951	3.031075
C	-0.571405	3.108156	1.143328
C	0.730355	-0.554715	2.938887
C	1.940054	1.305942	3.309307
H	2.639224	2.051629	3.665597
C	-2.454950	2.901082	0.028337
C	-1.022942	4.437526	0.836166
H	-0.508443	5.343622	1.129028
C	-2.201857	4.309877	0.164247
H	-2.847465	5.088954	-0.220220
O	-0.163484	-0.080641	-0.853739
H	-4.202747	3.053610	-1.163607
H	1.188421	3.656591	2.193857
H	0.839286	-2.523927	3.719126
H	-3.915724	-3.274777	-0.471303
C	0.528242	-1.073110	-1.377792
C	1.438710	-1.879172	-0.423357
H	2.493386	-1.633639	-0.571646
H	1.201058	-1.567783	0.592161
C	1.260541	-3.396734	-0.598508
H	1.983946	-3.915669	0.041071
H	0.266881	-3.703467	-0.251127
C	1.460070	-3.802023	-2.065389
H	2.484637	-3.535552	-2.359235
H	1.390415	-4.889617	-2.171606
C	0.439044	-3.103416	-3.006695
H	0.944755	-2.741905	-3.909403
H	-0.308679	-3.823654	-3.356714
C	-0.309053	-1.950040	-2.319078
H	-1.086797	-2.359183	-1.661441
H	-0.835425	-1.314495	-3.037998
C	2.350872	0.790227	-1.995525
C	1.717017	2.079589	-1.967381
C	0.491953	2.343898	-2.486917
H	-0.091938	1.537271	-2.920500
C	1.760422	-0.306887	-2.657302
H	1.091895	-0.088688	-3.483132
H	2.389665	-1.172239	-2.836831
C	3.568068	0.595085	-1.201945
C	4.602695	-0.254195	-1.647657
C	3.694251	1.213163	0.059598
C	5.728895	-0.470272	-0.859977
H	4.537961	-0.711249	-2.630228
C	4.806837	0.967826	0.856826
H	2.888260	1.834050	0.435765
C	5.830123	0.133784	0.396936
H	6.528960	-1.107317	-1.224507
H	4.874510	1.422426	1.840500
H	6.705200	-0.044364	1.015093
H	2.273771	2.896215	-1.514835
C	-0.121955	3.696433	-2.554250
H	-0.154360	4.025705	-3.603719
H	-1.158973	3.669002	-2.206757
H	0.428828	4.440188	-1.972869

^{6}TS2F$_{2}$

Fe	-1.118032	0.193491	0.667618
N	-2.853010	-0.895007	0.257502
N	-0.636042	-1.311620	2.033257
C	-2.221588	-3.038292	1.276127
C	-3.029459	-2.244700	0.465985
C	-4.720748	-1.618216	-0.888745
H	-5.606047	-1.570590	-1.509641
C	-4.114928	0.821677	-0.965157
C	-1.127669	-2.601001	2.019339
C	-3.884239	-0.489662	-0.561079
C	-4.191789	-2.703621	-0.255058
H	-4.557399	-3.722391	-0.252682
C	0.444617	-1.318414	2.894364
C	-0.338506	-3.434216	2.890203
H	-0.519397	-4.486641	3.066930
C	0.630610	-2.641500	3.432643
H	1.402533	-2.916645	4.139718
N	0.104027	1.459010	1.800753
N	-2.181398	1.903349	0.112340
C	-0.630790	3.634904	0.922876
C	0.238066	2.822658	1.646742
C	1.882766	2.208309	3.064301
H	2.710093	2.177450	3.761798
C	1.253660	-0.222541	3.177492
C	-1.777846	3.214426	0.255549
C	1.087701	1.068418	2.682888
C	1.359690	3.293375	2.421201
H	1.676042	4.326647	2.484969
C	-3.354269	1.934641	-0.612833
C	-2.723776	4.089588	-0.389251
H	-2.648730	5.169195	-0.412826
C	-3.695385	3.298302	-0.931272
H	-4.576963	3.601631	-1.481239
O	-0.100869	-0.257595	-0.852590
H	-4.995535	1.001324	-1.574149
H	-0.426236	4.701183	0.926173
H	2.072339	-0.382221	3.872236
H	-2.485594	-4.087881	1.360532
C	0.539305	-1.281321	-1.377153
C	1.497549	-2.055612	-0.452953
H	2.538768	-1.771303	-0.627678
H	1.272136	-1.754051	0.569870
C	1.347946	-3.578301	-0.623269
H	2.117625	-4.078619	-0.025347
H	0.381670	-3.901086	-0.216147
C	1.465344	-3.984836	-2.098214
H	2.464101	-3.701034	-2.456174
H	1.410344	-5.073908	-2.195342
C	0.374606	-3.312543	-2.978011
H	0.817846	-2.951121	-3.912646
H	-0.383439	-4.046186	-3.271805
C	-0.345030	-2.158980	-2.259632
H	-1.087261	-2.563304	-1.555250
H	-0.911180	-1.529961	-2.953334
C	2.237335	0.648806	-2.174780
C	1.509761	1.890520	-2.262412
C	0.273093	2.025624	-2.799941
H	-0.250832	1.146097	-3.165359
C	1.766269	-0.525451	-2.785994
H	1.087999	-0.425505	-3.626336
H	2.444785	-1.366690	-2.871549
C	3.414594	0.600687	-1.302968
C	4.527878	-0.207135	-1.616946
C	3.419691	1.324976	-0.092120
C	5.615502	-0.278699	-0.752193
H	4.554643	-0.743810	-2.560404
C	4.498305	1.228637	0.780087
H	2.556635	1.919088	0.189335
C	5.600658	0.434609	0.450364
H	6.476614	-0.885051	-1.016017
H	4.474670	1.773466	1.718535
H	6.448258	0.373324	1.126794
H	2.000585	2.777298	-1.870063
C	-0.443525	3.318483	-2.960173
H	-0.551347	3.545092	-4.031223
H	-1.459112	3.254584	-2.556077
H	0.079745	4.149901	-2.479968

Reaction A(Ru)

$^{2}IA_{Ru}$

Ru	0.931320	0.231595	0.124241
N	1.348200	-1.593592	0.997692
N	1.501422	-0.521956	-1.710855
O	-1.035660	-0.600933	-0.299953
C	2.197427	-2.761522	-0.988394
C	1.872109	-2.680055	0.392717
C	1.592281	-3.289287	2.549697
H	1.553613	-3.806546	3.499369

C	-3.404636	-0.659933	-0.404538
C	0.616052	-1.081269	3.284332
C	2.022350	-1.786644	-1.948098
C	1.153266	-1.921903	2.332404
C	2.035201	-3.757492	1.353078
H	2.434754	-4.736303	1.122355
C	-2.125774	-0.034007	-0.131660
H	-2.143146	0.999819	0.246696
C	1.454269	0.118142	-2.899878
C	2.316763	-1.917700	-3.363626
H	2.732169	-2.802208	-3.828362
C	1.963250	-0.744625	-3.951719
H	2.031753	-0.470990	-4.996439
C	-3.475562	-1.979981	-0.892418
C	-4.714620	-2.554020	-1.140627
C	-4.583336	0.071947	-0.170558
H	-4.521142	1.090327	0.205898
C	-5.822321	-0.509545	-0.420727
H	-6.734707	0.050362	-0.241292
C	-5.885665	-1.819812	-0.904881
H	-6.852795	-2.274071	-1.100380
N	0.332418	1.988747	-0.785410
N	0.180260	0.920883	1.925242
C	-0.508641	3.164137	1.203566
C	-0.204563	3.075755	-0.180231
C	-0.030203	3.637896	-2.360447
H	-0.052398	4.132023	-3.322938
C	0.972996	1.439049	-3.090196
C	-0.343798	2.182755	2.162883
C	0.460620	2.292612	-2.131507
C	-0.440725	4.121440	-1.156171
H	-0.864895	5.091800	-0.933570
C	0.156826	0.251003	3.100047
C	-0.708757	2.285000	3.563441
H	-1.143469	3.161053	4.026798
C	-0.402594	1.091047	4.140961
H	-0.534629	0.792892	5.172768
H	0.527548	-1.478900	4.291176
H	-0.933083	4.106239	1.538513
H	1.009855	1.817106	-4.107798
H	2.614624	-3.706537	-1.324009
H	-4.778919	-3.570436	-1.516605
H	-2.554828	-2.527586	-1.066419
C	2.614068	0.887825	0.482251
O	3.665388	1.308222	0.712795

^{2}TS1A$_{endo,Ru}$

N	-1.683532	-1.115643	-1.503099
N	-2.342867	0.829346	0.575741
C	-3.481011	0.578278	-1.586147
C	-2.733887	-0.475487	-2.125206
C	-1.981523	-2.036419	-3.579126
H	-1.825309	-2.679573	-4.435498
C	-0.123478	-2.927452	-2.129038
C	-3.302505	1.179238	-0.343659
C	-1.208138	-2.074493	-2.373115
C	-2.923126	-1.048319	-3.427036
H	-3.676834	-0.734488	-4.137707
C	-2.503993	1.636334	1.668089
C	-4.084741	2.276648	0.185274
H	-4.906884	2.753355	-0.332820
C	-3.592328	2.558922	1.424539
H	-3.932562	3.310733	2.124890
N	-0.220000	-0.288545	2.209885
N	0.507349	-2.152058	0.115847
C	1.650571	-1.903646	2.267684
C	0.866910	-0.897546	2.822132
C	0.117503	0.671131	4.271034
H	-0.023786	1.329895	5.118176
C	-1.737367	1.564669	2.833058
C	1.488083	-2.482997	1.010044
C	-0.689930	0.685285	3.084311
C	1.071192	-0.297471	4.110053
H	1.853136	-0.581113	4.802604
C	0.670530	-2.959305	-0.988468
C	2.318028	-3.527918	0.448897
H	3.171304	-3.969026	0.947210
C	1.813279	-3.823170	-0.779721
H	2.167722	-4.557475	-1.491410
O	0.286427	0.672063	-0.464212
C	2.645322	0.367560	-0.730690
C	1.262705	0.388560	-1.286898
H	1.067209	-0.411571	-2.012964
C	2.902020	0.927482	0.528563
C	4.185672	0.867890	1.068680
C	3.681559	-0.252064	-1.439943
H	3.478899	-0.700833	-2.410119
C	4.964743	-0.309927	-0.898015

H	5.763912	-0.797066	-1.448960
C	5.219053	0.252172	0.356240
H	6.218522	0.205583	0.778892
H	0.127471	-3.628061	-2.919542
H	2.475890	-2.266371	2.871961
H	-1.978692	2.270081	3.622258
H	-4.284795	0.965698	-2.204828
H	2.083178	1.379498	1.079074
H	4.379517	1.294656	2.048844
C	0.562724	3.590201	-1.151208
C	1.507062	2.917881	-2.015102
C	1.191007	1.724291	-2.685487
H	0.162082	1.522051	-2.965898
H	1.920017	1.358368	-3.404013
C	2.933068	3.376145	-2.012142
H	3.003734	4.452136	-2.208266
H	3.372849	3.204697	-1.018754
H	3.539980	2.840770	-2.743737
C	1.032712	4.748461	-0.297773
H	1.864930	4.458929	0.354210
H	1.381403	5.585948	-0.913296
H	0.222517	5.118316	0.335316
C	-0.710073	3.126741	-1.036654
H	-1.120925	2.345243	-1.658974
H	-1.396485	3.550019	-0.309579
Ru	-0.959072	-0.703588	0.375627
C	-2.216739	-1.962400	1.104014
O	-2.974537	-2.713295	1.518411

^{2}TS1A$_{exo,Ru}$

N	-1.397646	-0.114348	1.592523
N	-0.918277	2.206373	-0.075572
C	-3.094241	1.490178	0.851430
C	-2.685397	0.346414	1.533123
C	-2.760093	-1.528481	2.793034
H	-3.052560	-2.367790	3.410589
C	-0.287193	-2.023812	2.668659
C	-2.284431	2.342622	0.105808
C	-1.405238	-1.254951	2.364006
C	-3.547534	-0.543261	2.282075
H	-4.616342	-0.414617	2.391572
C	-0.511457	3.229012	-0.924216
C	-2.740444	3.483460	-0.637096
H	-3.768143	3.821001	-0.675188
C	-1.654025	4.024878	-1.271070
H	-1.627658	4.886558	-1.925521
N	1.905452	1.554869	-0.284835
N	1.448146	-0.724744	1.478289
C	3.623032	0.028484	0.577772
C	3.203128	1.117588	-0.177653
C	3.256221	2.912076	-1.553256
H	3.541378	3.722216	-2.211835
C	0.783034	3.429949	-1.393785
C	2.814761	-0.834968	1.323551
C	1.906801	2.657778	-1.094531
C	4.056162	1.968380	-0.981886
H	5.127854	1.851079	-1.079858
C	1.030822	-1.781524	2.264060
C	3.268512	-2.001954	2.023234
H	4.298408	-2.332021	2.073219
C	2.166662	-2.589696	2.597731
H	2.131967	-3.485597	3.204260
O	-0.056981	-0.379845	-0.961624
C	-2.156173	-1.464278	-1.329244
C	-0.715011	-1.510140	-0.945954
H	-0.496895	-2.196089	-0.118126
C	-2.650353	-0.389780	-2.081004
C	-4.009041	-0.316643	-2.375720
C	-3.028469	-2.465100	-0.882242
H	-2.644017	-3.290289	-0.286984
C	-4.388886	-2.386083	-1.173850
H	-5.065373	-3.156335	-0.815455
C	-4.880000	-1.313364	-1.923247
H	-5.939827	-1.253734	-2.153507
H	-0.449180	-2.899217	3.290093
H	4.686412	-0.189646	0.572238
H	0.933321	4.279347	-2.052935
H	-4.151714	1.731304	0.886857
H	-1.966556	0.389733	-2.401164
H	-4.392510	0.520358	-2.952198
C	2.373745	-1.819773	-2.305175
C	1.332945	-2.788745	-2.027994
C	-0.005343	-2.596266	-2.398634
H	-0.241871	-1.932843	-3.223891
H	-0.662856	-3.458058	-2.329547
C	1.669733	-3.964193	-1.158959
H	2.503527	-4.538115	-1.580466
H	2.001074	-3.620876	-0.167549

H	0.818154	-4.635361	-1.029476
C	3.808430	-2.167742	-1.972333
H	3.929812	-2.412558	-0.912593
H	4.150433	-3.033203	-2.552489
H	4.471522	-1.329871	-2.199388
C	2.057565	-0.590825	-2.789111
H	1.053912	-0.297084	-3.061717
H	2.822064	0.172413	-2.898422
Ru	0.263815	0.755438	0.696430
C	0.644225	1.830226	2.251336
O	0.879402	2.454612	3.180446

^{4}IIA under OEEF = -0.0015 AU

Fe 0.4019371583 -0.3114581438 -0.3180945268
N 2.395488636 -0.3648487188 -0.5968070515
N 0.5002765013 1.6671887366 -0.0327941517
O 0.1032500685 0.1042553185 -2.5320756471
C 2.9291115417 2.0117706312 -0.2439643012
C 3.2764362902 0.6956640195 -0.5089533828
C 4.558422973 -1.1095370499 -0.918595119
H 5.3690085084 -1.8037851052 -1.0995998433
C 0.5066098492 -0.2727199427 -4.8432420114
C 2.6991440144 -2.7753387529 -0.9949280259
C 1.6338772609 2.458069841 -0.0308075845
C 3.1730471774 -1.4807724899 -0.8402883856
C 4.6223375491 0.237653192 -0.7134790896
H 5.4960161288 0.8766035226 -0.69291448
C 0.7491299377 -0.4618394418 -3.415848984
H 1.5602733013 -1.1540087449 -3.1389444352
C -0.5568137476 2.5262028964 0.1935441497
C 1.2800768313 3.8272675607 0.2220185215
H 1.9904031263 4.6419622137 0.2818261503
C -0.0763916904 3.8696327786 0.3584527397
H -0.7082482994 4.7254887315 0.557781525
C -0.5249251565 0.5712888767 -5.2934238724
C -0.7355111979 0.7321153568 -6.6571318837
C 1.320385256 -0.948698023 -5.7667389046
H 2.1152369562 -1.5982084178 -5.4081303801
C 1.1064461812 -0.7823577709 -7.1328185098
H 1.7341805504 -1.3020483284 -7.8506521336
C 0.0795869799 0.056493004 -7.5749771729
H -0.0893189793 0.1857582306 -8.6407476358
N -1.5840426492 -0.2487943504 -0.1202173042
N 0.3076099412 -2.2839489964 -0.682655304
C -2.114582077 -2.6315198321 -0.4291035057
C -2.4608682735 -1.3134619515 -0.1797084008
C -3.7431745221 0.488228648 0.2420951688
H -4.5502292165 1.1789532001 0.4490624847
C -1.8919941292 2.1606687768 0.2675331607
C -0.8222988573 -3.0757644721 -0.6620313222
C -2.3609226243 0.8644340456 0.1296486171
C -3.8047393273 -0.8601411608 0.0518720528
H -4.6736550619 -1.5054559837 0.0679398708
C 1.3642058772 -3.1422208793 -0.914557987
C -0.4698880058 -4.4476465168 -0.9030252657
H -1.1792961466 -5.2646744038 -0.9355135201
C 0.8839223736 -4.4890078471 -1.0601225328
H 1.5155948643 -5.3476484496 -1.2484971543
O 0.7109815814 -0.7830475163 1.7646150273
C 1.8547936427 -0.6761948515 3.8400171109
C 1.4315785256 -0.1533802367 2.554772365
H 1.8107294616 0.8367117966 2.2625771814
C 1.3534366875 -1.9012507431 4.319970912
C 1.7861468742 -2.3842157553 5.5484390754
C 2.7821543722 0.0567283379 4.6011039559
H 3.1527346911 1.0082316094 4.2273773114
C 3.2205173916 -0.4380476557 5.8254194896
H 3.9404616065 0.1211863722 6.4148522161
C 2.7223398946 -1.6571150401 6.296689314
H 3.0603417024 -2.0412397124 7.2550304016
H -1.5312144603 1.38072976 -7.0116592557
H -1.1432564551 1.0840084839 -4.5632134153
H 0.623741251 -2.4408932993 3.7260560698
H 1.4006497799 -3.3247141559 5.9304206444
C -1.9733489856 -0.2037526859 4.6072146478
C -0.9918115374 0.9102810206 4.7380719309
C -0.7785710441 1.7803967006 3.7365461106
H -1.347493982 1.7369077462 2.814821317
H -0.0521069933 2.5849547258 3.8269752108
C -0.2508328823 1.0277302811 6.0480108283
H -0.9393015492 1.2339547434 6.8770095951
H 0.2721309568 0.0951929993 6.2910114516
H 0.4863128038 1.8353989496 6.0185600044
C -2.7456108112 -0.5952519841 5.8465281077
H -2.0801591015 -0.9838372332 6.6278548743
H -3.2729955639 0.2640411879 6.2802659095
H -3.4846745858 -1.3705319604 5.6227894909
C -2.1477555534 -0.8538140129 3.4449520795

H -1.5667839431 -0.6243043325 2.5595236004
H -2.8758388803 -1.6567100894 3.355843137
H 3.4269005523 -3.5590299901 -1.1763404344
H -2.6185925753 2.9418584486 0.462400591
H 3.7275295072 2.7453370031 -0.2050697779
H -2.9106692503 -3.3684147188 -0.4375757099

^{4}TS1A$_{endo}$ under OEEF = -0.0015 AU

Fe -0.0284841012 -0.010235022 0.0063750135
N 1.9862033984 -0.0328028274 -0.1294474318
N -0.0062260695 1.9991859189 -0.1230078695
O -0.0633916793 0.0355249858 -2.3484895742
C 2.4204281598 2.3871903873 -0.2180624064
C 2.8254599858 1.060322573 -0.1855380798
C 4.1873186684 -0.7306149664 -0.078543968
H 5.0275080315 -1.4112607809 -0.027642153
C 0.7742539155 -0.6306452445 -4.4746240192
C 2.3815184287 -2.4571101636 -0.0030473545
C 1.1005748285 2.8167562643 -0.183253348
C 2.8083854048 -1.1385266973 -0.0610952407
C 4.1981984311 0.6301569538 -0.1619011869
H 5.0497019142 1.2980315667 -0.1929484049
C 0.6779578595 -0.684233799 -3.0144218802
H 1.3248110975 -1.4208085586 -2.5094166639
C -1.0985007183 2.8381975019 -0.0954949473
C 0.6944413871 4.1978724271 -0.1884839416
H 1.377169948 5.0371564589 -0.2285574124
C -0.6673149283 4.2111277157 -0.1291051601
H -1.3346760082 5.0635017214 -0.1143041484
C 0.0063588361 0.2858852754 -5.2140712143
C 0.1213219984 0.3215951436 -6.5985654561
C 1.6532706866 -1.5056632482 -5.1318534964
H 2.2435027775 -2.2104221926 -4.5506880387
C 1.7653283775 -1.4667173866 -6.5199272866
H 2.4451641613 -2.142100701 -7.0311021479
C 0.9997913547 -0.5537937778 -7.2503452108
H 1.0871989176 -0.5218125508 -8.3331373933
N -2.0337328305 0.0044467115 -0.0901138162
N -0.0386704799 -2.0229079656 -0.0926438703
C -2.4662095978 -2.416029941 -0.1036604654
C -2.8710295751 -1.0888294142 -0.1000141524
C -4.2326779795 0.7053217433 -0.0640298106
H -5.0728904044 1.3878962229 -0.0478579923
C -2.4252702292 2.4327251703 -0.0600912243
C -1.146327444 -2.8422399724 -0.0874116556
C -2.8524996511 1.1123112478 -0.0733536979
C -4.2443835992 -0.6583050125 -0.0846215634
H -5.0962238101 -1.3265499879 -0.0887308349
C 1.054249982 -2.8614112059 -0.0243120252
C -0.7401886993 -4.2219875219 -0.0306053092
H -1.4237261255 -5.0614747397 -0.0175088274
C 0.6221560472 -4.2335359964 0.0141797764
H 1.2896005954 -5.0841021255 0.0697829276
O -0.0095989833 -0.0157102246 2.001410143
C 1.8832309575 -0.4519154843 3.4072502257
C 0.9261056597 0.49821156 2.7554249418
H 1.4191528665 1.3971926658 2.3581341128
C 1.5909884038 -1.8208975021 3.4513996405
C 2.4953690114 -2.7104321004 4.031632229
C 3.088241265 0.0160677019 3.9447979909
H 3.3203316799 1.0783422448 3.9025228928
C 3.9913759245 -0.8735190994 4.5257245356
H 4.9252518869 -0.5042710773 4.9403126751
C 3.6951399789 -2.2389306952 4.5718652753
H 4.3979419341 -2.9319875696 5.025767295
H -0.4685312273 1.02837643 -7.175149719
H -0.6659670369 0.9552462063 -4.6864122767
H 0.6633679983 -2.1718099017 3.0110979896
H 2.2658725737 -3.7723656949 4.0603967515
C -1.843868396 -0.0724716248 4.3977697346
C -0.6294779366 0.5444733534 4.8813934248
C 0.0737660313 1.5020125894 4.1243688287
H -0.4782050694 2.1602467116 3.4595128606
H 0.9053060134 1.9935318329 4.6237194656
C 0.0216734257 -0.0211815714 6.1026596353
H -0.6809384208 -0.0497371978 6.9442169761
H 0.3266871659 -1.0610938278 5.91496657
H 0.9068276149 0.54350907 6.3996317749
C -2.4378365652 -1.2387108214 5.1557991069
H -1.7235493744 -2.0653715821 5.2487040104
H -2.7302782332 -0.9513098043 6.172807197
H -3.3288698224 -1.6182762855 4.6486557333
C -2.3818704146 0.3217302356 3.2130000083
H -2.0470819453 1.1887126197 2.6636388982
H -3.2187952698 -0.215288199 2.7758836627
H 3.1412003805 -3.2287348294 0.0619837442
H -3.186238595 3.2060517389 -0.0412408216
H 3.1942009782 3.1468509503 -0.2576572044

H -3.2397957226 -3.1769250077 -0.1000553461

⁴IIIA$_{endo}$ under OEEF = -0.0015 AU

Fe -0.1196274768 0.0198050113 -0.0093834164
N 1.8727330033 -0.153936186 0.0351016134
N 0.0571466685 2.0132784972 -0.0328668032
O -0.0292485911 0.0755865696 -2.2800976098
C 2.508832526 2.2218071655 -0.0495564923
C 2.8020794445 0.8691463799 0.0162684531
C 4.0043374579 -1.0221361623 0.2306099509
H 4.7823508585 -1.7641994345 0.3528321762
C 0.7342903537 -0.7281761031 -4.383498417
C 2.074447599 -2.6021333022 0.1981507089
C 1.225626509 2.7486455803 -0.0585327849
C 2.6015366367 -1.3208540783 0.1625312712
C 4.12927382 0.3316660516 0.1294100529
H 5.0317924415 0.9285806493 0.1503792267
C 0.6242992586 -0.7448959297 -2.9280861151
H 1.166086874 -1.5465048477 -2.4012645324
C -0.9688961702 2.936797011 -0.0081855784
C 0.9249322173 4.1534305994 -0.0444517672
H 1.6695394716 4.9392271952 -0.0550434261
C -0.4332872453 4.2698034791 -0.0018840947
H -1.0326234275 5.1707537153 0.0238802419
C 0.092460974 0.2647592787 -5.1458565561
C 0.2176049976 0.2565511469 -6.529391638
C 1.4969645379 -1.7230219551 -5.0167803133
H 1.9889815137 -2.4856215088 -4.4179349708
C 1.6181249789 -1.7275321367 -6.4041384338
H 2.206285272 -2.4955179086 -6.8975876456
C 0.9791232667 -0.7385164955 -7.156891243
H 1.0740759971 -0.7405306946 -8.2394436839
N -2.1066989703 0.1886153013 -0.1619880977
N -0.2911364446 -1.9788963825 -0.0655164887
C -2.7294653294 -2.186861221 -0.3231805809
C -3.0264825584 -0.8328158828 -0.2969121414
C -4.2448930472 1.0595621123 -0.2313057174
H -5.0308356678 1.8039300901 -0.2209928339
C -2.3221301504 2.6374565784 -0.0286183826
C -1.453946252 -2.7149051059 -0.1913690921
C -2.843360681 1.3566331271 -0.1263377754
C -4.3575472752 -0.2947154037 -0.347357611
H -5.2556706583 -0.8904158928 -0.4478034022
C 0.7262418248 -2.9029995555 0.079927911
C -1.158133731 -4.1192264919 -0.1245056827
H -1.8996600349 -4.9049902357 -0.1926330528
C 0.1896230567 -4.2351916012 0.0523846339
H 0.7806982035 -5.1362000175 0.1547688736
O -0.2197120443 -0.0308063356 2.1763785878
C 1.9284466616 -0.0877789003 3.3394497523
C 0.7102675213 0.7414295731 2.9832066354
H 1.0193986614 1.5733267694 2.346904876
C 1.8806272336 -1.4816913323 3.4352497325
C 3.013476608 -2.2032884662 3.8164551678
C 3.1316054052 0.5716286788 3.6216256471
H 3.1806035783 1.6556469246 3.5404569529
C 4.263894068 -0.1469530189 4.0044370899
H 5.1894512869 0.3788964014 4.2228643717
C 4.2070415291 -1.5394145446 4.1043670025
H 5.0869218412 -2.1015826255 4.405159614
H -0.2737186112 1.0207282198 -7.1245979309
H -0.4912598988 1.0252439856 -4.6367087317
H 0.9595752675 -2.0011314027 3.1973822322
H 2.9609330266 -3.2864820726 3.8902490369
C -1.3676100092 -0.7056776015 4.2859180469
C -0.6662499431 0.1858269549 5.0052826569
C -0.0141229045 1.3019719554 4.2185070217
H -0.7624586049 2.0328319582 3.8777707372
H 0.713487558 1.8499818521 4.8246852939
C -0.4081233241 0.148593597 6.4828268149
H -0.9509843823 -0.6463786444 6.9988064494
H 0.6641846258 0.006494481 6.6760249358
H -0.6868828675 1.1036194549 6.9478310341
C -2.0913111749 -1.9252300282 4.7867226113
H -1.6493241088 -2.8401537999 4.3667520397
H -2.0717784658 -2.0148444006 5.874834741
H -3.1450413113 -1.9135529754 4.4740982827
C -1.4688966022 -0.4669071362 2.8016079701
H -2.2257813877 0.2934907127 2.5701459454
H -1.7392642818 -1.3803919901 2.2699806473
H 2.7681596459 -3.4273615985 0.3169158467
H -3.0226470608 3.4655711207 -0.0024488769
H 3.3400475605 2.9189682116 -0.0576341303
H -3.5562221471 -2.8831669835 -0.4161108372

⁴VA under OEEF = -0.0015 AU

Fe -0.0131547832 -0.0161570467 -0.0178257316
N 1.954649789 -0.0146137872 -0.3575685523

N -0.030080206 1.9736105839 -0.212366943
C 2.3769286427 2.4092518011 -0.4721975509
C 2.7852427469 1.0875033506 -0.487305877
C 4.1576823893 -0.6936185877 -0.5671097857
H 5.0017528896 -1.3683504798 -0.6284467058
C 2.3944823189 -2.4382654209 -0.3548335797
C 1.0611777169 2.8155972681 -0.3416520321
C 2.7953878516 -1.114739991 -0.4152426494
C 4.1508143159 0.6692093933 -0.6177060569
H 4.9882498836 1.3463817068 -0.7242829286
C -1.1253405481 2.8108665429 -0.0854031359
C 0.641858245 4.1865642503 -0.2967670988
H 1.3126168408 5.0323379439 -0.3751416248
C -0.7116254436 4.1837068203 -0.1323138098
H -1.385357321 5.0270221021 -0.0525933347
N -2.0144617051 -0.0191816706 -0.0586384959
N -0.0245145041 -2.0102900561 -0.2068887313
C -2.4445177383 -2.4421479048 -0.0649344669
C -2.8513066668 -1.1200688704 -0.0201739598
C -4.2179512716 0.6589887852 0.14208012
H -5.0591380486 1.3342517972 0.230876923
C -2.4403805887 2.4012700587 0.0483997203
C -1.1249915058 -2.8505608714 -0.1529837913
C -2.8491059484 1.0792253157 0.0459855556
C -4.2198022183 -0.7031884568 0.0957920741
H -5.0626024767 -1.3808485223 0.1412644502
C 1.0753492054 -2.850353455 -0.2754822802
C -0.7062286258 -4.2216872779 -0.1895640117
H -1.3819833175 -5.0668508741 -0.1620744938
C 0.6557912045 -4.2218488905 -0.2621072168
H 1.3305435106 -5.0671059896 -0.3068529129
O -0.0173406527 -0.0084484165 2.0516160039
C 0.0632748208 -0.887345154 4.2503586008
C 0.3463629176 -0.9090415057 2.8343127928
H 0.8689161895 -1.7871787538 2.4316653957
C -0.5361073647 0.2377250754 4.8519629139
C -0.8004826448 0.2241197308 6.2148256833
C 0.3945534953 -2.0149863052 5.0253220572
H 0.8650829228 -2.8734077011 4.5525616283
C 0.1159027383 -2.0254801717 6.3873534213
H 0.3638140264 -2.8940647969 6.9893283993
C -0.479916512 -0.9067400094 6.9795308419
H -0.6932690095 -0.9125325181 8.0446547113
H 3.1626430242 -3.2026118195 -0.4002351952
H -3.2102180246 -3.2091735957 -0.0236045307
H -3.2025283016 3.167045326 0.1434863854
H 3.1387527171 3.1757865837 -0.5607719618
H -0.7690467609 1.1038743 4.2419265595
H -1.2565339678 1.0872125992 6.6899185532
C 2.6876211378 2.0531492713 4.0786485163
C 3.0833596923 0.6177999325 4.1446774263
C 3.347208401 -0.0902134197 3.0329672862
H 3.3368198068 0.3687617807 2.0517396263
H 3.6285398271 -1.139672607 3.0818146621
C 3.1815175993 -0.0081763658 5.5145130736
H 3.978886102 0.4572749373 6.107097922
H 2.2485392324 0.1219807709 6.0754022399
H 3.3963691821 -1.0787384208 5.4505835595
C 3.1990546872 2.9628225661 5.1720055999
H 2.8012123716 2.6726803752 6.1526502812
H 4.293006442 2.9201494033 5.2504065253
H 2.9114590805 4.0026072919 4.9892980525
C 1.8823841658 2.5123550787 3.1069836047
H 1.4648913048 1.8629004481 2.3468761771
H 1.600836721 3.5617565225 3.0580621016

^{4}TS2A$_{endo}$ under OEEF = -0.0015 AU

Fe 0.1122447466 -0.1282213468 -0.0116504511
N 2.0486753727 0.3084542178 -0.3353353882
N -0.3210784663 1.8298170456 -0.1269196874
C 1.9500576322 2.7653528641 -0.2741675717
C 2.6313935613 1.5618622674 -0.3482150461
C 4.3502543996 0.1144821803 -0.4658217544
H 5.3185588365 -0.3652006577 -0.5287321088
C 2.9789812518 -1.9679548087 -0.4268214591
C 0.5734328825 2.8819133881 -0.1716120886
C 3.098059415 -0.587942229 -0.4069490026
C 4.061215145 1.445739927 -0.4337701124
H 4.7433477859 2.285807828 -0.4625668775
C -1.5693948194 2.4091052105 0.0001210183
C -0.1247501825 4.1332696539 -0.0687064103
H 0.3533867119 5.1043202382 -0.0838978516
C -1.4515520041 3.8399684574 0.044450133
H -2.2876651818 4.5208413198 0.1393335188
N -1.8524858361 -0.5452175438 -0.1670848238
N 0.5226937353 -2.0686092114 -0.3332052819
C -1.7554244225 -2.9995934514 -0.2736165475
C -2.435994448 -1.7962241345 -0.1964849836

C -4.1517457919 -0.3540823078 -0.0015553893
H -5.118312838 0.1241469977 0.0920325445
C -2.7730766057 1.7246391083 0.0531214057
C -0.3773631808 -3.1189417782 -0.3404726926
C -2.8978673177 0.3483448539 -0.0394486216
C -3.8655812049 -1.6822065561 -0.100741468
H -4.5478348581 -2.5224433359 -0.1017434839
C 1.7742323613 -2.6523270664 -0.4058027592
C 0.3213396027 -4.3709064031 -0.4159744015
H -0.1608406594 -5.3397304761 -0.435550038
C 1.6533012069 -4.0826224799 -0.4565718202
H 2.4901742087 -4.7665319669 -0.5173805005
O 0.1722256617 -0.2063250654 1.9311615727
C -0.8974717485 -1.8313119571 3.3311455829
C 0.3240741967 -1.2366718453 2.7166636449
H 1.0674967078 -1.9805616432 2.4008961395
C -2.118587641 -1.1484724108 3.2604746711
C -3.2695969407 -1.7183698973 3.8033686825
C -0.8390493965 -3.0871192165 3.9498939805
H 0.1072718081 -3.6219310656 3.996980254
C -1.9895502231 -3.6524300011 4.497515338
H -1.9394825545 -4.625525327 4.9776754028
C -3.2069775944 -2.9681090286 4.4260438925
H -4.103144648 -3.4099846585 4.8523401789
H 3.891856292 -2.5517578279 -0.4805935601
H -2.3411741393 -3.9125186475 -0.2760585423
H -3.6820287792 2.3073588787 0.1590001613
H 2.5337786027 3.6796905461 -0.2918568823
H -2.1543850233 -0.1882645084 2.7574786081
H -4.2166602495 -1.1902304153 3.7352920579
C 0.4723310861 1.6249671314 4.3261674106
C 0.7271564126 0.2938767769 4.8430377081
C 1.5071233806 -0.6434615906 4.1501067148
H 2.2966486671 -0.3038713733 3.4870459086
H 1.7344568751 -1.5715550838 4.668107374
C -0.0388822565 -0.1626442504 6.0433473153
H 0.1123919294 0.5252974594 6.8846487883
H -1.1168489698 -0.15996014 5.8283318789
H 0.2452542264 -1.1683115325 6.3568522587
C -0.5402188638 2.5039780396 5.025919761
H -1.5267710759 2.0277298913 5.0695481741
H -0.2428265311 2.7183804774 6.0591094632
H -0.6499703919 3.4598084172 4.5066772528
C 1.0612144542 2.0456827053 3.1773015651
H 1.8315376937 1.4936486136 2.6567099212
H 0.7866479944 2.9990987449 2.7359773233

^{6}TS2A$_{endo}$ under OEEF = -0.0015 AU

Fe 0.0704927257 -0.0116850953 0.0153558339
N 2.0675076437 -0.1874581563 -0.5561496762
N 0.161425932 1.9784647985 -0.5818935743
C 2.5990832839 2.1999586821 -0.8434280143
C 2.9508643893 0.8541702941 -0.7561507301
C 4.2112039141 -1.0189684568 -0.7268233796
H 5.014798251 -1.7441913628 -0.751983369
C 2.3109381763 -2.6299219982 -0.3628806356
C 1.3077009387 2.7201844868 -0.77499287
C 2.8201315491 -1.343641049 -0.5393972859
C 4.2917046476 0.3375330571 -0.8616400351
H 5.1750699721 0.9432656129 -1.0195077131
C -0.8906186683 2.8699638076 -0.5819511017
C 0.9675294022 4.1156309773 -0.8881299135
H 1.6828791621 4.9142453019 -1.0387748579
C -0.3899912885 4.2080178853 -0.7699797724
H -1.0067256482 5.0972297811 -0.80673497
N -1.9887527976 0.0919110161 -0.2270608444
N -0.0805055599 -2.0747855849 -0.2110823974
C -2.5197905657 -2.2972529379 0.0353811501
C -2.8730162027 -0.9524181257 -0.0616621037
C -4.1402524461 0.9141505932 -0.1314334784
H -4.9468435032 1.6363874569 -0.1302016686
C -2.2337284584 2.5308152051 -0.4275002835
C -1.2294899857 -2.8171296356 -0.035155342
C -2.7436436361 1.2438154639 -0.2704981101
C -4.2199112808 -0.4420091451 -0.0021128642
H -5.1048875171 -1.0520593871 0.1265964506
C 0.9679268607 -2.9696877871 -0.2161691937
C -0.8925217389 -4.214701044 0.0697169314
H -1.6103205168 -5.01173655 0.2158789464
C 0.4639411064 -4.3091454646 -0.0414618283
H 1.0789129318 -5.1994194386 -0.0050300789
O 0.2366469831 0.070420073 1.901627416
C 0.3416679197 -1.5322928532 3.6802004112
C 0.9969780611 -0.6528374044 2.6871840913
H 1.9024855825 -1.0689042104 2.2312032273
C -1.0267713514 -1.3946357276 3.9594360941
C -1.6350625519 -2.2383686616 4.8868215115
C 1.0912246988 -2.5196662867 4.3374148763

H 2.1518915358 -2.6266414299 4.1207745855
C 0.4802237126 -3.3583109967 5.2665398625
H 1.0630982509 -4.1198763331 5.7759195168
C -0.8832389521 -3.2170758018 5.5441819422
H -1.3576747244 -3.8681825279 6.2724843191
H 3.0282024715 -3.444979389 -0.3518238614
H -3.3273847437 -3.0082958775 0.1784043929
H -2.9511513217 3.3457035554 -0.4392338717
H 3.4065920708 2.9101337053 -0.9923115384
H -1.5995454905 -0.6354516234 3.436294886
H -2.6947115367 -2.1329108255 5.1012712097
C 0.3172396033 2.3557066559 3.9745329292
C 1.2157357885 1.3261509344 4.4837015355
C 2.1509463101 0.6763886168 3.6741242188
H 2.5202939087 1.1521794987 2.7714776063
H 2.8642240608 0.0180462301 4.1623968451
C 0.999879485 0.8191110748 5.8716019012
H 1.0264943946 1.6421737888 6.5971632285
H 0.0017013616 0.3657389111 5.9561669199
H 1.74140607 0.0730894313 6.1600724744
C -0.790933932 2.8678074367 4.8654840142
H -1.4543385463 2.0592653303 5.1934035402
H -0.3939384933 3.3415437623 5.7706068942
H -1.3980290124 3.6107170443 4.3409243756
C 0.4334028334 2.8064559847 2.7026159849
H 1.2317751515 2.5122094947 2.0355146922
H -0.2793806705 3.518915219 2.2990705483

^{4}VIA$_{endo}$ under OEEF = -0.0015 AU

Fe -0.0133057602 0.0053888438 0.0098110029
N 1.9799283013 -0.0068507294 -0.1845387866
N 0.0031998677 1.9972178581 -0.1904990671
C 2.4269090564 2.408996878 -0.0407020349
C 2.8274663472 1.0856941414 -0.1018883222
C 4.186957485 -0.7005929057 -0.2530297543
H 5.027616188 -1.3797251013 -0.3137425668
C 2.3911879324 -2.4272291937 -0.3447333689
C 1.109890926 2.8283606957 -0.1138481773
C 2.8128594231 -1.1100183234 -0.2780378725
C 4.1960092007 0.6581143988 -0.1332941737
H 5.0457549892 1.3265802751 -0.0818074551
C -1.0865359575 2.8480780315 -0.2845878773
C 0.7047487512 4.2031922487 -0.1555942857
H 1.3870519947 5.0422370256 -0.1103128526
C -0.6539852367 4.2152114633 -0.2727264102
H -1.3194825342 5.0663988351 -0.3371709663
N -2.0019563789 0.0294953732 -0.204680227
N -0.0250637819 -1.9719890655 -0.2317334416
C -2.4487957086 -2.3873282182 -0.1135050032
C -2.8500332755 -1.0636742589 -0.151389363
C -4.208386623 0.7250320234 -0.2783319674
H -5.0482293557 1.4057382582 -0.3326105587
C -2.412118433 2.4521858528 -0.3343302397
C -1.1313745618 -2.803340316 -0.1763699909
C -2.8333242033 1.134668274 -0.2843715408
C -4.2186383544 -0.6352953506 -0.1852952428
H -5.0686174059 -1.3046802274 -0.1532569236
C 1.066234823 -2.8217587531 -0.312200689
C -0.7254892306 -4.177905326 -0.2181419933
H -1.407912528 -5.0163671153 -0.1743189572
C 0.6335298166 -4.1894077169 -0.3138356681
H 1.3009633341 -5.0398564025 -0.3620507265
O -0.0420844828 -0.0159297889 2.1022550058
C 0.2518176328 -2.2473836978 3.0532239487
C 0.8708556918 -0.8812502665 2.8492928548
H 1.7587872253 -0.9766624626 2.2200095522
C -1.1274844831 -2.4294856898 3.1949890338
C -1.6471808608 -3.6987517743 3.4564298832
C 1.1013319029 -3.3550950822 3.1671329259
H 2.1747171714 -3.2230509043 3.049336331
C 0.5837378019 -4.622194207 3.4343525132
H 1.2550904384 -5.4715977533 3.5257117007
C -0.7944532028 -4.7969431577 3.5810436301
H -1.2000342762 -5.7826184215 3.7915935539
H 3.1491318868 -3.2008090945 -0.4034156822
H -3.2181252487 -3.1499119858 -0.0638249505
H -3.1703526271 3.2254049657 -0.3946422612
H 3.1976346339 3.1694233516 0.0228475307
H -1.7917962426 -1.5790007535 3.094359843
H -2.7205630068 -3.8269834671 3.5679444293
C -0.9137132441 0.8718792792 4.2439684769
C 0.0209991055 0.2074944678 4.9454967472
C 1.2575917978 -0.1969680682 4.1716760008
H 1.8804971972 0.6799417619 3.9415663871
H 1.8819559096 -0.8918331316 4.740599164
C -0.067525253 -0.2315975675 6.376954249
H -0.9563736124 0.1412032377 6.8908524548
H -0.076741739 -1.3287133845 6.4349668565

H 0.8139921662 0.104597709 6.9386988862
C -2.2506149339 1.3630865018 4.7262969977
H -3.0670628329 0.8610294543 4.1876124398
H -2.4018418859 1.1939849819 5.7944004656
H -2.3665038638 2.4398547723 4.5409844187
C -0.5953535191 1.1548823339 2.8009537546
H 0.1333955843 1.9669776073 2.6856085788
H -1.488349938 1.4149287614 2.2325497817

^{6}VIA$_{endo}$ under OEEF = -0.0015 AU

Fe -0.0363753302 -0.0052223289 0.1211893486
N 1.9987726798 -0.054684699 -0.2041674189
N 0.0208711183 2.0333707981 -0.1923172886
C 2.4624860744 2.3624809826 -0.1334196681
C 2.8576698598 1.0279575677 -0.1671814077
C 4.1817376621 -0.796726706 -0.2675721145
H 5.0128782906 -1.4890883964 -0.3110568069
C 2.3254053594 -2.4921026085 -0.317366715
C 1.1514482479 2.8300808294 -0.167550606
C 2.7960804369 -1.1820897192 -0.2732286193
C 4.2197455692 0.5672355833 -0.1978112999
H 5.0882291481 1.2131431802 -0.175734654
C -1.0590680297 2.8947301969 -0.2609852717
C 0.7697193641 4.2158668 -0.2084796946
H 1.4648711154 5.0457153009 -0.1972291258
C -0.5942292318 4.2555962371 -0.2721934827
H -1.2379420975 5.1246521447 -0.3198703637
N -2.0689826355 0.0669114248 -0.2035330937
N -0.0908698469 -2.0208602417 -0.2585692102
C -2.5308314219 -2.351870647 -0.2039547387
C -2.9270636721 -1.0168901027 -0.2037198314
C -4.2520277754 0.8081281068 -0.2789452413
H -5.0832949554 1.5005694186 -0.3181513708
C -2.3958805102 2.5048585587 -0.2863055688
C -1.220522286 -2.8174587267 -0.2416942683
C -2.8666281174 1.1947514097 -0.2597224232
C -4.2891978877 -0.5568354439 -0.2386476025
H -5.1568662261 -1.2041809223 -0.2415539851
C 0.9898732508 -2.881495085 -0.3082448559
C -0.8378908869 -4.203276018 -0.275798812
H -1.5326787148 -5.03286801 -0.2584324067
C 0.5256254384 -4.2429066794 -0.3207600316
H 1.1708048382 -5.1114907063 -0.345821941
O -0.0189763224 0.0186166047 2.1569365054
C 0.3605306219 -2.2097467757 3.0901393101
C 0.9214922205 -0.815416874 2.9212311009
H 1.8272354898 -0.8551398643 2.3108356535
C -1.0127505264 -2.4605069102 3.1628020497
C -1.4794739965 -3.7558508633 3.3951855553
C 1.2600243021 -3.2731224772 3.2382598346
H 2.3297063641 -3.0853552056 3.1730075112
C 0.7944380695 -4.5656766851 3.4764075995
H 1.5024368664 -5.3809778009 3.5965743259
C -0.5787964091 -4.809524397 3.5579426133
H -0.9431164894 -5.8157665394 3.7450721513
H 3.068454637 -3.2823379713 -0.3545340743
H -3.317936596 -3.0990581366 -0.1953177822
H -3.1385998817 3.2951714527 -0.3298333259
H 3.2502604304 3.1088530034 -0.1103467221
H -1.7137949713 -1.6441232867 3.0322799644
H -2.5490034514 -3.9392853568 3.4516729988
C -0.984893513 0.8522762031 4.281242727
C -0.0387393334 0.2226248861 5.0000818471
C 1.2352754145 -0.1173918849 4.255450711
H 1.8193850411 0.7902554126 4.0439452789
H 1.8801164984 -0.782334867 4.8371042068
C -0.1436909871 -0.2311064909 6.4256867668
H -1.0585113759 0.102918796 6.9200731907
H -0.1098441073 -1.327911171 6.4771321971
H 0.7098769847 0.1371408653 7.0097946229
C -2.3557573167 1.2768345429 4.730325473
H -3.1331614687 0.7432654572 4.1649493088
H -2.5270668854 1.090018701 5.7922507879
H -2.5160390661 2.3489064806 4.5517276888
C -0.6429775354 1.1671244037 2.8521013148
H 0.0606885983 2.002267969 2.7567311311
H -1.5302201333 1.3928372812 2.2606180478

^{4}IIA under OEEF = -0.003 AU

Fe 0.4121553555 -0.2880240651 -0.2914840073
N 2.4013239388 -0.3112555697 -0.606803114
N 0.4813418411 1.6935791401 -0.0252236685
O 0.1103192407 0.147292488 -2.5492468048
C 2.9049281036 2.0692986249 -0.2360232714
C 3.2679356884 0.7602159432 -0.5149291747
C 4.5726266428 -1.0239841016 -0.9432906119
H 5.3921613599 -1.7066347754 -1.1282463389
C 0.4606523643 -0.3247421356 -4.8518129281

C 2.7371713119 -2.7163468132 -1.0106736228
C 1.6051203532 2.4977507278 -0.0152486543
C 3.1927634688 -1.4147151586 -0.8600531446
C 4.6191923721 0.322401255 -0.7289114145
H 5.4842617814 0.9727961129 -0.7034982987
C 0.6663212348 -0.5251828117 -3.4149457534
H 1.3580668714 -1.3315298432 -3.1230641664
C -0.5856173153 2.5373511715 0.2105843337
C 1.2351846078 3.8602429069 0.2517986385
H 1.9361066309 4.6820128247 0.3242153976
C -0.1215178802 3.8850717491 0.3886271783
H -0.7632278656 4.7303020337 0.6014052134
C -0.3936200611 0.6884818498 -5.3204558238
C -0.5802768409 0.8538011855 -6.6877471481
C 1.1223885993 -1.1658297555 -5.759244721
H 1.7801882209 -1.9469017795 -5.3857737268
C 0.9309325066 -0.9976302844 -7.1291037603
H 1.4397698307 -1.6485649627 -7.8343751261
C 0.0807132281 0.0109471178 -7.5904262866
H -0.0699971411 0.1426846663 -8.6589348801
N -1.5767160663 -0.2484120511 -0.1184760105
N 0.3401084238 -2.2579459261 -0.6895768597
C -2.0757946564 -2.6385998791 -0.4231906879
C -2.4391314826 -1.3251072855 -0.1744703462
C -3.7443616716 0.4575747016 0.2576440358
H -4.5590342986 1.1357253651 0.4759790158
C -1.9158280187 2.1544908689 0.2834774527
C -0.7783756426 -3.0658381701 -0.6594163419
C -2.3678826058 0.8529777861 0.1394606301
C -3.7880855934 -0.8909400889 0.0645628448
H -4.6470659597 -1.5491712331 0.0889562876
C 1.4083113595 -3.1030611839 -0.919499866
C -0.4075933184 -4.4337167991 -0.89457872
H -1.1051304354 -5.2613279356 -0.9152873984
C 0.9462751642 -4.45699892 -1.0564695805
H 1.5898670209 -5.3083528342 -1.237737993
O 0.7314217455 -0.7557733518 1.7524309008
C 1.8650375593 -0.6608988577 3.834140975
C 1.4408987866 -0.1268065005 2.5592574171
H 1.8068124681 0.8718368199 2.2813370262
C 1.3798283822 -1.9004987028 4.2967415253
C 1.8131513231 -2.3908972511 5.5213632781
C 2.7776845003 0.0774682664 4.6103830949
H 3.1338682278 1.040207538 4.2517455267
C 3.2170391328 -0.4255379634 5.8301476377
H 3.9232082256 0.1375180647 6.432185972
C 2.7343400373 -1.6580908504 6.2835288708
H 3.0713116811 -2.0474942645 7.2399611223
H -1.2394516104 1.6350580065 -7.0552885072
H -0.8961591262 1.3275821273 -4.6010635062
H 0.6601589436 -2.4435244734 3.6937349387
H 1.439198936 -3.340403838 5.8920088096
C -1.9664707107 -0.2292666695 4.6030280047
C -0.9912690964 0.8900929953 4.7356147887
C -0.7842370674 1.7627133659 3.7344896861
H -1.3569352086 1.7176969238 2.815308559
H -0.0650178976 2.5735215555 3.8266513843
C -0.2513451465 1.009916883 6.0450532517
H -0.9414040997 1.2114047681 6.8735903463
H 0.2752116904 0.0793596493 6.2874920102
H 0.4814066559 1.821476399 6.0175571258
C -2.7417818187 -0.6223683803 5.8392626902
H -2.0774273927 -1.0060094769 6.6236223814
H -3.274175282 0.2351761829 6.2697440549
H -3.4759517785 -1.4018520704 5.6144048776
C -2.1301733966 -0.8821034708 3.4405108799
H -1.5439780789 -0.6503482276 2.5590006124
H -2.8517829926 -1.6906221789 3.3491490684
H 3.4757605563 -3.4899344629 -1.1916967891
H -2.651470634 2.9240490523 0.4900343352
H 3.6946319242 2.8117647452 -0.1888630384
H -2.861291106 -3.3868985084 -0.4228140882

^{4}TS1A$_{endo}$ under OEEF = -0.003 AU

Fe -0.0406930576 0.0042179868 0.0151717437
N 1.9725614746 -0.0202310506 -0.1518952438
N -0.0184183258 2.0120287682 -0.1340055095
O -0.0857551069 0.0444665734 -2.3906625926
C 2.4080955106 2.399532007 -0.235651897
C 2.8125155446 1.0725381047 -0.2068377205
C 4.1738833818 -0.718711748 -0.1008553022
H 5.0137993612 -1.3993316006 -0.0451731381
C 0.7785881253 -0.6555682758 -4.4952934182
C 2.3677605243 -2.4443444173 -0.0201769296
C 1.0886979695 2.8294377114 -0.1953689422
C 2.7948941779 -1.1260266852 -0.0822828977
C 4.185085485 0.6420522502 -0.1842733853
H 5.0367282501 1.3099827403 -0.2106035169

C 0.6643935939 -0.6848115305 -3.0308490241
H 1.3066437036 -1.4121994657 -2.5068519694
C -1.1095503995 2.8522875551 -0.0956160689
C 0.6837335909 4.2108398398 -0.1929879855
H 1.3672451043 5.0497316692 -0.2280446131
C -0.677613277 4.2249205792 -0.1263442795
H -1.3440917293 5.0776738206 -0.0993032377
C 0.0156848715 0.245336399 -5.2570578996
C 0.1433967894 0.2583729409 -6.641357487
C 1.6664635434 -1.537448142 -5.1287477588
H 2.2536693723 -2.2292088432 -4.5293617487
C 1.7917750946 -1.5218751117 -6.5167754923
H 2.4793556795 -2.2037391609 -7.0091062398
C 1.0303825249 -0.6247129334 -7.2702400519
H 1.127967729 -0.6109990258 -8.3529078751
N -2.0453328037 0.0199550156 -0.0872263829
N -0.0524413427 -2.0083717711 -0.1025048638
C -2.4800569047 -2.4001059949 -0.0894611492
C -2.8835450516 -1.0726381441 -0.0848041499
C -4.2440308177 0.7218003756 -0.0341808409
H -5.0834445733 1.4048250638 -0.0032451588
C -2.4361994825 2.4481036579 -0.0499894505
C -1.1604509197 -2.8271824662 -0.0837284296
C -2.8637508442 1.1279868116 -0.0595052953
C -4.2565278757 -0.6415922518 -0.0539975425
H -5.1084218066 -1.3096842597 -0.0425290376
C 1.0401759582 -2.8477075549 -0.0351697788
C -0.7550862108 -4.206930495 -0.0223304812
H -1.4392435524 -5.0455701621 0.0051023977
C 0.6074689037 -4.2192955042 0.0133546113
H 1.274516617 -5.0697962324 0.0745101819
O -0.0052973639 -0.0029481944 1.9928364882
C 1.8983636695 -0.4180795323 3.3904450469
C 0.9257288642 0.5145529713 2.7471493015
H 1.3888065671 1.4386873835 2.3734309159
C 1.6266354105 -1.79169774 3.4405056813
C 2.5483167345 -2.6650560518 4.0171345231
C 3.0997714119 0.0706328985 3.9187437146
H 3.3140372372 1.1365099197 3.8753603641
C 4.0195033188 -0.803397534 4.4967446356
H 4.94816534 -0.418743244 4.9085684954
C 3.7439064722 -2.1729928937 4.5487488302
H 4.4578095158 -2.8530750314 5.0046120945
H -0.4437289039 0.9539361412 -7.2346175161
H -0.6632928939 0.9207270022 -4.7457691942
H 0.6992756695 -2.1581615144 3.0122927804
H 2.3339275419 -3.7297616587 4.0550074155
C -1.8494608082 -0.1208040385 4.4263974673
C -0.630138347 0.510360462 4.8962443849
C 0.0333671502 1.5020371463 4.1557416995
H -0.5355033237 2.1446662423 3.4905517478
H 0.8618025843 2.0055082188 4.6477348591
C 0.0505619254 -0.063569749 6.0962269448
H -0.6278581287 -0.0785650423 6.958364309
H 0.3325637793 -1.1089482726 5.9049343601
H 0.9509100913 0.4893931777 6.3680783197
C -2.4141547116 -1.2969597554 5.1906629482
H -1.6936630558 -2.1211765668 5.2522489364
H -2.6743194489 -1.0219027337 6.2194772638
H -3.3194788515 -1.6752979638 4.7085820084
C -2.423353467 0.2805363243 3.2637010771
H -2.0989588725 1.1465279468 2.7058575562
H -3.2732680395 -0.2548080652 2.8500923664
H 3.1270909421 -3.2159542953 0.0491650932
H -3.1963898256 3.2217429538 -0.0187594917
H 3.1822311024 3.1590559457 -0.2712934048
H -3.2541580857 -3.1602819002 -0.0717961708

^{4}IIIA$_{endo}$ under OEEF = -0.003 AU

Fe -0.1211434827 0.0435307954 0.0117705752
N 1.8706313526 -0.1369705948 0.0346102285
N 0.0623215265 2.0359054612 -0.0243044619
O -0.0230639986 0.1037681244 -2.3042159696
C 2.5145186955 2.2372300631 -0.0322944773
C 2.8032143841 0.8834509868 0.0260633019
C 3.9987974847 -1.0118590086 0.242038508
H 4.7734124184 -1.7559526643 0.3728540767
C 0.7363254586 -0.7561307789 -4.3877007224
C 2.0644979158 -2.5858555095 0.1984961275
C 1.2329466872 2.7680181644 -0.0396846582
C 2.595555842 -1.3061499175 0.1653434755
C 4.1281959713 0.3417216097 0.1449862738
H 5.0319029712 0.9362243751 0.1785111321
C 0.6260148901 -0.7348475136 -2.927279816
H 1.1635622223 -1.526643865 -2.3814552791
C -0.9608474644 2.9624279206 0.0115818859
C 0.9366636555 4.1734986841 -0.0095875941
H 1.6837743949 4.9569885579 -0.0059221931

C -0.4212030843 4.2935911709 0.0338002773
H -1.0176370764 5.1958823218 0.0749527031
C 0.0984795185 0.2200583675 -5.1727867569
C 0.219457936 0.1767241817 -6.5566285552
C 1.4913862444 -1.7699686812 -4.9973660428
H 1.9803100336 -2.5196869834 -4.3800027887
C 1.6089152423 -1.8100921967 -6.3851238956
H 2.1920015125 -2.5939558723 -6.8597553083
C 0.9735362683 -0.8374182749 -7.161369736
H 1.065467479 -0.8674203095 -8.2441170293
N -2.1054212182 0.2193687039 -0.1688696311
N -0.2973280774 -1.9541351857 -0.0775789045
C -2.7355449035 -2.1535133074 -0.3414267108
C -3.0282633172 -0.7986447201 -0.3093433326
C -4.2416645528 1.0963081281 -0.227009315
H -5.025887928 1.8422126792 -0.2044248817
C -2.3148642256 2.6673143916 -0.011277857
C -1.4623129943 -2.6862237934 -0.2070371207
C -2.8393305274 1.3888828521 -0.1216354179
C -4.3579155152 -0.2566790872 -0.3541462347
H -5.2580502235 -0.8496705054 -0.4528131551
C 0.7159112918 -2.8821229218 0.0743285551
C -1.1718834828 -4.0915260378 -0.1375477509
H -1.9165087406 -4.8745889664 -0.2031244328
C 0.1745653706 -4.2122958414 0.045907855
H 0.7611694183 -5.1151586197 0.1573346911
O -0.2241894287 -0.0158409533 2.1703384351
C 1.9283952876 -0.0697012009 3.330073275
C 0.7025183877 0.7538639562 2.9903420101
H 1.0010530352 1.5990766746 2.3666036824
C 1.8879731155 -1.4637434974 3.4301042188
C 3.0270287403 -2.1784639643 3.8057530192
C 3.1301524663 0.5967048105 3.6018275838
H 3.1720231735 1.6811269078 3.5223699895
C 4.2683112617 -0.1151454839 3.9799294332
H 5.1912631939 0.4161181213 4.1958524379
C 4.2190291492 -1.5075787506 4.0840963357
H 5.1021899466 -2.0639461657 4.3857098555
H -0.2698926039 0.9291648411 -7.1686537008
H -0.4795046386 0.9959639162 -4.6803727818
H 0.9663073452 -1.9881180879 3.2058819935
H 2.9793579036 -3.2612031862 3.8880553361
C -1.3662125472 -0.7311795984 4.270189934
C -0.667973424 0.1509480986 5.0045583562
C -0.0268159043 1.2857977486 4.2353785149
H -0.7818524115 2.0160700329 3.9084993382
H 0.6968175339 1.8295380305 4.8497865489
C -0.4044807421 0.0883653555 6.4798214131
H -0.9441661469 -0.7157004378 6.9844777405
H 0.6686550314 -0.0541809305 6.667278606
H -0.6836669108 1.0347793316 6.9614347823
C -2.0757500784 -1.9680698051 4.7478205978
H -1.6395732363 -2.8680183934 4.2906744825
H -2.0316796108 -2.0909579396 5.8316492999
H -3.136433018 -1.9511779559 4.4597024422
C -1.4762485938 -0.4605712005 2.7929743292
H -2.2277940847 0.3098003254 2.5783395369
H -1.7512814752 -1.3615872782 2.2434172949
H 2.754571102 -3.4128852012 0.3253881499
H -3.0131498638 3.4967786303 0.027921331
H 3.3478055766 2.931965205 -0.0281277012
H -3.5648739045 -2.8468903384 -0.4335997576

[4]VA under OEEF = -0.003 AU

Fe -0.0081373977 -0.0205560469 -0.00969661
N 1.9586467643 -0.0175829566 -0.3659749452
N -0.0258258524 1.9707487444 -0.2074061007
C 2.3805121127 2.4064266188 -0.4705534792
C 2.7888017226 1.0846639704 -0.4924115473
C 4.1621621041 -0.6958890037 -0.5701112867
H 5.0070215699 -1.3703095851 -0.6240754132
C 2.3982408086 -2.4407927925 -0.3587132076
C 1.0654271462 2.8126876214 -0.3323327657
C 2.7993989783 -1.1172518919 -0.420618478
C 4.1549414003 0.6668159363 -0.6211079629
H 4.9928706566 1.3445672015 -0.7202920608
C -1.119153417 2.8071931932 -0.0627814648
C 0.6480692081 4.183733849 -0.2681694236
H 1.3200084972 5.0296359933 -0.334690299
C -0.7042123708 4.1803722804 -0.0948604335
H -1.3757115939 5.0232936081 0.0051524116
N -2.0114388604 -0.0222416917 -0.061886946
N -0.0211204096 -2.0135273311 -0.218665680
C -2.441241564 -2.4447990334 -0.0775074033
C -2.8481133213 -1.1228157268 -0.026237900
C -4.2130048877 0.6551754458 0.1614639488
H -5.0527303791 1.3297090556 0.2678121526
C -2.4337962587 2.3970099976 0.0748911693

C -1.1215733036 -2.8533876678 -0.165461879
C -2.8441463342 1.0753916892 0.0600893897
C -4.2160510588 -0.7063760047 0.1018318471
H -5.0585865205 -1.384059598 0.1518916297
C 1.0789322569 -2.8532179948 -0.2817691706
C -0.7026598493 -4.2248239558 -0.198808236
H -1.3781797621 -5.0700352019 -0.166912710
C 0.6595202562 -4.2249867316 -0.2669587539
H 1.3347368393 -5.0703166839 -0.3031824412
O -0.0136614852 -0.0195131839 2.040912451
C 0.0561820784 -0.886547164 4.2452937619
C 0.3465290219 -0.9158687885 2.8354308036
H 0.8725208322 -1.7950057748 2.4400456996
C -0.5504092126 0.2401014039 4.8398460052
C -0.8189474952 0.2320285676 6.2012614042
C 0.3900422125 -2.0095915219 5.0284789632
H 0.8666758193 -2.8681252303 4.5620626564
C 0.1065755802 -2.0143295768 6.3887706693
H 0.3560965796 -2.8778307938 6.9971299255
C -0.495968088 -0.8942889628 6.9728485759
H -0.712295437 -0.8952676458 8.0372789316
H 3.1668633695 -3.2051280307 -0.3968447322
H -3.206620851 -3.2120185557 -0.0334467525
H -3.194020991 3.1622434542 0.18806708
H 3.1428164302 3.1734033346 -0.5513674245
H -0.7845879486 1.102643459 4.2251339687
H -1.2793873819 1.0950805305 6.6718824192
C 2.6872004604 2.056742503 4.0624059868
C 3.0789314809 0.620356348 4.1335217359
C 3.3460326212 -0.0895114072 3.0234975349
H 3.3407152272 0.3694003212 2.0421699253
H 3.626351649 -1.1391438254 3.0748321357
C 3.1701688698 -0.0027525499 5.5045393011
H 3.9664592311 0.4619597149 6.0988452274
H 2.2359262989 0.1322441336 6.0620117812
H 3.3821963485 -1.0740014878 5.4443685656
C 3.2033772236 2.9692094132 5.1505819413
H 2.8059061513 2.6842448752 6.1326387721
H 4.2970784384 2.9224478097 5.2282934276
H 2.9187897134 4.0091803392 4.9645270488
C 1.8796137658 2.5121607762 3.0907060046
H 1.4569980985 1.8577466272 2.3377107986
H 1.597794208 3.5613755813 3.0384994659

^{4}TS2A$_{endo}$ under OEEF = -0.003 AU

Fe 0.1106954305 -0.1396310545 -0.0196551716
N 2.0423583019 0.3183725824 -0.3519730454
N -0.3448793402 1.8135803265 -0.1417943411
C 1.9163761018 2.7735698934 -0.2772048075
C 2.6109807522 1.5779357737 -0.35812316
C 4.346445208 0.1503616862 -0.4745527868
H 5.3205967544 -0.3183336832 -0.5297891418
C 2.99787142 -1.9474548268 -0.439170199
C 0.5385595658 2.8750896424 -0.173797732
C 3.101733851 -0.5661405098 -0.4209753171
C 4.0423622609 1.4781778113 -0.4398526928
H 4.7151403731 2.3260883269 -0.4584719407
C -1.5989195979 2.3783368188 -0.0090568986
C -0.1728273496 4.1183930769 -0.060351394
H 0.2954552103 5.0943371226 -0.0602770317
C -1.4963483028 3.8101694763 0.048331693
H -2.339505519 4.4806317322 0.1542898356
N -1.8500179426 -0.5783521471 -0.1849606083
N 0.5427910874 -2.0759131949 -0.3481397047
C -1.7245345281 -3.0319417839 -0.2793299367
C -2.4189429457 -1.8360490863 -0.2077054358
C -4.1510816231 -0.4134579904 -0.0136639646
H -5.1225120017 0.0535144352 0.0863654855
C -2.7952530689 1.6806407031 0.0397700117
C -0.3450615772 -3.1364106619 -0.347222014
C -2.9051842414 0.3031551722 -0.0558758115
C -3.8497289096 -1.7383645932 -0.1089464857
H -4.5216318883 -2.5868926041 -0.09978913
C 1.8008909435 -2.6454895775 -0.4163876144
C 0.3679719893 -4.3809491117 -0.4144365066
H -0.102652837 -5.3556401653 -0.4221266693
C 1.6965312776 -4.077440899 -0.4577539478
H 2.5416219941 -4.7520241439 -0.5091390747
O 0.170839289 -0.2170618614 1.9145373854
C -0.9400991862 -1.8166084023 3.3132330784
C 0.2907751073 -1.2502111499 2.7005450012
H 1.0297706301 -2.0044090101 2.4012127075
C -2.1460157374 -1.1049080649 3.2530368043
C -3.3070958237 -1.6537449992 3.7954040375
C -0.9071195336 -3.0782135062 3.9236083739
H 0.0282336954 -3.6323277253 3.9669203222
C -2.0676838719 -3.6215108229 4.4711950897
H -2.0372679309 -4.5968345255 4.9481163342

C -3.2696963479 -2.909363876 4.408612816
H -4.1728965996 -3.3325357667 4.8387339614
H 3.91738321 -2.5214148471 -0.4858842645
H -2.2999249616 -3.9514165461 -0.2715831065
H -3.7100510602 2.2527940505 0.1527834683
H 2.4909025461 3.6938606694 -0.2839595694
H -2.1620337936 -0.1373234741 2.76305582
H -4.2419058368 -1.1029173428 3.7399484125
C 0.5395388038 1.63082767 4.3514906666
C 0.7351982654 0.2768551055 4.8460162634
C 1.5020541679 -0.6721185742 4.160910729
H 2.3004942994 -0.3514997368 3.4990359046
H 1.6939027189 -1.6127552633 4.669944305
C -0.0661833175 -0.17175722 6.0252234981
H 0.0972707947 0.4918449427 6.883451154
H -1.1400966724 -0.1203783858 5.795075732
H 0.1733036666 -1.1936996633 6.3224407354
C -0.4028898496 2.5506013791 5.094175133
H -1.4077128648 2.1207371305 5.1783958294
H -0.051144804 2.7430848175 6.1146054772
H -0.4888523879 3.5140755188 4.584503986
C 1.1312264276 2.0422704991 3.202492397
H 1.8494572236 1.4523668775 2.6501156748
H 0.907238885 3.021263557 2.7889413805

^{6}TS2A$_{endo}$ under OEEF = -0.003 AU

Fe 0.0704927257 -0.0116850953 0.0153558339
N 2.0675076437 -0.1874581563 -0.5561496762
N 0.161425932 1.9784647985 -0.5818935743
C 2.5990832839 2.1999586821 -0.8434280143
C 2.9508643893 0.8541702941 -0.7561507301
C 4.2112039141 -1.0189684568 -0.7268233796
H 5.014798251 -1.7441913628 -0.751983369
C 2.3109381763 -2.6299219982 -0.3628806356
C 1.3077009387 2.7201844868 -0.77499287
C 2.8201315491 -1.343641049 -0.5393972859
C 4.2917046476 0.3375330571 -0.8616400351
H 5.1750699721 0.9432656129 -1.0195077131
C -0.8906186683 2.8699638076 -0.5819511017
C 0.9675294022 4.1156309773 -0.8881299135
H 1.6828791621 4.9142453019 -1.0387748579
C -0.3899912885 4.2080178853 -0.7699797724
H -1.0067256482 5.0972297811 -0.80673497
N -1.9887527976 0.0919110161 -0.2270608444
N -0.0805055599 -2.0747855849 -0.2110823974
C -2.5197905657 -2.2972529379 0.0353811501
C -2.8730162027 -0.9524181257 -0.0616621037
C -4.1402524461 0.9141505932 -0.1314334784
H -4.9468435032 1.6363874569 -0.1302016686
C -2.2337284584 2.5308152051 -0.4275002835
C -1.2294899857 -2.8171296356 -0.035155342
C -2.7436436361 1.2438154639 -0.2704981101
C -4.2199112808 -0.4420091451 -0.0021128642
H -5.1048875171 -1.0520593871 0.1265964506
C 0.9679268607 -2.9696877871 -0.2161691937
C -0.8925217389 -4.214701044 0.0697169314
H -1.6103205168 -5.01173655 0.2158789464
C 0.4639411064 -4.3091454646 -0.0414618283
H 1.0789129318 -5.1994194386 -0.0050300789
O 0.2366469831 0.070420073 1.901627416
C 0.3416679197 -1.5322928532 3.6802004112
C 0.9969780611 -0.6528374044 2.6871840913
H 1.9024855825 -1.0689042104 2.2312032273
C -1.0267713514 -1.3946357276 3.9594360941
C -1.6350625519 -2.2383686616 4.8868215115
C 1.0912246988 -2.5196662867 4.3374148763
H 2.1518915358 -2.6266414299 4.1207745855
C 0.4802237126 -3.3583109967 5.2665398625
H 1.0630982509 -4.1198763331 5.7759195168
C -0.8832389521 -3.2170758018 5.5441819422
H -1.3576747244 -3.8681825279 6.2724843191
H 3.0282024715 -3.444979389 -0.3518238614
H -3.3273847437 -3.0082958775 0.1784043929
H -2.9511513217 3.3457035554 -0.4392338717
H 3.4065920708 2.9101337053 -0.9923115384
H -1.5995454905 -0.6354516234 3.436294886
H -2.6947115367 -2.1329108255 5.1012712097
C 0.3172396033 2.3557066559 3.9745329292
C 1.2157357885 1.3261509344 4.4837015355
C 2.1509463101 0.6763886168 3.6741242188
H 2.5202939087 1.1521794987 2.7714776063
H 2.8642240608 0.0180462301 4.1623968451
C 0.999879485 0.8191110748 5.8716019012
H 1.0264943946 1.6421737888 6.5971632285
H 0.0017013616 0.3657389111 5.9561669199
H 1.74140607 0.0730894313 6.1600724744
C -0.790933932 2.8678074367 4.8654840142
H -1.4543385463 2.0592653303 5.1934035402

H -0.3939384933 3.3415437623 5.7706068942
H -1.3980290124 3.6107170443 4.3409243756
C 0.4334028334 2.8064559847 2.7026159849
H 1.2317751515 2.5122094947 2.0355146922
H -0.2793806705 3.518915219 2.2990705483

^{4}VIA$_{endo}$ under OEEF = -0.003 AU

Fe -0.0116700252 0.0053851409 0.0116858765
N 1.9815581552 -0.0076814836 -0.1946746536
N 0.0056013235 1.9974548589 -0.1992126796
C 2.4291717678 2.4074559034 -0.0443163957
C 2.8293599135 1.0839851452 -0.1068805198
C 4.1881230467 -0.7034144898 -0.2514265392
H 5.0284718723 -1.3837604402 -0.3026353919
C 2.3907232561 -2.4285971386 -0.3480259606
C 1.1123729503 2.827762063 -0.1182438291
C 2.8135413192 -1.1115103543 -0.2829725313
C 4.197872792 0.6552374589 -0.1315500601
H 5.0479362385 1.3224864259 -0.0704548734
C -1.08399027 2.848666534 -0.2874498414
C 0.7075692137 4.2030641307 -0.1532641108
H 1.3896611654 5.0417482045 -0.0989753091
C -0.6511732413 4.215878187 -0.2696312712
H -1.3169943194 5.0675431769 -0.3241947783
N -2.0013314792 0.0303564381 -0.2075970458
N -0.0252173554 -1.9720622306 -0.2368646543
C -2.4484867312 -2.3855974217 -0.1050048939
C -2.8495339167 -1.061884874 -0.1446618266
C -4.2076699954 0.7275762951 -0.2635412364
H -5.047415787 1.4092405763 -0.3067302705
C -2.4100151505 2.4532783234 -0.3336600719
C -1.1314712103 -2.8024866857 -0.1725185156
C -2.8321074566 1.1360204399 -0.2816370639
C -4.2182718043 -0.6324974884 -0.1678518008
H -5.0684899156 -1.3007827081 -0.1222167421
C 1.0655668269 -2.8224500647 -0.3139997342
C -0.7262579007 -4.1776304956 -0.2076473671
H -1.4084436628 -5.0155065644 -0.1512592109
C 0.6323880609 -4.1901443902 -0.3066657081
H 1.2997360172 -5.0411488538 -0.3459079094
O -0.0370601583 -0.0129443495 2.0926055335
C 0.2545828823 -2.246741984 3.0436028573
C 0.8737732339 -0.8802788919 2.8450875103
H 1.7643814253 -0.9737215362 2.2190869814
C -1.1241776275 -2.4291070772 3.1916985306
C -1.6424472609 -3.6988127915 3.4539837432
C 1.1047281916 -3.354615329 3.1518947378
H 2.1779679742 -3.2215023432 3.0335727233
C 0.5885960851 -4.6219432326 3.420929635
H 1.2607758552 -5.4705175627 3.5133796001
C -0.7889091293 -4.7969856706 3.5737078321
H -1.1929137966 -5.7819466036 3.7902235048
H 3.1483694425 -3.203053315 -0.399078713
H -3.2175334927 -3.1476004728 -0.0434771952
H -3.1681695334 3.2272641086 -0.3851469866
H 3.1998906272 3.167187277 0.0272781323
H -1.7887570461 -1.5777086163 3.1007461406
H -2.7147870608 -3.8265604781 3.5752712249
C -0.9193818298 0.8701761458 4.2340727881
C 0.0135013733 0.2062421395 4.939048593
C 1.253465655 -0.1957612478 4.1690694566
H 1.8764187624 0.6820820025 3.9425505871
H 1.8764604073 -0.8900913234 4.7400314512
C -0.0783356635 -0.2319717301 6.370014429
H -0.9685423804 0.1398204728 6.8817884452
H -0.08513212 -1.3289426998 6.429313665
H 0.801178023 0.1064343478 6.9332049953
C -2.25963559 1.3579568163 4.7107054108
H -3.072784198 0.857752391 4.165008527
H -2.4164113387 1.1828424574 5.7767912755
H -2.3753935464 2.435778908 4.5315271088
C -0.5943855975 1.1580036049 2.7943884643
H 0.1369853953 1.9681723801 2.6827593324
H -1.4849916612 1.4195905852 2.2230465981

^{6}VIA$_{endo}$ under OEEF = -0.003 AU

Fe -0.0337734922 -0.008174057 0.1342211846
N 1.9999882878 -0.0581049162 -0.2192802407
N 0.0231115024 2.0299246044 -0.2056061904
C 2.4647776385 2.358210891 -0.1466911668
C 2.8594758521 1.0235692357 -0.1796546283
C 4.1827008626 -0.8019978819 -0.2719874142
H 5.0133813038 -1.4954862924 -0.3060526432
C 2.3248834441 -2.4957246004 -0.3249248705
C 1.1538279414 2.8260708704 -0.180071443
C 2.7964064551 -1.186008041 -0.2826361477
C 4.221624172 0.5618715243 -0.2042146012
H 5.0905349573 1.2069118245 -0.1745763002

C -1.0564006509 2.8919604476 -0.2654114224
C 0.7727657738 4.212480714 -0.2147037486
H 1.4682940971 5.0419295849 -0.1974537064
C -0.5911494719 4.2530179925 -0.2734651041
H -1.2350159867 5.1225314734 -0.310131244
N -2.0676953439 0.065426208 -0.2042925784
N -0.0907715936 -2.022448452 -0.2714554649
C -2.5303675433 -2.3525619692 -0.197553290
C -2.9262228698 -1.0175372149 -0.195217719
C -4.2511193698 0.8079010366 -0.2566282746
H -5.0822827878 1.5011359082 -0.2823628019
C -2.3936399757 2.5031842229 -0.2821006412
C -1.2204297095 -2.8183840671 -0.243408841
C -2.8650199193 1.1934504429 -0.2518233912
C -4.288728335 -0.5568474338 -0.2158985653
H -5.1567708534 -1.203619307 -0.2048876216
C 0.9891228687 -2.883990898 -0.3156322799
C -0.8387689985 -4.2049275111 -0.269462834
H -1.5338384923 -5.0338899166 -0.238598687
C 0.5243205773 -4.2455575032 -0.3176976643
H 1.1692590388 -5.1145761151 -0.3325119012
O -0.0056822394 0.0291304452 2.1564325634
C 0.3686524727 -2.2041346954 3.0833259932
C 0.9302188141 -0.8089026394 2.9279948322
H 1.8415251638 -0.8430823794 2.3252325534
C -1.0048519083 -2.4540446284 3.1584044243
C -1.4719145163 -3.7501429627 3.3857494023
C 1.267801141 -3.2690512647 3.2227307693
H 2.3376061959 -3.0812154058 3.1596152727
C 0.8016762429 -4.5622683437 3.4564199013
H 1.5093156717 -5.3779867895 3.5753875623
C -0.5715301635 -4.8052281142 3.5411346697
H -0.9359753265 -5.8111038111 3.7296291668
H 3.0674423571 -3.2868181788 -0.3535873614
H -3.3173427992 -3.0996647848 -0.176532643
H -3.1361509369 3.2942580049 -0.3150783036
H 3.2526633819 3.1043062455 -0.1182359372
H -1.7054727635 -1.6354234014 3.0397241203
H -2.5412339458 -3.9322776184 3.4493950786
C -0.9915084827 0.8539223299 4.2772253366
C -0.0518064027 0.2208993673 5.002401741
C 1.2291199946 -0.1148907502 4.2675968468
H 1.8143392519 0.794215933 4.0658946798
H 1.8694464673 -0.7812229301 4.8525505297
C -0.1680236068 -0.2361095451 6.4255160629
H -1.0874720582 0.0943551614 6.9130871595
H -0.1304466038 -1.3327536097 6.4756331083
H 0.6793857861 0.1339562283 7.0170893703
C -2.3672868715 1.2754310428 4.7141984805
H -3.1386617171 0.7484574093 4.1342599236
H -2.5508212874 1.077474485 5.7718115679
H -2.5244063773 2.3496861021 4.5462119718
C -0.6354623427 1.1776501849 2.8546267466
H 0.0737220888 2.0090232525 2.7686271397
H -1.5169040593 1.4082948565 2.2563995147
